# Multiscale model of the different modes of cancer cell invasion

**DOI:** 10.1093/bioinformatics/btad374

**Published:** 2023-06-08

**Authors:** Marco Ruscone, Arnau Montagud, Philippe Chavrier, Olivier Destaing, Isabelle Bonnet, Andrei Zinovyev, Emmanuel Barillot, Vincent Noël, Laurence Calzone

**Affiliations:** Institut Curie, Université PSL, F-75005 Paris, France; INSERM, U900, F-75005 Paris, France; Mines ParisTech, Université PSL, F-75005 Paris, France; Sorbonne Université, Collège Doctoral, F-75005 Paris, France; Barcelona Supercomputing Center (BSC), Barcelona, Spain; Institut Curie, PSL Research University, CNRS, UMR 144, Paris, France; Institute for Advanced Biosciences, Centre de Recherche Université Grenoble Alpes, Inserm U 1209, CNRS UMR 5309, France; Institut Curie, Université PSL, Sorbonne Université, CNRS UMR168, Laboratoire Physico Chimie Curie, Paris, France; Institut Curie, Université PSL, F-75005 Paris, France; INSERM, U900, F-75005 Paris, France; Mines ParisTech, Université PSL, F-75005 Paris, France; Institut Curie, Université PSL, F-75005 Paris, France; INSERM, U900, F-75005 Paris, France; Mines ParisTech, Université PSL, F-75005 Paris, France; Institut Curie, Université PSL, F-75005 Paris, France; INSERM, U900, F-75005 Paris, France; Mines ParisTech, Université PSL, F-75005 Paris, France; Institut Curie, Université PSL, F-75005 Paris, France; INSERM, U900, F-75005 Paris, France; Mines ParisTech, Université PSL, F-75005 Paris, France

## Abstract

**Motivation:**

Mathematical models of biological processes altered in cancer are built using the knowledge of complex networks of signaling pathways, detailing the molecular regulations inside different cell types, such as tumor cells, immune and other stromal cells. If these models mainly focus on intracellular information, they often omit a description of the spatial organization among cells and their interactions, and with the tumoral microenvironment.

**Results:**

We present here a model of tumor cell invasion simulated with PhysiBoSS, a multiscale framework, which combines agent-based modeling and continuous time Markov processes applied on Boolean network models. With this model, we aim to study the different modes of cell migration and to predict means to block it by considering not only spatial information obtained from the agent-based simulation but also intracellular regulation obtained from the Boolean model.

Our multiscale model integrates the impact of gene mutations with the perturbation of the environmental conditions and allows the visualization of the results with 2D and 3D representations. The model successfully reproduces single and collective migration processes and is validated on published experiments on cell invasion. *In silico* experiments are suggested to search for possible targets that can block the more invasive tumoral phenotypes.

**Availability and implementation:**

https://github.com/sysbio-curie/Invasion_model_PhysiBoSS.

## 1 Introduction

Cancer is the top cause of disease burden in the world according to the World Health Organization (Mattiuzzi and Lippi 2019). The severity of cancer is greatly increased by the invasion of cancerous cells into their surrounding environment, through a metastatic process that may end up in the generation of a secondary cancer seeding (Suhail *et al.* 2019, Weiss *et al.* 2022). Cell invasion is a complex multiscale process that requires the coordinated action of entities at the cells’ molecular, cellular, and population level. In addition, cell invasion depends on the interplay between cells and the extracellular environment (Wolf *et al.* 2013): the presence of different types of collagens and molecules affects the density of the extracellular matrix (ECM). Likewise, the ECM can constrain cellular invasion (Čermák *et al.* 2018, Kim *et al.* 2018): the ECM stiffness and the availability of oxygen or nutrients impact intracellular mechanisms, and the ECM acts as a repository for a variety of growth factors (GFs) and matricellular proteins released upon ECM modification (Hinz 2015). This complex dynamic system causes a wide array of different invasion behaviors that have been described in different cancers (Guzman *et al.* 2020, Lüönd *et al.* 2021, Weiss *et al.* 2022).

Three main invasion modes have been categorized (Friedl and Wolf 2010, Friedl and Alexander 2011), where cells move either individually, collectively, or in streams of cells. Although these terms are arguably arbitrary and their description can be incomplete, notably at the molecular level, they are useful as they simplify and categorize the literature, and they facilitate the study of the molecular mechanisms underlying each mode (Friedl and Wolf 2010). Single cell or individual migration is the invasion mode where cell–cell junctions have been lost and cells are free to degrade and roam the ECM (Friedl and Alexander 2011). Depending on the tissue, this single cell mode can be further described as ameboid, where cells have low adhesion force and spherical shapes, or as mesenchymal, where cells have cytoskeletal protrusions, adhesion capabilities, and a spindle shape, with elongated morphologies and proteolytic activity toward ECM substrates. In collective migration, cell–cell adhesions are retained and cells invade as multicellular groups, requiring a certain coordination between cell–cell adhesion and migration (Vilchez Mercedes *et al.* 2021).

This mode has been identified as more aggressive and with higher metastatic potential, especially in circulating tumor cells and breast cancer (Yang *et al.* 2019b, Lee *et al.* 2021). Trail or multicellular streaming migration is a special case of the single cell invasion mode where individual cells move one after the other using the same path within the tissue (Friedl and Wolf 2010). This can be caused by a given chemoattractant or due to local ECM heterogeneities that allow for a path of less friction for cells. In this work, we will not detail this mode of invasion which may be considered as a special case of single cell migration but remains a subject of discussion. In all invasion modes, the first step of cellular invasion is the detachment of cells from their neighboring tumor tissue. The epithelial-to-mesenchymal transition (EMT) is a molecular program that triggers tumor cell invasion in response to environmental signals (Thiery *et al.* 2009) by detaching cells from their neighbors (Friedl and Alexander 2011), promoting cell–matrix adhesion and the formation of protrusion at the cell membrane (Destaing *et al.* 2011, Bergman *et al.* 2014), and secreting matrix metalloproteases (MMPs) that degrade the ECM (Ferrari *et al.* 2019). EMT allows the cell to switch to a more motile phenotype, losing adhesion to neighboring cells and promoting invasiveness at the single cell level.

To study the invasion process that spans many time and spatial scales, multiscale models, such as agent-based modeling (ABM), are ideally suited as they consider agents as surrogate of cells that move, divide, and die, which facilitates the specific description of both intracellular, secretory and microenvironmental behaviors (Metzcar *et al.* 2019).

ABM is a computational approach that has gained popularity in the field of cancer research over the last decades. It was applied in a variety of cancer studies, including breast, lung, and glioma, and used to investigate the efficacy of treatments, such as chemotherapy (Gong *et al.* 2017). Some agent-based studies have focused on describing the effect of the pressure and the interactions between the agents and the ECM (Gonçalves and Garcia-Aznar 2021) without considering intracellular pathways. Notable applications of ABM to cancer invasion (Anderson 2005, 2007, Franssen *et al.* 2019, Macfarlane *et al.* 2019, Macnamara *et al.* 2020, Sfakianakis *et al.* 2020) have underlined how modeling the interactions between cancer cells and the ECM, to simulate EMT and migration, is essential for developing new therapies that target cancer invasion and metastasis. In particular, hybrid models have emerged as a powerful tool to simulate cancer cell invasion. By combining ABM and gene regulatory networks described by partial and ordinary differential equations (Pally *et al.* 2019), hybrid models explore the spatial and temporal dynamics of cancer cell invasion in a more comprehensive and mechanistic way, highlighting the role of specific genes and signaling pathways in cancer and providing a tool to study heterogeneity at cell state and cell phenotype (Jiang *et al.* 2005, Deisboeck *et al.* 2011, Weerasinghe *et al.* 2019). However, this type of dynamical models only covers a small part of the biological mechanisms for two reasons: because of the computational cost for simulating a high number of variables and because these models require many parameters that need to be fitted to experiments (Schwab *et al.* 2020). One way to cope with this limitation is to describe the intracellular regulations with the Boolean formalism, which, although providing a less detailed description, requires no or very few parameters.

In this study, we use PhysiBoSS (Letort *et al.* 2019, Ponce-de Leon *et al.* 2022), a software that combines PhysiCell (Ghaffarizadeh *et al.* 2018), an ABM framework that integrates interactions between cells and with the microenvironment, and MaBoSS (Stoll *et al.* 2012, 2017), a tool that relies on stochastic simulations of Boolean intracellular signaling models. PhysiBoSS allows for the combined genetic and environmental perturbations of tumors and inspects their effect at the population level, enabling the study of drug treatments and cellular heterogeneity and their effect on cancer phenotypes. PhysiBoSS is used to describe a novel model of tumor growth that combines a description of the signaling pathways that trigger events leading to an invasive phenotype, focusing on both single cell and collective features. By varying the parameters of the ABM and various intracellular components, the model reproduces the different modes of invasion and provides a tool for the suggestion of potential drug treatments and genetic perturbations that could block or perturb specific invasion modes. The model can be used to reproduce multiple scenarios both from the spatial point of view (changing the initial cells’ position, the ECM density and organization, or 2D/3D visualization) and from the intracellular point of view (simulating mutations and initial conditions, as well as giving the possibility to include a different network as long as the input and output remain the same).

## 2 Materials and methods

### 2.1 PhysiBoSS—a multiscale framework combining Boolean and agent-based models

The model of the different modes of invasion uses PhysiBoSS to connect two levels of description: one at the level of the individual cell and one at the level of cell population. PhysiBoSS allows this multiscale description by combining simulation of Boolean models using continuous time Markov process (with MaBoSS), and center-based ABM of physico-chemical cell–cell and cell–environment interactions (with PhysiCell). The multiscale model presented here combines an intracellular gene regulatory network with several signaling pathways and generic cell-level parameters. The intracellular model represents interactions often deregulated in cancer: it receives diverse stimuli from the environment by activating a membrane receptor (input) that triggers signaling pathways and molecular interactions, ultimately leading to different cell responses through MaBoSS simulations. These cell fates are represented by phenotypes that, among others, include cell division (by activating the M-type cyclins), cell death (by cleaving the effector caspase 3), and EMT (see Supplementary Tables S2 and S3). Some components can be secreted by the cell, released in the cell environment, which can activate or inactivate the neighboring cells. These cell fates, in turn, affect different variables and parameters of the agent-based model and of neighboring cells (Supplementary Section S1 and Supplementary Figs S1 and S2). For instance, the release of MMPs will degrade the ECM, and subsequently free some proteins that will trigger a new signaling from neighboring cells.

### 2.2 The intracellular model

**Figure 1.**
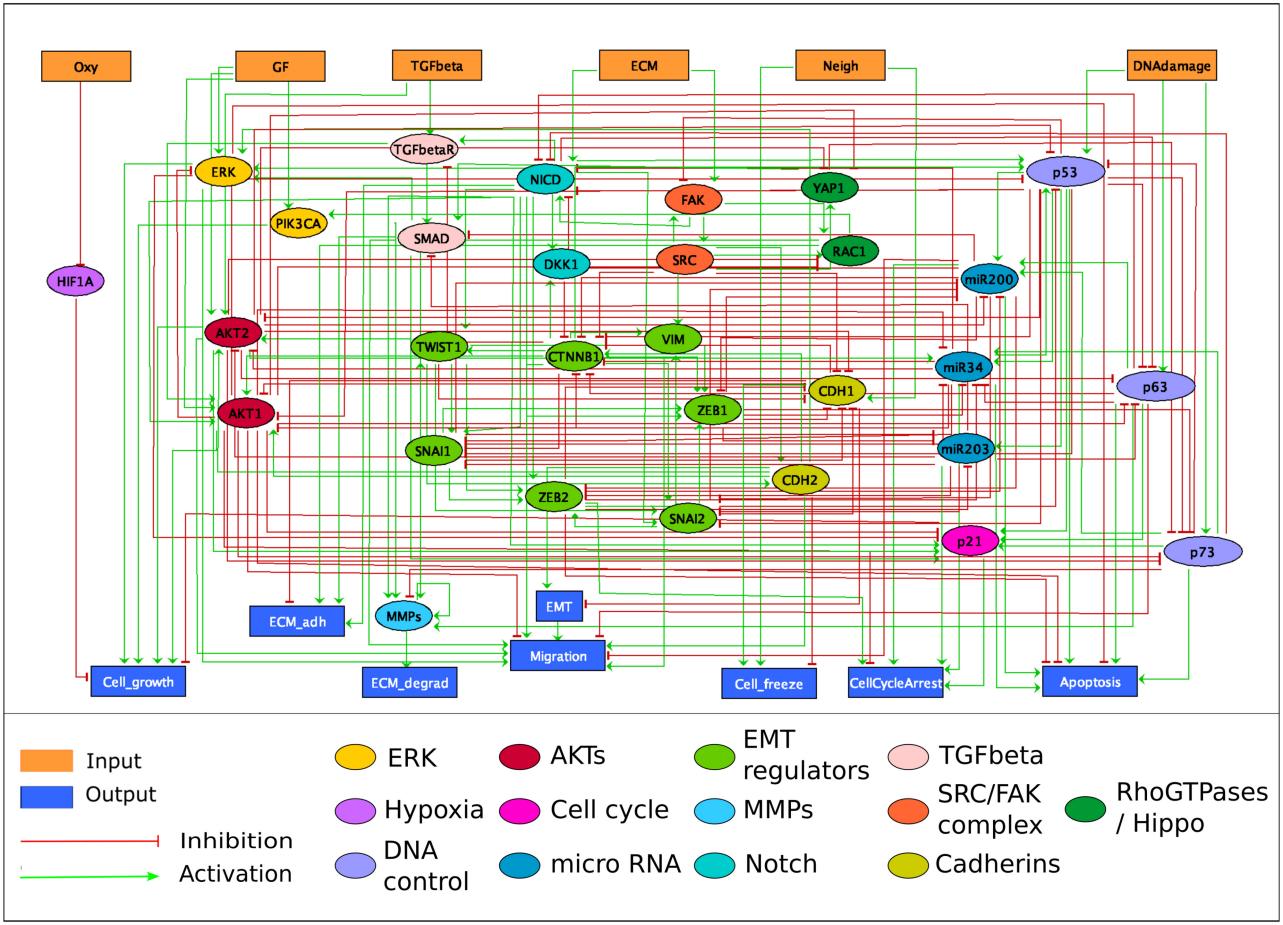
Influence network of the intracellular model, illustrating the intricate interplay between genes, proteins, inputs, and outputs. Rectangular nodes at the top represent inputs, while rectangular nodes at the bottom depict outputs (or phenotypic read-outs). Notably, the figure reveals the presence of highly connected nodes, characterized by a high number of incoming and outgoing arrows, highlighting the complexity and interconnectedness of the pathways involved.

The intracellular model builds upon two published models focused on the early steps of metastasis (Cohen *et al.* 2015) and on EMT process (Selvaggio *et al.* 2020). The initial model of Cohen and colleagues was built with two inputs: the “ECMenv,” which monitored the status of the ECM, and “DNA_damage,” which considered DNA alterations that trigger death signals. Four additional inputs were added to account for the presence of “Oxygen,” GFs, “TGFbeta,” and the contact with other neighboring cells (as “Neigh”) (Fig. 1). The phenotypes, or outputs of the model include “CellCycleArrest,” “Apoptosis,” “EMT,” “ECM_adh” (for cell adhesion), “ECM_degrad” (for cell degradation), “Cell_growth” (for the dynamics of the tumor growth), and “Cell_freeze” (for cell motility ability). New genes and pathways include mechanisms around p63 (Celardo *et al.* 2014) and SRC (Moitrier *et al.* 2019, Shaaya *et al.* 2020). Genes from the Hippo pathway and RhoGTPases, such as YAP1 (Hall *et al.* 2010), focal adhesion kinase (FAK), and RAC1 (Parri and Chiarugi 2010), were also inserted to link external signals (i.e. cell–cell contact, stiffness of the ECM, and stress signals) and intracellular regulation. Currently, the model does not include a mesenchymal to epithelial transition (MET) (Yao *et al.* 2011, Hamilton and Rath 2017), and a cell in a mesenchymal cell cannot revert its state. The resulting network encompasses 45 nodes, with 6 input nodes, representing the possible interactions of an individual cell with external elements, and 8 output nodes or read-outs describing the possible observed phenotypes (Fig. 1). The model is also provided in SIF and SBML formats. The SIF format can be used to facilitate the network visualization with Cytoscape (Shaaya *et al.* 2020) and is analyzed in a Jupyter notebook available in dedicated GitHub and Supplementary Material. The SBML format can be accessed on Biomodels https://www.ebi.ac.uk/biomodels/MODEL2304070002.

### 2.3 Initial conditions for the simulation of the intracellular model

The initial conditions of the model were set to represent a given cell’s position in the tumor: in the center of the tumor with cells surrounding it (“Oxygen” and GFs are ON), and at the periphery of the tumor in direct contact with the ECM (“Oxygen,” GFs, “ECM,” and “TGFbeta” ON with or without “DNAdamage”) (Supplementary Fig. S3). Because of the contact with the ECM, cells on the surface of the tumor are more likely to switch from an epithelial to a mesenchymal state (Tzanakakis *et al.* 2018), while in the center, they form tight cell junctions preventing movement. For the inner tumor cell conditions, the simulation with MaBoSS shows a high proportion of cells with “Cell_freeze” and “Cell_growth” phenotypes, corresponding to an increase in the tumor mass without signs of invasion. Instead, activation of “DNA_damage” leads to the activation of the apoptotic pathway. The pressure due to cell crowding activates the *DNA_damage* node, which leads to the secretion of MMPs and the remodeling and degradation of ECM (Nader *et al.* 2021). In the absence of DNA damage, a high probability of cells with the “EMT/Migration/ECM_adh” phenotypes is observed, corresponding to a high invasive potential. This is also true in the absence of oxygen and GF. The presence of DNA damage promotes secretion of MMPs. The combination of the absence of GF and DNA damage leads to an apoptotic state (see Supplementary Section S3 for MaBoSS simulations).

Different combinations of initial states can be simulated using our dedicated Jupyter notebook (included as Supplementary Material) and in the GitHub repository https://github.com/sysbio-curie/Invasion_model_PhysiBoSS.

### 2.4 Setup of the agent-based model for the integration of the intracellular model

At the beginning of the simulation, the tumor is represented as a spheroid composed of cancer cells in an epithelial state, surrounded by the ECM. The ECM is defined as a static substrate, i.e. not characterized by a diffusion coefficient and without any decay, that can be present in any voxel around the cells, acts as a barrier for the cells by creating a repulsion force. The oxygen freely diffuses into the microenvironment toward the center of the spheroid from the border of the simulation box, while TGFbeta is encapsulated into the ECM. In the model, oxygen is needed for both cellular respiration and as a trigger for the cells’ motility, and is uptaken by the cells.

It is only when the cells have acquired the appropriate phenotype (“ECM_degrad” and “Migration” active) that they can trigger invasion. Secondly, the ECM contains a certain amount of TGFbeta, which is released when the cell degrades the ECM above a threshold. We fitted this threshold to reproduce experimental data, although this parameter does not seem to have a strong impact the different modes of invasion. When ECM is degraded, TGFbeta becomes accessible to the neighboring cells that initiate its uptake. The presence of oxygen and TGFbeta in the microenvironment, if above a certain threshold, can trigger the activation of the corresponding input node (“TGFbeta,” “Oxy”) in the intracellular model. The ECM input node is triggered when a cell has more contact with the ECM than a user-defined threshold. This value is stored into the variable *ecm_contact* and is based on the amount of overlap between the cell and the voxel. The intracellular node “Neigh” is the readout for cell–cell contact. In order to quantify cellular contact, the distance between neighboring cells is stored into an environmental variable *cell_contact* and corresponds to the percentage of their overlapping cell radii. This value is compared to *cell_cell_contact_threshold* thresholds to set “Neigh”’s value. To simulate DNA damage, we consider the physical stress that the cells at the border of the tumor have when they are pushed against the ECM barrier, suffering high nuclear pressure. This pressure is represented as an overlap (distance) between the nucleus radius of the agents and the voxel containing the ECM. When this overlap reaches a given threshold (*DNA_damage_threshold *=0.8), it triggers the “DNA_damage” node.

Similarly, the outputs (or read-outs) of the intracellular model are connected to functions inside each agent. The “Apoptosis” node triggers the apoptotic death model of PhysiCell. “Migration” changes the direction of the motility of the cells, from a random walk to a chemotaxis movement toward the highest concentration of nutrients (represented by oxygen). As “Migration” is activated, the internal variable of the cell “pmotility” increases. This parameter modifies the motility speed (*motility_speed*), accelerating the cells from 0 until it reaches the maximum value set in the configuration file.

“EMT” is considered as a necessary early step for cell invasion that modifies some mechanical aspects of the agent: once activated, it impacts the *padhesion* variable that indicates the percentage of cell adhesion with neighboring cells. EMT value also determines whether a cell attaches to neighboring cells or not, a phenomenon that is characterized by a spring-like adhesion.

The formed attachment is disbanded if: (i) *padhesion* is below a certain threshold, simulating the lack of cell junctions by the E-cadherins, or (ii) an external mechanical force causes the separation of the attachment. The node “ECM_adh” that accounts for the junction between the cell and the ECM activates a function that increases the amount of integrins in the agent (corresponding to the parameter *integrins* in the model). The node “ECM_degrad” is monitored by the MMPs, enzymes that allow the cells to degrade the ECM and hence start invasion. In the model “ECM_degrad” triggers the uptake rate of ECM substrates based on the amount of integrins displayed by a cell, reducing the density of the ECM value in the target voxel and facilitating the cell’s movement through it.

### 2.5 Role of key model parameters

**Figure 2.**
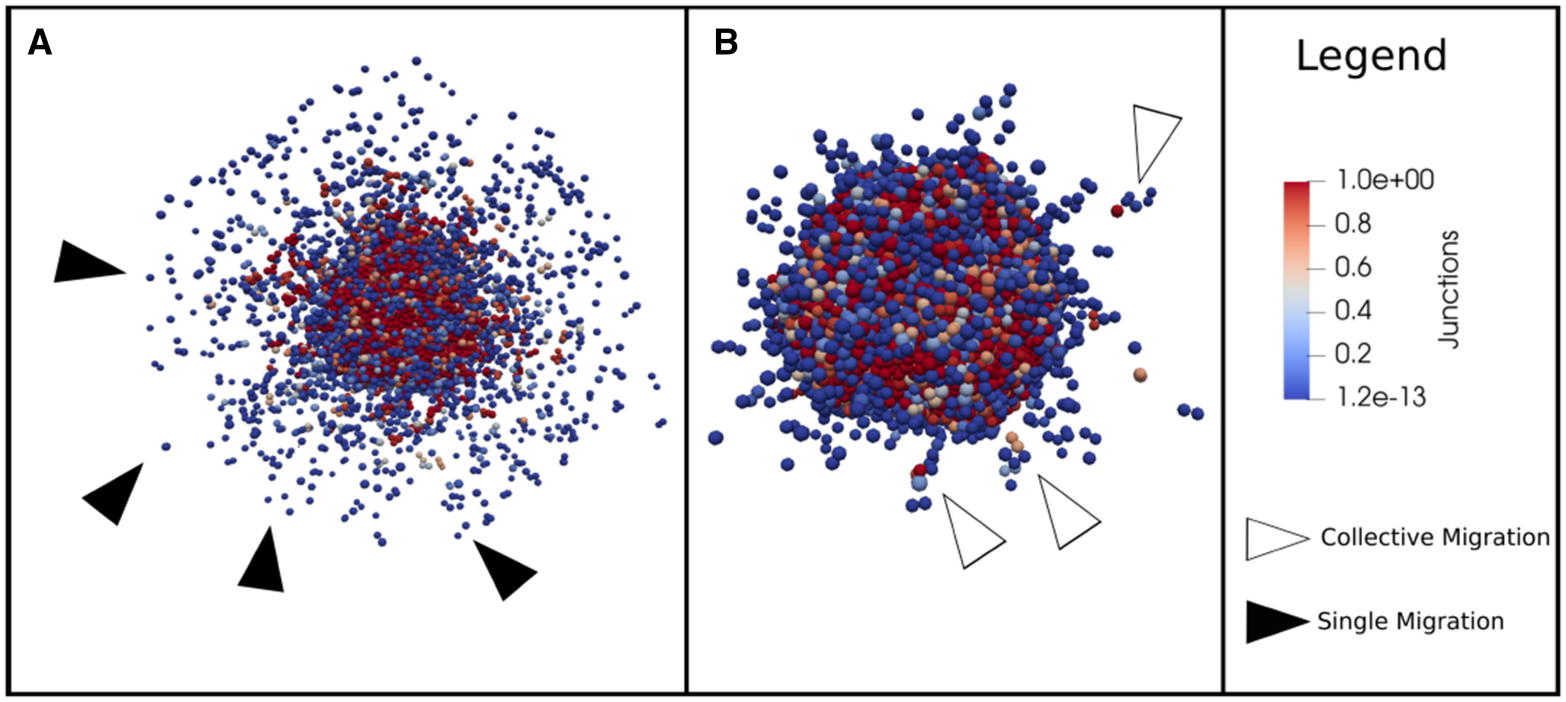
The 3D representation of the simulation. For (A) and (B), different values of the parameters have been used to reproduce single (black arrows) and collective cell migration (white arrows). More details about the quantification of single and collective migration are given in Supplementary Section S13. The color bar represents the amount of cell junction used to establish cellular adhesion. (A) cell_ecm_repulsion =10, epith_cell_junction_attach =0.5, mes_cell_junction_detach =0.03, migration_bias =0.9, migration_speed =0.8. (B) cell_ecm_repulsion =15, cell_junction_attach =0.005, cell_junction_detach =0.001, migration_bias =0.8, migration_speed =0.7.

Some model parameters correspond to biophysical mechanisms that are difficult to infer from experiments. A parameter set is proposed in order to simulate the different invasion modes, qualitatively reproducing the experimental images in Section 3. A subset of seven parameters were chosen to perform a sensitivity analysis (listed in Table 1) that cause the simulation to switch from one mode of invasion to another (Fig. 2).

**Table 1.** List of the seven parameters studied in the sensitivity analysis.^a^

Parameters	Description	Range
*cell_ecm_repulsion*	Regulates the amount of repulsion between cell and ECM	0 < **15 **<** **75
*epith_cell_attach_threshold*	Changes the activation threshold needed to attach cells in cluster with cell junction	0.0 < **0.05 **<** **1
*mes_cell_detach_threshold*	Changes the activation threshold needed to detach cells in cluster with cell junction	0.0 < **0.03 **<** **1
*cell_cell_contact_threshold*	Changes the activation threshold of the value cell_contact needed to trigger the Neigh node	0.0 < **0.3 **<** **3.5
*cell_ecm_contact_threshold*	Changes the activation threshold of the value ecm_contact needed to trigger ECM node	0.0 < **0.05 **<** **1
*migration_bias*	Changes the value of migration bias for cells with Migration node active	0.0 < **0.8 **<** **1
*migration_speed*	Changes the value of migration speed for cells with Migration node active	0.0 < **0.7 **<** **1

a For each parameter, we focused on a range of values, picked several values in it, ran 50 simulations for each parameter set and selected the number in bold to reproduce the published experiments presented here.

The *cell_ecm_repulsion* regulates the force of repulsion between a cell and the ECM. The parameter *epith_cell_attach_threshold* controls the activation threshold that allows cells in the epithelial state to form tight junctions with the cells around them, and *mes_cell_detach_threshold* describes the force needed to break this bond in mesenchymal cells. The *cell_cell_contact_threshold* changes the required distance between a cell and its neighbors to activate the “Neigh” node and *cell_ecm_contact_threshold* the distance between a cell and the ECM to activate the ECM node. The parameter *migration_bias* governs the stochasticity of the movement of mesenchymal cells. The higher its value, the less stochastic the movement is. Finally, the parameter *migration_speed* describes the velocity of a mesenchymal cell.

For each parameter, a range of values is selected (Table 1) and run 50 replicates for each. This number of replicates has been selected so as to minimize variations of the results for a reasonable computation time. By varying their value, we are able to modulate the presence of single cells and cells migrating in clusters (see Supplementary Section S15).

To quantify the number of cells in clusters or without attachment, we classify each agent based on the proximity of other cells and the links that exist between them. The information is interpreted as a network where nodes are agents and edges represent the link between two cells. With tools, such as Cytoscape (Shannon *et al.* 2003), we can visualize the network and count the number of single cells, number of clusters, and the number of cells in a cluster. This quantification is useful when performing parameter sensitivity analyses. For more details, see Supplementary Section S13.

## 3 Results

In order to validate the model, we successfully reproduced experimental observations reported in two studies: the first one focuses on the secretion of MMPs through the modification of p63 (Lodillinsky *et al.* 2021) and the second one on the local activation of SRC, which leads to collective migration (Moitrier *et al.* 2019). These applications show how interventions to the intracellular or extracellular mechanisms of the cell can affect the global behavior of the tumor growth and invasion. The model can then suggest means to interfere with these behaviors, either by modifying the parameters of the agent-based model, or by simulating perturbations in the signaling pathway by modifying the intracellular model. In practice this would be translated experimentally by modifying, among many things, the density of the matrigel, the adhesive affinities between cell types or performing gene knockouts to simulate potential drug targets. An additional example is provided in Supplementary Section S12 and focuses on the role of the ECM in the different modes of invasion (Ilina *et al.* 2020).

### 3.1 The role of p63/MT1-MMP in tumor invasion

**Figure 3.**
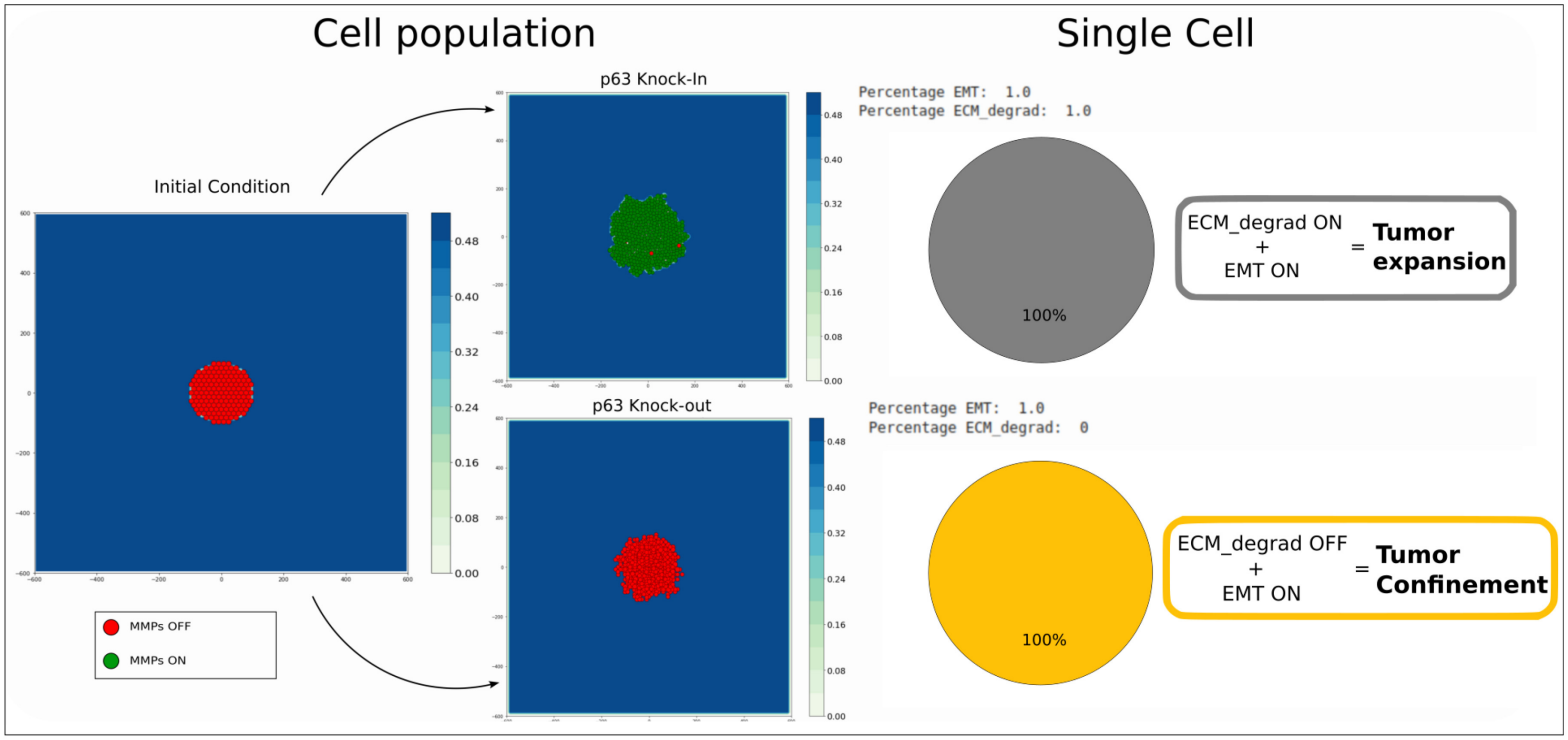
Reproduction of the p63 knockin/knockout experiment from Lodillinsky *et al.* (2021). The color of the ECM (blue gradient) shows its local density. The initial conditions are the same for both simulations (left panel): Oxygen =1, Growth_factor =1, Neighbor =1, ECM =1, TGFbeta =1, and DNAdamage =0. In p63 overexpression condition (upper middle panel), the ECM can be degraded with the MMPs ON and shows tumor expansion at the cell population level. The intracellular model (right upper panel) shows the two phenotypes: ECM_degrad and EMT ON. In p63 knockout condition (lower middle panel), the MMPs cannot be released leading to the inhibition of the ECM degradation phenotype. The intracellular model (right lower panel) shows that the cell has undergone EMT but is unable to degrade the ECM, which is interpreted at the population level as tumor confinement. Cell density analysis confirms that in a condition of p63 overexpression, the area occupied by the tumor increases, decreasing the cell density. On the other hand, inhibition of p63 decreases the area occupied by the tumor, increasing cell density (Supplementary Section S9 and Supplementary Fig. S7).

Lodillinsky and colleagues have reported that, in basal-like breast cancer, the secretion of MT1-MMP and subsequent cell migration, was strictly linked to the up-regulation of p63 (Lodillinsky *et al.* 2021). It was shown in this study that the contact of cells with the ECM increased the level of an isoform of p63, Δp63, and as a consequence of MT1-MMP. Through the inhibition of p63, they noticed a strong decrease in the MT1-MMP level, thereby decreasing the process of invasion. The overexpression of MT1-MMP instead reestablished the invasive capacity of p63 depleted cells (Fig. 3, top middle panel). In this example, we modified the intracellular activity of p63 and observed the consequence at the population level. The node “MMPs” of the model accounts for a set of MMPs consisting of MT1-MMP (MMP14), MMP13, and MMP2 (Ferrari *et al.* 2019) and its activity is regulated by Notch, SMAD, RAC1, p73, and p63 (Supplementary Section S2). We first simulated the full inhibition of “p63” (setting the node to 0) in conditions that would activate the migration process (Oxygen =1, Growth_factor =1, Neighbor =1, ECM =1, TGFbeta =1, DNAdamage = 0). In this simulation, MMPs could not be released, blocking the invasive capacity of the cells and confining the tumor (Fig. 3, lower panel). When p63 is over-expressed, and as a consequence MMPs are over-activated, the cells are able to degrade the ECM, allowing the tumor to expand and grow (Fig. 3) as observed in the published experiment. The model was able to mimic experimental observations. We notice that the inhibition of p63 does not block the EMT activation. Here, the cells are still able to shift to a mesenchymal state, to grow and to divide. On the other hand, overexpression of p63 in the simulation promotes ECM degradation but partially blocks EMT transition and completely blocks Migration, allowing tumor expansion but not single or collective migration as reported in Lodillinsky *et al.* (2021).

To further explore this scenario, we searched for an additional mutation that could stop invasion in the p63/MMPs overexpression condition. We initially performed a sensitivity analysis of the intracellular model, which consisted in automatically deleting and overexpressing all the nodes of the network, and selected ERK knockout, which affects the expression of p21 and the phenotype “Cell_growth,” “Apoptosis,” and “Migration”. We simulated the p63/MMPs conditions first, without the mutation, and we then introduced the knockout halfway of the simulation. ERK knockout caused the triggering of apoptosis in almost all the cells completely stopping the invasion process. Interestingly, the knockout of ERK without p63 overexpression only caused a small percentage of cells to go into apoptosis without stopping the invasion process (Supplementary Section S9). In practice, targeting ERK might have too many collateral effects, but this type of simulations highlights how the model can be used to better understand the processes linked to invasion and search for means to slow it down or suppress it.

### 3.2 Simulation of local light activation of the SRC oncoprotein in an epithelial monolayer

Moitrier *et al.* used a synthetic light-sensitive version of SRC to trigger its activity upon light activation (Moitrier *et al.* 2019). They subjected a monolayer of cells to intermittent blue light to induce a spatially constrained activation of the pro-invasive SRC protein and correlated this with the formation of extruded cells that remain cohesive.

**Figure 4.**
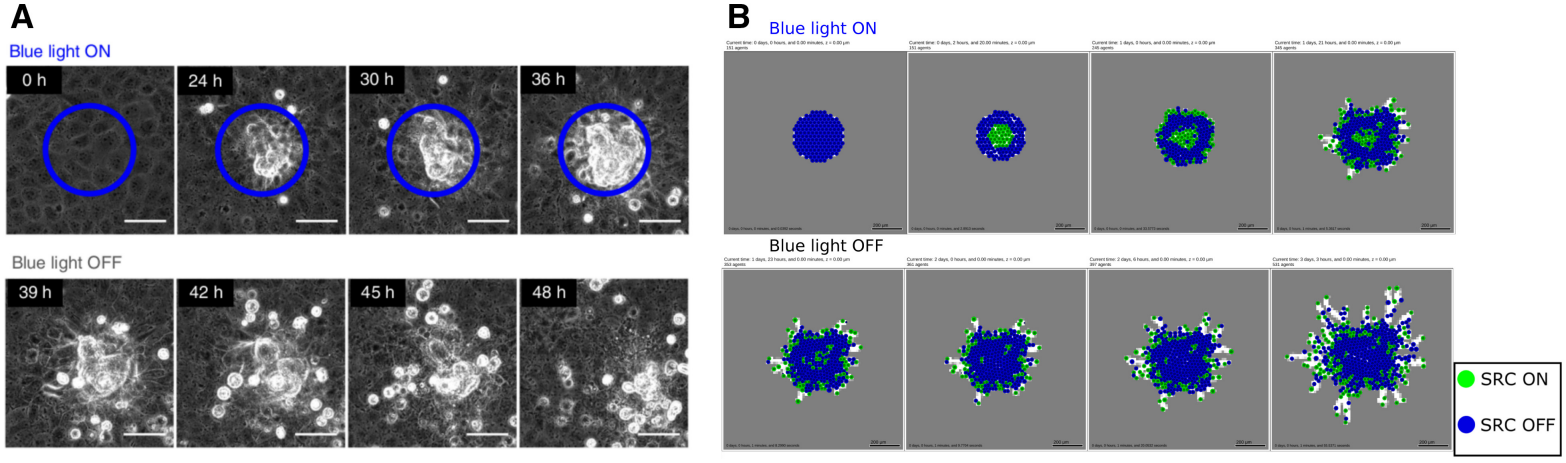
Reproduction of the SRC experiments. (A) Experimental results of SRC activation at different time points from Moitrier *et al.* (2019). The blue circle indicates the area of the blue light activation. After 33 h, the light source is removed, reversing the collective extrusion phenotype. (B) Model simulation in 2D of the SRC experiments. We introduced a substrate that simulates the light in the middle of the epithelial monolayer (blue cells) trapped in the ECM (grey voxels). The white voxels correspond to space where the ECM has been degraded. The substrate virtually interacts with cells with a SRC activating mutation. The cells undergo EMT and become mesenchymal (green cells), trying to migrate and forming aggregates. When the substrate is removed, the mesenchymal cells return epithelials. SRC is found active at the borders of the monolayer in contact with ECM.

In the intracellular model, SRC regulates the activity of CTNNB1 and CDH2 (Qi *et al.* 2006), CDH1 (Selvaggio *et al.* 2020) and the production of vimentin (Yang *et al.* 2019a). SRC is activated by FAK. We used two different setups in 2D and 3D to simulate this experimental setting. In the 2D scenario (Fig. 4), upon activation of the blue light, the affected cells start to develop a migratory phenotype similar to that of cells in contact with the ECM. SRC activation triggers the disgregation of cell junctions and EMT, pushing the cells to migrate toward the source of oxygen. The layer of epithelial cells surrounding the cells that have acquired the EMT phenotype confines them and favors the formation of aggregates that try to free themselves by pushing these EMT-like cells outwards, leading to collective migration. Removing the light source from monolayer reverses the cell’s phenotype. The cells that went through EMT return in the epithelial state, blocking the migration process. We then replicate the experiment in 3D (Supplementary Fig. S11). For this case, the experiment setup was modified: the ECM was removed to avoid the activation of the mesenchymal phenotype at the border of the monolayer and the cells were placed at the bottom of the domain. With this setup, the cells no longer push against the layer of epithelial cells, but rather migrate vertically forming an extrusion on top of the monolayer as observed in the original experiment. With the deactivation of the light source, the cells undergo a SRC inhibition, reversing the EMT phenotype and freezing. The epithelial cells in the simulation keep proliferating, filling the gap left by the mesenchymal cells.

We extended the study by testing the effect of SRC overexpression on the whole tumor. As expected, it caused a burst of invasion, speeding up the formation of both clusters and migrating single cells.

We also searched for a way to limit or even suppress the metastatic formation in these conditions. We first tried to stop the activation of the “ECM_degradation” phenotype through a knockout of “p63,” which was insufficient to inhibit MMPs activation. After a parameter sensitivity analysis, we targeted the ERK gene. As in the previous example, we introduced an inhibitory mutation of ERK halfway through the simulation, which stopped the tumor progression not by promoting apoptosis, but rather by inhibiting the cell movement and cell cycle.

### 3.3 Simulation of drug candidates that can block migration

As already mentioned, in addition to reproducing published studies, the model can also be used as a tool to explore means to revert invasive phenotypes or prioritize possible points of intervention and drug treatments with *in silico* experiments. We tested possible drug candidates that could block migration in conditions where cells have already undergone EMT and have started to invade the surrounding ECM. As a result of a systematic search for single and combined drug treatments, we found that overexpressing the activity of “CTNNB1” (forcing its value to 1) would not prevent tumor growth but would greatly limit the invasive capacity (Supplementary Fig. S10). We tested this hypothesis by introducing an overexpression mutation both at the beginning and halfway through the simulation. In both cases, the treatment blocked the invasive capacities of the cells, preventing the activation of the node MMPs and, as a result, blocking the ECM degradation process. Identifying drugs that target CTNNB1 or any of its downstream actuators could have the potential of limiting invasion. In fact, there have been proposals for targeting CTNNB1 as the main player in the WNT canonical pathway in breast cancer as with 3,6-dihydroxyflavone in MDA-MB-231 cell line (Anastas and Moon 2013), even though many unknowns remain (van Schie and van Amerongen 2020). A more extensive list of candidates can be found in the Jupyter notebook “intercellular_model_analysis” and in Supplementary Section S10, including miR34, which shows complete blockade of EMT.

### 3.4 Sensitivity analysis of the model parameters

The choice for the model parameters linking the environment and the tumor cells is always a difficult task as it cannot be fit to known values because most of these parameters are not easily measurable. We performed a sensitivity analysis on a subset of seven parameters, as shown in Table 1, that are crucial in determining the modes of invasion, to investigate their impact on the results. More details about the sensitivity analysis, including choice of the values, parameters ranges, and number of replicates, can be found in Supplementary Section S15. The parameter *cell_ecm_repulsion* controls the repulsion force that the ECM applies to the cells. For *cell_ecm_repulsion *=0, the ECM loses its confinement property, allowing the tumor to expand evenly and minimizing invasive behavior. For values higher than 5, the ECM starts repelling the cells, allowing the triggering of the invasive properties of the cells, but for higher values, it also increases its confinement capacity, minimizing invasion, and blocking tumor expansion (Supplementary Fig. S22). This is because, for low repulsion values, the cells can reach the ECM voxel more easily and, upon contact, are able to change to a mesenchymal phenotype before developing cell–cell adhesion and junction.

*Epith_cell_attach_threshold* and *mes_cell_detach_threshold* monitor the process of junction formation between cells. We varied these two parameters independently. Increasing *epith_cell_attach_threshold* from its minimum value of 0 results in an increase in the number of single cells, the total number of cells in the clusters, and a slight increase in the number of clusters. The latter reaches a plateau around a value of 0.2, while the number of single cells and cells in clusters continues to increase. For high values of the parameter, the number of cells in clusters tends to exceed the number of single cells (Supplementary Fig. S23).

The analysis of *mes_cell_detach_threshold*, instead, shows a robust behavior for values higher than 0.05 and no change in the rate between single cells and cells in the clusters (Supplementary Fig. S24).

The range over which to vary the threshold value for *cell_ecm_contact_threshold* and *cell_cell_contact_threshold* was calculated based on the maximum compression reached between cell/cell and cell/ECM. The analysis of *cell_ecm_contact_threshold*, which represents the ability of the cell to detect ECM, shows a slight decrease in the number of single cells and cells in clusters, while the total number of clusters remains almost unchanged. For values >0.7, the number of single cells and cells in clusters decreases significantly, almost reaching zero for values >0.85 (Supplementary Fig. S26). For *cell_cell_contact_threshold*, the model proves to be very robust, with little impact on the separation of clusters and single cells (Supplementary Fig. S25).

*Migration_bias* controls the tendency of a cell to migrate toward its chemoattractant source, varying from 0 (pure random walk) to 1 (pure deterministic movement). Our analysis reveals that this parameter heavily influences the number of single cells. For values below 0.45, the number of cells in clusters increases similarly to the number of single cells. For higher values, the number of single cells grows faster, exceeding the number of cells in clusters and reaching a plateau around a value of 0.55 (Supplementary Fig. S27).

The analysis of *migration_speed* reveals that this parameter has minimal impact on the segregation of clusters and single cells. When values exceed 0.1, the number of cells in clusters reaches a plateau. However, the number of single cells continues to increase until a value of 0.2, after which it stabilizes around 30. When *migration_speed *=0, cells do not move due to oxygen attraction but rather due to cellular replication-induced pushing, resulting in homogeneous tumor growth (Supplementary Fig. S28). In biological terms, we can conclude that the more motile a cell is, the less likely it will form a cluster.

## 4 Discussion

In this study, we present a multiscale model that combines spatial cell representation and intracellular signaling to reproduce the different modes of cell invasion using PhysiBoSS. The model proved to be efficient in reproducing *in vitro* experiments and simulating different experimental scenarios. We managed to reproduce two invasion modes: single and collective, thanks to a combination of phenotypes and mechanical interactions with the ECM. This study confirms that tumor invasion is a complex process that benefits from considering spatial information, interaction with the microenvironment and intracellular representation.

One application of this model could be to suggest and anticipate the potential risk of metastasis for patients that have a combination of mutations. Currently, the model includes a reasonable number of genes to allow for a fast simulation and is able to capture the biological differences between the different modes of invasion. However, to be more precise in the model predictions, more genes and pathways could be included in the future based on new experiments. The choice of these pathways could be suggested by identifying differentially expressed genes or molecular signatures for patients with high metastatic potential (Montagud *et al.* 2022), exploring public patient datasets, such as The Cancer Genome Atlas, data for which clinical data about tumor invasiveness are provided. In future releases, we also plan to include more cell types, such as cancer-associated fibroblasts and T-cells, to represent the role of the immune response. In this setting, each cell type will have its own intracellular model and would interact with other cell types according to biologically relevant rules.

The current model has demonstrated its predictive potential, but it has some limitations. One limitation is that the intracellular model can reproduce the EMT transition, but the commitment to EMT is final and non-reversible spontaneously. The reverse process of MET occurs just as a consequence of mutations or DNA damage, and not as an active process initiated by the cell itself. The second limitation concerns the difficulty of choosing a proper set of parameters that can reproduce experimental data. While performing the sensitivity analysis, we mainly ran into two problems: (i) the number of parameters to analyze for this model is large and requires many long computations, and (ii) we noted a high presence of noise in the results, even for a large number of simulations per parameter (50 runs for each tested value, see Supplementary Section S15). This adds up to other computational costs of running PhysiBoSS simulations on a regular laptop. The 2D simulations can take a couple of minutes, but 3D simulations require at least 1 h. One way to tackle this issue that is currently being investigated is to parallelize further this simulation by using GPUs (Stack *et al.* 2021) or multiple MPI nodes (Saxena *et al.* 2021).

Another way to address this parameter exploration issue is to rely on simpler, approximate simulations. For that, we plan to explore surrogate models and use them to learn subsets of parameters (Preen *et al.* 2019). More specifically, we want to train a machine learning algorithm connecting the inputs (parameter values) and the outputs of PhysiBoSS model, which will act as a surrogate for further parameter optimization. We will compare the output of the surrogate model with *in vitro*/*in vivo* data to find the best combination of parameters, which will be tested in a relatively small number of simulations. With sufficiently many new simulations, the surrogate model will be synchronized with the original model by re-training to maintain the correspondence between the two models.

Finally, the multiscale model can be used as an exploratory and predictive tool to test hypotheses before performing wet lab experiments. The model is accompanied by the Jupyter Notebook and the Nanohub Tool to facilitate the reproducibility of the model results but also to allow users (biologists and/or modelers) to test additional experiments.

## Supplementary Material

btad374_Supplementary_Data
