# Supplementary material for: Multiscale model of the different modes of cancer cell invasion
Source: Bioinformatics. 2023 Jun 8;39(6):btad374. doi: 10.1093/bioinformatics/btad374 (PMC10293590; doi:10.1093/bioinformatics/btad374)
Supplement: btad374_Supplementary_Data [file btad374_supplementary_data.zip › Supplementary File 2.pdf]

Multiscale model of the different modes of invasion

This notebook is a companion for the article on a model describing the different modes of invasion using the tool PhysiBoSS.

PhysiBoSS is a modeling tool combining an agent-based approach for intercellular interactions and a Boolean model for the intracellular description.

We focus here on the Boolean model describing the signaling pathways inside each cell

TABLE OF CONTENT

1) Libraries for simulations

2) Loading of the model

3) Network visualization

4) Stable state analysis

5) Definitions of the outputs of the model

6) Wild type simulation

7) Combinations of inputs: ECM/TGFbeta

8) Combinations of inputs: Oxy/GF

9) Combinations of inputs: DNAdamage

10) Mutants' simulations

11) CTNNB1 mutant

12) TP63 mutants

13) TP63 mutant with ECM/TGFbeta initial condition

14) SRC mutant

15) SRC mutant without ECM/TGFbeta initial condition

16) Model testing

17) Automatic (single/double) mutations_mutations)

Libraries for simulations

The libraries related to the tools needed for this analysis were developed in the context of CoLoMoTo, an initiative of the logical community to make all tools, methodologies and models in standard format to facilitate compatibility, interoperability and reproducibilty.

In [45]:
```
import maboss
import colomoto_jupyter
import maboss_test
import ginsim
import biolqm
import boolean
import matplotlib.pyplot as plt
plt.style.use('seaborn-colorblind')
```

In [2]:
```
import numpy as np
import seaborn as sns
```

The Boolean model is simulated using MaBoSS, which is based on a stochastic simulation applied to the Boolean network. The MaBoSS model is composed of two files, one for the model description (.bnd) and the a second one for the definition of both the model and simulation parameters (.cfg).

In this notebook, we aim to show the behavior of the individual cell by fixing the values of the inputs that usually vary in the agent-based model. The purpose here is to explore the possible fates of the cell depending on the state of these inputs.

The analysis will first be done on the wild type case (with no alterations) in different initial conditions, which corresponds to the different conditions in which the cells may be found during the simulation. Next, we explore the cell dynamics in diseased conditions by simulating different gene mutations.

Loading of the model

Here we load the .bnd and .cfg file necessary for the intracellular analysis.

The .bnd file contains the nodes and the logical formulas that regulates the interactions between genes. The .cfg file contains instead the parameters of the simulation for MaBoSS as well as the initial condition defined for the model.

We have defined a "standard initial condition", which represents a set of initial conditions for the model, defined in the MaBoSS configuration file (.cfg provided in the supplementary materials).

The initial conditions are set as follows: ECM=0, TGFbeta=0, Neighbor=0, Oxygen=1, Growth_factor=1, DNAdamage=0 (where 0 means inactive, 1 means active). Those conditions represent a favorable proliferating condition, where all the cells have access to oxygen and nutrients, are confined but not subjected to DNA damage or crowding.

Note that the simulation is set to represent the cells in an optimal condition to grow, and hence growth factors are assumed to be in a sufficient amount to constantly activate growth and maintain basal metabolism.

In [3]:
```
intracellular_model = maboss.load("./boolean_network/intracellular_model.bnd", "./boolean_network/intracellular_mod

on = [0, 1]
off = [1, 0]
```

Network visualization

The network can be visualized using GINsim representation and exported into the standard format SBML-Qual.

In [4]:
```
def convert_and_show(model):
    biolqm_model = maboss.to_biolqm(model)
    lrg = biolqm.to_ginsim(biolqm_model)
    return ginsim.show(lrg)
def convert_and_export(model):
    biolqm_model = maboss.to_biolqm(model)
    biolqm.save(biolqm_model, "mymodel.bnet", "bnet")
    return
```

In [5]:
```
convert_and_export(intracellular_model)
convert_and_show(intracellular_model)
```

Out[5]:

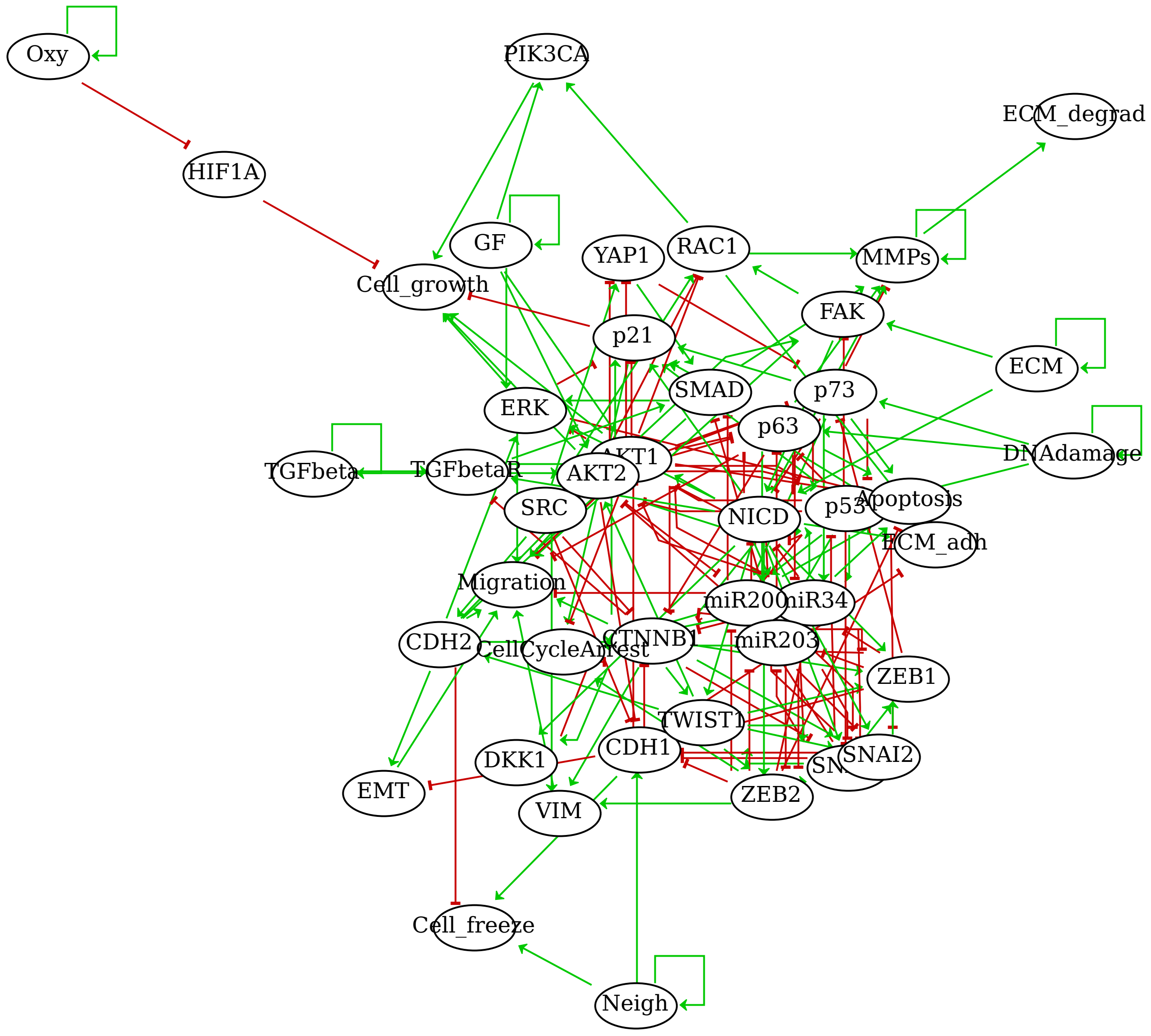

Stable state analysis

The model has 192 fixed points

In [6]:
```
biolqm_model = maboss.to_biolqm(intracellular_model)
fps = biolqm.fixpoints(biolqm_model)
print(len(fps), "fixpoints") # shows the number of fixpoints
```

```
192 fixpoints
```

In [7]:
```
tps=biolqm.trapspaces(biolqm_model)
print(len(tps), "trap spaces")
```

```
192 trap spaces
```

They can be visualized on a PCA plot with 3 outputs to show how they distribute among the phenotypes of interest

In [8]:
```
# Getting the list of nodes
nodes = list(fps[0].keys())

# Building a 2D numpy array with the nodes order defined above, with each fixpoint value
data = np.array([[fp[node] for node in nodes] for fp in fps])

# The array is XX fixpoints * XX nodes
data.shape

from sklearn import decomposition
from sklearn.decomposition import PCA
#pca = PCA(n_components=3)
#pca_out = pca.fit(data)

from pandas import DataFrame

# training the PCA
pca = decomposition.PCA()
pca.fit(data)

# getting the weights for original parameters
for i in range(0,3):
    featureWeights = list(pca.components_[i])
    weights = DataFrame(data = featureWeights)
    filename = 'principal_component_' + str(i) + '.txt'
#    weights.to_csv(filename, sep = '\t')
# Uncomment the last function if you wish to create the files of the weights

# Here the weights show that for PC1, RTK, p21, pRB, p27, MCL1, BCL2, BCLXL, IAPs contribute to 20% each
# For PC2, TRAIL and DR4_5 contribute negatively (-70%)
# For PC3, GF and GF_High contribute to 70%

## Visualise the results in the form of a graph
# Getting the new coordinates
X_pca = pca.transform(data)

# Plotting the steady states
plt.figure(figsize=(3, 4), dpi=200)
plt.plot(X_pca[:, 0], X_pca[:, 1], ".")

# Printing arrows, but there is too much. We need to select some nodes
#some_nodes = ["EGFR", "TP53","CDK1"]
some_nodes = ["Cell_growth", "Apoptosis","EMT"]
for some_node in some_nodes:

    v = pca.components_[0:2, nodes.index(some_node)].T
    plt.arrow(0, 0, v[0], v[1], color='red', width=0.01)
    plt.text(v[0], v[1], some_node, color='black', ha='center', va='center', fontsize=5)


plt.xlabel("PC1: %.0f%% variance explained" % (pca.explained_variance_ratio_[0]*100))
plt.ylabel("PC2: %.0f%% variance explained" % (pca.explained_variance_ratio_[1]*100))
```

Out[8]:
```
Text(0, 0.5, 'PC2: 9% variance explained')
```

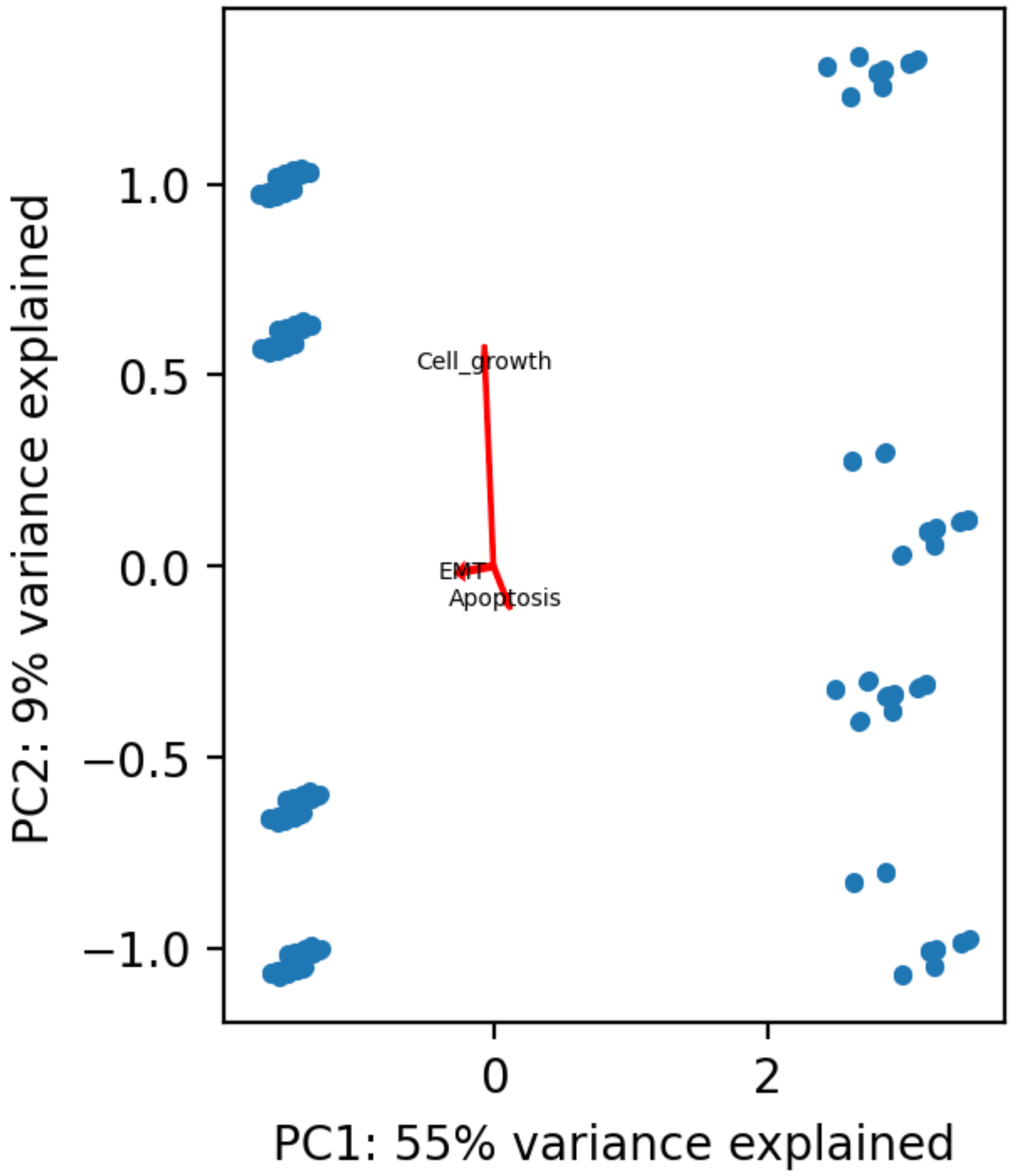

Definitions of the outputs of the model

The model outputs can be defined as a combination of some nodes that can be interpreted as read-outs of the model or phenotypes

In [5]:
```
set_output1 = ["Apoptosis", "Migration", "ECM_adh", "EMT", "Cell_growth", "ECM_degrad", "Cell_freeze"]
```

Wild type simulation

As a first exploratory analysis of the model, we simulate a wild type condition (no mutations) with random initial values as there is no information about the initial state of the model entities.

In [6]:
```
intracellular_model.param["thread_count"] = 10
intracellular_model.param["max_time"] = 100
maboss.set_nodes_istate(intracellular_model, intracellular_model.network.keys(), {0: 0.5, 1: 0.5})
maboss.set_output(intracellular_model, set_output1)
```

In [7]:
```
res1 = intracellular_model.run()
res1.plot_piechart()
```

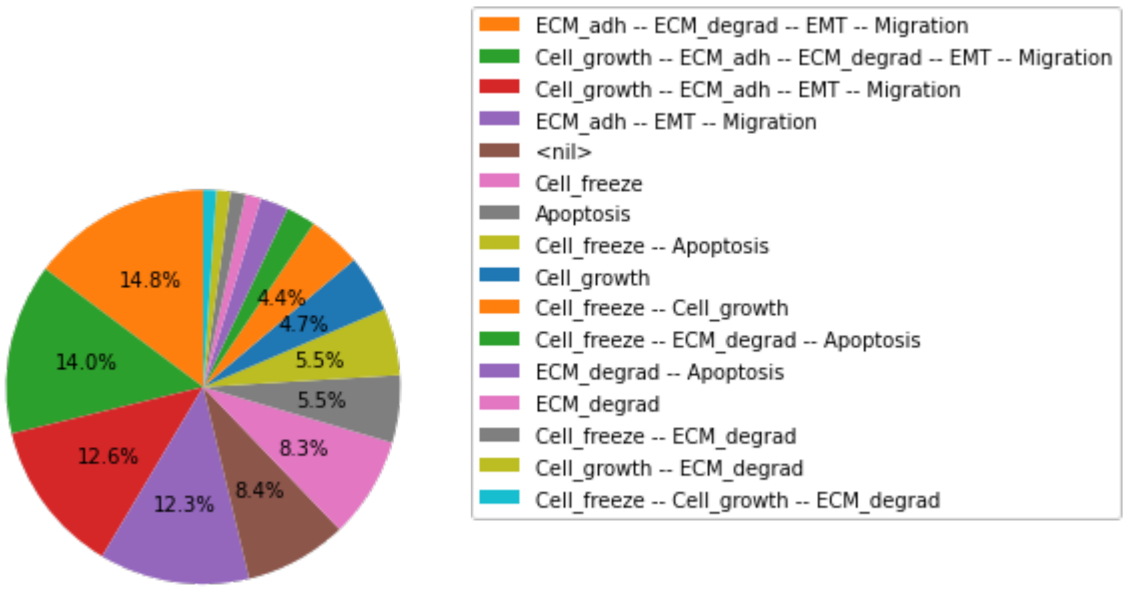

Note that every pie in the pie chart corresponds to the sum of the probabilities of all the stable states that have the corresponding output nodes equal to 1.

We can check the existence of limit cycles in the model by computing the entropy and the transition entropy which measures the internal chaos of the system. The obtained signature confirms the presence of a limit cycle but that cannot be determined easily

In [12]:
```
res1.plot_entropy_trajectory()
plt.title('WT entropy')
```

Out[12]:
```
Text(0.5, 1.0, 'WT entropy')
```

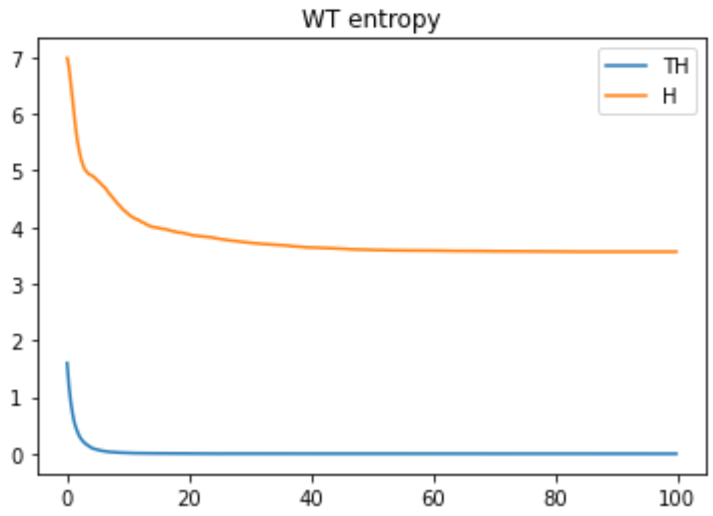

The simulation of MaBoSS can also show the probability for a node to be active in wild type and random initial conditions.

In [13]:
```
res1.plot_node_trajectory()
for phenotype in set_output1:
    try:
        print(phenotype, ": ", res1.get_last_nodes_probtraj()[phenotype].values[0])
    except:
        print(phenotype, ": ", 0)
```

```
Apoptosis :  0.1506
Migration :  0.546
ECM_adh :  0.546
EMT :  0.546
Cell_growth :  0.38589999999999997
ECM_degrad :  0.3923
Cell_freeze :  0.2288
```

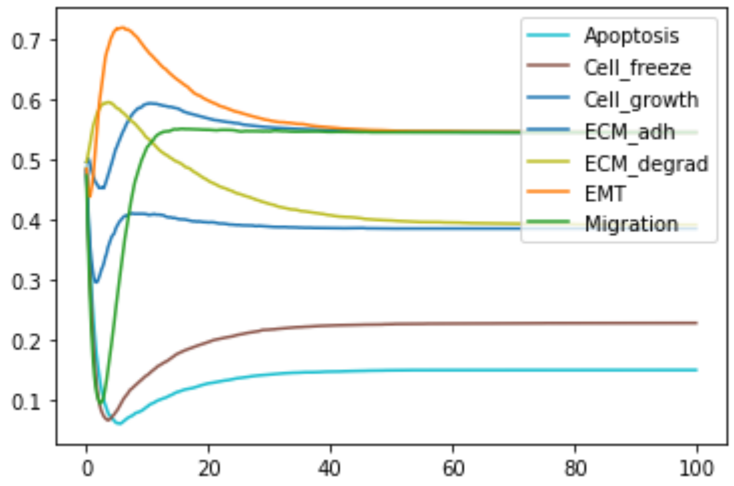

These conditions do not correspond to real physiological conditions. We can show that all phenotypes are reachable, though.

Next, we show which solutions are favored with a particular combination of inputs.

Combinations of inputs: ECM/TGFbeta

We explore here the possible states of the cells at the border of the spheroid, which get in **contact with the ECM and TGFbeta**.

In [17]:
```
i_condition = intracellular_model.copy()
i_condition.param["max_time"] = 100
maboss.set_nodes_istate(i_condition, i_condition.network.keys(), off)
i_condition.network.set_istate("Oxy",on)
i_condition.network.set_istate("GF",on)
i_condition.network.set_istate("Neigh",on)
i_condition.network.set_istate("ECM",on)
i_condition.network.set_istate("TGFbeta",on)
i_condition.network.set_istate("DNAdamage",off)
maboss.set_output(i_condition, set_output1)
```

In [18]:
```
res2 = i_condition.run()
res2.plot_piechart()
```

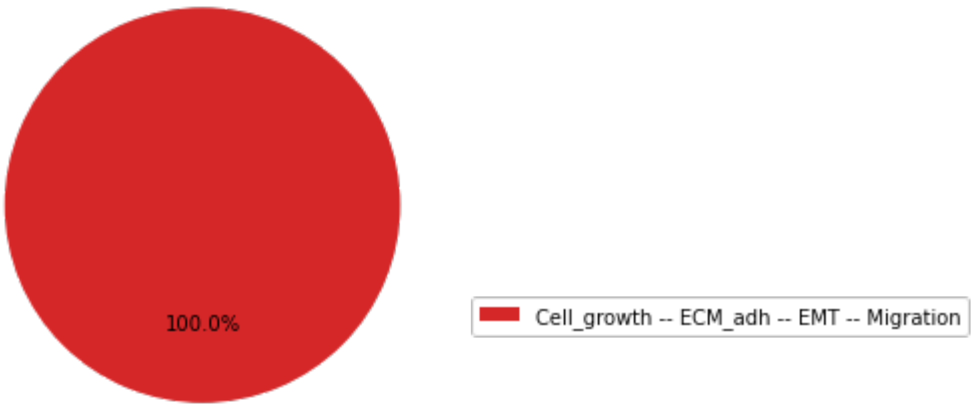

In [10]:
```python
res2.plot_node_trajectory()
for phenotype in set_output1:
    try:
        print(phenotype, ": ", res2.get_last_nodes_probtraj()[phenotype].values[0])
    except:
        print(phenotype, ": ", 0)
```

```
Apoptosis :  0
Migration :  1.0
ECM_adh :  1.0
EMT :  1.0
Cell_growth :  1.0
ECM_degrad :  0
Cell_freeze :  0
```

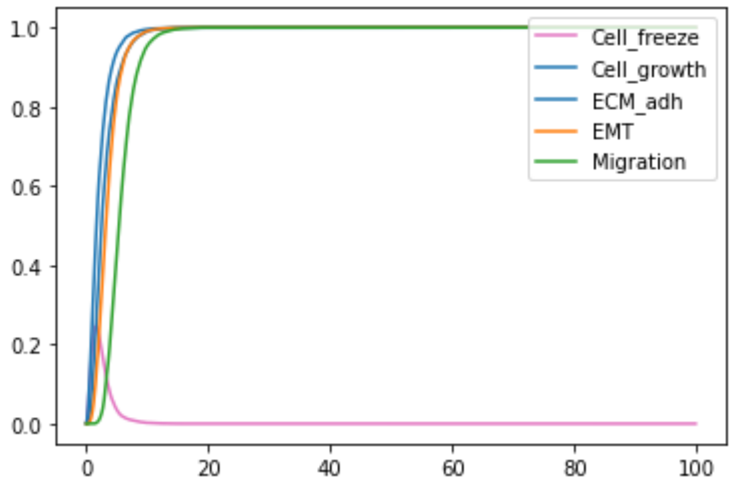

The simulation shows that the cell have the capacity to migrate and divide but not to degrade the ECM.

We explore now the phenotypes in the case of **contact with ECM** but in **absence of Growth Factor**

In [15]:
```python
i_condition1_1 = intracellular_model.copy()
i_condition1_1.param["max_time"] = 30
maboss.set_nodes_istate(i_condition1_1, i_condition1_1.network.keys(), off)
i_condition1_1.network.set_istate("Oxy",on)
i_condition1_1.network.set_istate("GF",off)
i_condition1_1.network.set_istate("Neigh",on)
i_condition1_1.network.set_istate("ECM",on)
i_condition1_1.network.set_istate("TGFbeta",on)
i_condition1_1.network.set_istate("DNAdamage",off)
maboss.set_output(i_condition1_1, set_output1)
```

In [16]:
```python
res2_1 = i_condition1_1.run()
res2_1.plot_piechart()
```

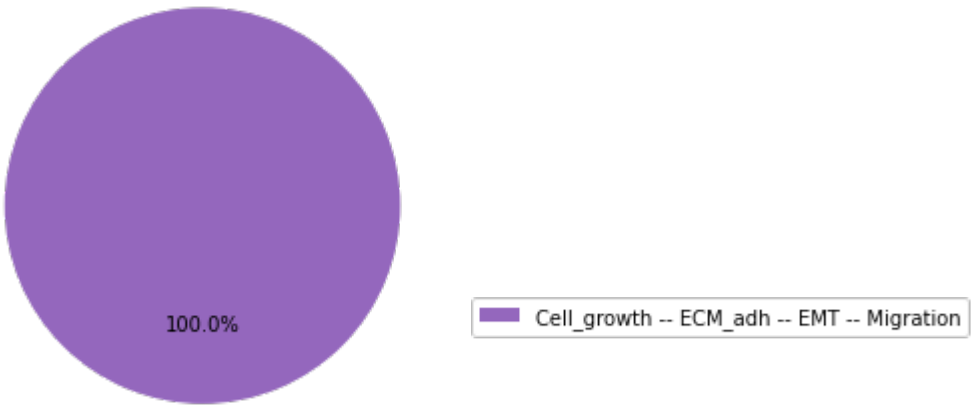

In [17]:
```python
res2_1.plot_node_trajectory()
for phenotype in set_output1:
    try:
        print(phenotype, ": ", res2_1.get_last_nodes_probtraj()[phenotype].values[0])
    except:
        print(phenotype, ": ", 0)
```

```
Apoptosis :  0
Migration :  1.0
ECM_adh :  1.0
EMT :  1.0
Cell_growth :  1.0
ECM_degrad :  0
Cell_freeze :  0
```

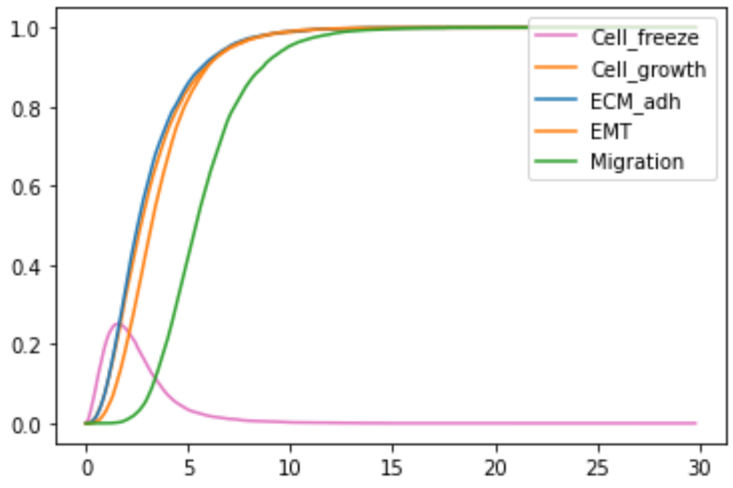

The presence or absence of growth factors does not seem to affect the fate of the cells

We explore now the phenotypes in the case of **contact with ECM** but in **absence of Growth Factor and with DNA damage** .

In [17]:
```python
i_condition1_2 = intracellular_model.copy()
i_condition1_2.param["max_time"] = 150
maboss.set_nodes_istate(i_condition1_2, i_condition1_2.network.keys(), off)
i_condition1_2.network.set_istate("Oxy",on)
i_condition1_2.network.set_istate("GF",off)
i_condition1_2.network.set_istate("Neigh",on)
i_condition1_2.network.set_istate("ECM",on)
i_condition1_2.network.set_istate("TGFbeta",on)
i_condition1_2.network.set_istate("DNAdamage",on)
maboss.set_output(i_condition1_2, set_output1)
```

In [18]:
```python
res2_2 = i_condition1_2.run()
res2_2.plot_piechart()
```

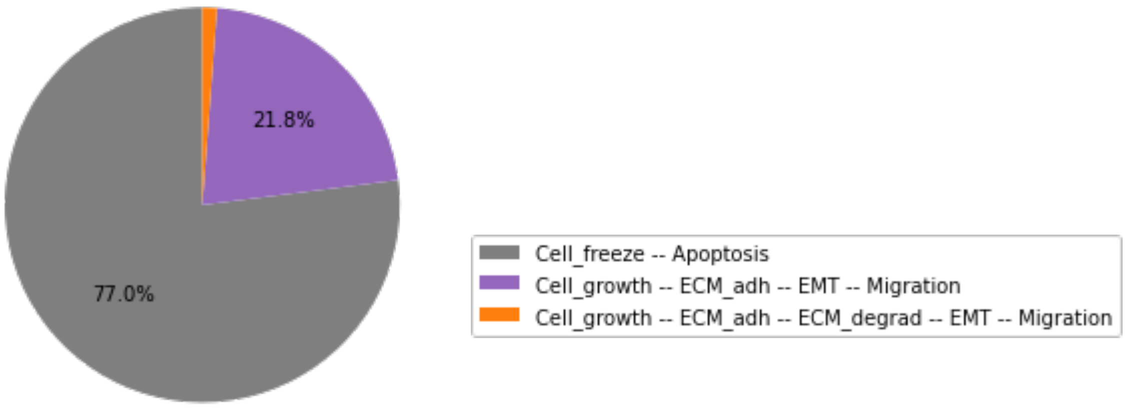

In [19]:
```python
res2_2.plot_node_trajectory()
for phenotype in set_output1:
    try:
        print(phenotype, ": ", res2_2.get_last_nodes_probtraj()[phenotype].values[0])
    except:
        print(phenotype, ": ", 0)
```

```
Apoptosis :  0.7701
Migration :  0.2299
ECM_adh :  0.2299
EMT :  0.2299
Cell_growth :  0.2299
ECM_degrad :  0.0118
Cell_freeze :  0.7701
```

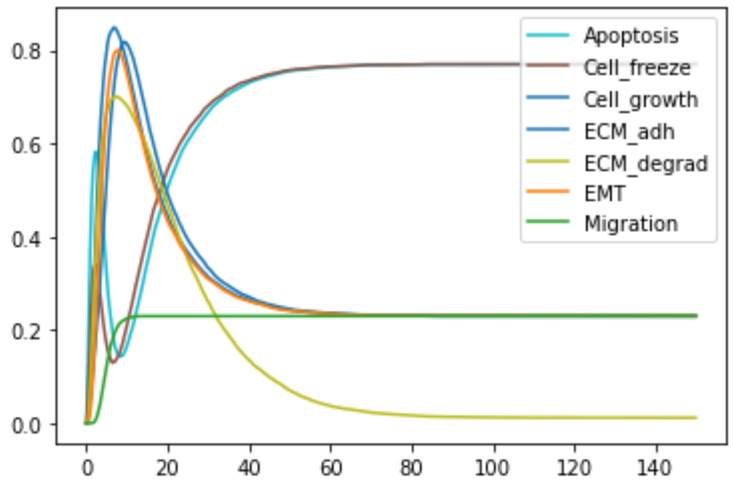

In the presence of DNA damage, a big majority of the cells will go into **apoptosis or stop growing**..

Combinations of inputs: Oxy/GF

We explore here the possible states of the cells inside the spheroid, **without contact with the ECM**, but in optimal conditions to grow (**Grow factor and Oxygen ON**)

In [21]:
```
i_condition2 = intracellular_model.copy()
i_condition2.param["max_time"] = 30
maboss.set_nodes_istate(i_condition2, i_condition2.network.keys(), off)
i_condition2.network.set_istate("Oxy",on)
i_condition2.network.set_istate("GF",on)
i_condition2.network.set_istate("Neigh",on)
i_condition2.network.set_istate("ECM",off)
i_condition2.network.set_istate("TGFbeta",off)
i_condition2.network.set_istate("DNAdamage",off)
maboss.set_output(i_condition2, set_output1)
```

In [22]:
```
res3 = i_condition2.run()
res3.plot_piechart()
```

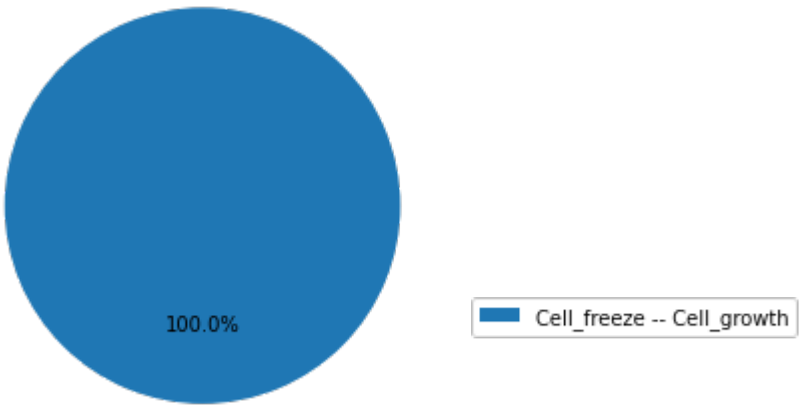

In [25]:
```
res3.plot_node_trajectory()
for phenotype in set_output1:
    try:
        print(phenotype, ": ", res3.get_last_nodes_probtraj()[phenotype].values[0])
    except:
        print(phenotype, ": ", 0)
```

```
Apoptosis :  0
Migration :  0
ECM_adh :  0
EMT :  0
Cell_growth :  1.0
ECM_degrad :  0
Cell_freeze :  1.0
```

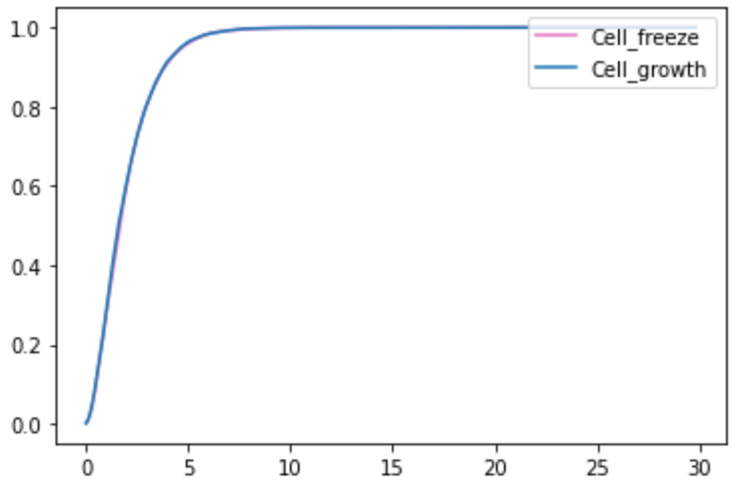

In the same condition but **without Growth Factor**, the cells will stop growing, rimaining in a freezing condition.

In [25]:
```
i_condition2_bis = intracellular_model.copy()
i_condition2_bis.param["max_time"] = 30
maboss.set_nodes_istate(i_condition2_bis, i_condition2_bis.network.keys(), off)
i_condition2_bis.network.set_istate("Oxy",on)
i_condition2_bis.network.set_istate("GF",off)
i_condition2_bis.network.set_istate("Neigh",on)
i_condition2_bis.network.set_istate("ECM",off)
i_condition2_bis.network.set_istate("TGFbeta",off)
i_condition2_bis.network.set_istate("DNAdamage",off)
maboss.set_output(i_condition2_bis, set_output1)
```

In [26]:
```
res3_bis = i_condition2_bis.run()
res3_bis.plot_piechart()
```

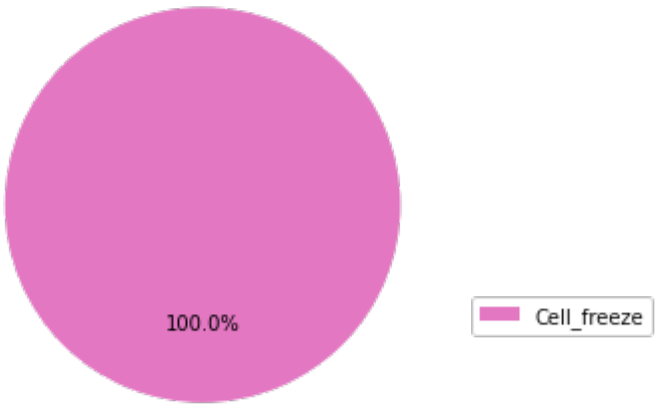

Combinations of inputs: DNAdamage

We explore here the possible states of the **cells at the border of the spheroid**, in high pressure conditions (**crowding**), which cause **nucleus deformation** and as consequence, the rupture of nuclear envelope. This is then interpretated as **DNA damage** in the model. This situation differs from the one illustrated from **i_condition1_2** for the **presence of Growth Factor**, which in i_condition1_2 was absent.

In [26]:
```
i_condition3 = intracellular_model.copy()
i_condition3.param["max_time"] = 150
maboss.set_nodes_istate(i_condition3, i_condition3.network.keys(), off)
i_condition3.network.set_istate("Oxy",on)
i_condition3.network.set_istate("GF",on)
i_condition3.network.set_istate("Neigh",on)
i_condition3.network.set_istate("ECM",on)
i_condition3.network.set_istate("TGFbeta",on)
i_condition3.network.set_istate("DNAdamage",on)
maboss.set_output(i_condition3, set_output1)
```

In [27]:
```
res4 = i_condition3.run()
res4.plot_piechart()
```

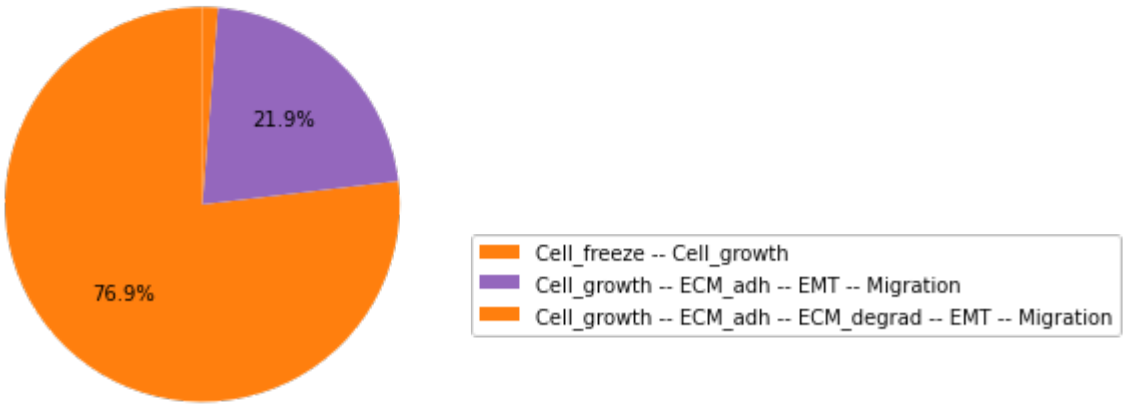

In [28]:
```
res4.plot_node_trajectory()
for phenotype in set_output1:
    try:
        print(phenotype, ": ", res4.get_last_nodes_probtraj()[phenotype].values[0])
    except:
        print(phenotype, ": ", 0)
```

```
Apoptosis :  0
Migration :  0.2311
ECM_adh :  0.2311
EMT :  0.2311
Cell_growth :  1.0
ECM_degrad :  0.0126
Cell_freeze :  0.7689
```

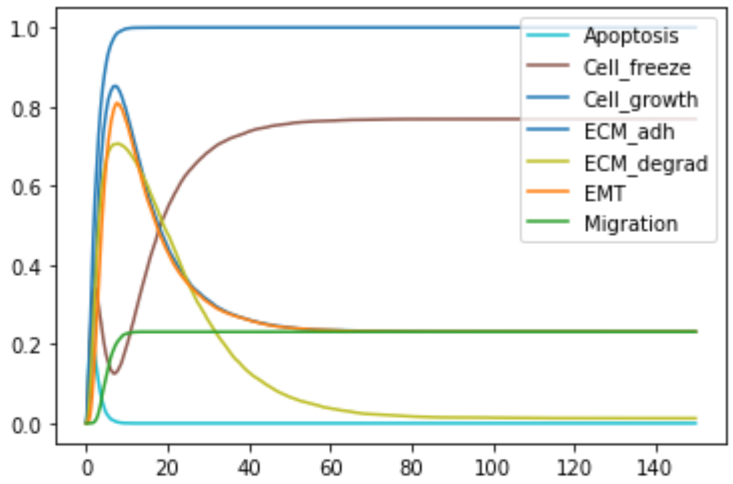

The rupture of nuclear envelope, cause the **activation of p63** that as conquence **triggers the secretion of MMPs**. This allows the degradation of the ECM and diminuish the pressure on the nucleus. Without DNA damage, the cell can undergo EMT and invade. However, for high value of max time, the percentage of cells that can degrade ECM is very low (1.26%). To cope with this problem, we want to look at the transient effects that are more evident for max time < 20.

In [29]:
```
i_condition3_2 = intracellular_model.copy()
i_condition3_2.param["max_time"] = 20
maboss.set_nodes_istate(i_condition3_2, i_condition3_2.network.keys(), off)
i_condition3_2.network.set_istate("Oxy",on)
i_condition3_2.network.set_istate("GF",on)
i_condition3_2.network.set_istate("Neigh",on)
i_condition3_2.network.set_istate("ECM",on)
i_condition3_2.network.set_istate("TGFbeta",on)
i_condition3_2.network.set_istate("DNAdamage",on)
maboss.set_output(i_condition3_2, set_output1)
```

In [30]:
```
res4_2 = i_condition3_2.run()
res4_2.plot_piechart()
```

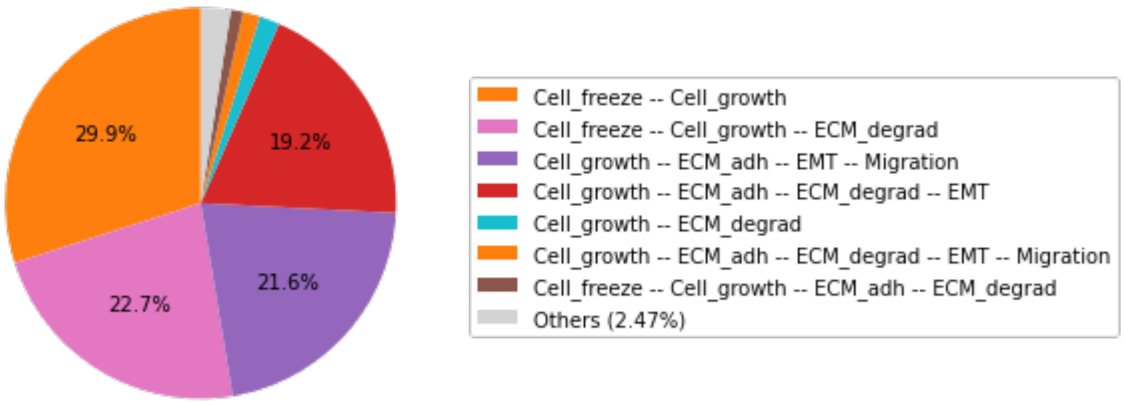

In [31]:
```
res4_2.plot_node_trajectory()
for phenotype in set_output1:
    try:
        print(phenotype, ": ", res4_2.get_last_nodes_probtraj()[phenotype].values[0])
    except:
        print(phenotype, ": ", 0)
```

```
Apoptosis :  0
Migration :  0.22990000000000002
ECM_adh :  0.442705
EMT :  0.434918
Cell_growth :  0.9999999999999999
ECM_degrad :  0.47975500000000004
Cell_freeze :  0.542064
```

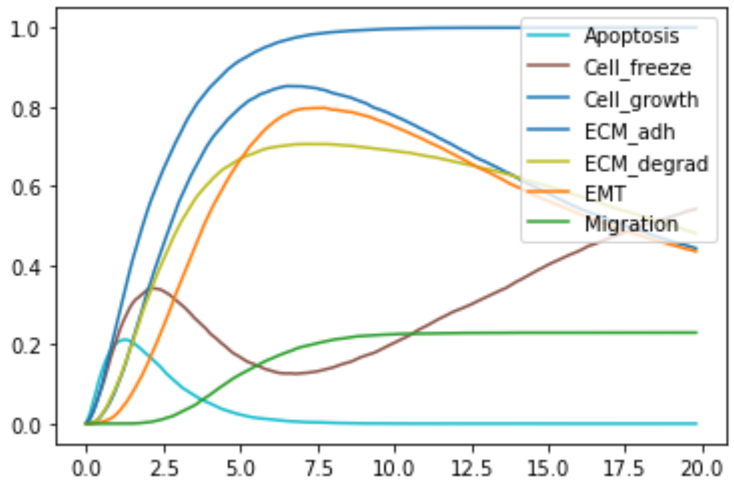

At time step == 20 the percentage of cells able to degrade the ECM is around 48%. To take this into account in the PhysiBoSS simulation we set the MaBoSS max_time to 12. Doing so, the transient phenotypes of MaBoSS will be more evident in PhysiBoSS simulation.

In the following case, we explore the effect of DNAdamage on the model in **absence of growth factor (GF) and oxygen** for the portion of cells **not in contact with the ECM**.

In [29]:
```
i_condition4 = intracellular_model.copy()
i_condition4.param["max_time"] = 30
maboss.set_nodes_istate(i_condition4, i_condition4.network.keys(), off)
i_condition4.network.set_istate("Oxy",off)
i_condition4.network.set_istate("GF",off)
i_condition4.network.set_istate("Neigh",on)
i_condition4.network.set_istate("ECM",off)
i_condition4.network.set_istate("TGFbeta",off)
i_condition4.network.set_istate("DNAdamage",on)
maboss.set_output(i_condition4, set_output1)
```

In [30]:
```
res5 = i_condition4.run()
res5.plot_piechart()
```

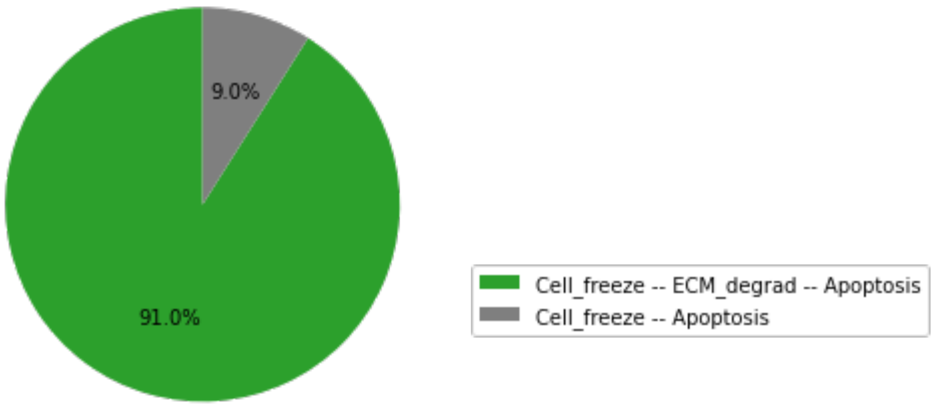

In [31]:
```
res5.plot_node_trajectory()
for phenotype in set_output1:
    try:
        print(phenotype, ": ", res5.get_last_nodes_probtraj()[phenotype].values[0])
    except:
        print(phenotype, ": ", 0)
```

```
Apoptosis :  1.0
Migration :  0
ECM_adh :  0
EMT :  0
Cell_growth :  0
ECM_degrad :  0.9102
Cell_freeze :  1.0
```

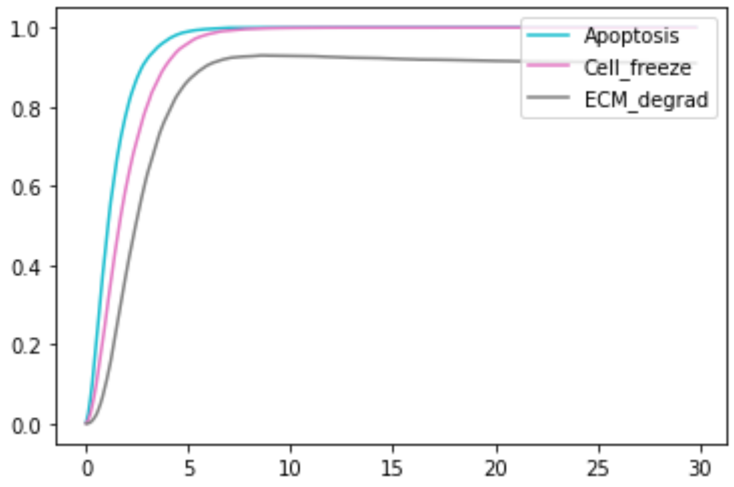

We notice how, in absence of GF, the model changed to an apoptotic phenotype. The growth factor input activates the ERK node in the intracellular model, which is necessary to substain the growth of the cells. In the model presented in the paper we decided to focus on the invasion aspect. For this reason, we run all the simulations in the best condition for the cells to grow justifying the presence of GF in most of our simulations.

Mutants' simulations

The model can be used to perform in silico experiments and test the importance of each component of the model and their contribution to the phenotypes. We tested the knock out and knock in on some of the components of the model below.

CTNNB1 mutant

In [32]:
```
# Initial condition corresponds to i_condition2 defined in the wild type simulation
mutant2_simulation = maboss.copy_and_mutate(i_condition, ["CTNNB1"], "ON")

mut_res = mutant2_simulation.run()

mut_res.plot_piechart()

mut_res.plot_node_trajectory()
#mut_res.get_last_nodes_probtraj()["ECM_degrad"].values[0]
```

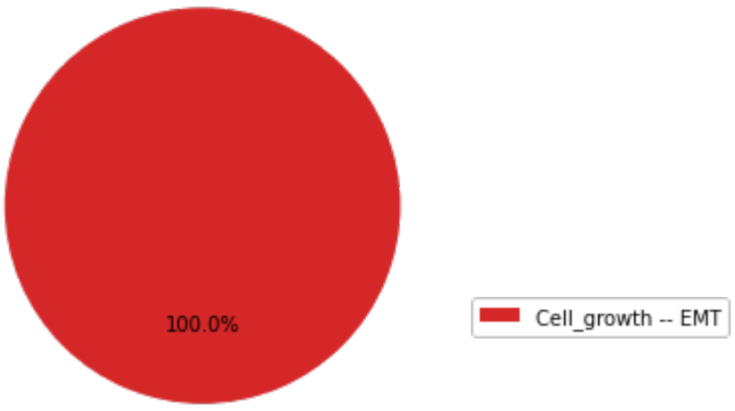

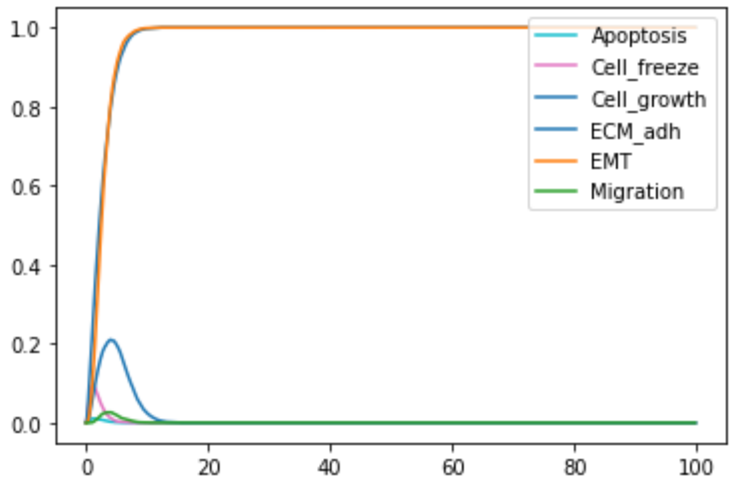

Conclusion: beta-catenin could be a potential target in the model to stop the release of MMPs and so the process of metastatis. However, this mutation does not prevent the cells from going through the EMT process and the tumor to growth.

TP63 mutants

TP63 overexpression

In [55]:
```
# Initial condition corresponds to a random initial condition as defined in the wild type simulation
intracellular_model.param["max_time"] = 40
mutant_simulation = maboss.copy_and_mutate(intracellular_model, ["p63"], "ON")
maboss.set_output(mutant_simulation, set_output1)

mut_res = mutant_simulation.run()

emt = mut_res.get_last_nodes_probtraj()["EMT"].values[0]
degrad = mut_res.get_last_nodes_probtraj()["ECM_degrad"].values[0]

print("Percentage EMT: ", emt)
print("Percentage ECM_degrad: ", degrad)

mut_res.plot_piechart()
```

```
Percentage EMT:  0.588096
Percentage ECM_degrad:  1.0
```

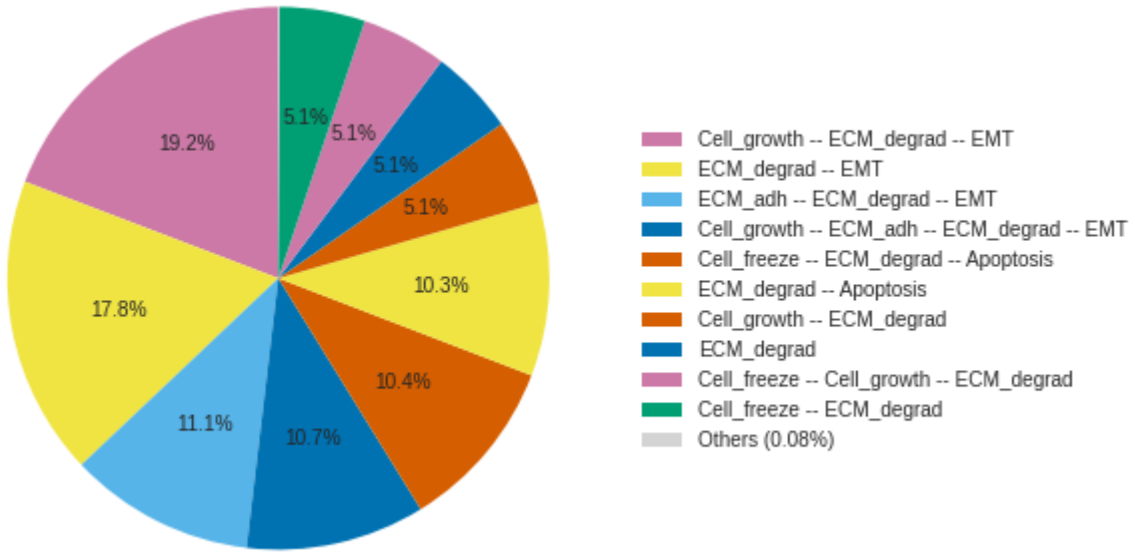

In [56]:
```
mut_res.plot_node_trajectory()
for phenotype in set_output1:
    try:
        print(phenotype, ": ", mut_res.get_last_nodes_probtraj()[phenotype].values[0])
    except:
        print(phenotype, ": ", 0)
```

```
Apoptosis :  0.2072
Migration :  0
ECM_adh :  0.21800000000000003
EMT :  0.588096
Cell_growth :  0.40159999999999996
ECM_degrad :  1.0
Cell_freeze :  0.20589999999999997
```

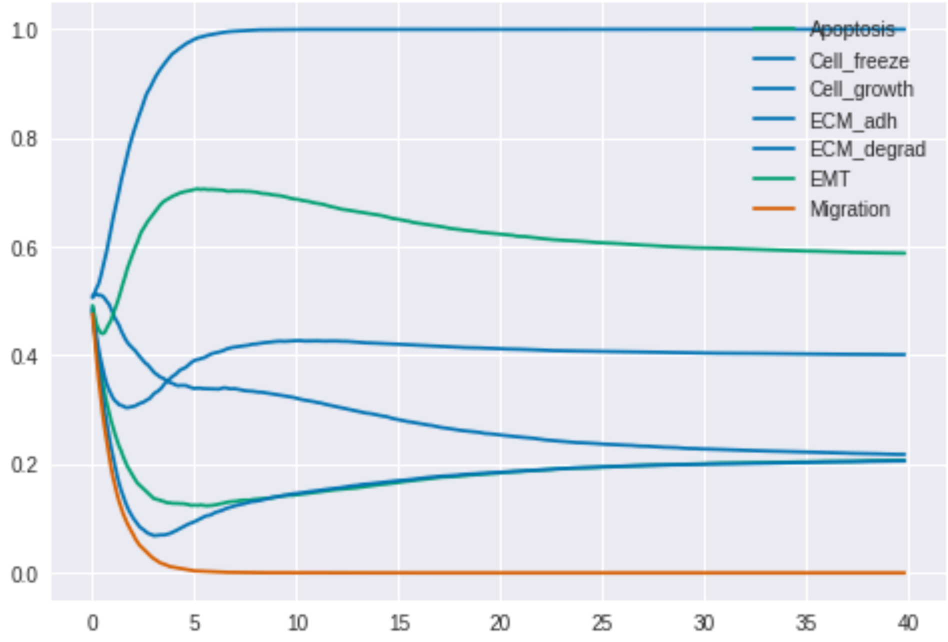

Conclusion: overexpression of p63 on WT model increase the release of MMPs up to 100%, matching the results obtained from Lodillinski et al.

TP63 knock out

In [57]:
```
mutant_simulation = maboss.copy_and_mutate(intracellular_model, ["p63"], "OFF")
maboss.set_output(mutant_simulation, set_output1)

mut_res = mutant_simulation.run()

mut_res.plot_piechart()

emt = mut_res.get_last_nodes_probtraj()["EMT"].values[0]
degrad = mut_res.get_last_nodes_probtraj()["ECM_degrad"].values[0]

print("Percentage EMT: ", emt)
print("Percentage ECM_degrad: ", degrad)
```

```
Percentage EMT:  0.620065
Percentage ECM_degrad:  0.25196500000000005
/home/marco/anaconda3/envs/MaBoSS_env/lib/python3.9/site-packages/maboss/figures.py:86: MatplotlibDeprecationWarni
ng: normalize=None does not normalize if the sum is less than 1 but this behavior is deprecated since 3.3 until tw
o minor releases later. After the deprecation period the default value will be normalize=True. To prevent normaliz
ation pass normalize=False
  ax.pie(plot_line, labels=plotting_labels, radius=1.2,
```

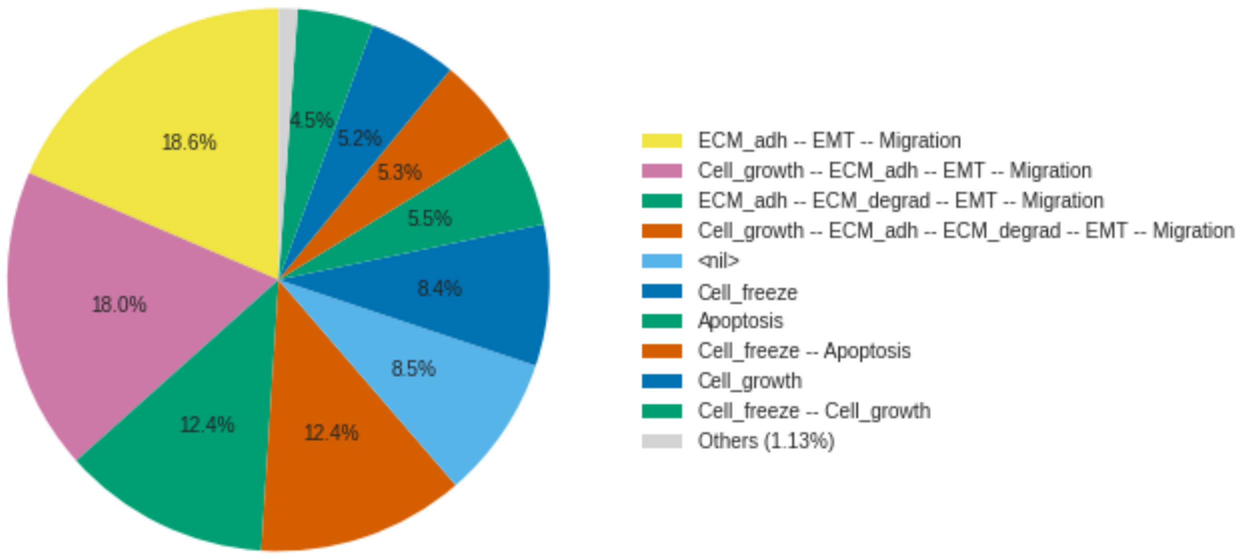

Conclusion: The knock-out of p63 on WT model partially decreases the release of MMPs. To have a better understanding of the impact of overexpression of p63, we simulate next two different scenarios, with different initial conditions.

In [58]:
```
mut_res.plot_node_trajectory()
for phenotype in set_output1:
    try:
        print(phenotype, ": ", mut_res.get_last_nodes_probtraj()[phenotype].values[0])
    except:
        print(phenotype, ": ", 0)
```

```
Apoptosis :  0.10909999999999999
Migration :  0.6150760000000001
ECM_adh :  0.6134000000000001
EMT :  0.620065
Cell_growth :  0.406
ECM_degrad :  0.25196500000000005
Cell_freeze :  0.18439999999999998
```

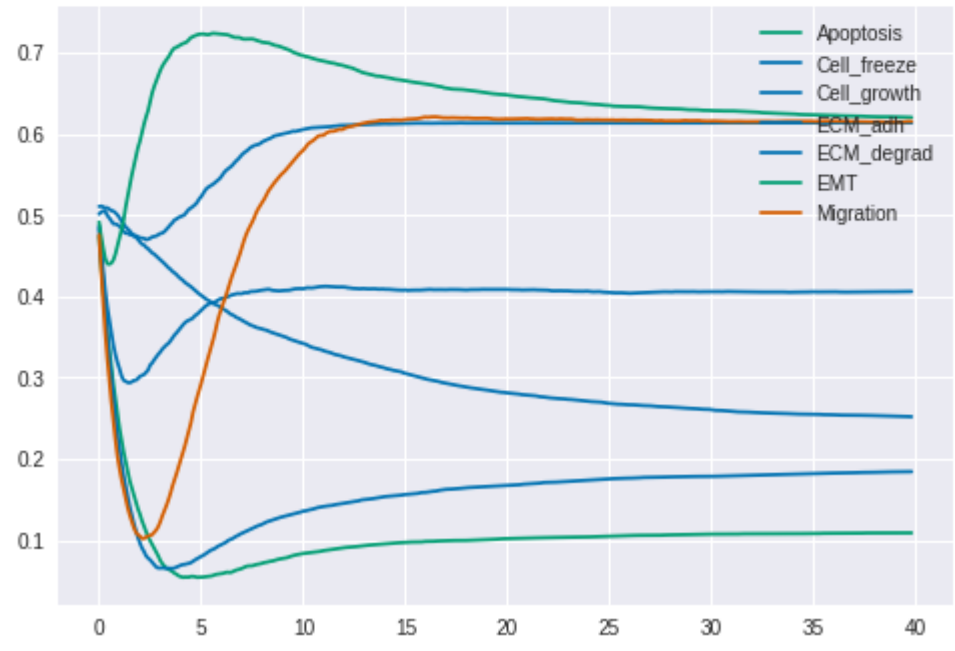

TP63 mutant with ECM/TGFbeta initial condition

TP63 overexpression

In [50]:
```
mutant_simulation = maboss.copy_and_mutate(i_condition, ["p63"], "ON")
maboss.set_output(mutant_simulation, set_output1)

mut_res = mutant_simulation.run()

mut_res.plot_piechart()

try:
    emt = mut_res.get_last_nodes_probtraj()["EMT"].values[0]
except:
    emt = 0

degrad = mut_res.get_last_nodes_probtraj()["ECM_degrad"].values[0]

print("Percentage EMT: ", emt)
print("Percentage ECM_degrad: ", degrad)
```

```
Percentage EMT:  1.0
Percentage ECM_degrad:  1.0
```

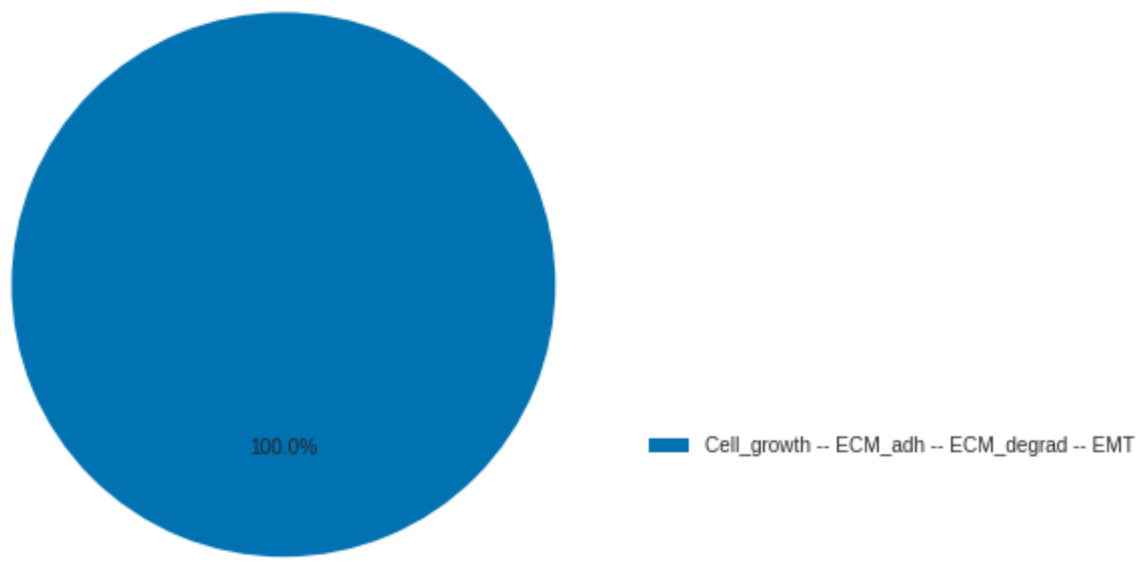

Conclusion: the initial condition applied for this case, simulate those cells at the border of the tumor in direct contact with the ECM. Overexpression of p63 leads totally leads to MMPs release which cause the degradation of the ECM. At the same time, p63 inhibit migration. This is in accord with the results from Lodillinski et al.

TP63 knock out

In [51]:
```
mutant_simulation = maboss.copy_and_mutate(i_condition, ["p63"], "OFF")
maboss.set_output(mutant_simulation, set_output1)

mut_res = mutant_simulation.run()

mut_res.plot_piechart()

try:
    emt = mut_res.get_last_nodes_probtraj()["EMT"].values[0]
except:
    emt = 0
try:
    degrad = mut_res.get_last_nodes_probtraj()["ECM_degrad"].values[0]
except:
    degrad = 0

print("Percentage EMT: ", emt)
print("Percentage ECM_degrad: ", degrad)
```

```
Percentage EMT:  1.0
Percentage ECM_degrad:  0
```

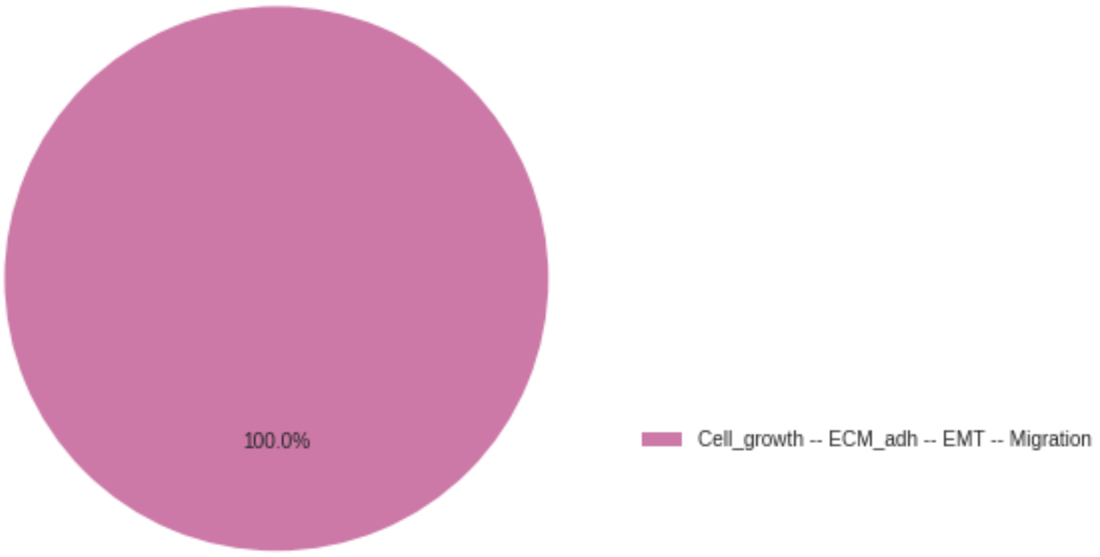

Conclusion: The knock-out of p63 in this scenario decreases the release of MMPs and allows the transition to a mesenchymal state. This is partially in accordance with the results from Lodillinsky: Knock-out of p63 should totally block MMPs release, confining the cells and avoiding metastasis.

SRC mutant

In this simulation, we test the effect of SRC activation on the model.

SRC overexpression

In [38]:
```
mutant_simulation5 = maboss.copy_and_mutate(intracellular_model, ["SRC"], "ON")

mut_res5 = mutant_simulation5.run()

mut_res5.plot_piechart()

print("Migration: ", mut_res5.get_last_nodes_probtraj()["Migration"].values[0])
print("EMT: ", mut_res5.get_last_nodes_probtraj()["EMT"].values[0])
```

```
Migration:  0.7613
EMT:  1.0
/Users/laurence/opt/miniconda3/envs/colosys/lib/python3.7/site-packages/maboss/figures.py:87: MatplotlibDeprecatio
nWarning: normalize=None does not normalize if the sum is less than 1 but this behavior is deprecated since 3.3 un
til two minor releases later. After the deprecation period the default value will be normalize=True. To prevent no
rmalization pass normalize=False
  startangle=90, colors=color_list, **opts)
```

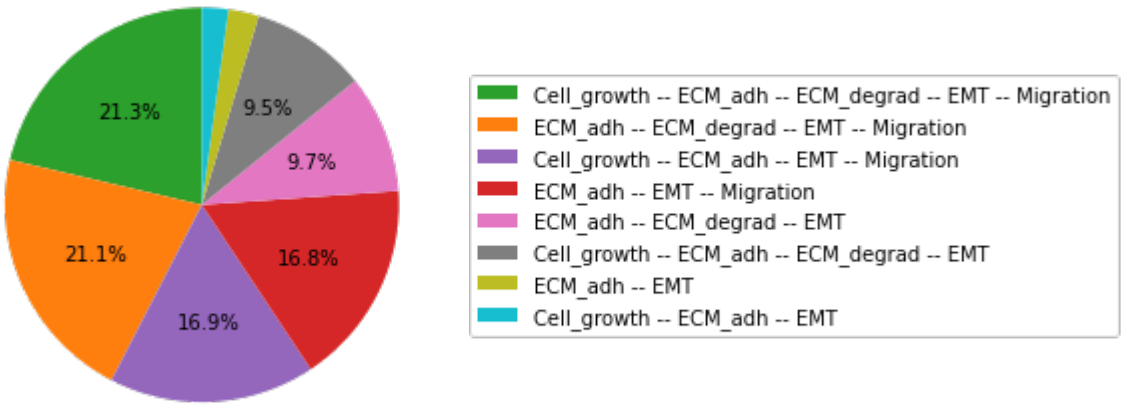

Conclusion: SRC overexpression in WT model increase the possibility for migration and EMT.

SRC knock out

In [39]:
```
intracellular_model.param["max_time"] = 100
mutant_simulation6 = maboss.copy_and_mutate(intracellular_model, ["SRC"], "OFF")

mut_res6 = mutant_simulation6.run()

mut_res6.plot_piechart()

print("Migration: ", mut_res6.get_last_nodes_probtraj()["Migration"].values[0])
print("EMT: ", mut_res6.get_last_nodes_probtraj()["EMT"].values[0])
```

```
Migration:  0.1827
EMT:  0.37970000000000004
```

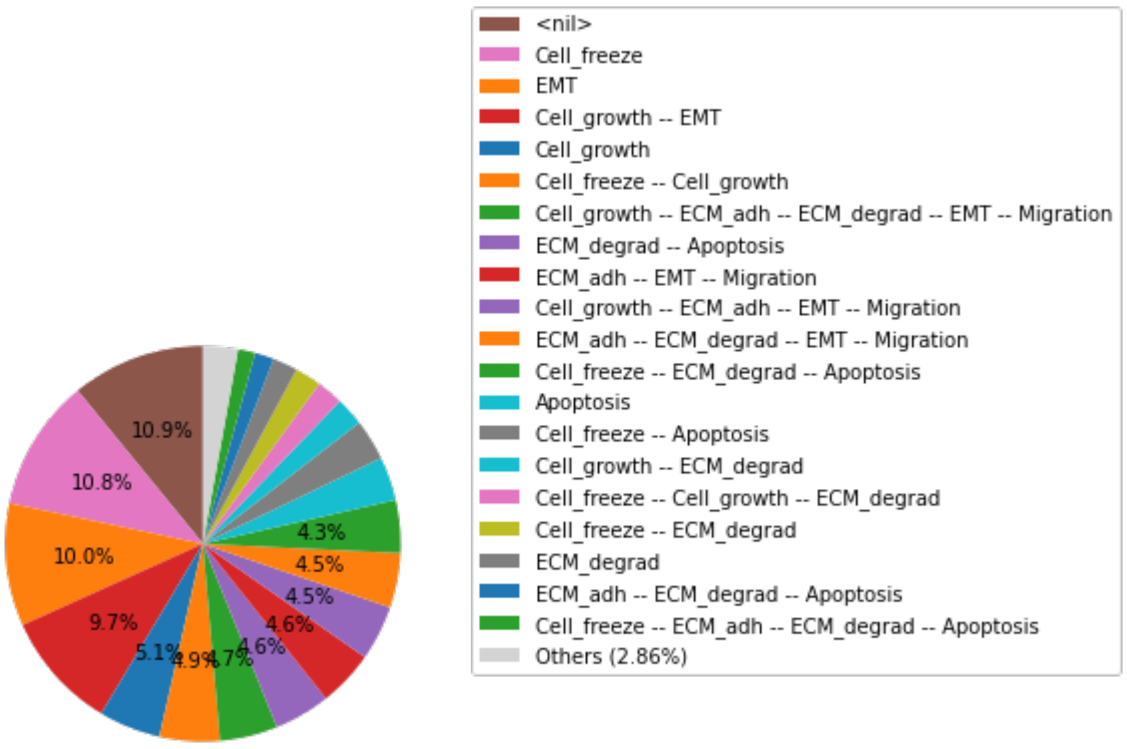

In [40]:
```
#mut_res6.plot_trajectory()
```

In [41]:
```
mut_res6.get_nodes_probtraj().plot(legend=True)
```

Out[41]: <AxesSubplot:>

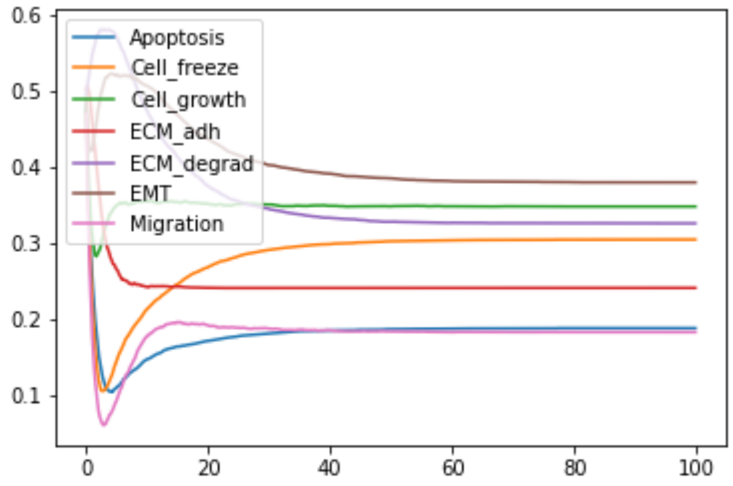

Conclusion: Knock-out of SRC partially inhibits the formation of mesenchymal phenotypes. This is in accordance with the fact that SRC is involved in many mechanisms, like adhesion, migration and degradation of ECM, forming complexes with FAK and promoting mesenchymal phenotype.

SRC mutant without ECM/TGFbeta initial condition (center of the monolayer)

SRC overexpression

In [42]:
```
mutant_simulation7 = maboss.copy_and_mutate(i_condition2, ["SRC"], "ON")

mut_res7 = mutant_simulation7.run()

mut_res7.plot_piechart()

print("Migration: ", mut_res7.get_last_nodes_probtraj()["Migration"].values[0])
print("EMT: ", mut_res7.get_last_nodes_probtraj()["EMT"].values[0])
```

```
Migration:  1.0
EMT:  1.0
```

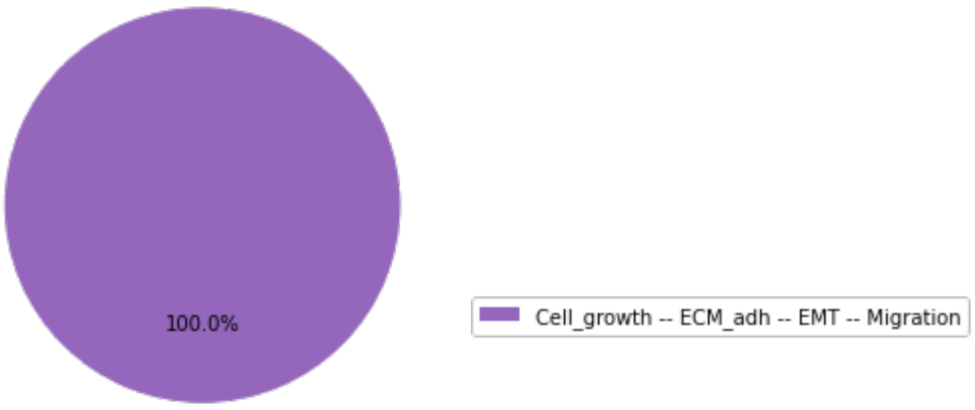

Conclusion: the results obtained are in accordance with the experimental results (see main article). Cells in the middle of the monolayer, upon activation of SRC, develop a mesenchymal-like phenotype.

SRC knock out

In [43]:
```
mutant_simulation8 = maboss.copy_and_mutate(i_condition2, ["SRC"], "OFF")

mut_res8 = mutant_simulation8.run()

mut_res8.plot_piechart()

#print(mut_res8.get_last_nodes_probtraj()["Migration"].values[0])
#print(mut_res8.get_last_nodes_probtraj()["EMT"].values[0])
```

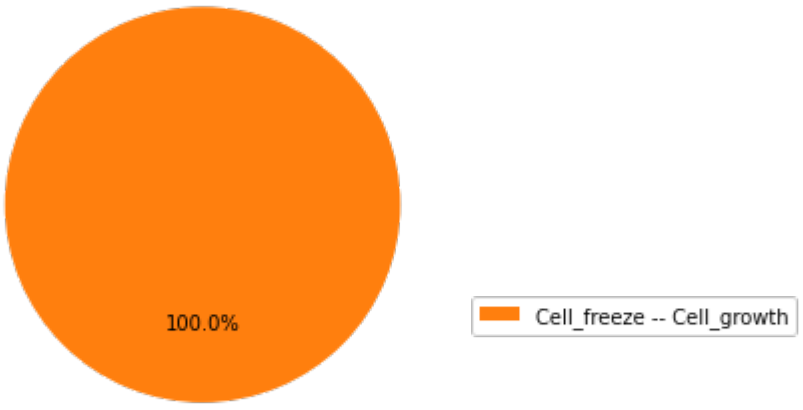

Conclusion: knock-out of SRC prevents the mesenchymal phenotype in cells in the middle of the layer.

Model testing

In this section, we use a tool we developed, MaboSS_test to verify that the model is able to reproduce observations or reported behaviors. These experimental observations are translated into a computer-readable format for fast-checking.

In [44]:
```
init_condition = {"Oxy":[0, 1], "Neigh":[0, 1], "GF":[0, 1], "DNAdamage":[1, 0], "ECM":[0, 1]}
```

In [45]:
```
p63_inhibition = {"p63":"OFF"}
p63_activation = {"p63":"ON"}
```

In [46]:
```
SRC_inhibition = {"SRC":"OFF"}
SRC_activation = {"SRC":"ON"}
```

In this simulation, we test that when p63 is overexpressed (or constitutively active), the probability of activating MMPs increase when compared to the wild type case.

For all the tests below the same format is applied: [mutation to simulate, initial condition, observable (which read-out), behavior (increase, decrease)]

In [47]:
```
test_model = i_condition.copy()
run_test_model = maboss_test.MaBoSSTestCase(test_model)

run_test_model.assertStateProbabilityEvolution(p63_activation, init_condition, {"MMPs":1}, "increase")
```

```
True!
The new probability of reaching the state is: 1.0
The old one is: 0
```

We then verify that the probability to activate MMPs decreases in the case of TP63 knock out.

In [48]:
```
test_model = i_condition.copy()
run_test_model = maboss_test.MaBoSSTestCase(test_model)

run_test_model.assertStateProbabilityEvolution(p63_inhibition, init_condition, {"MMPs":1}, "decrease")
```

```
False!
The new probability of reaching the state is: 0
The old one is: 0
```

We then verify that the probability to activate EMT increase in the case of SRC overexpression.

In [49]:
```
test_model = i_condition2.copy()
run_test_model = maboss_test.MaBoSSTestCase(test_model)

run_test_model.assertStateProbabilityEvolution(SRC_activation, init_condition, {"EMT":1}, "increase")
```

```
False!
The new probability of reaching the state is: 1.0
The old one is: 1.0
```

We then verify that the probability to activate EMT decrease in the case of SRC knock-out.

In [50]:
```
test_model = i_condition2.copy()
run_test_model = maboss_test.MaBoSSTestCase(test_model)

run_test_model.assertStateProbabilityEvolution(SRC_inhibition, init_condition, {"EMT":1}, "decrease")
```

```
False!
The new probability of reaching the state is: 1.0
The old one is: 1.0
```

We then verify that the probability to activate Migration increase in the case of SRC overexpression.

In [51]:
```
test_model = i_condition2.copy()
run_test_model = maboss_test.MaBoSSTestCase(test_model)

run_test_model.assertStateProbabilityEvolution(SRC_activation, init_condition, {"Migration":1}, "increase")
```

```
False!
The new probability of reaching the state is: 1.0
The old one is: 1.0
```

We then verify that the probability to activate Migration decrease in the case of SRC knock-out.

In [52]:
```
test_model = i_condition2.copy()
run_test_model = maboss_test.MaBoSSTestCase(test_model)

run_test_model.assertStateProbabilityEvolution(SRC_inhibition, init_condition, {"Migration":1}, "decrease")
```

```
True!
The new probability of reaching the state is: 0
The old one is: 1.0
```

Conclusion: we verified that in the intracellular model, p63 directly control the activation of MMPs, proving to be a good candidate to stop tumor progression, inhibiting the release of matrix metalloproteases. On the other hand, overexpression of SRC leads those cells in the middle of the monolayer, in an epithelial state, to differentiate into mesenchymal phenotype. Knock-out of SRC instead, block this differentiation, preventing the switch to mesenchymal phenotype.

Automatic (single/double) mutations

Here we perform a sensititvity analysis on the model, triggering one or two mutations at the time over the nodes on the network. The purpose is to search for single or double mutants that exhibit interesting phenotypes, such as complete loss of migration, apoptosis, etc.

In [53]:
```
def simulate_single_mutants(model, list_nodes, sign="BOTH"):

    list_single_mutants = []
    if (sign == "BOTH" or sign == "ON"):
        list_single_mutants += [(node, "ON") for node in list_nodes]
    if (sign == "BOTH" or sign == "OFF"):
        list_single_mutants += [(node, "OFF") for node in list_nodes]

    res = {}
    for single_mutant in list_single_mutants:
        t_model = model.copy()
        t_model.mutate(*single_mutant)
        res.update({single_mutant: t_model.run()})

    return res
```

In [54]:
```
def simulate_double_mutants(model, list_nodes, sign="BOTH"):

    list_single_mutants = []
    if (sign == "BOTH" or sign == "ON"):
        list_single_mutants += [(node, "ON") for node in list_nodes]
    if (sign == "BOTH" or sign == "OFF"):
        list_single_mutants += [(node, "OFF") for node in list_nodes]

    list_double_mutants = [(a, b) for idx, a in enumerate(list_single_mutants) for b in list_single_mutants[idx + 1

    res = {}
    for double_mutant in list_double_mutants:
        t_model = model.copy()
        t_model.mutate(*(double_mutant[0]))
        t_model.mutate(*(double_mutant[1]))
        res.update({double_mutant: t_model.run()})

    return res
```

In [55]:
```
def filter_sensititivy(results, state=None, node=None, minimum=None, maximum=None):
    ret_res = {}
    for (mutant, res) in results.items():

        if state is not None:
            t_res = res.get_last_states_probtraj()
            if state in t_res.columns:
                if minimum is not None and maximum is not None:
                    if t_res[state].values[0] > minimum and t_res[state].values[0] < maximum:
                        ret_res.update({mutant: res})
                elif minimum is not None and t_res[state].values[0] > minimum:
                    ret_res.update({mutant: res})
                elif maximum is not None and t_res[state].values[0] < maximum:
                    ret_res.update({mutant: res})
            elif maximum is not None and minimum is None:
                ret_res.update({mutant:res})

        elif node is not None:

            t_res = res.get_last_nodes_probtraj()

            if node in t_res.columns:
                if minimum is not None and maximum is not None:
                    if t_res[node].values[0] > minimum and t_res[node].values[0] < maximum:
                        ret_res.update({mutant: res})
                elif minimum is not None and t_res[node].values[0] > minimum:
                    ret_res.update({mutant: res})
                elif maximum is not None and t_res[node].values[0] < maximum:
                    ret_res.update({mutant: res})
            elif maximum is not None and minimum is None:
                ret_res.update({mutant: res})

    return ret_res
```

In [56]:
```
intracellular_model.network.keys()

target_genes = ["AKT1", "CTNNB1", 'DKK1', 'AKT2', 'RAC1', 'YAP1', 'PIK3CA', 'ERK', 'p21', 'SMAD', 'VIM', 'p53'
               , 'p63', 'p73', 'miR203', 'miR34', 'miR200', 'TGFbetaR', 'FAK']
```

In [57]:
```
i_condition4 = intracellular_model.copy()
i_condition4.param["max_time"] = 30
maboss.set_nodes_istate(i_condition4, i_condition4.network.keys(), off)
i_condition4.network.set_istate("Oxy",on)
i_condition4.network.set_istate("GF",on)
i_condition4.network.set_istate("Neigh",off)
i_condition4.network.set_istate("ECM",on)
i_condition4.network.set_istate("TGFbeta",on)
i_condition4.network.set_istate("DNAdamage",off)
maboss.set_output(i_condition4, set_output1)
```

In [58]:
```
res_single = simulate_single_mutants(i_condition, target_genes)
for mutant, res in res_single.items():
    print(mutant)
    res.plot_piechart()
    plt.title(mutant)
```

```
('AKT1', 'ON')
('CTNNB1', 'ON')
('DKK1', 'ON')
('AKT2', 'ON')
('RAC1', 'ON')
('YAP1', 'ON')
('PIK3CA', 'ON')
('ERK', 'ON')
('p21', 'ON')
('SMAD', 'ON')
('VIM', 'ON')
('p53', 'ON')
('p63', 'ON')
('p73', 'ON')
('miR203', 'ON')
('miR34', 'ON')
('miR200', 'ON')
('TGFbetaR', 'ON')
('FAK', 'ON')
('AKT1', 'OFF')
('CTNNB1', 'OFF')
('DKK1', 'OFF')
('AKT2', 'OFF')
('RAC1', 'OFF')
('YAP1', 'OFF')
('PIK3CA', 'OFF')
('ERK', 'OFF')
('p21', 'OFF')
('SMAD', 'OFF')
/Users/laurence/opt/miniconda3/envs/colosys/lib/python3.7/site-packages/maboss/results/baseresult.py:102: RuntimeW
arning: More than 20 figures have been opened. Figures created through the pyplot interface (`matplotlib.pyplot.fi
gure`) are retained until explicitly closed and may consume too much memory. (To control this warning, see the rcP
aram `figure.max_open_warning`).
  _, axes = plt.subplots(1,1)
('VIM', 'OFF')
('p53', 'OFF')
('p63', 'OFF')
('p73', 'OFF')
('miR203', 'OFF')
('miR34', 'OFF')
('miR200', 'OFF')
('TGFbetaR', 'OFF')
('FAK', 'OFF')
```

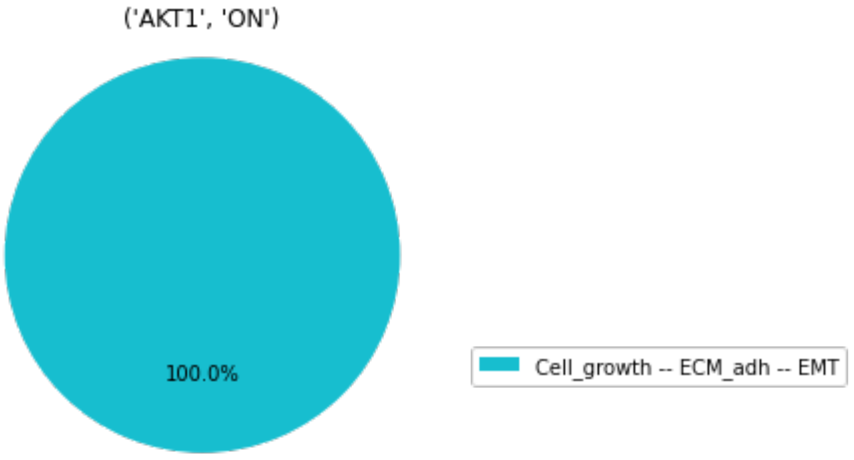

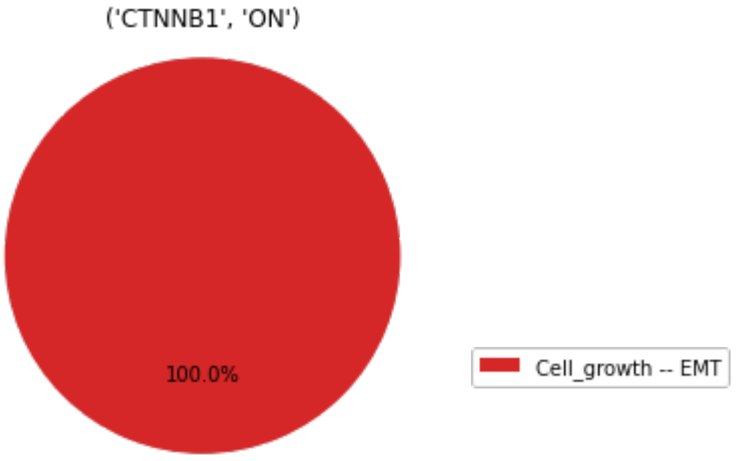

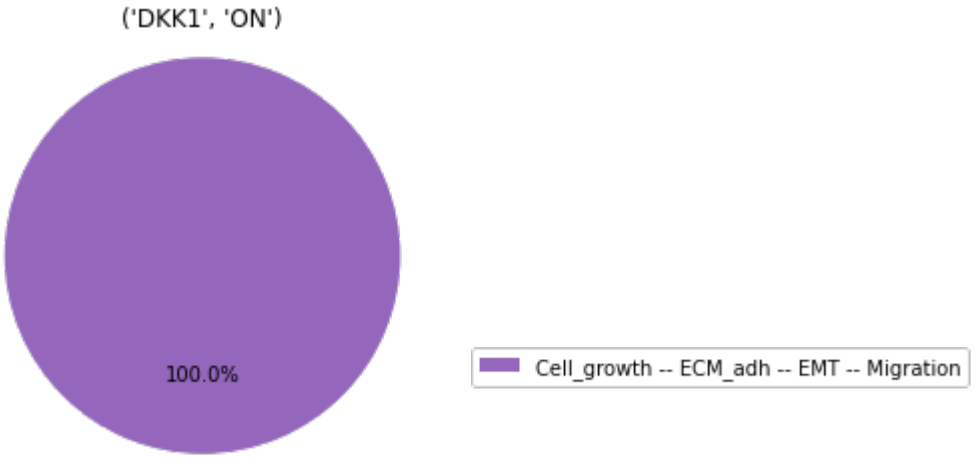

('AKT2', 'ON')

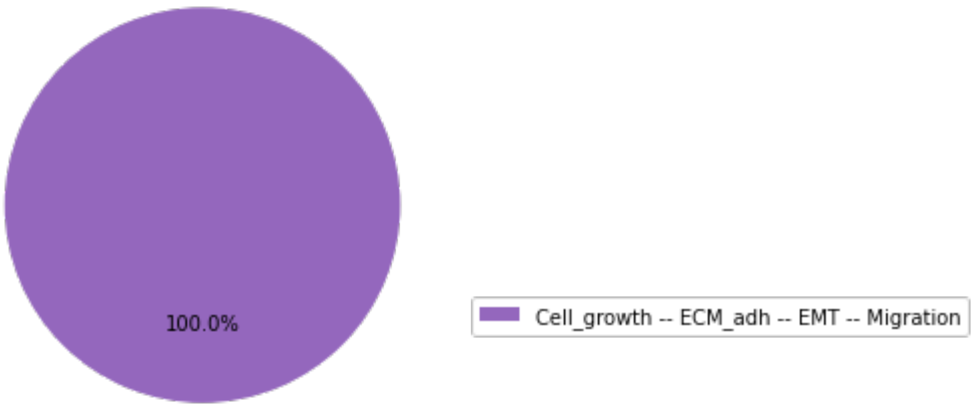

('RAC1', 'ON')

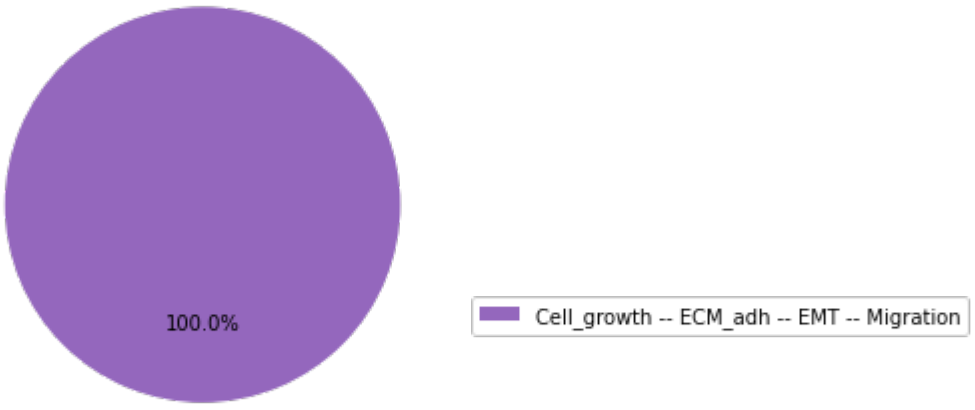

('YAP1', 'ON')

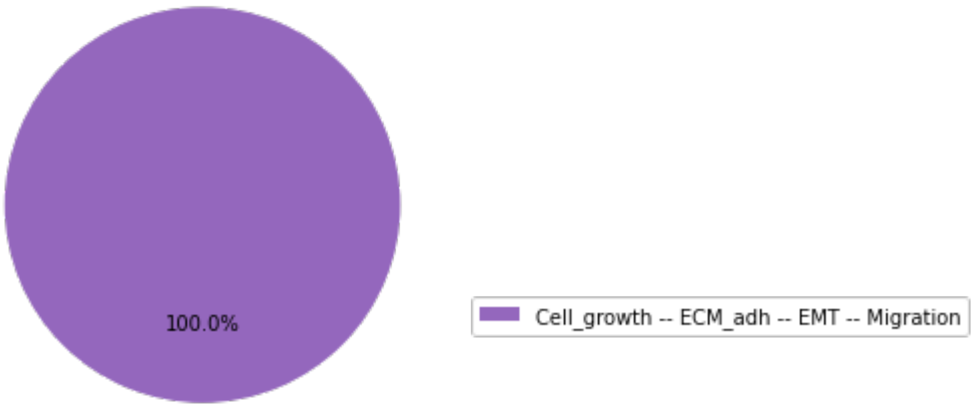

('PIK3CA', 'ON')

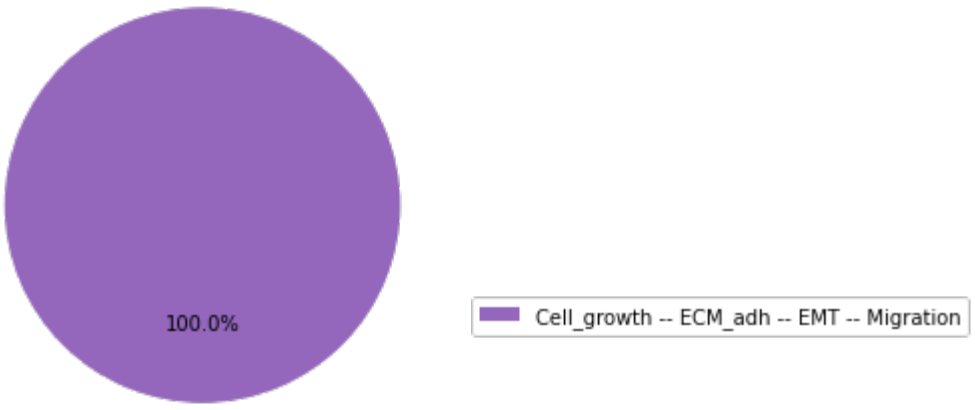

('ERK', 'ON')

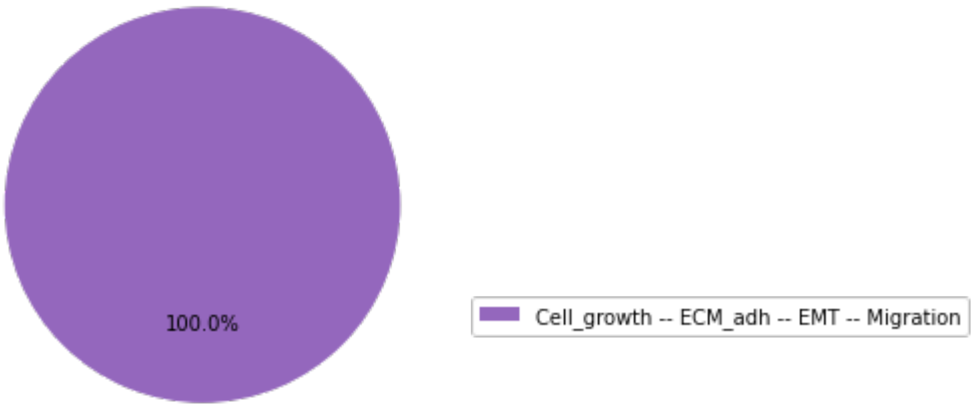

('p21', 'ON')

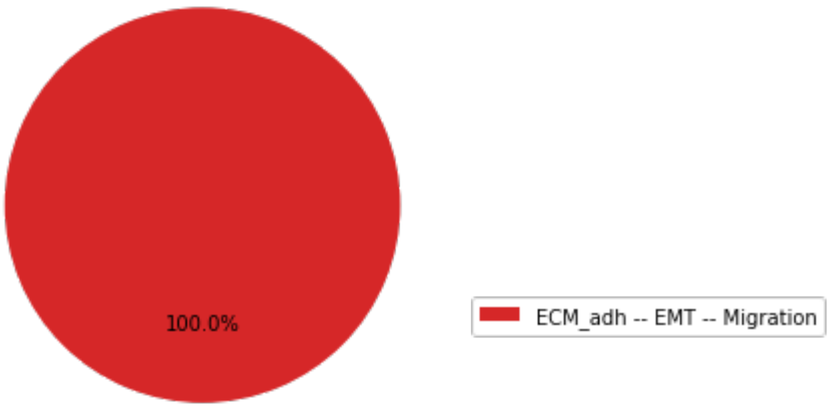

('SMAD', 'ON')

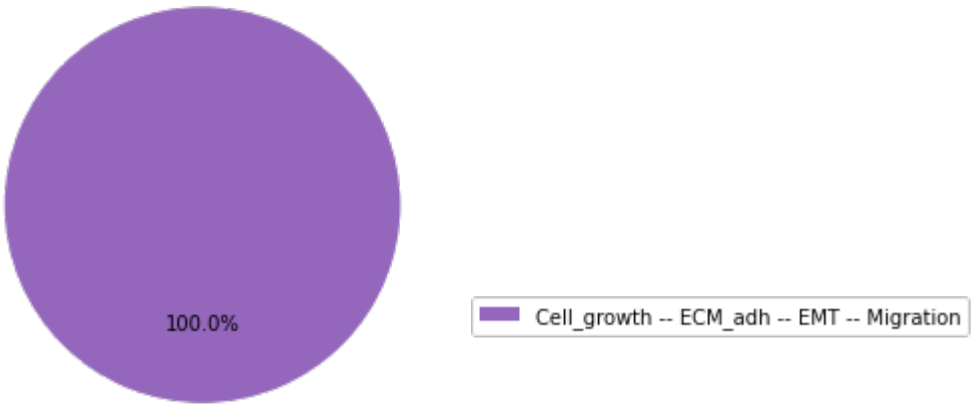

('VIM', 'ON')

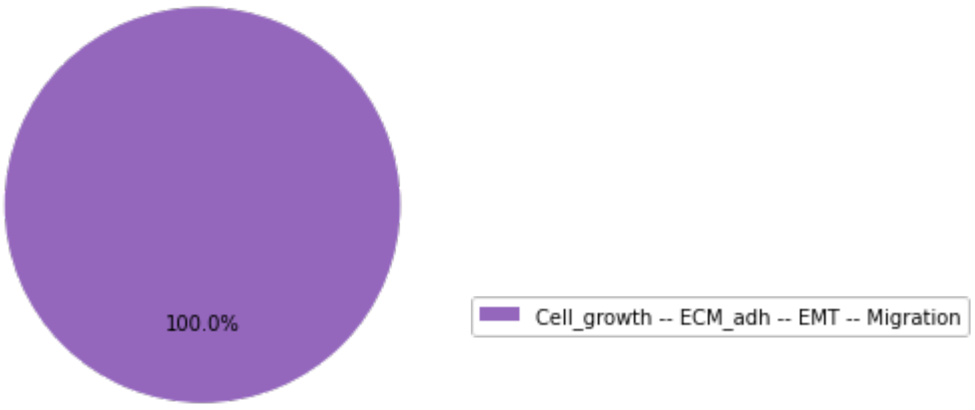

('p53', 'ON')

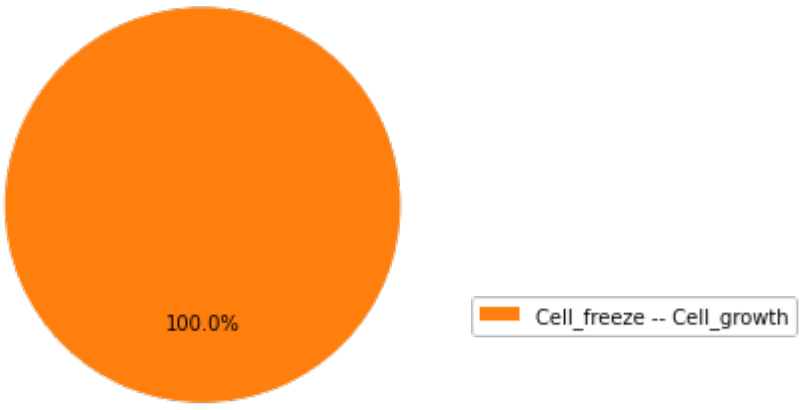

('p63', 'ON')

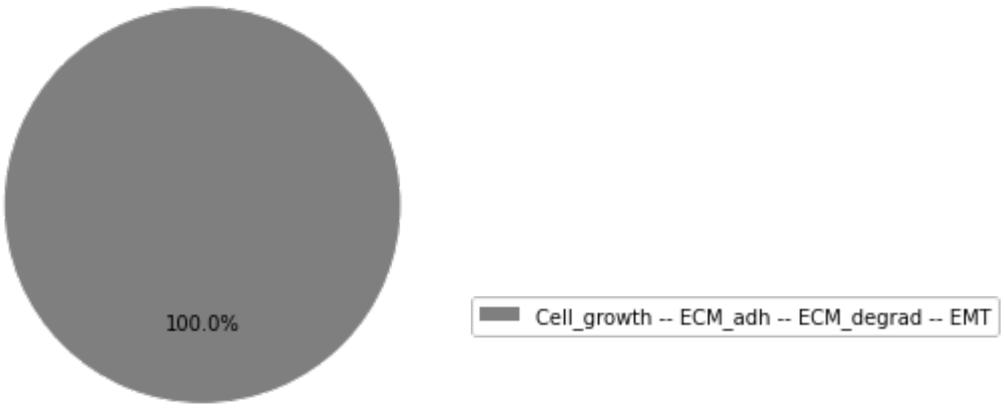

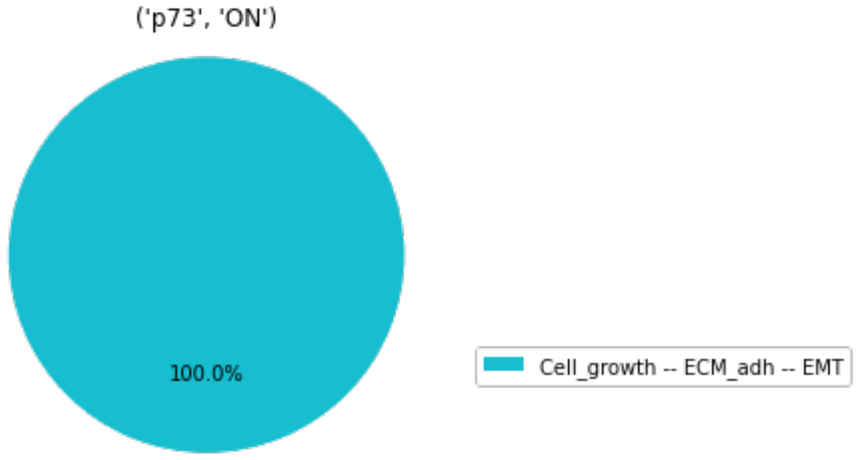
('p73', 'ON')
100.0%
Cell_growth -- ECM_adh -- EMT

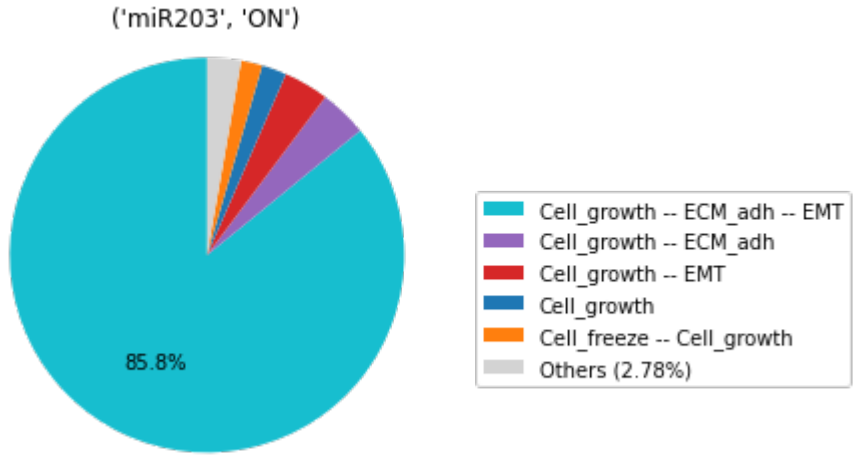
('miR203', 'ON')
85.8%
Cell_growth -- ECM_adh -- EMT
Cell_growth -- ECM_adh
Cell_growth -- EMT
Cell_growth
Cell_freeze -- Cell_growth
Others (2.78%)

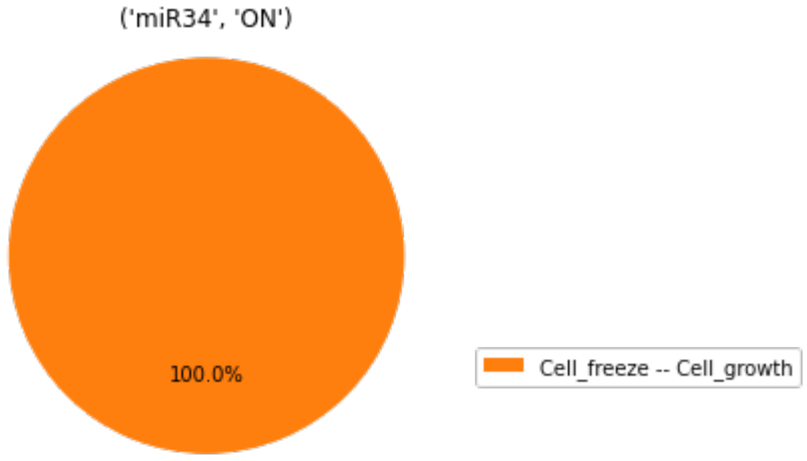
('miR34', 'ON')
100.0%
Cell_freeze -- Cell_growth

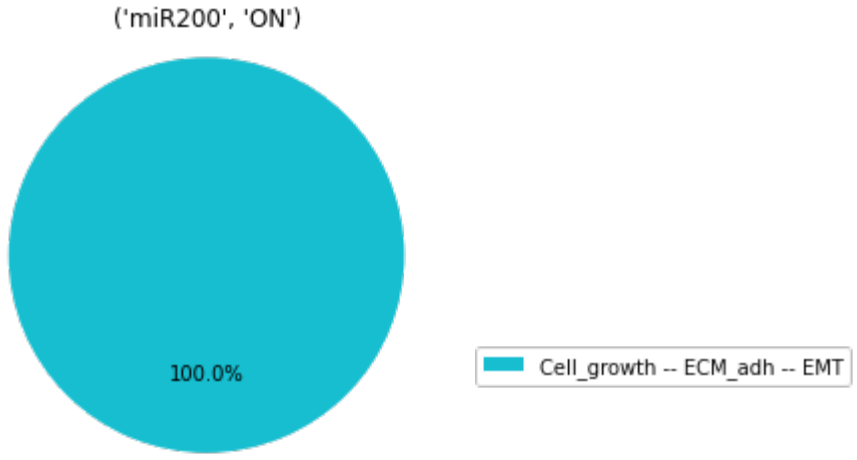
('miR200', 'ON')
100.0%
Cell_growth -- ECM_adh -- EMT

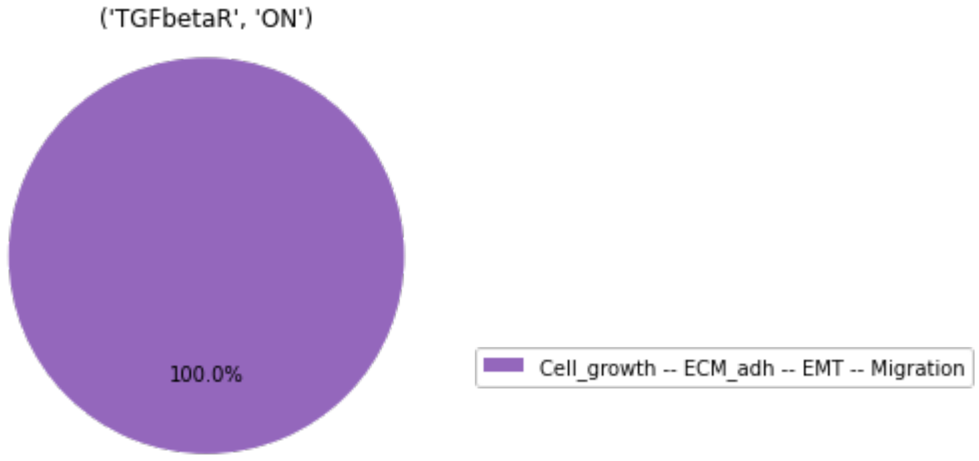
('TGFbetaR', 'ON')
100.0%
Cell_growth -- ECM_adh -- EMT -- Migration

('FAK', 'ON')

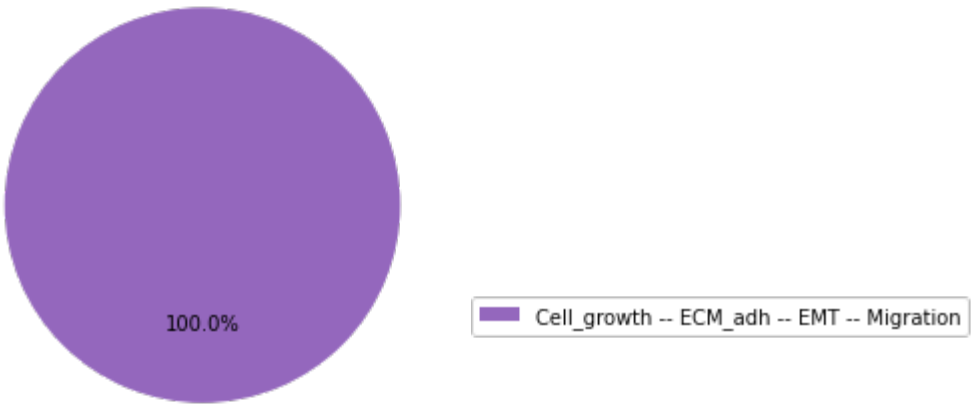

('AKT1', 'OFF')

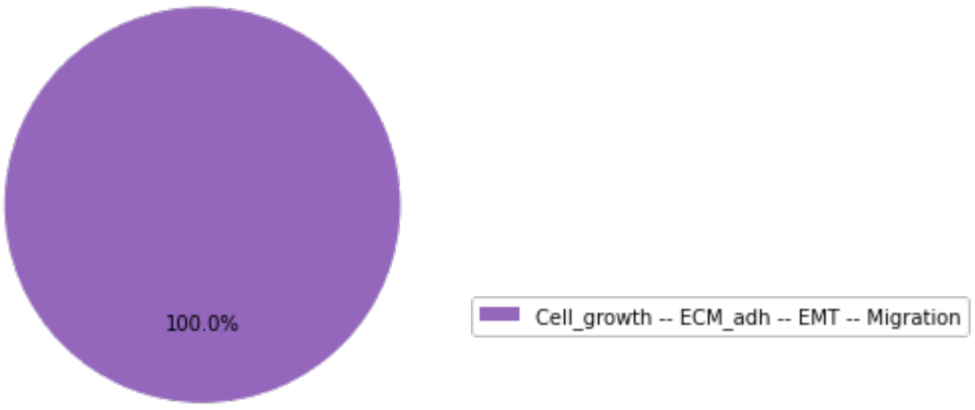

('CTNNB1', 'OFF')

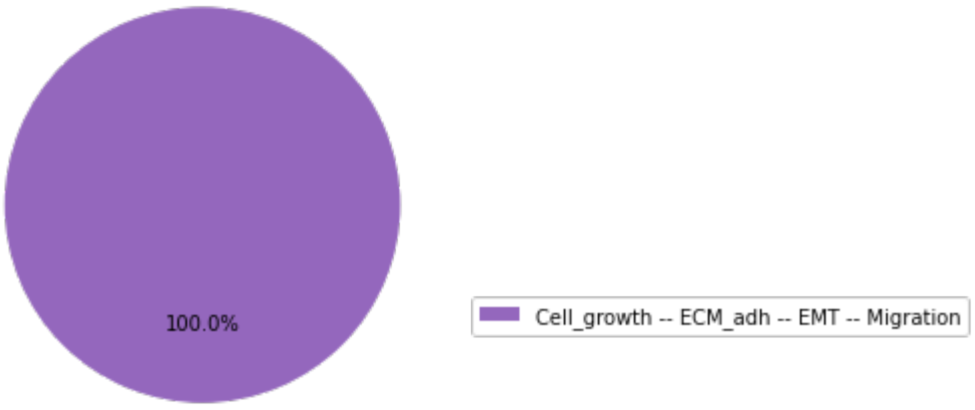

('DKK1', 'OFF')

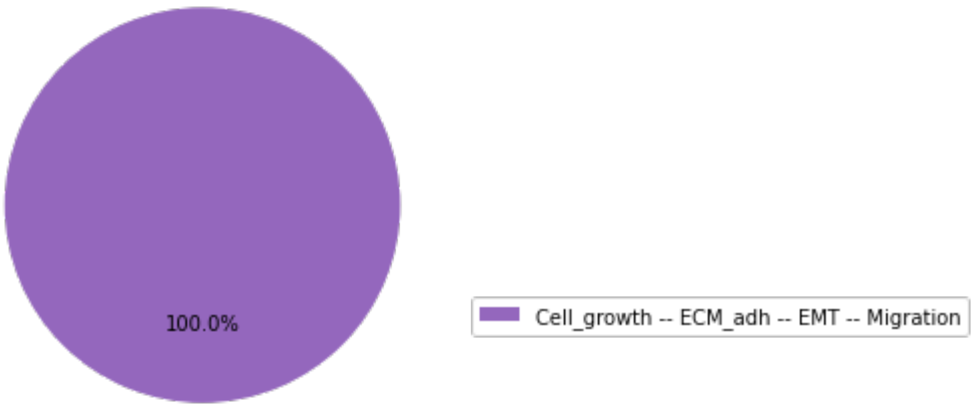

('AKT2', 'OFF')

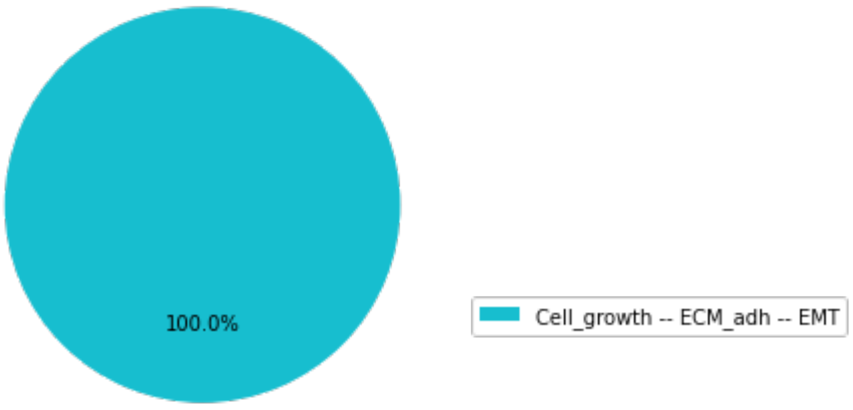

('RAC1', 'OFF')

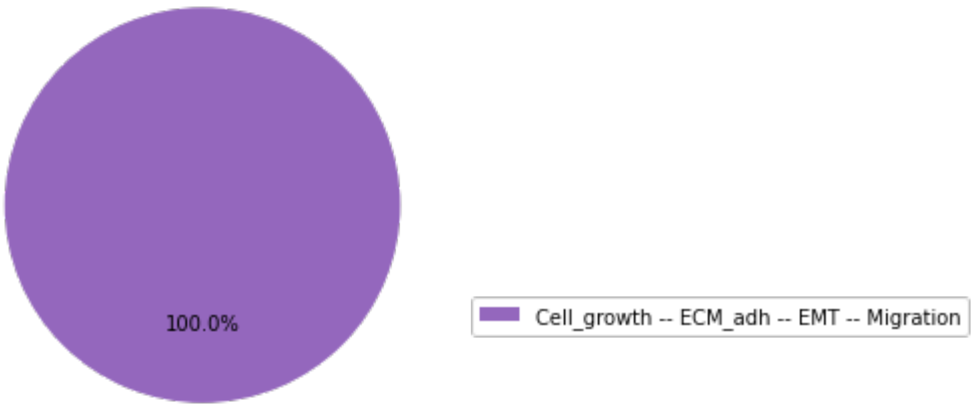

('YAP1', 'OFF')

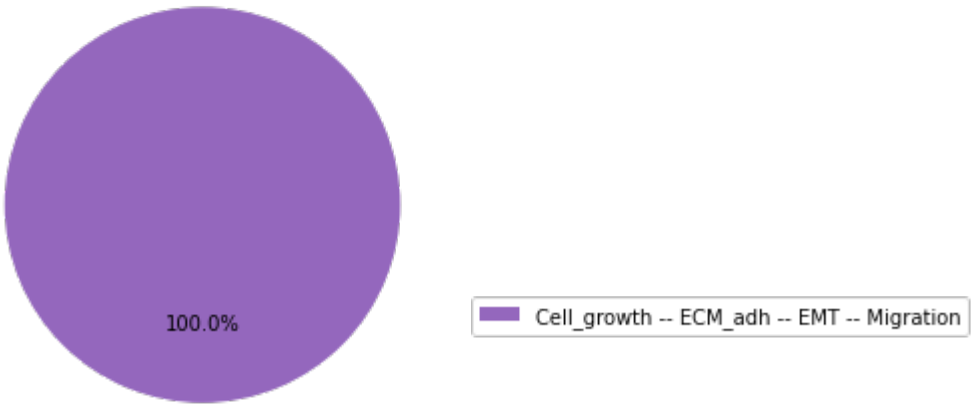

('PIK3CA', 'OFF')

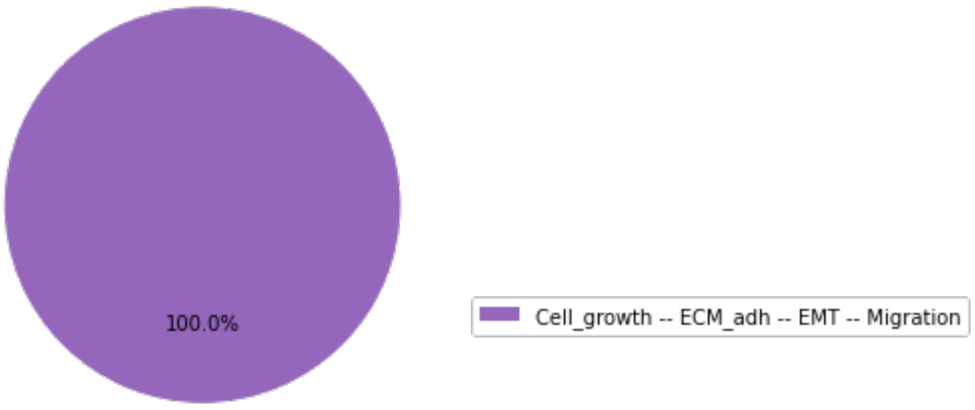

('ERK', 'OFF')

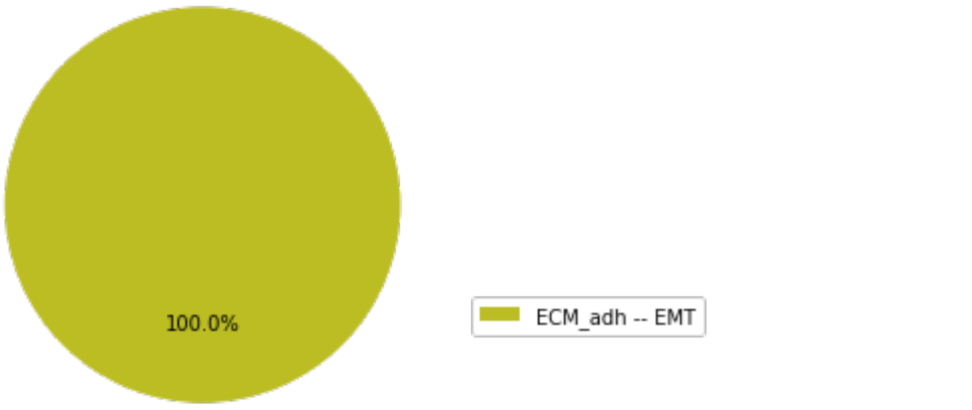

('p21', 'OFF')

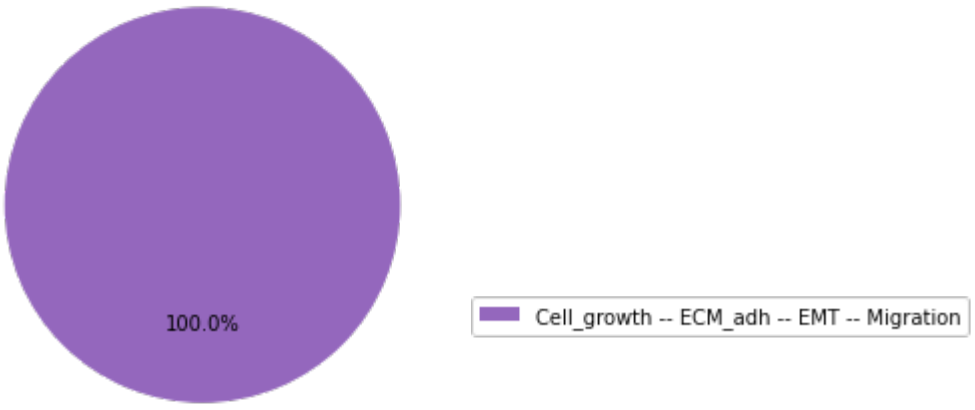

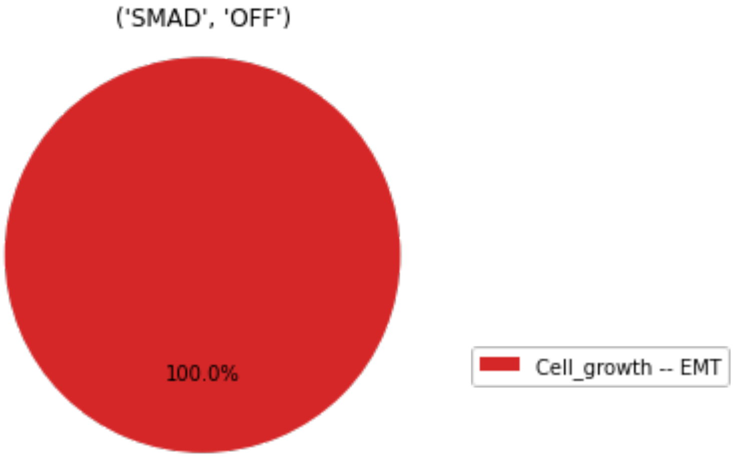
('SMAD', 'OFF')
100.0%
Cell_growth -- EMT

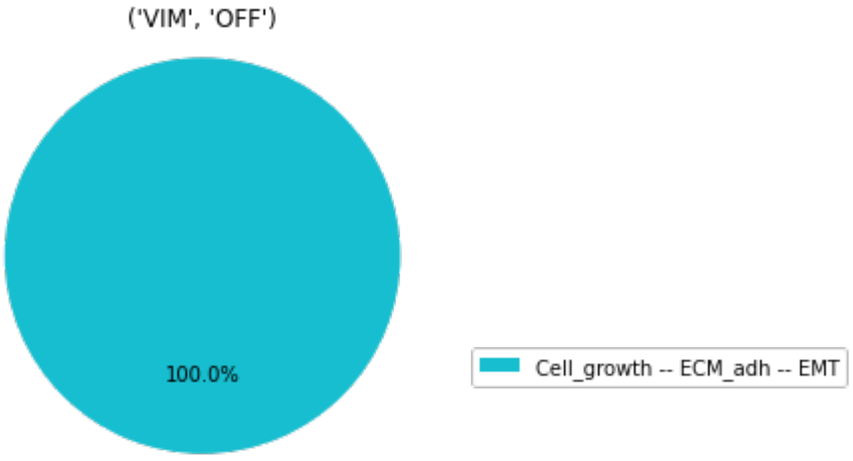
('VIM', 'OFF')
100.0%
Cell_growth -- ECM_adh -- EMT

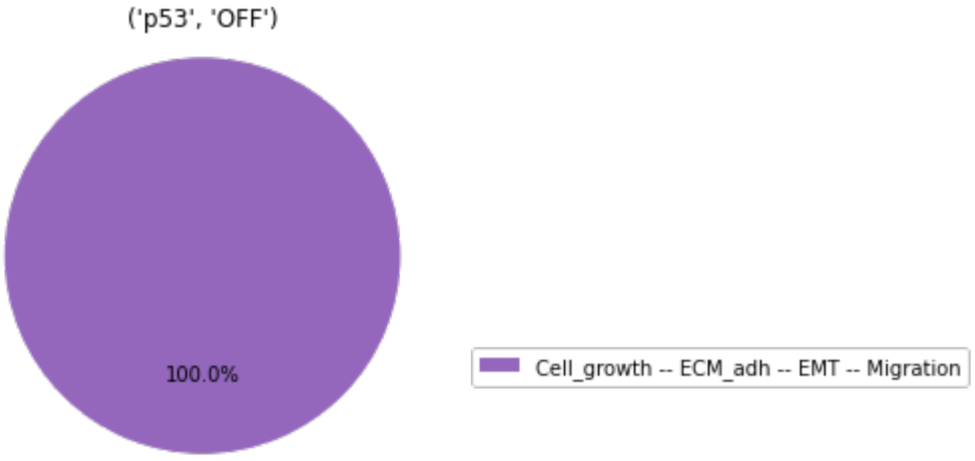
('p53', 'OFF')
100.0%
Cell_growth -- ECM_adh -- EMT -- Migration

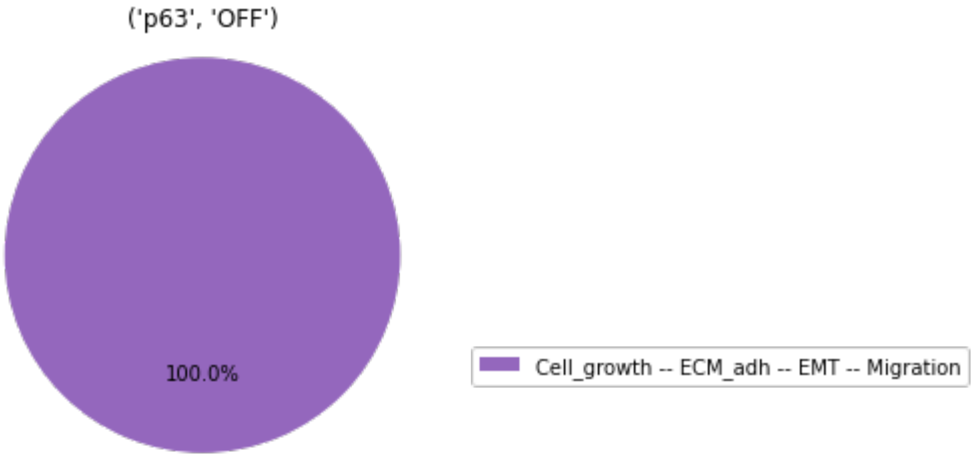
('p63', 'OFF')
100.0%
Cell_growth -- ECM_adh -- EMT -- Migration

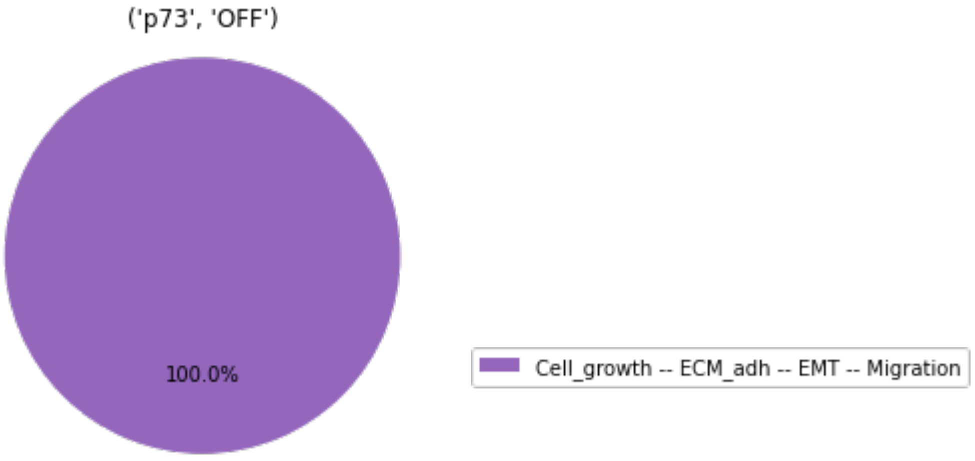
('p73', 'OFF')
100.0%
Cell_growth -- ECM_adh -- EMT -- Migration

('miR203', 'OFF')

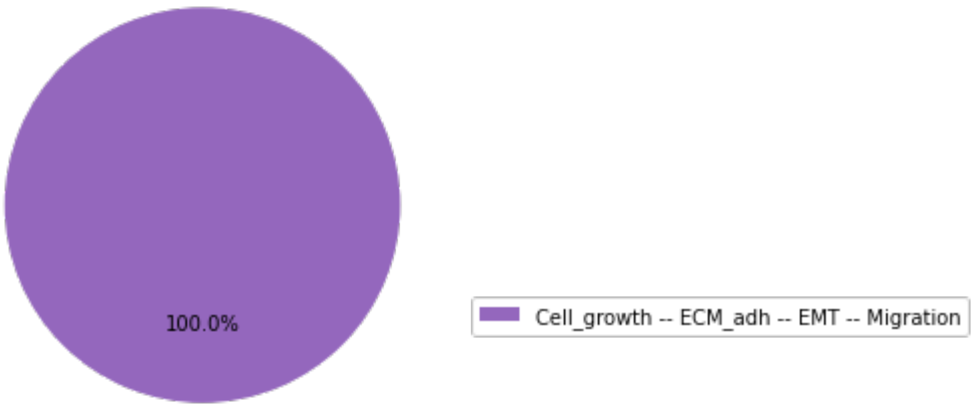

('miR34', 'OFF')

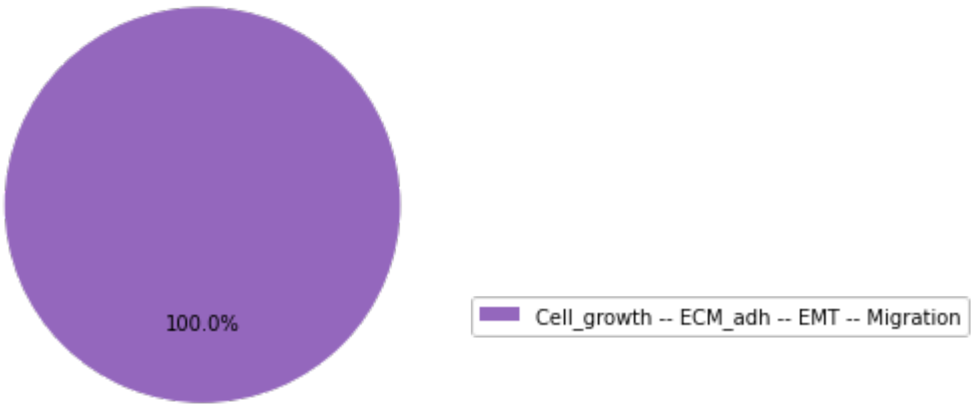

('miR200', 'OFF')

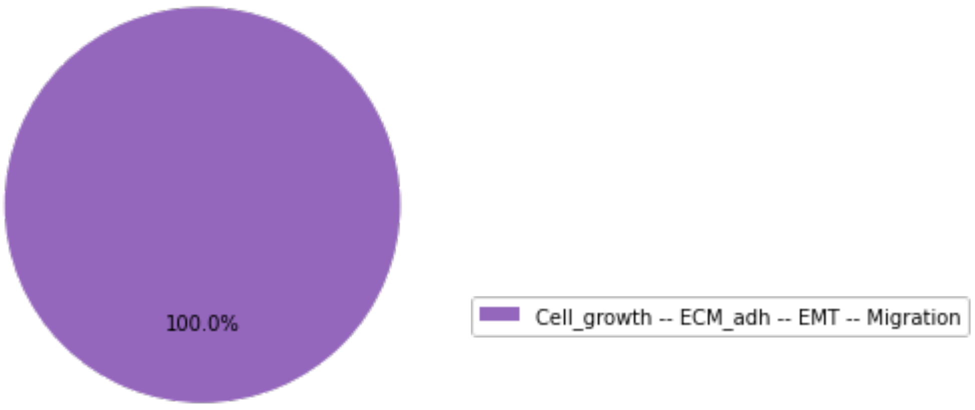

('TGFbetaR', 'OFF')

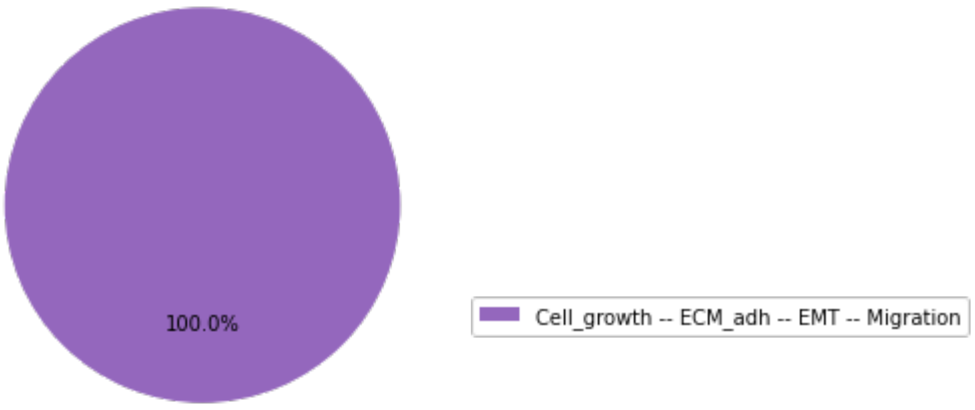

('FAK', 'OFF')

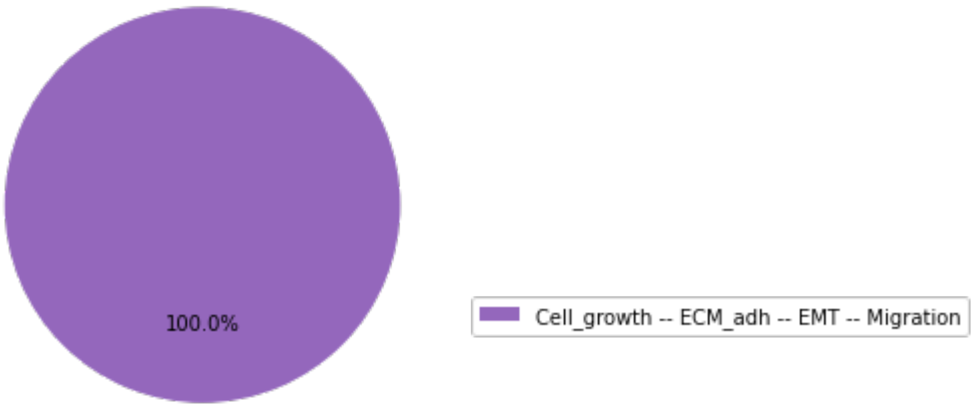

In [59]:

```
res_double = simulate_double_mutants(i_condition, target_genes)
for mutant, res in res_double.items():
    print(mutant)
    res.plot_piechart()
    plt.title(mutant)
```

```
(('AKT1', 'ON'), ('CTNNB1', 'ON'))
(('AKT1', 'ON'), ('DKK1', 'ON'))
(('AKT1', 'ON'), ('AKT2', 'ON'))
(('AKT1', 'ON'), ('RAC1', 'ON'))
(('AKT1', 'ON'), ('YAP1', 'ON'))
(('AKT1', 'ON'), ('PIK3CA', 'ON'))
(('AKT1', 'ON'), ('ERK', 'ON'))
(('AKT1', 'ON'), ('p21', 'ON'))
(('AKT1', 'ON'), ('SMAD', 'ON'))
(('AKT1', 'ON'), ('VIM', 'ON'))
(('AKT1', 'ON'), ('p53', 'ON'))
(('AKT1', 'ON'), ('p63', 'ON'))
(('AKT1', 'ON'), ('p73', 'ON'))
(('AKT1', 'ON'), ('miR203', 'ON'))
(('AKT1', 'ON'), ('miR34', 'ON'))
(('AKT1', 'ON'), ('miR200', 'ON'))
(('AKT1', 'ON'), ('TGFbetaR', 'ON'))
(('AKT1', 'ON'), ('FAK', 'ON'))
(('AKT1', 'ON'), ('CTNNB1', 'OFF'))
(('AKT1', 'ON'), ('DKK1', 'OFF'))
(('AKT1', 'ON'), ('AKT2', 'OFF'))
(('AKT1', 'ON'), ('RAC1', 'OFF'))
(('AKT1', 'ON'), ('YAP1', 'OFF'))
(('AKT1', 'ON'), ('PIK3CA', 'OFF'))
(('AKT1', 'ON'), ('ERK', 'OFF'))
(('AKT1', 'ON'), ('p21', 'OFF'))
(('AKT1', 'ON'), ('SMAD', 'OFF'))
(('AKT1', 'ON'), ('VIM', 'OFF'))
/Users/laurence/opt/miniconda3/envs/colosys/lib/python3.7/site-packages/maboss/results/baseresult.py:102: RuntimeW
arning: More than 20 figures have been opened. Figures created through the pyplot interface (`matplotlib.pyplot.fi
gure`) are retained until explicitly closed and may consume too much memory. (To control this warning, see the rcP
aram `figure.max_open_warning`).
  _, axes = plt.subplots(1,1)
```

```
(('AKT1', 'ON'), ('p53', 'OFF'))
(('AKT1', 'ON'), ('p63', 'OFF'))
(('AKT1', 'ON'), ('p73', 'OFF'))
(('AKT1', 'ON'), ('miR203', 'OFF'))
(('AKT1', 'ON'), ('miR34', 'OFF'))
(('AKT1', 'ON'), ('miR200', 'OFF'))
(('AKT1', 'ON'), ('TGFbetaR', 'OFF'))
(('AKT1', 'ON'), ('FAK', 'OFF'))
(('CTNNB1', 'ON'), ('DKK1', 'ON'))
(('CTNNB1', 'ON'), ('AKT2', 'ON'))
(('CTNNB1', 'ON'), ('RAC1', 'ON'))
(('CTNNB1', 'ON'), ('YAP1', 'ON'))
(('CTNNB1', 'ON'), ('PIK3CA', 'ON'))
(('CTNNB1', 'ON'), ('ERK', 'ON'))
(('CTNNB1', 'ON'), ('p21', 'ON'))
(('CTNNB1', 'ON'), ('SMAD', 'ON'))
(('CTNNB1', 'ON'), ('VIM', 'ON'))
(('CTNNB1', 'ON'), ('p53', 'ON'))
(('CTNNB1', 'ON'), ('p63', 'ON'))
(('CTNNB1', 'ON'), ('p73', 'ON'))
(('CTNNB1', 'ON'), ('miR203', 'ON'))
(('CTNNB1', 'ON'), ('miR34', 'ON'))
(('CTNNB1', 'ON'), ('miR200', 'ON'))
(('CTNNB1', 'ON'), ('TGFbetaR', 'ON'))
(('CTNNB1', 'ON'), ('FAK', 'ON'))
(('CTNNB1', 'ON'), ('AKT1', 'OFF'))
(('CTNNB1', 'ON'), ('DKK1', 'OFF'))
(('CTNNB1', 'ON'), ('AKT2', 'OFF'))
(('CTNNB1', 'ON'), ('RAC1', 'OFF'))
(('CTNNB1', 'ON'), ('YAP1', 'OFF'))
(('CTNNB1', 'ON'), ('PIK3CA', 'OFF'))
(('CTNNB1', 'ON'), ('ERK', 'OFF'))
(('CTNNB1', 'ON'), ('p21', 'OFF'))
(('CTNNB1', 'ON'), ('SMAD', 'OFF'))
(('CTNNB1', 'ON'), ('VIM', 'OFF'))
(('CTNNB1', 'ON'), ('p53', 'OFF'))
(('CTNNB1', 'ON'), ('p63', 'OFF'))
(('CTNNB1', 'ON'), ('p73', 'OFF'))
(('CTNNB1', 'ON'), ('miR203', 'OFF'))
(('CTNNB1', 'ON'), ('miR34', 'OFF'))
(('CTNNB1', 'ON'), ('miR200', 'OFF'))
(('CTNNB1', 'ON'), ('TGFbetaR', 'OFF'))
(('CTNNB1', 'ON'), ('FAK', 'OFF'))
(('DKK1', 'ON'), ('AKT2', 'ON'))
(('DKK1', 'ON'), ('RAC1', 'ON'))
(('DKK1', 'ON'), ('YAP1', 'ON'))
(('DKK1', 'ON'), ('PIK3CA', 'ON'))
(('DKK1', 'ON'), ('ERK', 'ON'))
(('DKK1', 'ON'), ('p21', 'ON'))
(('DKK1', 'ON'), ('SMAD', 'ON'))
(('DKK1', 'ON'), ('VIM', 'ON'))
(('DKK1', 'ON'), ('p53', 'ON'))
(('DKK1', 'ON'), ('p63', 'ON'))
(('DKK1', 'ON'), ('p73', 'ON'))
(('DKK1', 'ON'), ('miR203', 'ON'))
(('DKK1', 'ON'), ('miR34', 'ON'))
(('DKK1', 'ON'), ('miR200', 'ON'))
(('DKK1', 'ON'), ('TGFbetaR', 'ON'))
(('DKK1', 'ON'), ('FAK', 'ON'))
(('DKK1', 'ON'), ('AKT1', 'OFF'))
(('DKK1', 'ON'), ('CTNNB1', 'OFF'))
/Users/laurence/opt/miniconda3/envs/colosys/lib/python3.7/site-packages/maboss/figures.py:87: MatplotlibDeprecatio
nWarning: normalize=None does not normalize if the sum is less than 1 but this behavior is deprecated since 3.3 un
til two minor releases later. After the deprecation period the default value will be normalize=True. To prevent no
rmalization pass normalize=False
  startangle=90, colors=color_list, **opts)
```

```
(('DKK1', 'ON'), ('AKT2', 'OFF'))
(('DKK1', 'ON'), ('RAC1', 'OFF'))
(('DKK1', 'ON'), ('YAP1', 'OFF'))
(('DKK1', 'ON'), ('PIK3CA', 'OFF'))
(('DKK1', 'ON'), ('ERK', 'OFF'))
(('DKK1', 'ON'), ('p21', 'OFF'))
(('DKK1', 'ON'), ('SMAD', 'OFF'))
(('DKK1', 'ON'), ('VIM', 'OFF'))
(('DKK1', 'ON'), ('p53', 'OFF'))
(('DKK1', 'ON'), ('p63', 'OFF'))
(('DKK1', 'ON'), ('p73', 'OFF'))
(('DKK1', 'ON'), ('miR203', 'OFF'))
(('DKK1', 'ON'), ('miR34', 'OFF'))
(('DKK1', 'ON'), ('miR200', 'OFF'))
(('DKK1', 'ON'), ('TGFbetaR', 'OFF'))
(('DKK1', 'ON'), ('FAK', 'OFF'))
(('AKT2', 'ON'), ('RAC1', 'ON'))
(('AKT2', 'ON'), ('YAP1', 'ON'))
(('AKT2', 'ON'), ('PIK3CA', 'ON'))
(('AKT2', 'ON'), ('ERK', 'ON'))
(('AKT2', 'ON'), ('p21', 'ON'))
(('AKT2', 'ON'), ('SMAD', 'ON'))
(('AKT2', 'ON'), ('VIM', 'ON'))
(('AKT2', 'ON'), ('p53', 'ON'))
(('AKT2', 'ON'), ('p63', 'ON'))
(('AKT2', 'ON'), ('p73', 'ON'))
(('AKT2', 'ON'), ('miR203', 'ON'))
(('AKT2', 'ON'), ('miR34', 'ON'))
(('AKT2', 'ON'), ('miR200', 'ON'))
(('AKT2', 'ON'), ('TGFbetaR', 'ON'))
(('AKT2', 'ON'), ('FAK', 'ON'))
(('AKT2', 'ON'), ('AKT1', 'OFF'))
(('AKT2', 'ON'), ('CTNNB1', 'OFF'))
(('AKT2', 'ON'), ('DKK1', 'OFF'))
(('AKT2', 'ON'), ('RAC1', 'OFF'))
(('AKT2', 'ON'), ('YAP1', 'OFF'))
(('AKT2', 'ON'), ('PIK3CA', 'OFF'))
(('AKT2', 'ON'), ('ERK', 'OFF'))
(('AKT2', 'ON'), ('p21', 'OFF'))
(('AKT2', 'ON'), ('SMAD', 'OFF'))
(('AKT2', 'ON'), ('VIM', 'OFF'))
(('AKT2', 'ON'), ('p53', 'OFF'))
(('AKT2', 'ON'), ('p63', 'OFF'))
(('AKT2', 'ON'), ('p73', 'OFF'))
(('AKT2', 'ON'), ('miR203', 'OFF'))
(('AKT2', 'ON'), ('miR34', 'OFF'))
(('AKT2', 'ON'), ('miR200', 'OFF'))
(('AKT2', 'ON'), ('TGFbetaR', 'OFF'))
(('AKT2', 'ON'), ('FAK', 'OFF'))
(('RAC1', 'ON'), ('YAP1', 'ON'))
(('RAC1', 'ON'), ('PIK3CA', 'ON'))
(('RAC1', 'ON'), ('ERK', 'ON'))
(('RAC1', 'ON'), ('p21', 'ON'))
(('RAC1', 'ON'), ('SMAD', 'ON'))
(('RAC1', 'ON'), ('VIM', 'ON'))
(('RAC1', 'ON'), ('p53', 'ON'))
(('RAC1', 'ON'), ('p63', 'ON'))
(('RAC1', 'ON'), ('p73', 'ON'))
(('RAC1', 'ON'), ('miR203', 'ON'))
(('RAC1', 'ON'), ('miR34', 'ON'))
(('RAC1', 'ON'), ('miR200', 'ON'))
(('RAC1', 'ON'), ('TGFbetaR', 'ON'))
(('RAC1', 'ON'), ('FAK', 'ON'))
(('RAC1', 'ON'), ('AKT1', 'OFF'))
(('RAC1', 'ON'), ('CTNNB1', 'OFF'))
(('RAC1', 'ON'), ('DKK1', 'OFF'))
(('RAC1', 'ON'), ('AKT2', 'OFF'))
(('RAC1', 'ON'), ('YAP1', 'OFF'))
(('RAC1', 'ON'), ('PIK3CA', 'OFF'))
(('RAC1', 'ON'), ('ERK', 'OFF'))
(('RAC1', 'ON'), ('p21', 'OFF'))
(('RAC1', 'ON'), ('SMAD', 'OFF'))
(('RAC1', 'ON'), ('VIM', 'OFF'))
(('RAC1', 'ON'), ('p53', 'OFF'))
(('RAC1', 'ON'), ('p63', 'OFF'))
(('RAC1', 'ON'), ('p73', 'OFF'))
(('RAC1', 'ON'), ('miR203', 'OFF'))
(('RAC1', 'ON'), ('miR34', 'OFF'))
(('RAC1', 'ON'), ('miR200', 'OFF'))
(('RAC1', 'ON'), ('TGFbetaR', 'OFF'))
(('RAC1', 'ON'), ('FAK', 'OFF'))
(('YAP1', 'ON'), ('PIK3CA', 'ON'))
(('YAP1', 'ON'), ('ERK', 'ON'))
(('YAP1', 'ON'), ('p21', 'ON'))
(('YAP1', 'ON'), ('SMAD', 'ON'))
(('YAP1', 'ON'), ('VIM', 'ON'))
(('YAP1', 'ON'), ('p53', 'ON'))
(('YAP1', 'ON'), ('p63', 'ON'))
(('YAP1', 'ON'), ('p73', 'ON'))
(('YAP1', 'ON'), ('miR203', 'ON'))
(('YAP1', 'ON'), ('miR34', 'ON'))
```

```
(('YAP1', 'ON'), ('miR200', 'ON'))
(('YAP1', 'ON'), ('TGFbetaR', 'ON'))
(('YAP1', 'ON'), ('FAK', 'ON'))
(('YAP1', 'ON'), ('AKT1', 'OFF'))
(('YAP1', 'ON'), ('CTNNB1', 'OFF'))
(('YAP1', 'ON'), ('DKK1', 'OFF'))
(('YAP1', 'ON'), ('AKT2', 'OFF'))
(('YAP1', 'ON'), ('RAC1', 'OFF'))
(('YAP1', 'ON'), ('PIK3CA', 'OFF'))
(('YAP1', 'ON'), ('ERK', 'OFF'))
(('YAP1', 'ON'), ('p21', 'OFF'))
(('YAP1', 'ON'), ('SMAD', 'OFF'))
(('YAP1', 'ON'), ('VIM', 'OFF'))
(('YAP1', 'ON'), ('p53', 'OFF'))
(('YAP1', 'ON'), ('p63', 'OFF'))
(('YAP1', 'ON'), ('p73', 'OFF'))
(('YAP1', 'ON'), ('miR203', 'OFF'))
(('YAP1', 'ON'), ('miR34', 'OFF'))
(('YAP1', 'ON'), ('miR200', 'OFF'))
(('YAP1', 'ON'), ('TGFbetaR', 'OFF'))
(('YAP1', 'ON'), ('FAK', 'OFF'))
(('PIK3CA', 'ON'), ('ERK', 'ON'))
(('PIK3CA', 'ON'), ('p21', 'ON'))
(('PIK3CA', 'ON'), ('SMAD', 'ON'))
(('PIK3CA', 'ON'), ('VIM', 'ON'))
(('PIK3CA', 'ON'), ('p53', 'ON'))
(('PIK3CA', 'ON'), ('p63', 'ON'))
(('PIK3CA', 'ON'), ('p73', 'ON'))
(('PIK3CA', 'ON'), ('miR203', 'ON'))
(('PIK3CA', 'ON'), ('miR34', 'ON'))
(('PIK3CA', 'ON'), ('miR200', 'ON'))
(('PIK3CA', 'ON'), ('TGFbetaR', 'ON'))
(('PIK3CA', 'ON'), ('FAK', 'ON'))
(('PIK3CA', 'ON'), ('AKT1', 'OFF'))
(('PIK3CA', 'ON'), ('CTNNB1', 'OFF'))
(('PIK3CA', 'ON'), ('DKK1', 'OFF'))
(('PIK3CA', 'ON'), ('AKT2', 'OFF'))
(('PIK3CA', 'ON'), ('RAC1', 'OFF'))
(('PIK3CA', 'ON'), ('YAP1', 'OFF'))
(('PIK3CA', 'ON'), ('ERK', 'OFF'))
(('PIK3CA', 'ON'), ('p21', 'OFF'))
(('PIK3CA', 'ON'), ('SMAD', 'OFF'))
(('PIK3CA', 'ON'), ('VIM', 'OFF'))
(('PIK3CA', 'ON'), ('p53', 'OFF'))
(('PIK3CA', 'ON'), ('p63', 'OFF'))
(('PIK3CA', 'ON'), ('p73', 'OFF'))
(('PIK3CA', 'ON'), ('miR203', 'OFF'))
(('PIK3CA', 'ON'), ('miR34', 'OFF'))
(('PIK3CA', 'ON'), ('miR200', 'OFF'))
(('PIK3CA', 'ON'), ('TGFbetaR', 'OFF'))
(('PIK3CA', 'ON'), ('FAK', 'OFF'))
(('ERK', 'ON'), ('p21', 'ON'))
(('ERK', 'ON'), ('SMAD', 'ON'))
(('ERK', 'ON'), ('VIM', 'ON'))
(('ERK', 'ON'), ('p53', 'ON'))
(('ERK', 'ON'), ('p63', 'ON'))
(('ERK', 'ON'), ('p73', 'ON'))
(('ERK', 'ON'), ('miR203', 'ON'))
(('ERK', 'ON'), ('miR34', 'ON'))
(('ERK', 'ON'), ('miR200', 'ON'))
(('ERK', 'ON'), ('TGFbetaR', 'ON'))
(('ERK', 'ON'), ('FAK', 'ON'))
(('ERK', 'ON'), ('AKT1', 'OFF'))
(('ERK', 'ON'), ('CTNNB1', 'OFF'))
(('ERK', 'ON'), ('DKK1', 'OFF'))
(('ERK', 'ON'), ('AKT2', 'OFF'))
(('ERK', 'ON'), ('RAC1', 'OFF'))
(('ERK', 'ON'), ('YAP1', 'OFF'))
(('ERK', 'ON'), ('PIK3CA', 'OFF'))
(('ERK', 'ON'), ('p21', 'OFF'))
(('ERK', 'ON'), ('SMAD', 'OFF'))
(('ERK', 'ON'), ('VIM', 'OFF'))
(('ERK', 'ON'), ('p53', 'OFF'))
(('ERK', 'ON'), ('p63', 'OFF'))
(('ERK', 'ON'), ('p73', 'OFF'))
(('ERK', 'ON'), ('miR203', 'OFF'))
(('ERK', 'ON'), ('miR34', 'OFF'))
(('ERK', 'ON'), ('miR200', 'OFF'))
(('ERK', 'ON'), ('TGFbetaR', 'OFF'))
(('ERK', 'ON'), ('FAK', 'OFF'))
(('p21', 'ON'), ('SMAD', 'ON'))
(('p21', 'ON'), ('VIM', 'ON'))
(('p21', 'ON'), ('p53', 'ON'))
(('p21', 'ON'), ('p63', 'ON'))
(('p21', 'ON'), ('p73', 'ON'))
(('p21', 'ON'), ('miR203', 'ON'))
(('p21', 'ON'), ('miR34', 'ON'))
(('p21', 'ON'), ('miR200', 'ON'))
(('p21', 'ON'), ('TGFbetaR', 'ON'))
(('p21', 'ON'), ('FAK', 'ON'))
(('p21', 'ON'), ('AKT1', 'OFF'))
```

```
(('p21', 'ON'), ('CTNNB1', 'OFF'))
(('p21', 'ON'), ('DKK1', 'OFF'))
(('p21', 'ON'), ('AKT2', 'OFF'))
(('p21', 'ON'), ('RAC1', 'OFF'))
(('p21', 'ON'), ('YAP1', 'OFF'))
(('p21', 'ON'), ('PIK3CA', 'OFF'))
(('p21', 'ON'), ('ERK', 'OFF'))
(('p21', 'ON'), ('SMAD', 'OFF'))
(('p21', 'ON'), ('VIM', 'OFF'))
(('p21', 'ON'), ('p53', 'OFF'))
(('p21', 'ON'), ('p63', 'OFF'))
(('p21', 'ON'), ('p73', 'OFF'))
(('p21', 'ON'), ('miR203', 'OFF'))
(('p21', 'ON'), ('miR34', 'OFF'))
(('p21', 'ON'), ('miR200', 'OFF'))
(('p21', 'ON'), ('TGFbetaR', 'OFF'))
(('p21', 'ON'), ('FAK', 'OFF'))
(('SMAD', 'ON'), ('VIM', 'ON'))
(('SMAD', 'ON'), ('p53', 'ON'))
(('SMAD', 'ON'), ('p63', 'ON'))
(('SMAD', 'ON'), ('p73', 'ON'))
(('SMAD', 'ON'), ('miR203', 'ON'))
(('SMAD', 'ON'), ('miR34', 'ON'))
(('SMAD', 'ON'), ('miR200', 'ON'))
(('SMAD', 'ON'), ('TGFbetaR', 'ON'))
(('SMAD', 'ON'), ('FAK', 'ON'))
(('SMAD', 'ON'), ('AKT1', 'OFF'))
(('SMAD', 'ON'), ('CTNNB1', 'OFF'))
(('SMAD', 'ON'), ('DKK1', 'OFF'))
(('SMAD', 'ON'), ('AKT2', 'OFF'))
(('SMAD', 'ON'), ('RAC1', 'OFF'))
(('SMAD', 'ON'), ('YAP1', 'OFF'))
(('SMAD', 'ON'), ('PIK3CA', 'OFF'))
(('SMAD', 'ON'), ('ERK', 'OFF'))
(('SMAD', 'ON'), ('p21', 'OFF'))
(('SMAD', 'ON'), ('VIM', 'OFF'))
(('SMAD', 'ON'), ('p53', 'OFF'))
(('SMAD', 'ON'), ('p63', 'OFF'))
(('SMAD', 'ON'), ('p73', 'OFF'))
(('SMAD', 'ON'), ('miR203', 'OFF'))
(('SMAD', 'ON'), ('miR34', 'OFF'))
(('SMAD', 'ON'), ('miR200', 'OFF'))
(('SMAD', 'ON'), ('TGFbetaR', 'OFF'))
(('SMAD', 'ON'), ('FAK', 'OFF'))
(('VIM', 'ON'), ('p53', 'ON'))
(('VIM', 'ON'), ('p63', 'ON'))
(('VIM', 'ON'), ('p73', 'ON'))
(('VIM', 'ON'), ('miR203', 'ON'))
(('VIM', 'ON'), ('miR34', 'ON'))
(('VIM', 'ON'), ('miR200', 'ON'))
(('VIM', 'ON'), ('TGFbetaR', 'ON'))
(('VIM', 'ON'), ('FAK', 'ON'))
(('VIM', 'ON'), ('AKT1', 'OFF'))
(('VIM', 'ON'), ('CTNNB1', 'OFF'))
(('VIM', 'ON'), ('DKK1', 'OFF'))
(('VIM', 'ON'), ('AKT2', 'OFF'))
(('VIM', 'ON'), ('RAC1', 'OFF'))
(('VIM', 'ON'), ('YAP1', 'OFF'))
(('VIM', 'ON'), ('PIK3CA', 'OFF'))
(('VIM', 'ON'), ('ERK', 'OFF'))
(('VIM', 'ON'), ('p21', 'OFF'))
(('VIM', 'ON'), ('SMAD', 'OFF'))
(('VIM', 'ON'), ('p53', 'OFF'))
(('VIM', 'ON'), ('p63', 'OFF'))
(('VIM', 'ON'), ('p73', 'OFF'))
(('VIM', 'ON'), ('miR203', 'OFF'))
(('VIM', 'ON'), ('miR34', 'OFF'))
(('VIM', 'ON'), ('miR200', 'OFF'))
(('VIM', 'ON'), ('TGFbetaR', 'OFF'))
(('VIM', 'ON'), ('FAK', 'OFF'))
(('p53', 'ON'), ('p63', 'ON'))
(('p53', 'ON'), ('p73', 'ON'))
(('p53', 'ON'), ('miR203', 'ON'))
(('p53', 'ON'), ('miR34', 'ON'))
(('p53', 'ON'), ('miR200', 'ON'))
(('p53', 'ON'), ('TGFbetaR', 'ON'))
(('p53', 'ON'), ('FAK', 'ON'))
(('p53', 'ON'), ('AKT1', 'OFF'))
(('p53', 'ON'), ('CTNNB1', 'OFF'))
(('p53', 'ON'), ('DKK1', 'OFF'))
(('p53', 'ON'), ('AKT2', 'OFF'))
(('p53', 'ON'), ('RAC1', 'OFF'))
(('p53', 'ON'), ('YAP1', 'OFF'))
(('p53', 'ON'), ('PIK3CA', 'OFF'))
(('p53', 'ON'), ('ERK', 'OFF'))
(('p53', 'ON'), ('p21', 'OFF'))
(('p53', 'ON'), ('SMAD', 'OFF'))
(('p53', 'ON'), ('VIM', 'OFF'))
(('p53', 'ON'), ('p63', 'OFF'))
(('p53', 'ON'), ('p73', 'OFF'))
(('p53', 'ON'), ('miR203', 'OFF'))
```

```
(('p53', 'ON'), ('miR34', 'OFF'))
(('p53', 'ON'), ('miR200', 'OFF'))
(('p53', 'ON'), ('TGFbetaR', 'OFF'))
(('p53', 'ON'), ('FAK', 'OFF'))
(('p63', 'ON'), ('p73', 'ON'))
(('p63', 'ON'), ('miR203', 'ON'))
(('p63', 'ON'), ('miR34', 'ON'))
(('p63', 'ON'), ('miR200', 'ON'))
(('p63', 'ON'), ('TGFbetaR', 'ON'))
(('p63', 'ON'), ('FAK', 'ON'))
(('p63', 'ON'), ('AKT1', 'OFF'))
(('p63', 'ON'), ('CTNNB1', 'OFF'))
(('p63', 'ON'), ('DKK1', 'OFF'))
(('p63', 'ON'), ('AKT2', 'OFF'))
(('p63', 'ON'), ('RAC1', 'OFF'))
(('p63', 'ON'), ('YAP1', 'OFF'))
(('p63', 'ON'), ('PIK3CA', 'OFF'))
(('p63', 'ON'), ('ERK', 'OFF'))
(('p63', 'ON'), ('p21', 'OFF'))
(('p63', 'ON'), ('SMAD', 'OFF'))
(('p63', 'ON'), ('VIM', 'OFF'))
(('p63', 'ON'), ('p53', 'OFF'))
(('p63', 'ON'), ('p73', 'OFF'))
(('p63', 'ON'), ('miR203', 'OFF'))
(('p63', 'ON'), ('miR34', 'OFF'))
(('p63', 'ON'), ('miR200', 'OFF'))
(('p63', 'ON'), ('TGFbetaR', 'OFF'))
(('p63', 'ON'), ('FAK', 'OFF'))
(('p73', 'ON'), ('miR203', 'ON'))
(('p73', 'ON'), ('miR34', 'ON'))
(('p73', 'ON'), ('miR200', 'ON'))
(('p73', 'ON'), ('TGFbetaR', 'ON'))
(('p73', 'ON'), ('FAK', 'ON'))
(('p73', 'ON'), ('AKT1', 'OFF'))
(('p73', 'ON'), ('CTNNB1', 'OFF'))
(('p73', 'ON'), ('DKK1', 'OFF'))
(('p73', 'ON'), ('AKT2', 'OFF'))
(('p73', 'ON'), ('RAC1', 'OFF'))
(('p73', 'ON'), ('YAP1', 'OFF'))
(('p73', 'ON'), ('PIK3CA', 'OFF'))
(('p73', 'ON'), ('ERK', 'OFF'))
(('p73', 'ON'), ('p21', 'OFF'))
(('p73', 'ON'), ('SMAD', 'OFF'))
(('p73', 'ON'), ('VIM', 'OFF'))
(('p73', 'ON'), ('p53', 'OFF'))
(('p73', 'ON'), ('p63', 'OFF'))
(('p73', 'ON'), ('miR203', 'OFF'))
(('p73', 'ON'), ('miR34', 'OFF'))
(('p73', 'ON'), ('miR200', 'OFF'))
(('p73', 'ON'), ('TGFbetaR', 'OFF'))
(('p73', 'ON'), ('FAK', 'OFF'))
(('miR203', 'ON'), ('miR34', 'ON'))
(('miR203', 'ON'), ('miR200', 'ON'))
(('miR203', 'ON'), ('TGFbetaR', 'ON'))
(('miR203', 'ON'), ('FAK', 'ON'))
(('miR203', 'ON'), ('AKT1', 'OFF'))
(('miR203', 'ON'), ('CTNNB1', 'OFF'))
(('miR203', 'ON'), ('DKK1', 'OFF'))
(('miR203', 'ON'), ('AKT2', 'OFF'))
(('miR203', 'ON'), ('RAC1', 'OFF'))
(('miR203', 'ON'), ('YAP1', 'OFF'))
(('miR203', 'ON'), ('PIK3CA', 'OFF'))
(('miR203', 'ON'), ('ERK', 'OFF'))
(('miR203', 'ON'), ('p21', 'OFF'))
(('miR203', 'ON'), ('SMAD', 'OFF'))
(('miR203', 'ON'), ('VIM', 'OFF'))
(('miR203', 'ON'), ('p53', 'OFF'))
(('miR203', 'ON'), ('p63', 'OFF'))
(('miR203', 'ON'), ('p73', 'OFF'))
(('miR203', 'ON'), ('miR34', 'OFF'))
(('miR203', 'ON'), ('miR200', 'OFF'))
(('miR203', 'ON'), ('TGFbetaR', 'OFF'))
(('miR203', 'ON'), ('FAK', 'OFF'))
(('miR34', 'ON'), ('miR200', 'ON'))
(('miR34', 'ON'), ('TGFbetaR', 'ON'))
(('miR34', 'ON'), ('FAK', 'ON'))
(('miR34', 'ON'), ('AKT1', 'OFF'))
(('miR34', 'ON'), ('CTNNB1', 'OFF'))
(('miR34', 'ON'), ('DKK1', 'OFF'))
(('miR34', 'ON'), ('AKT2', 'OFF'))
(('miR34', 'ON'), ('RAC1', 'OFF'))
(('miR34', 'ON'), ('YAP1', 'OFF'))
(('miR34', 'ON'), ('PIK3CA', 'OFF'))
(('miR34', 'ON'), ('ERK', 'OFF'))
(('miR34', 'ON'), ('p21', 'OFF'))
(('miR34', 'ON'), ('SMAD', 'OFF'))
(('miR34', 'ON'), ('VIM', 'OFF'))
(('miR34', 'ON'), ('p53', 'OFF'))
(('miR34', 'ON'), ('p63', 'OFF'))
(('miR34', 'ON'), ('p73', 'OFF'))
(('miR34', 'ON'), ('miR203', 'OFF'))
```

```
(('miR34', 'ON'), ('miR200', 'OFF'))
(('miR34', 'ON'), ('TGFbetaR', 'OFF'))
(('miR34', 'ON'), ('FAK', 'OFF'))
(('miR200', 'ON'), ('TGFbetaR', 'ON'))
(('miR200', 'ON'), ('FAK', 'ON'))
(('miR200', 'ON'), ('AKT1', 'OFF'))
(('miR200', 'ON'), ('CTNNB1', 'OFF'))
(('miR200', 'ON'), ('DKK1', 'OFF'))
(('miR200', 'ON'), ('AKT2', 'OFF'))
(('miR200', 'ON'), ('RAC1', 'OFF'))
(('miR200', 'ON'), ('YAP1', 'OFF'))
(('miR200', 'ON'), ('PIK3CA', 'OFF'))
(('miR200', 'ON'), ('ERK', 'OFF'))
(('miR200', 'ON'), ('p21', 'OFF'))
(('miR200', 'ON'), ('SMAD', 'OFF'))
(('miR200', 'ON'), ('VIM', 'OFF'))
(('miR200', 'ON'), ('p53', 'OFF'))
(('miR200', 'ON'), ('p63', 'OFF'))
(('miR200', 'ON'), ('p73', 'OFF'))
(('miR200', 'ON'), ('miR203', 'OFF'))
(('miR200', 'ON'), ('miR34', 'OFF'))
(('miR200', 'ON'), ('TGFbetaR', 'OFF'))
(('miR200', 'ON'), ('FAK', 'OFF'))
(('TGFbetaR', 'ON'), ('FAK', 'ON'))
(('TGFbetaR', 'ON'), ('AKT1', 'OFF'))
(('TGFbetaR', 'ON'), ('CTNNB1', 'OFF'))
(('TGFbetaR', 'ON'), ('DKK1', 'OFF'))
(('TGFbetaR', 'ON'), ('AKT2', 'OFF'))
(('TGFbetaR', 'ON'), ('RAC1', 'OFF'))
(('TGFbetaR', 'ON'), ('YAP1', 'OFF'))
(('TGFbetaR', 'ON'), ('PIK3CA', 'OFF'))
(('TGFbetaR', 'ON'), ('ERK', 'OFF'))
(('TGFbetaR', 'ON'), ('p21', 'OFF'))
(('TGFbetaR', 'ON'), ('SMAD', 'OFF'))
(('TGFbetaR', 'ON'), ('VIM', 'OFF'))
(('TGFbetaR', 'ON'), ('p53', 'OFF'))
(('TGFbetaR', 'ON'), ('p63', 'OFF'))
(('TGFbetaR', 'ON'), ('p73', 'OFF'))
(('TGFbetaR', 'ON'), ('miR203', 'OFF'))
(('TGFbetaR', 'ON'), ('miR34', 'OFF'))
(('TGFbetaR', 'ON'), ('miR200', 'OFF'))
(('TGFbetaR', 'ON'), ('FAK', 'OFF'))
(('FAK', 'ON'), ('AKT1', 'OFF'))
(('FAK', 'ON'), ('CTNNB1', 'OFF'))
(('FAK', 'ON'), ('DKK1', 'OFF'))
(('FAK', 'ON'), ('AKT2', 'OFF'))
(('FAK', 'ON'), ('RAC1', 'OFF'))
(('FAK', 'ON'), ('YAP1', 'OFF'))
(('FAK', 'ON'), ('PIK3CA', 'OFF'))
(('FAK', 'ON'), ('ERK', 'OFF'))
(('FAK', 'ON'), ('p21', 'OFF'))
(('FAK', 'ON'), ('SMAD', 'OFF'))
(('FAK', 'ON'), ('VIM', 'OFF'))
(('FAK', 'ON'), ('p53', 'OFF'))
(('FAK', 'ON'), ('p63', 'OFF'))
(('FAK', 'ON'), ('p73', 'OFF'))
(('FAK', 'ON'), ('miR203', 'OFF'))
(('FAK', 'ON'), ('miR34', 'OFF'))
(('FAK', 'ON'), ('miR200', 'OFF'))
(('FAK', 'ON'), ('TGFbetaR', 'OFF'))
(('AKT1', 'OFF'), ('CTNNB1', 'OFF'))
(('AKT1', 'OFF'), ('DKK1', 'OFF'))
(('AKT1', 'OFF'), ('AKT2', 'OFF'))
(('AKT1', 'OFF'), ('RAC1', 'OFF'))
(('AKT1', 'OFF'), ('YAP1', 'OFF'))
(('AKT1', 'OFF'), ('PIK3CA', 'OFF'))
(('AKT1', 'OFF'), ('ERK', 'OFF'))
(('AKT1', 'OFF'), ('p21', 'OFF'))
(('AKT1', 'OFF'), ('SMAD', 'OFF'))
(('AKT1', 'OFF'), ('VIM', 'OFF'))
(('AKT1', 'OFF'), ('p53', 'OFF'))
(('AKT1', 'OFF'), ('p63', 'OFF'))
(('AKT1', 'OFF'), ('p73', 'OFF'))
(('AKT1', 'OFF'), ('miR203', 'OFF'))
(('AKT1', 'OFF'), ('miR34', 'OFF'))
(('AKT1', 'OFF'), ('miR200', 'OFF'))
(('AKT1', 'OFF'), ('TGFbetaR', 'OFF'))
(('AKT1', 'OFF'), ('FAK', 'OFF'))
(('CTNNB1', 'OFF'), ('DKK1', 'OFF'))
(('CTNNB1', 'OFF'), ('AKT2', 'OFF'))
(('CTNNB1', 'OFF'), ('RAC1', 'OFF'))
(('CTNNB1', 'OFF'), ('YAP1', 'OFF'))
(('CTNNB1', 'OFF'), ('PIK3CA', 'OFF'))
(('CTNNB1', 'OFF'), ('ERK', 'OFF'))
(('CTNNB1', 'OFF'), ('p21', 'OFF'))
(('CTNNB1', 'OFF'), ('SMAD', 'OFF'))
(('CTNNB1', 'OFF'), ('VIM', 'OFF'))
(('CTNNB1', 'OFF'), ('p53', 'OFF'))
(('CTNNB1', 'OFF'), ('p63', 'OFF'))
(('CTNNB1', 'OFF'), ('p73', 'OFF'))
(('CTNNB1', 'OFF'), ('miR203', 'OFF'))
```

```
(('CTNNB1', 'OFF'), ('miR34', 'OFF'))
(('CTNNB1', 'OFF'), ('miR200', 'OFF'))
(('CTNNB1', 'OFF'), ('TGFbetaR', 'OFF'))
(('CTNNB1', 'OFF'), ('FAK', 'OFF'))
(('DKK1', 'OFF'), ('AKT2', 'OFF'))
(('DKK1', 'OFF'), ('RAC1', 'OFF'))
(('DKK1', 'OFF'), ('YAP1', 'OFF'))
(('DKK1', 'OFF'), ('PIK3CA', 'OFF'))
(('DKK1', 'OFF'), ('ERK', 'OFF'))
(('DKK1', 'OFF'), ('p21', 'OFF'))
(('DKK1', 'OFF'), ('SMAD', 'OFF'))
(('DKK1', 'OFF'), ('VIM', 'OFF'))
(('DKK1', 'OFF'), ('p53', 'OFF'))
(('DKK1', 'OFF'), ('p63', 'OFF'))
(('DKK1', 'OFF'), ('p73', 'OFF'))
(('DKK1', 'OFF'), ('miR203', 'OFF'))
(('DKK1', 'OFF'), ('miR34', 'OFF'))
(('DKK1', 'OFF'), ('miR200', 'OFF'))
(('DKK1', 'OFF'), ('TGFbetaR', 'OFF'))
(('DKK1', 'OFF'), ('FAK', 'OFF'))
(('AKT2', 'OFF'), ('RAC1', 'OFF'))
(('AKT2', 'OFF'), ('YAP1', 'OFF'))
(('AKT2', 'OFF'), ('PIK3CA', 'OFF'))
(('AKT2', 'OFF'), ('ERK', 'OFF'))
(('AKT2', 'OFF'), ('p21', 'OFF'))
(('AKT2', 'OFF'), ('SMAD', 'OFF'))
(('AKT2', 'OFF'), ('VIM', 'OFF'))
(('AKT2', 'OFF'), ('p53', 'OFF'))
(('AKT2', 'OFF'), ('p63', 'OFF'))
(('AKT2', 'OFF'), ('p73', 'OFF'))
(('AKT2', 'OFF'), ('miR203', 'OFF'))
(('AKT2', 'OFF'), ('miR34', 'OFF'))
(('AKT2', 'OFF'), ('miR200', 'OFF'))
(('AKT2', 'OFF'), ('TGFbetaR', 'OFF'))
(('AKT2', 'OFF'), ('FAK', 'OFF'))
(('RAC1', 'OFF'), ('YAP1', 'OFF'))
(('RAC1', 'OFF'), ('PIK3CA', 'OFF'))
(('RAC1', 'OFF'), ('ERK', 'OFF'))
(('RAC1', 'OFF'), ('p21', 'OFF'))
(('RAC1', 'OFF'), ('SMAD', 'OFF'))
(('RAC1', 'OFF'), ('VIM', 'OFF'))
(('RAC1', 'OFF'), ('p53', 'OFF'))
(('RAC1', 'OFF'), ('p63', 'OFF'))
(('RAC1', 'OFF'), ('p73', 'OFF'))
(('RAC1', 'OFF'), ('miR203', 'OFF'))
(('RAC1', 'OFF'), ('miR34', 'OFF'))
(('RAC1', 'OFF'), ('miR200', 'OFF'))
(('RAC1', 'OFF'), ('TGFbetaR', 'OFF'))
(('RAC1', 'OFF'), ('FAK', 'OFF'))
(('YAP1', 'OFF'), ('PIK3CA', 'OFF'))
(('YAP1', 'OFF'), ('ERK', 'OFF'))
(('YAP1', 'OFF'), ('p21', 'OFF'))
(('YAP1', 'OFF'), ('SMAD', 'OFF'))
(('YAP1', 'OFF'), ('VIM', 'OFF'))
(('YAP1', 'OFF'), ('p53', 'OFF'))
(('YAP1', 'OFF'), ('p63', 'OFF'))
(('YAP1', 'OFF'), ('p73', 'OFF'))
(('YAP1', 'OFF'), ('miR203', 'OFF'))
(('YAP1', 'OFF'), ('miR34', 'OFF'))
(('YAP1', 'OFF'), ('miR200', 'OFF'))
(('YAP1', 'OFF'), ('TGFbetaR', 'OFF'))
(('YAP1', 'OFF'), ('FAK', 'OFF'))
(('PIK3CA', 'OFF'), ('ERK', 'OFF'))
(('PIK3CA', 'OFF'), ('p21', 'OFF'))
(('PIK3CA', 'OFF'), ('SMAD', 'OFF'))
(('PIK3CA', 'OFF'), ('VIM', 'OFF'))
(('PIK3CA', 'OFF'), ('p53', 'OFF'))
(('PIK3CA', 'OFF'), ('p63', 'OFF'))
(('PIK3CA', 'OFF'), ('p73', 'OFF'))
(('PIK3CA', 'OFF'), ('miR203', 'OFF'))
(('PIK3CA', 'OFF'), ('miR34', 'OFF'))
(('PIK3CA', 'OFF'), ('miR200', 'OFF'))
(('PIK3CA', 'OFF'), ('TGFbetaR', 'OFF'))
(('PIK3CA', 'OFF'), ('FAK', 'OFF'))
(('ERK', 'OFF'), ('p21', 'OFF'))
(('ERK', 'OFF'), ('SMAD', 'OFF'))
(('ERK', 'OFF'), ('VIM', 'OFF'))
(('ERK', 'OFF'), ('p53', 'OFF'))
(('ERK', 'OFF'), ('p63', 'OFF'))
(('ERK', 'OFF'), ('p73', 'OFF'))
(('ERK', 'OFF'), ('miR203', 'OFF'))
(('ERK', 'OFF'), ('miR34', 'OFF'))
(('ERK', 'OFF'), ('miR200', 'OFF'))
(('ERK', 'OFF'), ('TGFbetaR', 'OFF'))
(('ERK', 'OFF'), ('FAK', 'OFF'))
(('p21', 'OFF'), ('SMAD', 'OFF'))
(('p21', 'OFF'), ('VIM', 'OFF'))
(('p21', 'OFF'), ('p53', 'OFF'))
(('p21', 'OFF'), ('p63', 'OFF'))
(('p21', 'OFF'), ('p73', 'OFF'))
(('p21', 'OFF'), ('miR203', 'OFF'))
```

```
(('p21', 'OFF'), ('miR34', 'OFF'))
(('p21', 'OFF'), ('miR200', 'OFF'))
(('p21', 'OFF'), ('TGFbetaR', 'OFF'))
(('p21', 'OFF'), ('FAK', 'OFF'))
(('SMAD', 'OFF'), ('VIM', 'OFF'))
(('SMAD', 'OFF'), ('p53', 'OFF'))
(('SMAD', 'OFF'), ('p63', 'OFF'))
(('SMAD', 'OFF'), ('p73', 'OFF'))
(('SMAD', 'OFF'), ('miR203', 'OFF'))
(('SMAD', 'OFF'), ('miR34', 'OFF'))
(('SMAD', 'OFF'), ('miR200', 'OFF'))
(('SMAD', 'OFF'), ('TGFbetaR', 'OFF'))
(('SMAD', 'OFF'), ('FAK', 'OFF'))
(('VIM', 'OFF'), ('p53', 'OFF'))
(('VIM', 'OFF'), ('p63', 'OFF'))
(('VIM', 'OFF'), ('p73', 'OFF'))
(('VIM', 'OFF'), ('miR203', 'OFF'))
(('VIM', 'OFF'), ('miR34', 'OFF'))
(('VIM', 'OFF'), ('miR200', 'OFF'))
(('VIM', 'OFF'), ('TGFbetaR', 'OFF'))
(('VIM', 'OFF'), ('FAK', 'OFF'))
(('p53', 'OFF'), ('p63', 'OFF'))
(('p53', 'OFF'), ('p73', 'OFF'))
(('p53', 'OFF'), ('miR203', 'OFF'))
(('p53', 'OFF'), ('miR34', 'OFF'))
(('p53', 'OFF'), ('miR200', 'OFF'))
(('p53', 'OFF'), ('TGFbetaR', 'OFF'))
(('p53', 'OFF'), ('FAK', 'OFF'))
(('p63', 'OFF'), ('p73', 'OFF'))
(('p63', 'OFF'), ('miR203', 'OFF'))
(('p63', 'OFF'), ('miR34', 'OFF'))
(('p63', 'OFF'), ('miR200', 'OFF'))
(('p63', 'OFF'), ('TGFbetaR', 'OFF'))
(('p63', 'OFF'), ('FAK', 'OFF'))
(('p73', 'OFF'), ('miR203', 'OFF'))
(('p73', 'OFF'), ('miR34', 'OFF'))
(('p73', 'OFF'), ('miR200', 'OFF'))
(('p73', 'OFF'), ('TGFbetaR', 'OFF'))
(('p73', 'OFF'), ('FAK', 'OFF'))
(('miR203', 'OFF'), ('miR34', 'OFF'))
(('miR203', 'OFF'), ('miR200', 'OFF'))
(('miR203', 'OFF'), ('TGFbetaR', 'OFF'))
(('miR203', 'OFF'), ('FAK', 'OFF'))
(('miR34', 'OFF'), ('miR200', 'OFF'))
(('miR34', 'OFF'), ('TGFbetaR', 'OFF'))
(('miR34', 'OFF'), ('FAK', 'OFF'))
(('miR200', 'OFF'), ('TGFbetaR', 'OFF'))
(('miR200', 'OFF'), ('FAK', 'OFF'))
(('TGFbetaR', 'OFF'), ('FAK', 'OFF'))
```

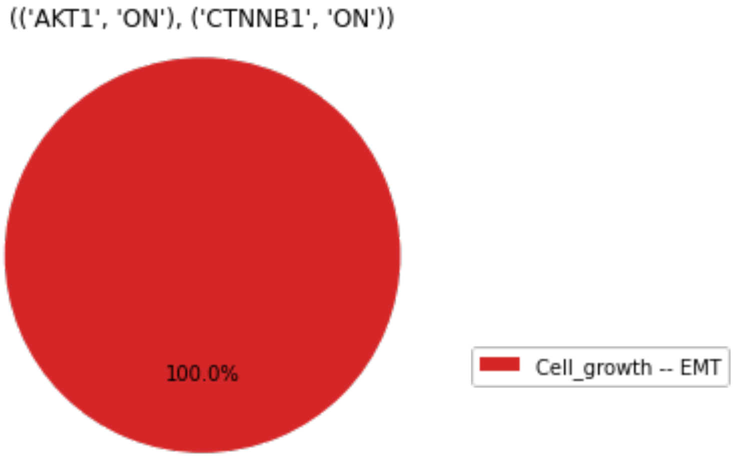

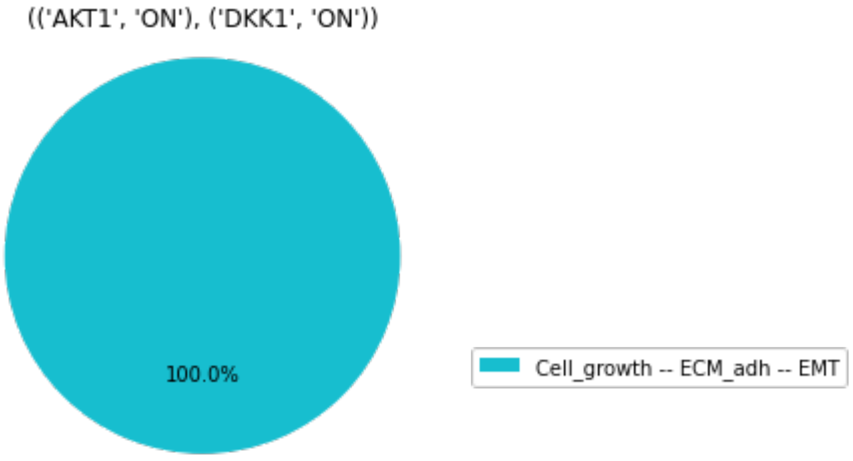

(('AKT1', 'ON'), ('AKT2', 'ON'))

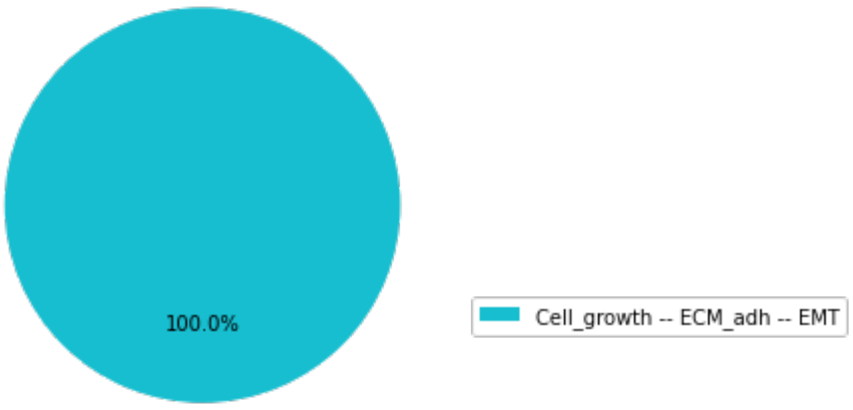

(('AKT1', 'ON'), ('RAC1', 'ON'))

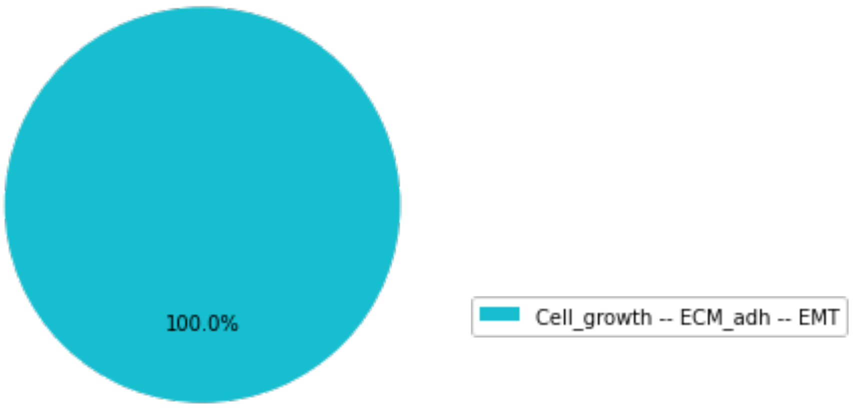

(('AKT1', 'ON'), ('YAP1', 'ON'))

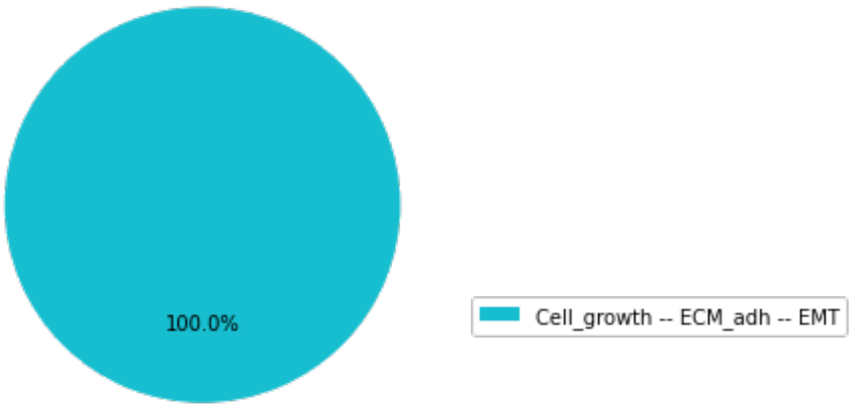

(('AKT1', 'ON'), ('PIK3CA', 'ON'))

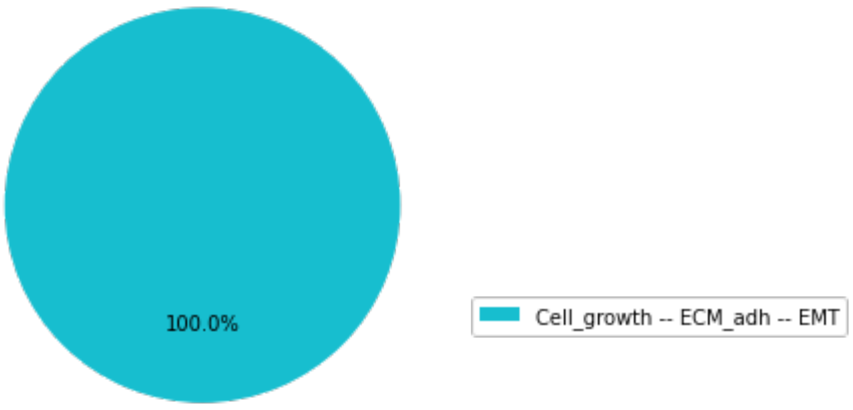

(('AKT1', 'ON'), ('ERK', 'ON'))

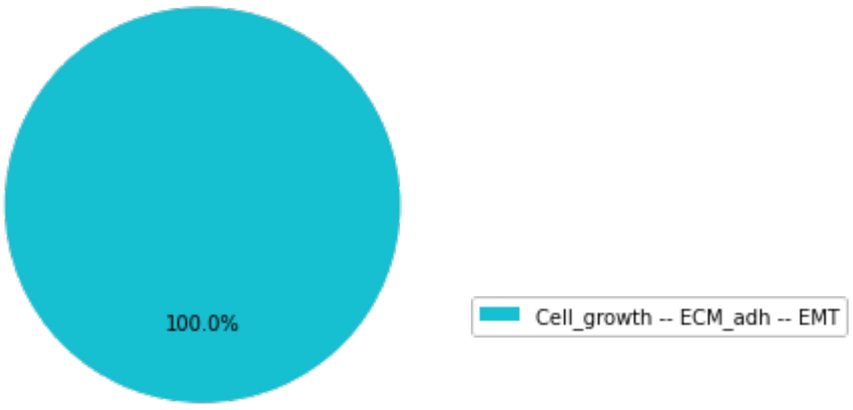

(('AKT1', 'ON'), ('p21', 'ON'))

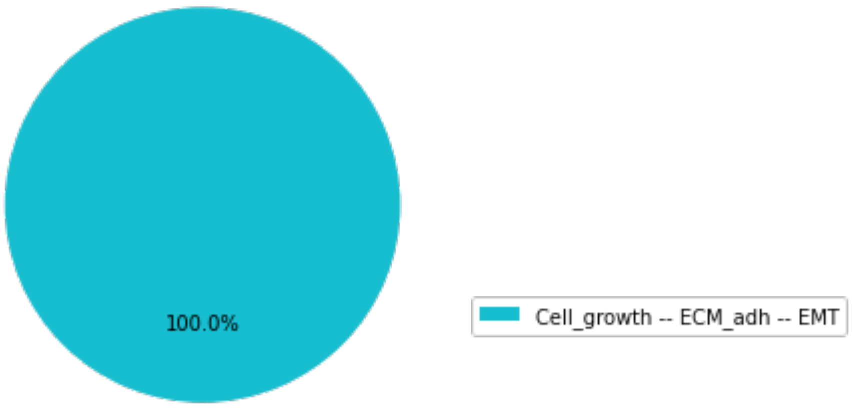

(('AKT1', 'ON'), ('SMAD', 'ON'))

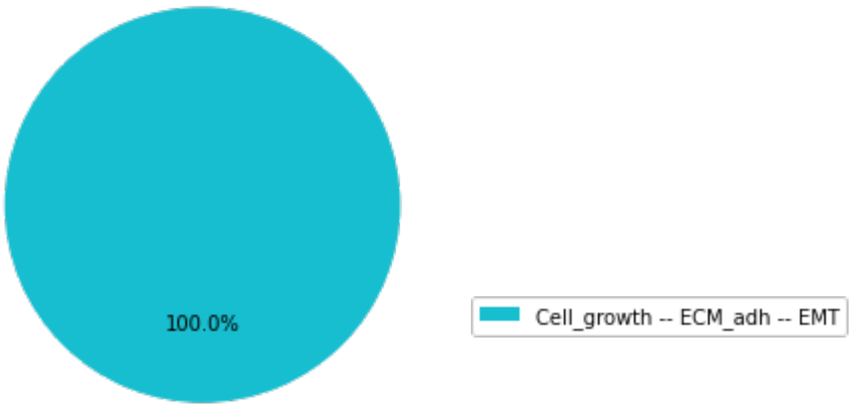

(('AKT1', 'ON'), ('VIM', 'ON'))

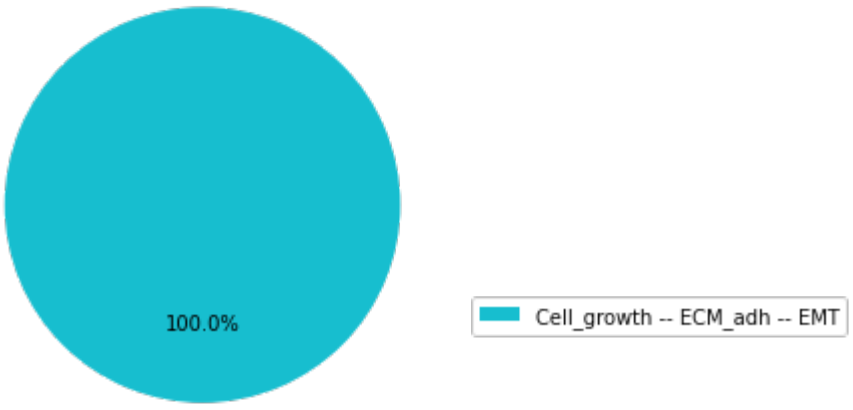

(('AKT1', 'ON'), ('p53', 'ON'))

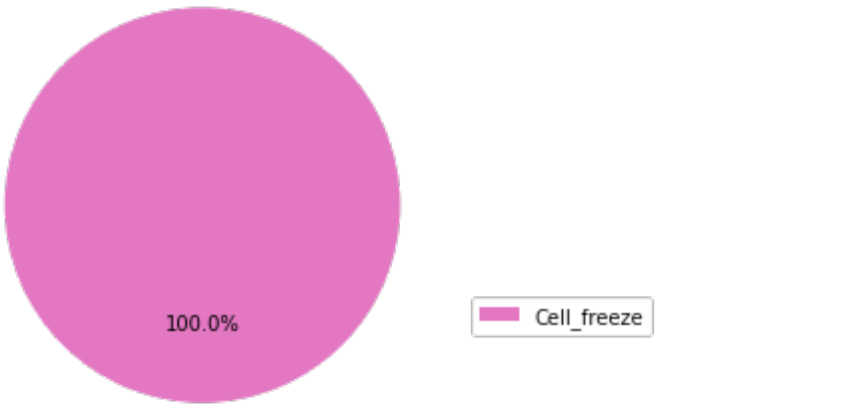

(('AKT1', 'ON'), ('p63', 'ON'))

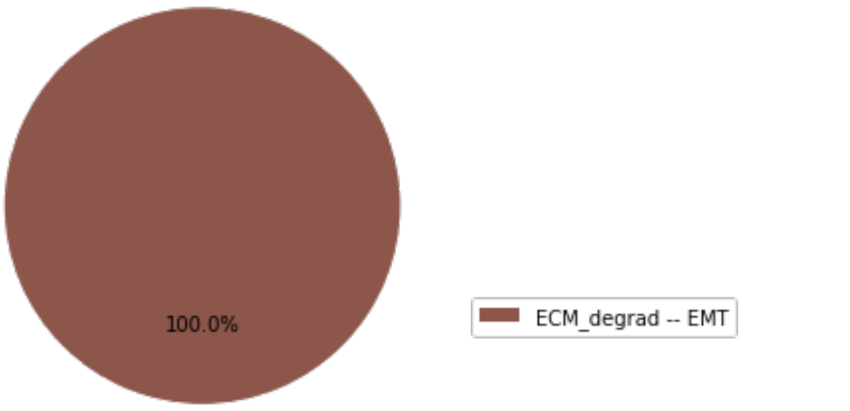

(('AKT1', 'ON'), ('p73', 'ON'))

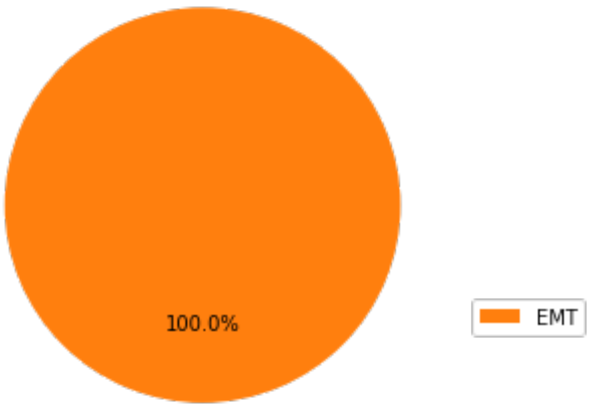

(('AKT1', 'ON'), ('miR203', 'ON'))

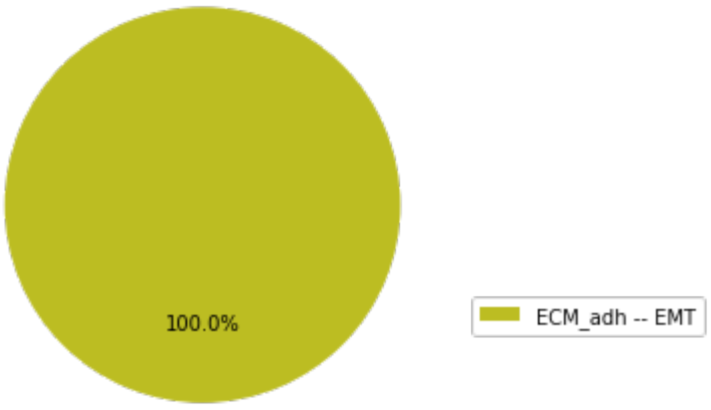

(('AKT1', 'ON'), ('miR34', 'ON'))

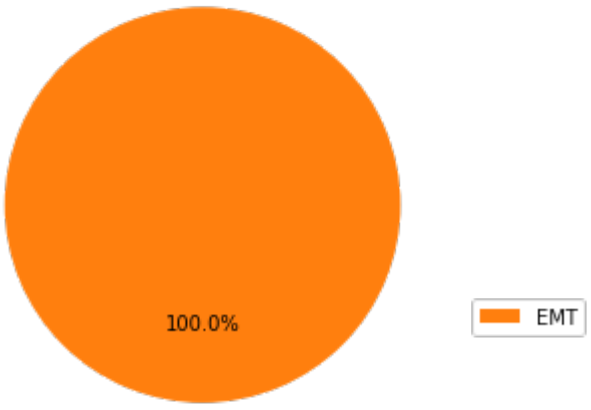

(('AKT1', 'ON'), ('miR200', 'ON'))

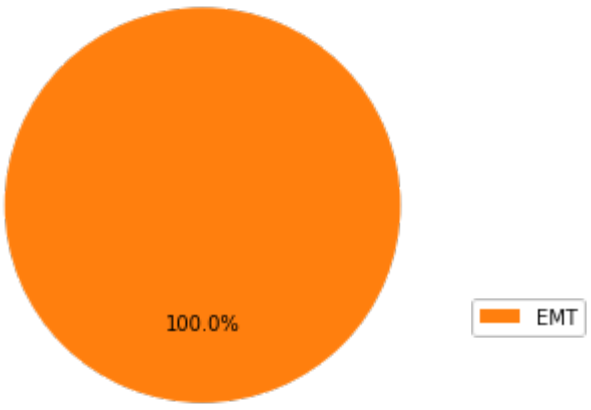

(('AKT1', 'ON'), ('TGFbetaR', 'ON'))

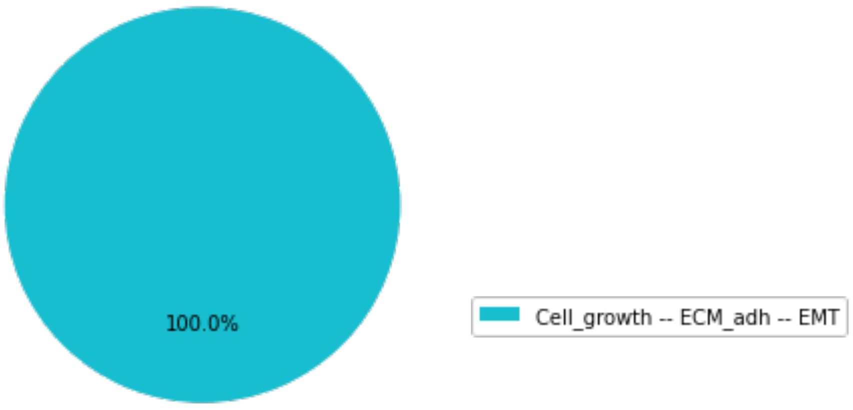

(('AKT1', 'ON'), ('FAK', 'ON'))

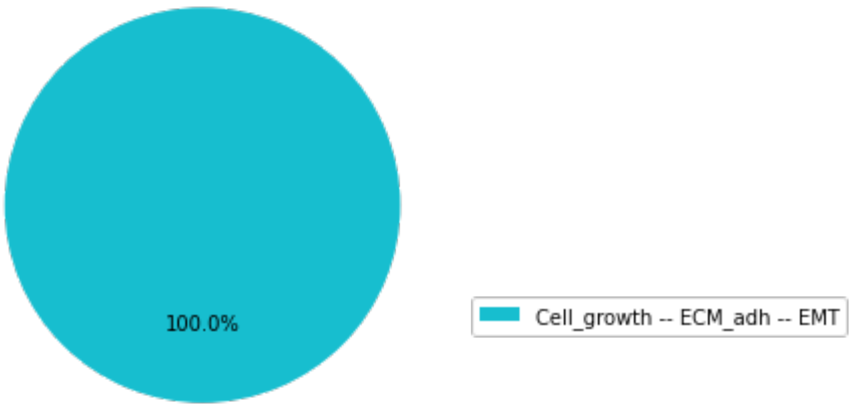

(('AKT1', 'ON'), ('CTNNB1', 'OFF'))

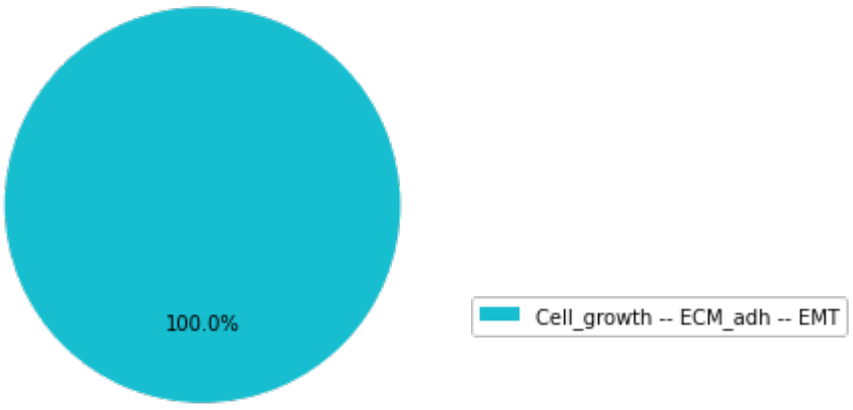

(('AKT1', 'ON'), ('DKK1', 'OFF'))

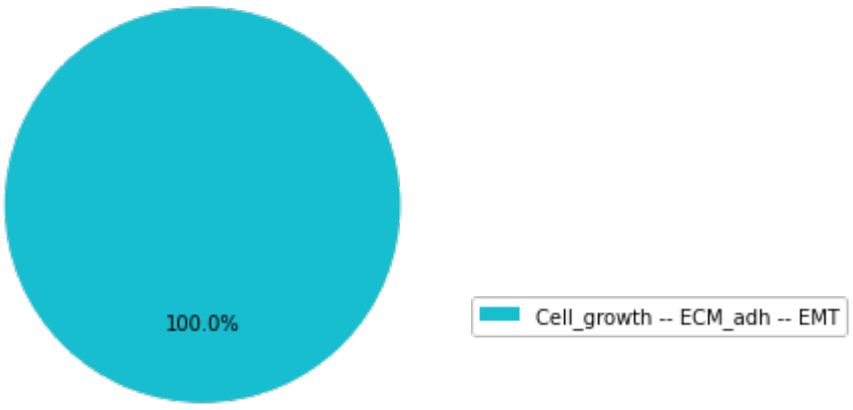

(('AKT1', 'ON'), ('AKT2', 'OFF'))

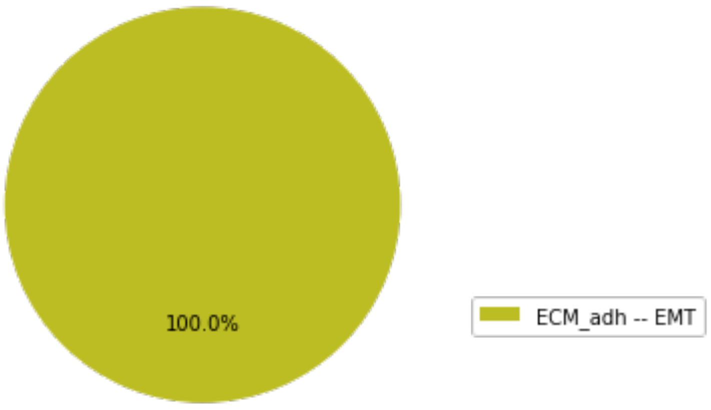

(('AKT1', 'ON'), ('RAC1', 'OFF'))

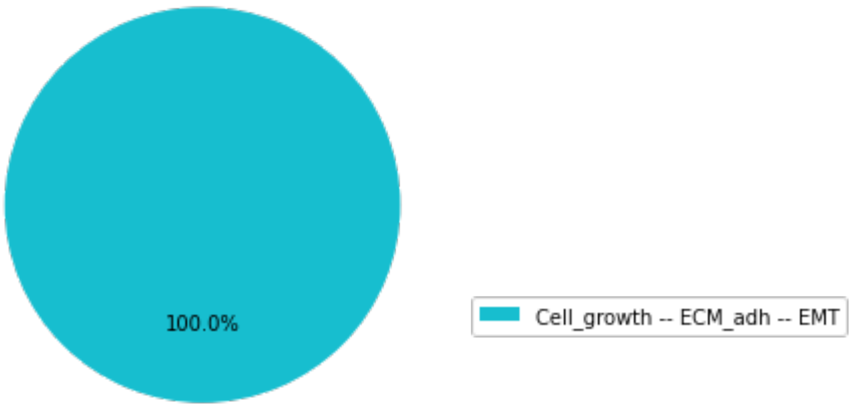

(('AKT1', 'ON'), ('YAP1', 'OFF'))

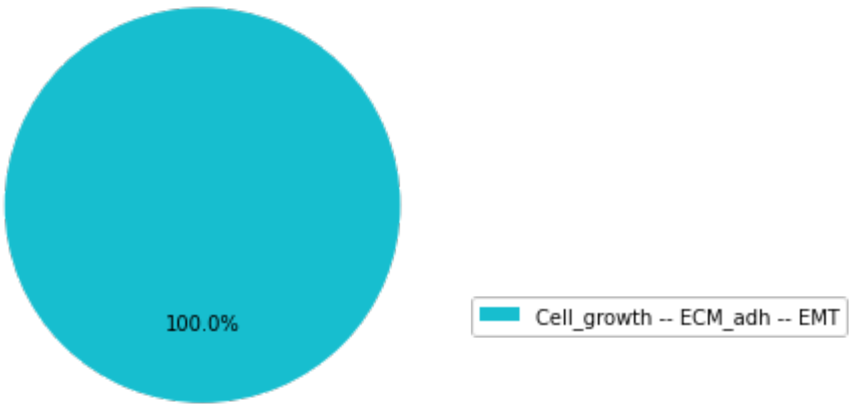

(('AKT1', 'ON'), ('PIK3CA', 'OFF'))

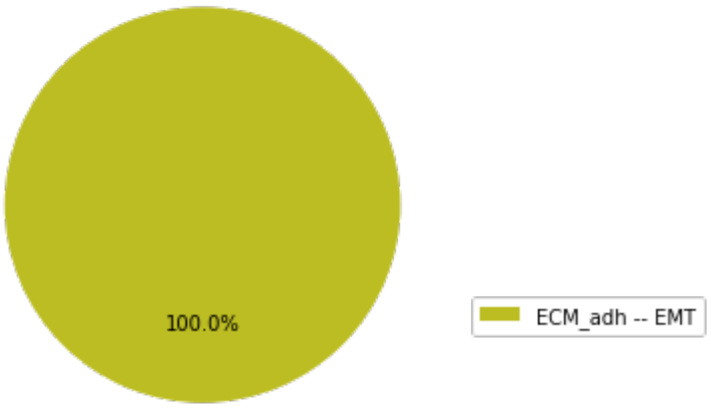

(('AKT1', 'ON'), ('ERK', 'OFF'))

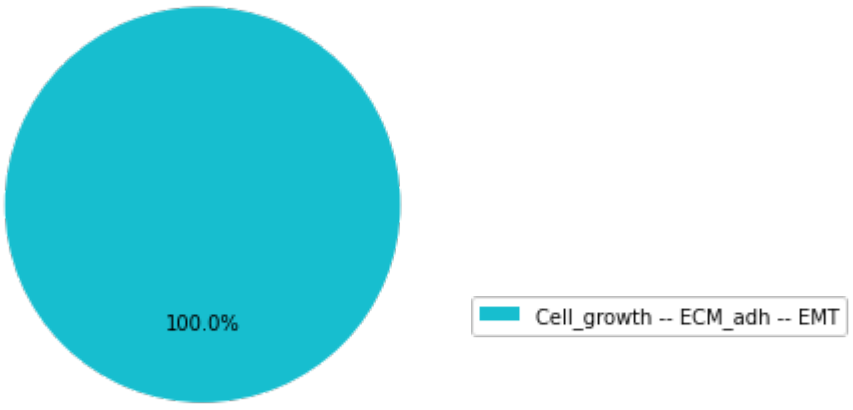

(('AKT1', 'ON'), ('p21', 'OFF'))

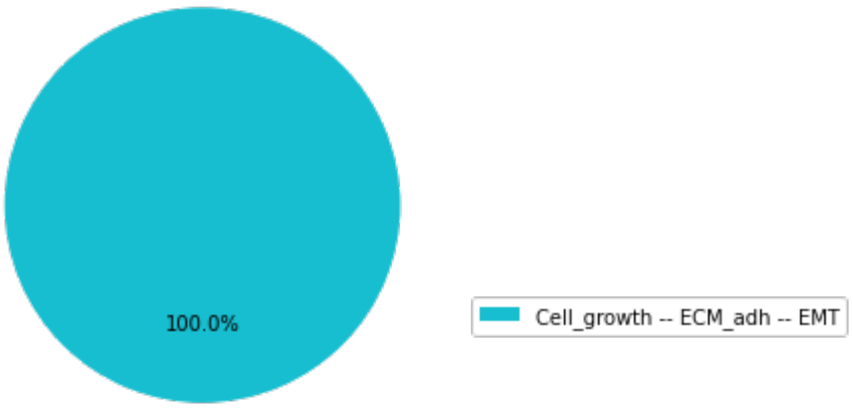

(('AKT1', 'ON'), ('SMAD', 'OFF'))

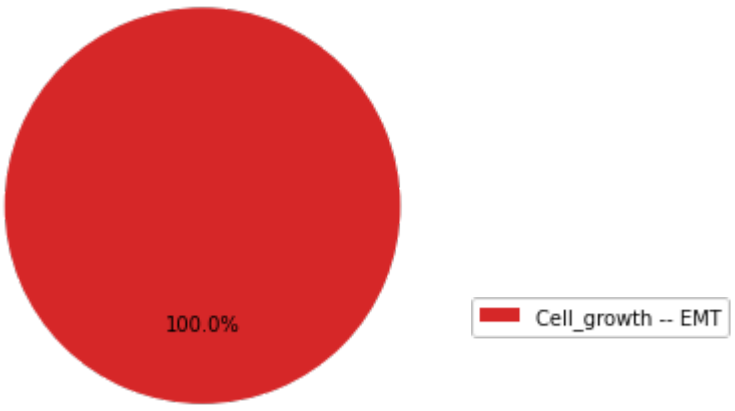

(('AKT1', 'ON'), ('VIM', 'OFF'))

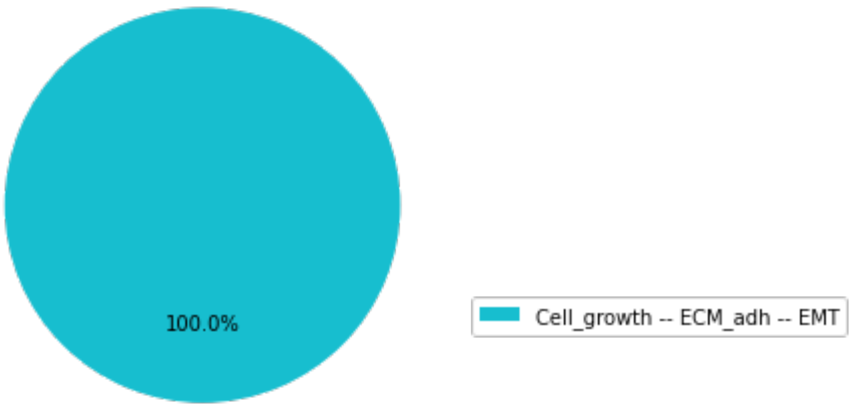

(('AKT1', 'ON'), ('p53', 'OFF'))

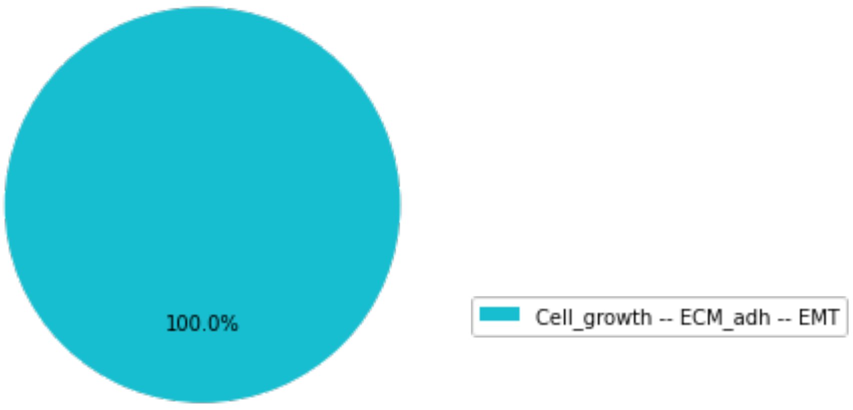

(('AKT1', 'ON'), ('p63', 'OFF'))

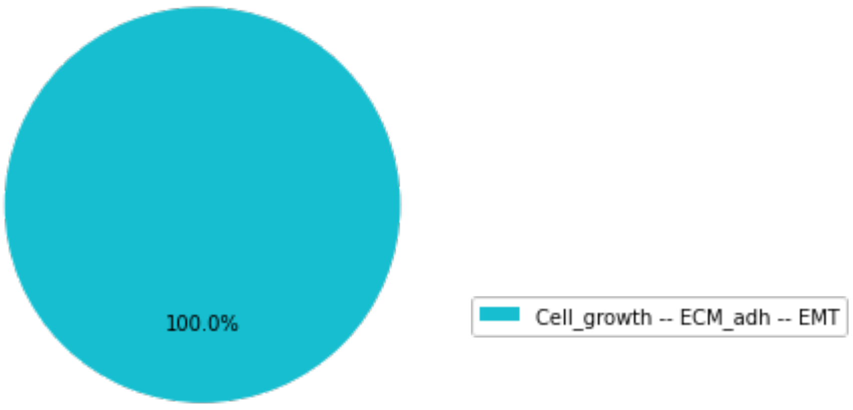

(('AKT1', 'ON'), ('p73', 'OFF'))

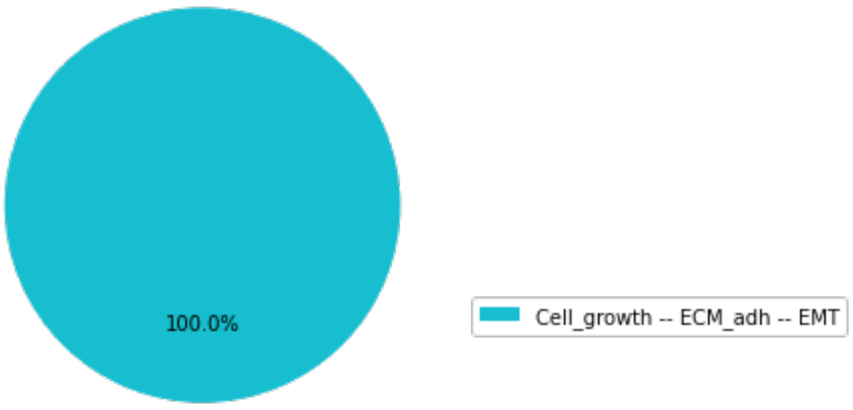

(('AKT1', 'ON'), ('miR203', 'OFF'))

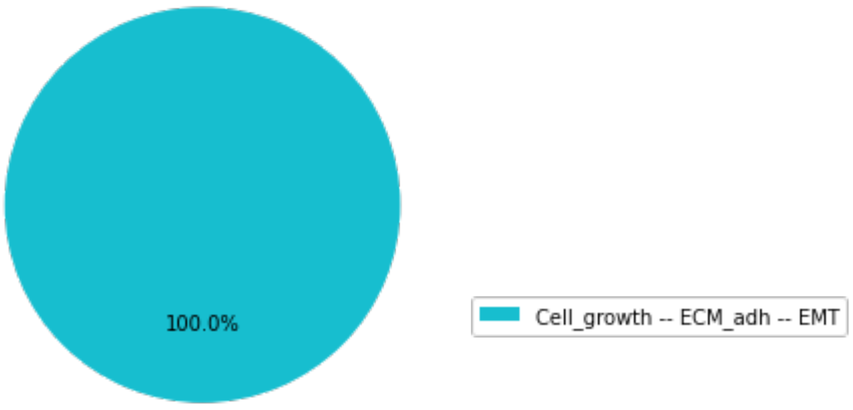

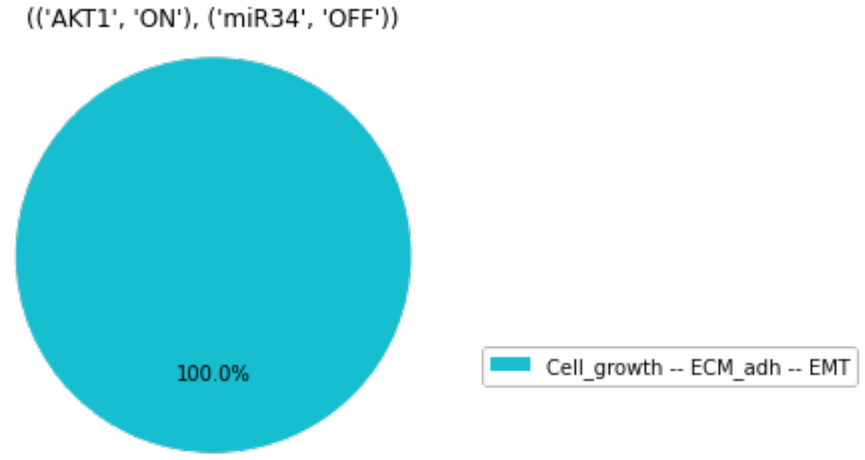
(('AKT1', 'ON'), ('miR34', 'OFF'))
100.0%
Cell_growth -- ECM_adh -- EMT

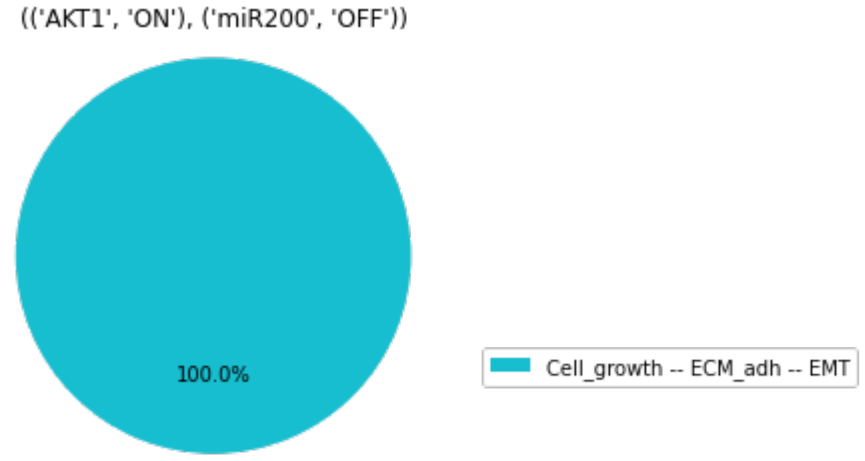
(('AKT1', 'ON'), ('miR200', 'OFF'))
100.0%
Cell_growth -- ECM_adh -- EMT

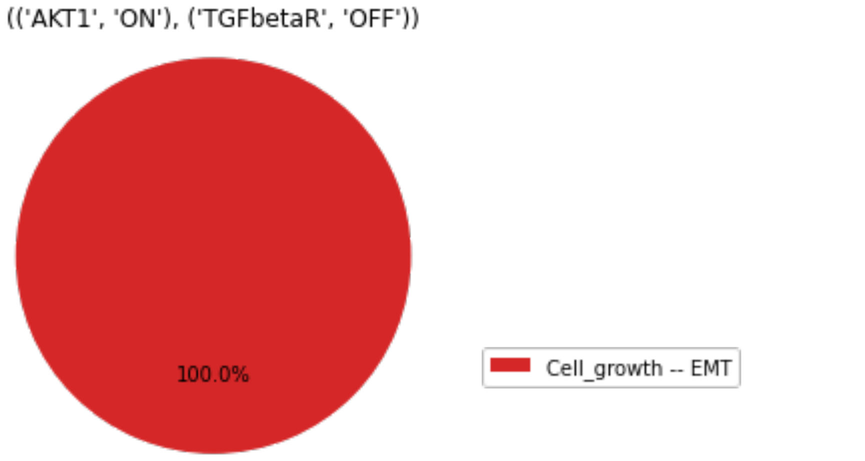
(('AKT1', 'ON'), ('TGFbetaR', 'OFF'))
100.0%
Cell_growth -- EMT

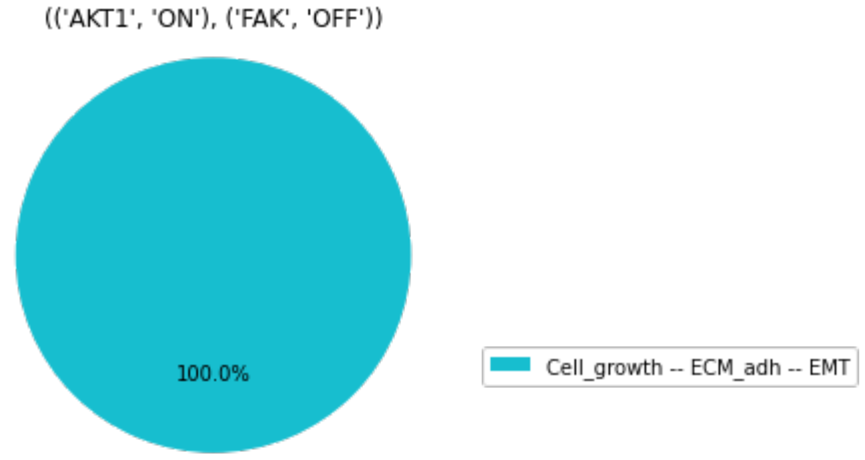
(('AKT1', 'ON'), ('FAK', 'OFF'))
100.0%
Cell_growth -- ECM_adh -- EMT

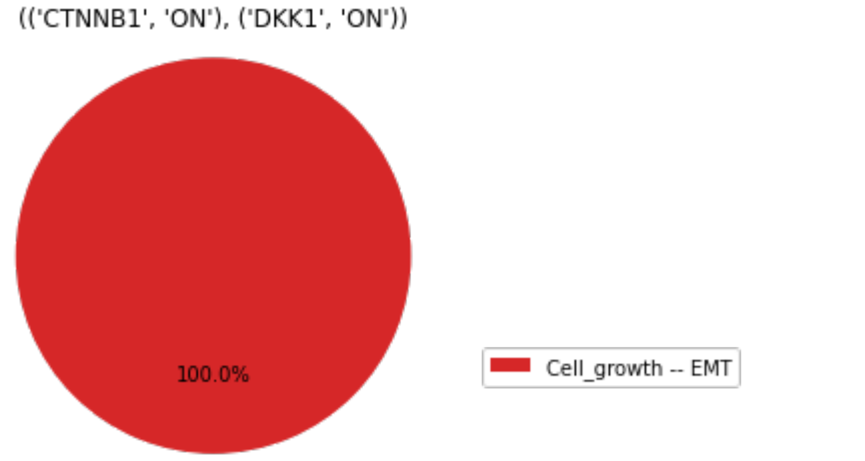
(('CTNNB1', 'ON'), ('DKK1', 'ON'))
100.0%
Cell_growth -- EMT

(('CTNNB1', 'ON'), ('AKT2', 'ON'))

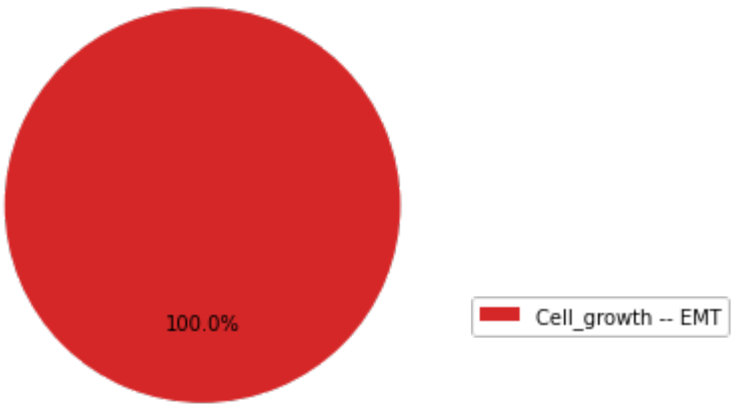

(('CTNNB1', 'ON'), ('RAC1', 'ON'))

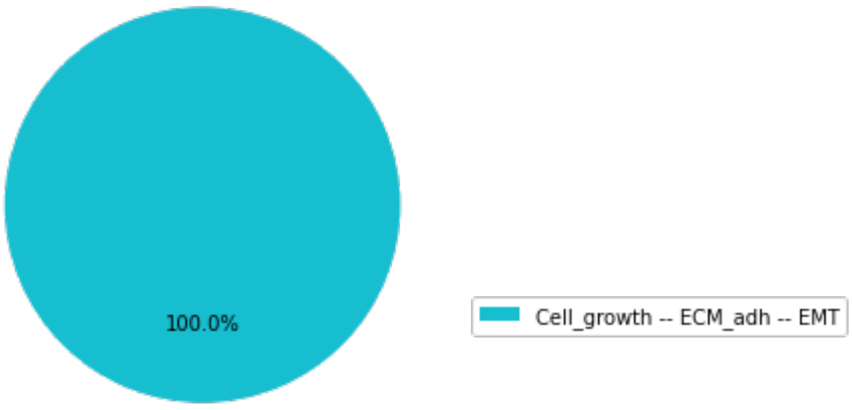

(('CTNNB1', 'ON'), ('YAP1', 'ON'))

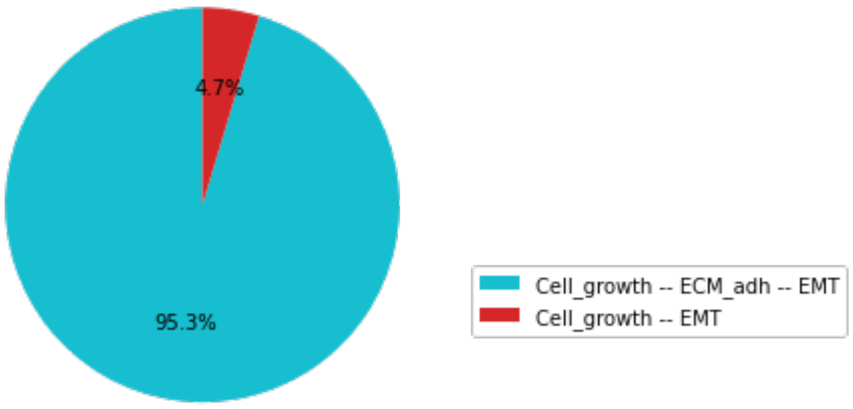

(('CTNNB1', 'ON'), ('PIK3CA', 'ON'))

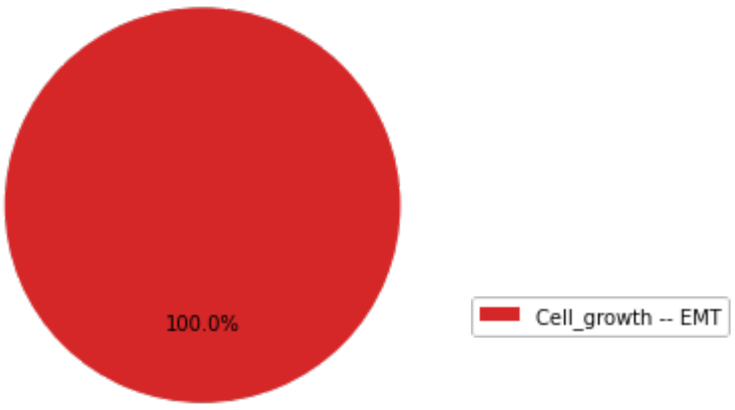

(('CTNNB1', 'ON'), ('ERK', 'ON'))

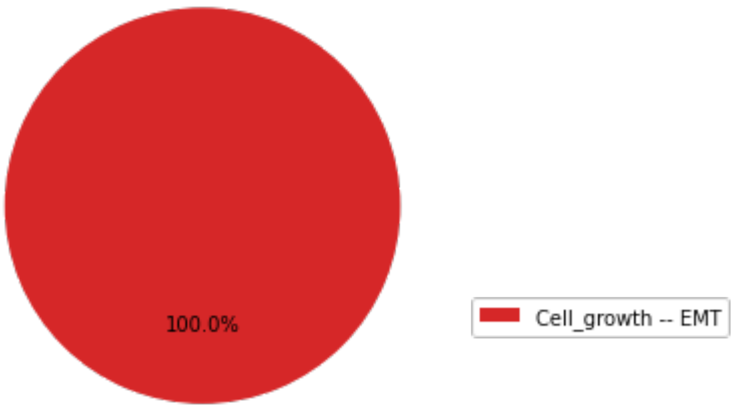

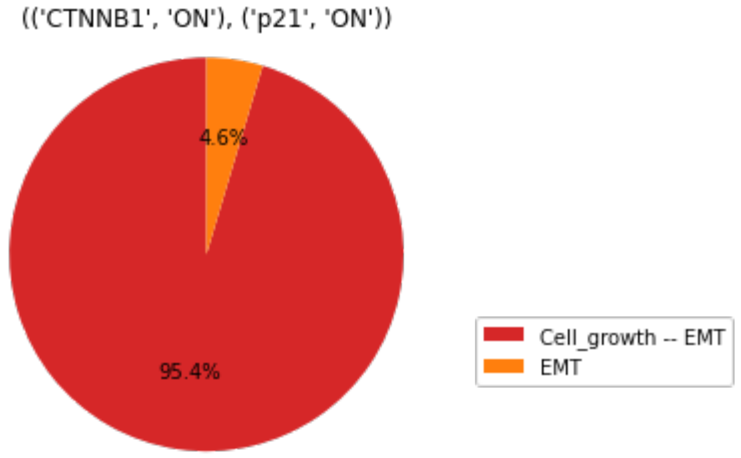

(('CTNNB1', 'ON'), ('p21', 'ON'))
4.6%
95.4%
Cell_growth -- EMT
EMT

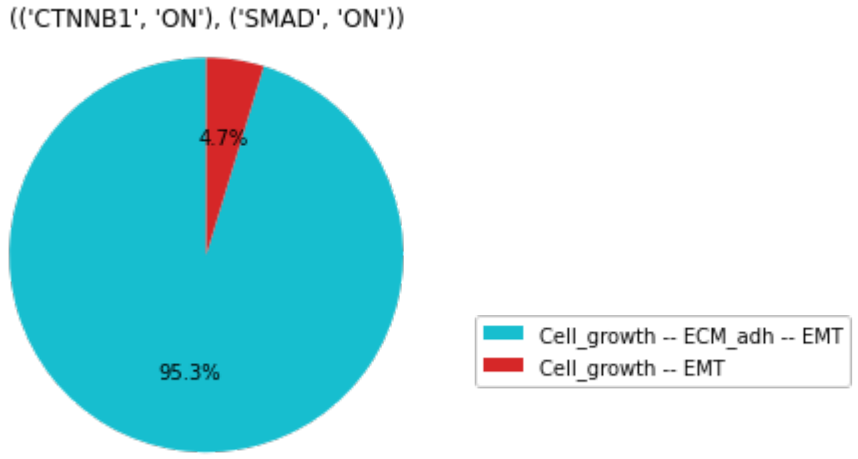

(('CTNNB1', 'ON'), ('SMAD', 'ON'))
4.7%
95.3%
Cell_growth -- ECM_adh -- EMT
Cell_growth -- EMT

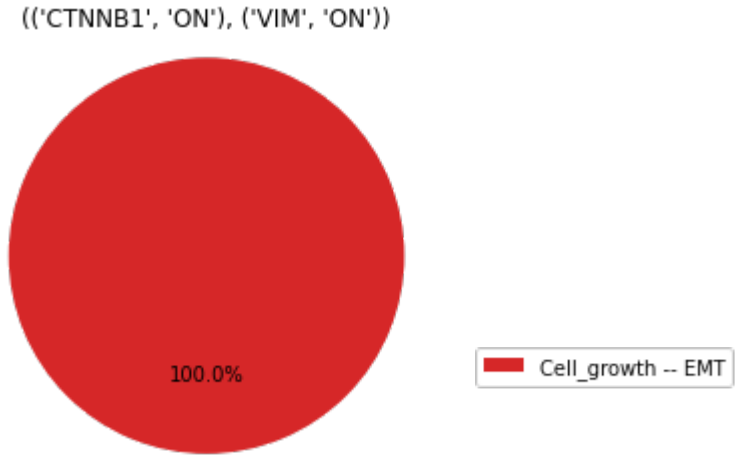

(('CTNNB1', 'ON'), ('VIM', 'ON'))
100.0%
Cell_growth -- EMT

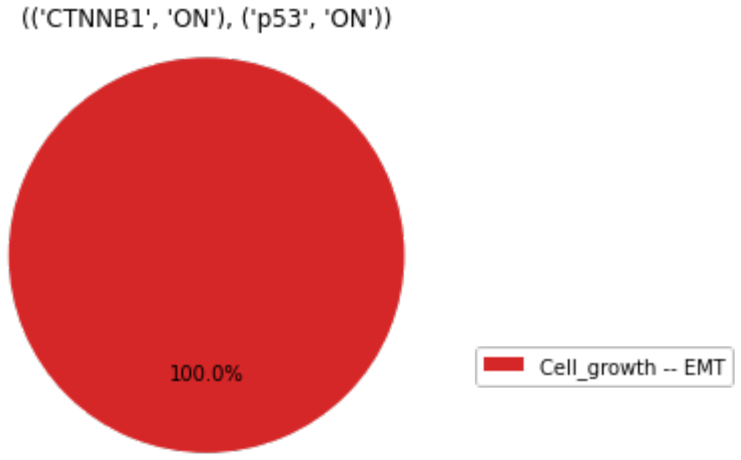

(('CTNNB1', 'ON'), ('p53', 'ON'))
100.0%
Cell_growth -- EMT

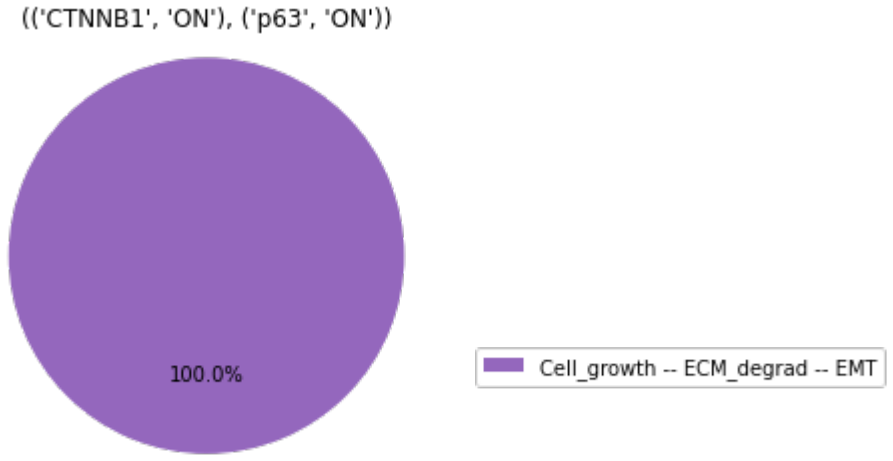

(('CTNNB1', 'ON'), ('p63', 'ON'))
100.0%
Cell_growth -- ECM_degrad -- EMT

(('CTNNB1', 'ON'), ('p73', 'ON'))

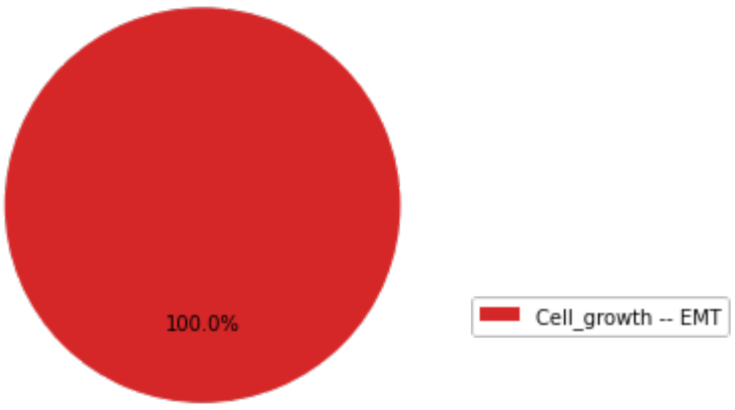

(('CTNNB1', 'ON'), ('miR203', 'ON'))

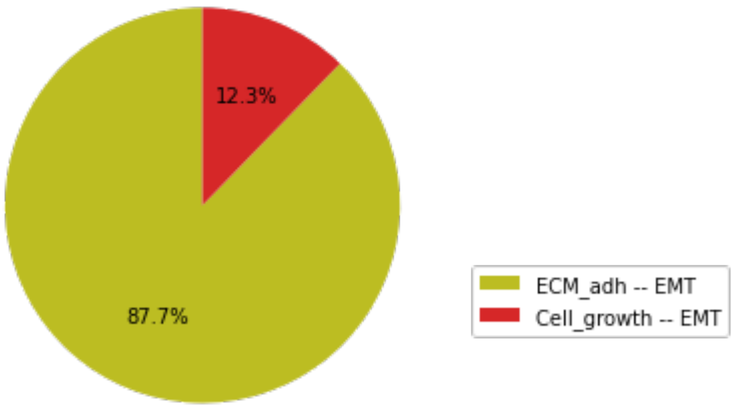

(('CTNNB1', 'ON'), ('miR34', 'ON'))

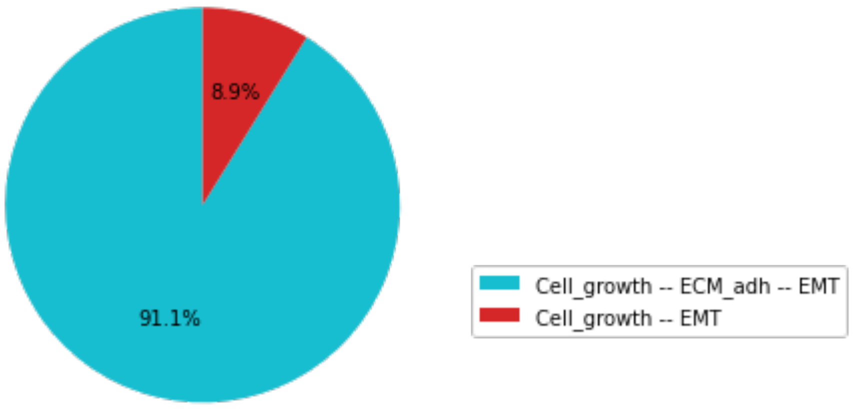

(('CTNNB1', 'ON'), ('miR200', 'ON'))

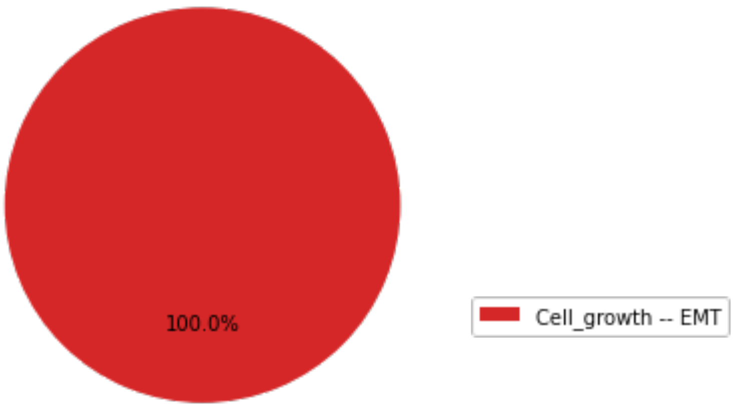

(('CTNNB1', 'ON'), ('TGFbetaR', 'ON'))

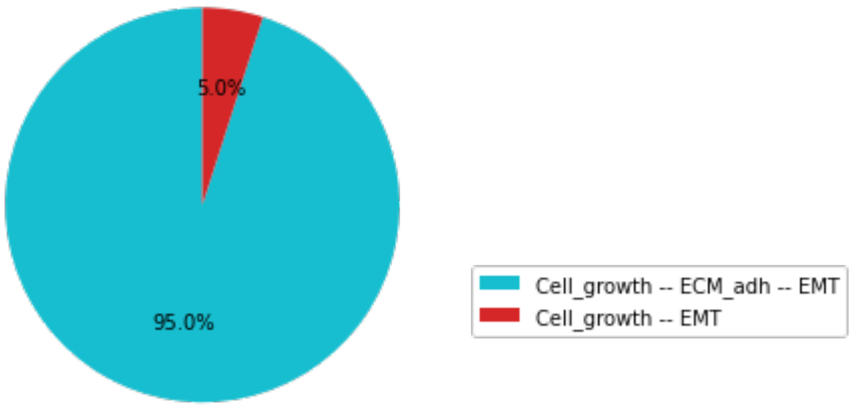

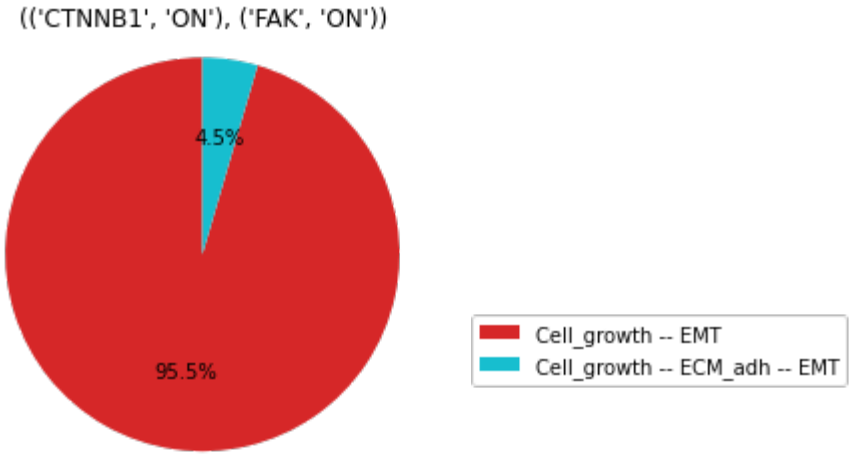
(('CTNNB1', 'ON'), ('FAK', 'ON'))
4.5%
95.5%
Cell_growth -- EMT
Cell_growth -- ECM_adh -- EMT

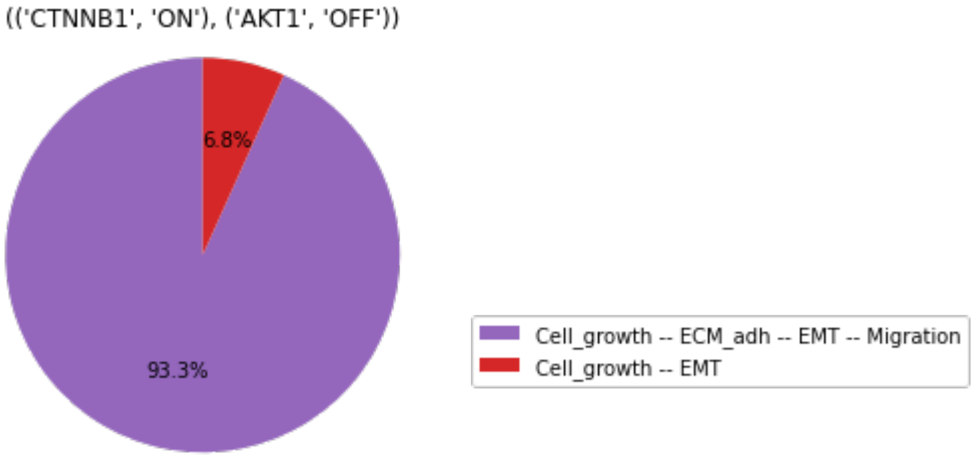
(('CTNNB1', 'ON'), ('AKT1', 'OFF'))
6.8%
93.3%
Cell_growth -- ECM_adh -- EMT -- Migration
Cell_growth -- EMT

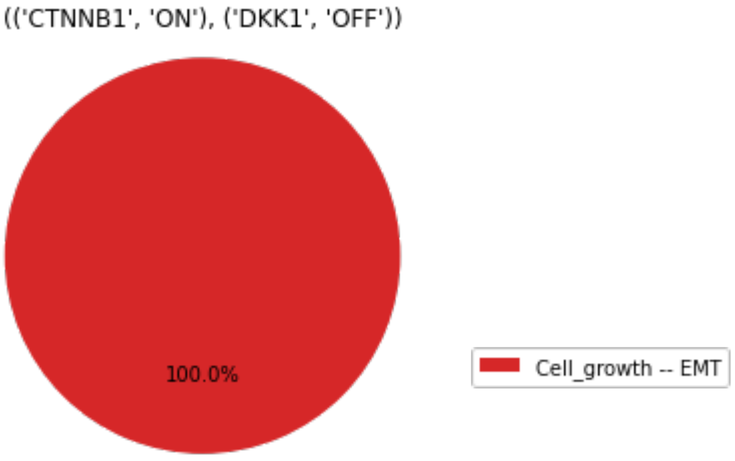
(('CTNNB1', 'ON'), ('DKK1', 'OFF'))
100.0%
Cell_growth -- EMT

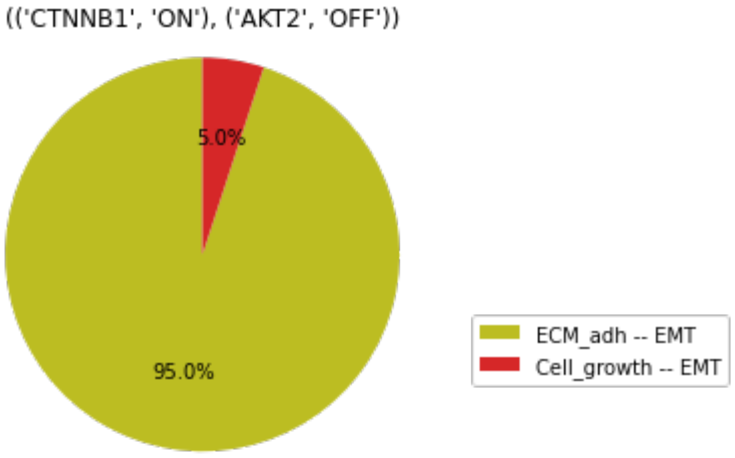
(('CTNNB1', 'ON'), ('AKT2', 'OFF'))
5.0%
95.0%
ECM_adh -- EMT
Cell_growth -- EMT

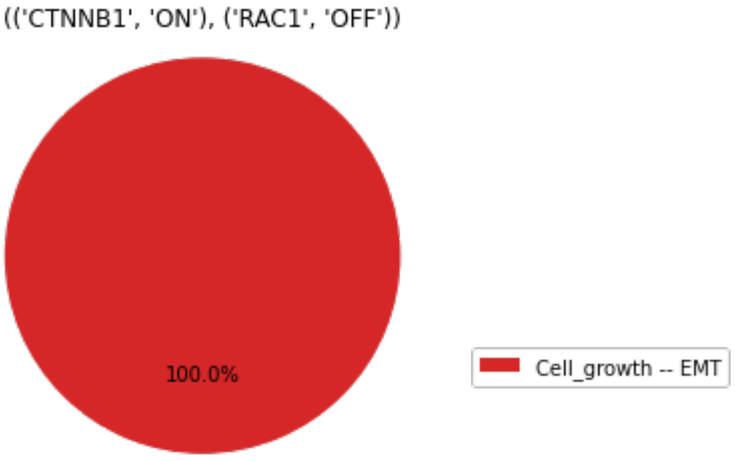
(('CTNNB1', 'ON'), ('RAC1', 'OFF'))
100.0%
Cell_growth -- EMT

(('CTNNB1', 'ON'), ('YAP1', 'OFF'))

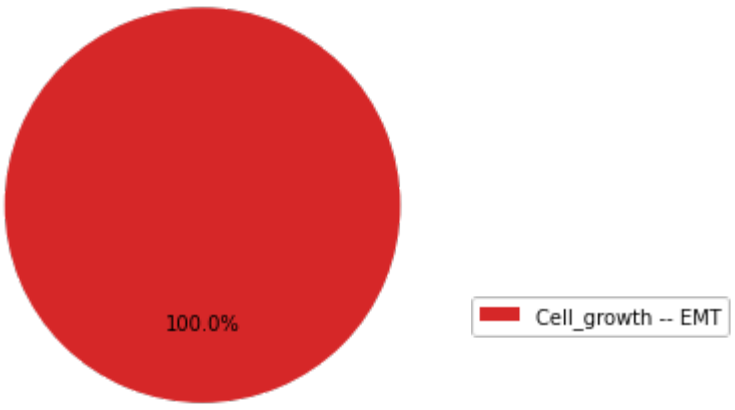

(('CTNNB1', 'ON'), ('PIK3CA', 'OFF'))

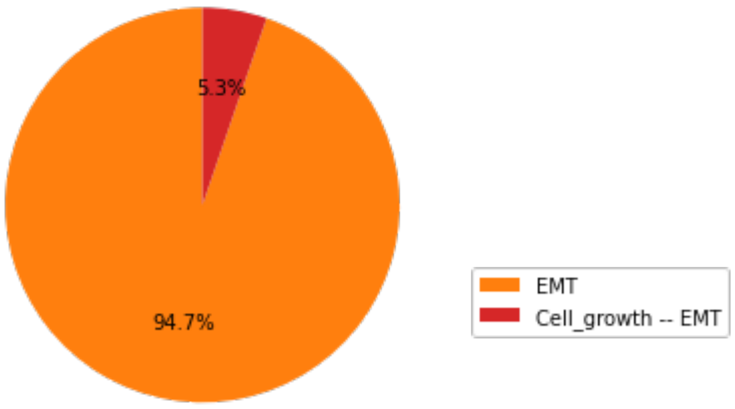

(('CTNNB1', 'ON'), ('ERK', 'OFF'))

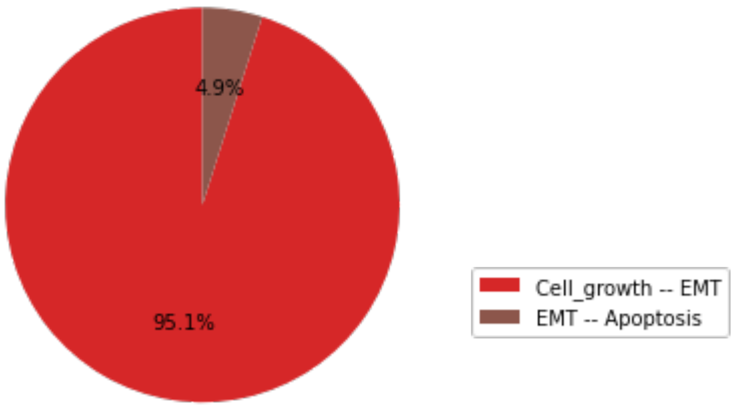

(('CTNNB1', 'ON'), ('p21', 'OFF'))

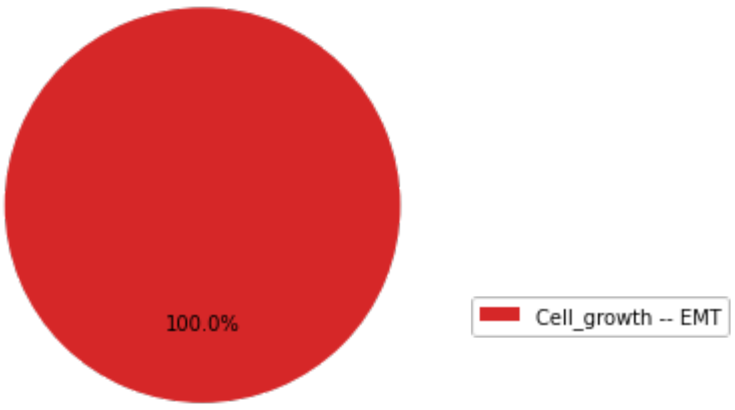

(('CTNNB1', 'ON'), ('SMAD', 'OFF'))

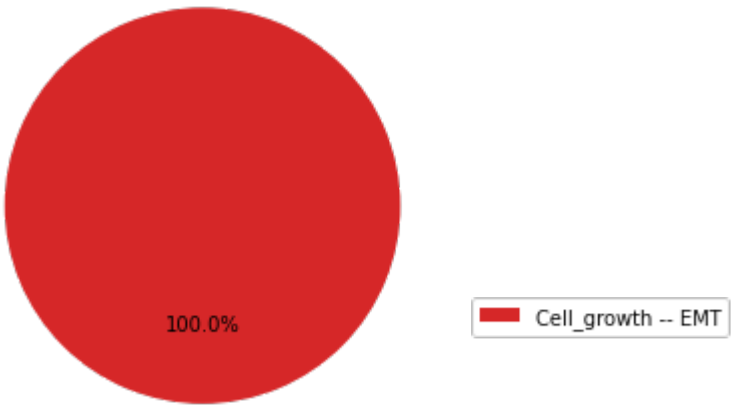

(('CTNNB1', 'ON'), ('VIM', 'OFF'))

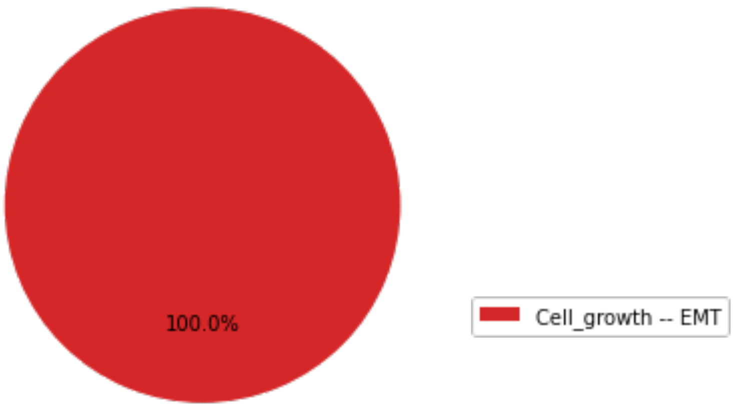

(('CTNNB1', 'ON'), ('p53', 'OFF'))

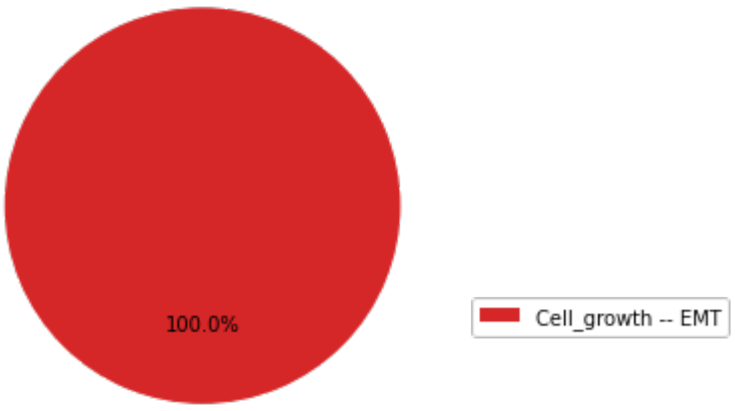

(('CTNNB1', 'ON'), ('p63', 'OFF'))

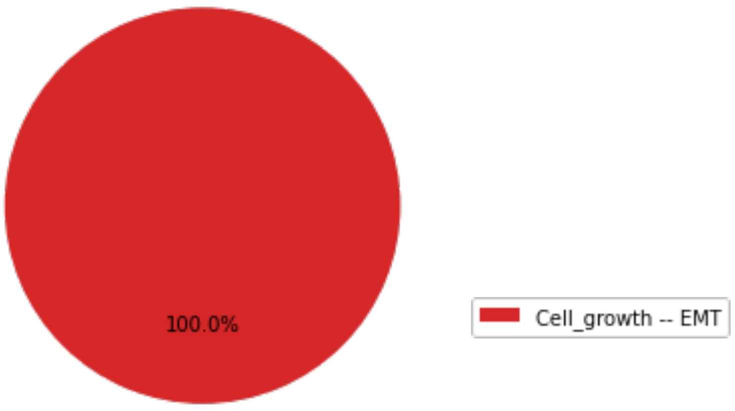

(('CTNNB1', 'ON'), ('p73', 'OFF'))

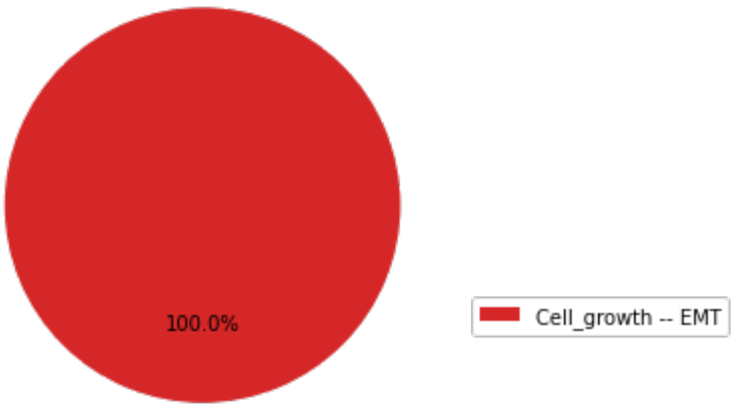

(('CTNNB1', 'ON'), ('miR203', 'OFF'))

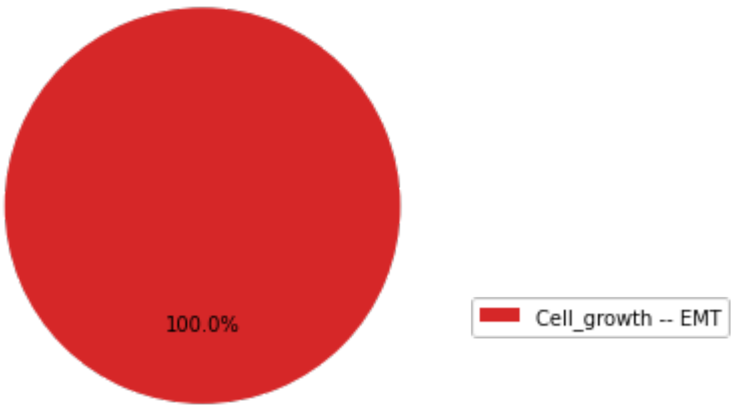

(('CTNNB1', 'ON'), ('miR34', 'OFF'))

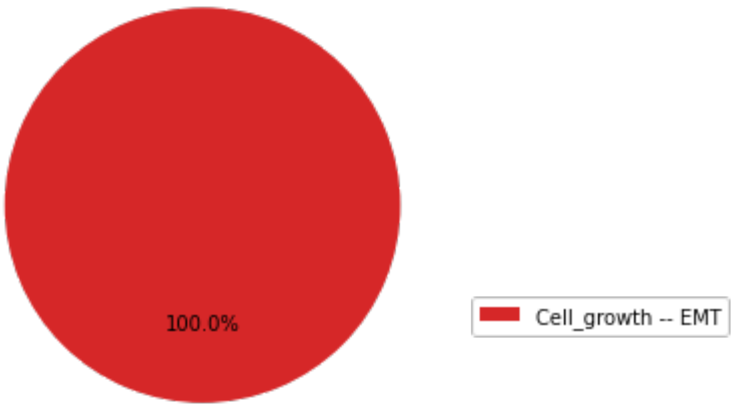

(('CTNNB1', 'ON'), ('miR200', 'OFF'))

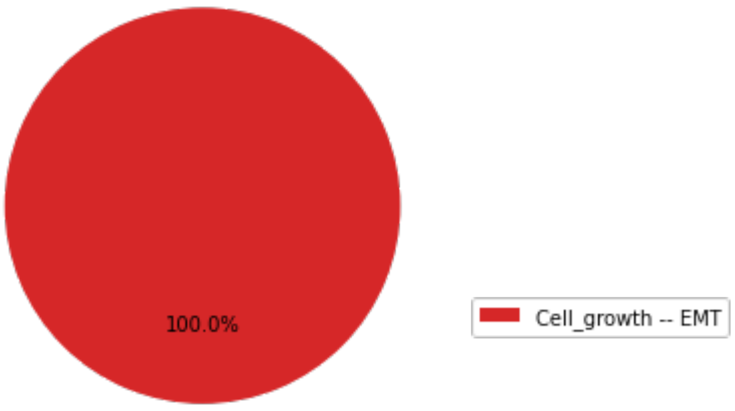

(('CTNNB1', 'ON'), ('TGFbetaR', 'OFF'))

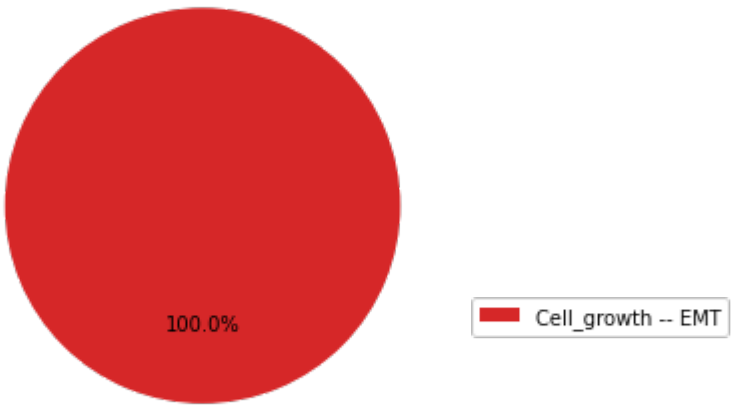

(('CTNNB1', 'ON'), ('FAK', 'OFF'))

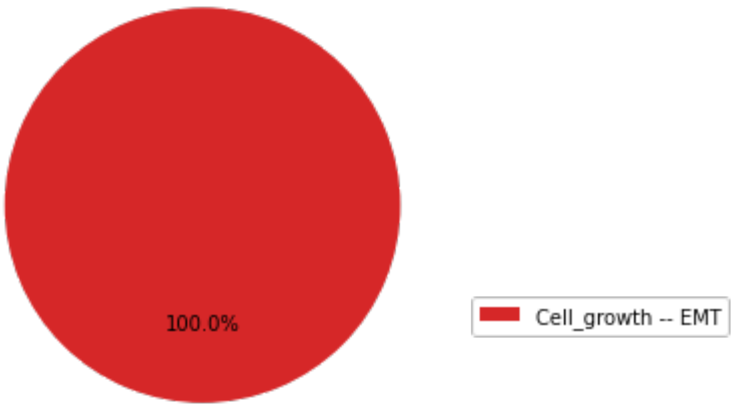

(('DKK1', 'ON'), ('AKT2', 'ON'))

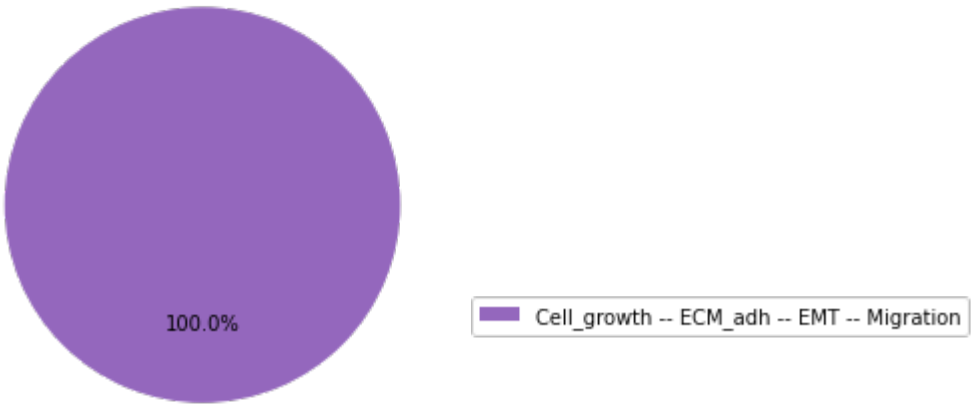

(('DKK1', 'ON'), ('RAC1', 'ON'))

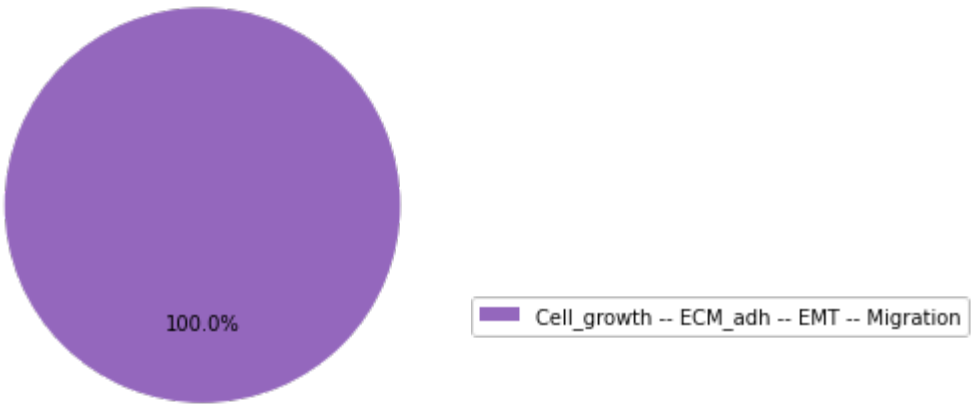

(('DKK1', 'ON'), ('YAP1', 'ON'))

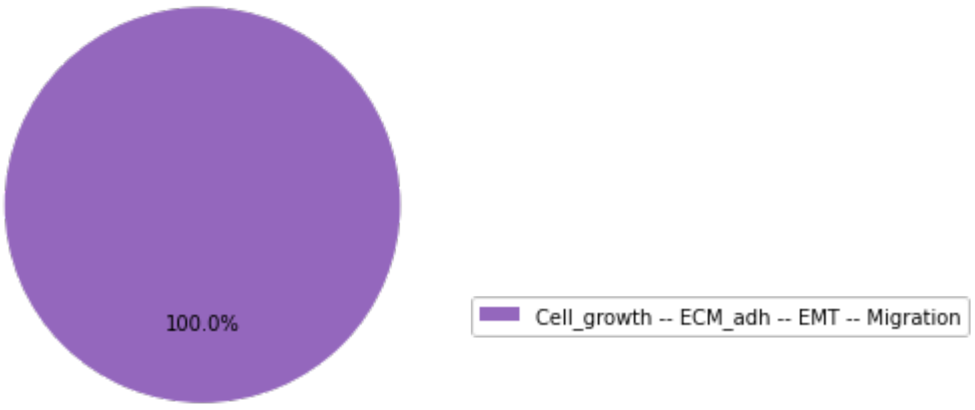

(('DKK1', 'ON'), ('PIK3CA', 'ON'))

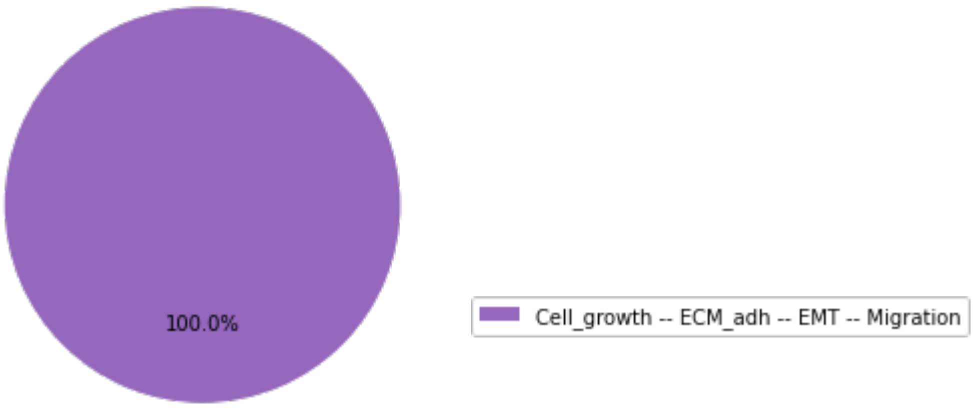

(('DKK1', 'ON'), ('ERK', 'ON'))

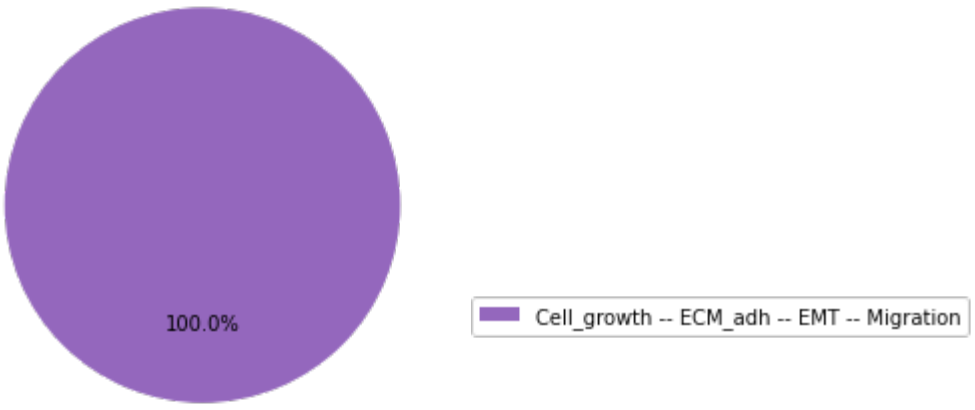

(('DKK1', 'ON'), ('p21', 'ON'))

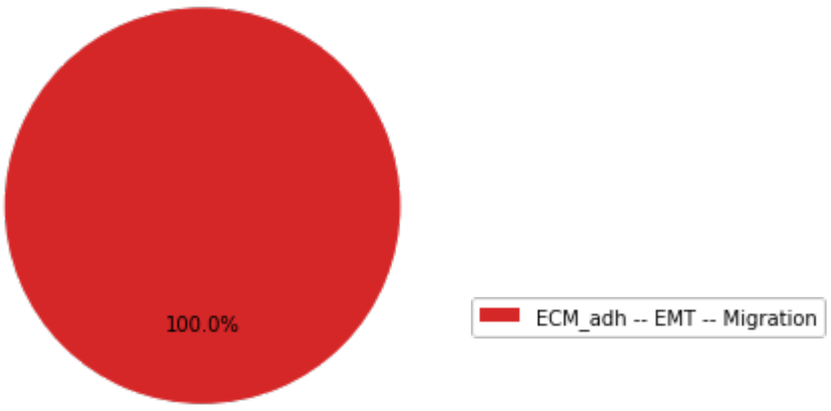

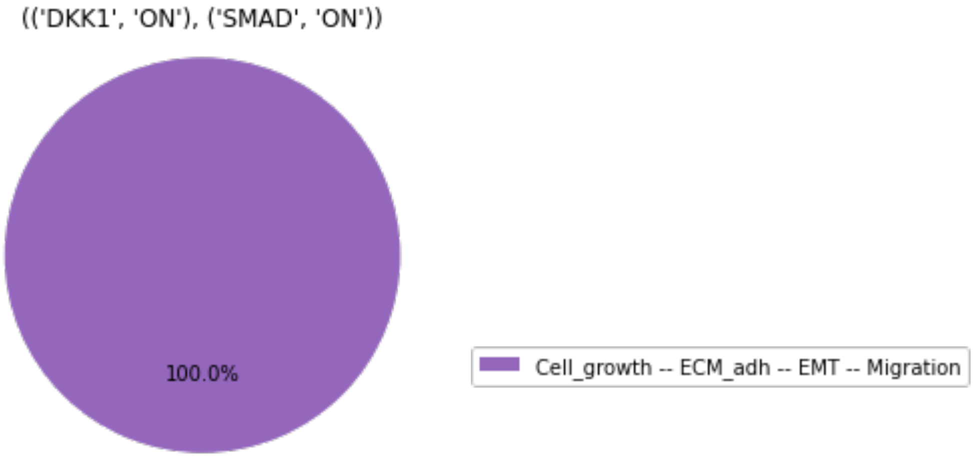
(('DKK1', 'ON'), ('SMAD', 'ON'))
100.0%
Cell_growth -- ECM_adh -- EMT -- Migration

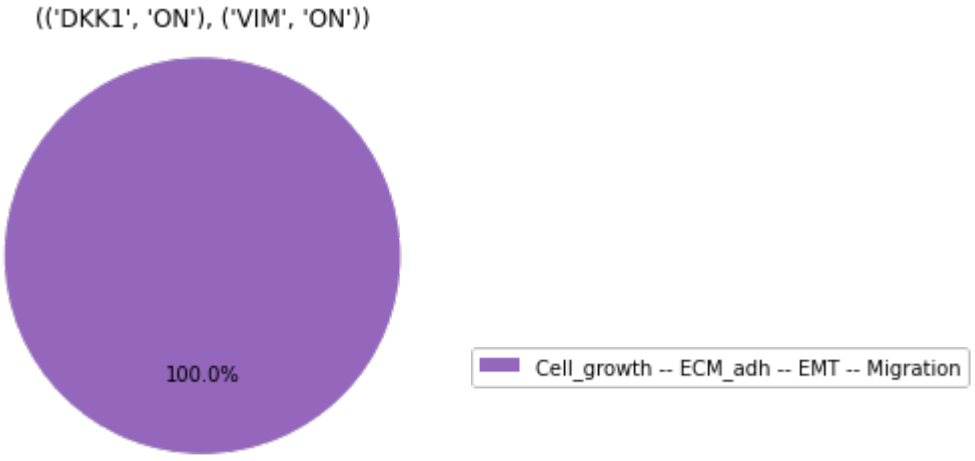
(('DKK1', 'ON'), ('VIM', 'ON'))
100.0%
Cell_growth -- ECM_adh -- EMT -- Migration

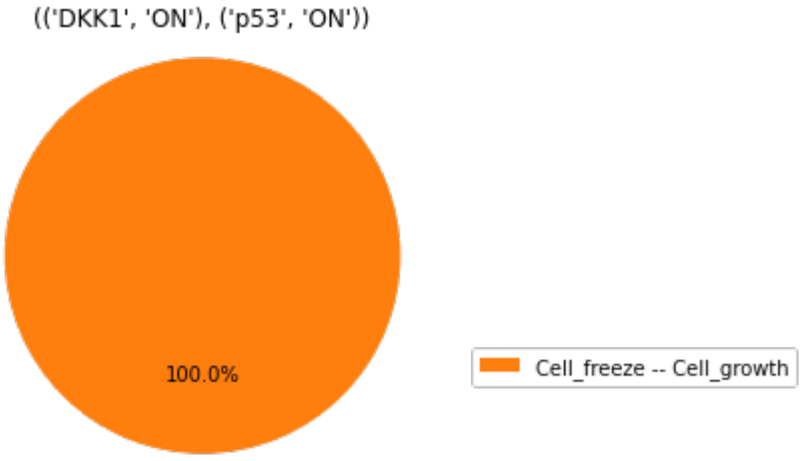
(('DKK1', 'ON'), ('p53', 'ON'))
100.0%
Cell_freeze -- Cell_growth

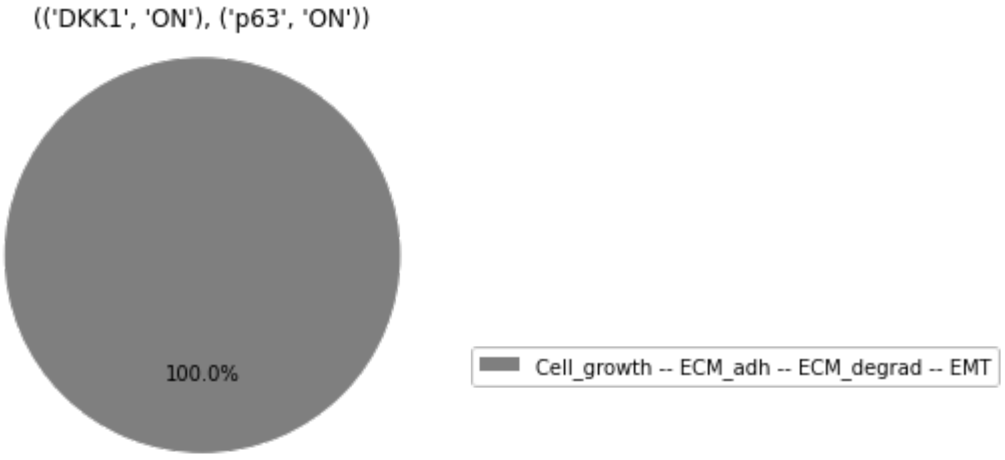
(('DKK1', 'ON'), ('p63', 'ON'))
100.0%
Cell_growth -- ECM_adh -- ECM_degrad -- EMT

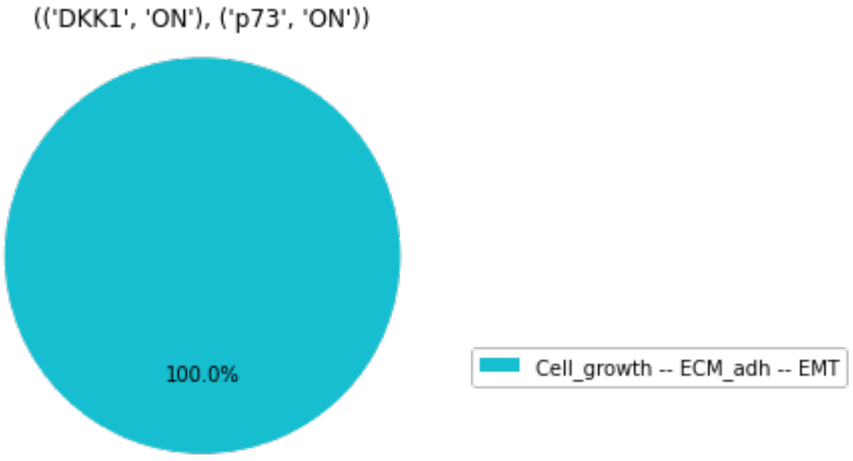
(('DKK1', 'ON'), ('p73', 'ON'))
100.0%
Cell_growth -- ECM_adh -- EMT

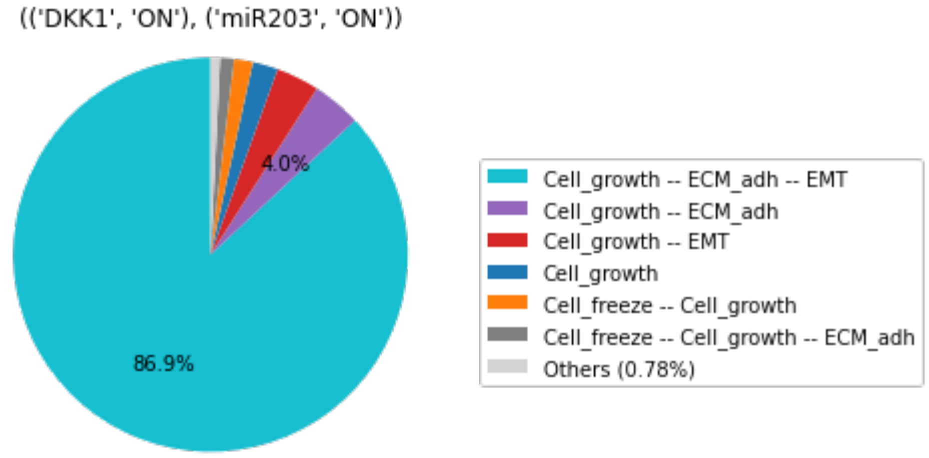
(('DKK1', 'ON'), ('miR203', 'ON'))
4.0%
86.9%
Cell_growth -- ECM_adh -- EMT
Cell_growth -- ECM_adh
Cell_growth -- EMT
Cell_growth
Cell_freeze -- Cell_growth
Cell_freeze -- Cell_growth -- ECM_adh
Others (0.78%)

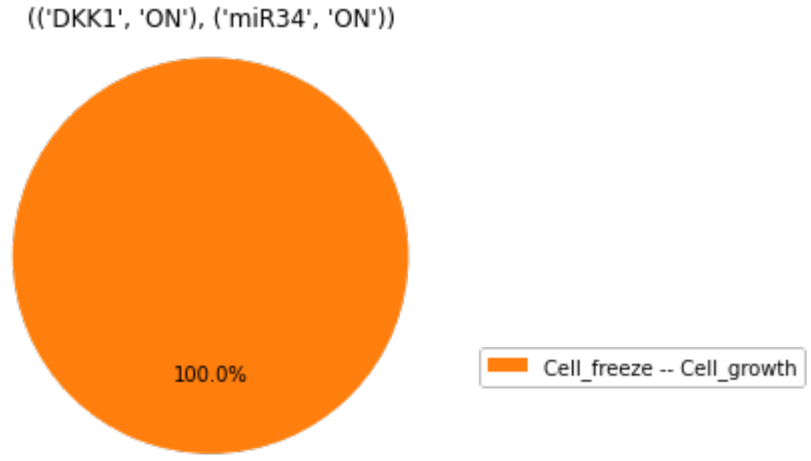
(('DKK1', 'ON'), ('miR34', 'ON'))
100.0%
Cell_freeze -- Cell_growth

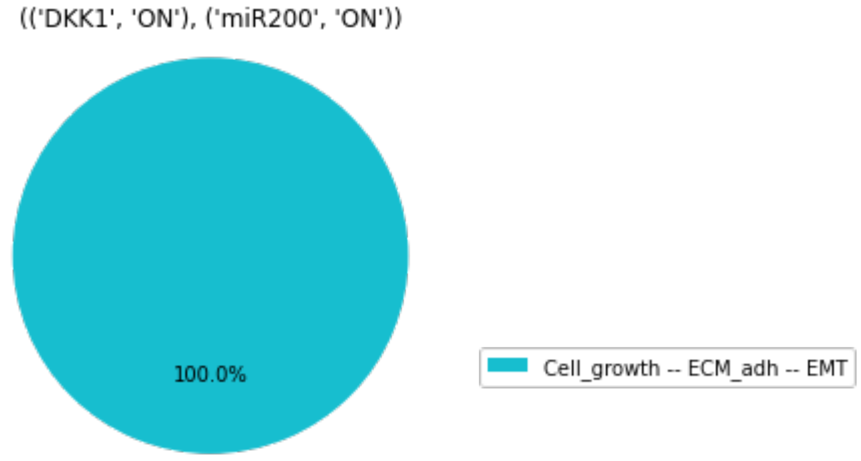
(('DKK1', 'ON'), ('miR200', 'ON'))
100.0%
Cell_growth -- ECM_adh -- EMT

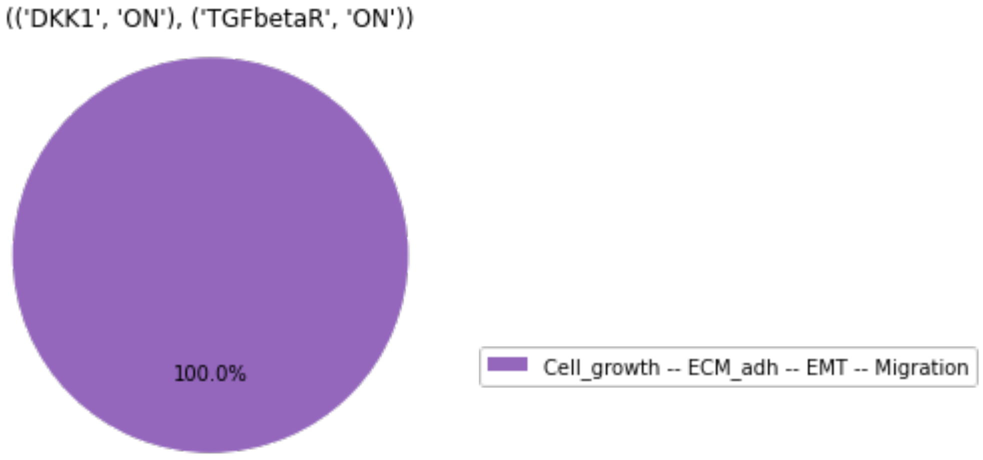
(('DKK1', 'ON'), ('TGFbetaR', 'ON'))
100.0%
Cell_growth -- ECM_adh -- EMT -- Migration

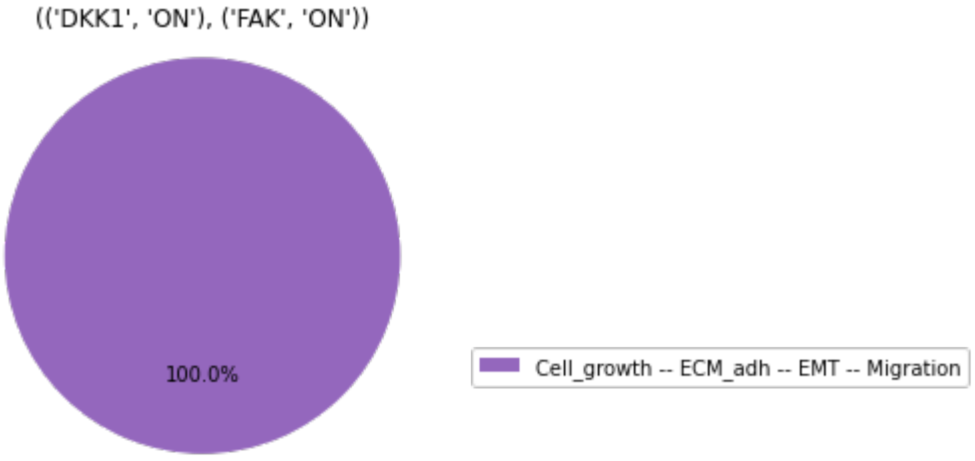
(('DKK1', 'ON'), ('FAK', 'ON'))
100.0%
Cell_growth -- ECM_adh -- EMT -- Migration

(('DKK1', 'ON'), ('AKT1', 'OFF'))

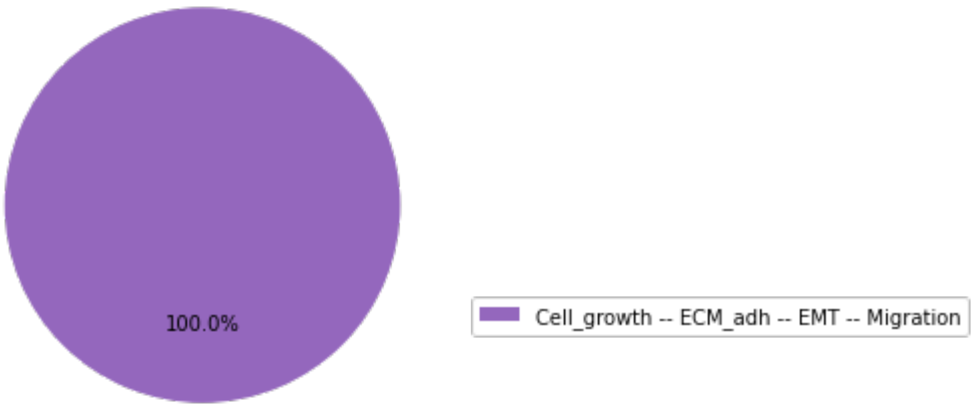

(('DKK1', 'ON'), ('CTNNB1', 'OFF'))

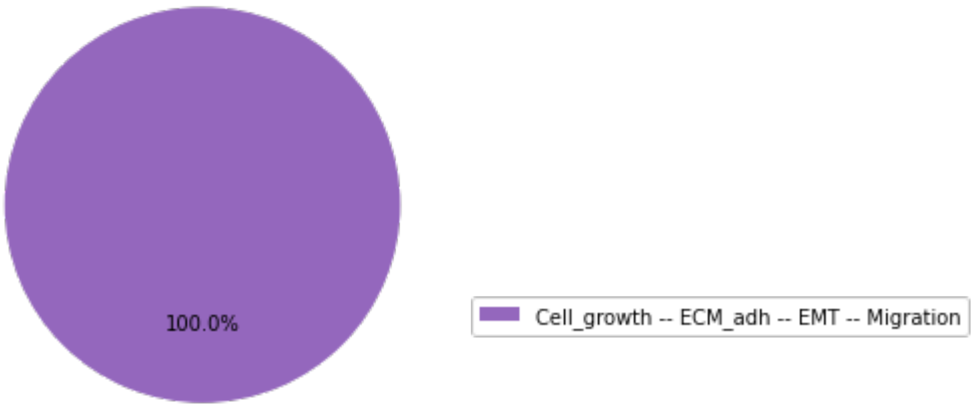

(('DKK1', 'ON'), ('AKT2', 'OFF'))

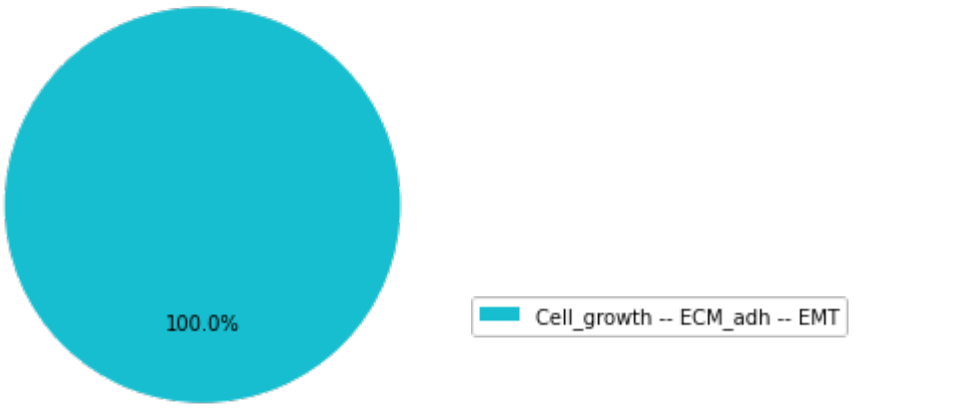

(('DKK1', 'ON'), ('RAC1', 'OFF'))

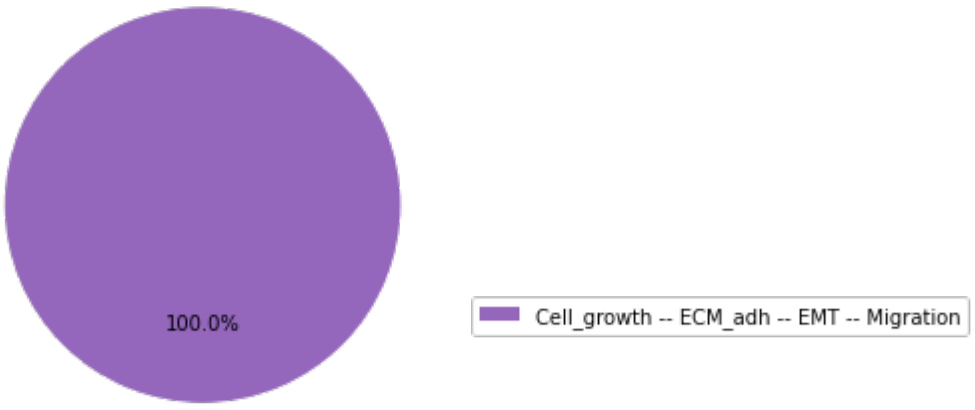

(('DKK1', 'ON'), ('YAP1', 'OFF'))

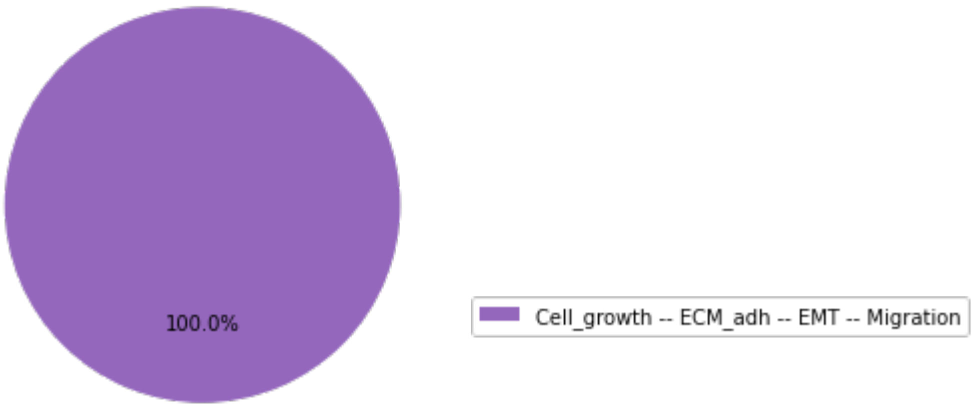

(('DKK1', 'ON'), ('PIK3CA', 'OFF'))

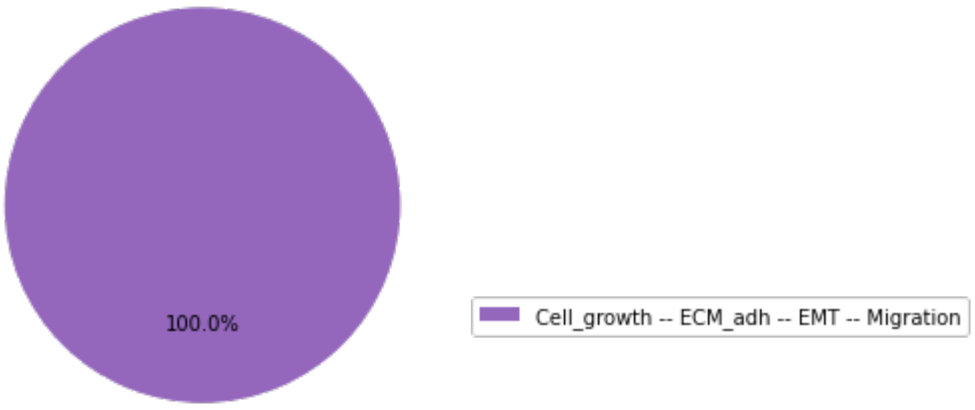

(('DKK1', 'ON'), ('ERK', 'OFF'))

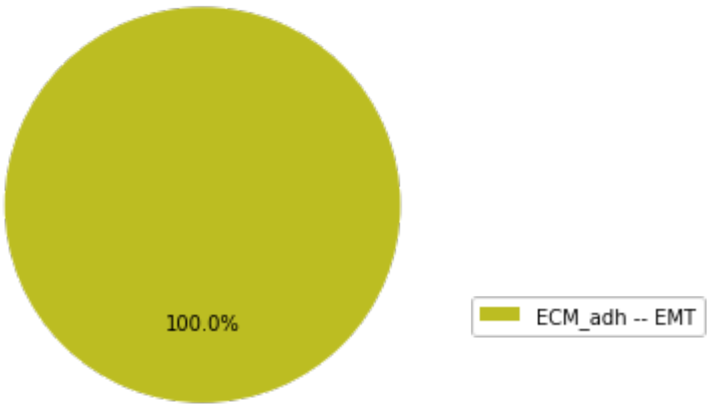

(('DKK1', 'ON'), ('p21', 'OFF'))

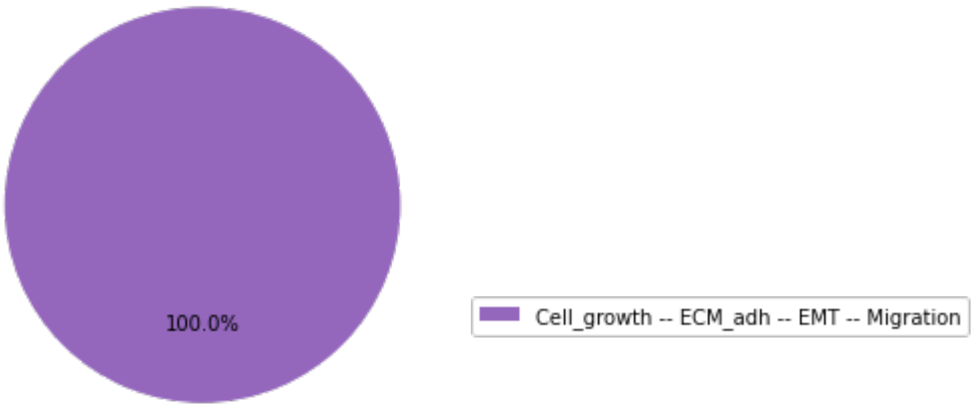

(('DKK1', 'ON'), ('SMAD', 'OFF'))

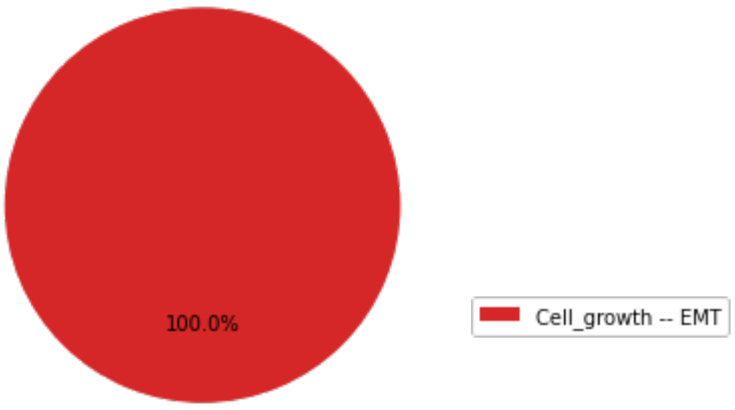

(('DKK1', 'ON'), ('VIM', 'OFF'))

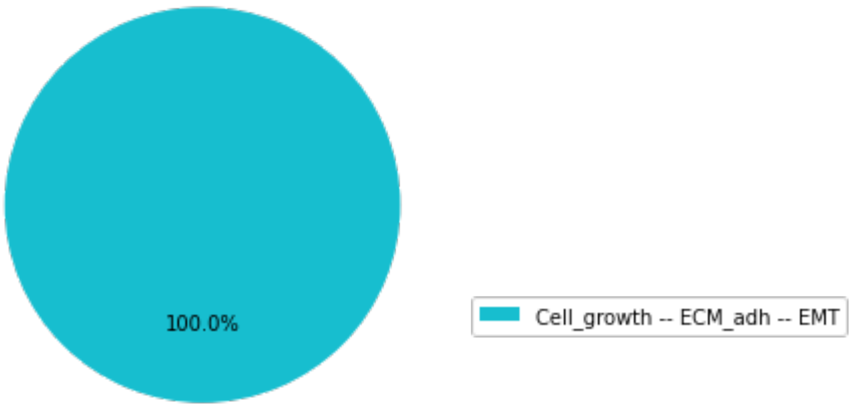

(('DKK1', 'ON'), ('p53', 'OFF'))

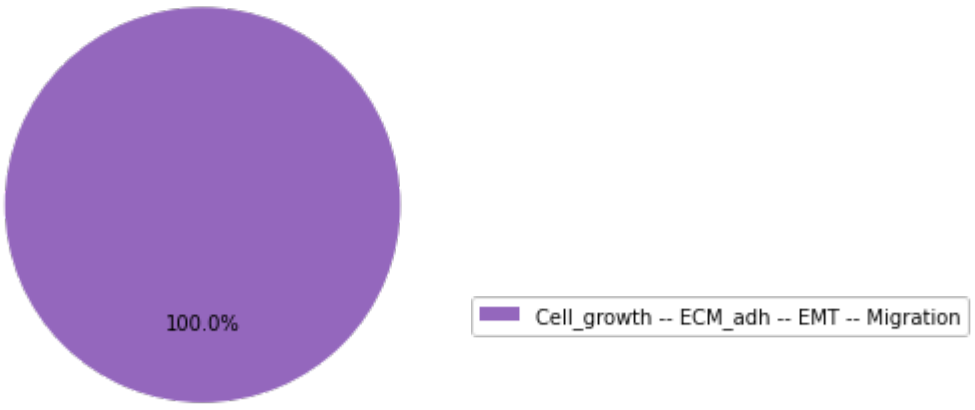

(('DKK1', 'ON'), ('p63', 'OFF'))

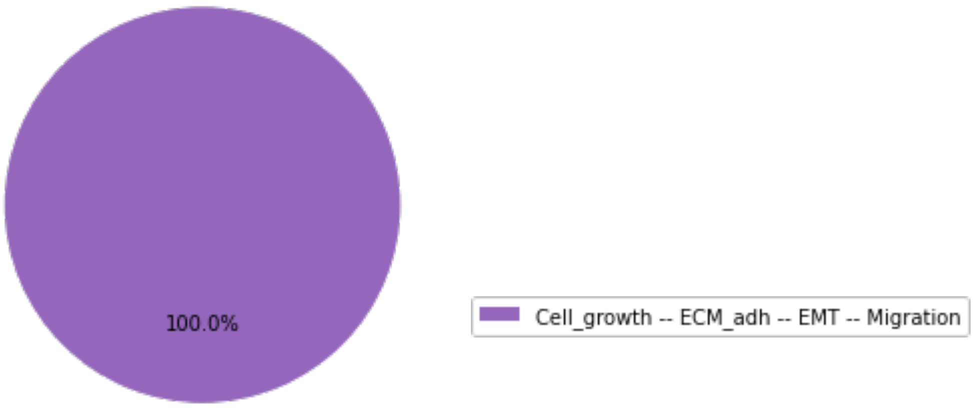

(('DKK1', 'ON'), ('p73', 'OFF'))

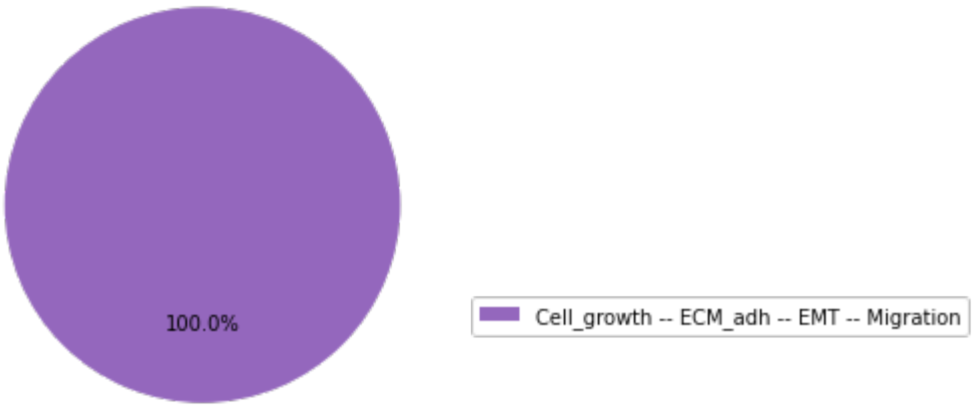

(('DKK1', 'ON'), ('miR203', 'OFF'))

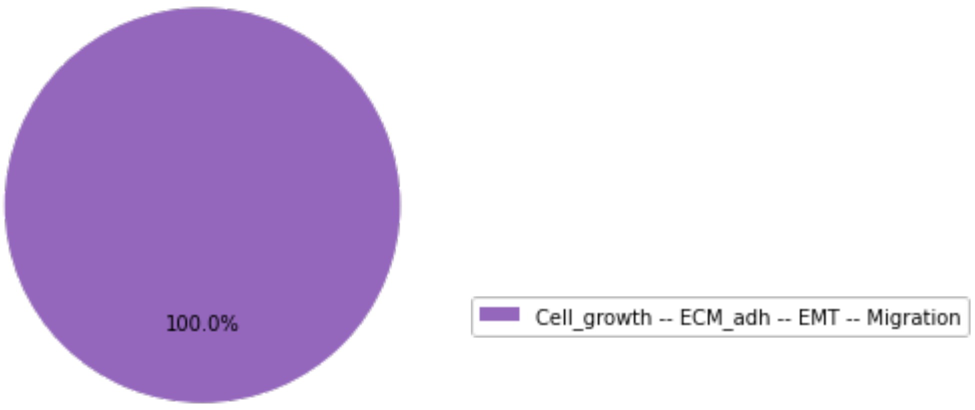

(('DKK1', 'ON'), ('miR34', 'OFF'))

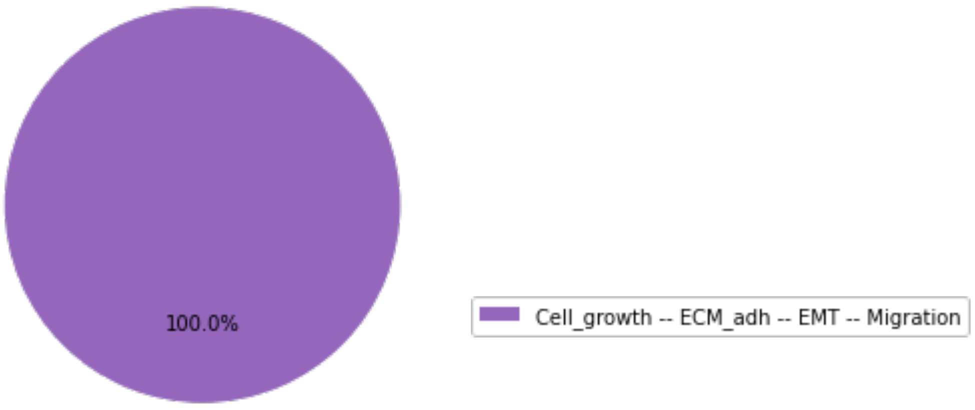

(('DKK1', 'ON'), ('miR200', 'OFF'))

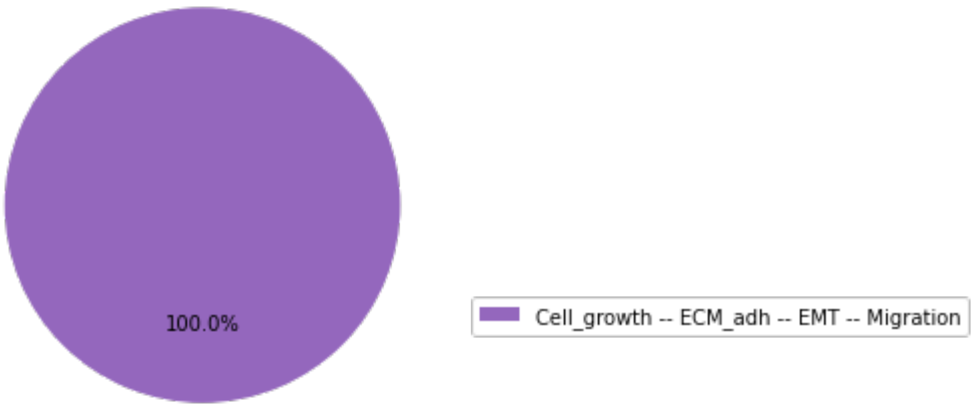

(('DKK1', 'ON'), ('TGFbetaR', 'OFF'))

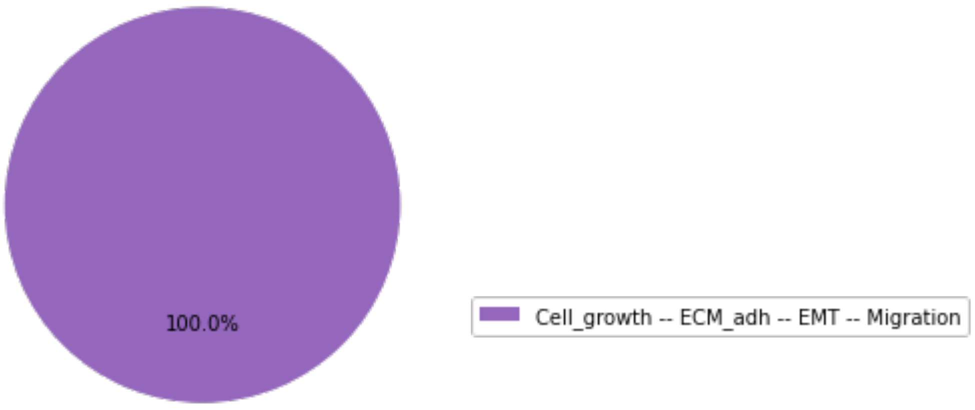

(('DKK1', 'ON'), ('FAK', 'OFF'))

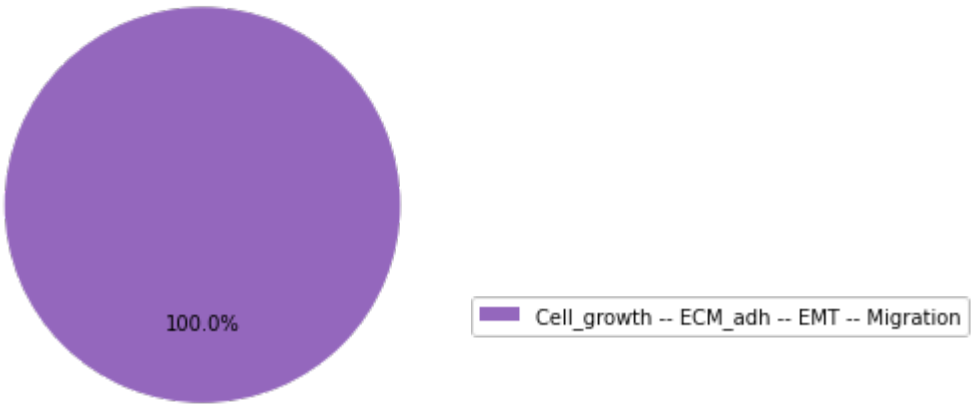

(('AKT2', 'ON'), ('RAC1', 'ON'))

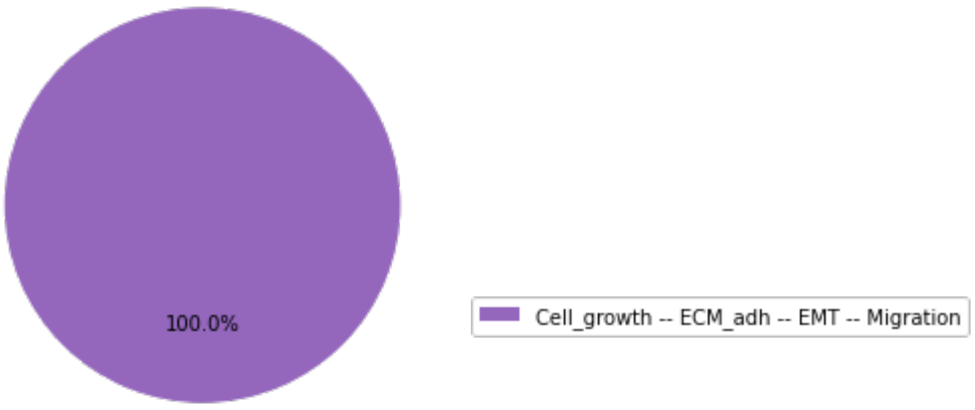

(('AKT2', 'ON'), ('YAP1', 'ON'))

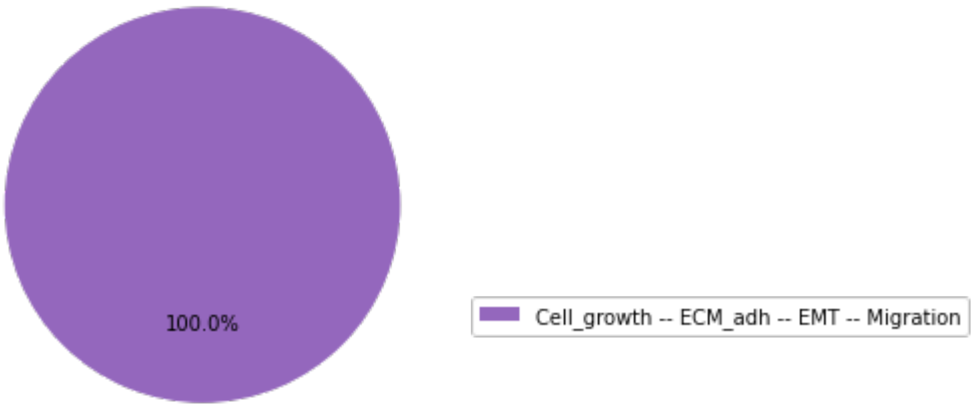

(('AKT2', 'ON'), ('PIK3CA', 'ON'))

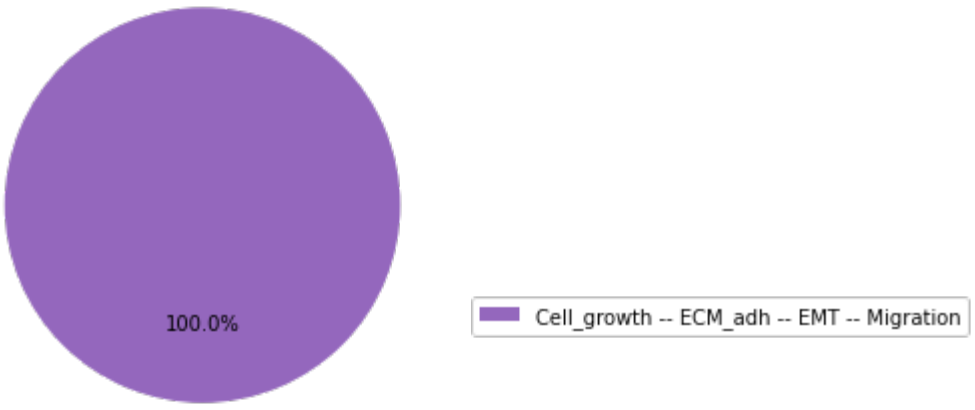

(('AKT2', 'ON'), ('ERK', 'ON'))

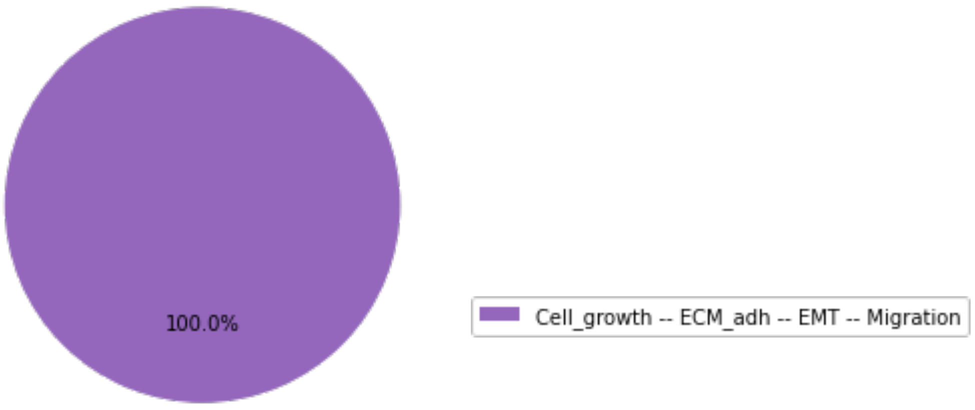

(('AKT2', 'ON'), ('p21', 'ON'))

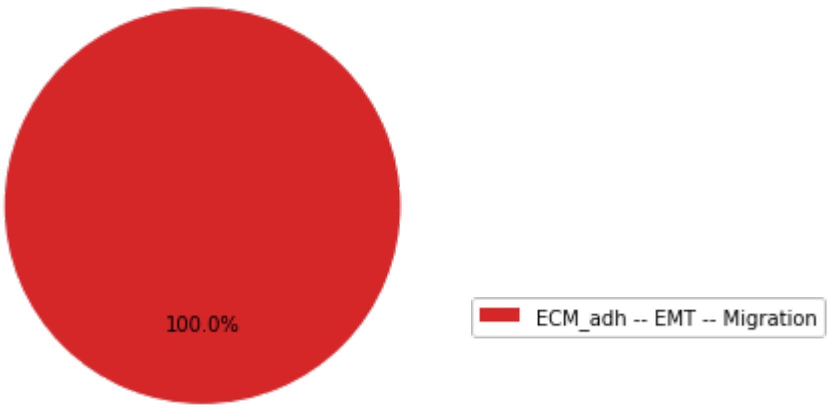

(('AKT2', 'ON'), ('SMAD', 'ON'))

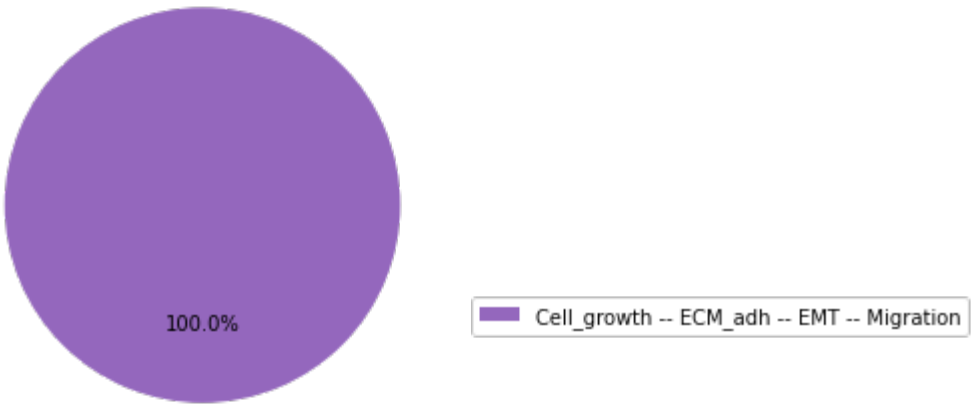

(('AKT2', 'ON'), ('VIM', 'ON'))

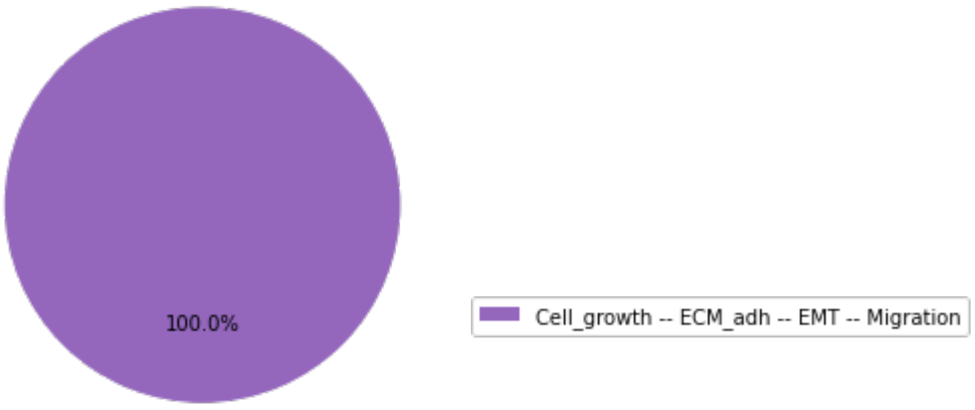

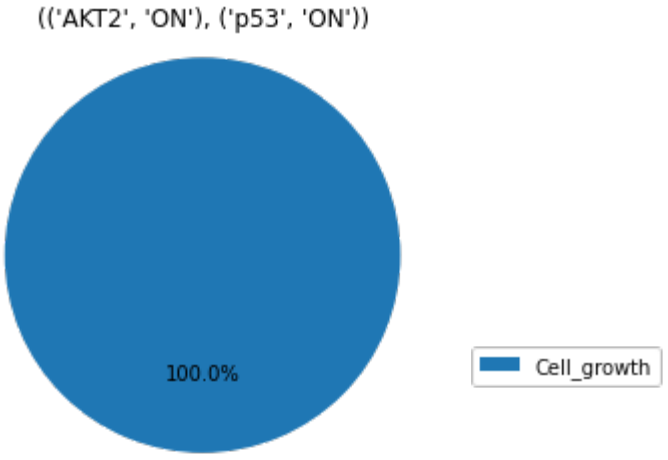
(('AKT2', 'ON'), ('p53', 'ON'))
100.0%
Cell_growth

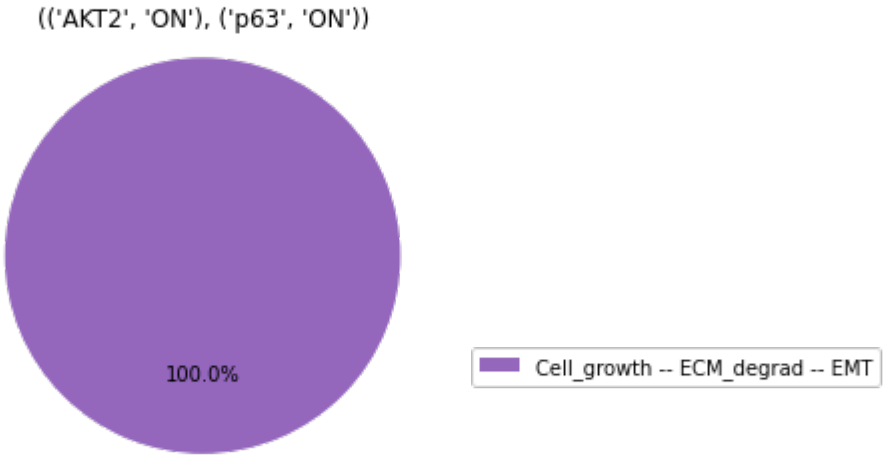
(('AKT2', 'ON'), ('p63', 'ON'))
100.0%
Cell_growth -- ECM_degrad -- EMT

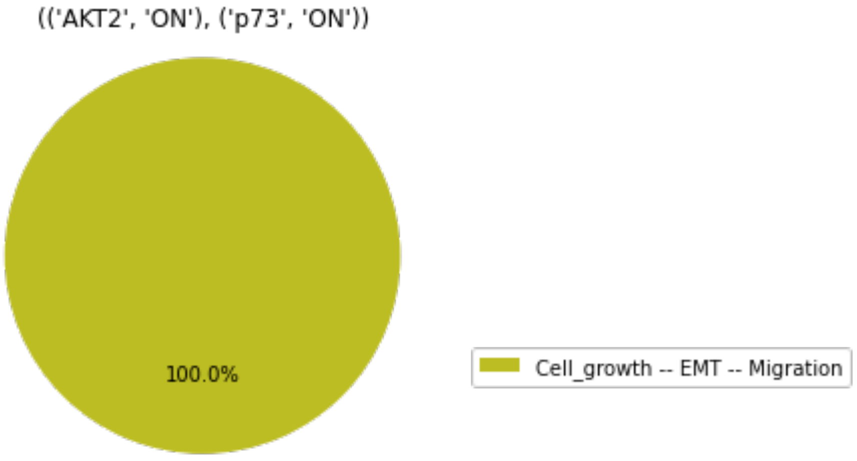
(('AKT2', 'ON'), ('p73', 'ON'))
100.0%
Cell_growth -- EMT -- Migration

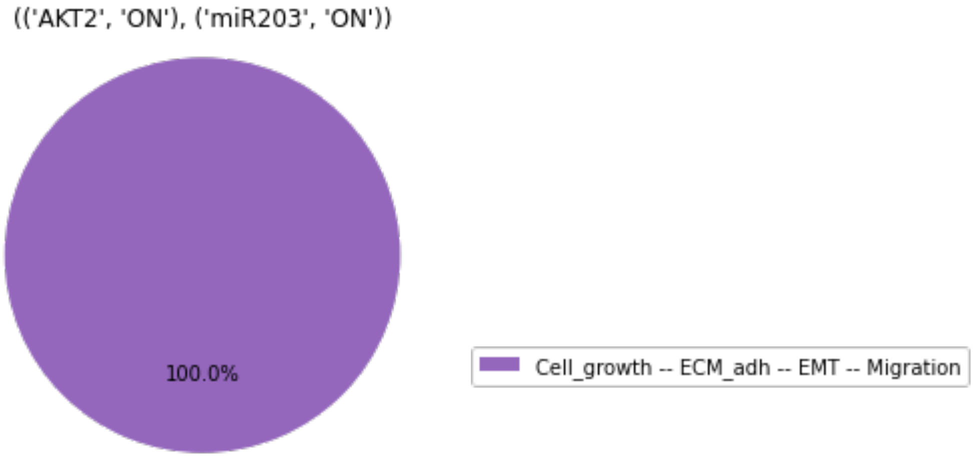
(('AKT2', 'ON'), ('miR203', 'ON'))
100.0%
Cell_growth -- ECM_adh -- EMT -- Migration

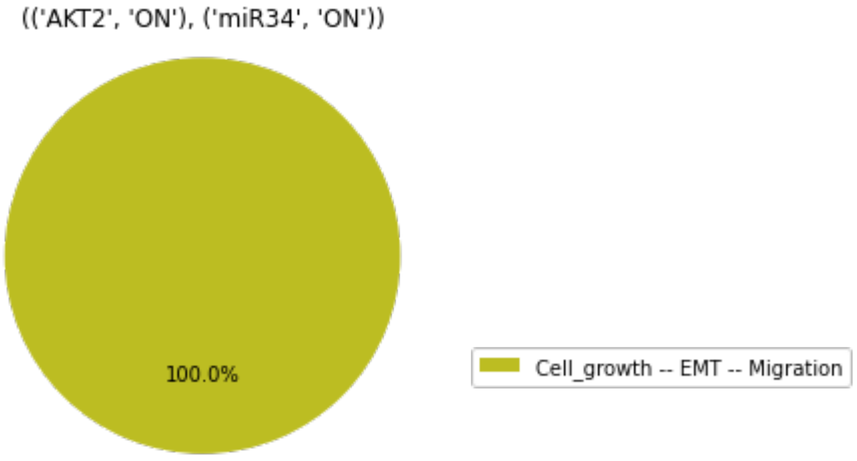
(('AKT2', 'ON'), ('miR34', 'ON'))
100.0%
Cell_growth -- EMT -- Migration

(('AKT2', 'ON'), ('miR200', 'ON'))

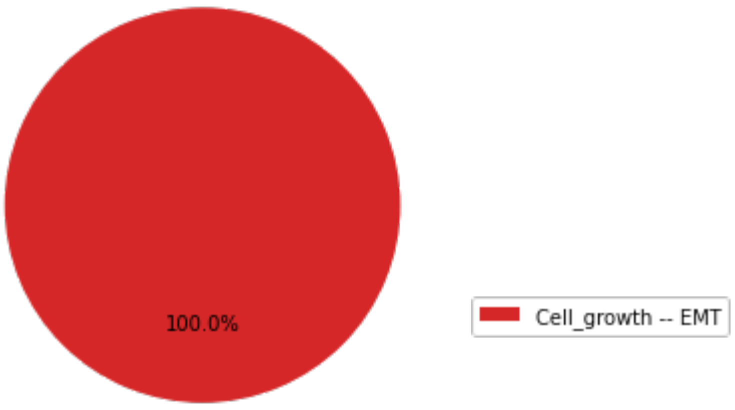

(('AKT2', 'ON'), ('TGFbetaR', 'ON'))

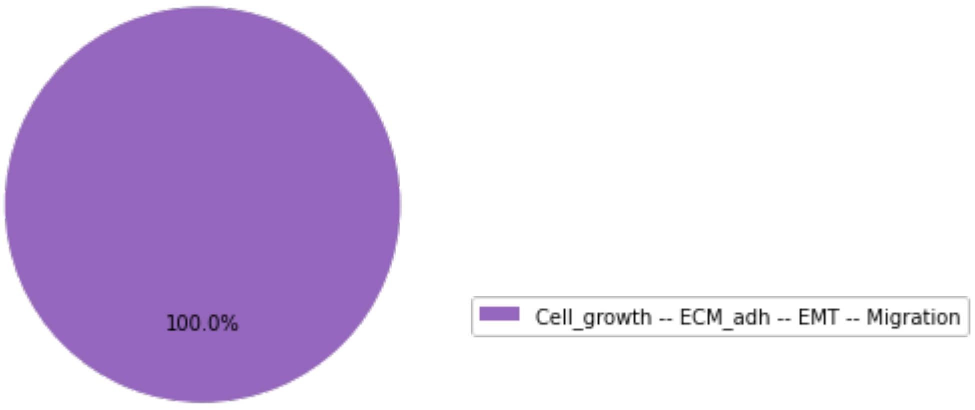

(('AKT2', 'ON'), ('FAK', 'ON'))

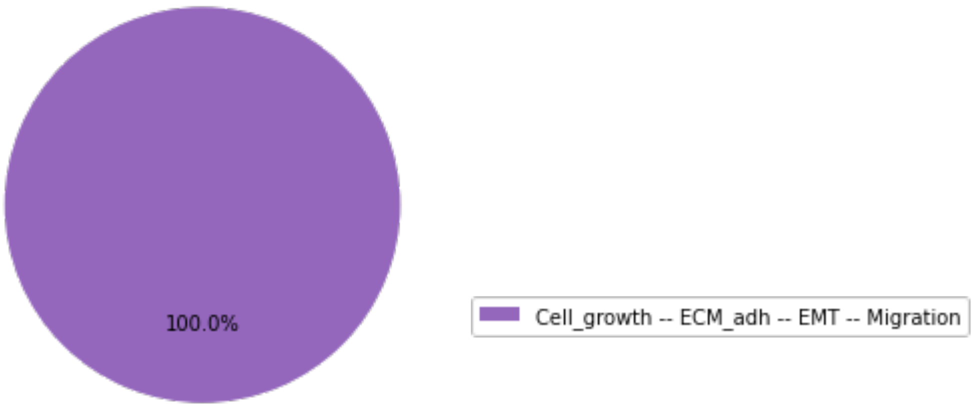

(('AKT2', 'ON'), ('AKT1', 'OFF'))

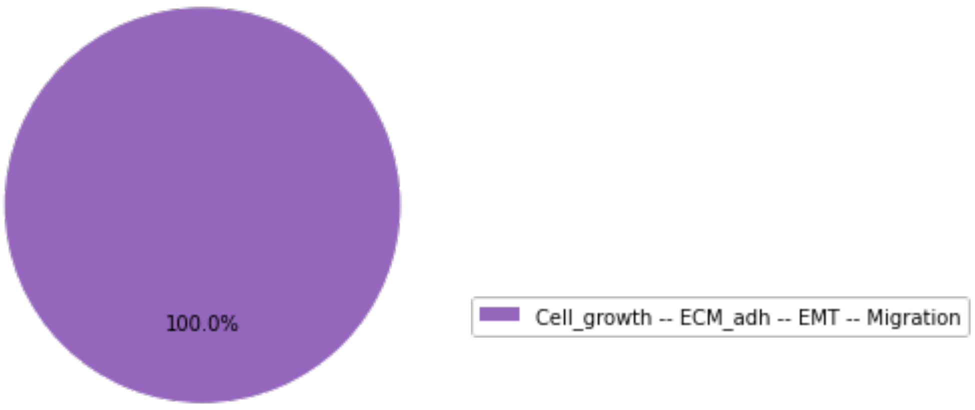

(('AKT2', 'ON'), ('CTNNB1', 'OFF'))

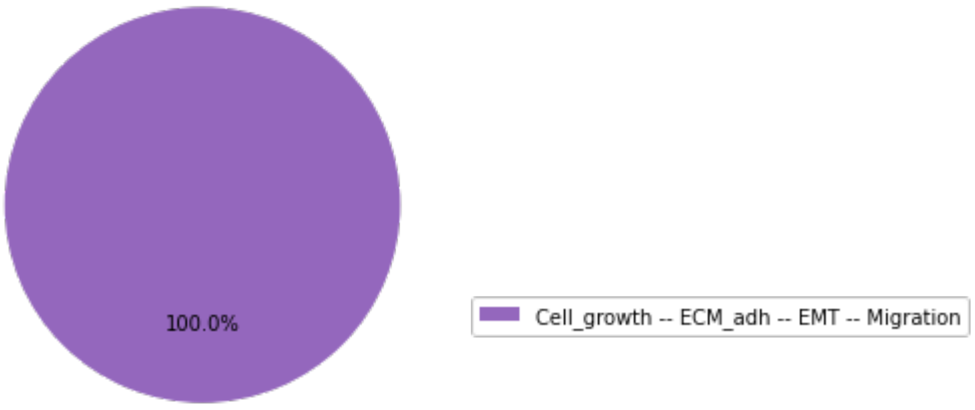

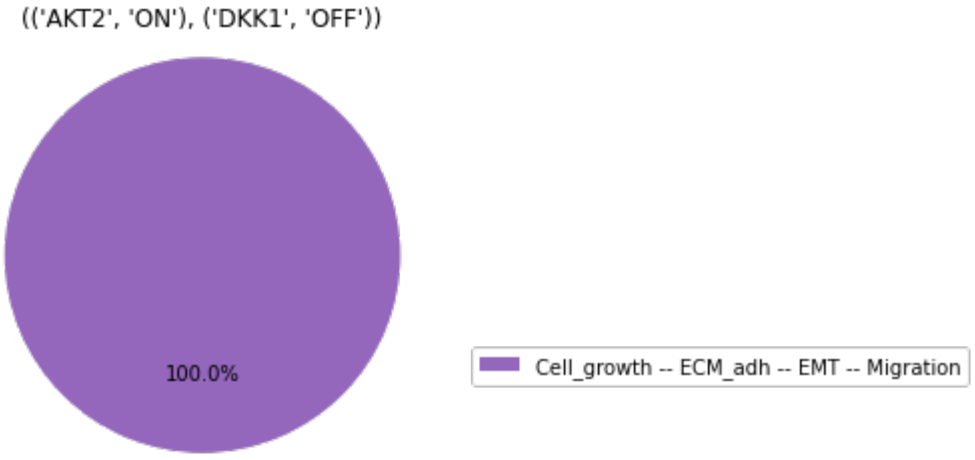
(('AKT2', 'ON'), ('DKK1', 'OFF'))
100.0%
Cell_growth -- ECM_adh -- EMT -- Migration

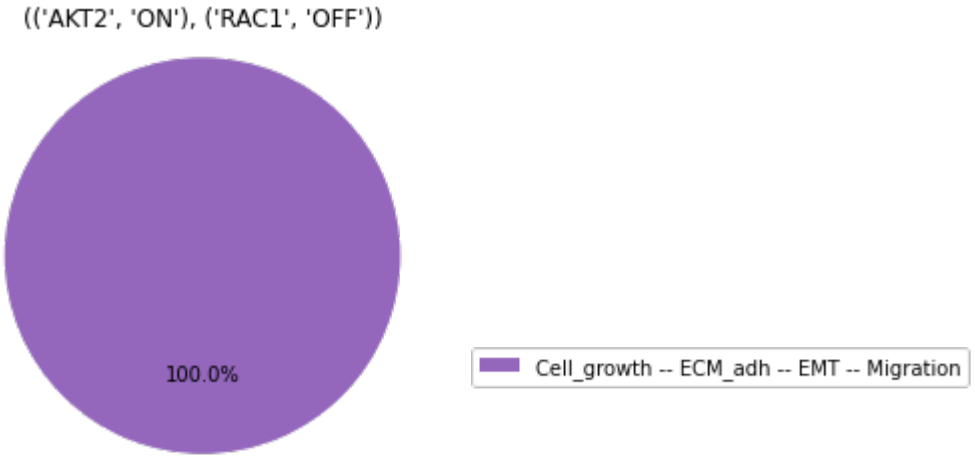
(('AKT2', 'ON'), ('RAC1', 'OFF'))
100.0%
Cell_growth -- ECM_adh -- EMT -- Migration

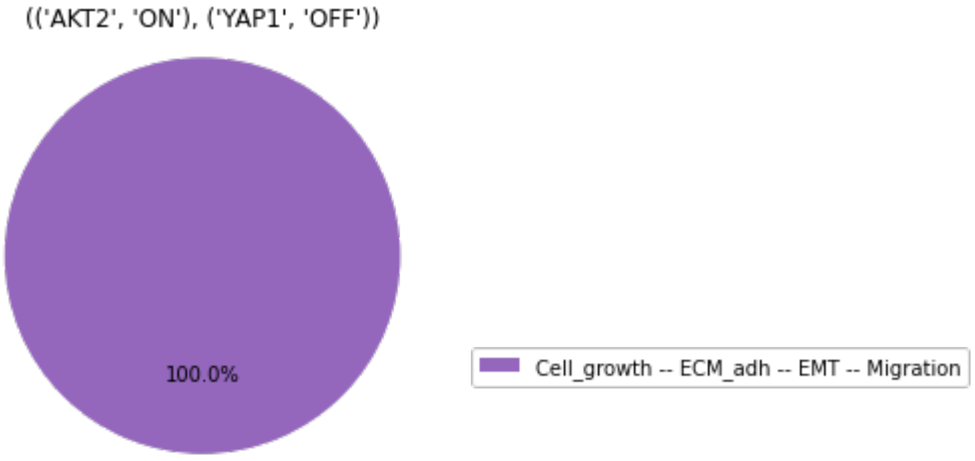
(('AKT2', 'ON'), ('YAP1', 'OFF'))
100.0%
Cell_growth -- ECM_adh -- EMT -- Migration

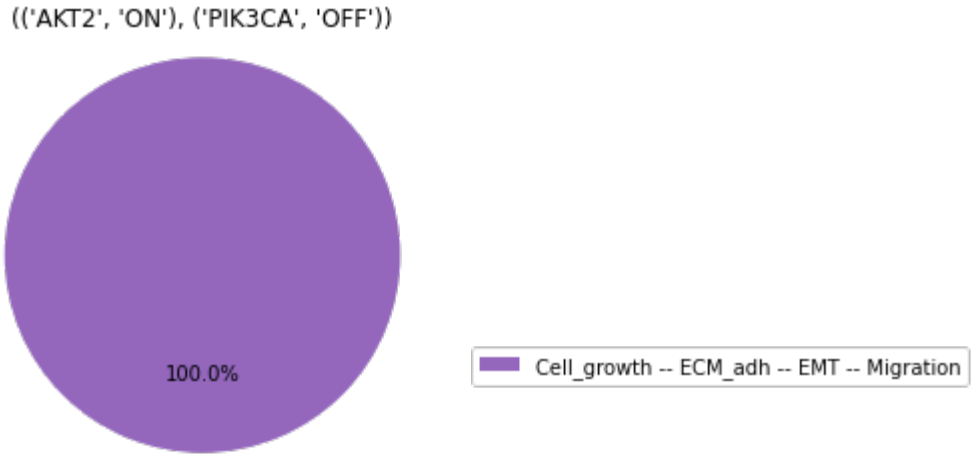
(('AKT2', 'ON'), ('PIK3CA', 'OFF'))
100.0%
Cell_growth -- ECM_adh -- EMT -- Migration

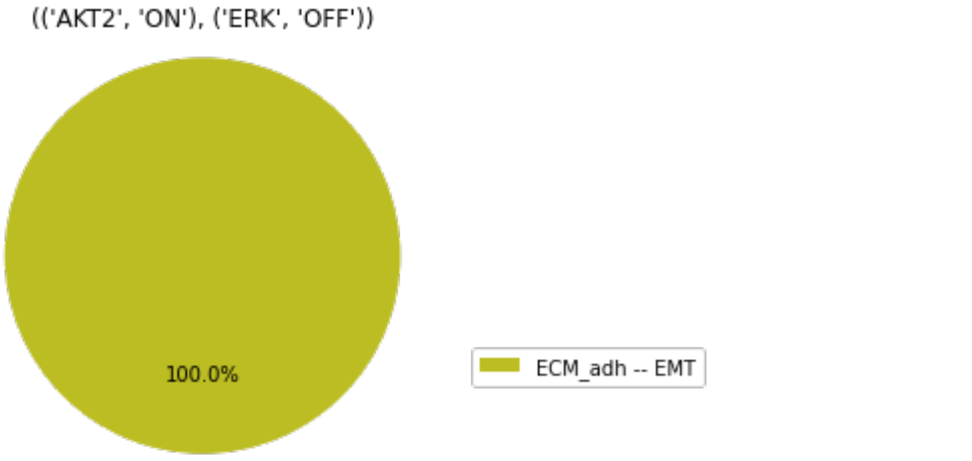
(('AKT2', 'ON'), ('ERK', 'OFF'))
100.0%
ECM_adh -- EMT

(('AKT2', 'ON'), ('p21', 'OFF'))

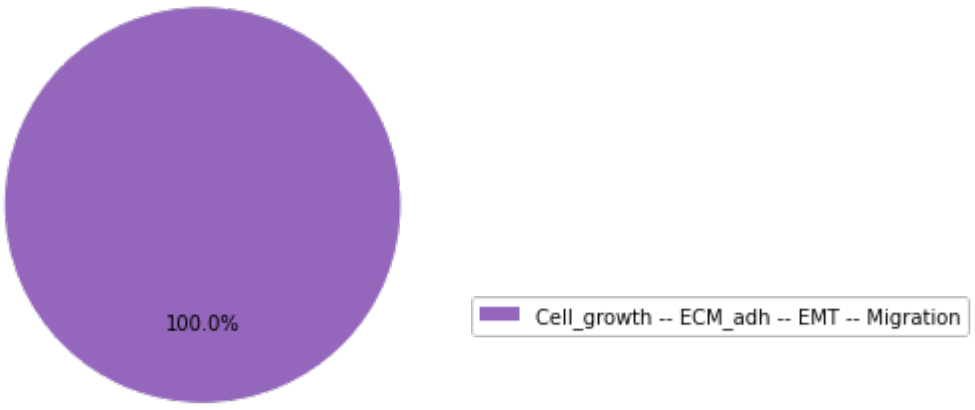

(('AKT2', 'ON'), ('SMAD', 'OFF'))

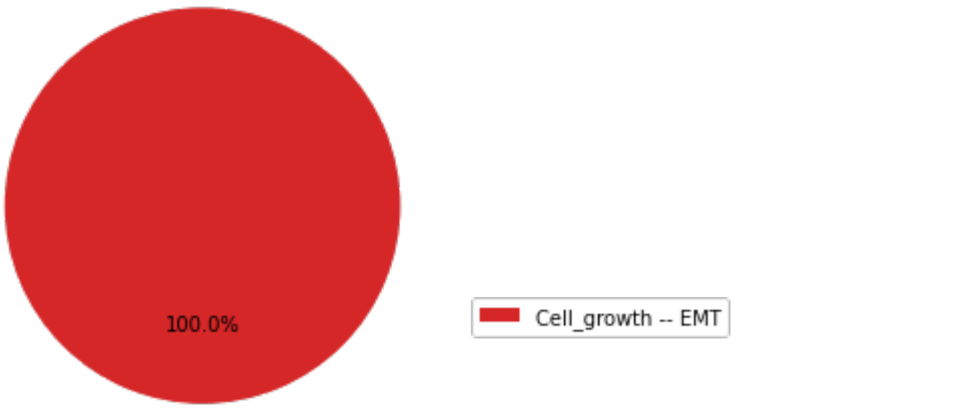

(('AKT2', 'ON'), ('VIM', 'OFF'))

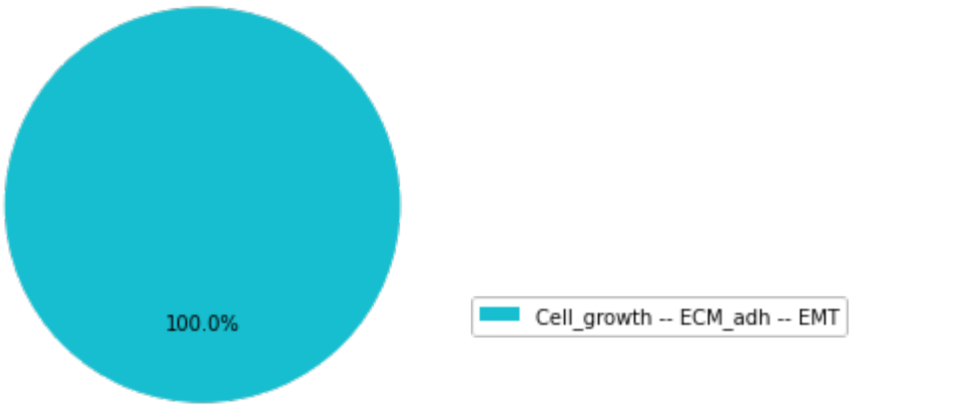

(('AKT2', 'ON'), ('p53', 'OFF'))

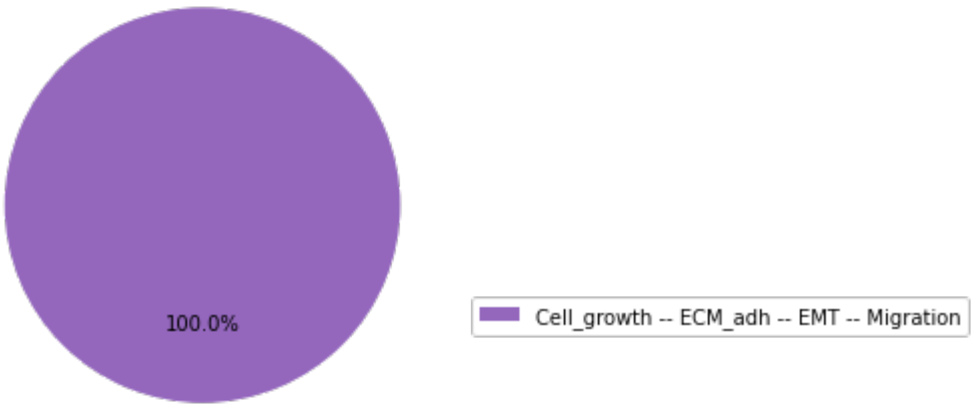

(('AKT2', 'ON'), ('p63', 'OFF'))

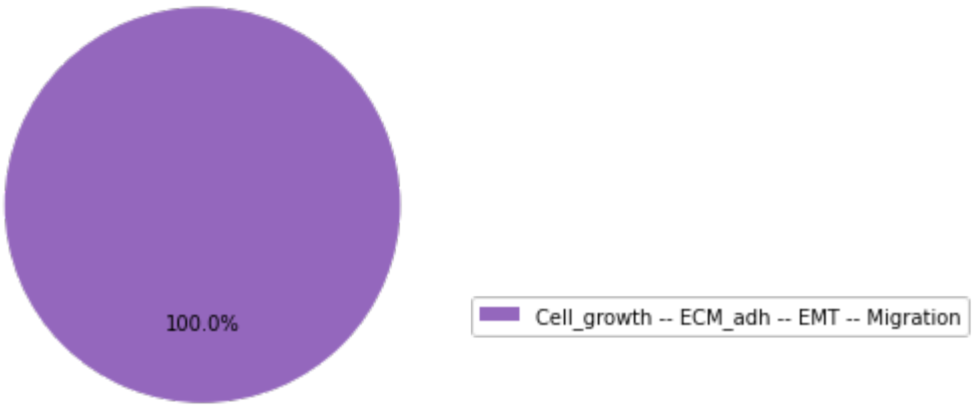

(('AKT2', 'ON'), ('p73', 'OFF'))

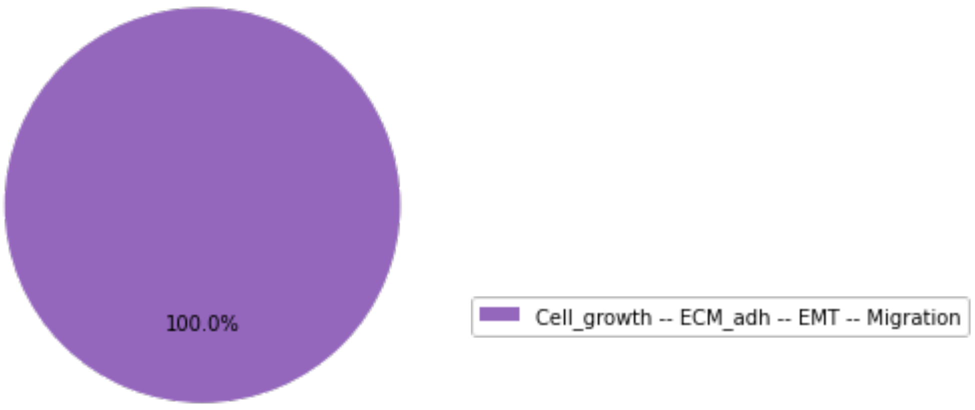

(('AKT2', 'ON'), ('miR203', 'OFF'))

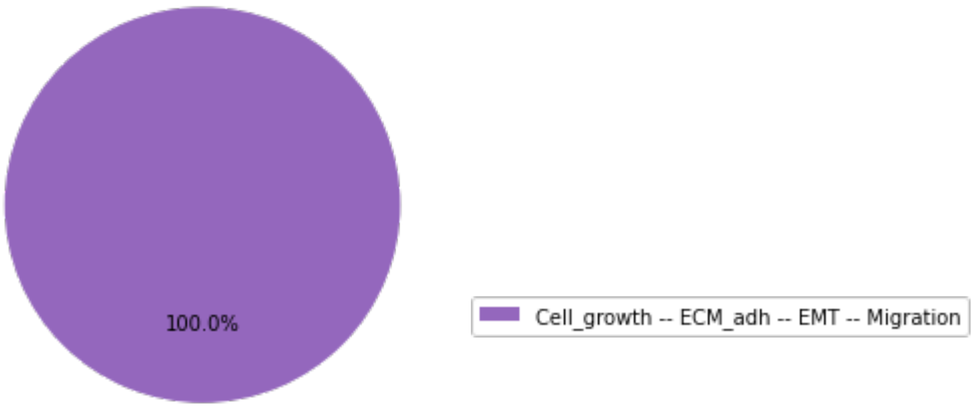

(('AKT2', 'ON'), ('miR34', 'OFF'))

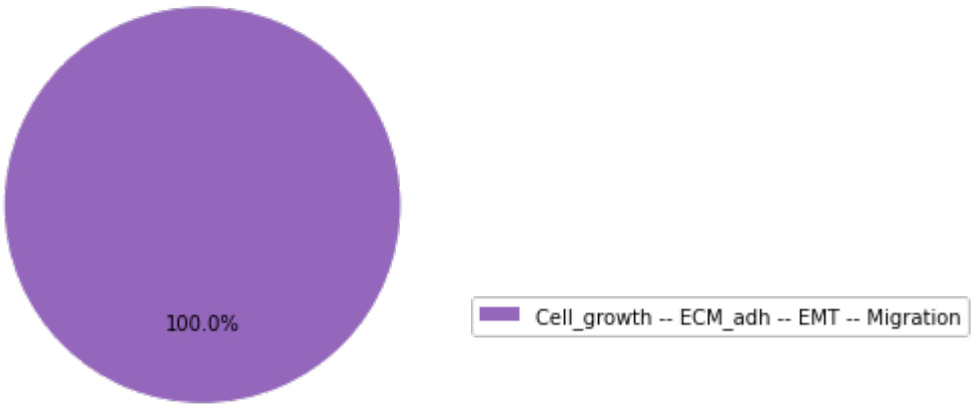

(('AKT2', 'ON'), ('miR200', 'OFF'))

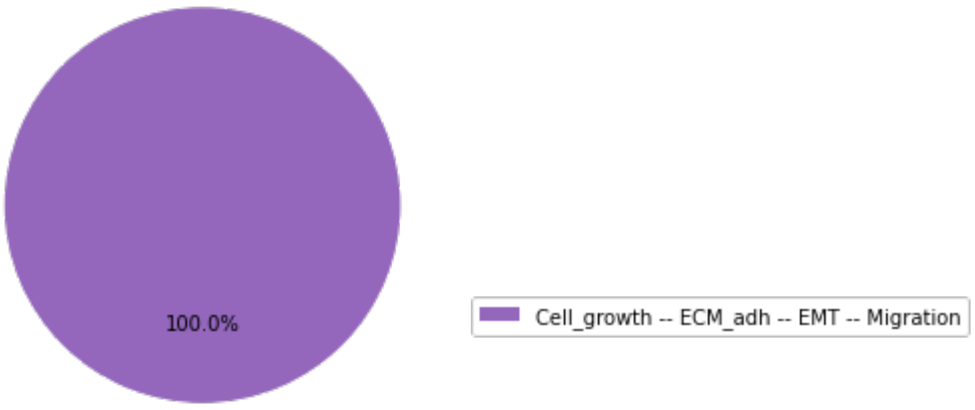

(('AKT2', 'ON'), ('TGFbetaR', 'OFF'))

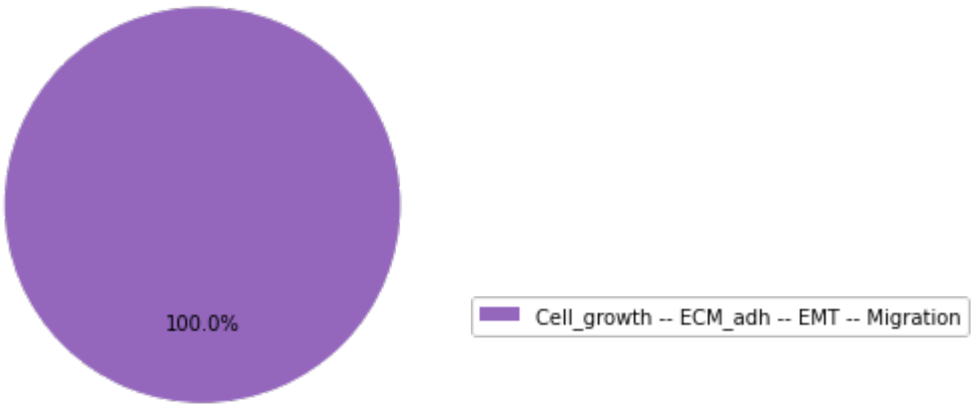

(('AKT2', 'ON'), ('FAK', 'OFF'))

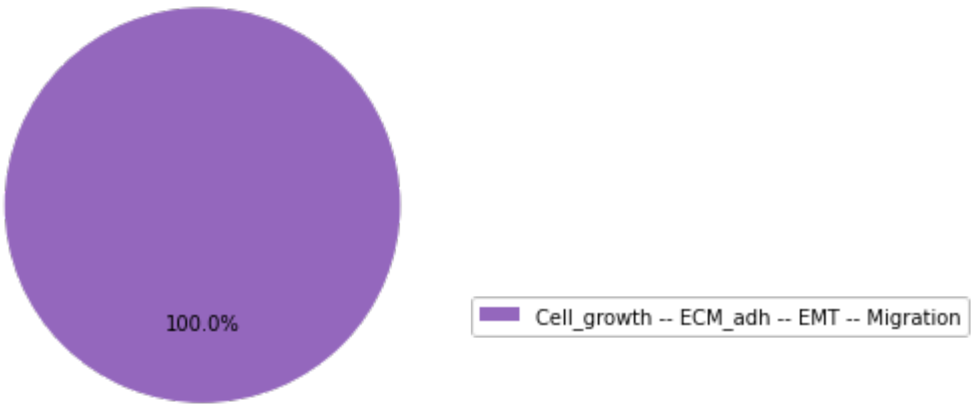

(('RAC1', 'ON'), ('YAP1', 'ON'))

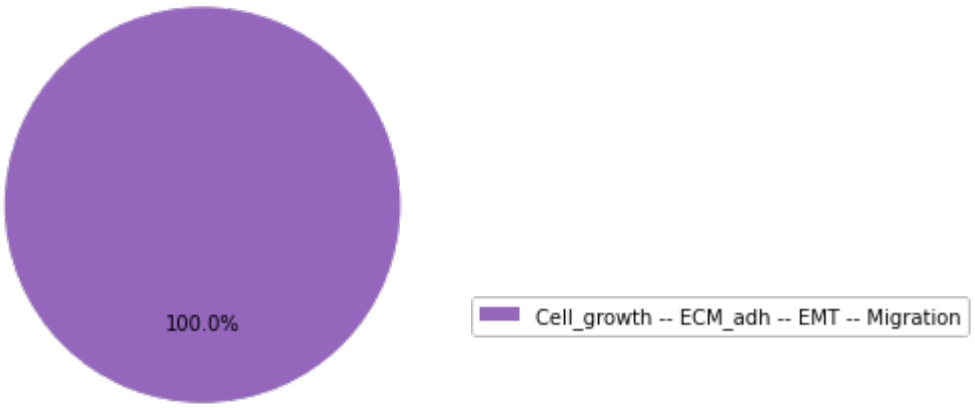

(('RAC1', 'ON'), ('PIK3CA', 'ON'))

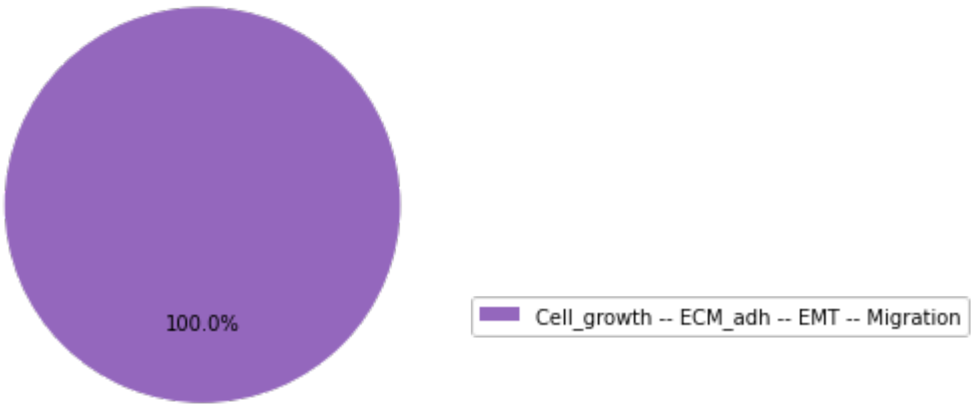

(('RAC1', 'ON'), ('ERK', 'ON'))

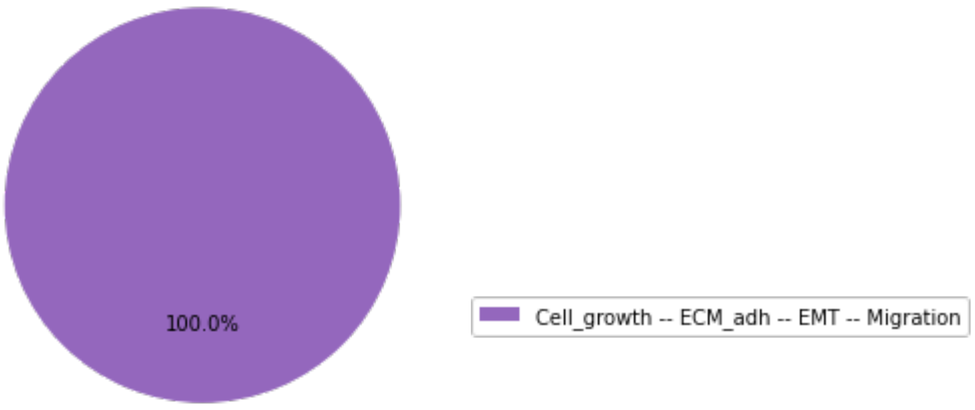

(('RAC1', 'ON'), ('p21', 'ON'))

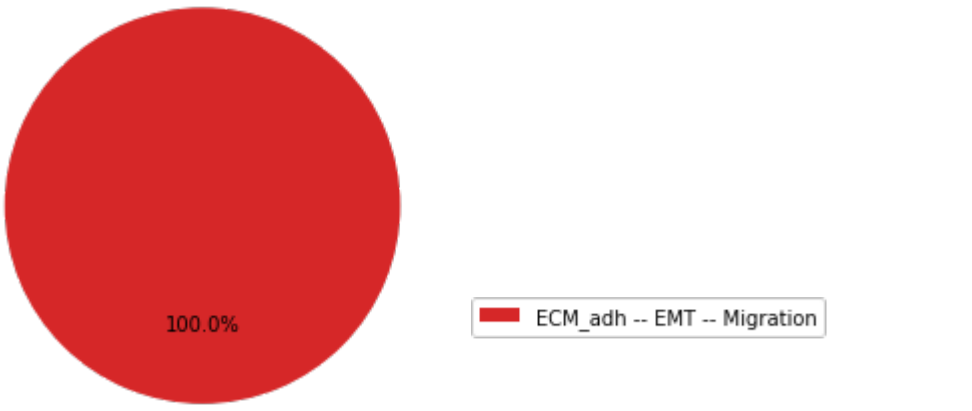

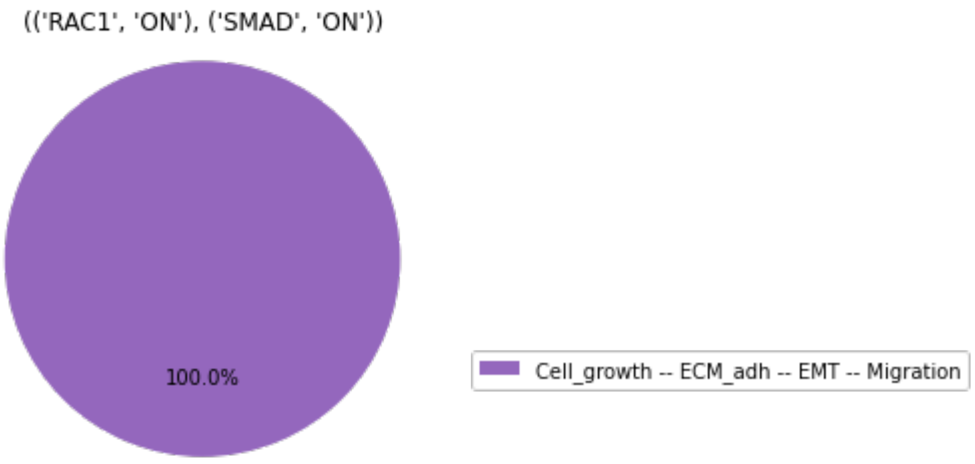
(('RAC1', 'ON'), ('SMAD', 'ON'))
100.0%
Cell_growth -- ECM_adh -- EMT -- Migration

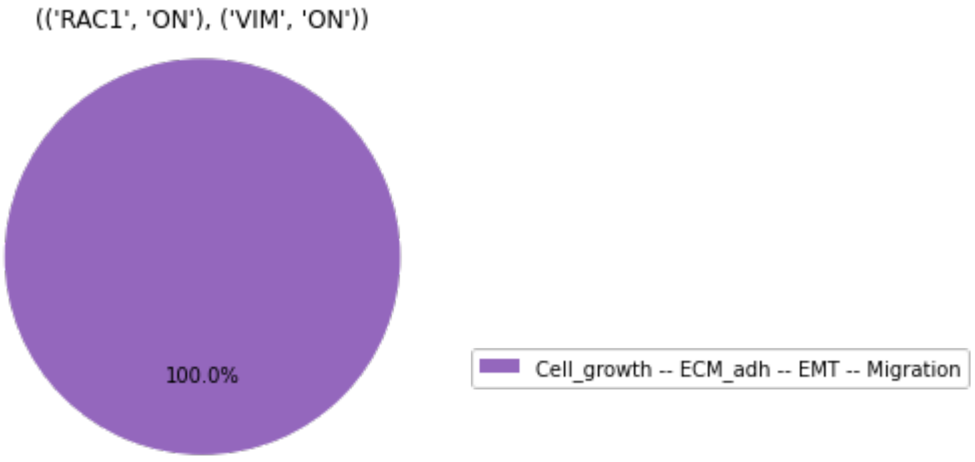
(('RAC1', 'ON'), ('VIM', 'ON'))
100.0%
Cell_growth -- ECM_adh -- EMT -- Migration

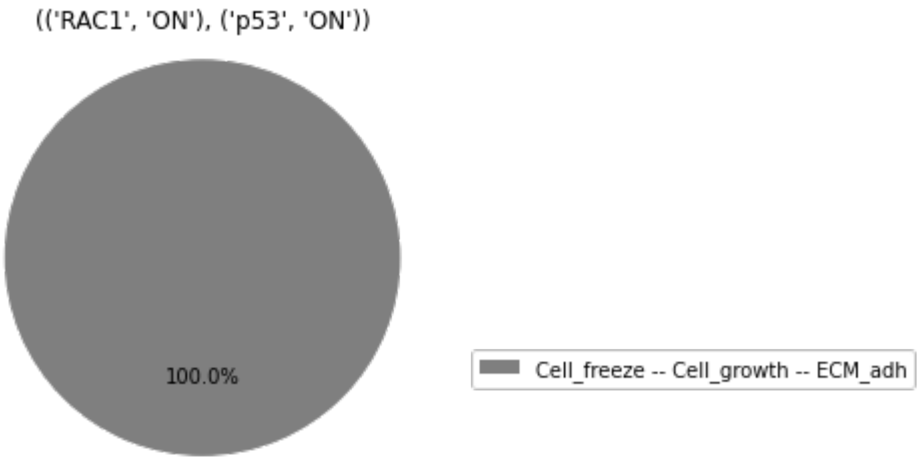
(('RAC1', 'ON'), ('p53', 'ON'))
100.0%
Cell_freeze -- Cell_growth -- ECM_adh

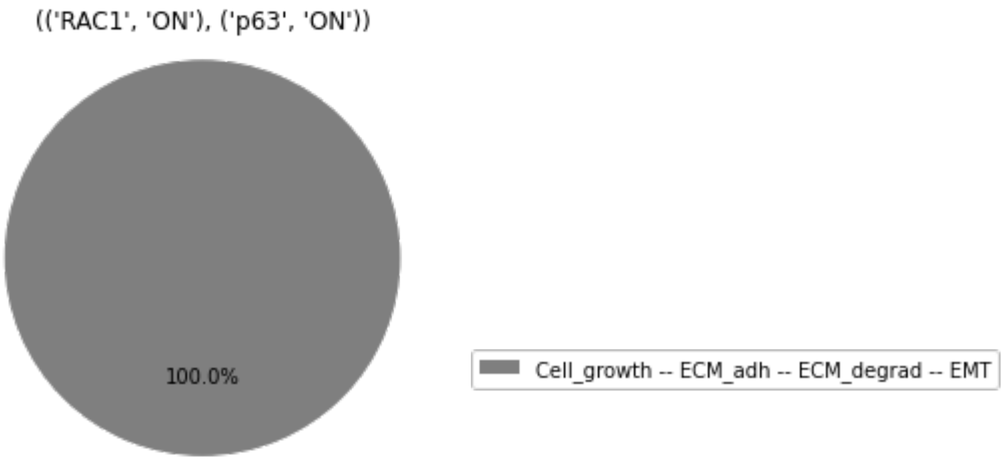
(('RAC1', 'ON'), ('p63', 'ON'))
100.0%
Cell_growth -- ECM_adh -- ECM_degrad -- EMT

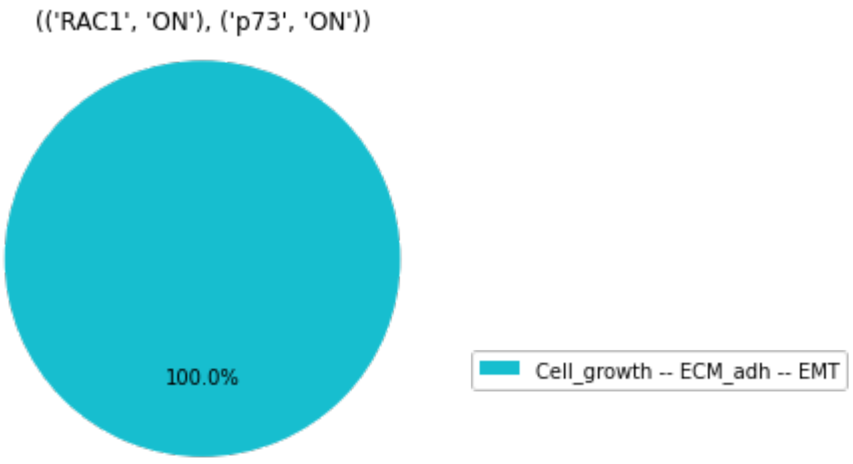
(('RAC1', 'ON'), ('p73', 'ON'))
100.0%
Cell_growth -- ECM_adh -- EMT

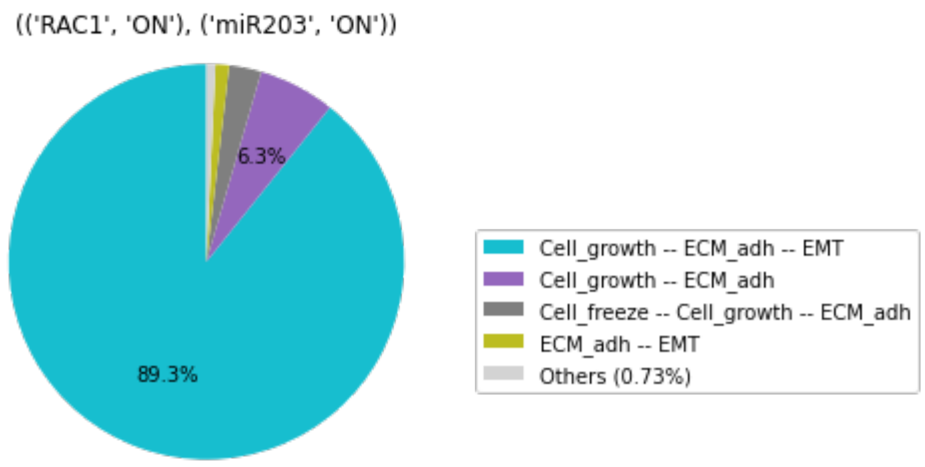

(('RAC1', 'ON'), ('miR203', 'ON'))
6.3%
89.3%
Cell_growth -- ECM_adh -- EMT
Cell_growth -- ECM_adh
Cell_freeze -- Cell_growth -- ECM_adh
ECM_adh -- EMT
Others (0.73%)

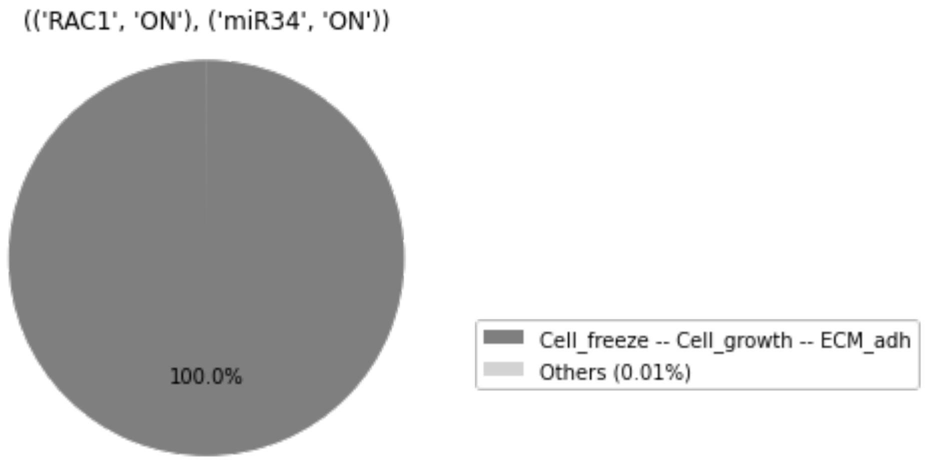

(('RAC1', 'ON'), ('miR34', 'ON'))
100.0%
Cell_freeze -- Cell_growth -- ECM_adh
Others (0.01%)

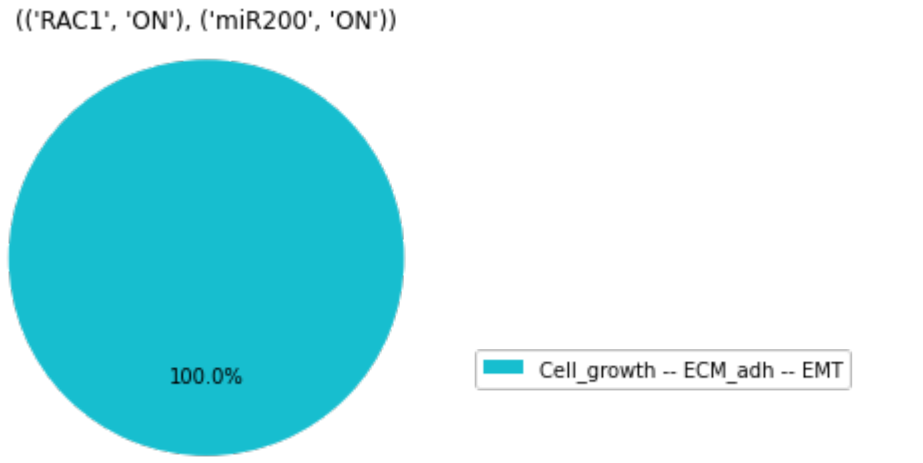

(('RAC1', 'ON'), ('miR200', 'ON'))
100.0%
Cell_growth -- ECM_adh -- EMT

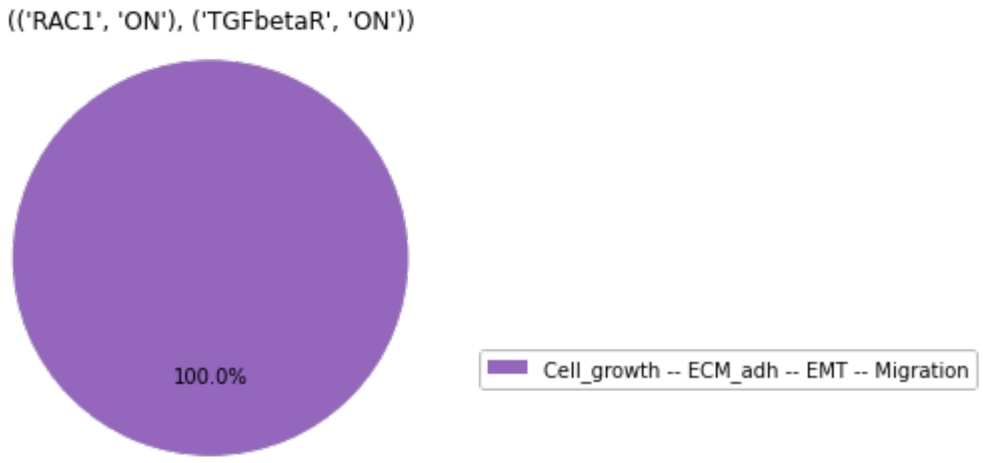

(('RAC1', 'ON'), ('TGFbetaR', 'ON'))
100.0%
Cell_growth -- ECM_adh -- EMT -- Migration

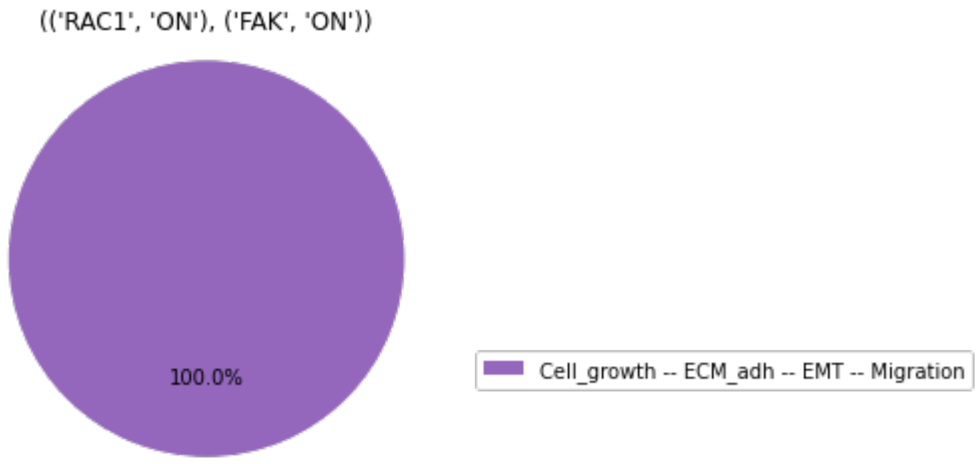

(('RAC1', 'ON'), ('FAK', 'ON'))
100.0%
Cell_growth -- ECM_adh -- EMT -- Migration

(('RAC1', 'ON'), ('AKT1', 'OFF'))

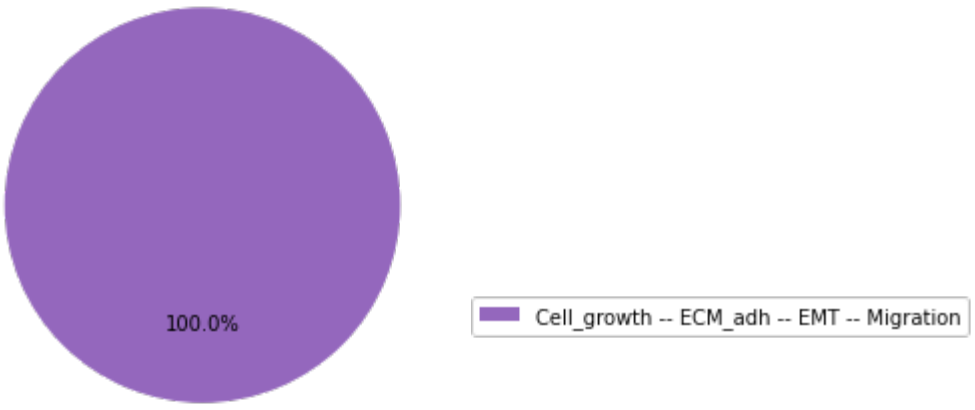

(('RAC1', 'ON'), ('CTNNB1', 'OFF'))

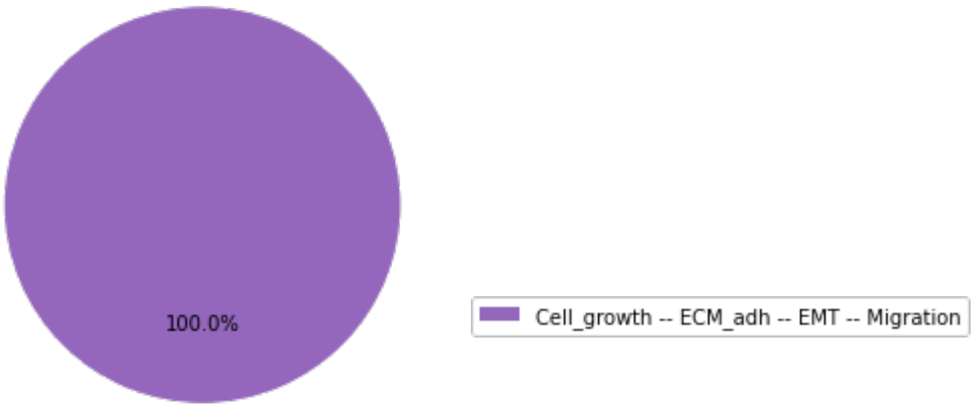

(('RAC1', 'ON'), ('DKK1', 'OFF'))

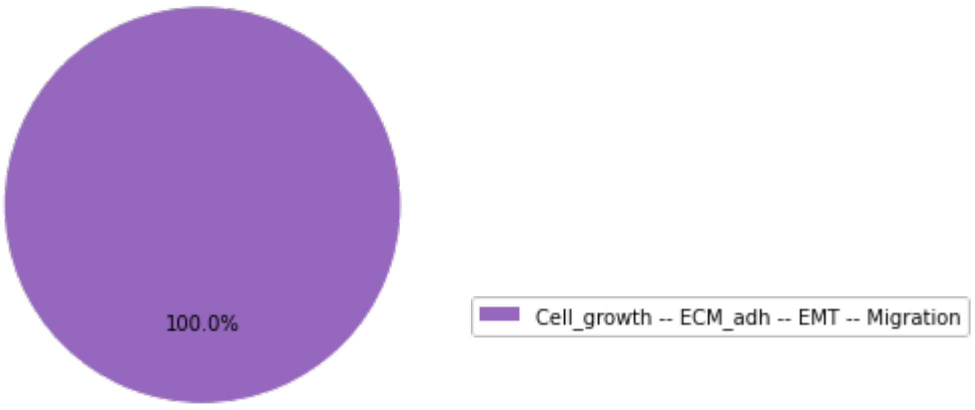

(('RAC1', 'ON'), ('AKT2', 'OFF'))

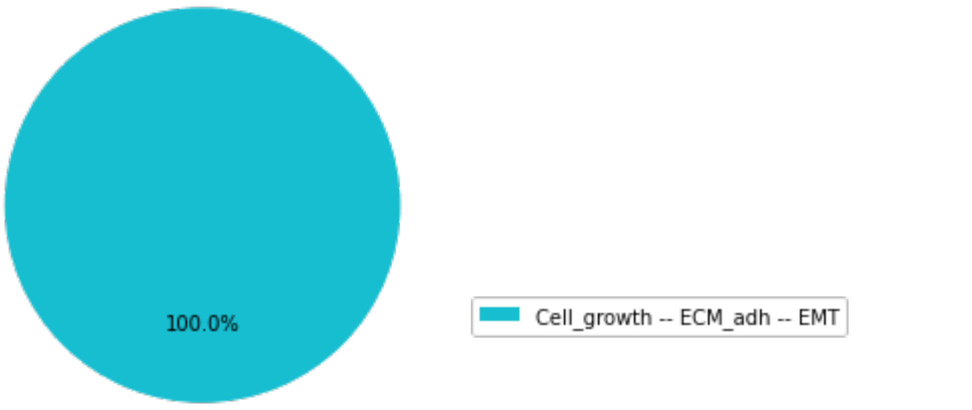

(('RAC1', 'ON'), ('YAP1', 'OFF'))

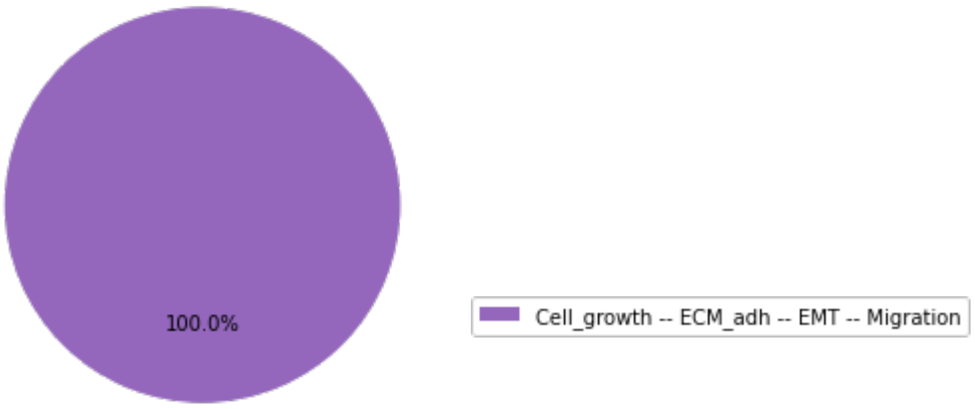

(('RAC1', 'ON'), ('PIK3CA', 'OFF'))

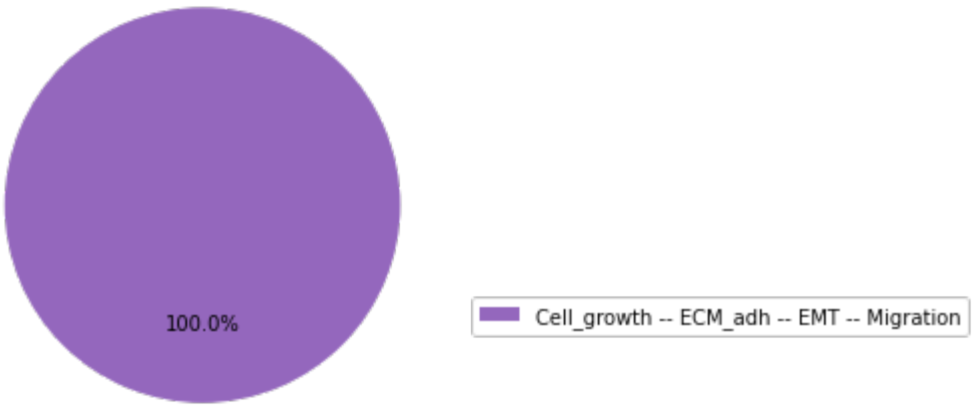

(('RAC1', 'ON'), ('ERK', 'OFF'))

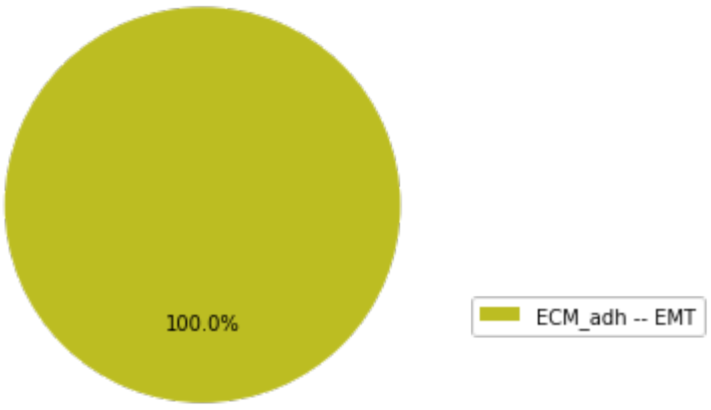

(('RAC1', 'ON'), ('p21', 'OFF'))

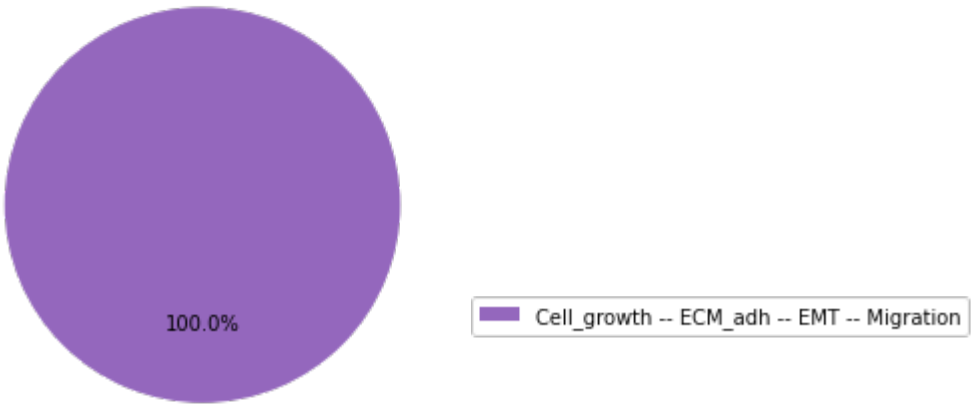

(('RAC1', 'ON'), ('SMAD', 'OFF'))

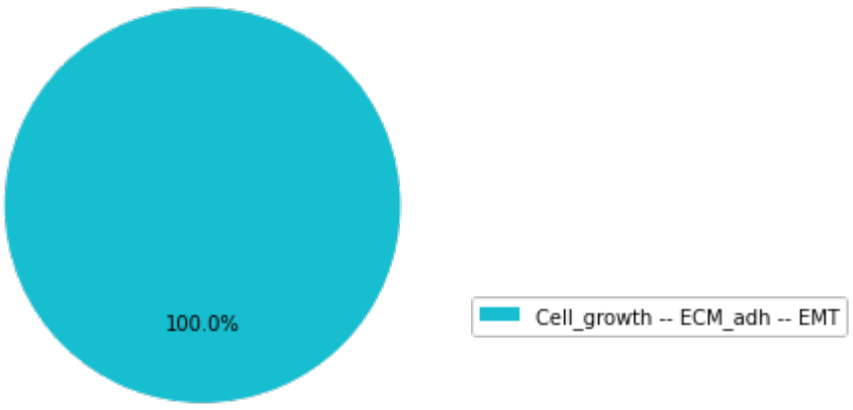

(('RAC1', 'ON'), ('VIM', 'OFF'))

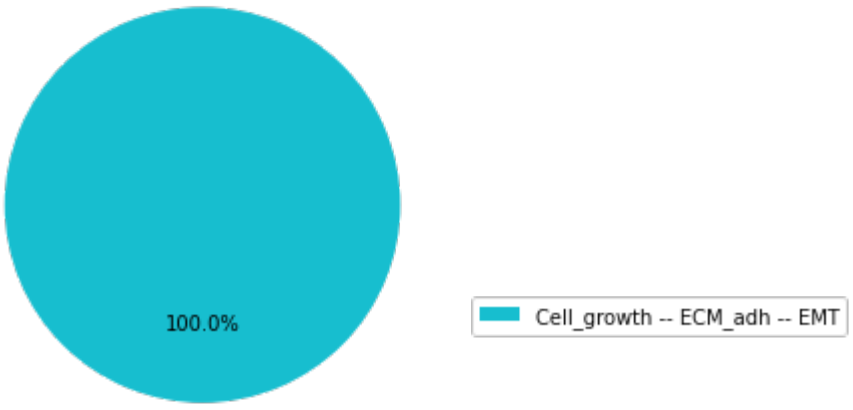

(('RAC1', 'ON'), ('p53', 'OFF'))

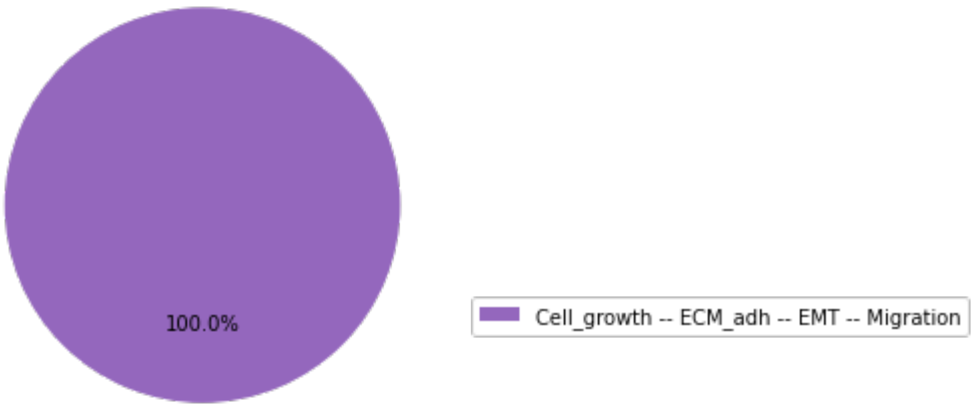

(('RAC1', 'ON'), ('p63', 'OFF'))

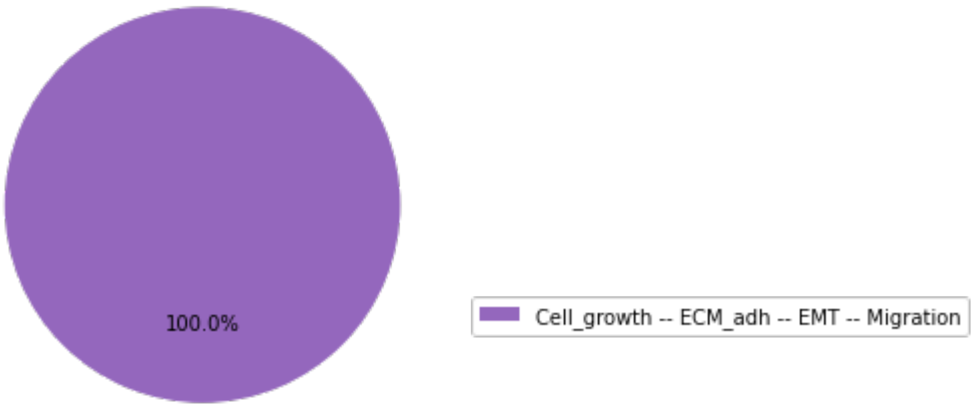

(('RAC1', 'ON'), ('p73', 'OFF'))

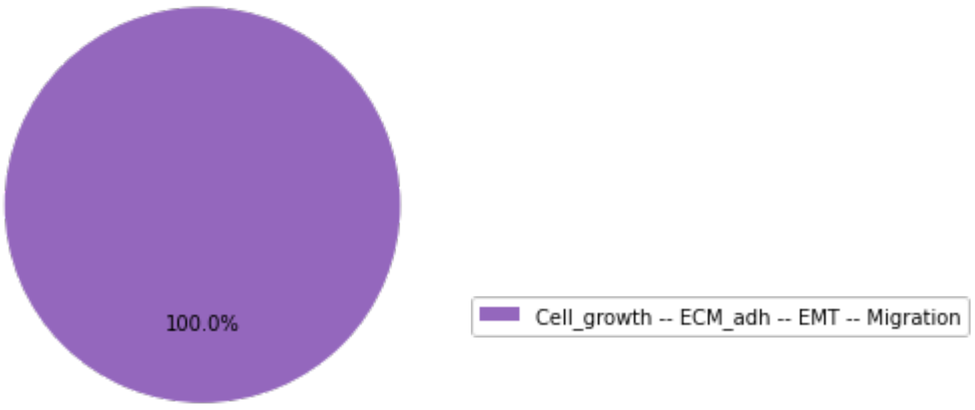

(('RAC1', 'ON'), ('miR203', 'OFF'))

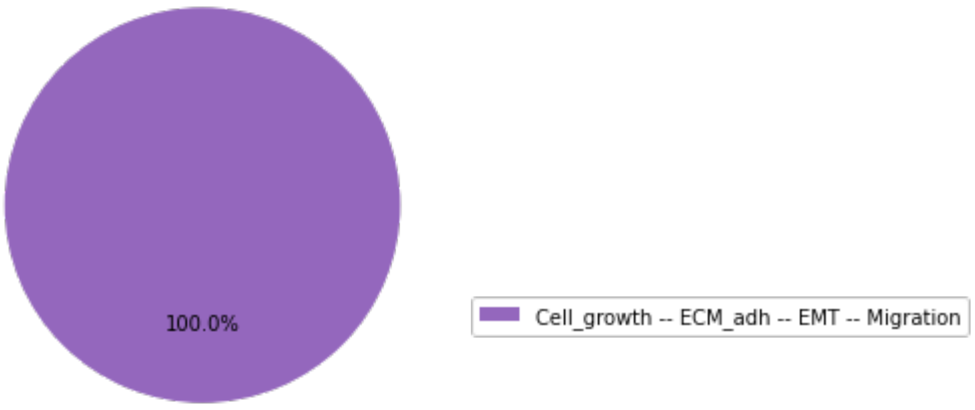

(('RAC1', 'ON'), ('miR34', 'OFF'))

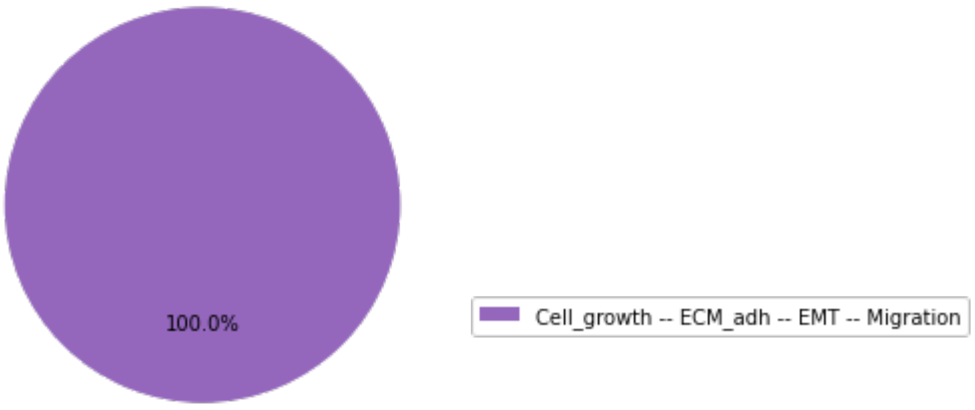

(('RAC1', 'ON'), ('miR200', 'OFF'))

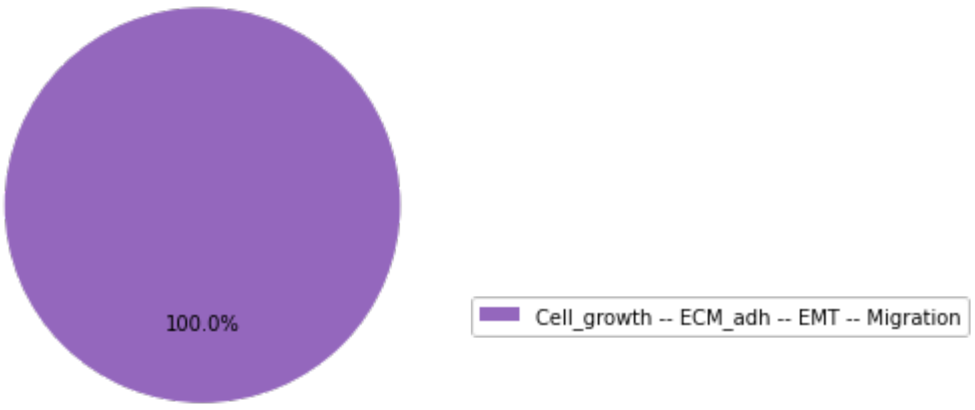

(('RAC1', 'ON'), ('TGFbetaR', 'OFF'))

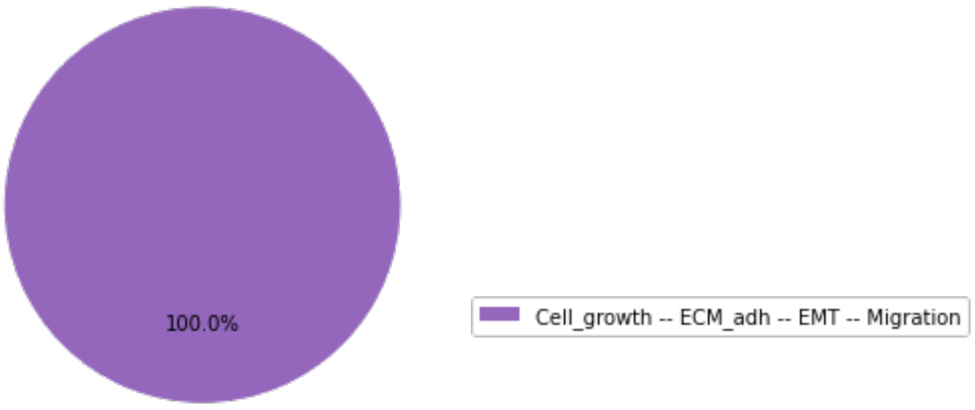

(('RAC1', 'ON'), ('FAK', 'OFF'))

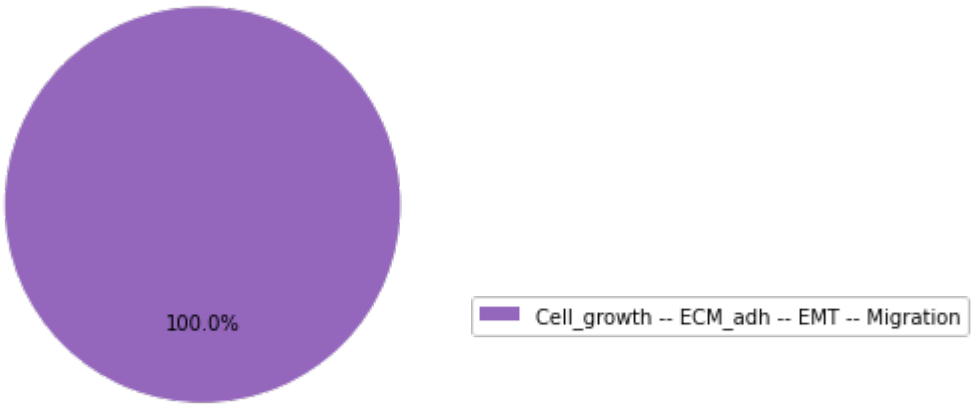

(('YAP1', 'ON'), ('PIK3CA', 'ON'))

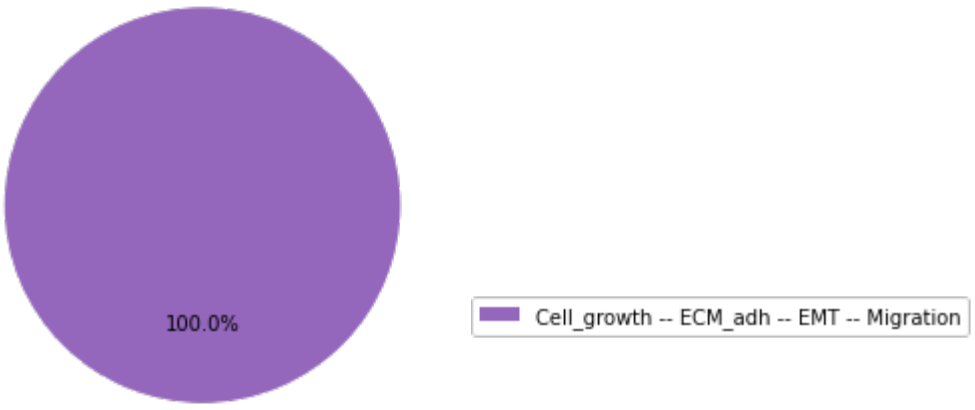

(('YAP1', 'ON'), ('ERK', 'ON'))

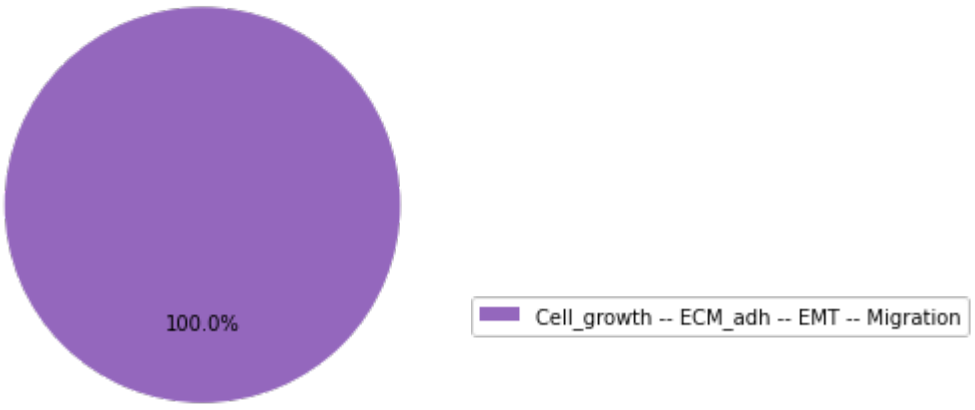

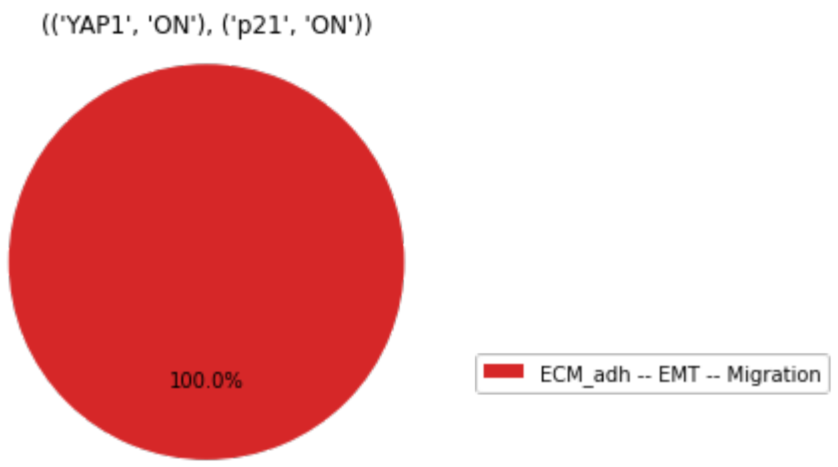
(('YAP1', 'ON'), ('p21', 'ON'))
100.0%
ECM_adh -- EMT -- Migration

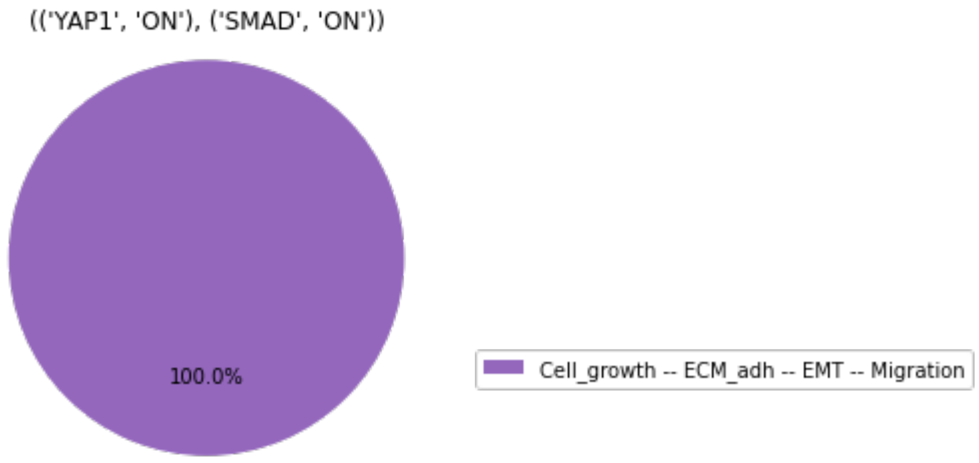
(('YAP1', 'ON'), ('SMAD', 'ON'))
100.0%
Cell_growth -- ECM_adh -- EMT -- Migration

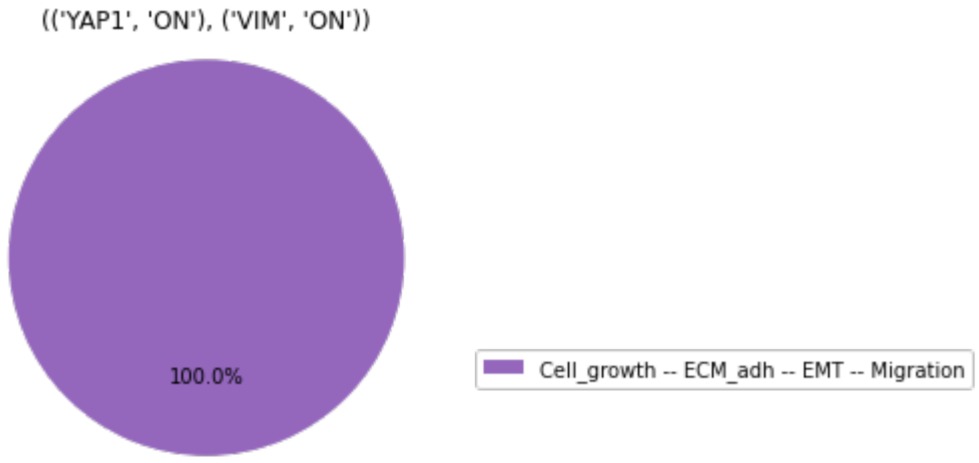
(('YAP1', 'ON'), ('VIM', 'ON'))
100.0%
Cell_growth -- ECM_adh -- EMT -- Migration

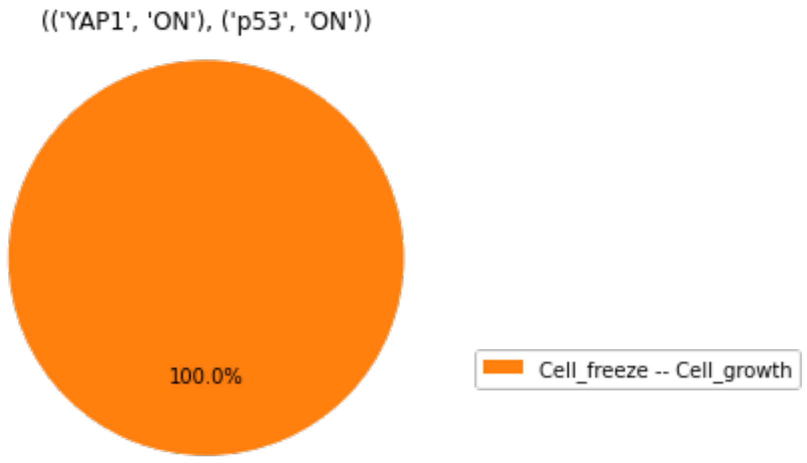
(('YAP1', 'ON'), ('p53', 'ON'))
100.0%
Cell_freeze -- Cell_growth

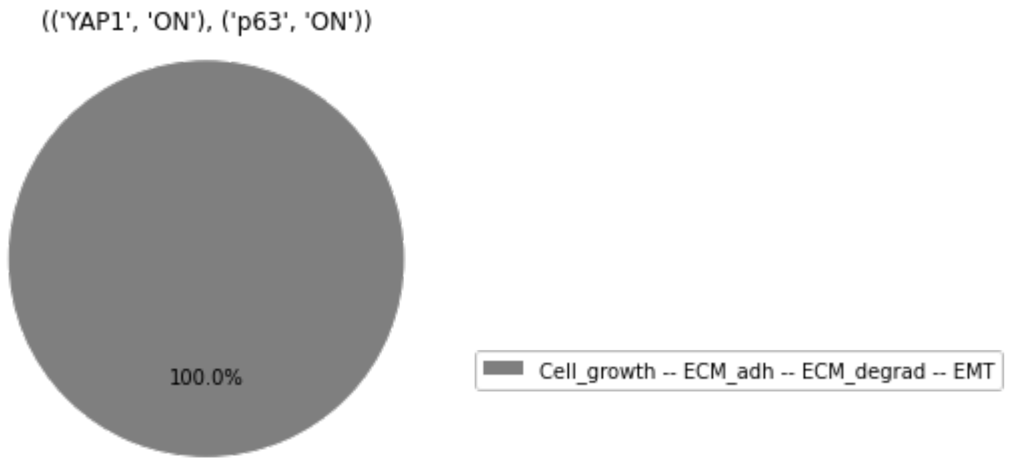
(('YAP1', 'ON'), ('p63', 'ON'))
100.0%
Cell_growth -- ECM_adh -- ECM_degrad -- EMT

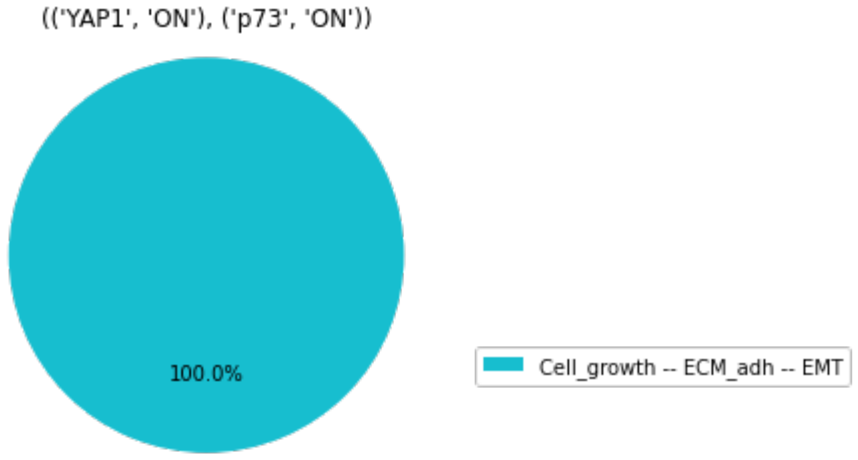
(('YAP1', 'ON'), ('p73', 'ON'))
100.0%
Cell_growth -- ECM_adh -- EMT

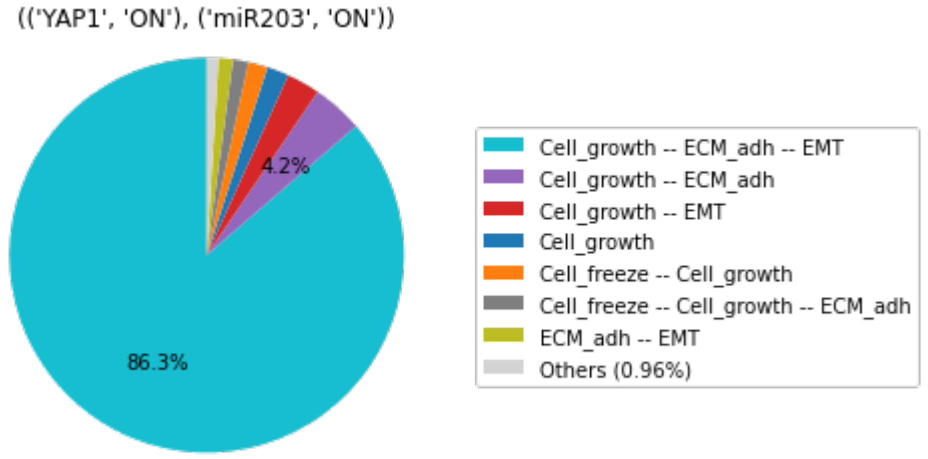
(('YAP1', 'ON'), ('miR203', 'ON'))
4.2%
86.3%
Cell_growth -- ECM_adh -- EMT
Cell_growth -- ECM_adh
Cell_growth -- EMT
Cell_growth
Cell_freeze -- Cell_growth
Cell_freeze -- Cell_growth -- ECM_adh
ECM_adh -- EMT
Others (0.96%)

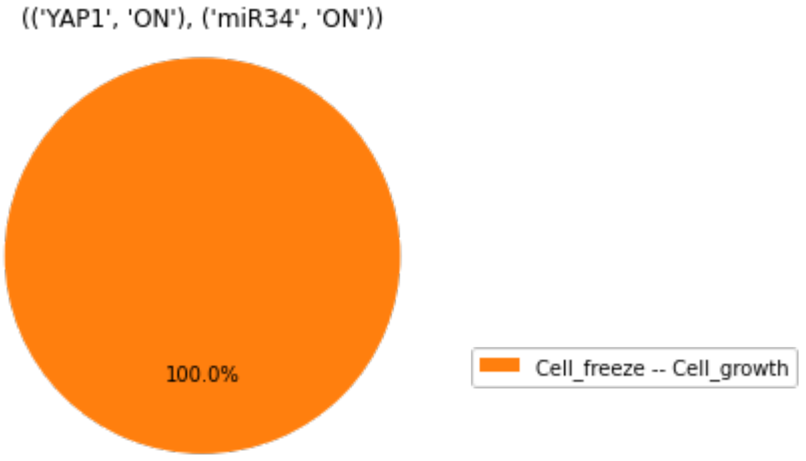
(('YAP1', 'ON'), ('miR34', 'ON'))
100.0%
Cell_freeze -- Cell_growth

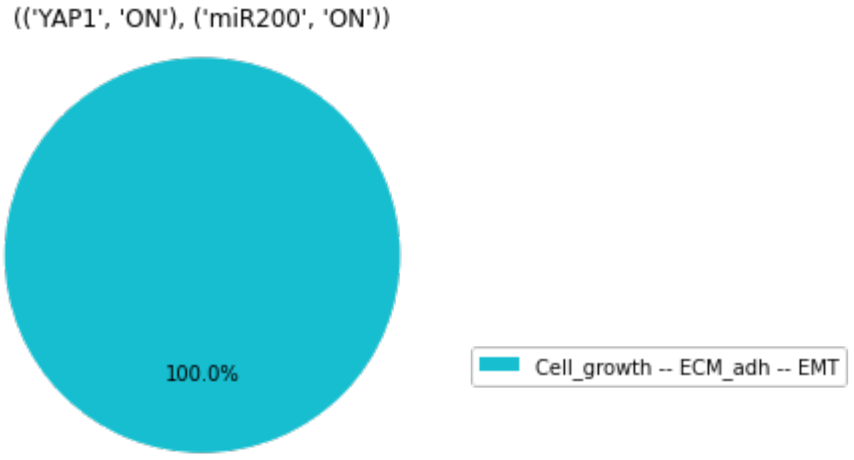
(('YAP1', 'ON'), ('miR200', 'ON'))
100.0%
Cell_growth -- ECM_adh -- EMT

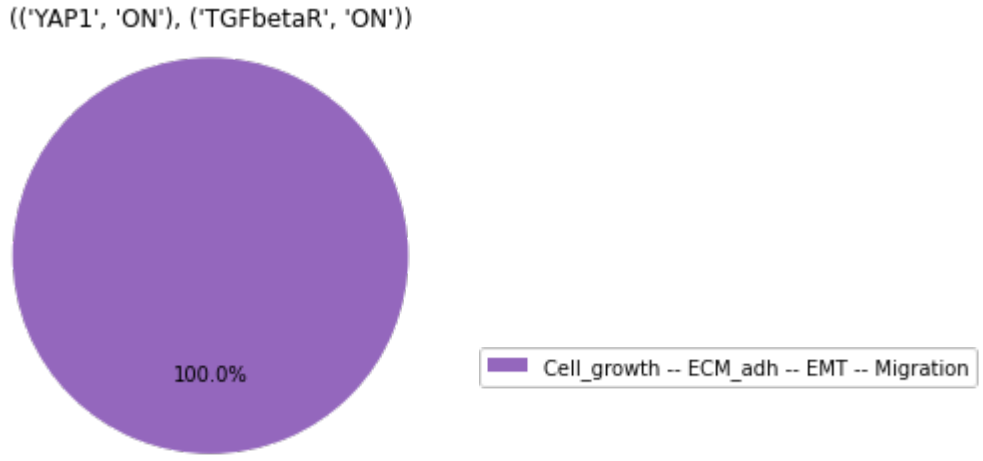
(('YAP1', 'ON'), ('TGFbetaR', 'ON'))
100.0%
Cell_growth -- ECM_adh -- EMT -- Migration

(('YAP1', 'ON'), ('FAK', 'ON'))

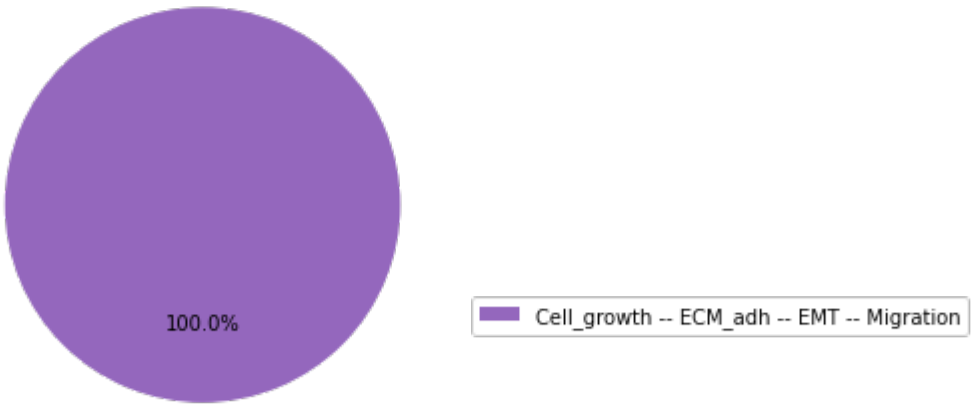

(('YAP1', 'ON'), ('AKT1', 'OFF'))

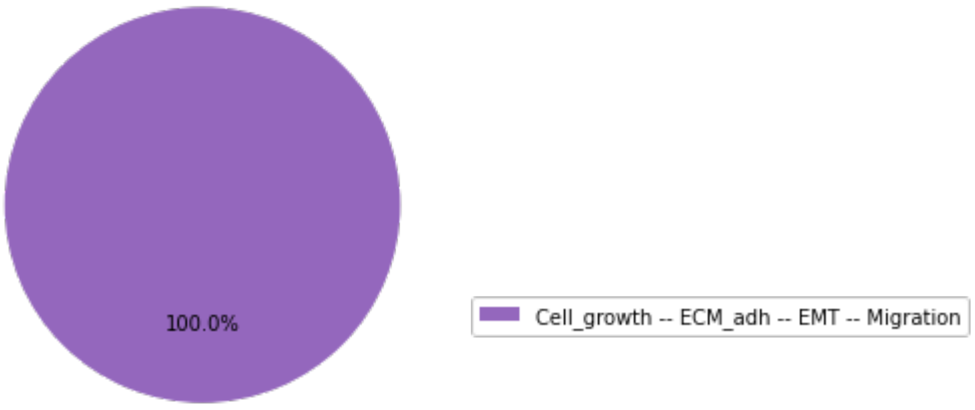

(('YAP1', 'ON'), ('CTNNB1', 'OFF'))

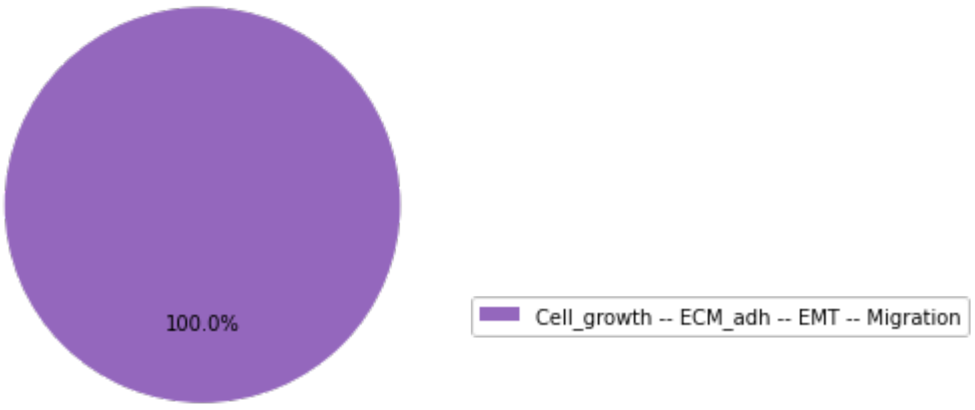

(('YAP1', 'ON'), ('DKK1', 'OFF'))

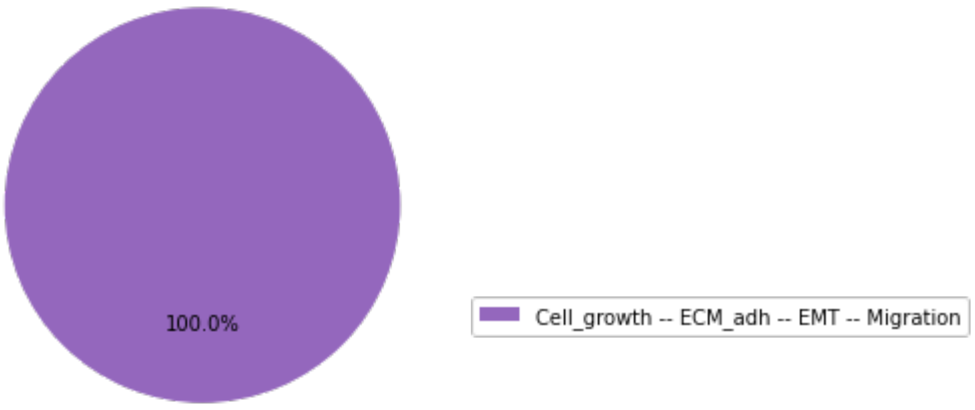

(('YAP1', 'ON'), ('AKT2', 'OFF'))

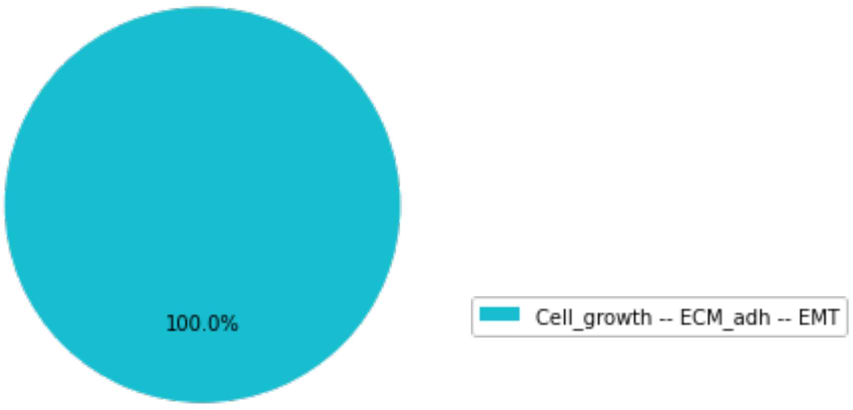

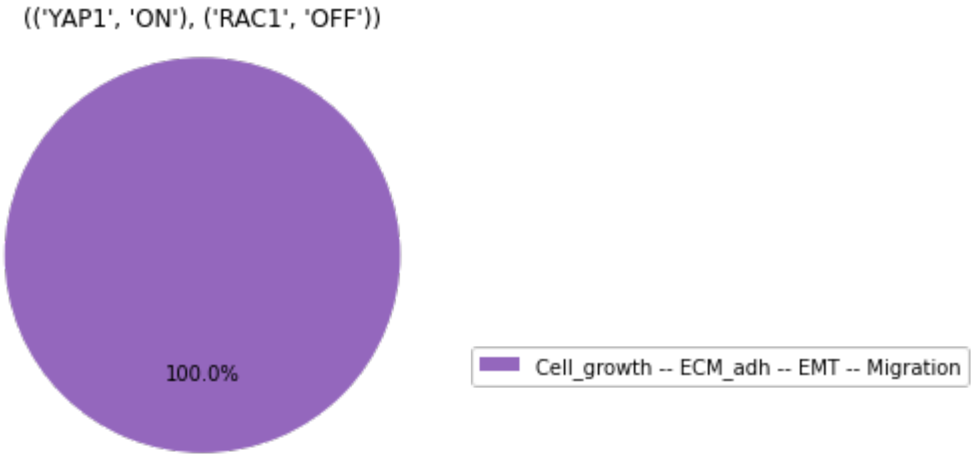
(('YAP1', 'ON'), ('RAC1', 'OFF'))
100.0%
Cell_growth -- ECM_adh -- EMT -- Migration

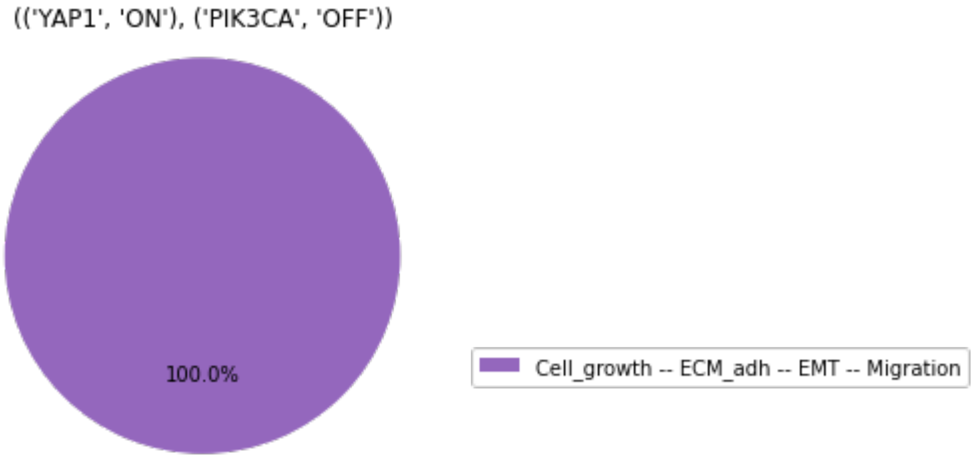
(('YAP1', 'ON'), ('PIK3CA', 'OFF'))
100.0%
Cell_growth -- ECM_adh -- EMT -- Migration

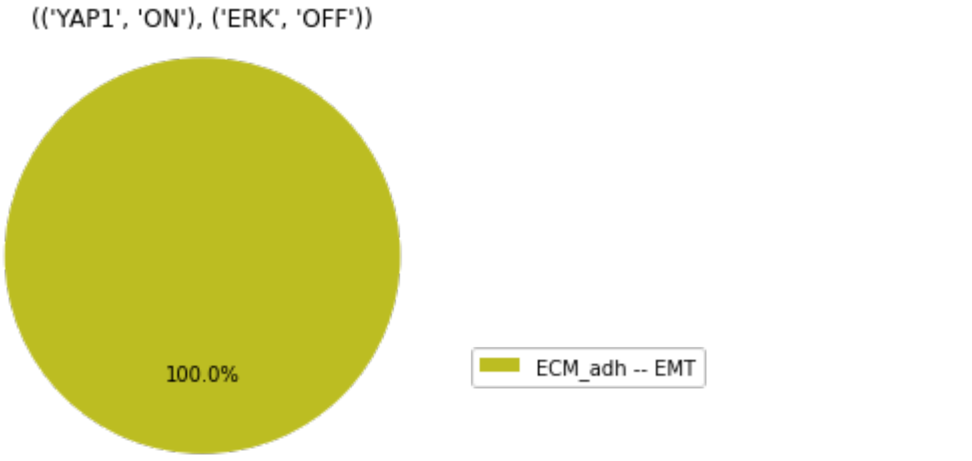
(('YAP1', 'ON'), ('ERK', 'OFF'))
100.0%
ECM_adh -- EMT

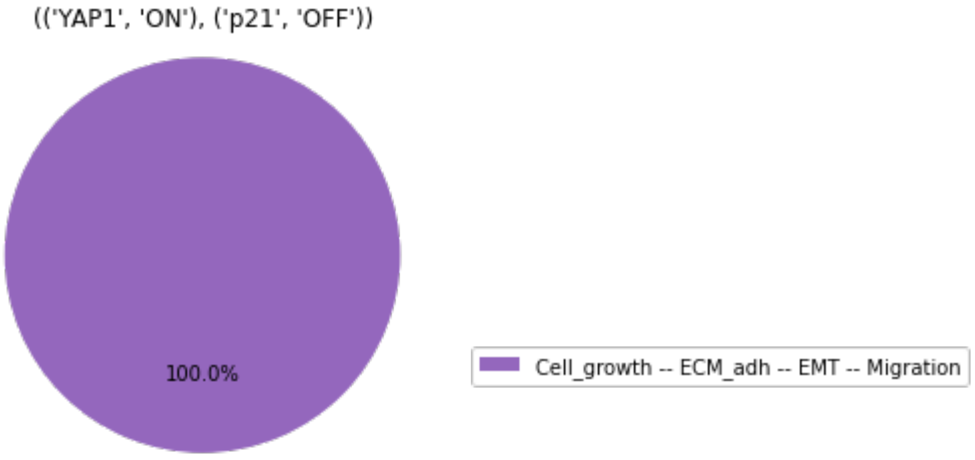
(('YAP1', 'ON'), ('p21', 'OFF'))
100.0%
Cell_growth -- ECM_adh -- EMT -- Migration

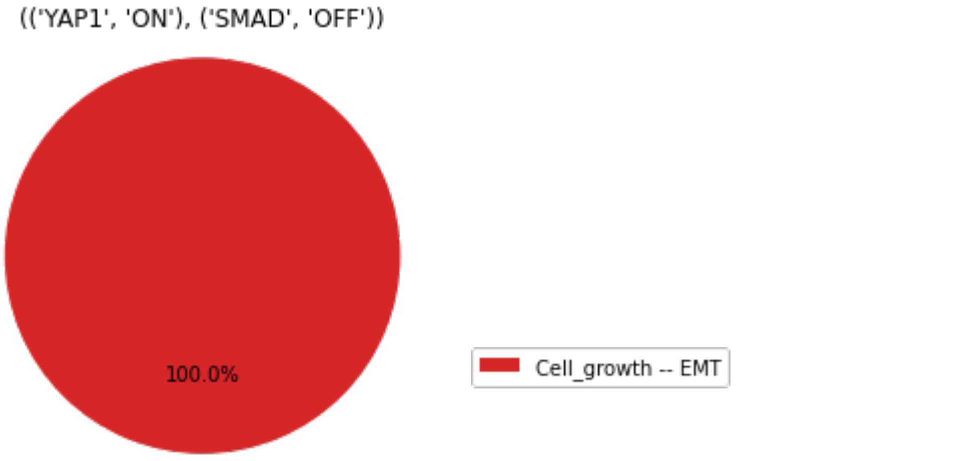
(('YAP1', 'ON'), ('SMAD', 'OFF'))
100.0%
Cell_growth -- EMT

(('YAP1', 'ON'), ('VIM', 'OFF'))

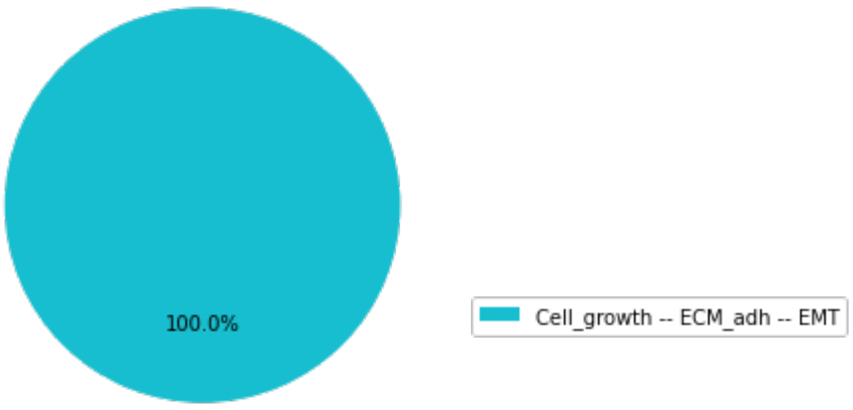

(('YAP1', 'ON'), ('p53', 'OFF'))

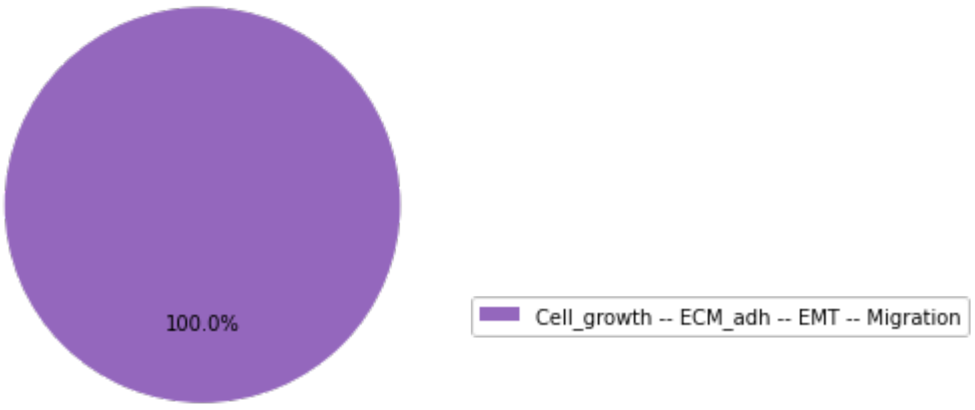

(('YAP1', 'ON'), ('p63', 'OFF'))

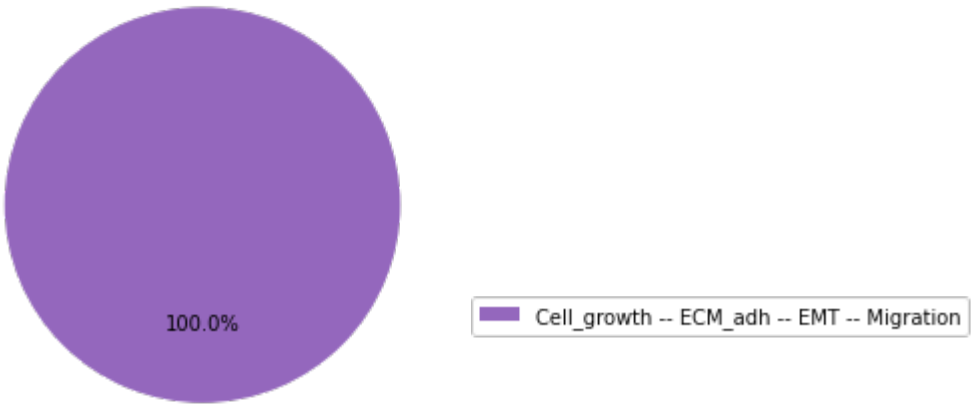

(('YAP1', 'ON'), ('p73', 'OFF'))

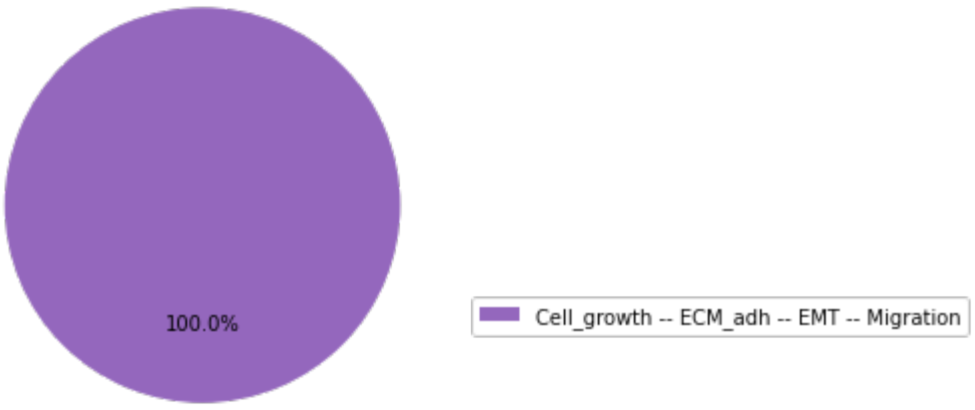

(('YAP1', 'ON'), ('miR203', 'OFF'))

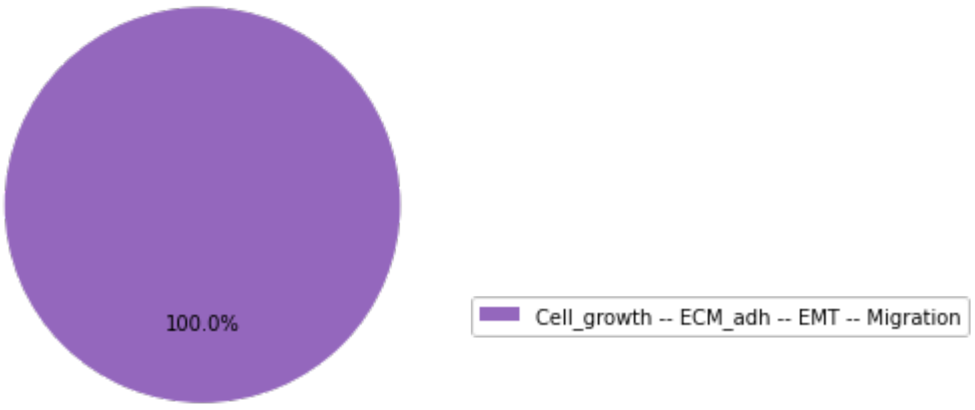

(('YAP1', 'ON'), ('miR34', 'OFF'))

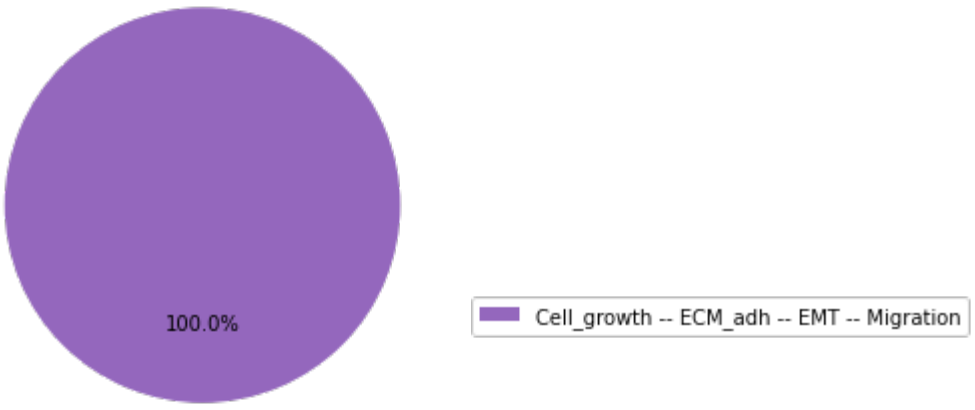

(('YAP1', 'ON'), ('miR200', 'OFF'))

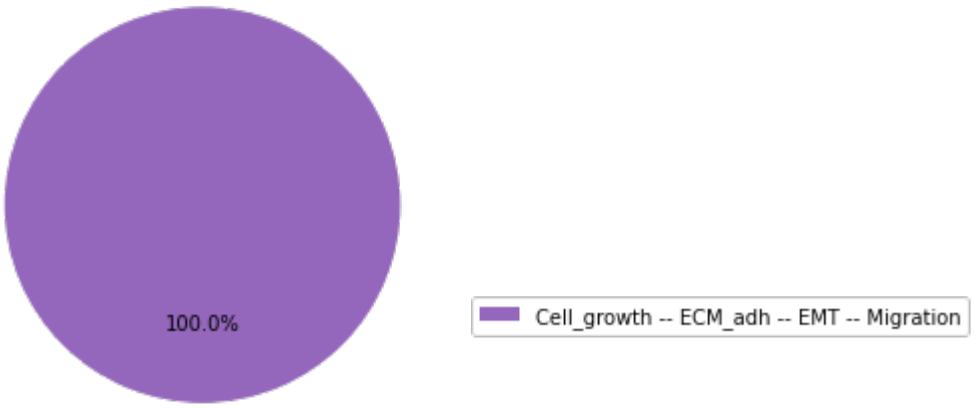

(('YAP1', 'ON'), ('TGFbetaR', 'OFF'))

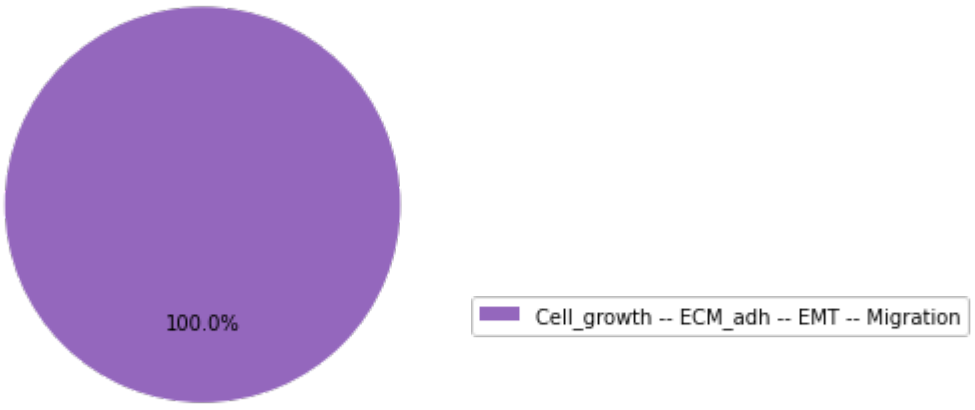

(('YAP1', 'ON'), ('FAK', 'OFF'))

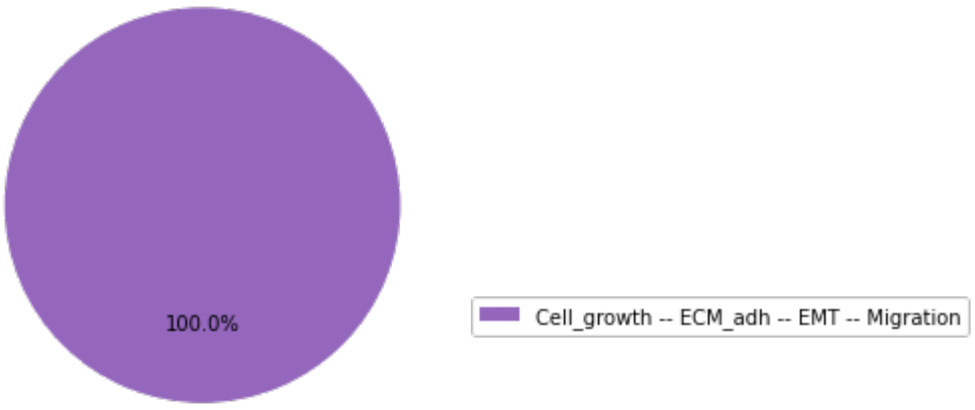

(('PIK3CA', 'ON'), ('ERK', 'ON'))

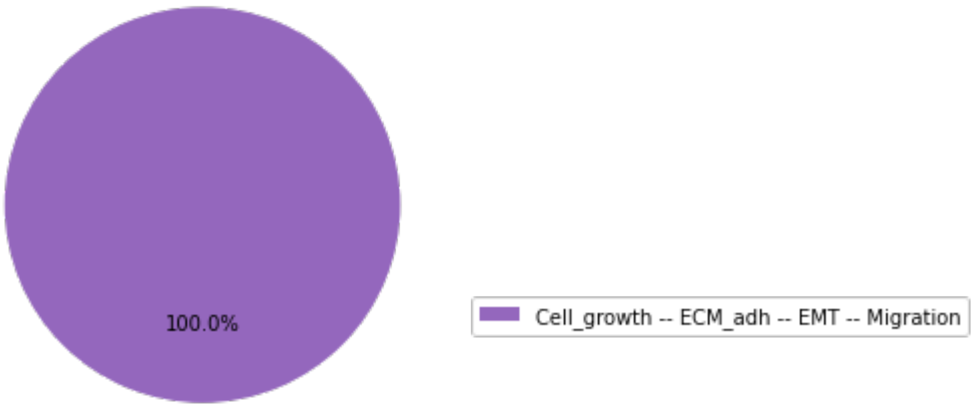

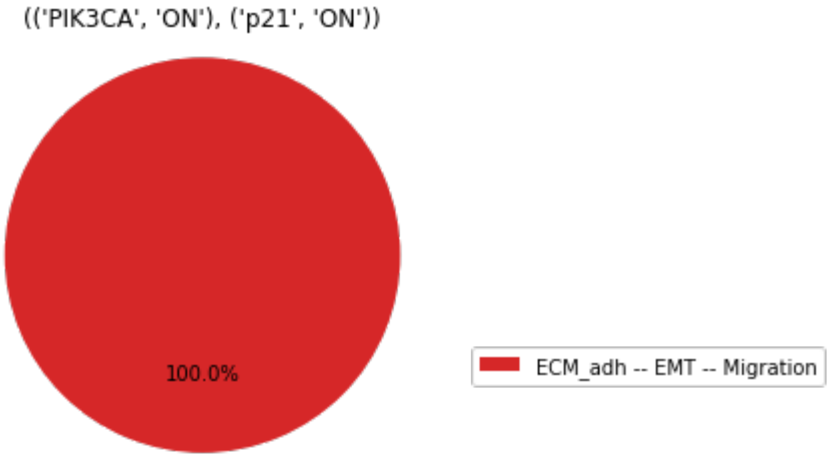
(('PIK3CA', 'ON'), ('p21', 'ON'))
100.0%
ECM_adh -- EMT -- Migration

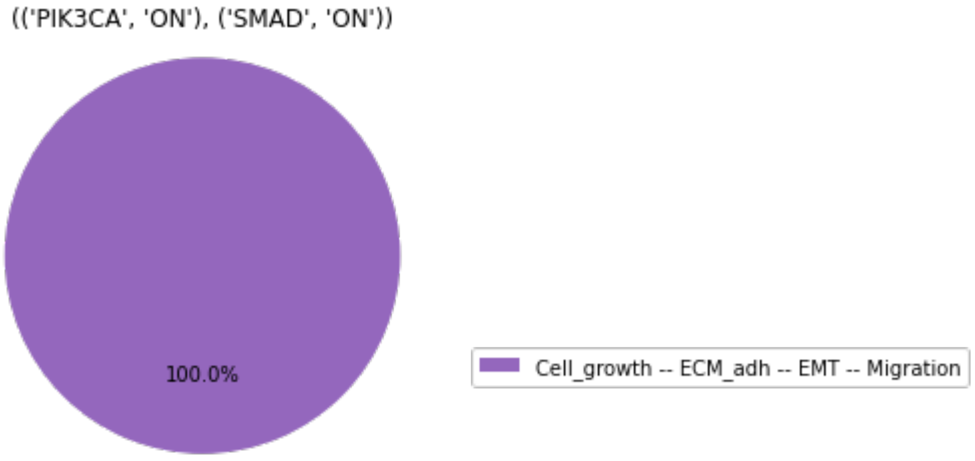
(('PIK3CA', 'ON'), ('SMAD', 'ON'))
100.0%
Cell_growth -- ECM_adh -- EMT -- Migration

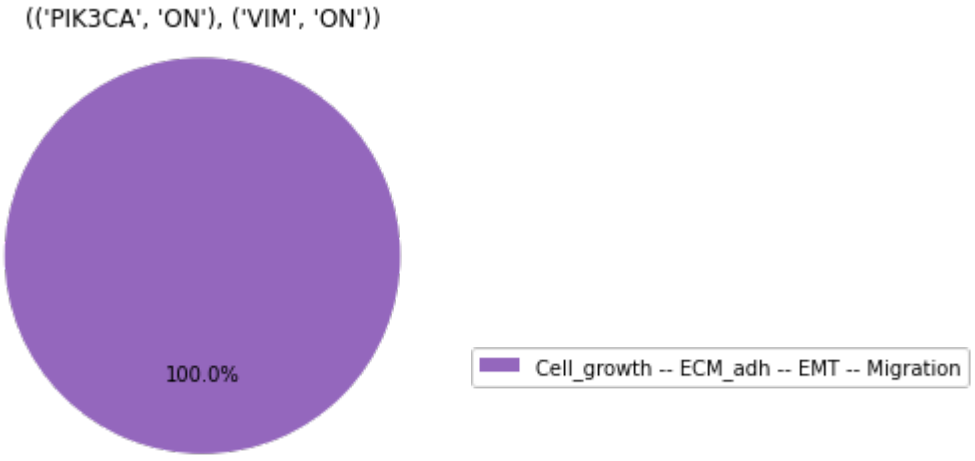
(('PIK3CA', 'ON'), ('VIM', 'ON'))
100.0%
Cell_growth -- ECM_adh -- EMT -- Migration

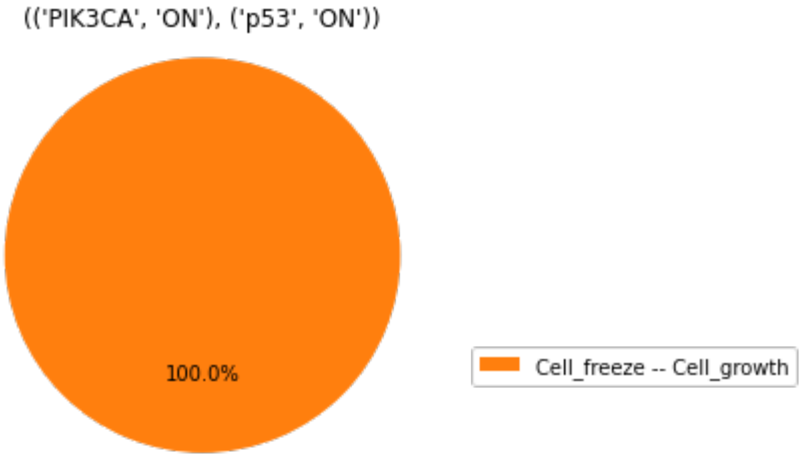
(('PIK3CA', 'ON'), ('p53', 'ON'))
100.0%
Cell_freeze -- Cell_growth

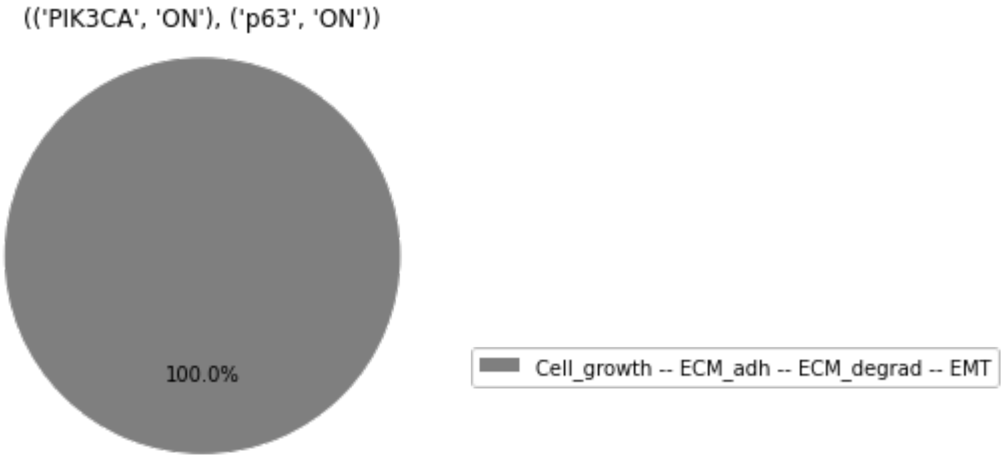
(('PIK3CA', 'ON'), ('p63', 'ON'))
100.0%
Cell_growth -- ECM_adh -- ECM_degrad -- EMT

(('PIK3CA', 'ON'), ('p73', 'ON'))

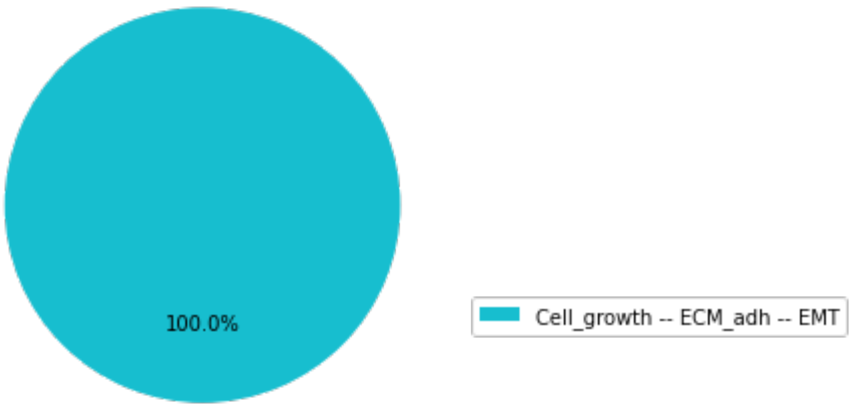

(('PIK3CA', 'ON'), ('miR203', 'ON'))

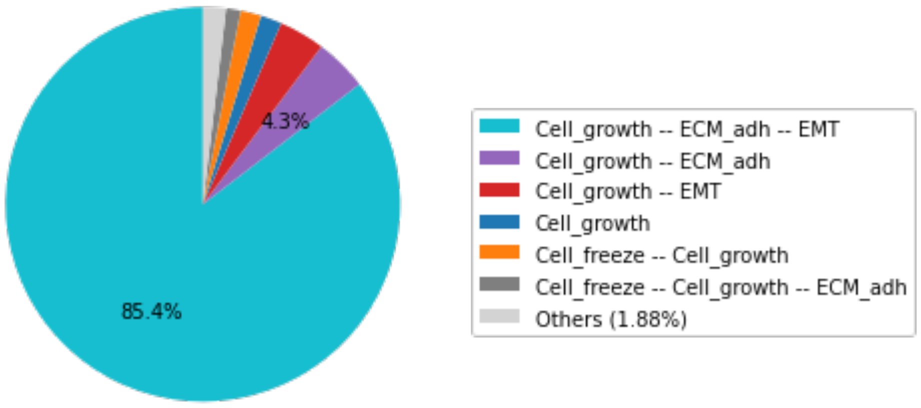

(('PIK3CA', 'ON'), ('miR34', 'ON'))

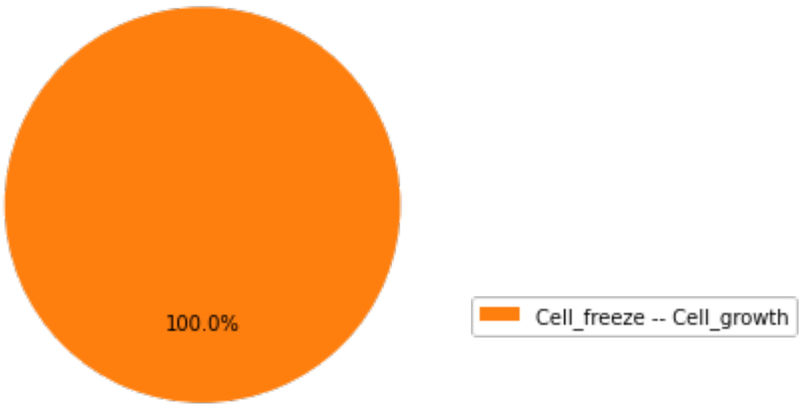

(('PIK3CA', 'ON'), ('miR200', 'ON'))

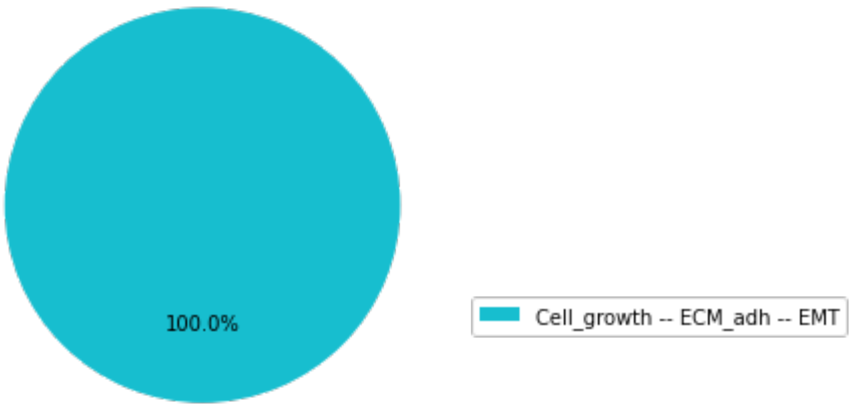

(('PIK3CA', 'ON'), ('TGFbetaR', 'ON'))

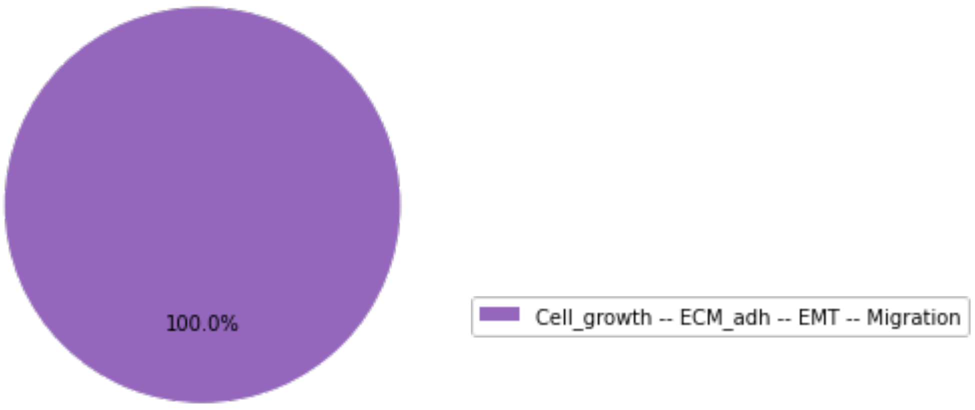

(('PIK3CA', 'ON'), ('FAK', 'ON'))

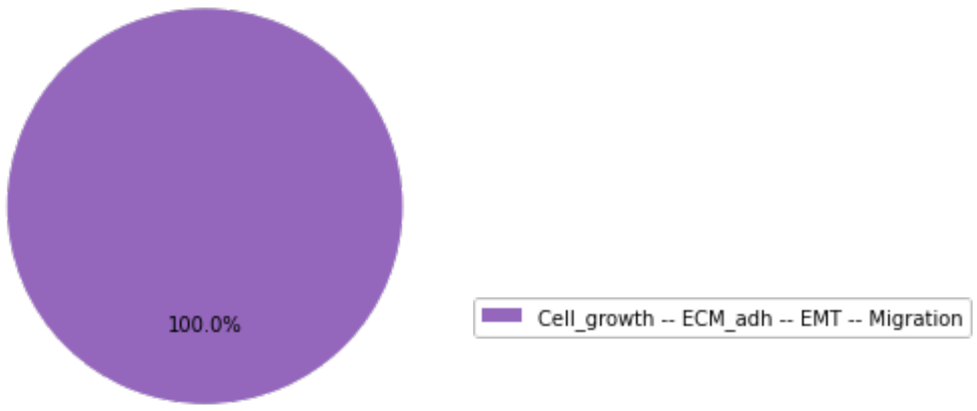

(('PIK3CA', 'ON'), ('AKT1', 'OFF'))

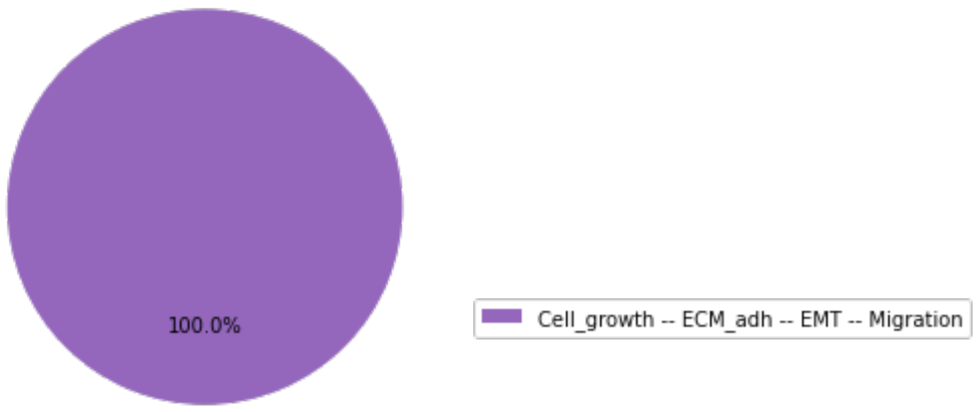

(('PIK3CA', 'ON'), ('CTNNB1', 'OFF'))

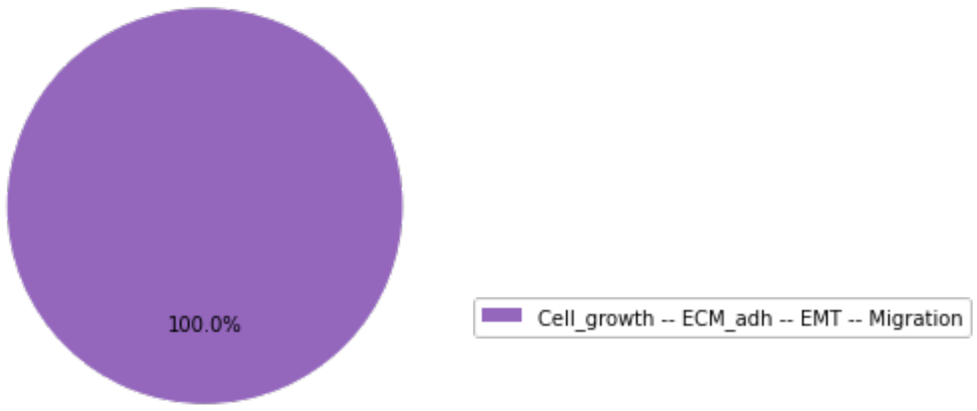

(('PIK3CA', 'ON'), ('DKK1', 'OFF'))

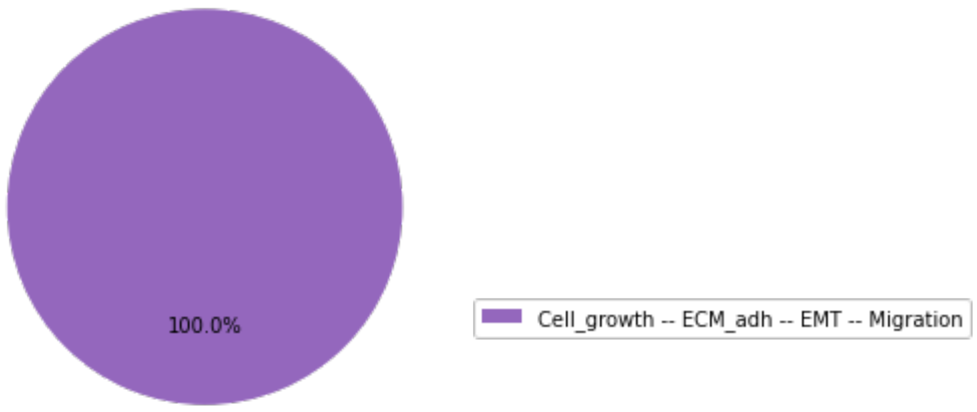

(('PIK3CA', 'ON'), ('AKT2', 'OFF'))

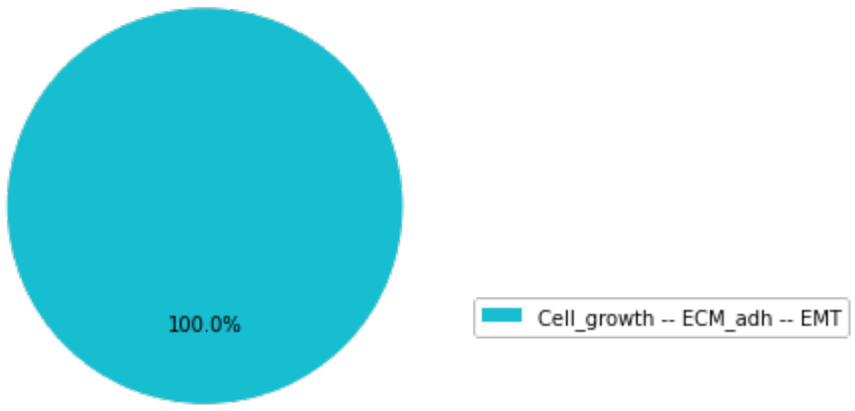

(('PIK3CA', 'ON'), ('RAC1', 'OFF'))

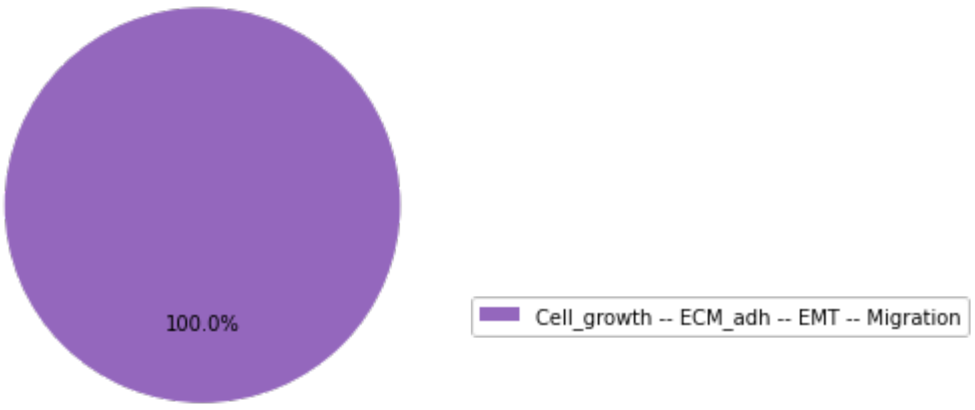

(('PIK3CA', 'ON'), ('YAP1', 'OFF'))

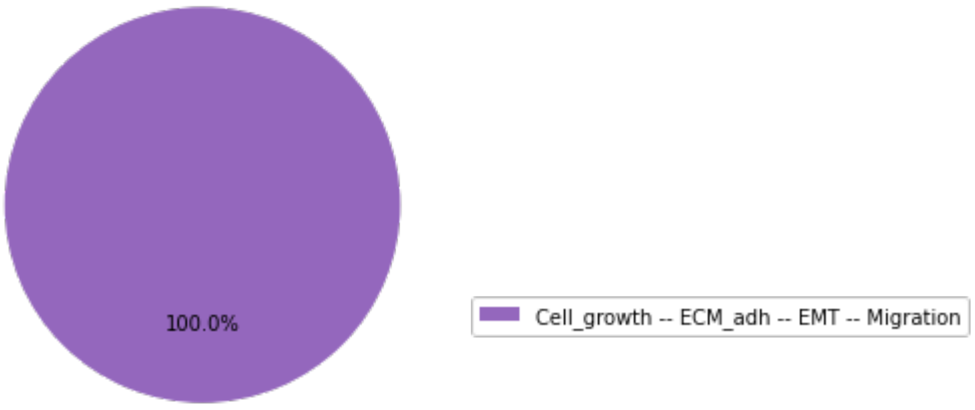

(('PIK3CA', 'ON'), ('ERK', 'OFF'))

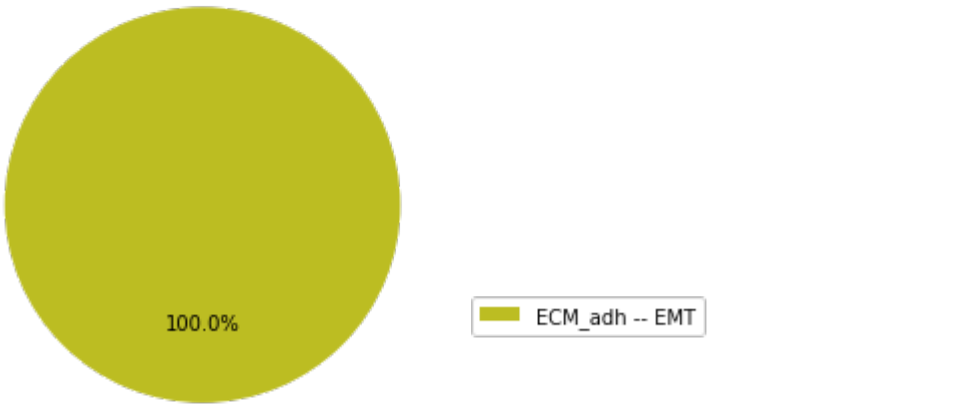

(('PIK3CA', 'ON'), ('p21', 'OFF'))

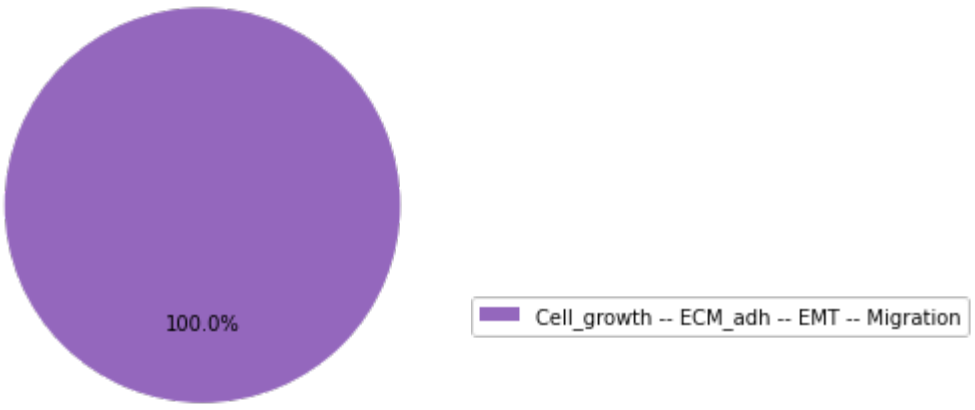

(('PIK3CA', 'ON'), ('SMAD', 'OFF'))

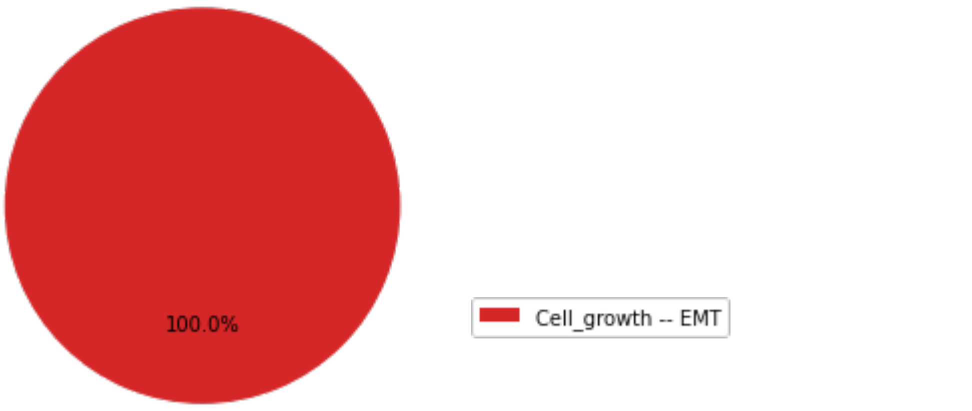

(('PIK3CA', 'ON'), ('VIM', 'OFF'))

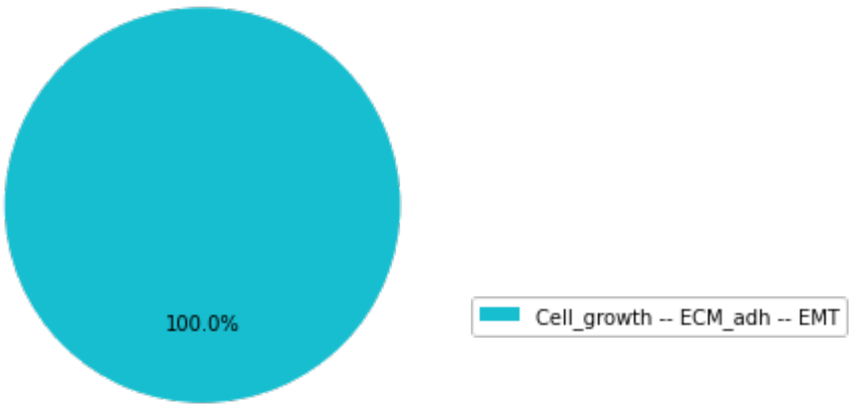

(('PIK3CA', 'ON'), ('p53', 'OFF'))

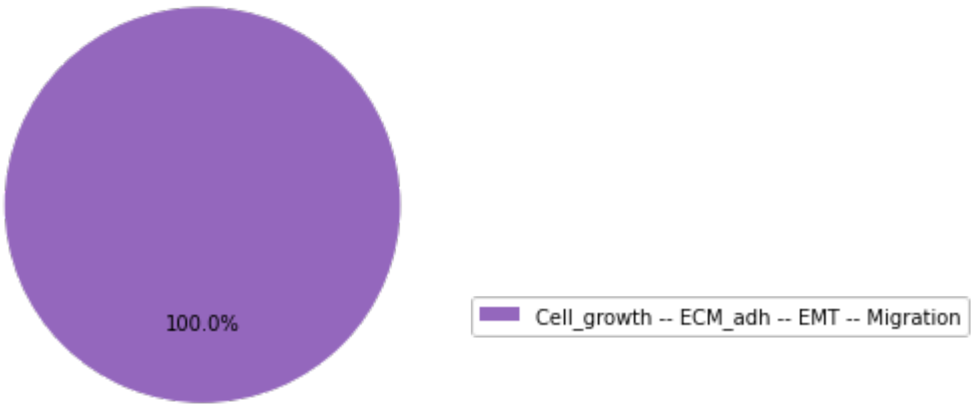

(('PIK3CA', 'ON'), ('p63', 'OFF'))

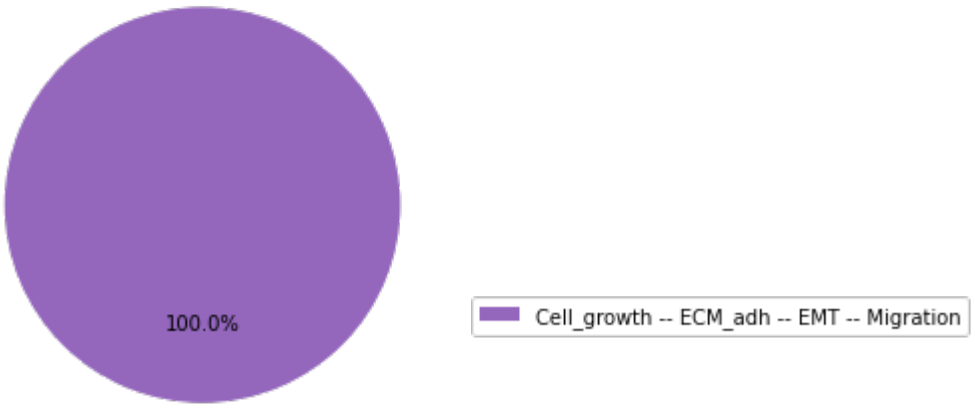

(('PIK3CA', 'ON'), ('p73', 'OFF'))

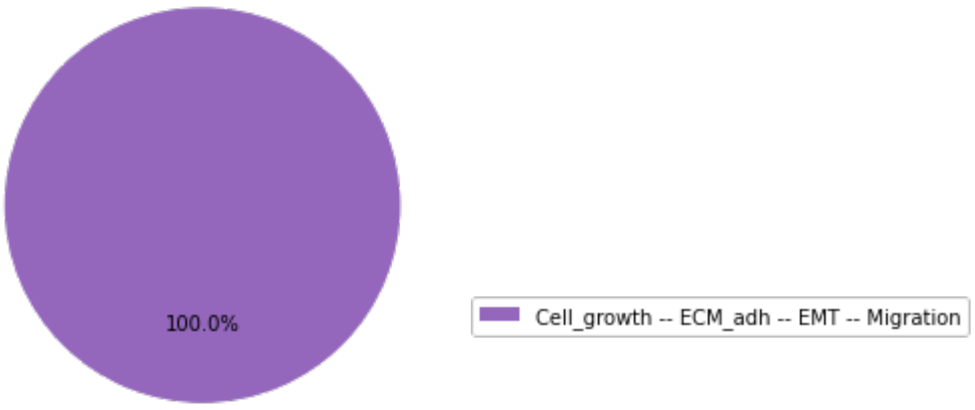

(('PIK3CA', 'ON'), ('miR203', 'OFF'))

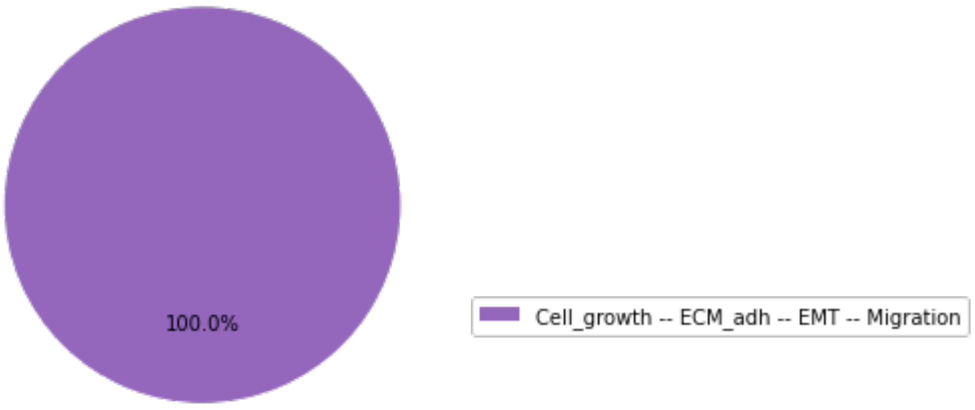

(('PIK3CA', 'ON'), ('miR34', 'OFF'))

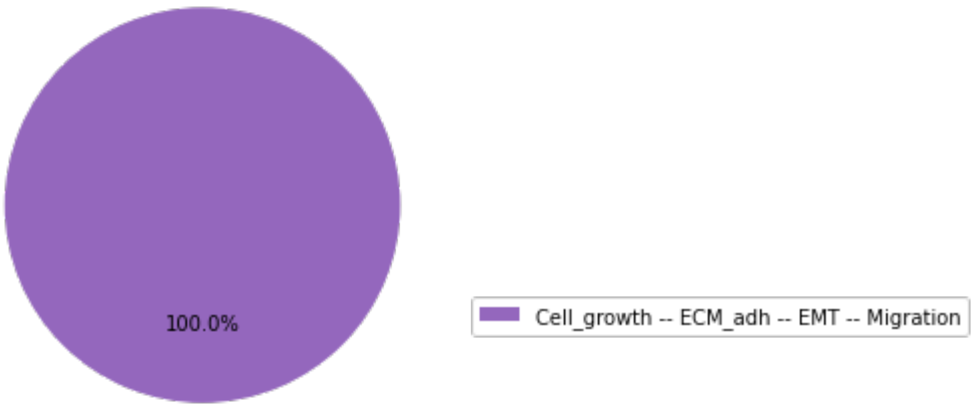

(('PIK3CA', 'ON'), ('miR200', 'OFF'))

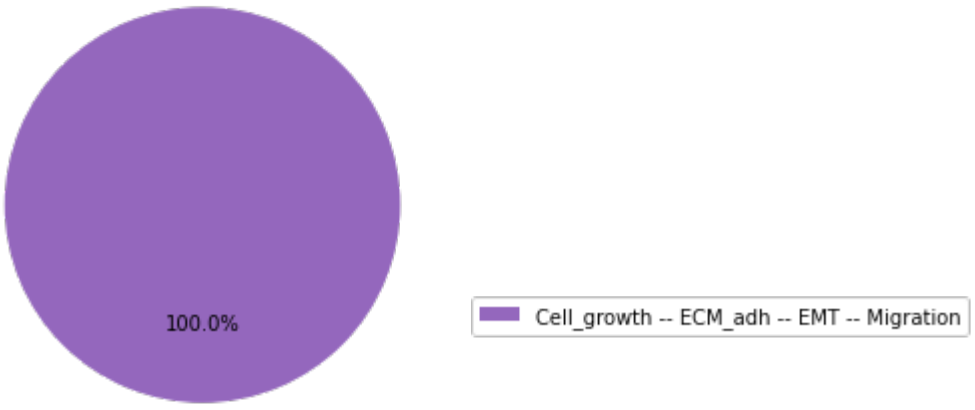

(('PIK3CA', 'ON'), ('TGFbetaR', 'OFF'))

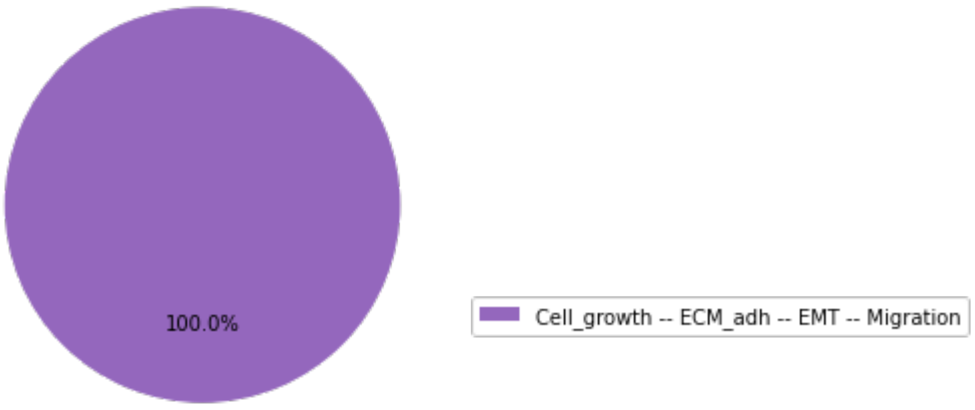

(('PIK3CA', 'ON'), ('FAK', 'OFF'))

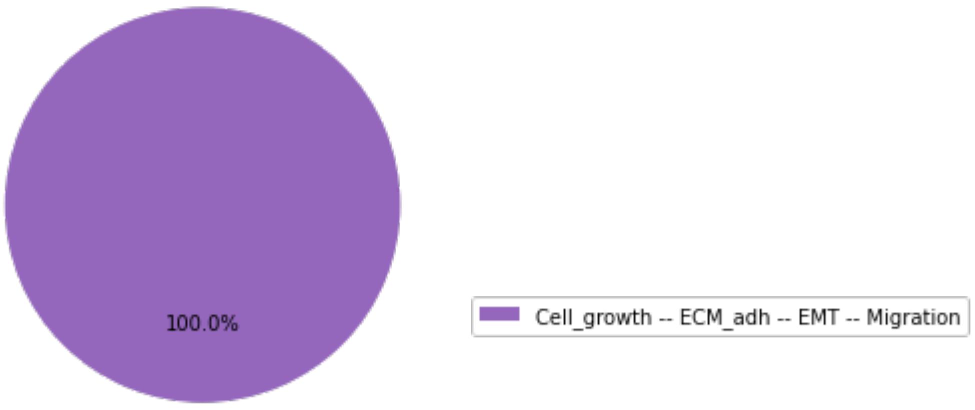

(('ERK', 'ON'), ('p21', 'ON'))

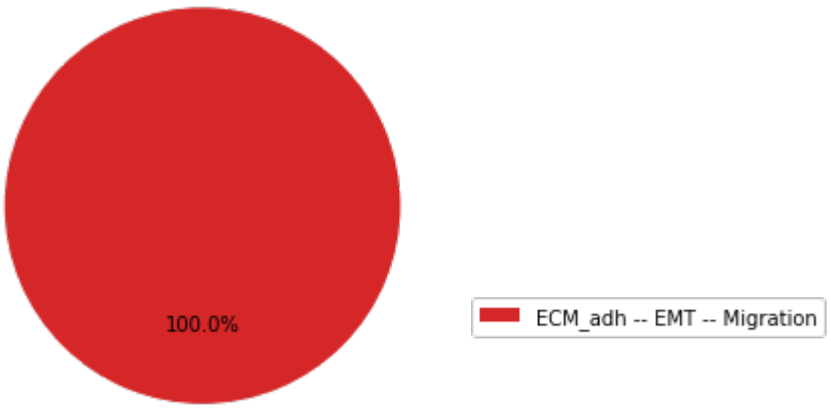

(('ERK', 'ON'), ('SMAD', 'ON'))

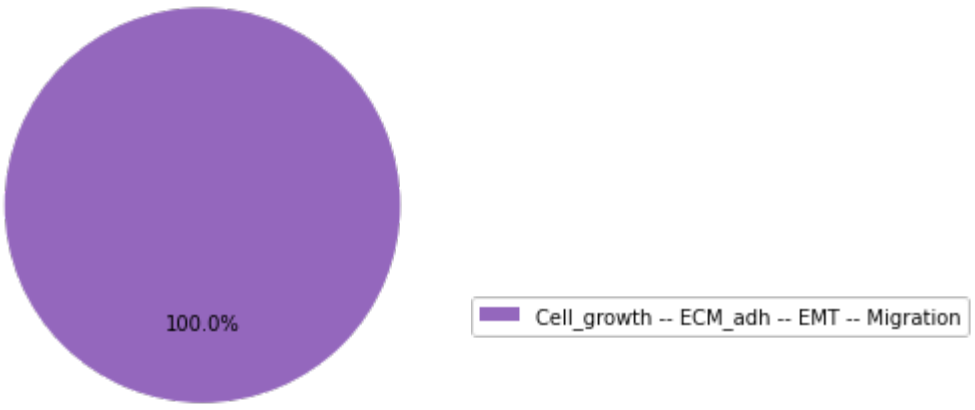

(('ERK', 'ON'), ('VIM', 'ON'))

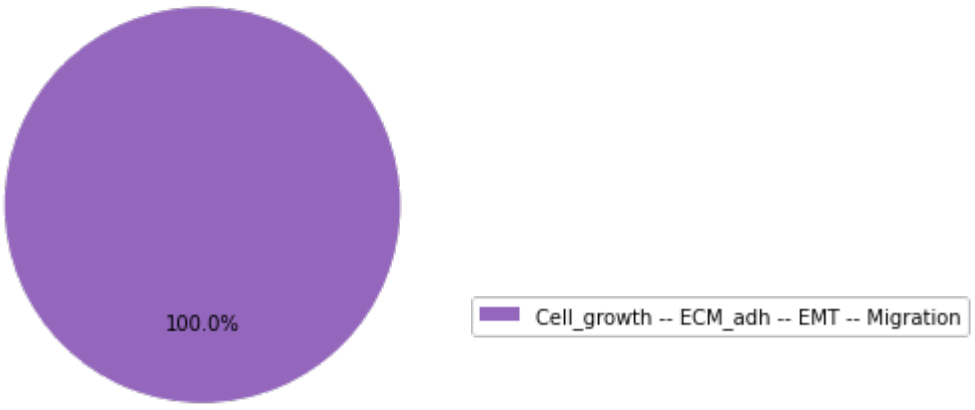

(('ERK', 'ON'), ('p53', 'ON'))

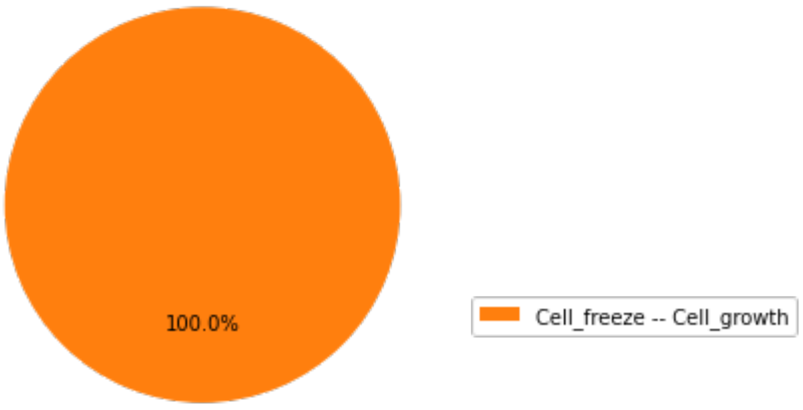

(('ERK', 'ON'), ('p63', 'ON'))

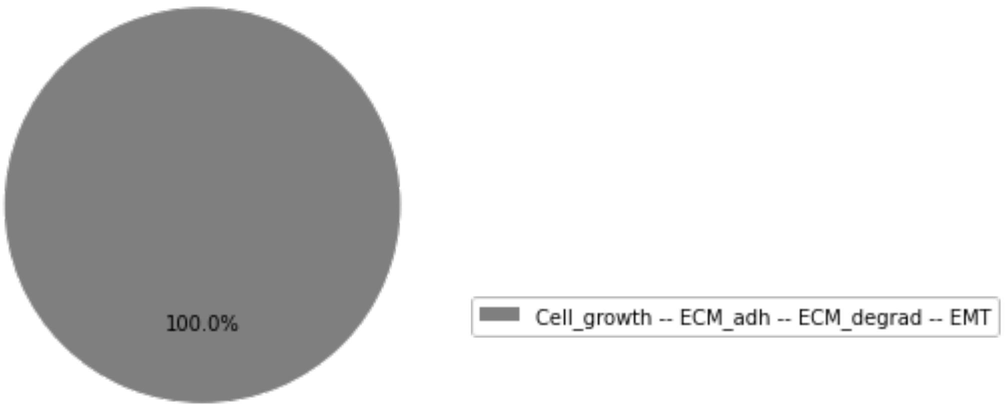

(('ERK', 'ON'), ('p73', 'ON'))

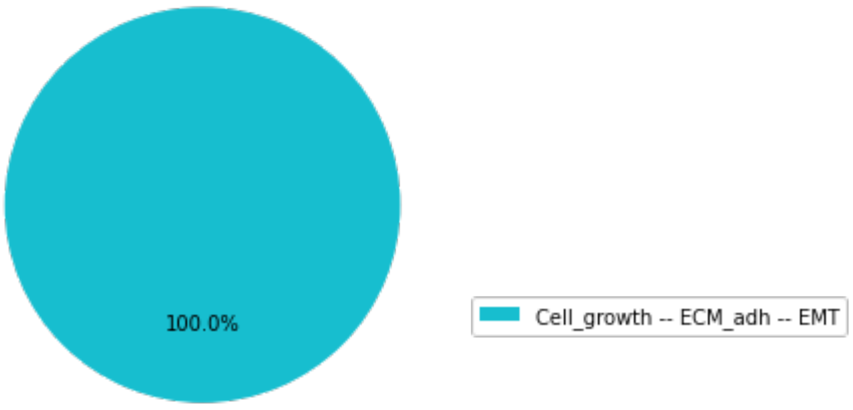

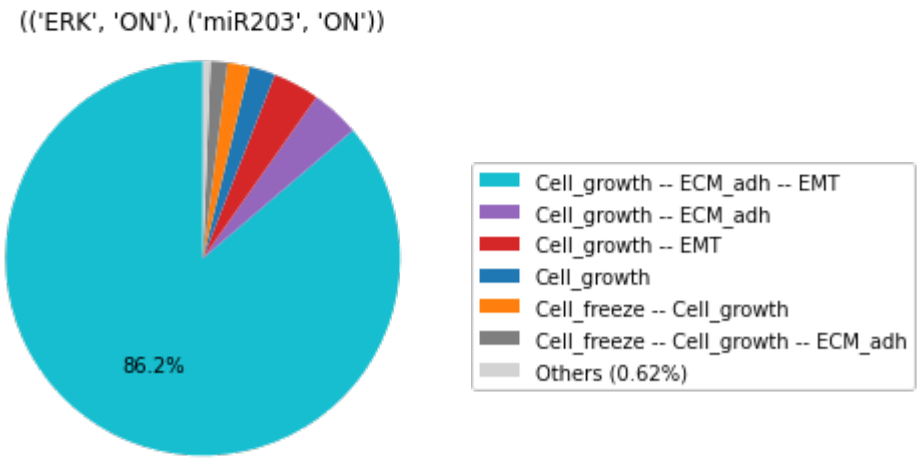
(('ERK', 'ON'), ('miR203', 'ON'))
86.2%
Cell_growth -- ECM_adh -- EMT
Cell_growth -- ECM_adh
Cell_growth -- EMT
Cell_growth
Cell_freeze -- Cell_growth
Cell_freeze -- Cell_growth -- ECM_adh
Others (0.62%)

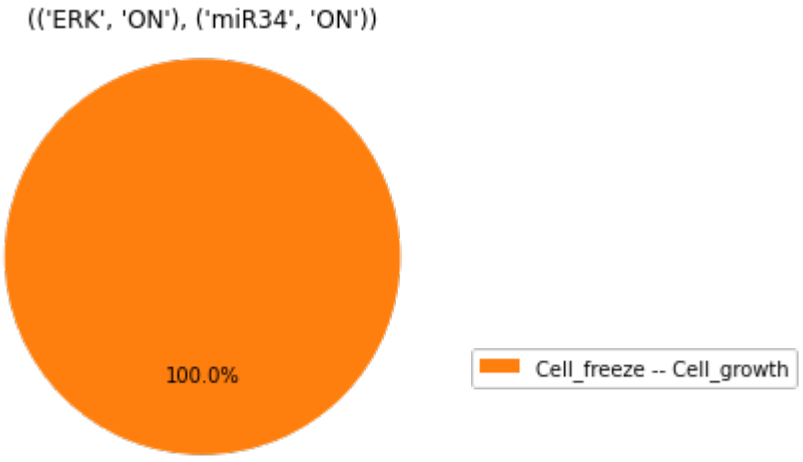
(('ERK', 'ON'), ('miR34', 'ON'))
100.0%
Cell_freeze -- Cell_growth

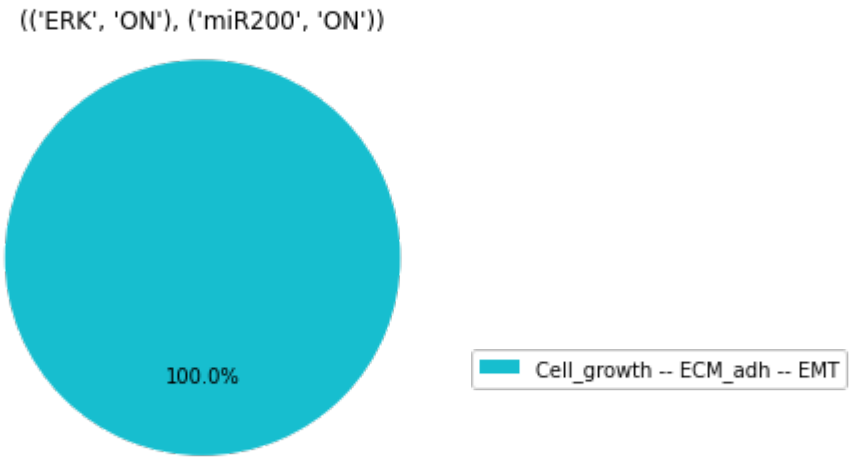
(('ERK', 'ON'), ('miR200', 'ON'))
100.0%
Cell_growth -- ECM_adh -- EMT

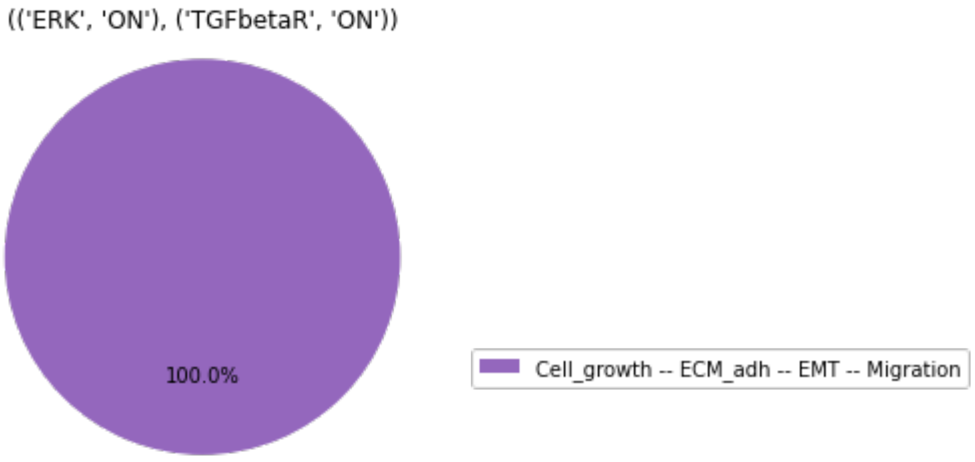
(('ERK', 'ON'), ('TGFbetaR', 'ON'))
100.0%
Cell_growth -- ECM_adh -- EMT -- Migration

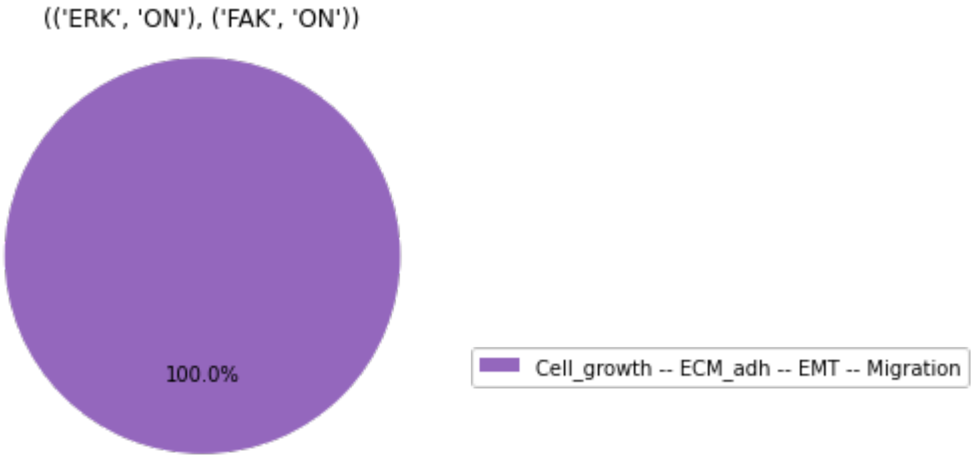
(('ERK', 'ON'), ('FAK', 'ON'))
100.0%
Cell_growth -- ECM_adh -- EMT -- Migration

(('ERK', 'ON'), ('AKT1', 'OFF'))

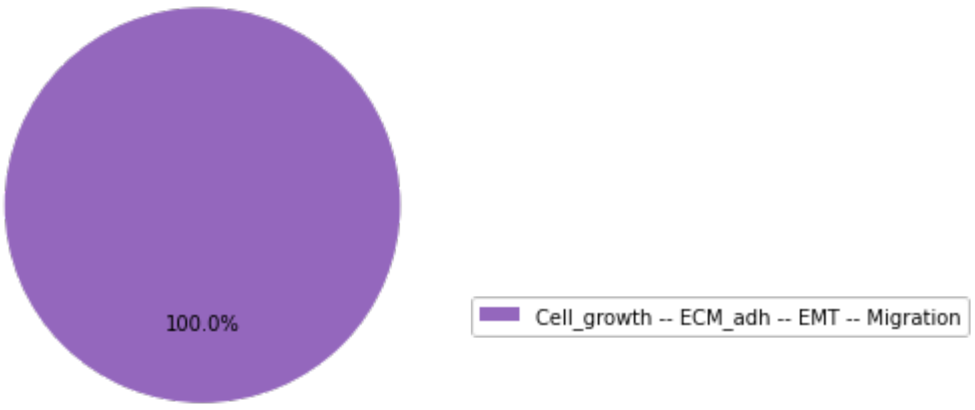

(('ERK', 'ON'), ('CTNNB1', 'OFF'))

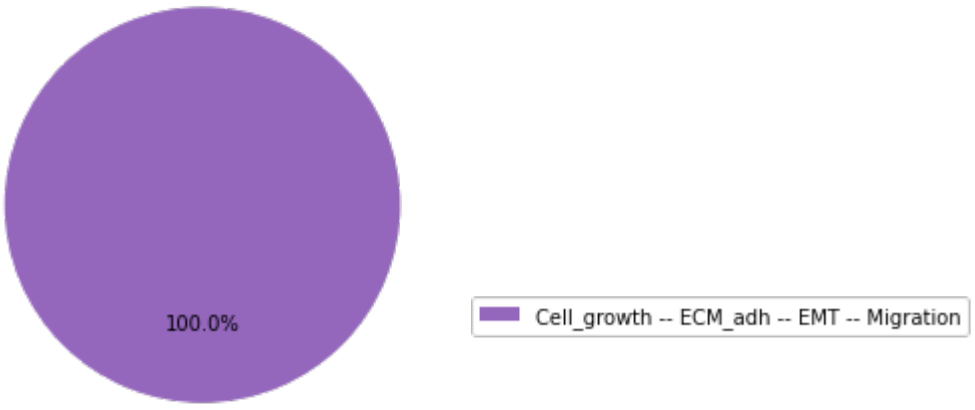

(('ERK', 'ON'), ('DKK1', 'OFF'))

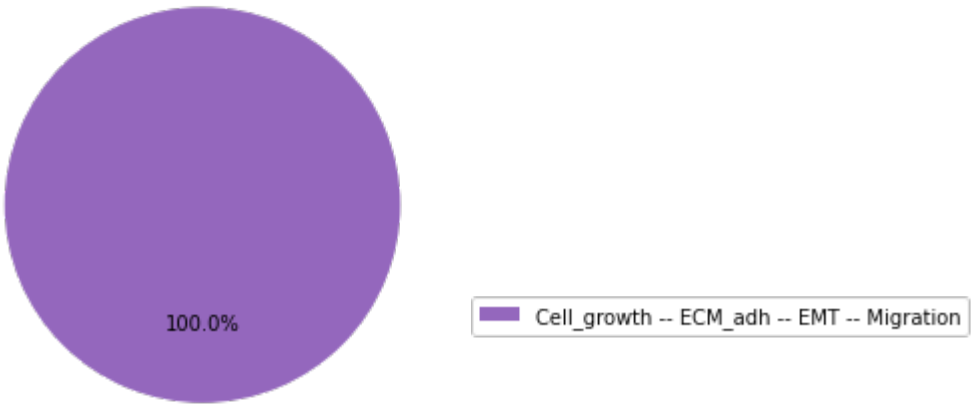

(('ERK', 'ON'), ('AKT2', 'OFF'))

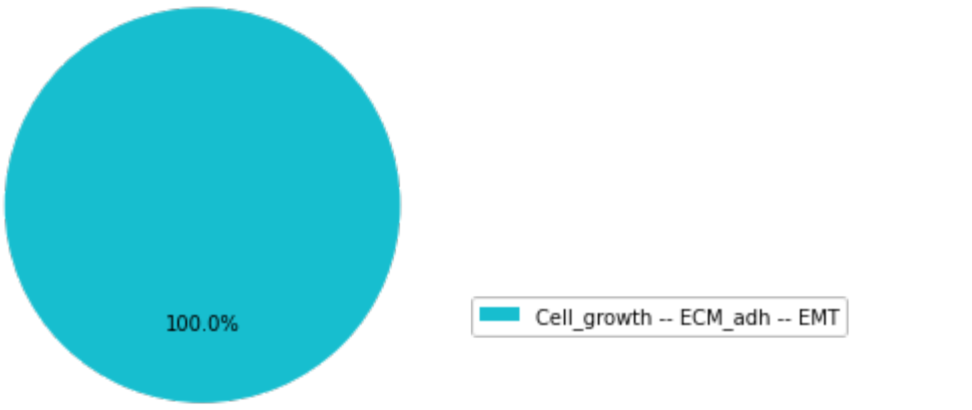

(('ERK', 'ON'), ('RAC1', 'OFF'))

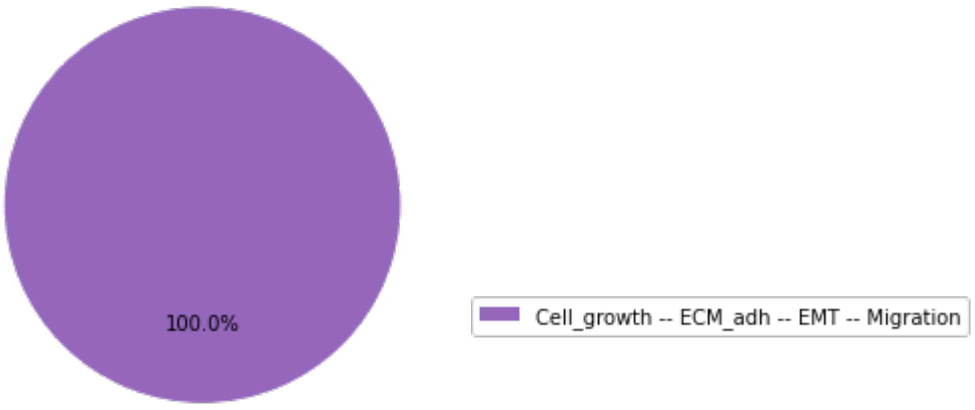

(('ERK', 'ON'), ('YAP1', 'OFF'))

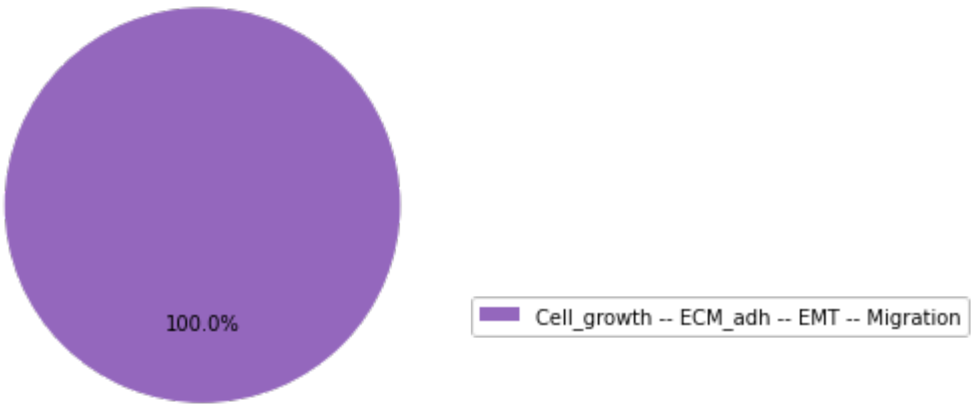

(('ERK', 'ON'), ('PIK3CA', 'OFF'))

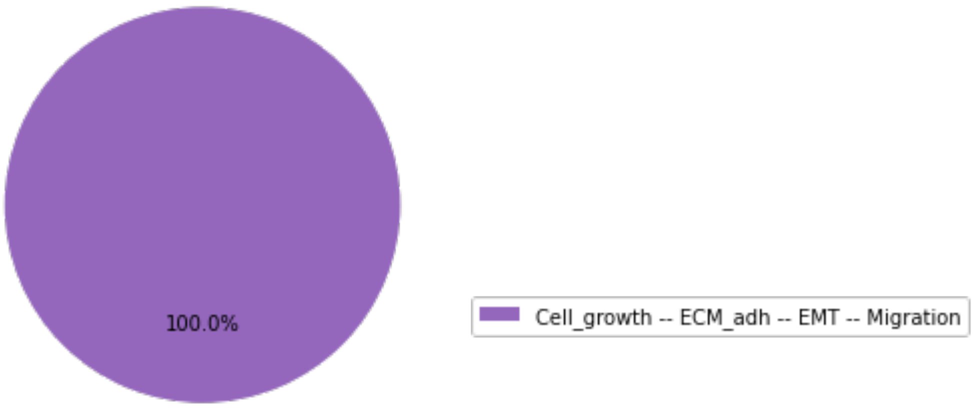

(('ERK', 'ON'), ('p21', 'OFF'))

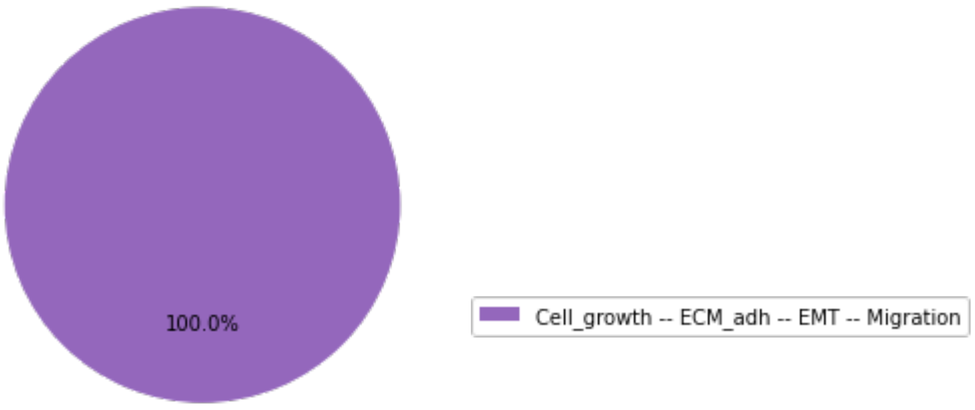

(('ERK', 'ON'), ('SMAD', 'OFF'))

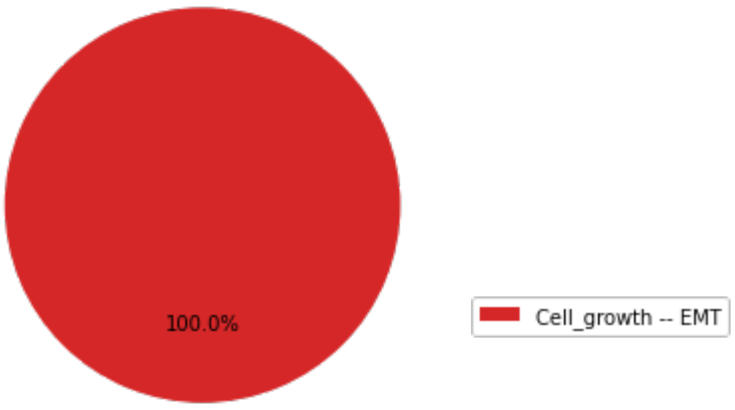

(('ERK', 'ON'), ('VIM', 'OFF'))

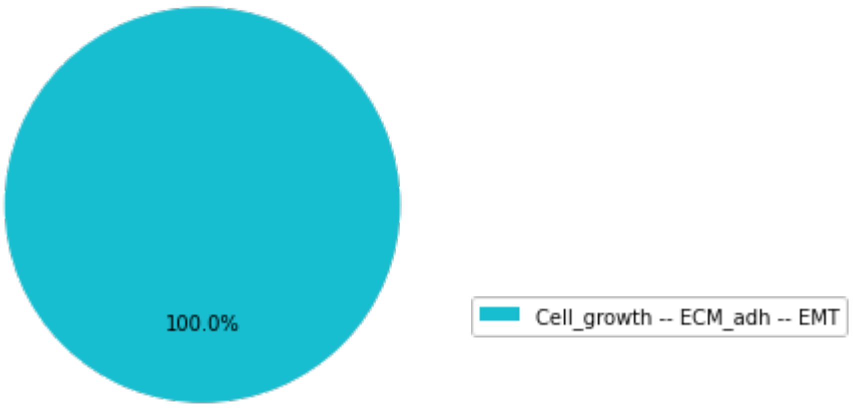

(('ERK', 'ON'), ('p53', 'OFF'))

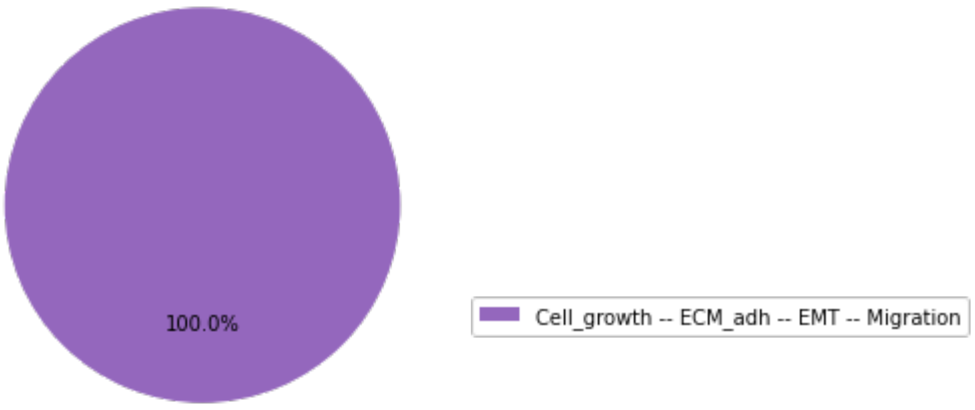

(('ERK', 'ON'), ('p63', 'OFF'))

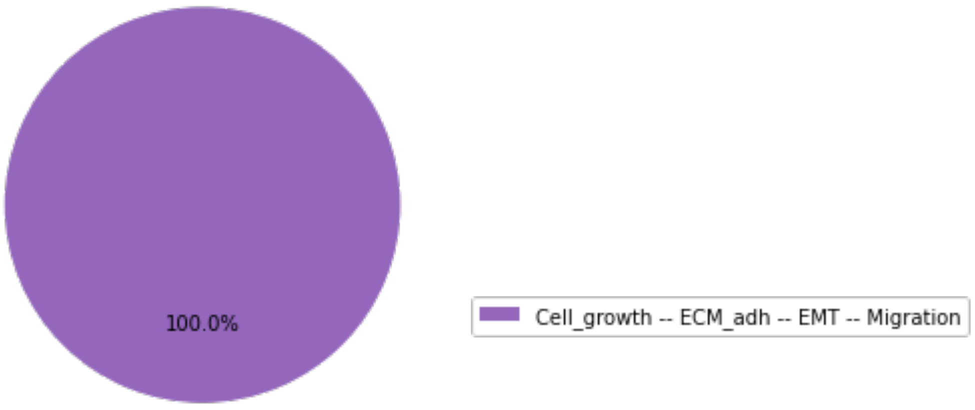

(('ERK', 'ON'), ('p73', 'OFF'))

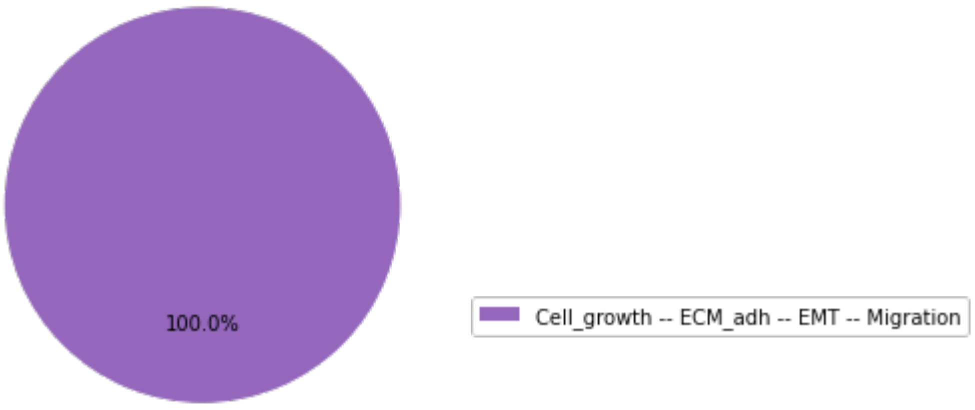

(('ERK', 'ON'), ('miR203', 'OFF'))

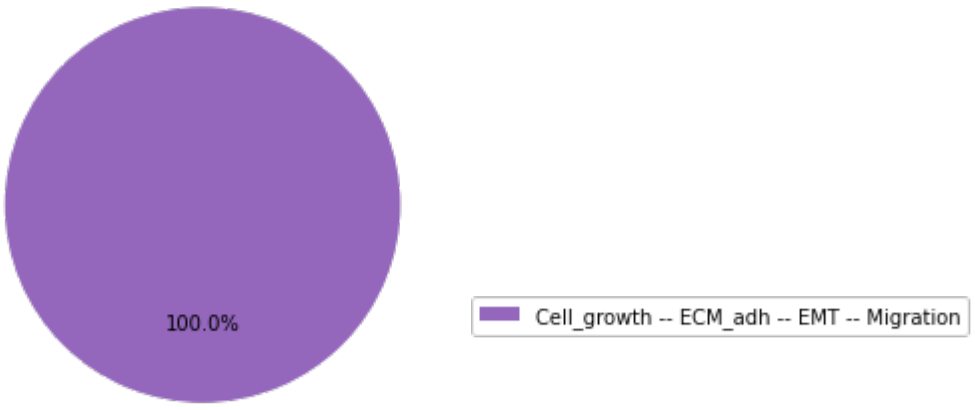

(('ERK', 'ON'), ('miR34', 'OFF'))

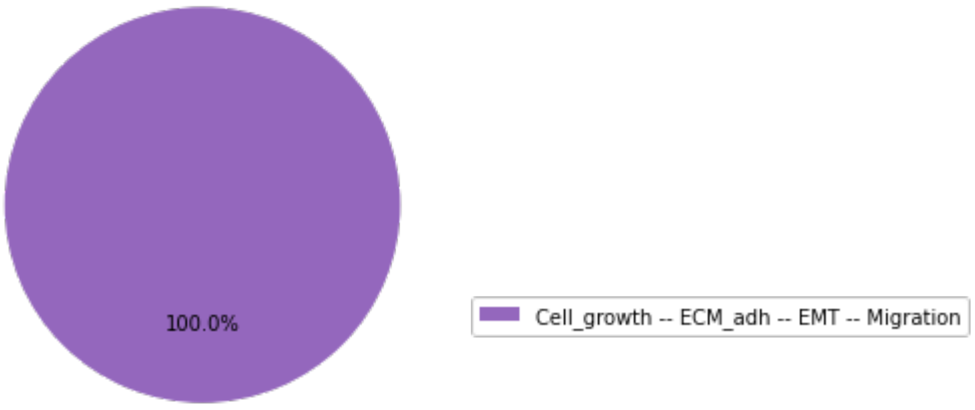

(('ERK', 'ON'), ('miR200', 'OFF'))

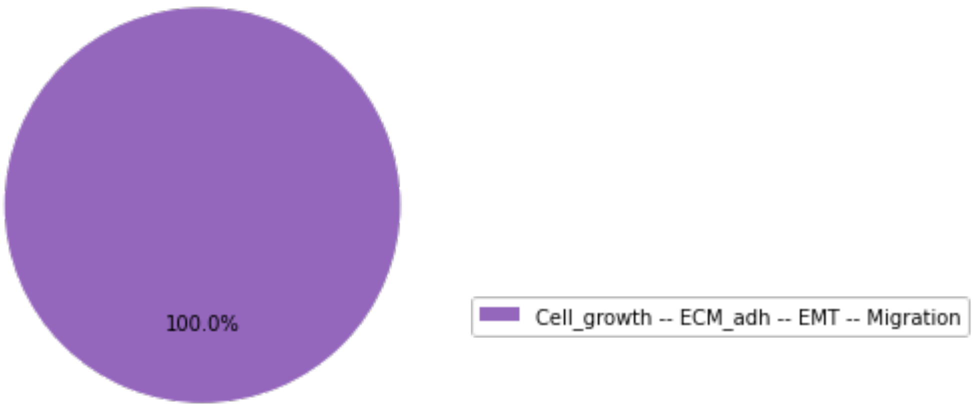

(('ERK', 'ON'), ('TGFbetaR', 'OFF'))

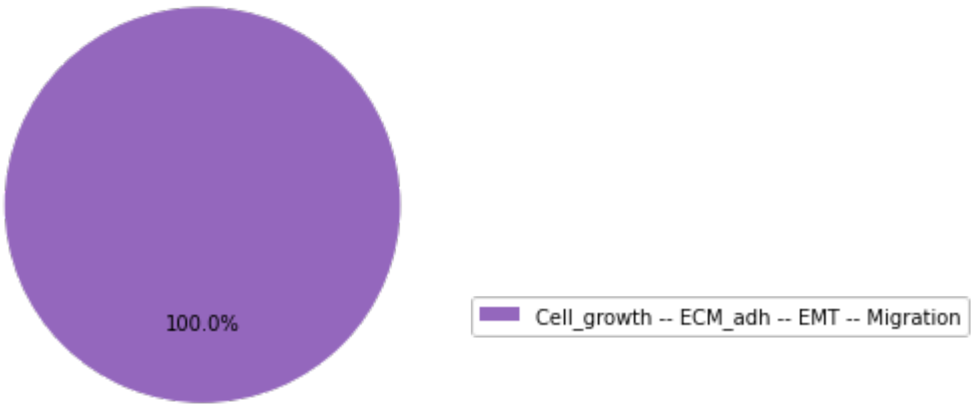

(('ERK', 'ON'), ('FAK', 'OFF'))

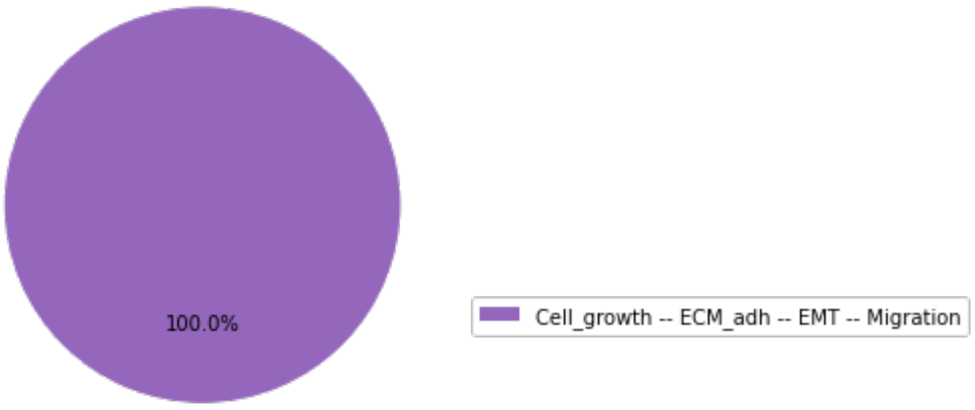

(('p21', 'ON'), ('SMAD', 'ON'))

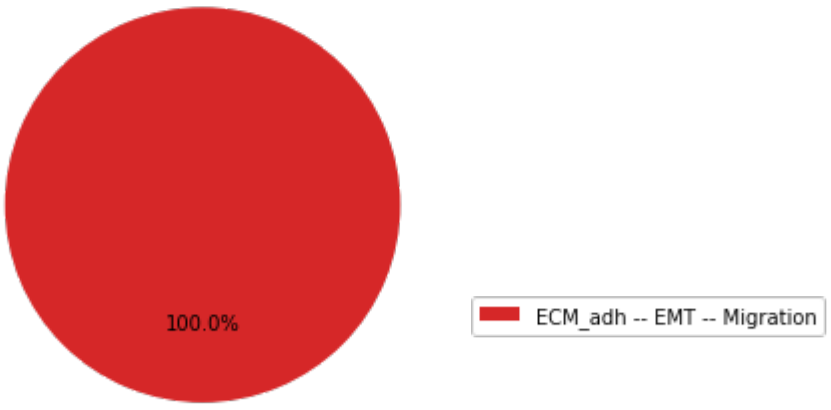

(('p21', 'ON'), ('VIM', 'ON'))

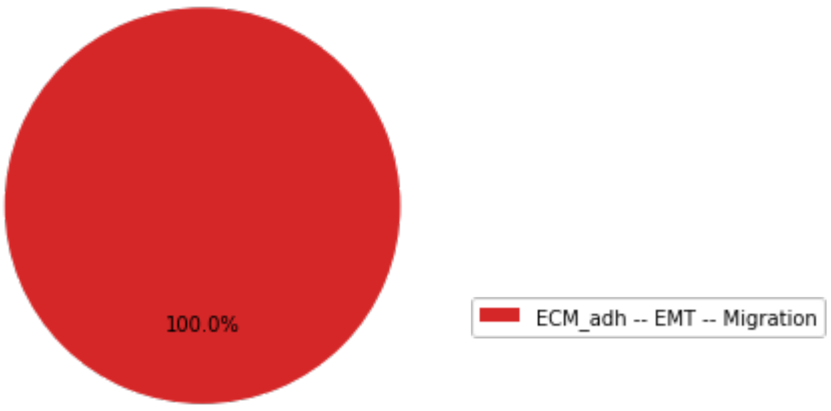

(('p21', 'ON'), ('p53', 'ON'))

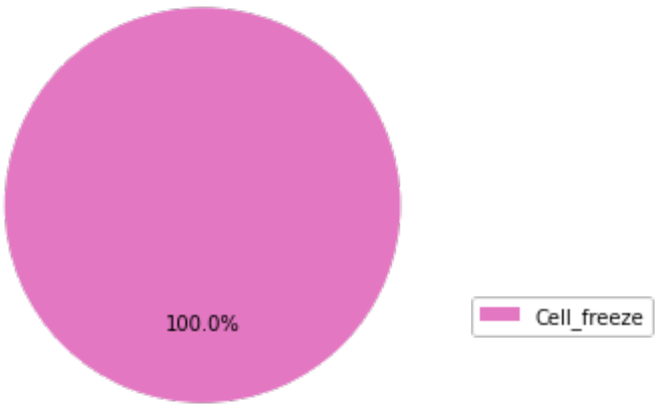

(('p21', 'ON'), ('p63', 'ON'))

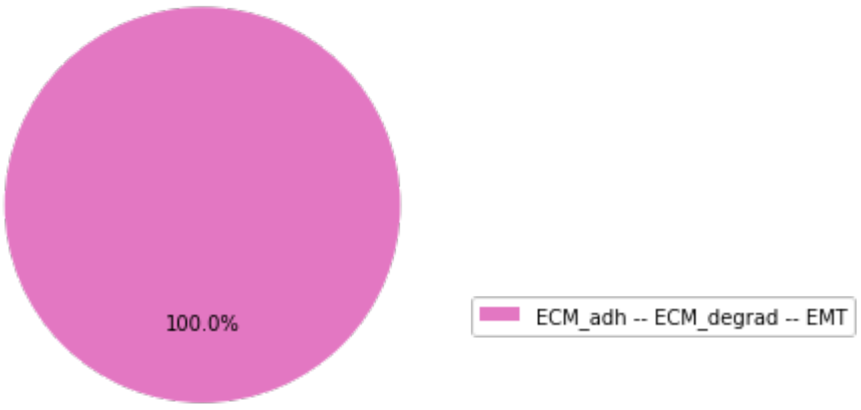

(('p21', 'ON'), ('p73', 'ON'))

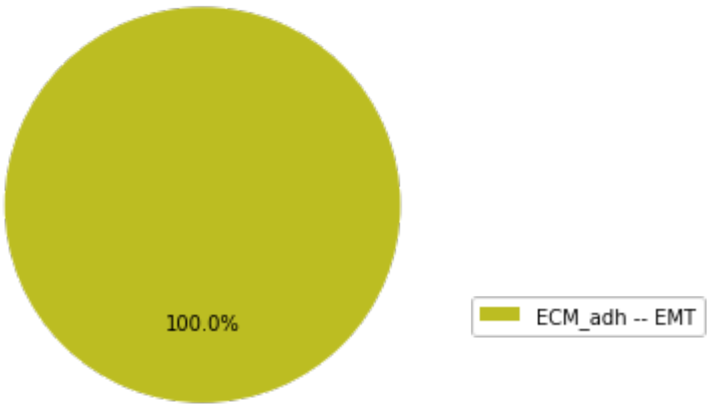

(('p21', 'ON'), ('miR203', 'ON'))

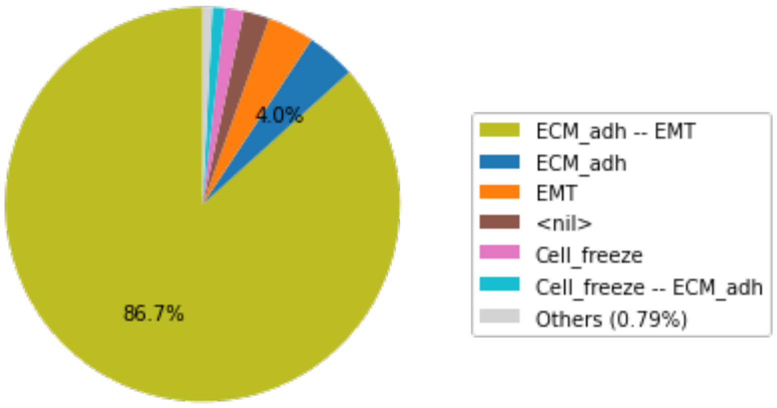

(('p21', 'ON'), ('miR34', 'ON'))

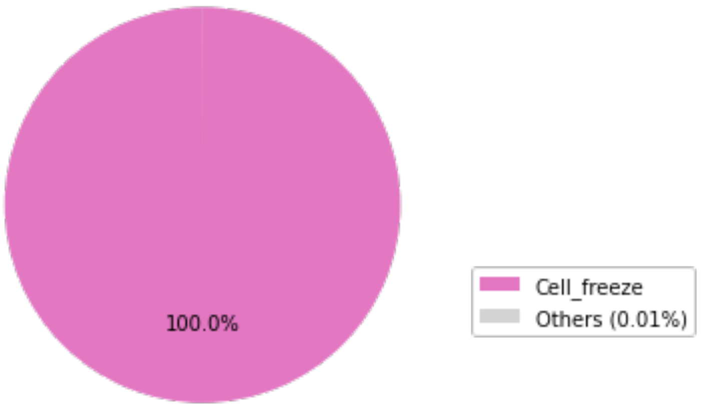

(('p21', 'ON'), ('miR200', 'ON'))

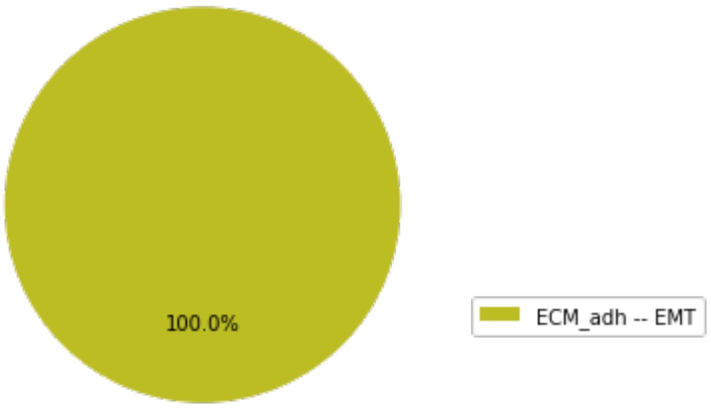

(('p21', 'ON'), ('TGFbetaR', 'ON'))

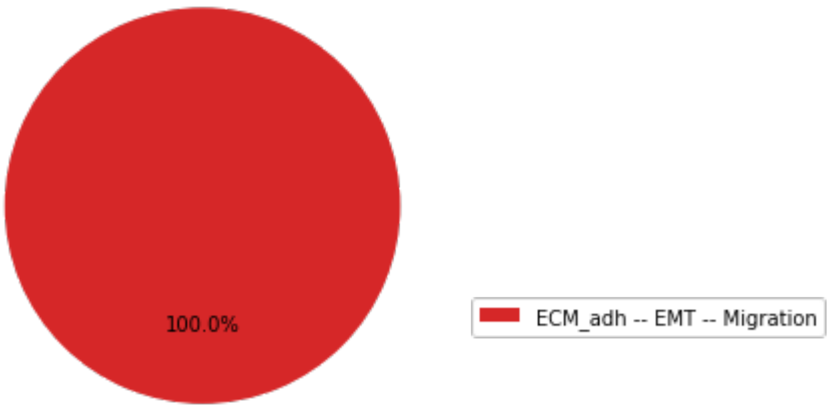

(('p21', 'ON'), ('FAK', 'ON'))

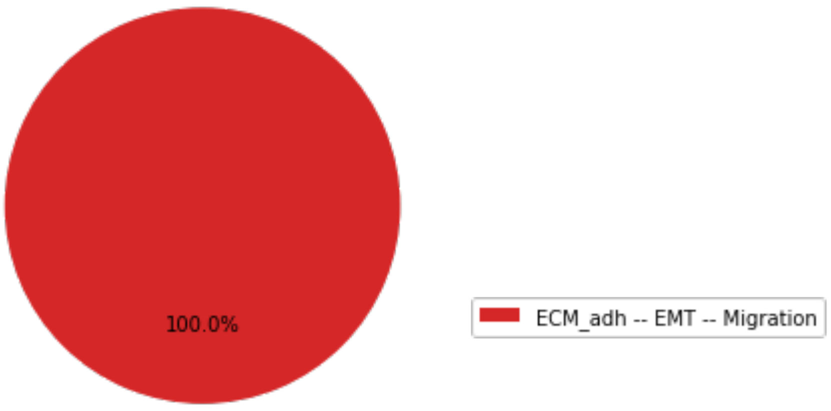

(('p21', 'ON'), ('AKT1', 'OFF'))

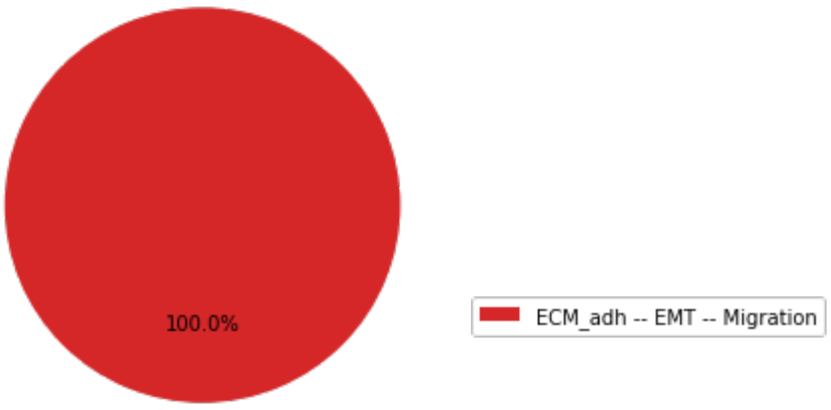

(('p21', 'ON'), ('CTNNB1', 'OFF'))

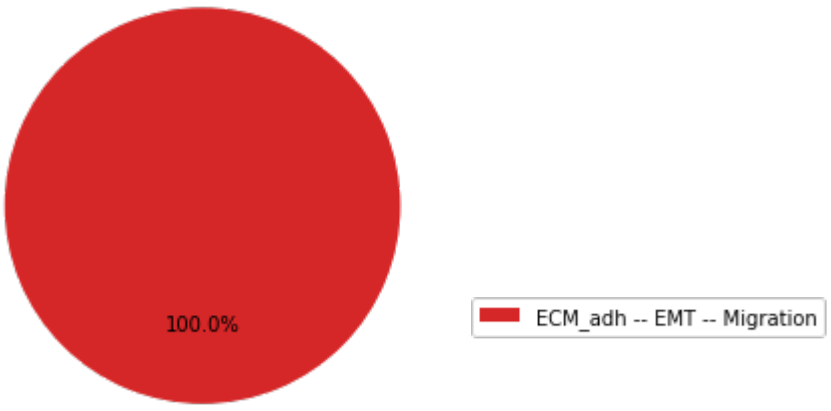

(('p21', 'ON'), ('DKK1', 'OFF'))

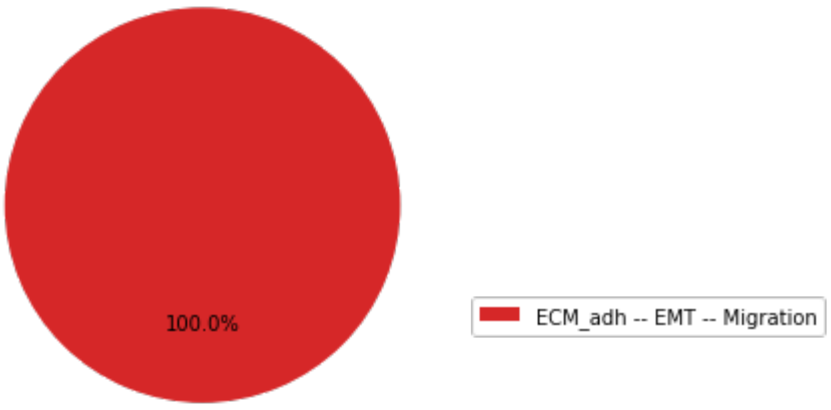

(('p21', 'ON'), ('AKT2', 'OFF'))

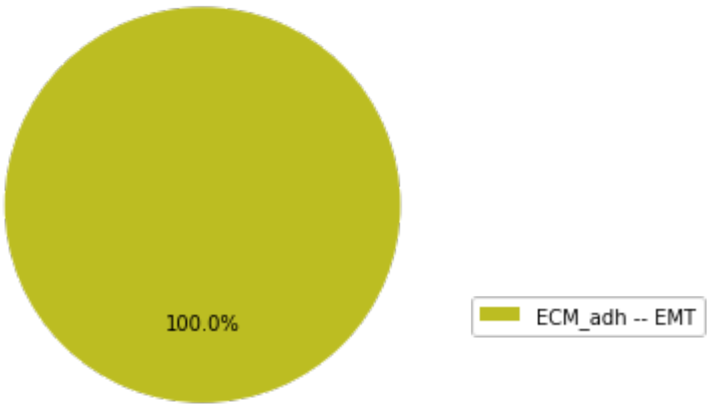

(('p21', 'ON'), ('RAC1', 'OFF'))

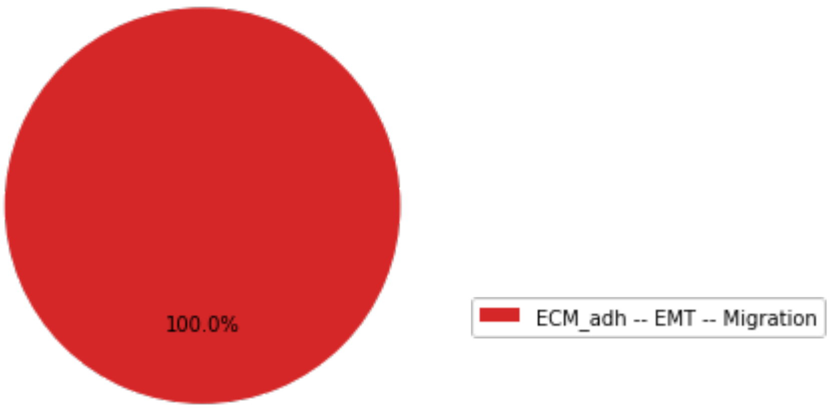

(('p21', 'ON'), ('YAP1', 'OFF'))

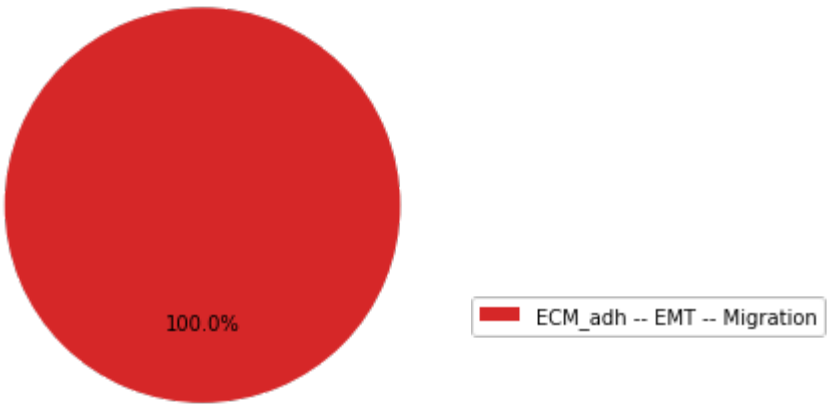

(('p21', 'ON'), ('PIK3CA', 'OFF'))

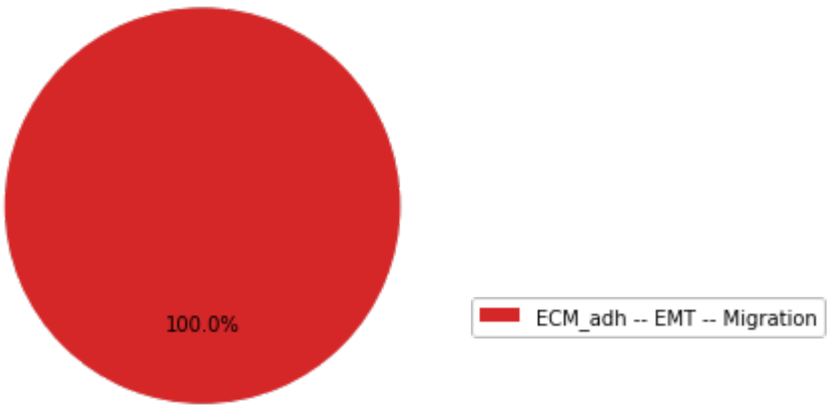

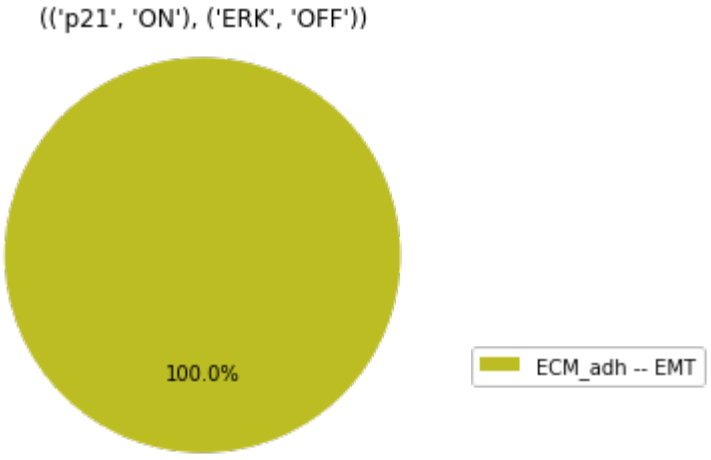
(('p21', 'ON'), ('ERK', 'OFF'))
100.0%
ECM_adh -- EMT

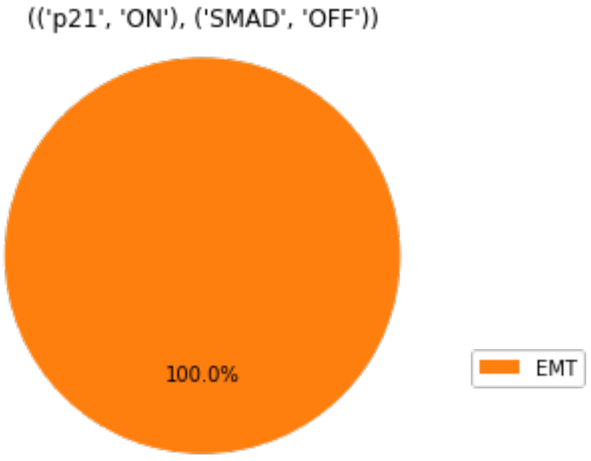
(('p21', 'ON'), ('SMAD', 'OFF'))
100.0%
EMT

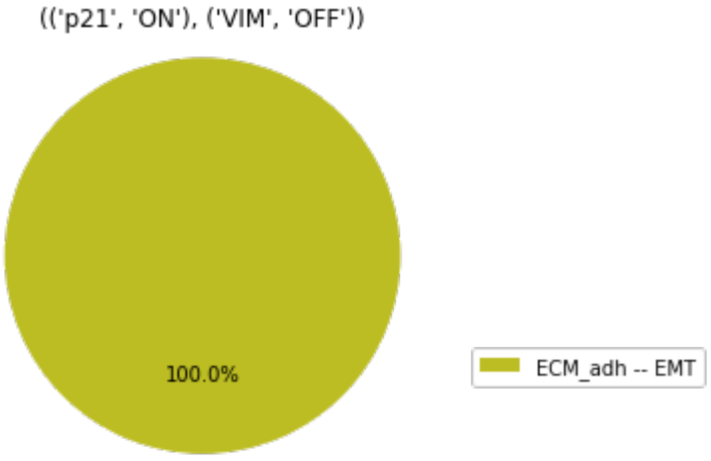
(('p21', 'ON'), ('VIM', 'OFF'))
100.0%
ECM_adh -- EMT

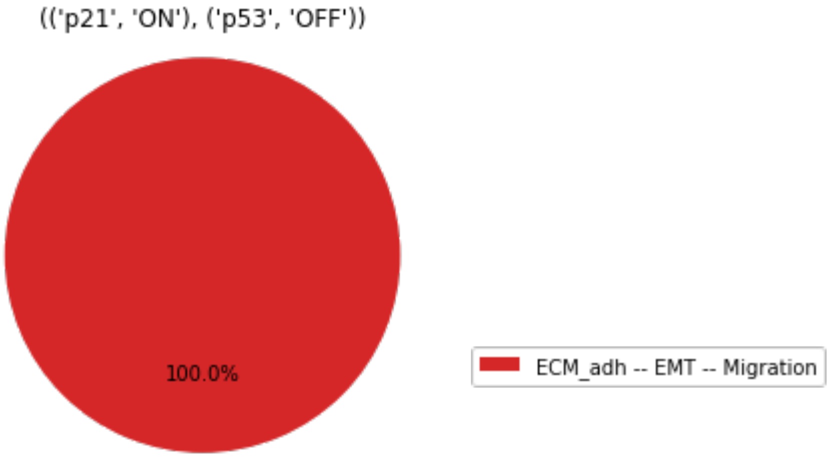
(('p21', 'ON'), ('p53', 'OFF'))
100.0%
ECM_adh -- EMT -- Migration

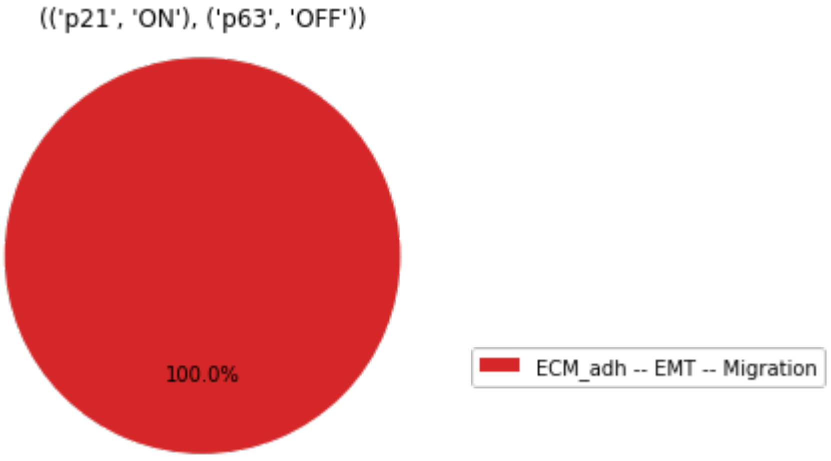
(('p21', 'ON'), ('p63', 'OFF'))
100.0%
ECM_adh -- EMT -- Migration

(('p21', 'ON'), ('p73', 'OFF'))

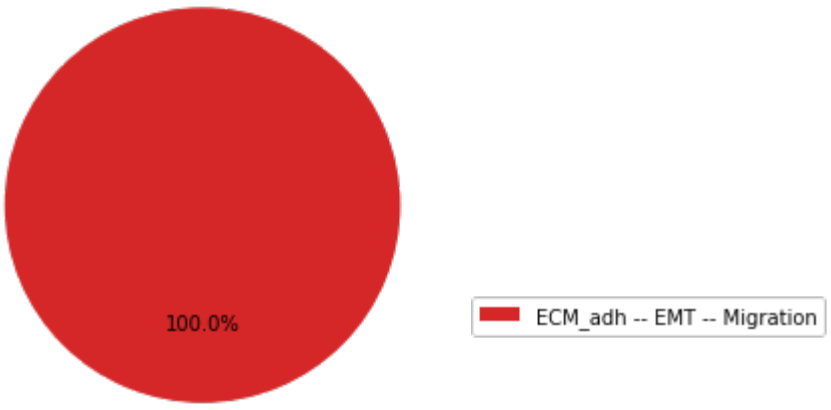

(('p21', 'ON'), ('miR203', 'OFF'))

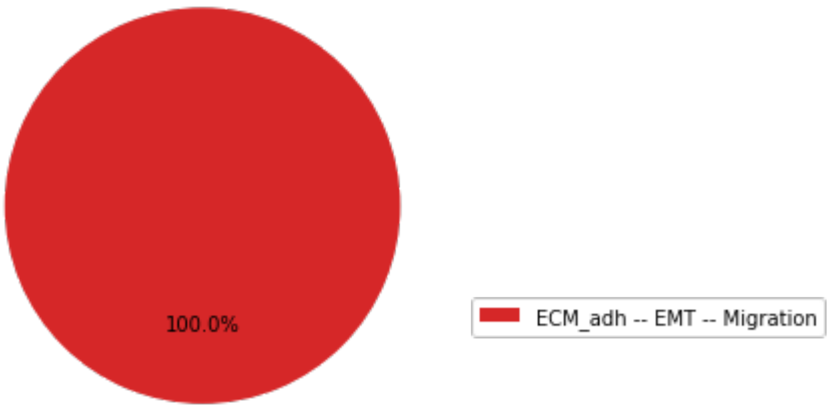

(('p21', 'ON'), ('miR34', 'OFF'))

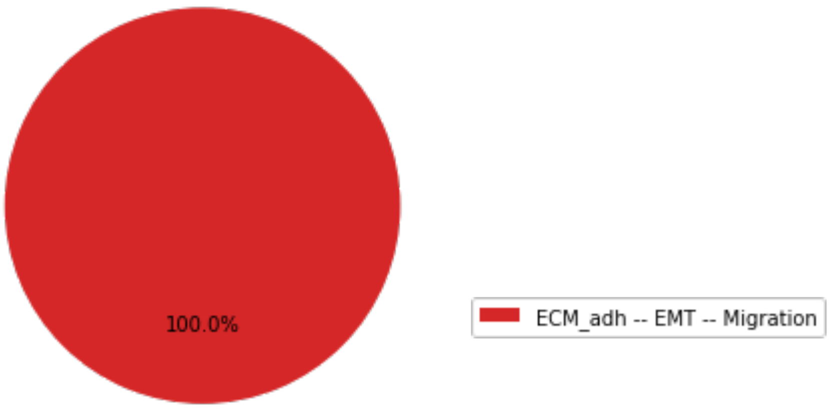

(('p21', 'ON'), ('miR200', 'OFF'))

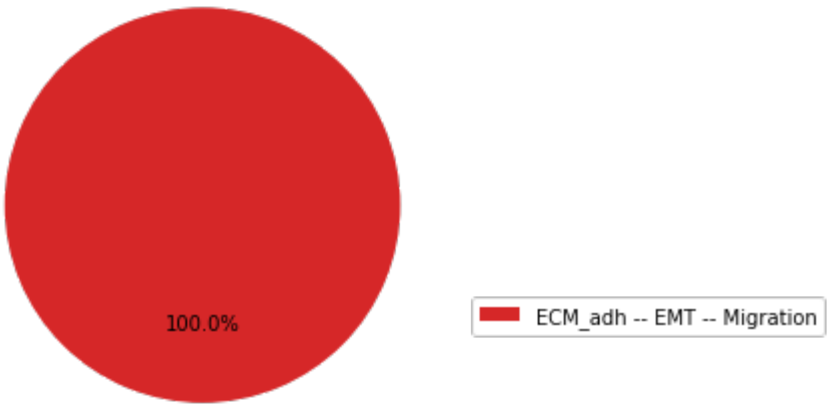

(('p21', 'ON'), ('TGFbetaR', 'OFF'))

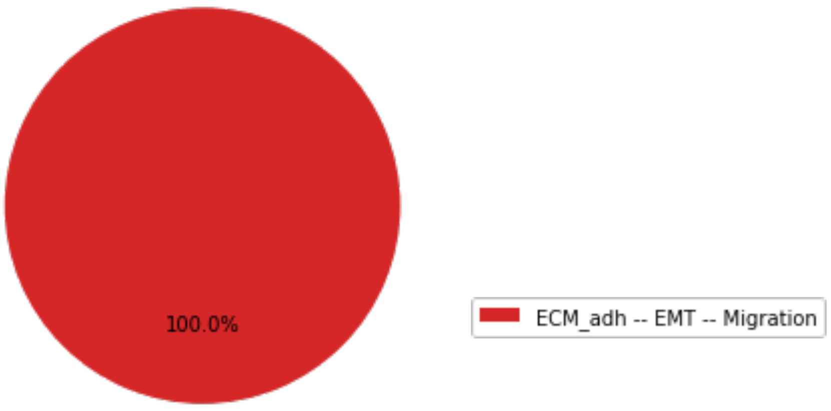

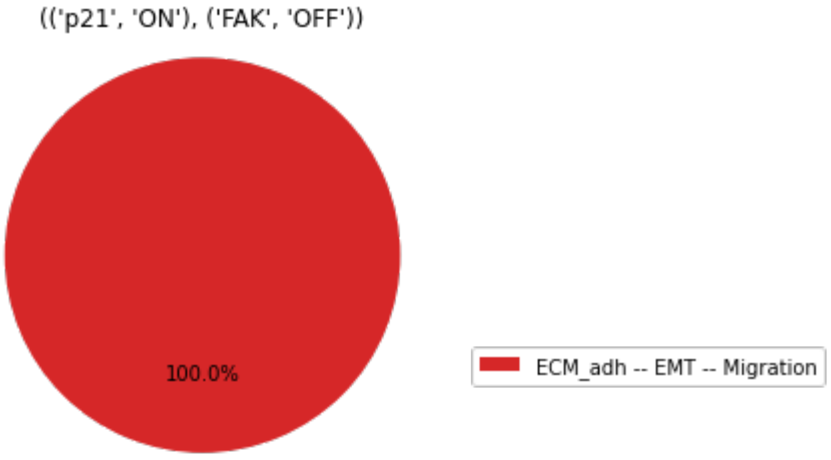
(('p21', 'ON'), ('FAK', 'OFF'))
100.0%
ECM_adh -- EMT -- Migration

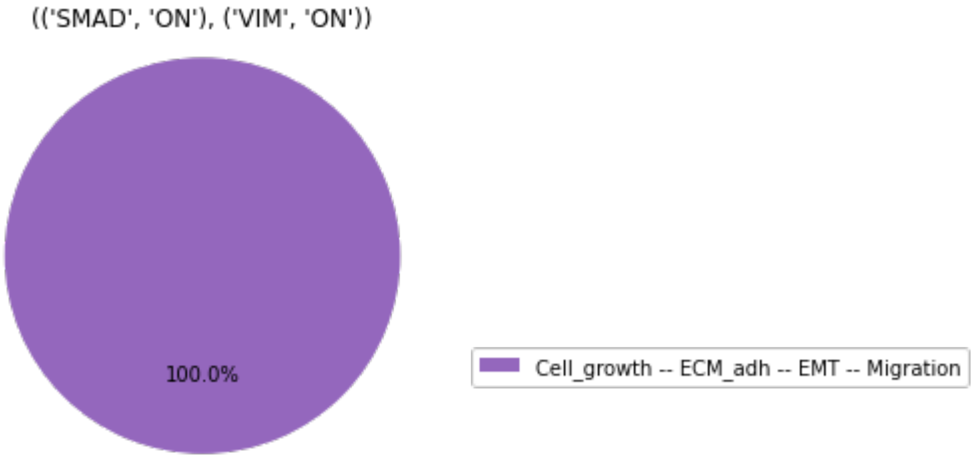
(('SMAD', 'ON'), ('VIM', 'ON'))
100.0%
Cell_growth -- ECM_adh -- EMT -- Migration

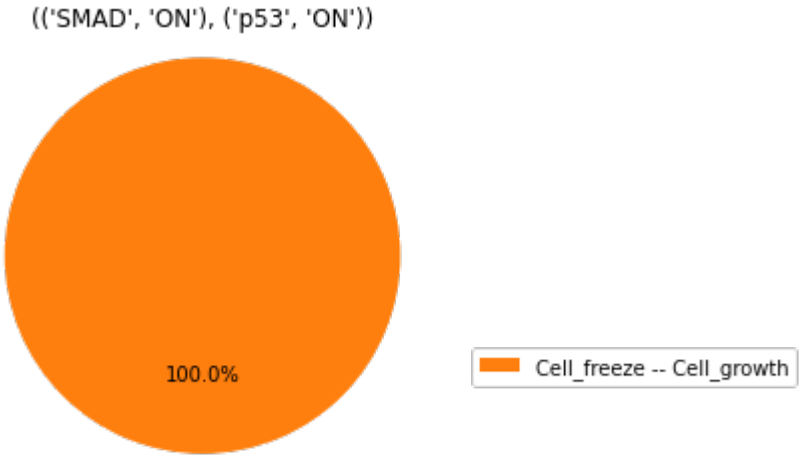
(('SMAD', 'ON'), ('p53', 'ON'))
100.0%
Cell_freeze -- Cell_growth

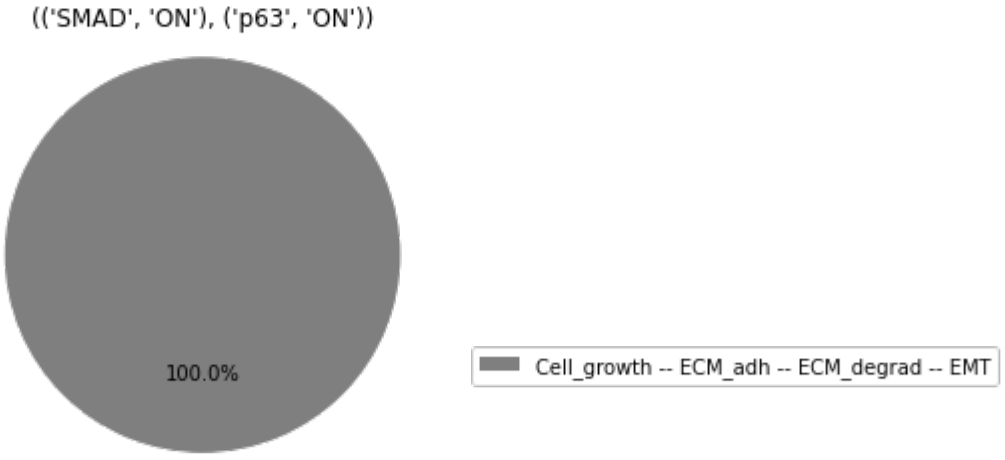
(('SMAD', 'ON'), ('p63', 'ON'))
100.0%
Cell_growth -- ECM_adh -- ECM_degrad -- EMT

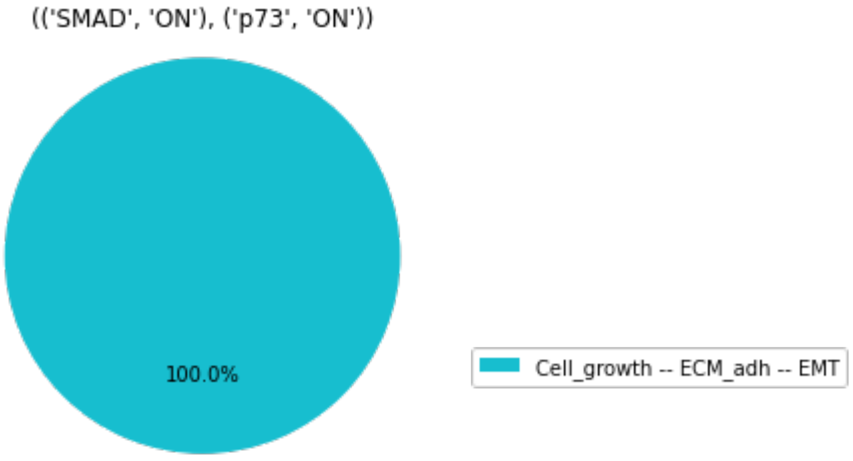
(('SMAD', 'ON'), ('p73', 'ON'))
100.0%
Cell_growth -- ECM_adh -- EMT

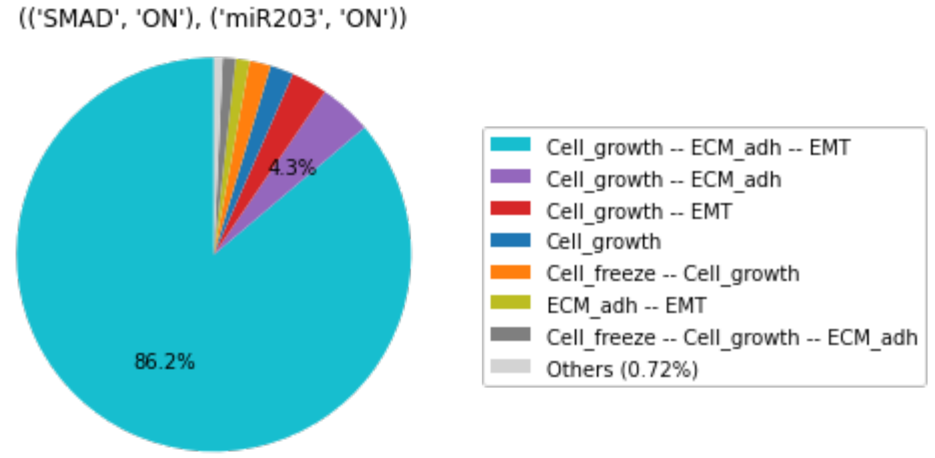
(('SMAD', 'ON'), ('miR203', 'ON'))
4.3%
86.2%
Cell_growth -- ECM_adh -- EMT
Cell_growth -- ECM_adh
Cell_growth -- EMT
Cell_growth
Cell_freeze -- Cell_growth
ECM_adh -- EMT
Cell_freeze -- Cell_growth -- ECM_adh
Others (0.72%)

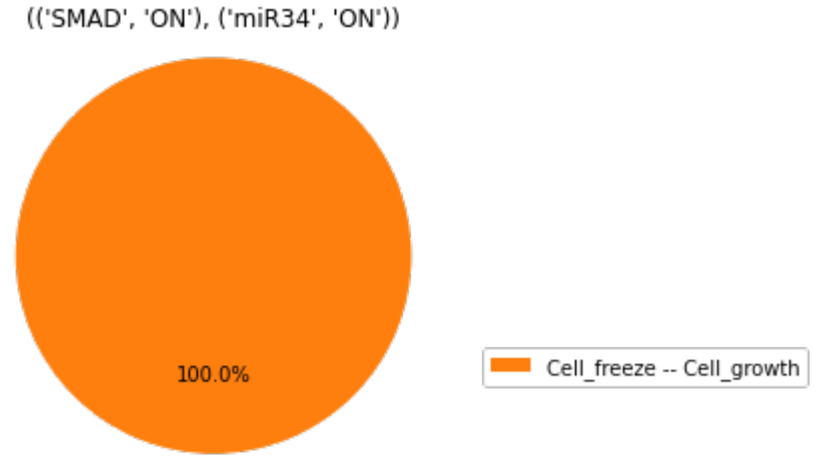
(('SMAD', 'ON'), ('miR34', 'ON'))
100.0%
Cell_freeze -- Cell_growth

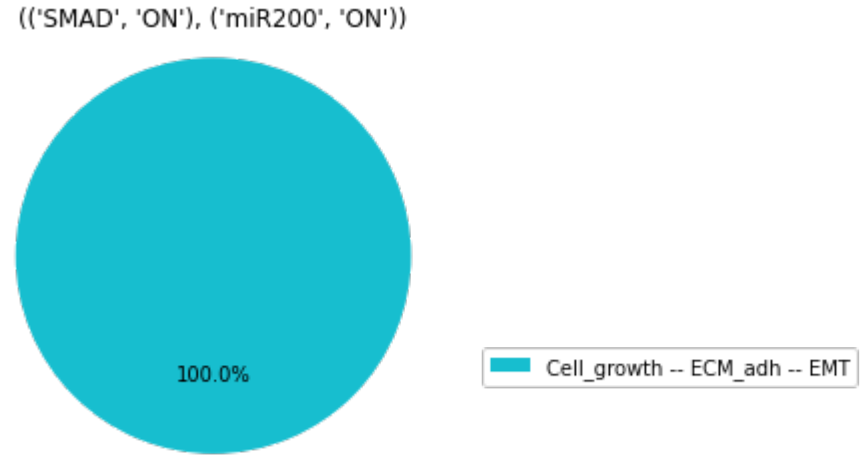
(('SMAD', 'ON'), ('miR200', 'ON'))
100.0%
Cell_growth -- ECM_adh -- EMT

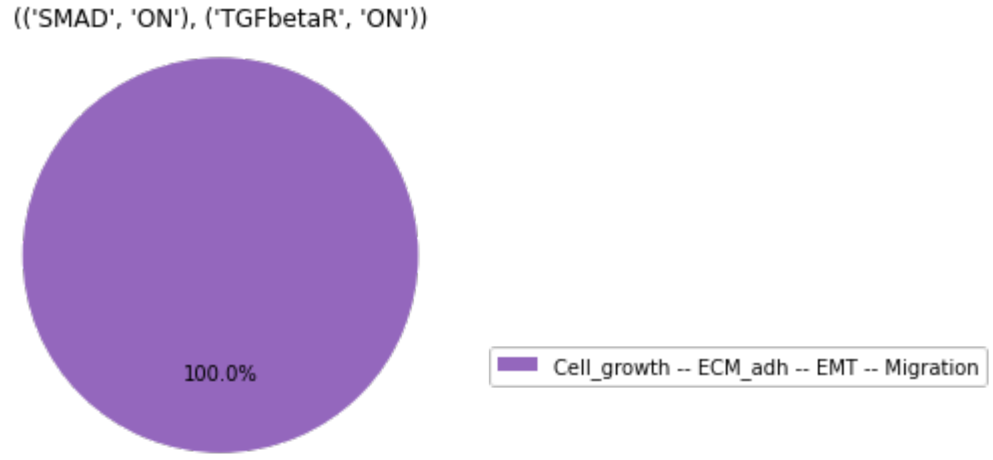
(('SMAD', 'ON'), ('TGFbetaR', 'ON'))
100.0%
Cell_growth -- ECM_adh -- EMT -- Migration

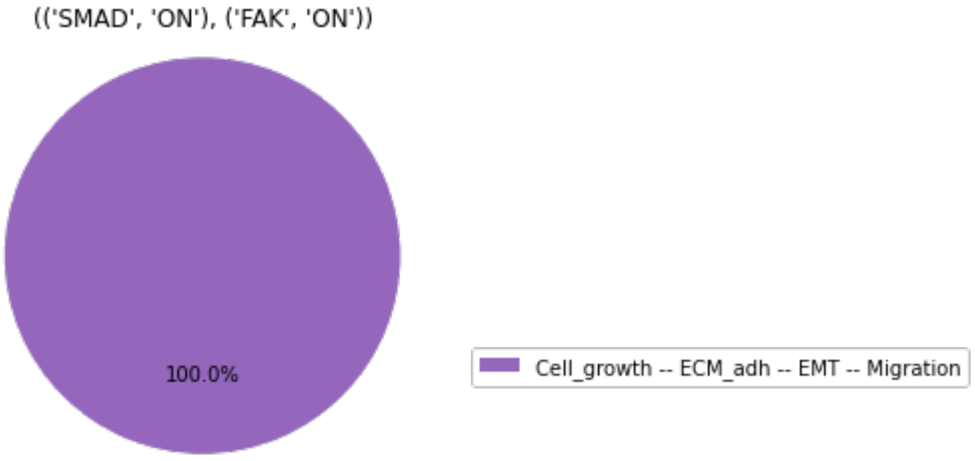
(('SMAD', 'ON'), ('FAK', 'ON'))
100.0%
Cell_growth -- ECM_adh -- EMT -- Migration

(('SMAD', 'ON'), ('AKT1', 'OFF'))

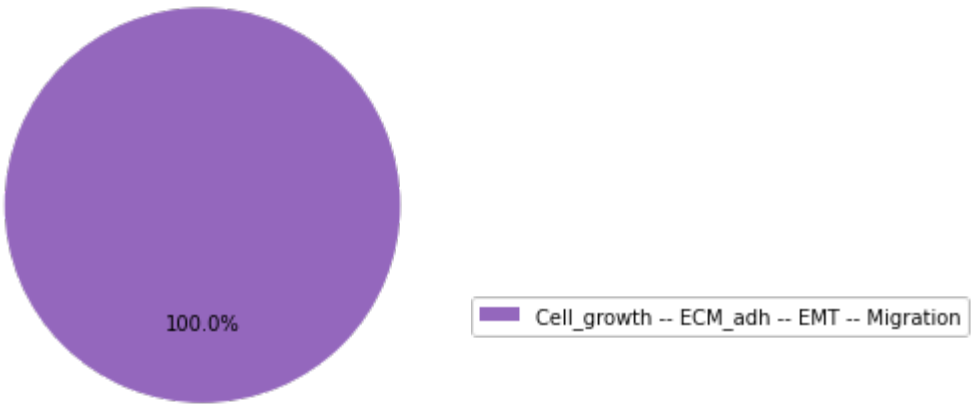

(('SMAD', 'ON'), ('CTNNB1', 'OFF'))

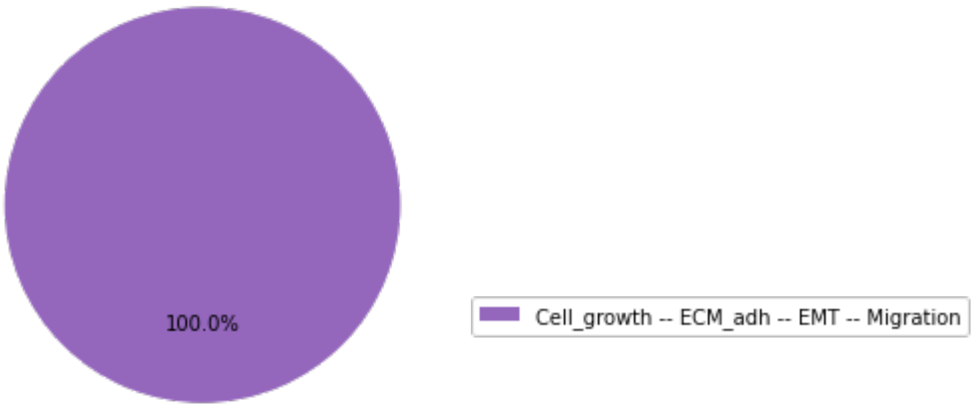

(('SMAD', 'ON'), ('DKK1', 'OFF'))

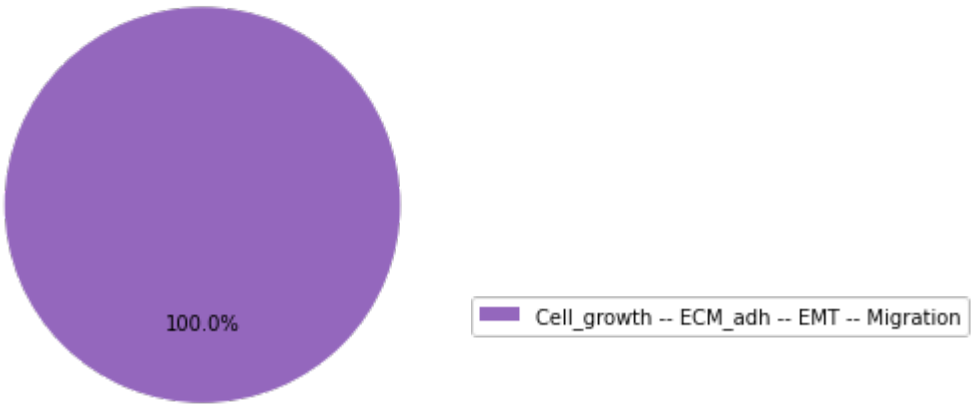

(('SMAD', 'ON'), ('AKT2', 'OFF'))

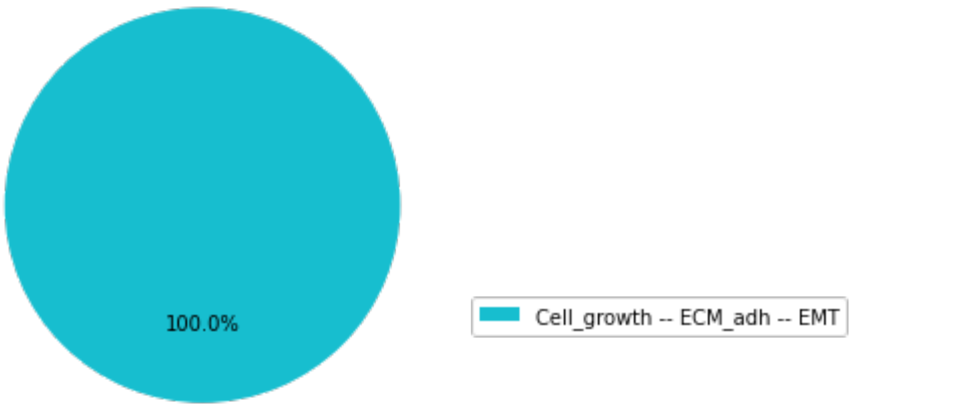

(('SMAD', 'ON'), ('RAC1', 'OFF'))

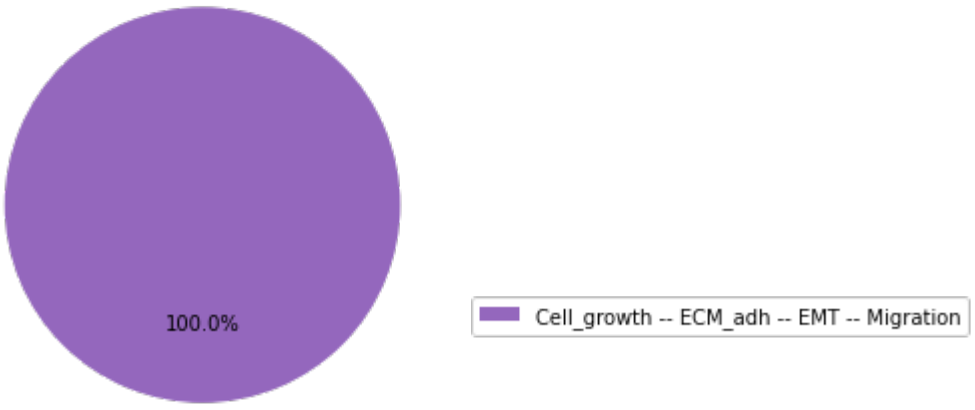

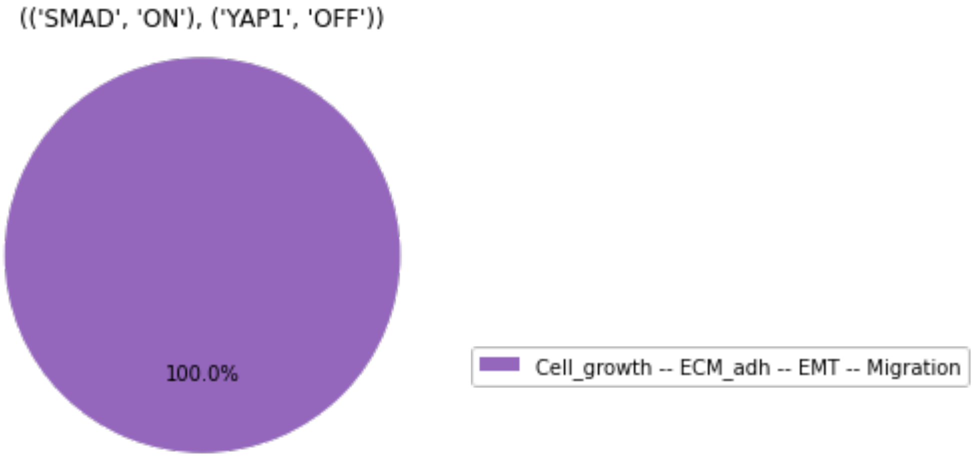
(('SMAD', 'ON'), ('YAP1', 'OFF'))
100.0%
Cell_growth -- ECM_adh -- EMT -- Migration

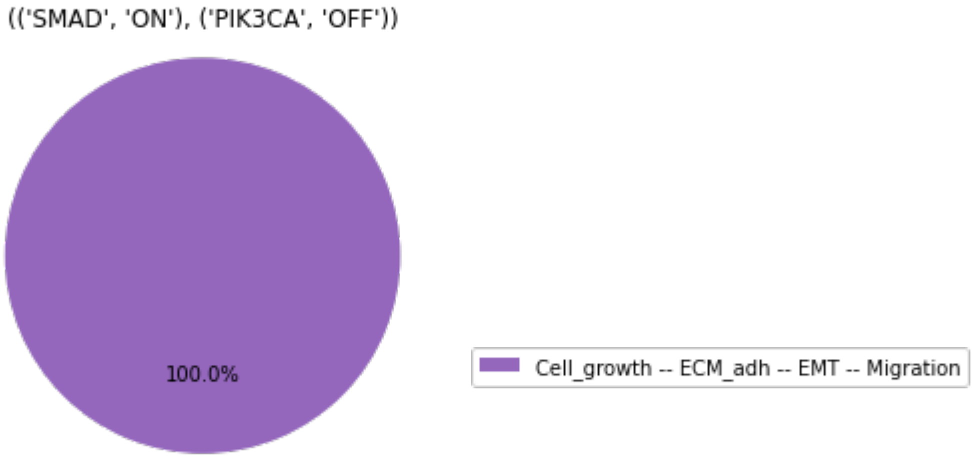
(('SMAD', 'ON'), ('PIK3CA', 'OFF'))
100.0%
Cell_growth -- ECM_adh -- EMT -- Migration

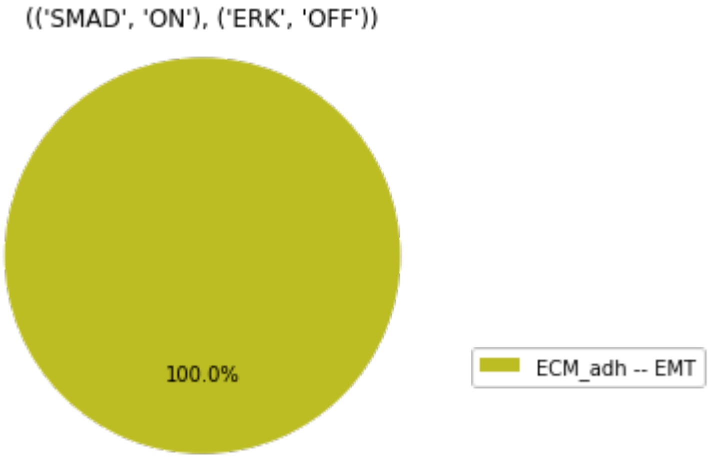
(('SMAD', 'ON'), ('ERK', 'OFF'))
100.0%
ECM_adh -- EMT

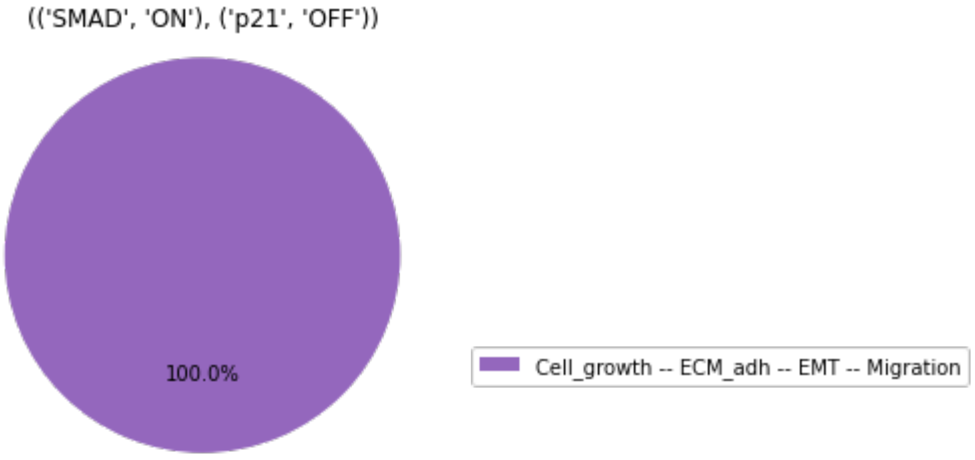
(('SMAD', 'ON'), ('p21', 'OFF'))
100.0%
Cell_growth -- ECM_adh -- EMT -- Migration

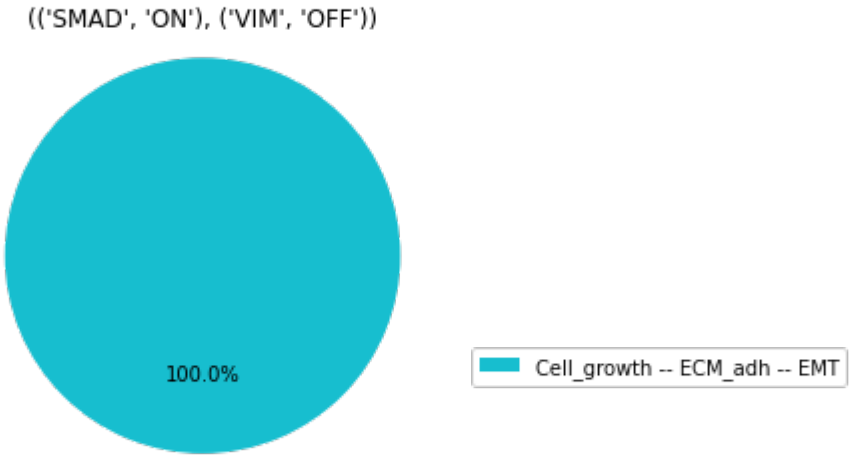
(('SMAD', 'ON'), ('VIM', 'OFF'))
100.0%
Cell_growth -- ECM_adh -- EMT

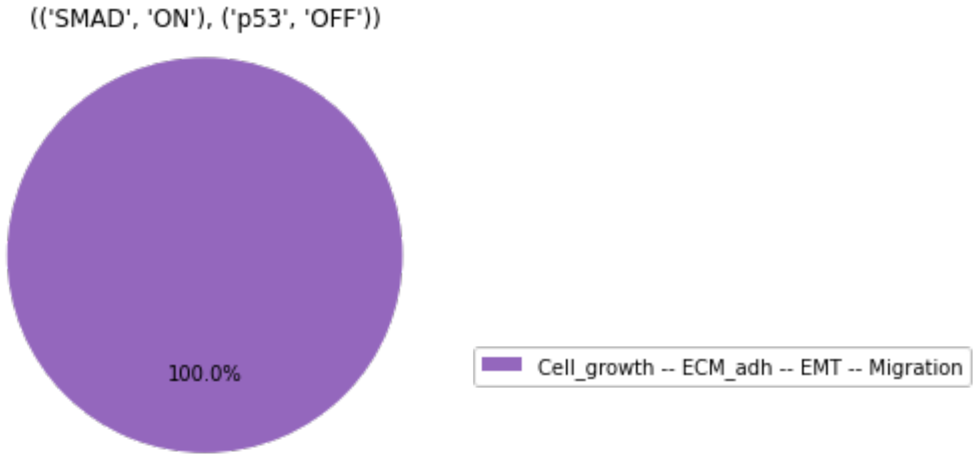
(('SMAD', 'ON'), ('p53', 'OFF'))
100.0%
Cell_growth -- ECM_adh -- EMT -- Migration

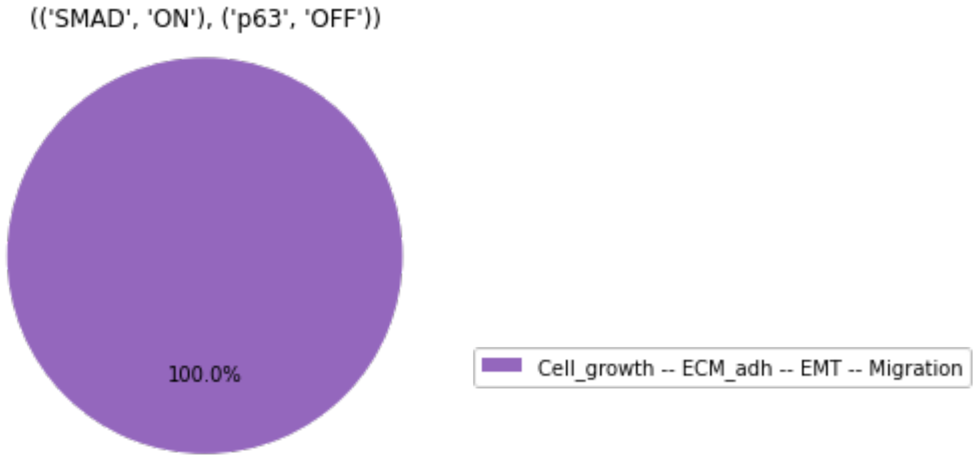
(('SMAD', 'ON'), ('p63', 'OFF'))
100.0%
Cell_growth -- ECM_adh -- EMT -- Migration

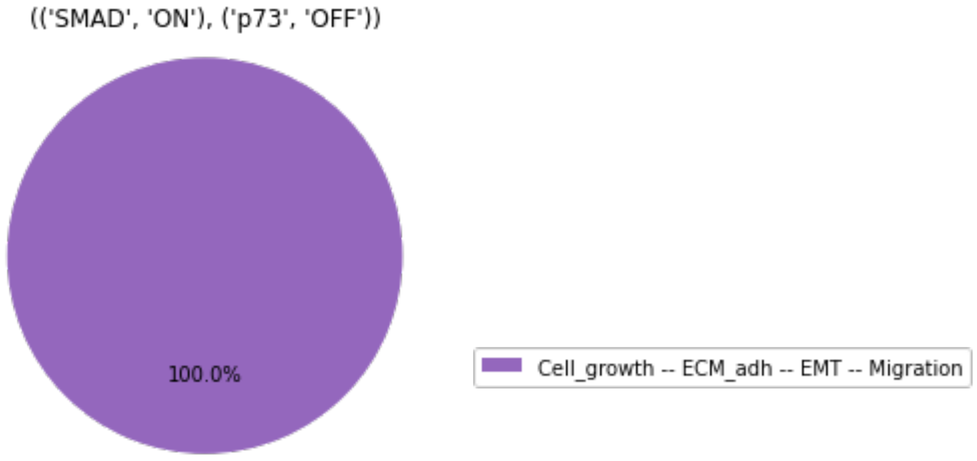
(('SMAD', 'ON'), ('p73', 'OFF'))
100.0%
Cell_growth -- ECM_adh -- EMT -- Migration

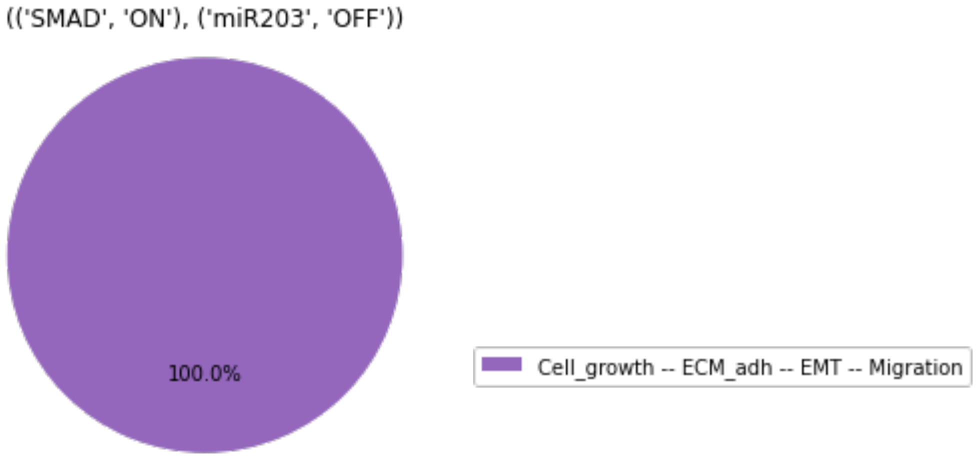
(('SMAD', 'ON'), ('miR203', 'OFF'))
100.0%
Cell_growth -- ECM_adh -- EMT -- Migration

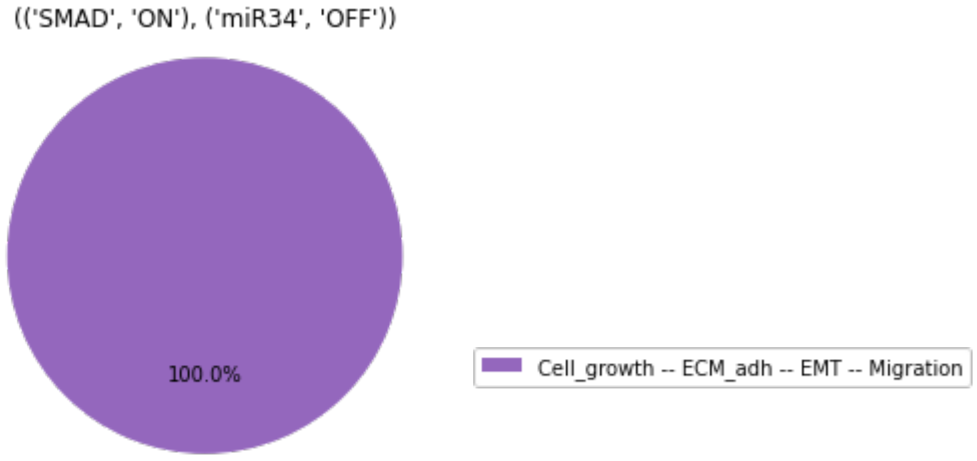
(('SMAD', 'ON'), ('miR34', 'OFF'))
100.0%
Cell_growth -- ECM_adh -- EMT -- Migration

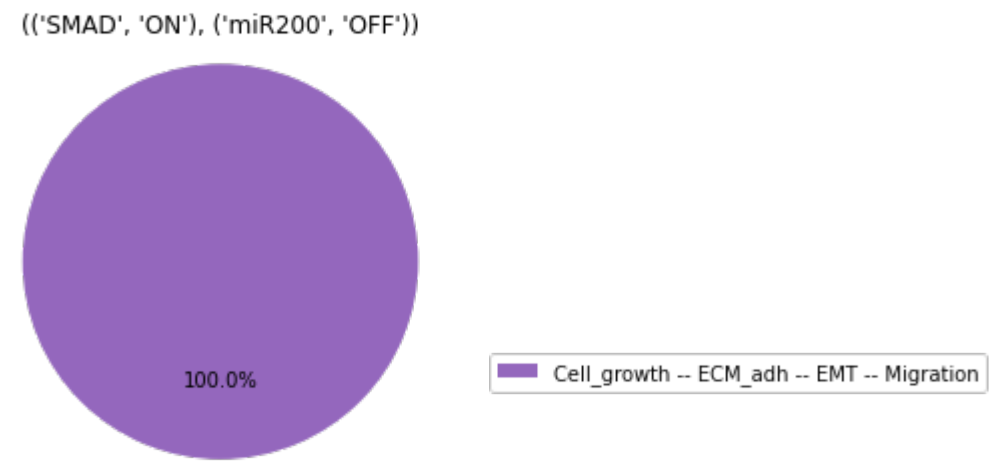
(('SMAD', 'ON'), ('miR200', 'OFF'))
100.0%
Cell_growth -- ECM_adh -- EMT -- Migration

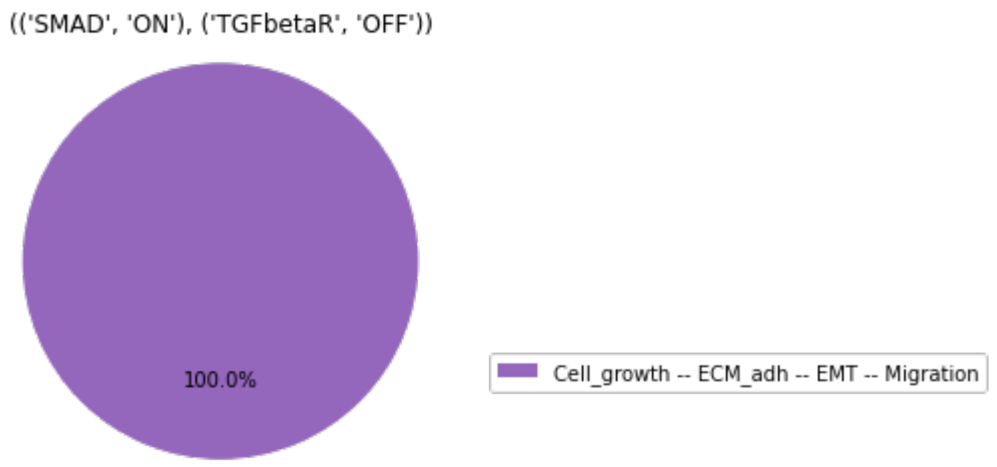
(('SMAD', 'ON'), ('TGFbetaR', 'OFF'))
100.0%
Cell_growth -- ECM_adh -- EMT -- Migration

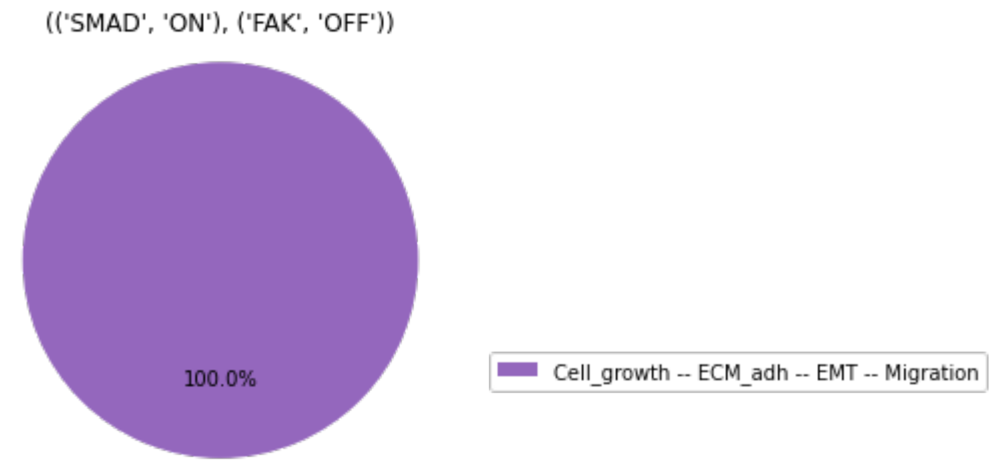
(('SMAD', 'ON'), ('FAK', 'OFF'))
100.0%
Cell_growth -- ECM_adh -- EMT -- Migration

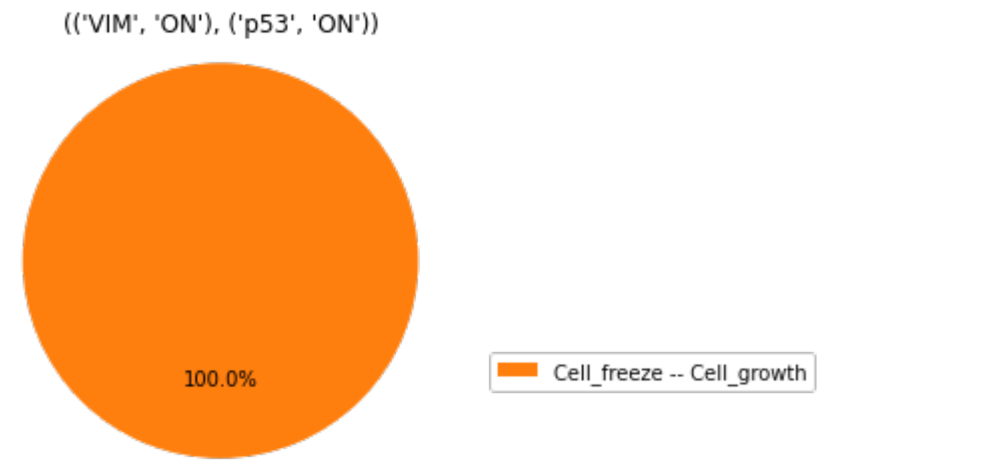
(('VIM', 'ON'), ('p53', 'ON'))
100.0%
Cell_freeze -- Cell_growth

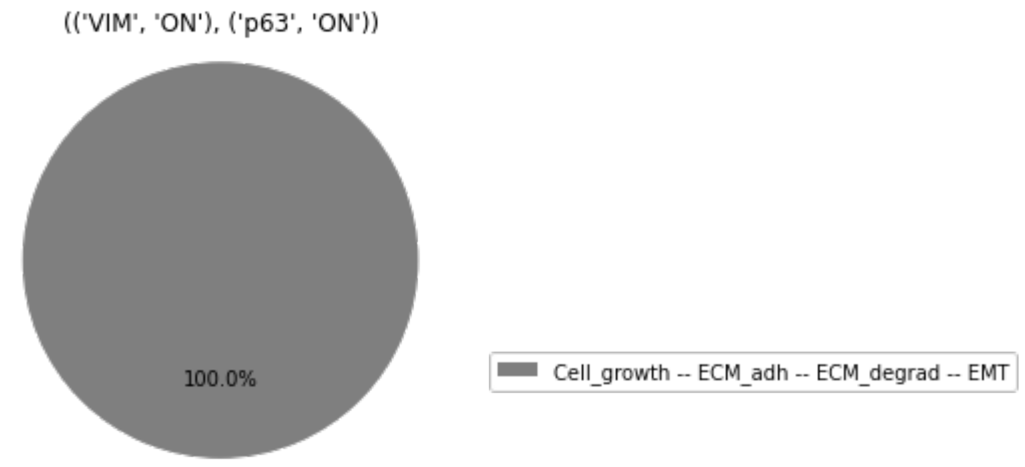
(('VIM', 'ON'), ('p63', 'ON'))
100.0%
Cell_growth -- ECM_adh -- ECM_degrad -- EMT

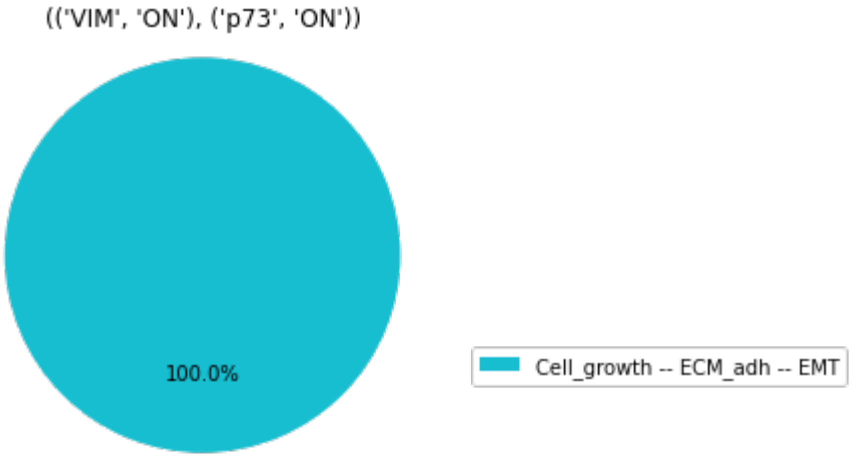
(('VIM', 'ON'), ('p73', 'ON'))
100.0%
Cell_growth -- ECM_adh -- EMT

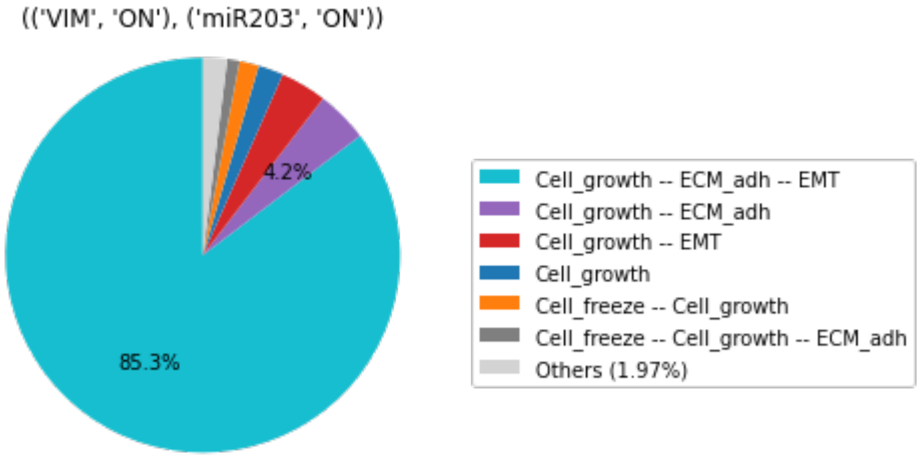
(('VIM', 'ON'), ('miR203', 'ON'))
4.2%
85.3%
Cell_growth -- ECM_adh -- EMT
Cell_growth -- ECM_adh
Cell_growth -- EMT
Cell_growth
Cell_freeze -- Cell_growth
Cell_freeze -- Cell_growth -- ECM_adh
Others (1.97%)

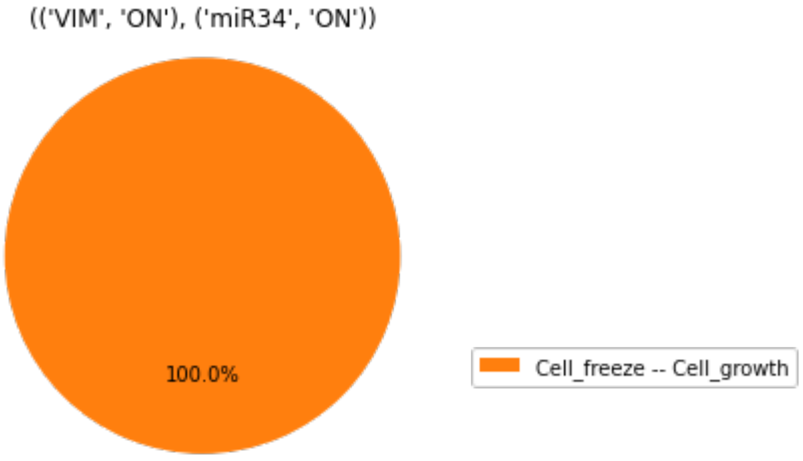
(('VIM', 'ON'), ('miR34', 'ON'))
100.0%
Cell_freeze -- Cell_growth

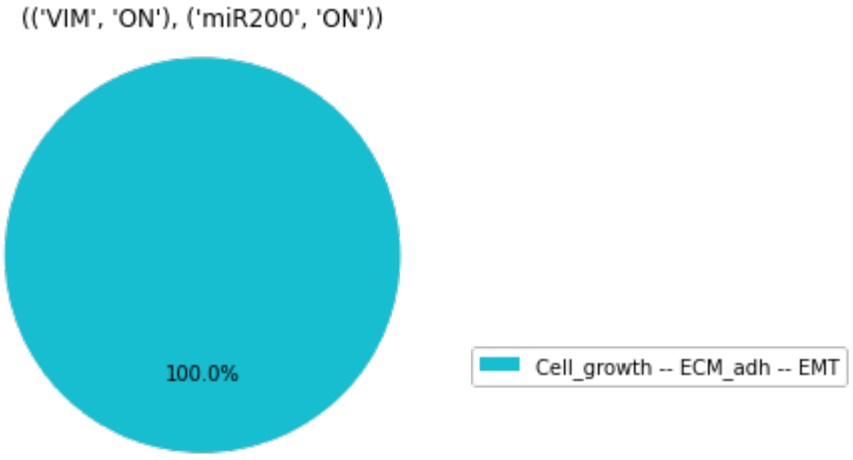
(('VIM', 'ON'), ('miR200', 'ON'))
100.0%
Cell_growth -- ECM_adh -- EMT

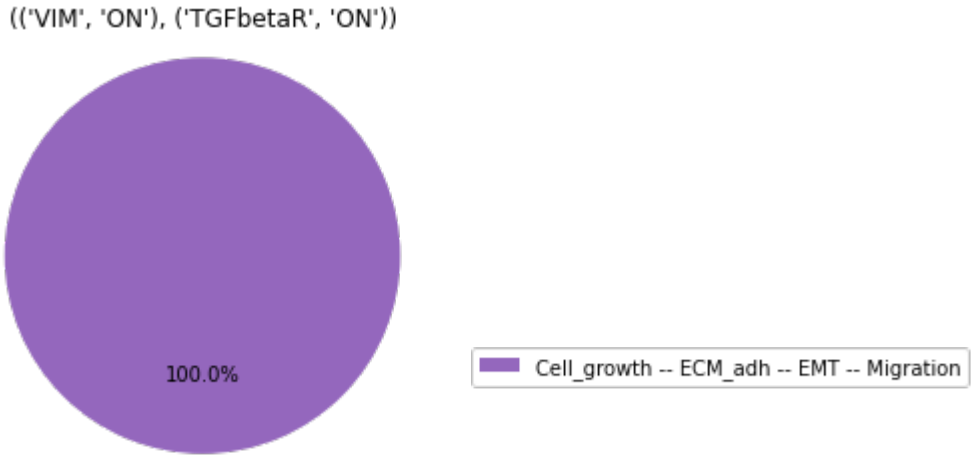
(('VIM', 'ON'), ('TGFbetaR', 'ON'))
100.0%
Cell_growth -- ECM_adh -- EMT -- Migration

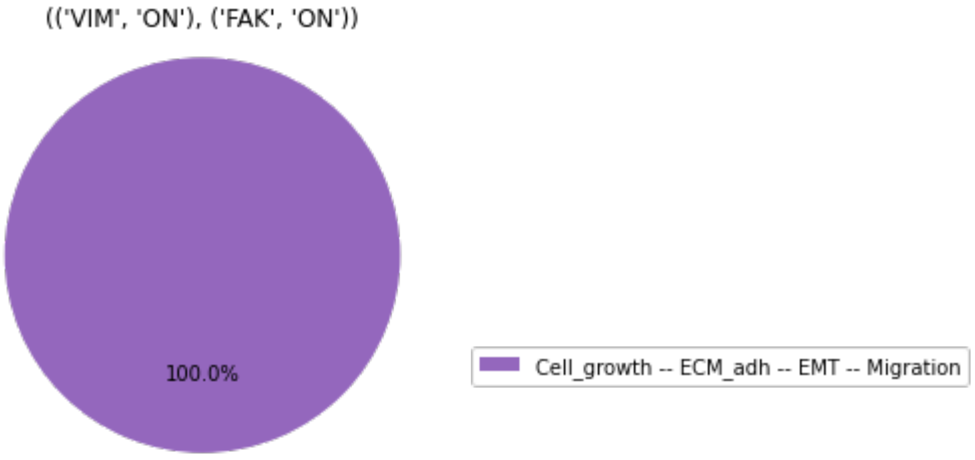
(('VIM', 'ON'), ('FAK', 'ON'))
100.0%
Cell_growth -- ECM_adh -- EMT -- Migration

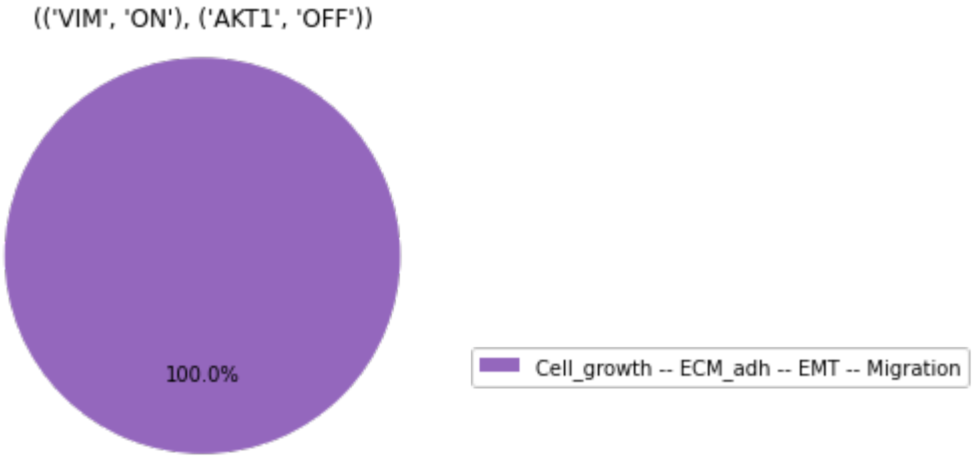
(('VIM', 'ON'), ('AKT1', 'OFF'))
100.0%
Cell_growth -- ECM_adh -- EMT -- Migration

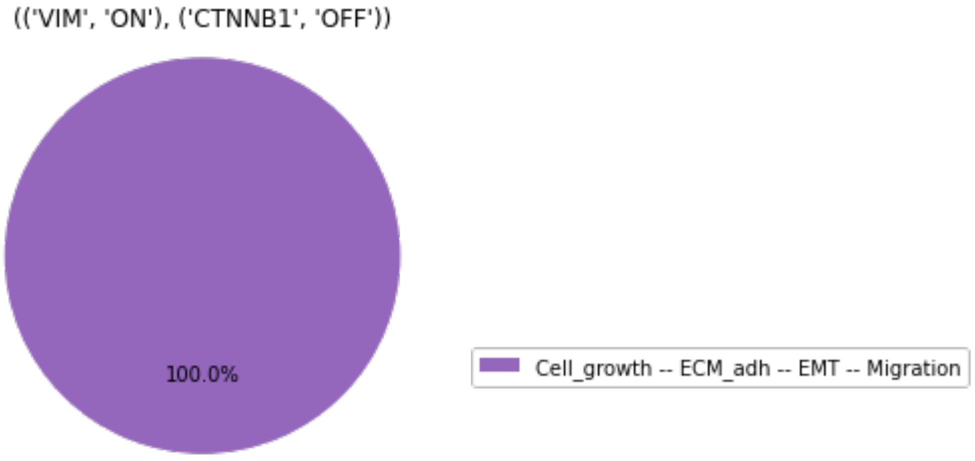
(('VIM', 'ON'), ('CTNNB1', 'OFF'))
100.0%
Cell_growth -- ECM_adh -- EMT -- Migration

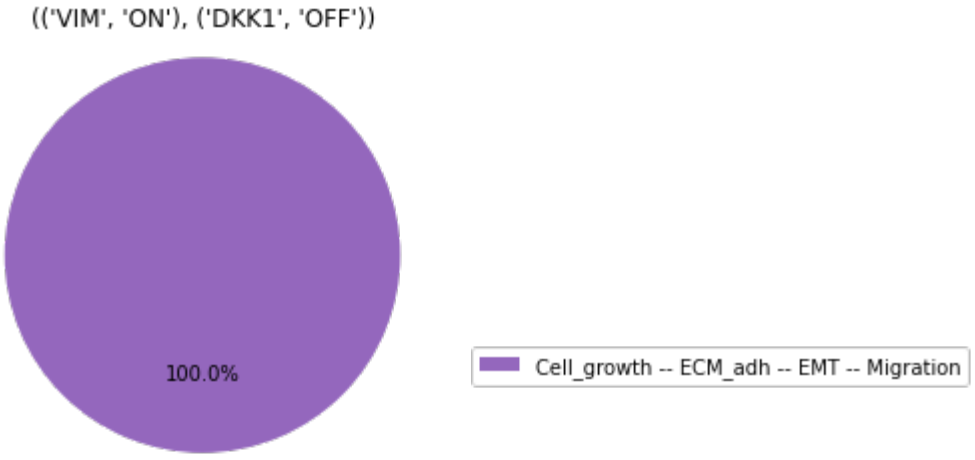
(('VIM', 'ON'), ('DKK1', 'OFF'))
100.0%
Cell_growth -- ECM_adh -- EMT -- Migration

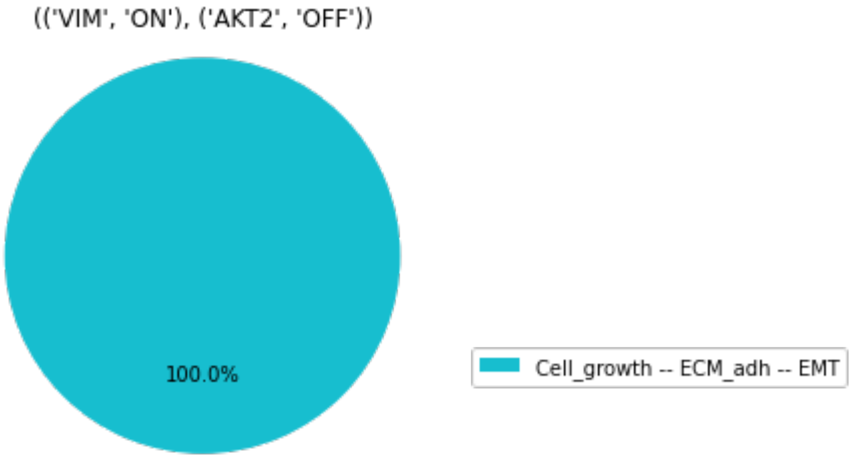
(('VIM', 'ON'), ('AKT2', 'OFF'))
100.0%
Cell_growth -- ECM_adh -- EMT

(('VIM', 'ON'), ('RAC1', 'OFF'))

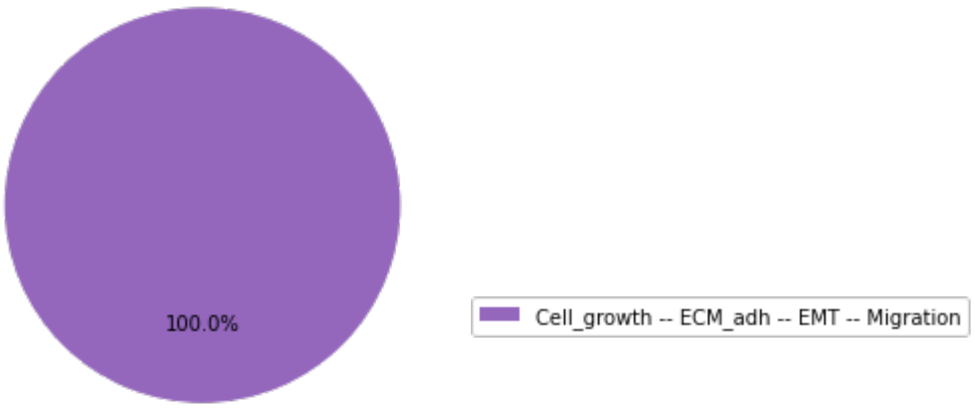

(('VIM', 'ON'), ('YAP1', 'OFF'))

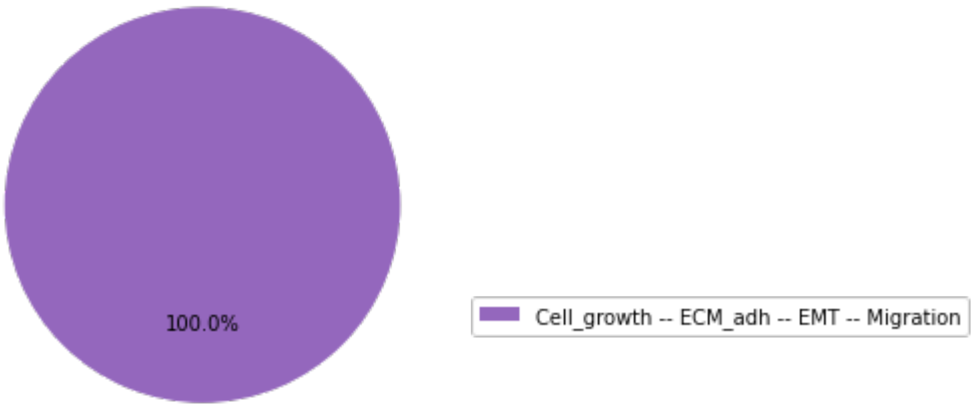

(('VIM', 'ON'), ('PIK3CA', 'OFF'))

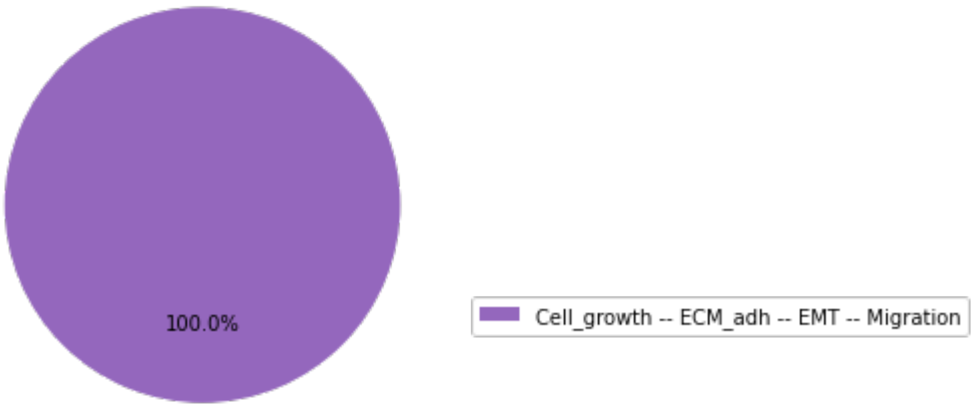

(('VIM', 'ON'), ('ERK', 'OFF'))

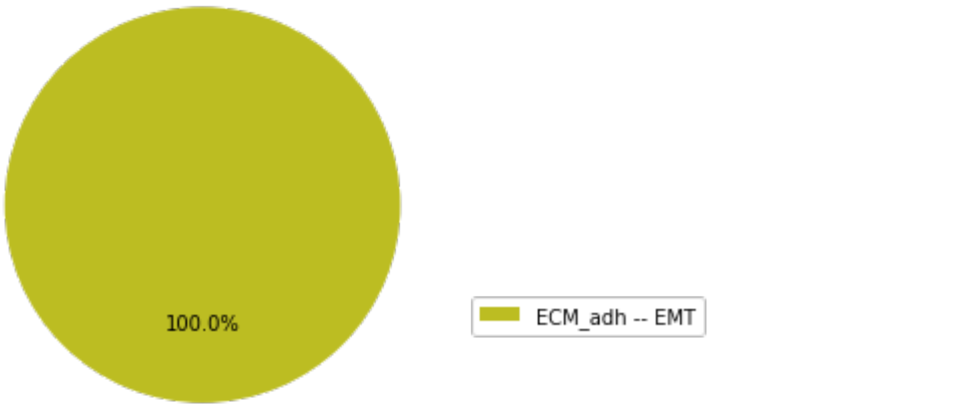

(('VIM', 'ON'), ('p21', 'OFF'))

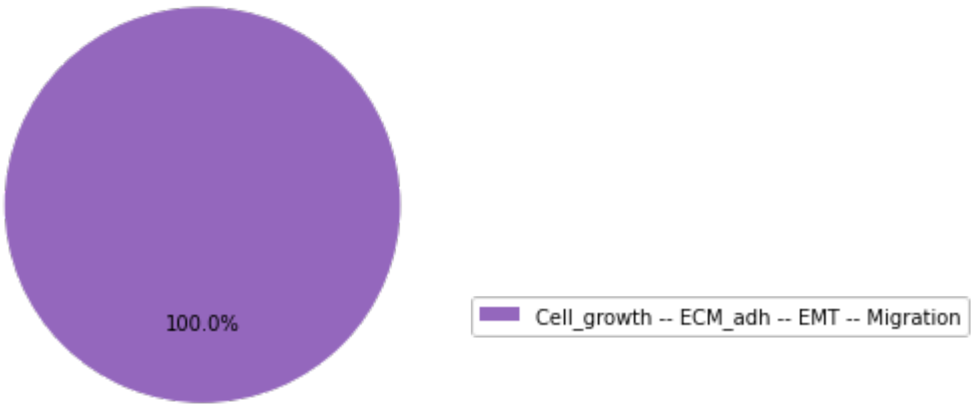

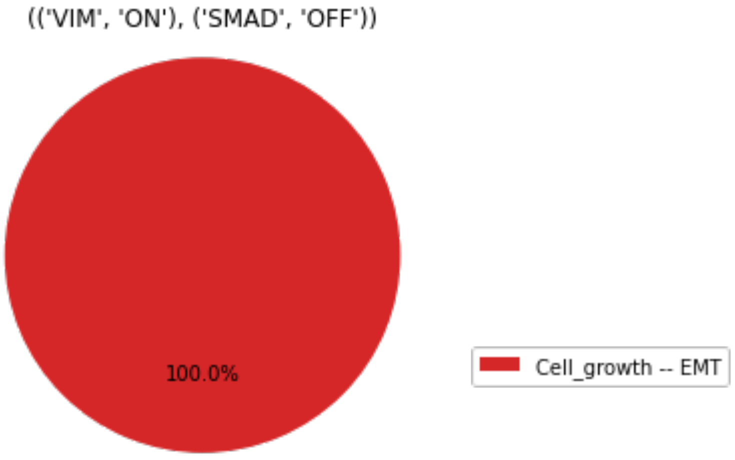
(('VIM', 'ON'), ('SMAD', 'OFF'))
100.0%
Cell_growth -- EMT

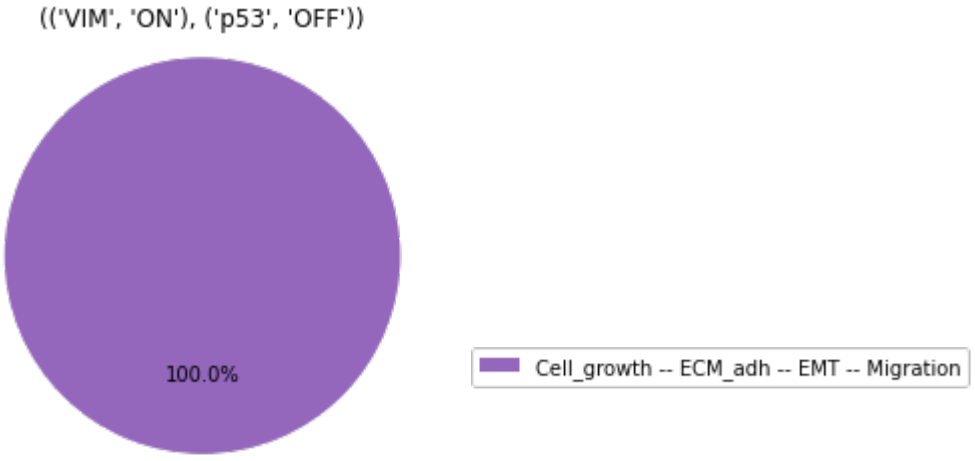
(('VIM', 'ON'), ('p53', 'OFF'))
100.0%
Cell_growth -- ECM_adh -- EMT -- Migration

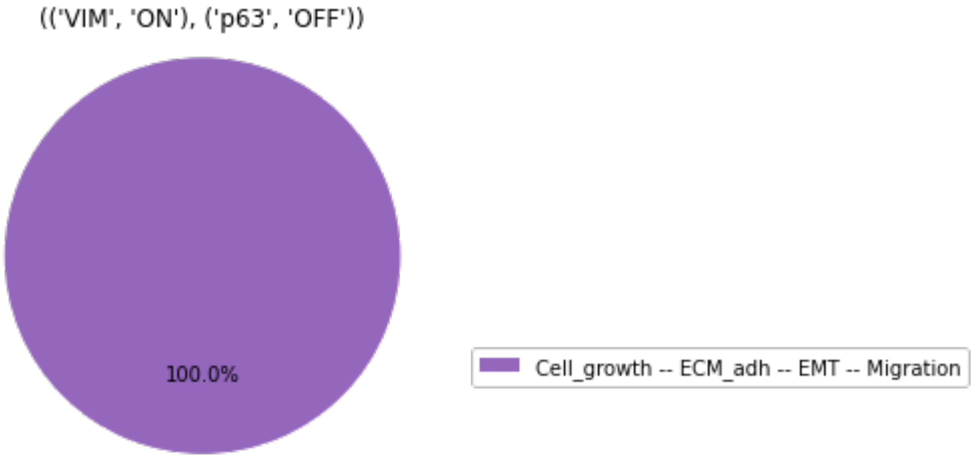
(('VIM', 'ON'), ('p63', 'OFF'))
100.0%
Cell_growth -- ECM_adh -- EMT -- Migration

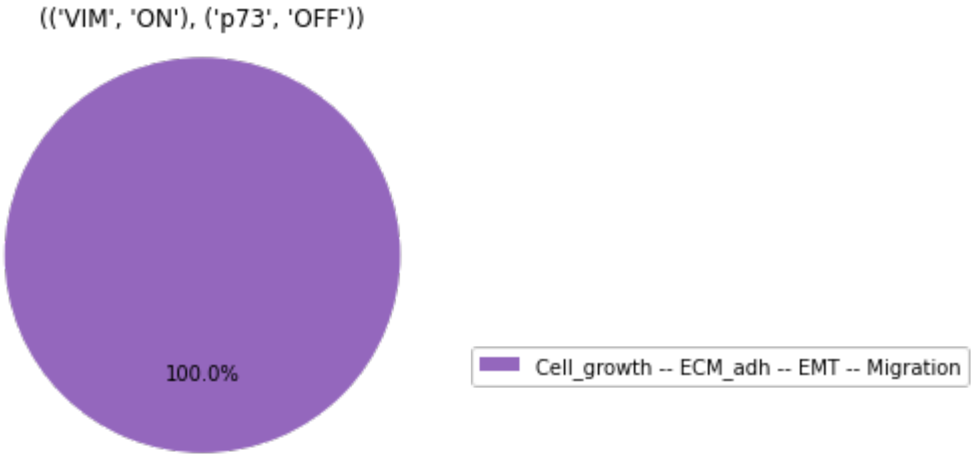
(('VIM', 'ON'), ('p73', 'OFF'))
100.0%
Cell_growth -- ECM_adh -- EMT -- Migration

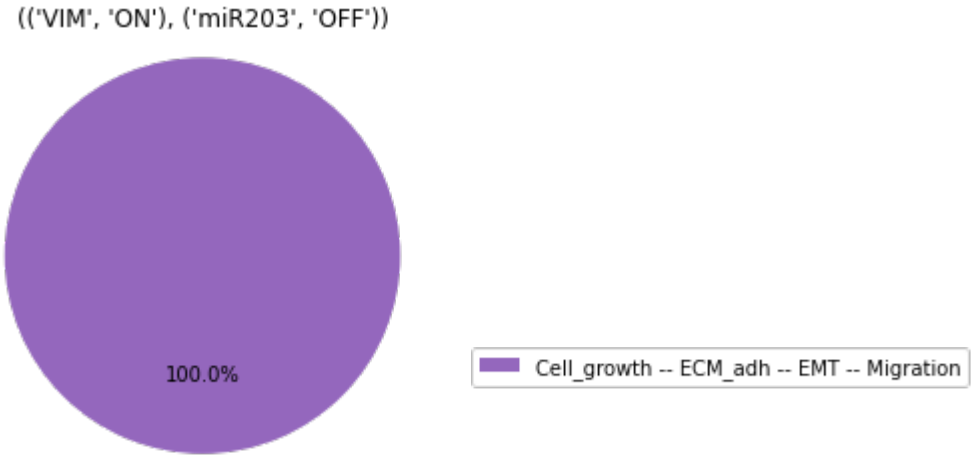
(('VIM', 'ON'), ('miR203', 'OFF'))
100.0%
Cell_growth -- ECM_adh -- EMT -- Migration

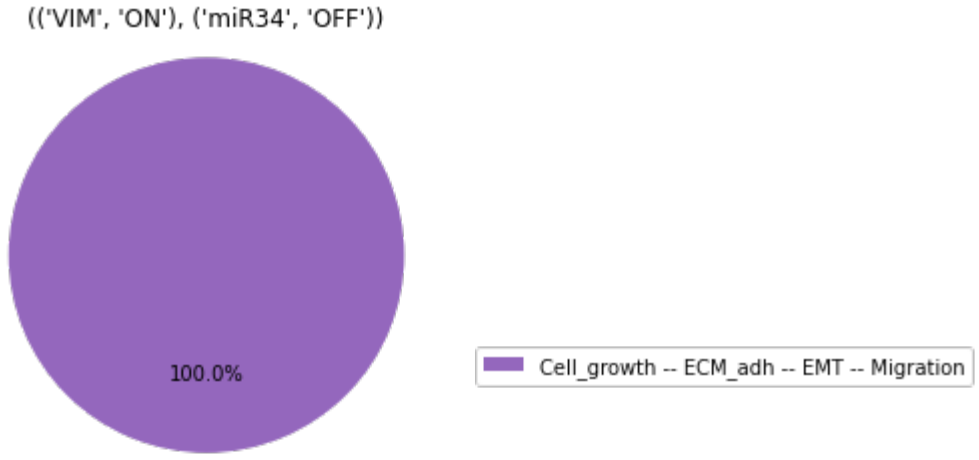
(('VIM', 'ON'), ('miR34', 'OFF'))
100.0%
Cell_growth -- ECM_adh -- EMT -- Migration

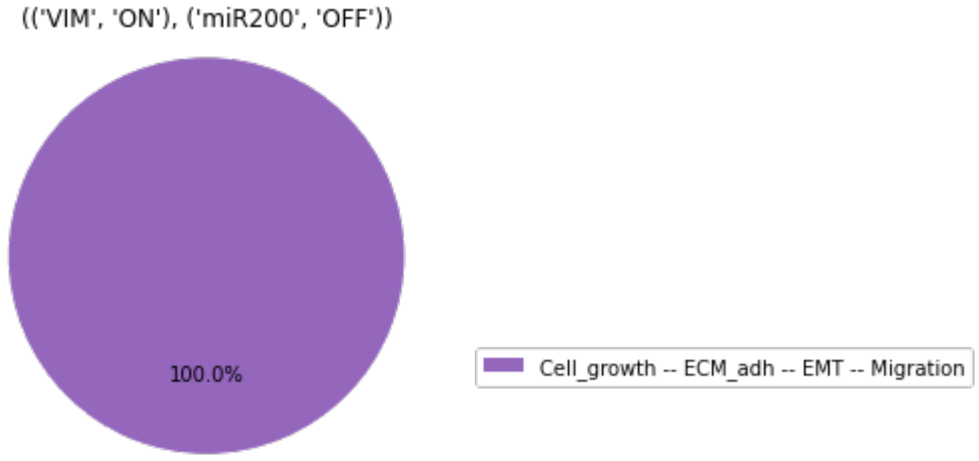
(('VIM', 'ON'), ('miR200', 'OFF'))
100.0%
Cell_growth -- ECM_adh -- EMT -- Migration

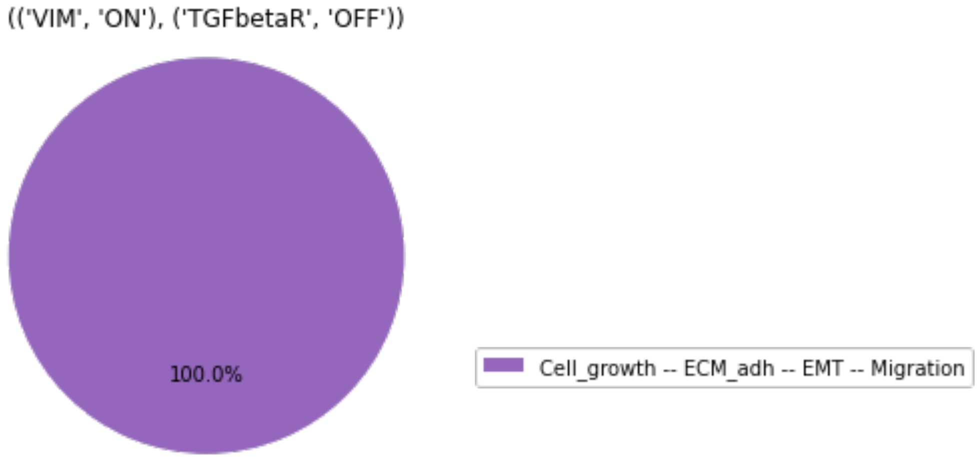
(('VIM', 'ON'), ('TGFbetaR', 'OFF'))
100.0%
Cell_growth -- ECM_adh -- EMT -- Migration

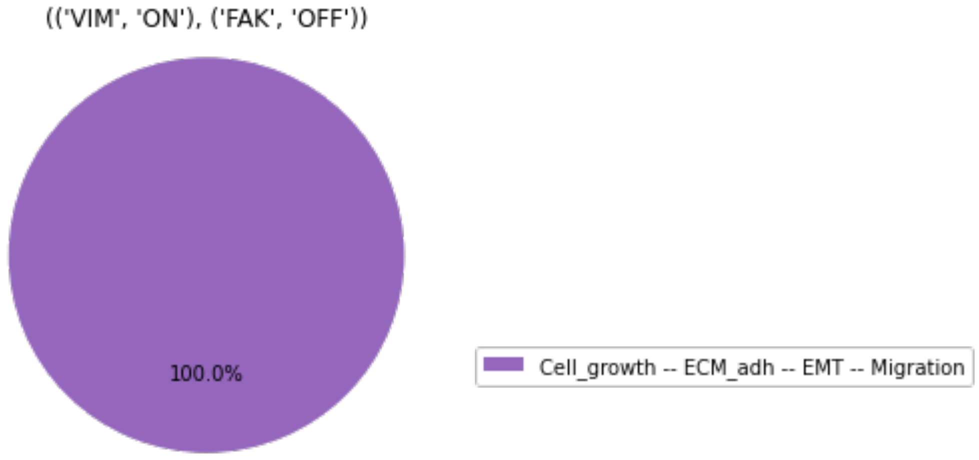
(('VIM', 'ON'), ('FAK', 'OFF'))
100.0%
Cell_growth -- ECM_adh -- EMT -- Migration

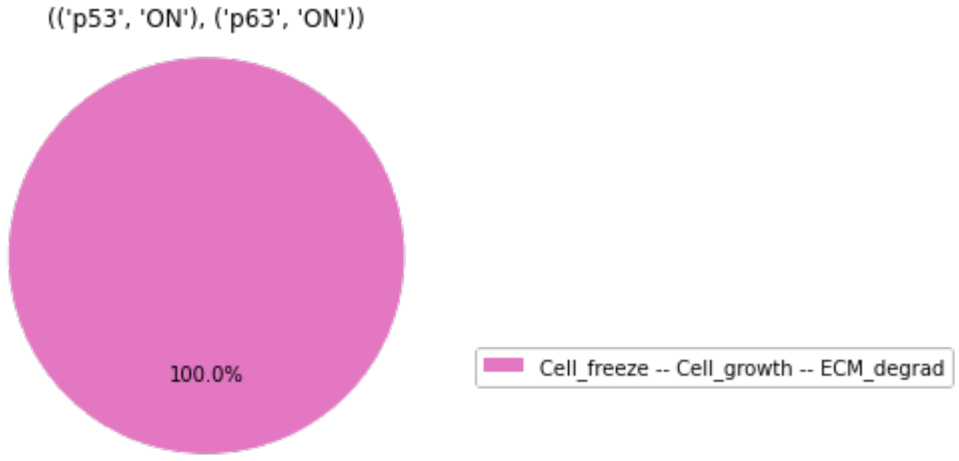
(('p53', 'ON'), ('p63', 'ON'))
100.0%
Cell_freeze -- Cell_growth -- ECM_degrad

(('p53', 'ON'), ('p73', 'ON'))

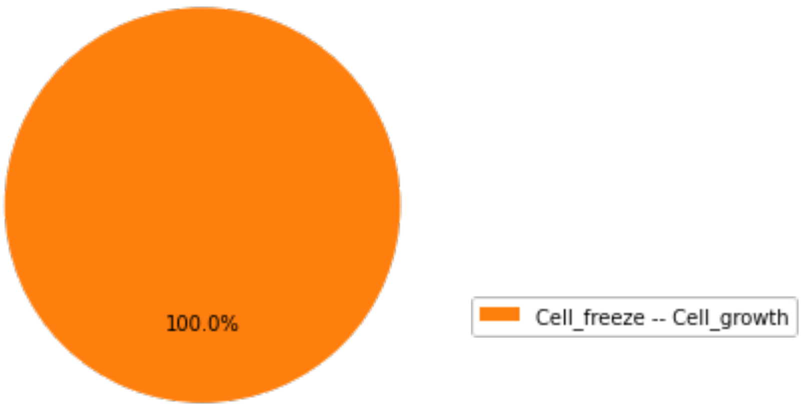

(('p53', 'ON'), ('miR203', 'ON'))

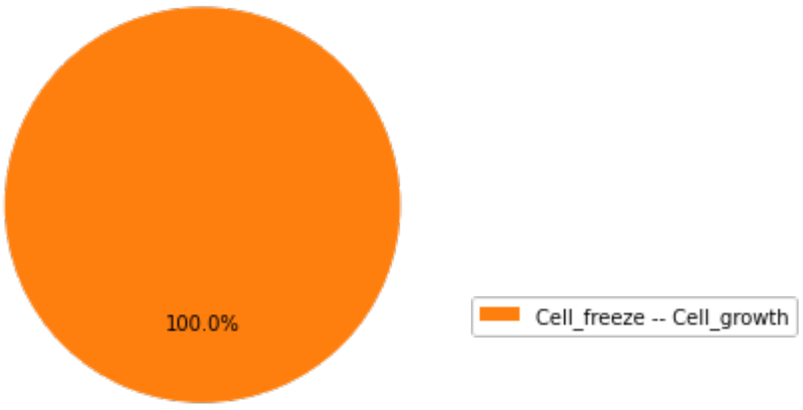

(('p53', 'ON'), ('miR34', 'ON'))

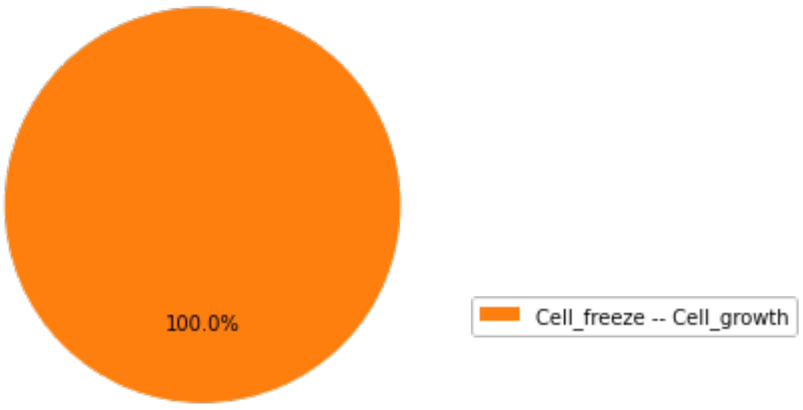

(('p53', 'ON'), ('miR200', 'ON'))

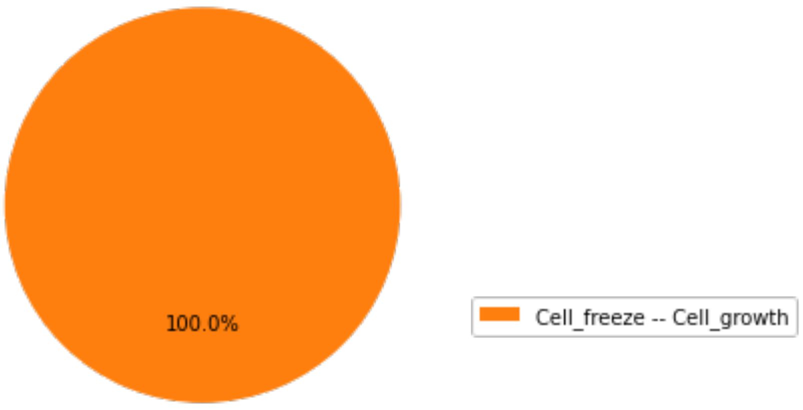

(('p53', 'ON'), ('TGFbetaR', 'ON'))

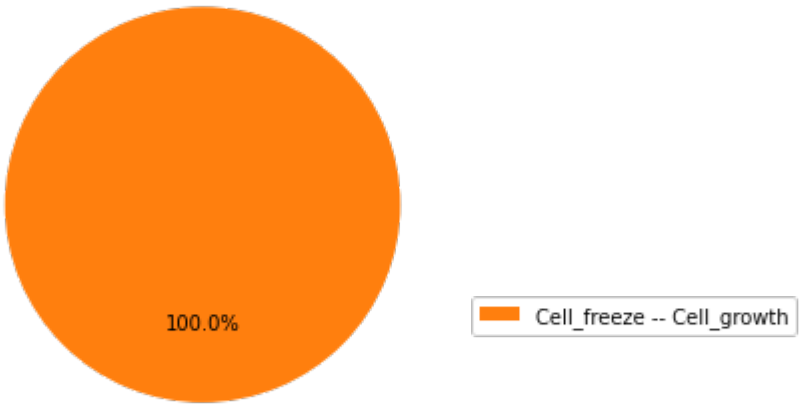

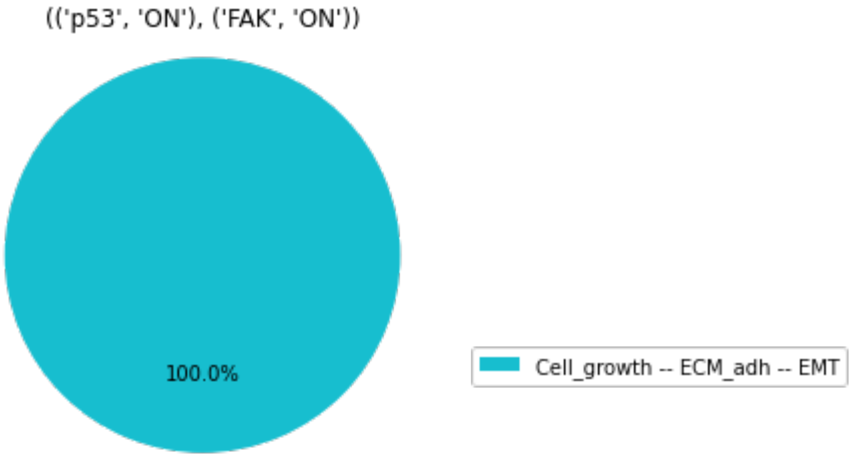
(('p53', 'ON'), ('FAK', 'ON'))
100.0%
Cell_growth -- ECM_adh -- EMT

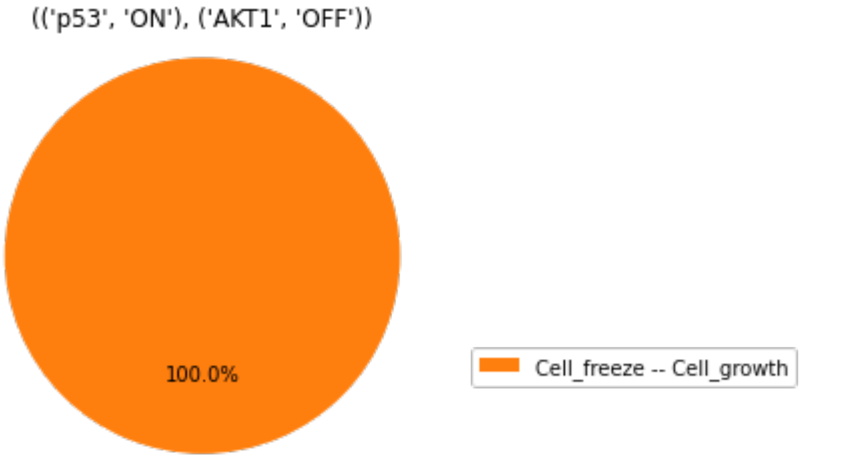
(('p53', 'ON'), ('AKT1', 'OFF'))
100.0%
Cell_freeze -- Cell_growth

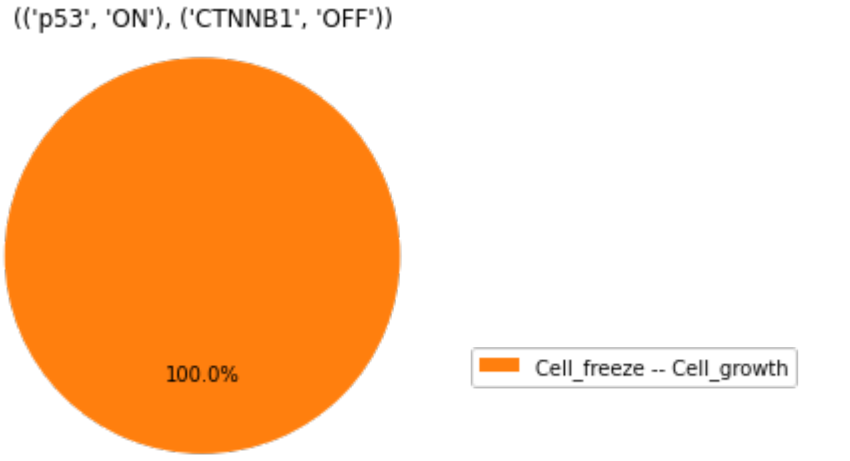
(('p53', 'ON'), ('CTNNB1', 'OFF'))
100.0%
Cell_freeze -- Cell_growth

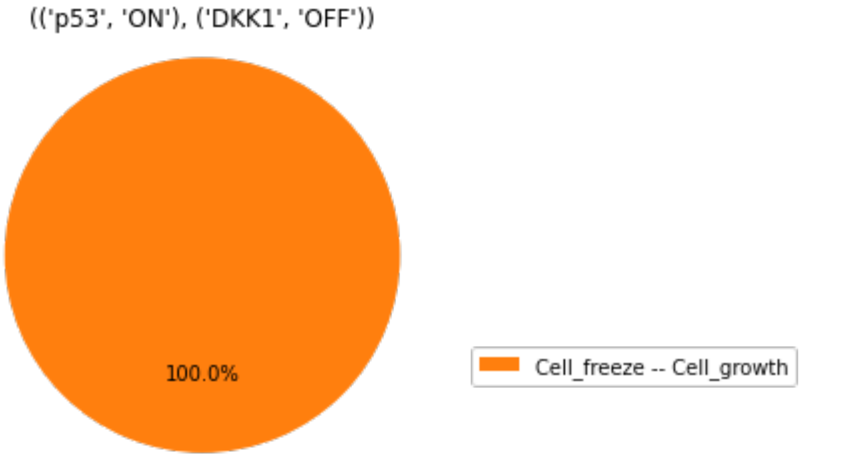
(('p53', 'ON'), ('DKK1', 'OFF'))
100.0%
Cell_freeze -- Cell_growth

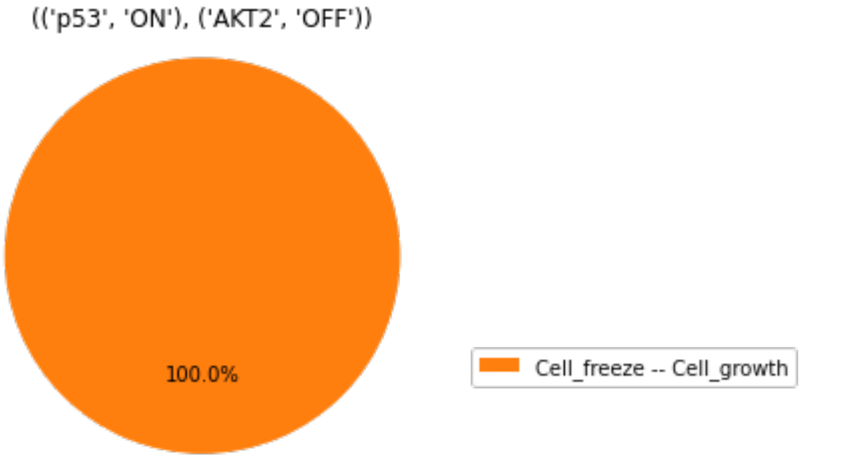
(('p53', 'ON'), ('AKT2', 'OFF'))
100.0%
Cell_freeze -- Cell_growth

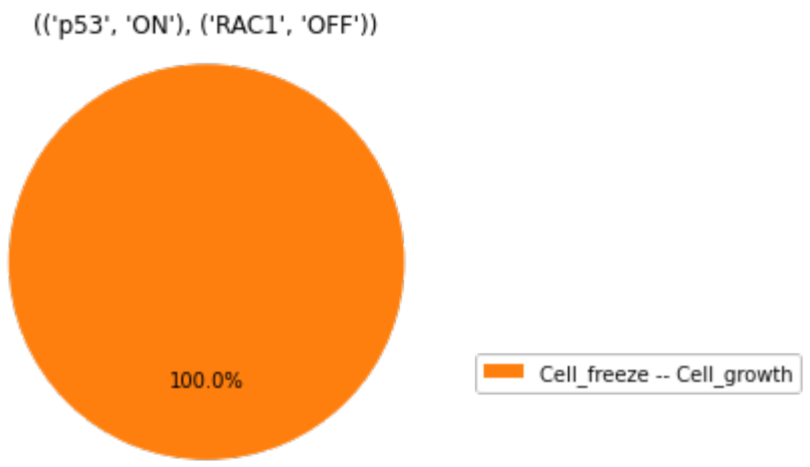
(('p53', 'ON'), ('RAC1', 'OFF'))
100.0%
Cell_freeze -- Cell_growth

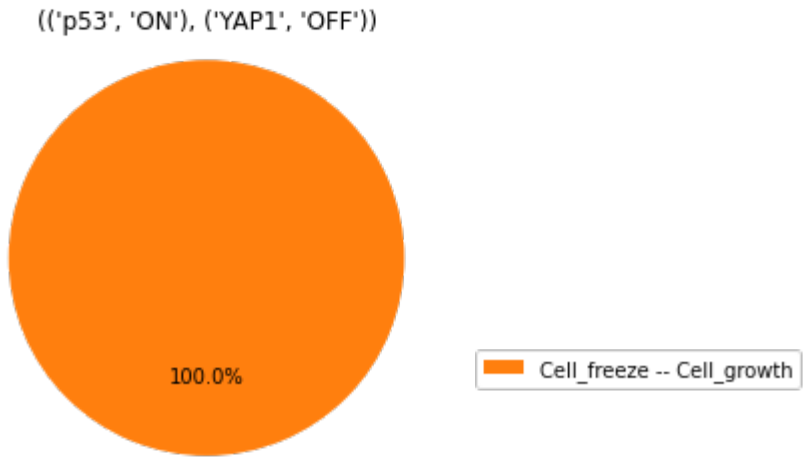
(('p53', 'ON'), ('YAP1', 'OFF'))
100.0%
Cell_freeze -- Cell_growth

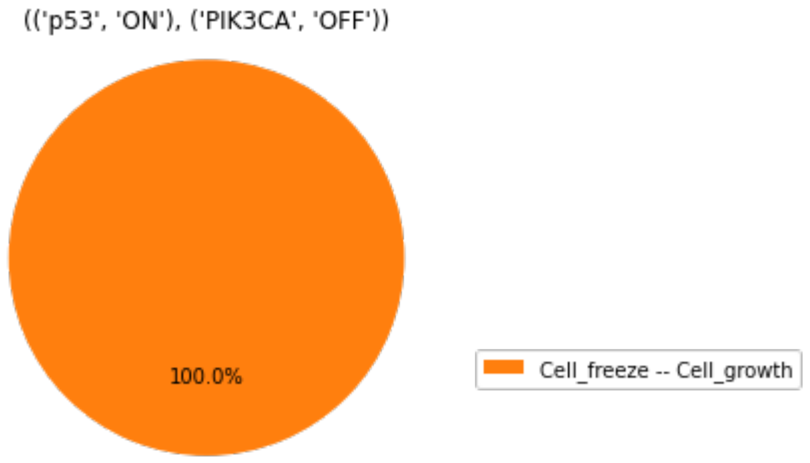
(('p53', 'ON'), ('PIK3CA', 'OFF'))
100.0%
Cell_freeze -- Cell_growth

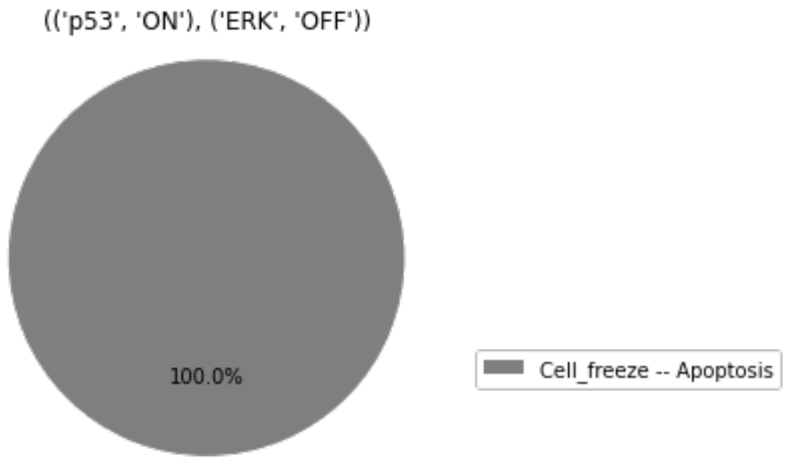
(('p53', 'ON'), ('ERK', 'OFF'))
100.0%
Cell_freeze -- Apoptosis

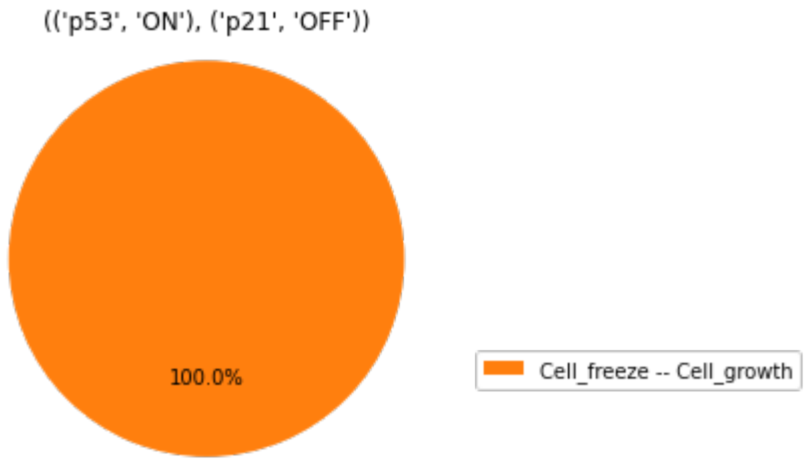
(('p53', 'ON'), ('p21', 'OFF'))
100.0%
Cell_freeze -- Cell_growth

(('p53', 'ON'), ('SMAD', 'OFF'))

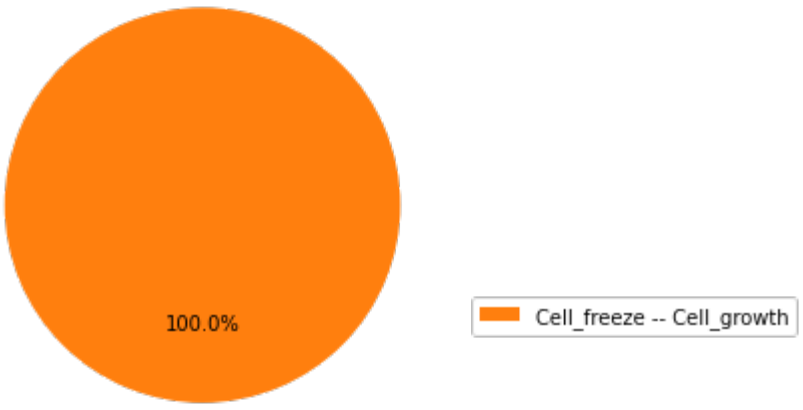

(('p53', 'ON'), ('VIM', 'OFF'))

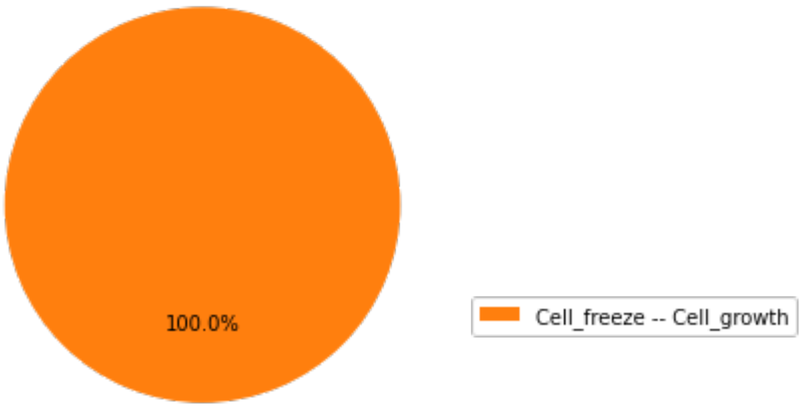

(('p53', 'ON'), ('p63', 'OFF'))

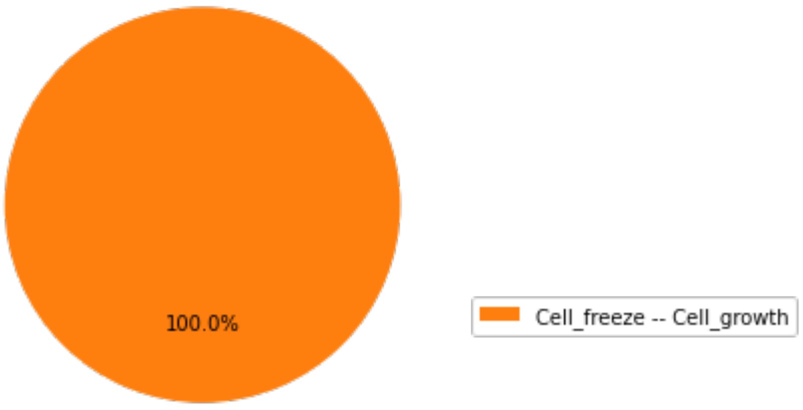

(('p53', 'ON'), ('p73', 'OFF'))

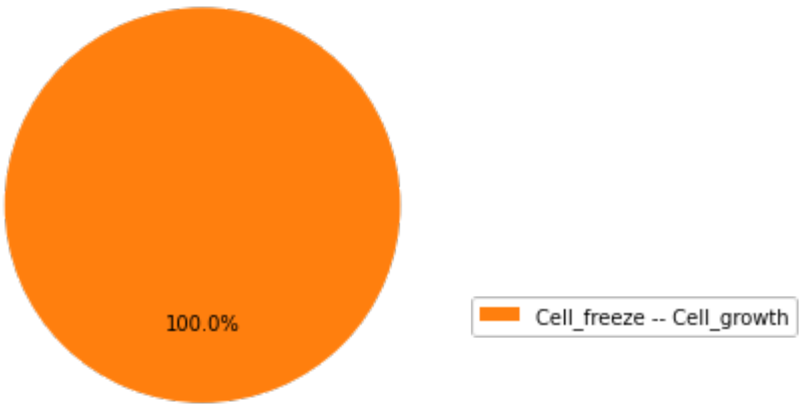

(('p53', 'ON'), ('miR203', 'OFF'))

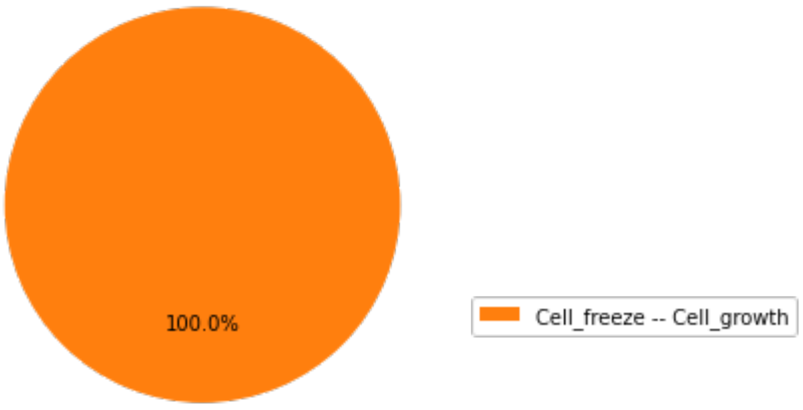

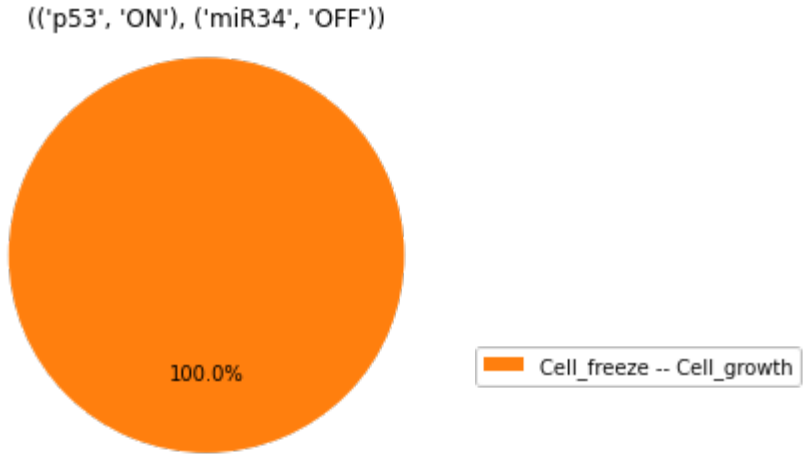
(('p53', 'ON'), ('miR34', 'OFF'))
100.0%
Cell_freeze -- Cell_growth

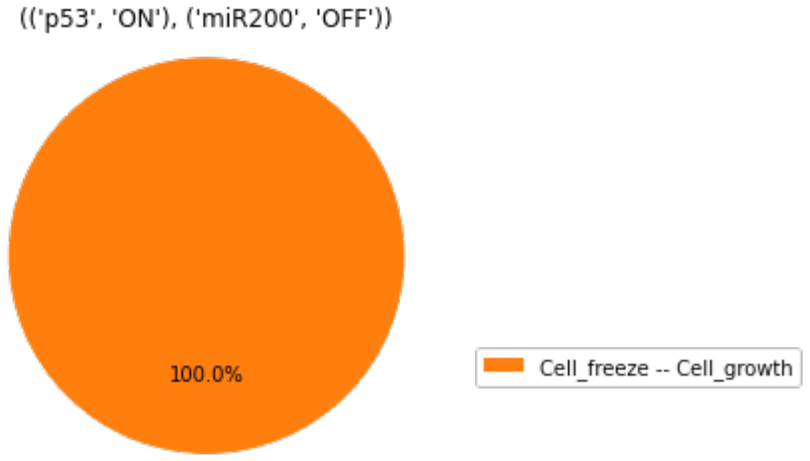
(('p53', 'ON'), ('miR200', 'OFF'))
100.0%
Cell_freeze -- Cell_growth

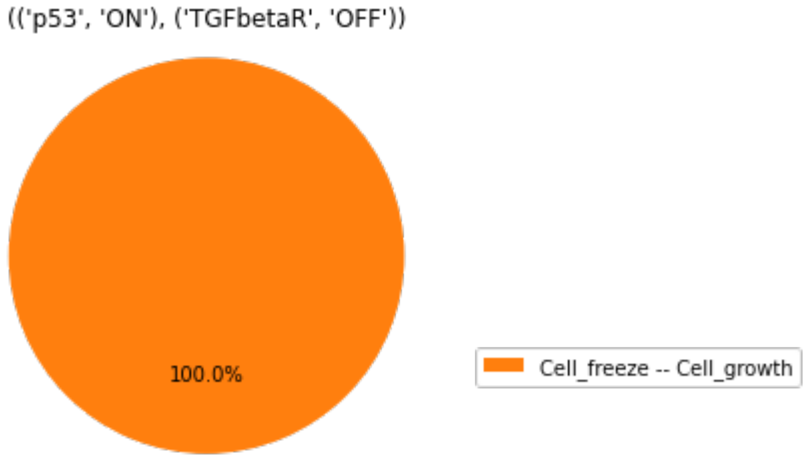
(('p53', 'ON'), ('TGFbetaR', 'OFF'))
100.0%
Cell_freeze -- Cell_growth

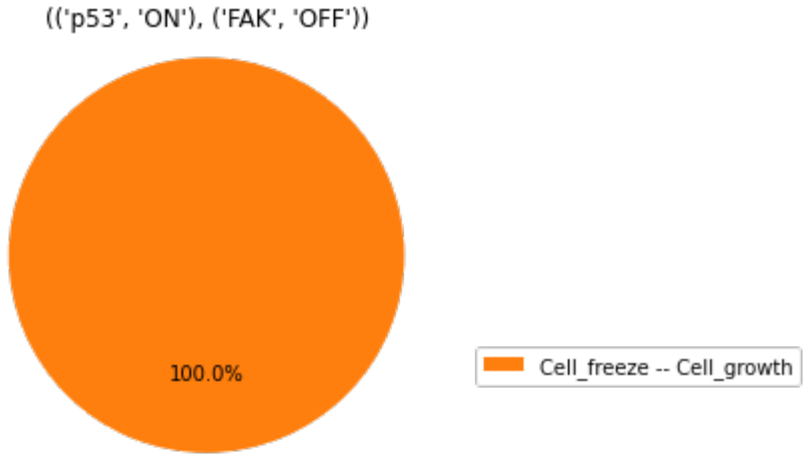
(('p53', 'ON'), ('FAK', 'OFF'))
100.0%
Cell_freeze -- Cell_growth

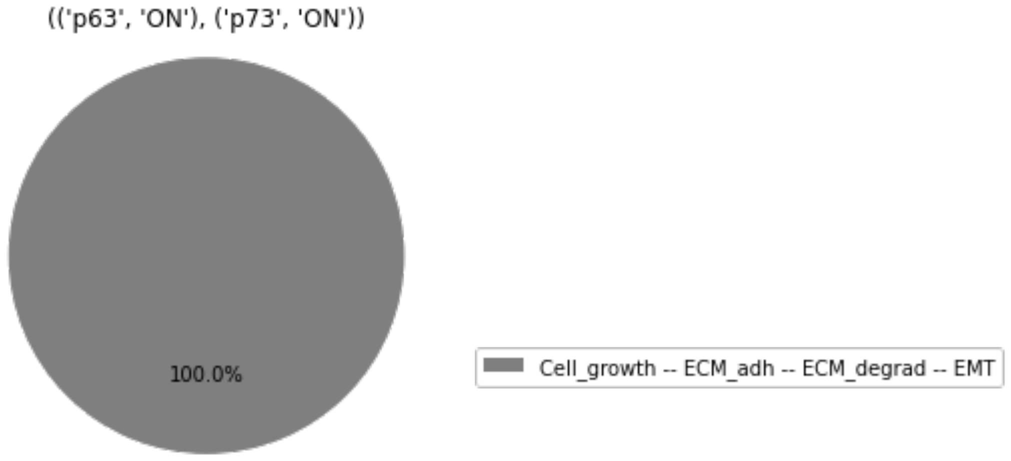
(('p63', 'ON'), ('p73', 'ON'))
100.0%
Cell_growth -- ECM_adh -- ECM_degrad -- EMT

(('p63', 'ON'), ('miR203', 'ON'))

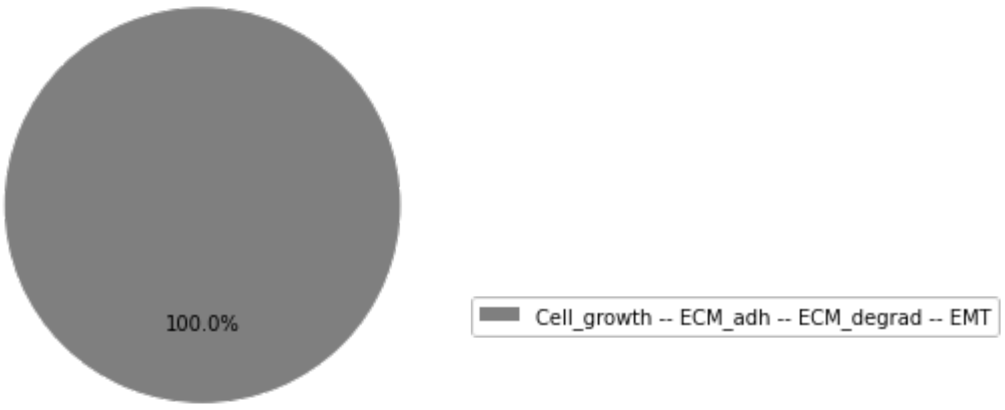

(('p63', 'ON'), ('miR34', 'ON'))

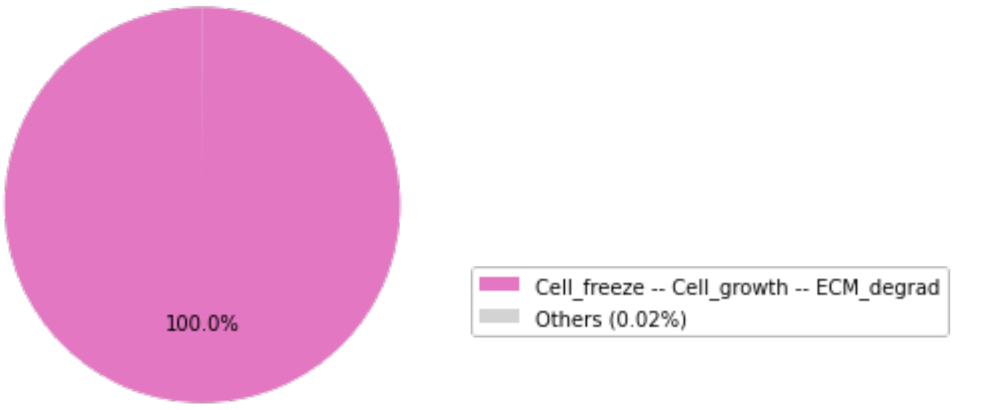

(('p63', 'ON'), ('miR200', 'ON'))

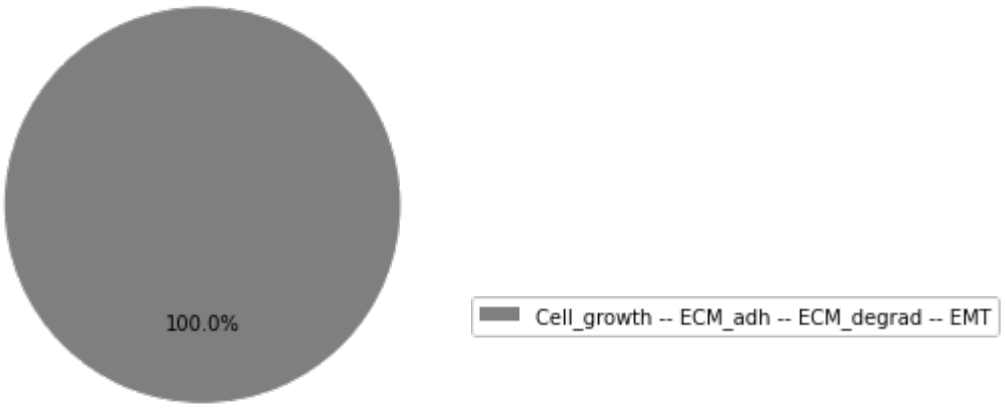

(('p63', 'ON'), ('TGFbetaR', 'ON'))

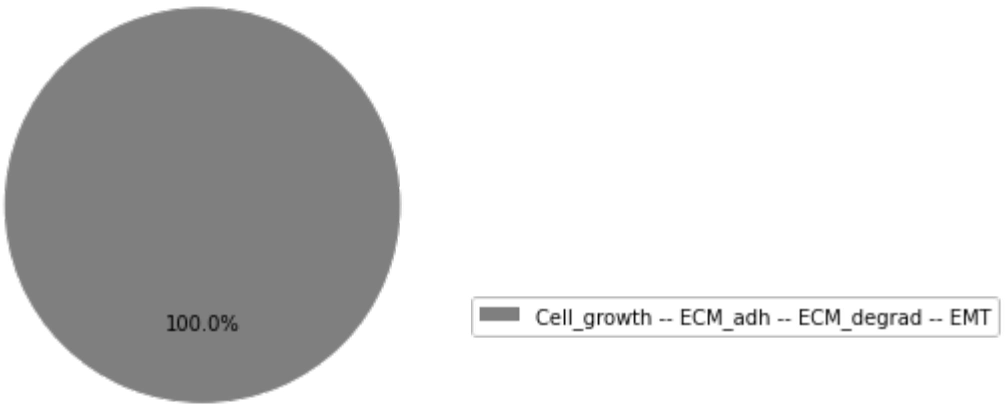

(('p63', 'ON'), ('FAK', 'ON'))

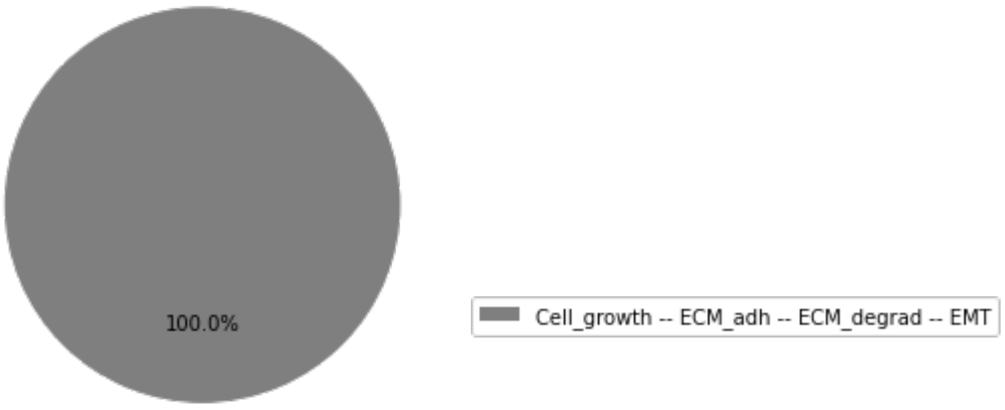

(('p63', 'ON'), ('AKT1', 'OFF'))

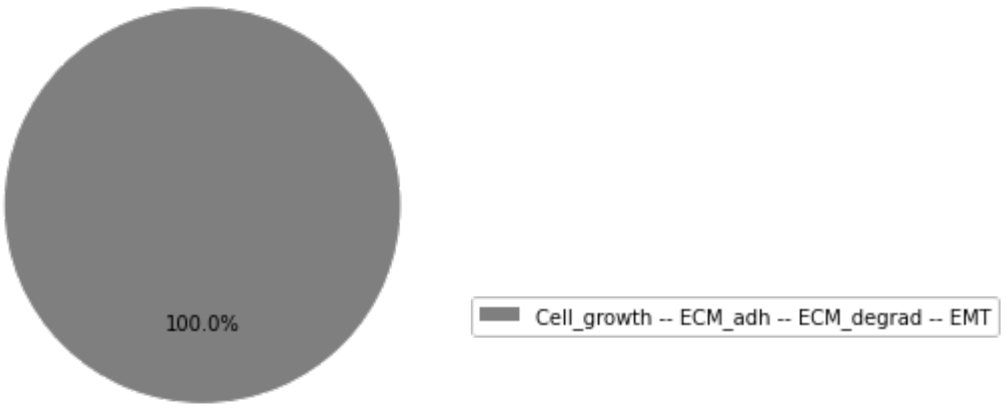

(('p63', 'ON'), ('CTNNB1', 'OFF'))

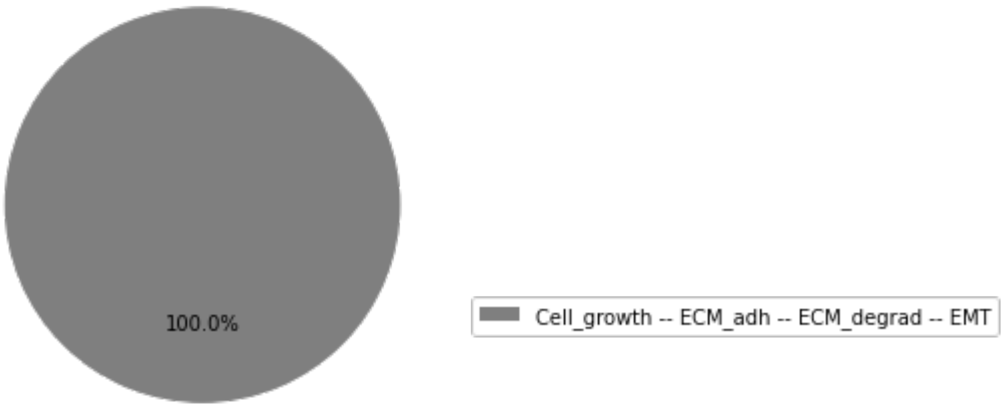

(('p63', 'ON'), ('DKK1', 'OFF'))

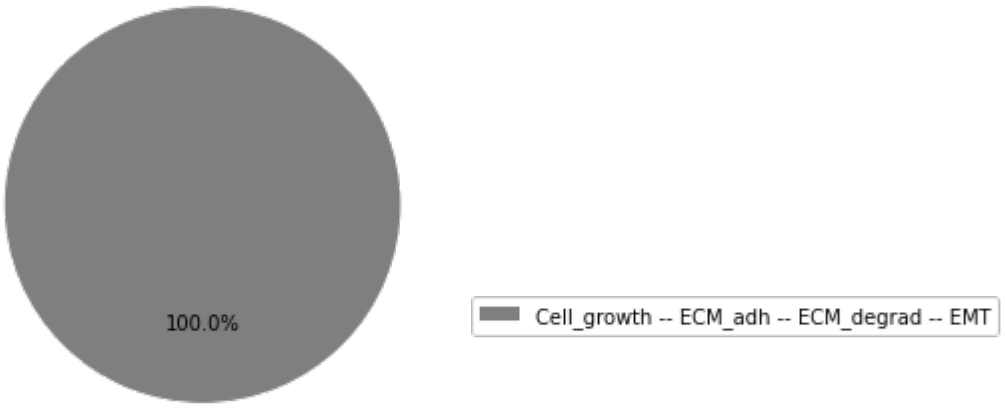

(('p63', 'ON'), ('AKT2', 'OFF'))

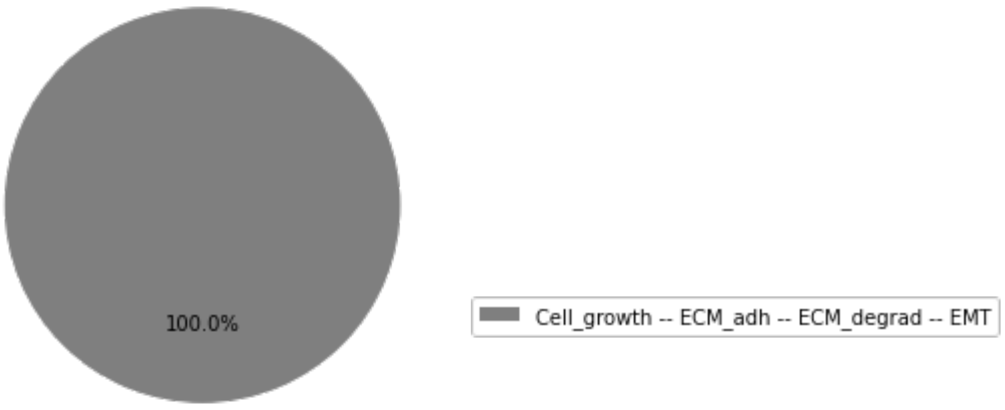

(('p63', 'ON'), ('RAC1', 'OFF'))

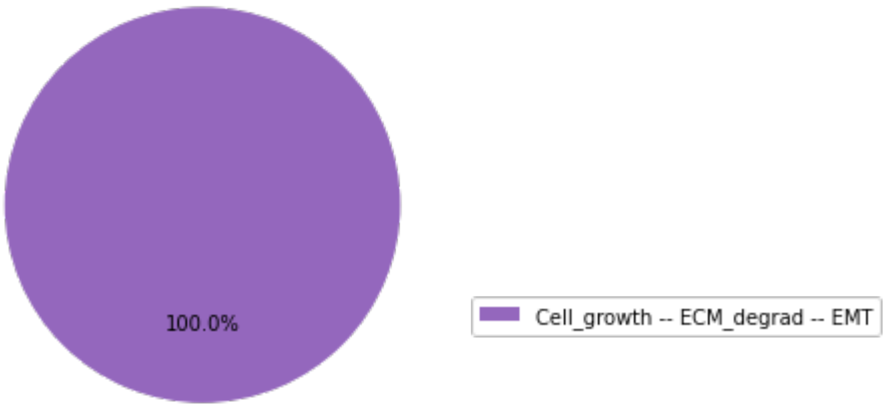

(('p63', 'ON'), ('YAP1', 'OFF'))

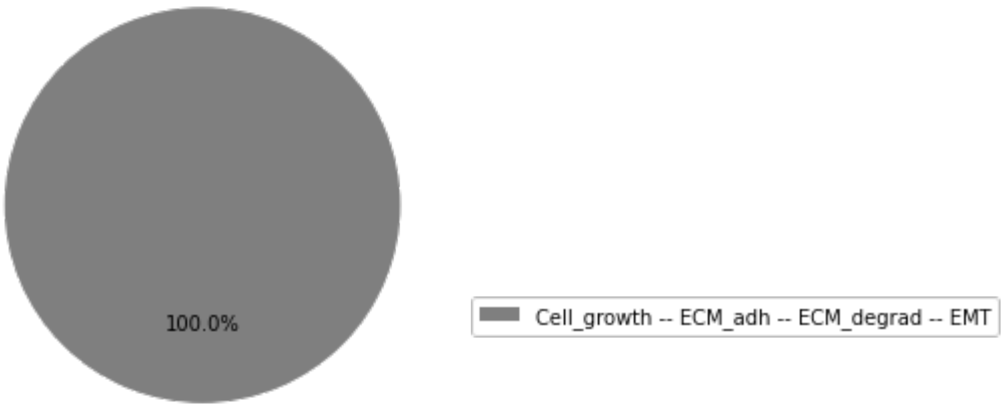

(('p63', 'ON'), ('PIK3CA', 'OFF'))

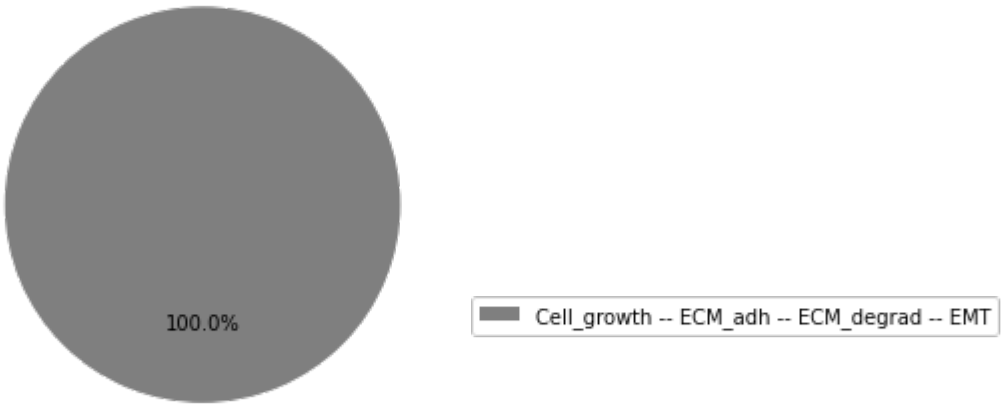

(('p63', 'ON'), ('ERK', 'OFF'))

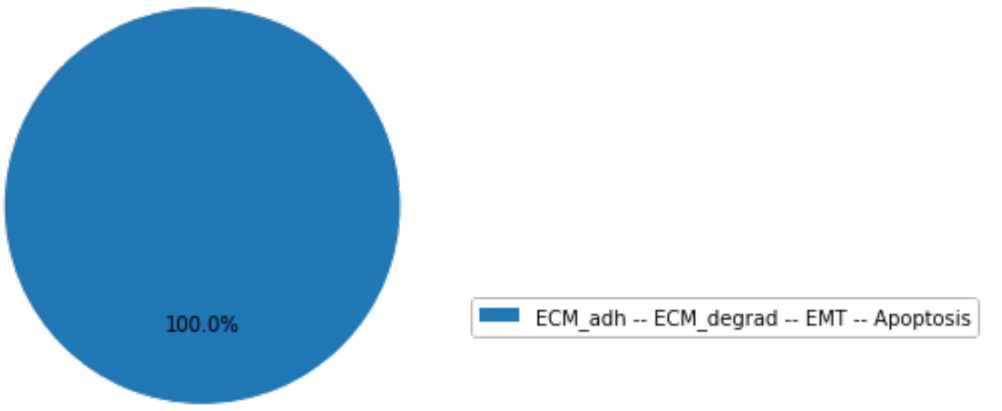

(('p63', 'ON'), ('p21', 'OFF'))

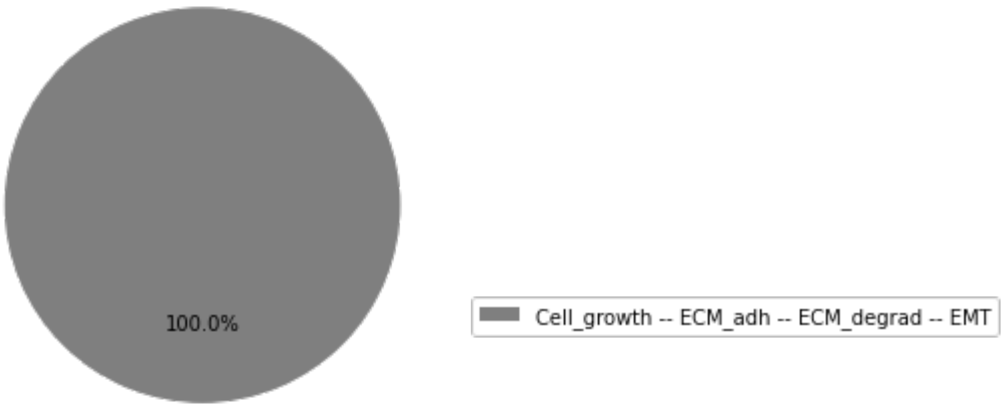

(('p63', 'ON'), ('SMAD', 'OFF'))

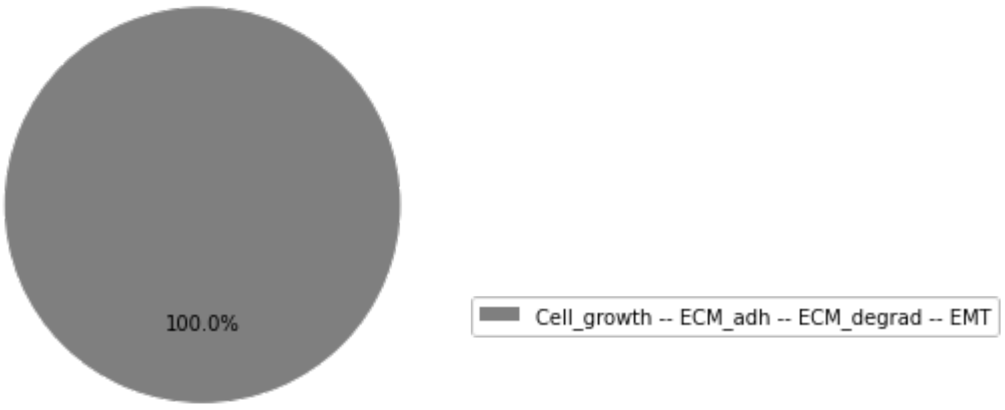

(('p63', 'ON'), ('VIM', 'OFF'))

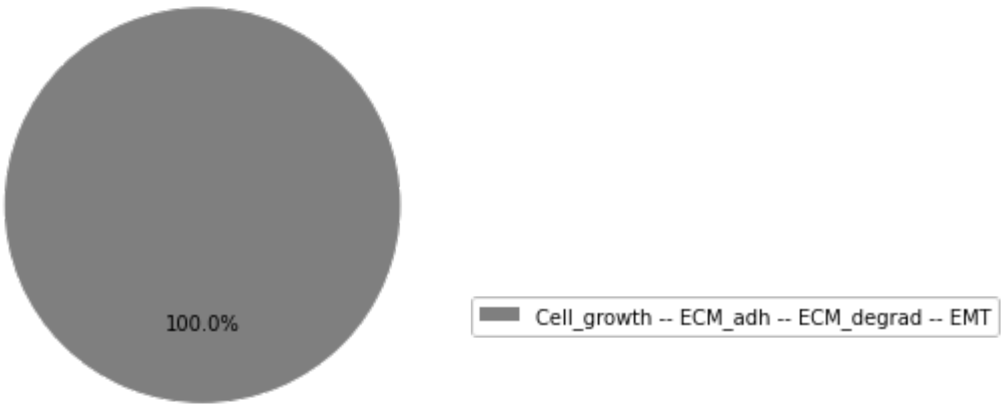

(('p63', 'ON'), ('p53', 'OFF'))

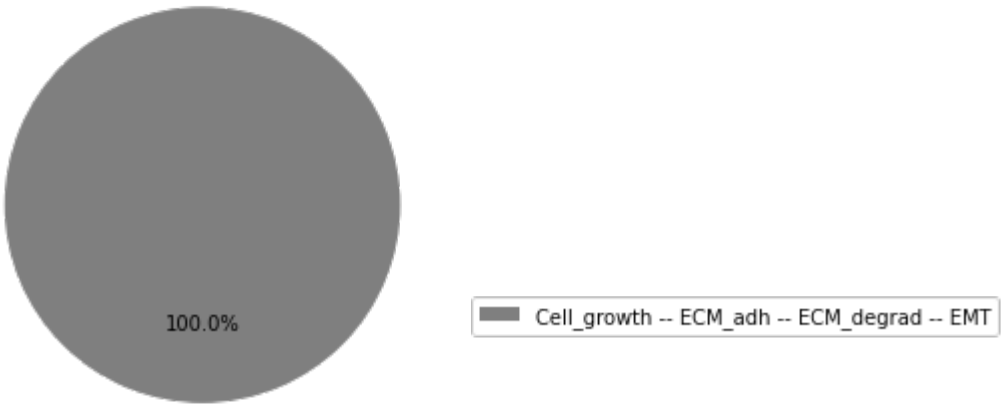

(('p63', 'ON'), ('p73', 'OFF'))

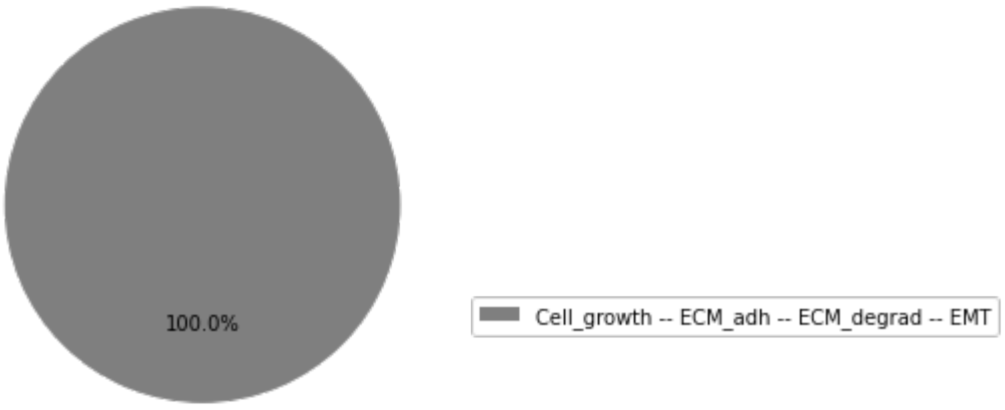

(('p63', 'ON'), ('miR203', 'OFF'))

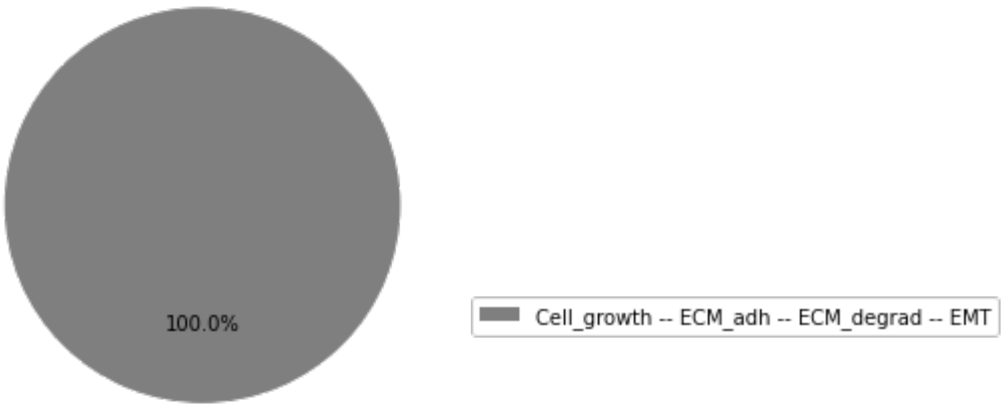

(('p63', 'ON'), ('miR34', 'OFF'))

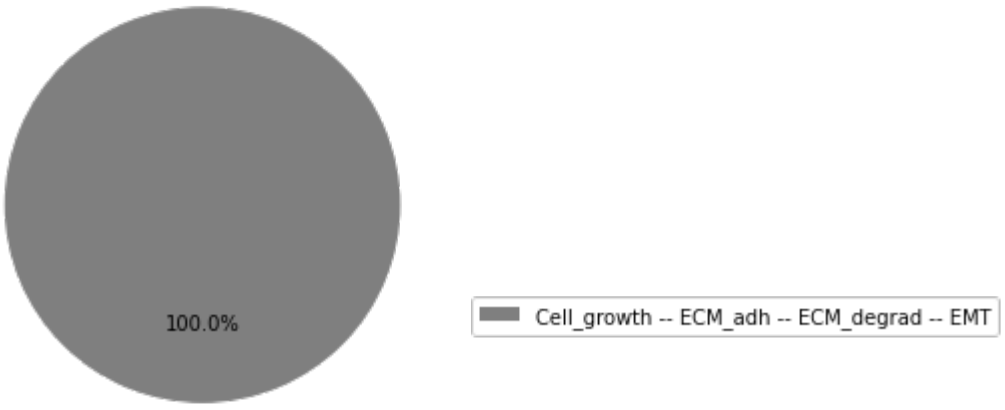

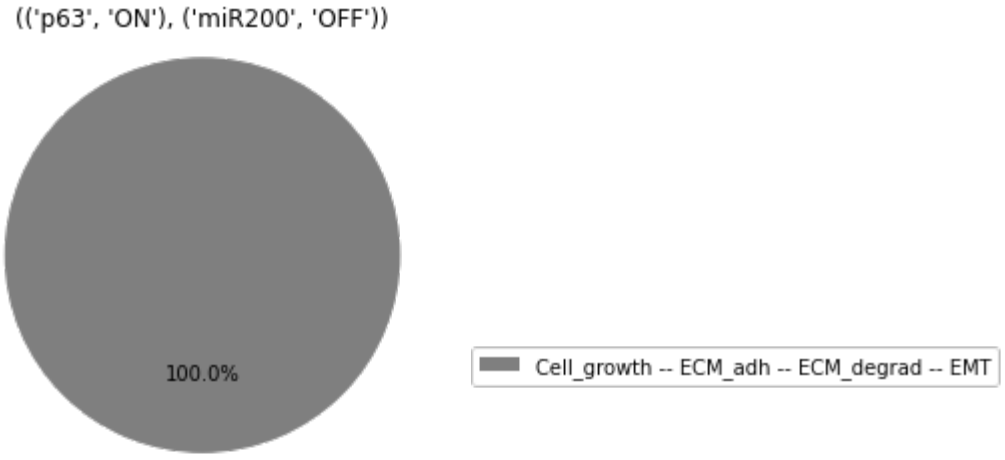
(('p63', 'ON'), ('miR200', 'OFF'))
100.0%
Cell_growth -- ECM_adh -- ECM_degrad -- EMT

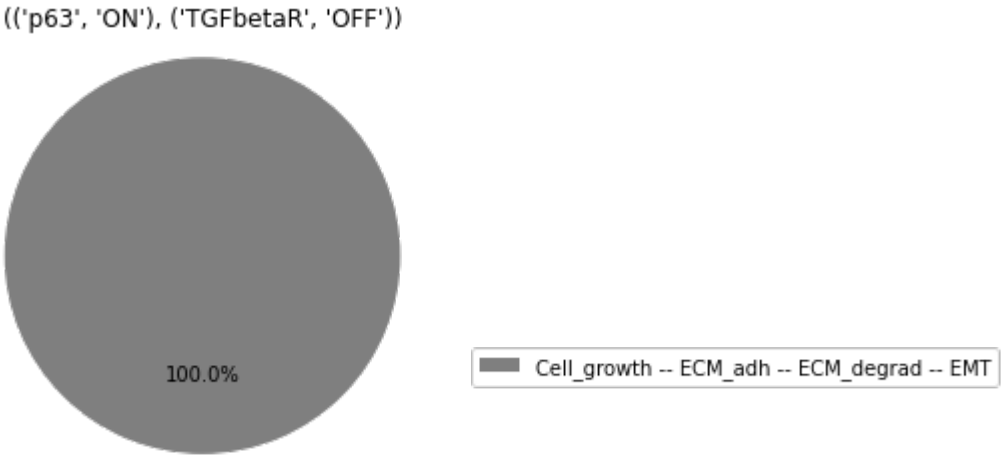
(('p63', 'ON'), ('TGFbetaR', 'OFF'))
100.0%
Cell_growth -- ECM_adh -- ECM_degrad -- EMT

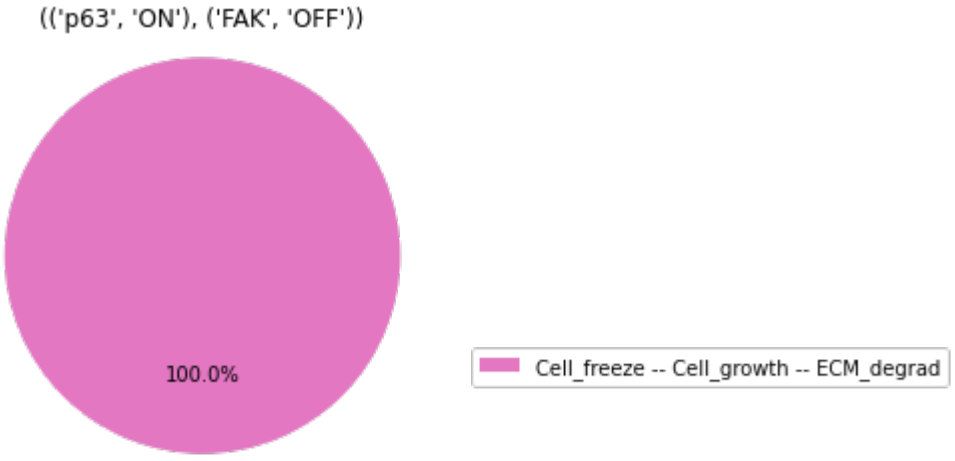
(('p63', 'ON'), ('FAK', 'OFF'))
100.0%
Cell_freeze -- Cell_growth -- ECM_degrad

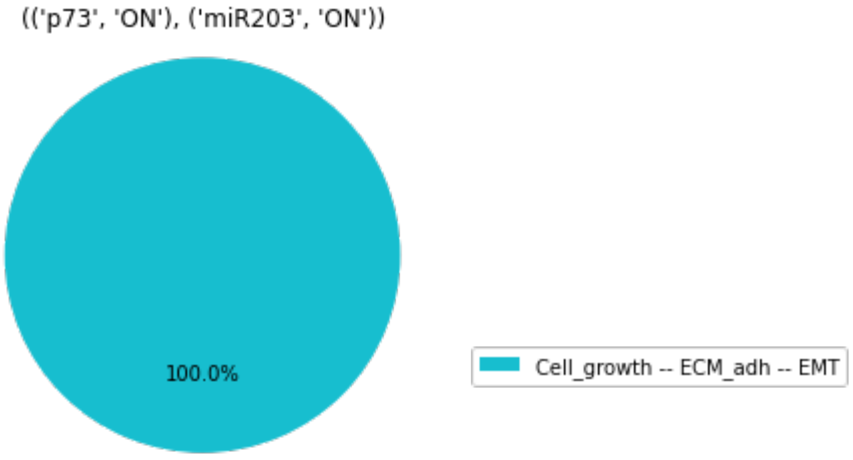
(('p73', 'ON'), ('miR203', 'ON'))
100.0%
Cell_growth -- ECM_adh -- EMT

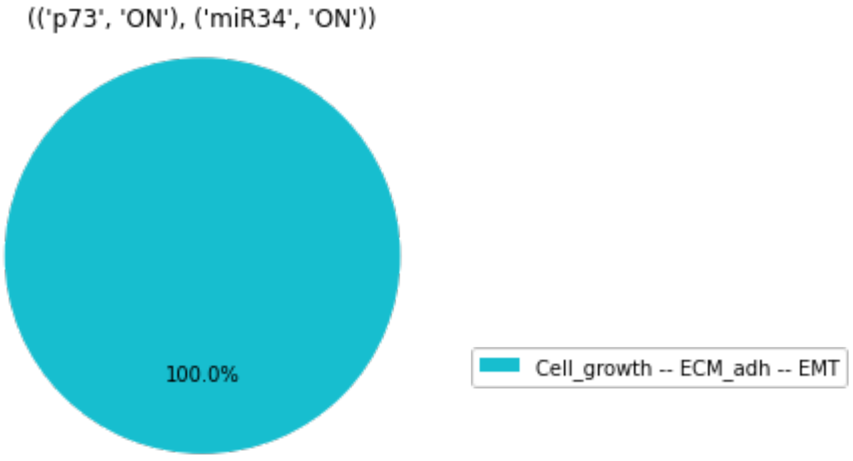
(('p73', 'ON'), ('miR34', 'ON'))
100.0%
Cell_growth -- ECM_adh -- EMT

(('p73', 'ON'), ('miR200', 'ON'))

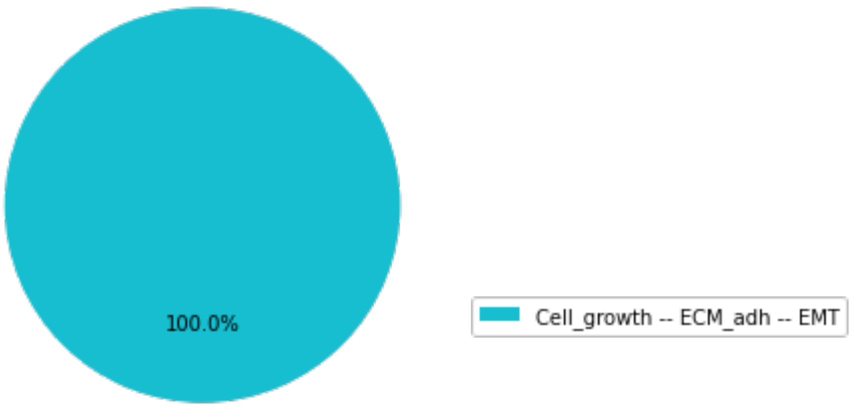

(('p73', 'ON'), ('TGFbetaR', 'ON'))

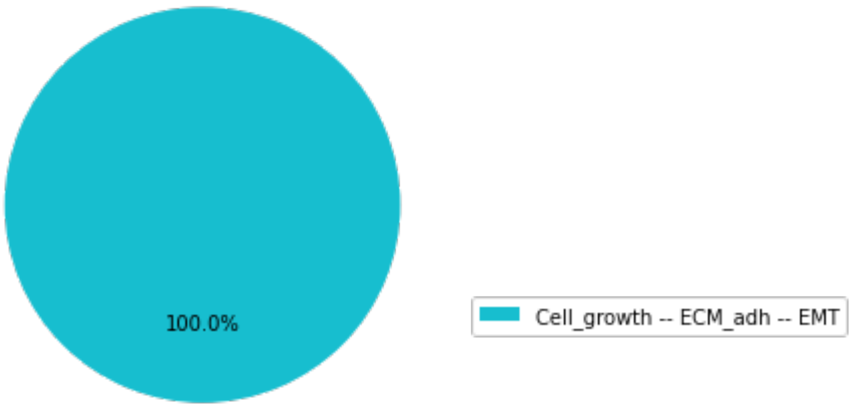

(('p73', 'ON'), ('FAK', 'ON'))

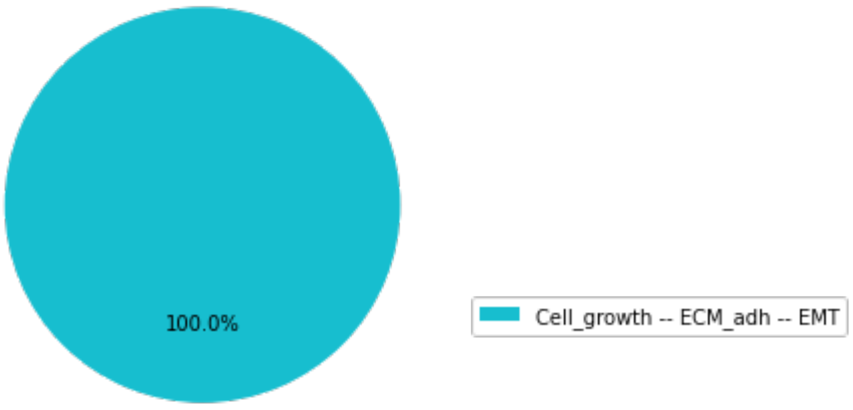

(('p73', 'ON'), ('AKT1', 'OFF'))

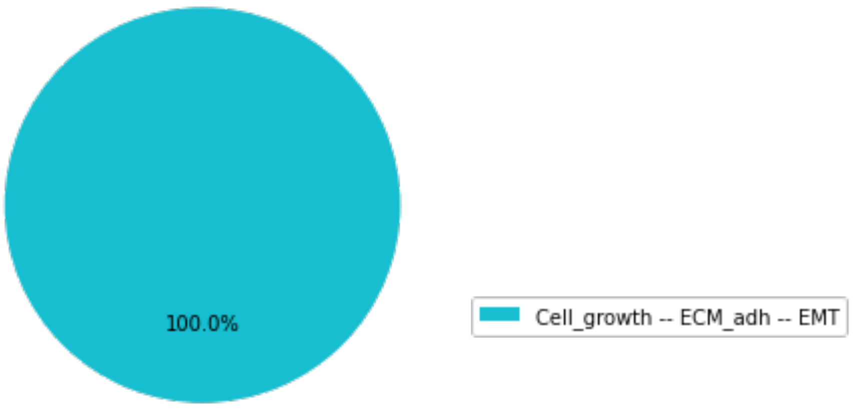

(('p73', 'ON'), ('CTNNB1', 'OFF'))

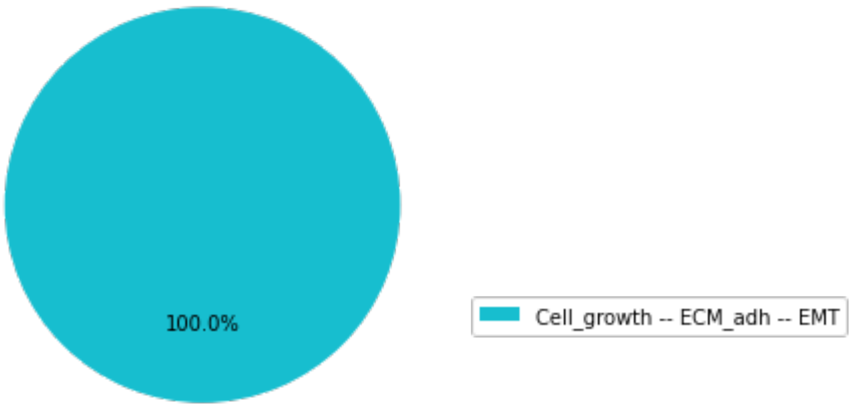

(('p73', 'ON'), ('DKK1', 'OFF'))

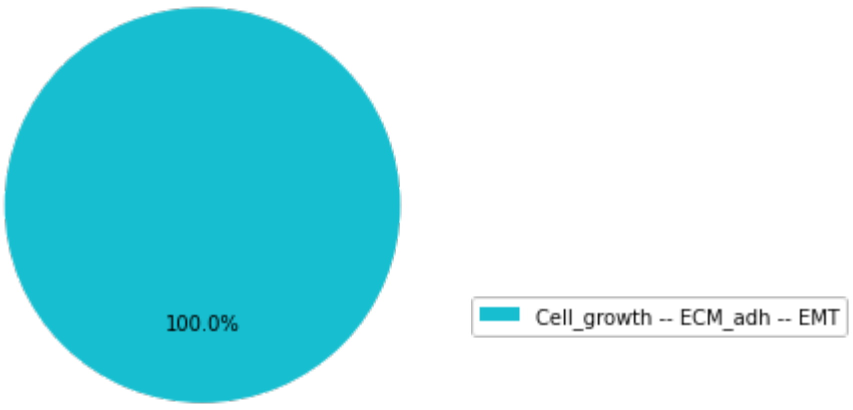

(('p73', 'ON'), ('AKT2', 'OFF'))

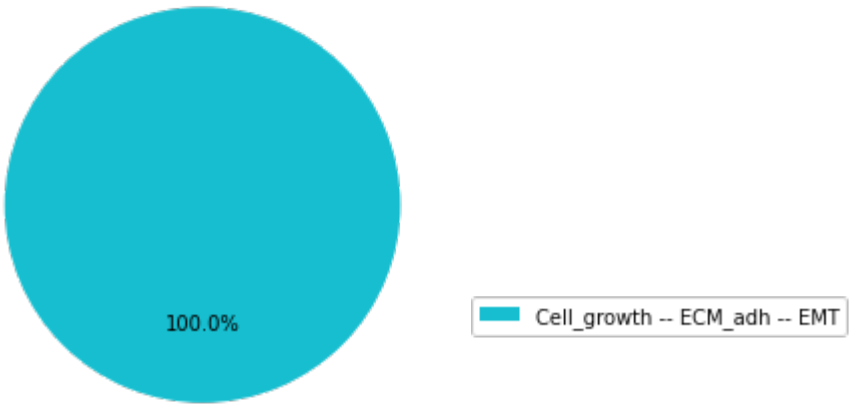

(('p73', 'ON'), ('RAC1', 'OFF'))

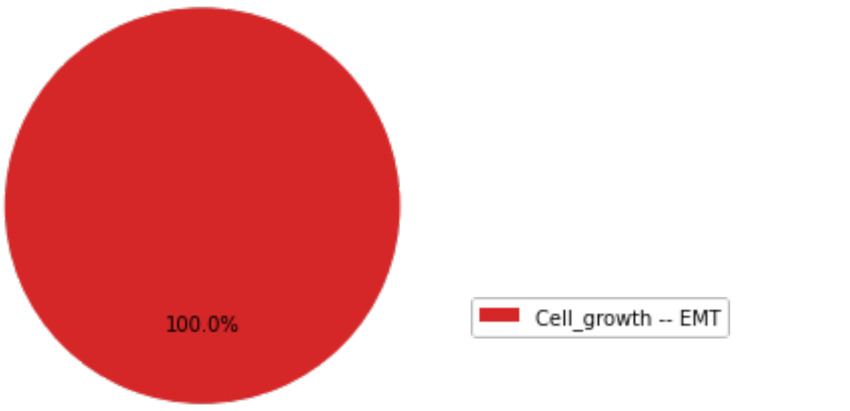

(('p73', 'ON'), ('YAP1', 'OFF'))

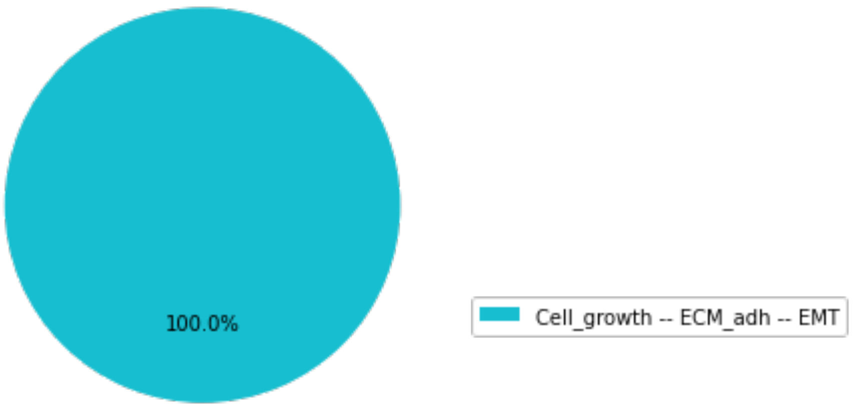

(('p73', 'ON'), ('PIK3CA', 'OFF'))

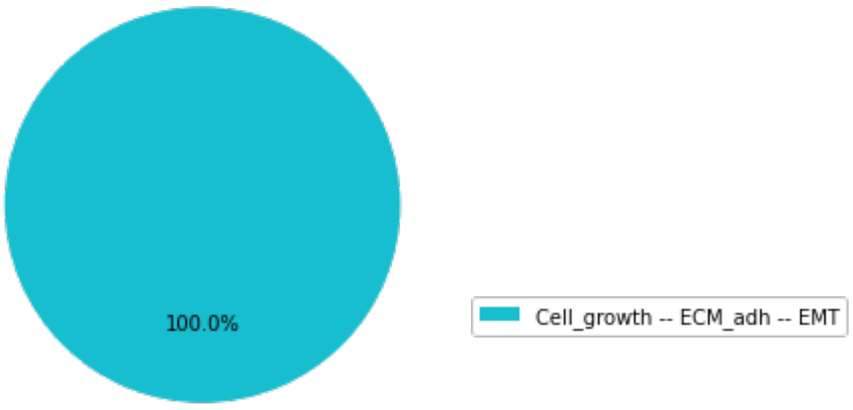

(('p73', 'ON'), ('ERK', 'OFF'))

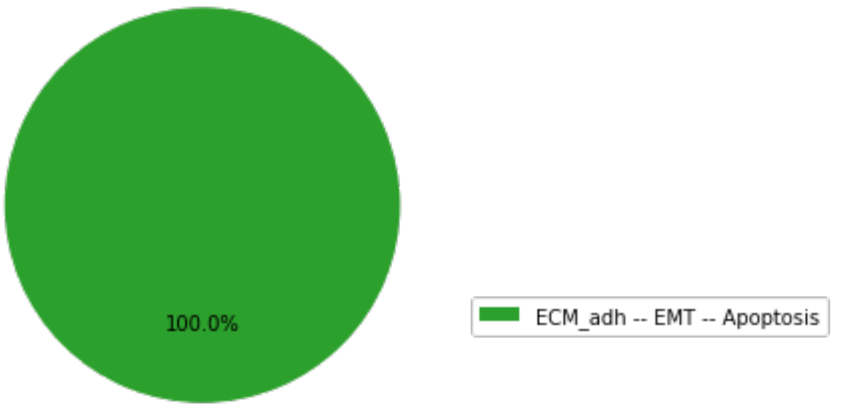

(('p73', 'ON'), ('p21', 'OFF'))

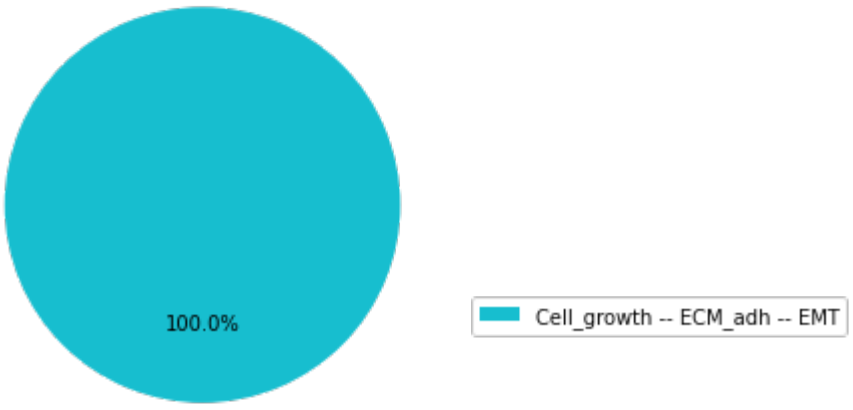

(('p73', 'ON'), ('SMAD', 'OFF'))

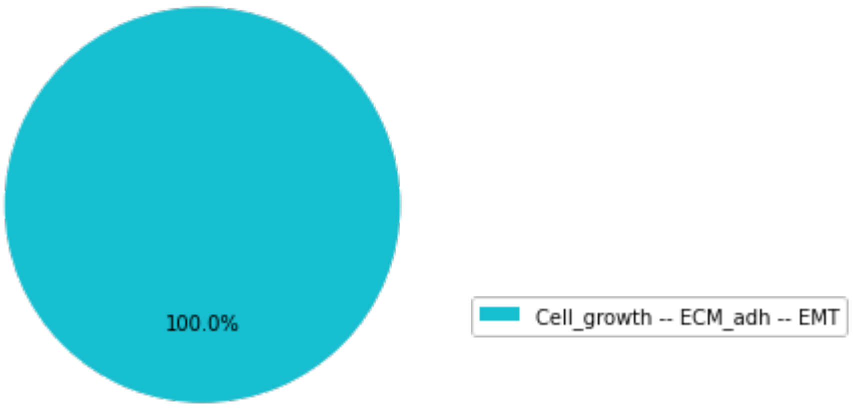

(('p73', 'ON'), ('VIM', 'OFF'))

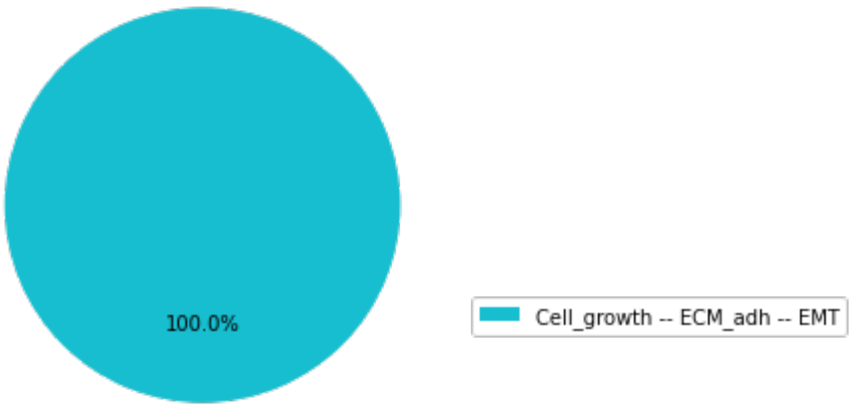

(('p73', 'ON'), ('p53', 'OFF'))

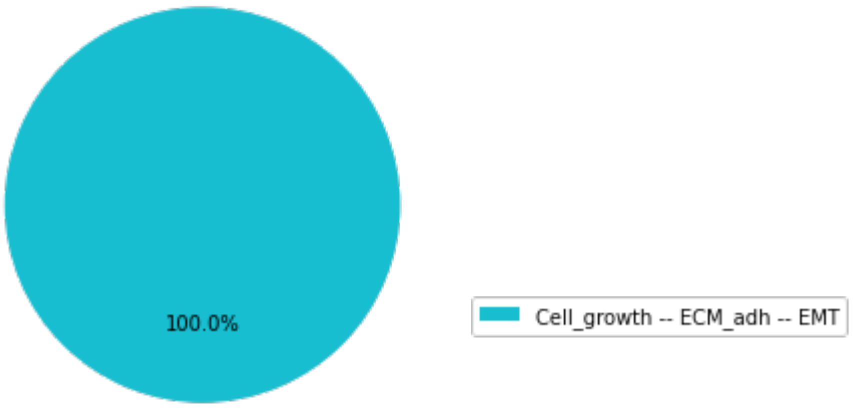

(('p73', 'ON'), ('p63', 'OFF'))

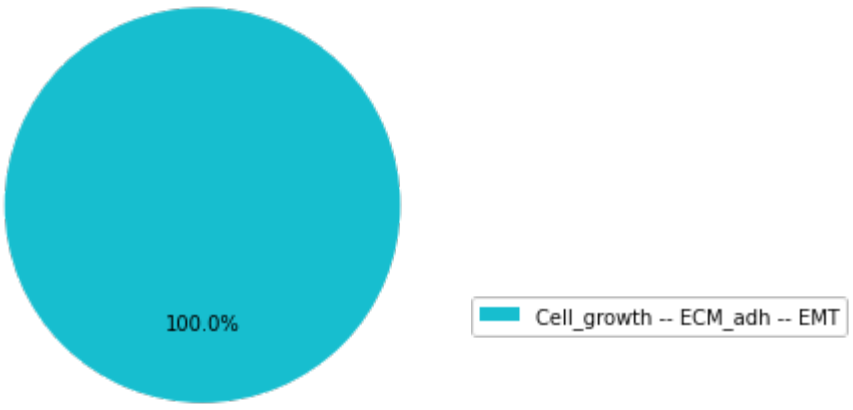

(('p73', 'ON'), ('miR203', 'OFF'))

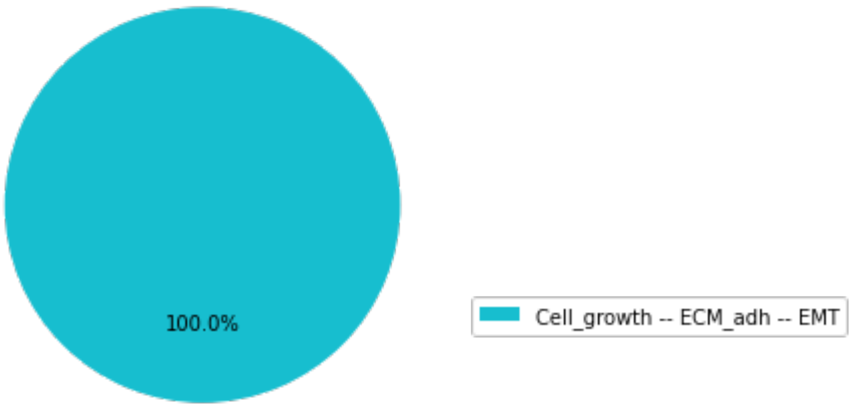

(('p73', 'ON'), ('miR34', 'OFF'))

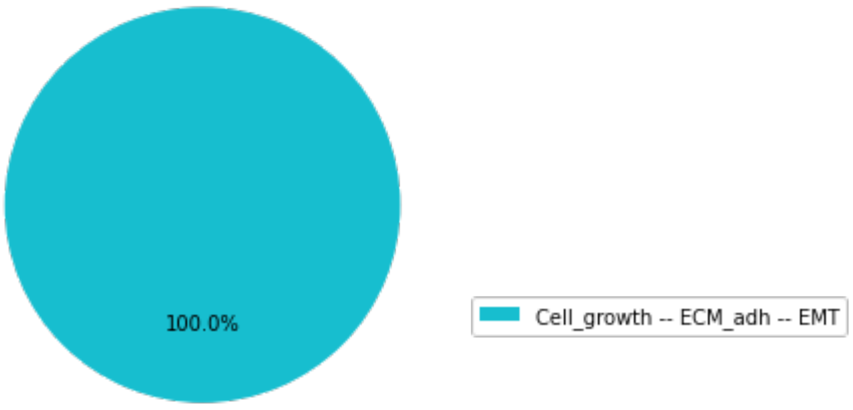

(('p73', 'ON'), ('miR200', 'OFF'))

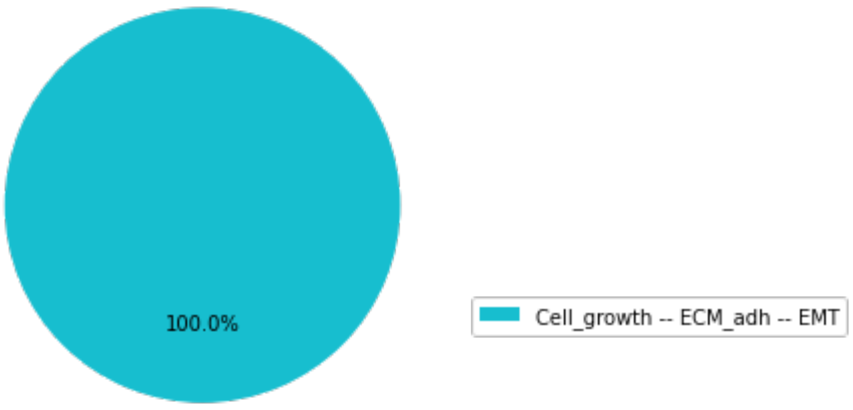

(('p73', 'ON'), ('TGFbetaR', 'OFF'))

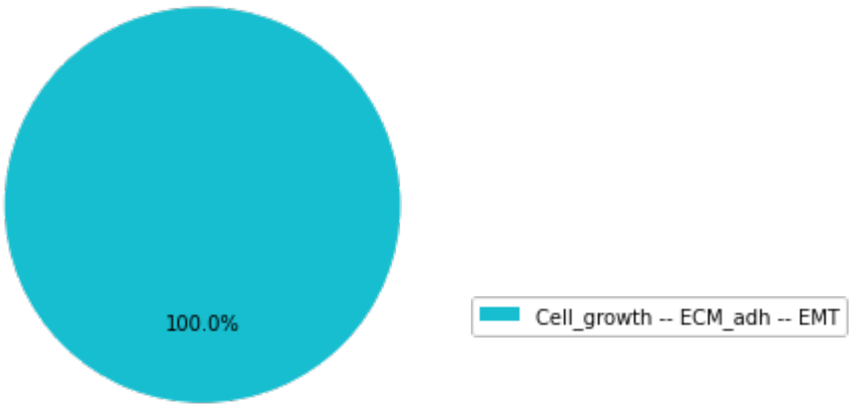

(('p73', 'ON'), ('FAK', 'OFF'))

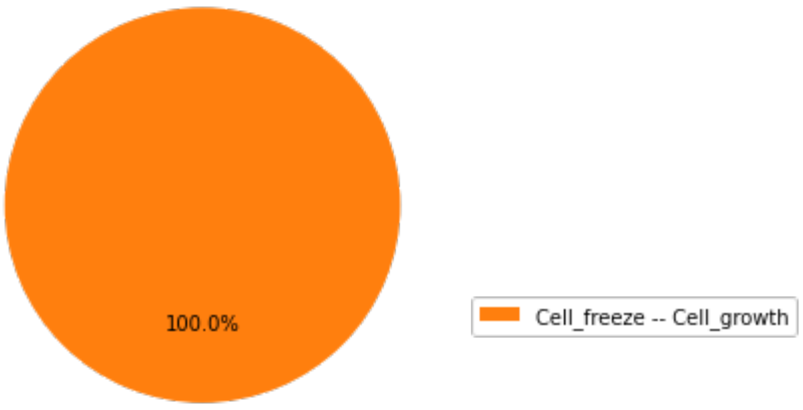

(('miR203', 'ON'), ('miR34', 'ON'))

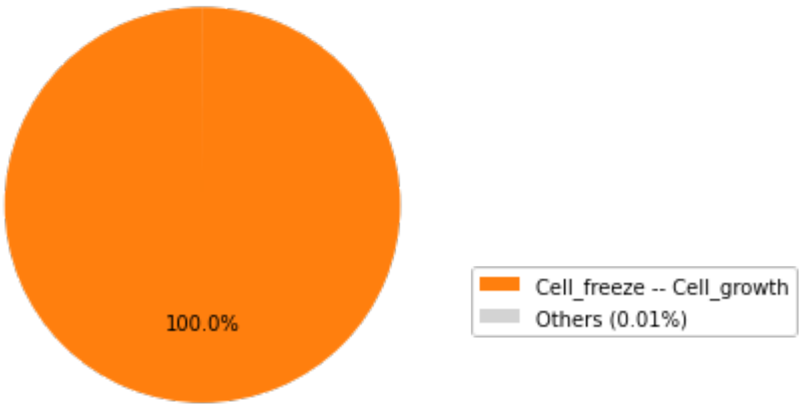

(('miR203', 'ON'), ('miR200', 'ON'))

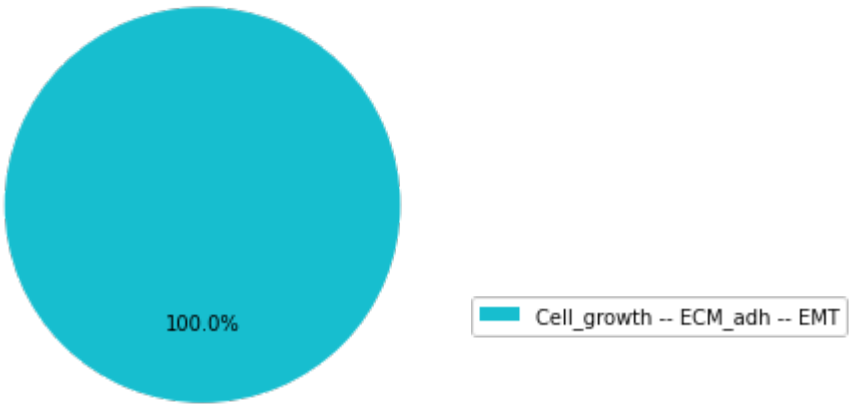

(('miR203', 'ON'), ('TGFbetaR', 'ON'))

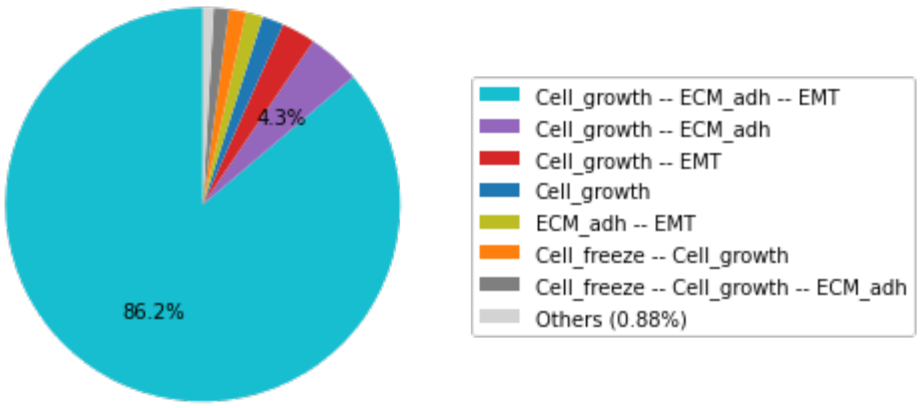

(('miR203', 'ON'), ('FAK', 'ON'))

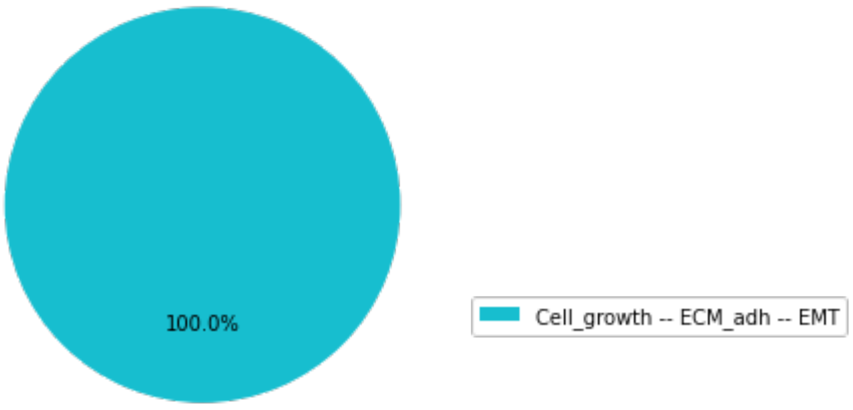

(('miR203', 'ON'), ('AKT1', 'OFF'))

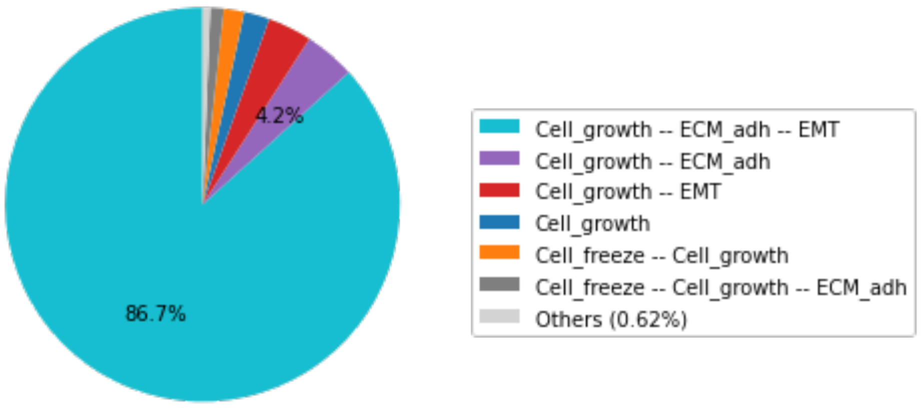

(('miR203', 'ON'), ('CTNNB1', 'OFF'))

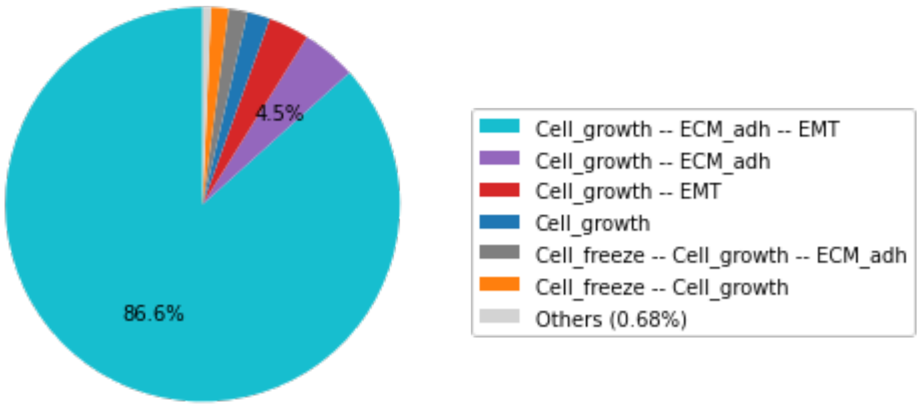

(('miR203', 'ON'), ('DKK1', 'OFF'))

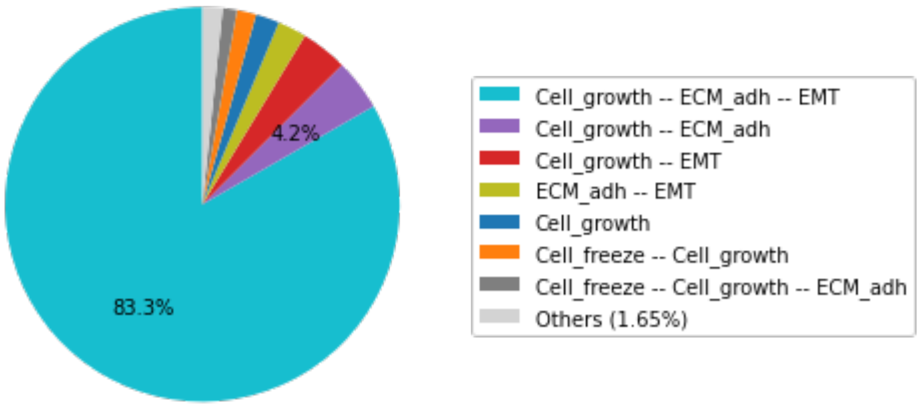

(('miR203', 'ON'), ('AKT2', 'OFF'))

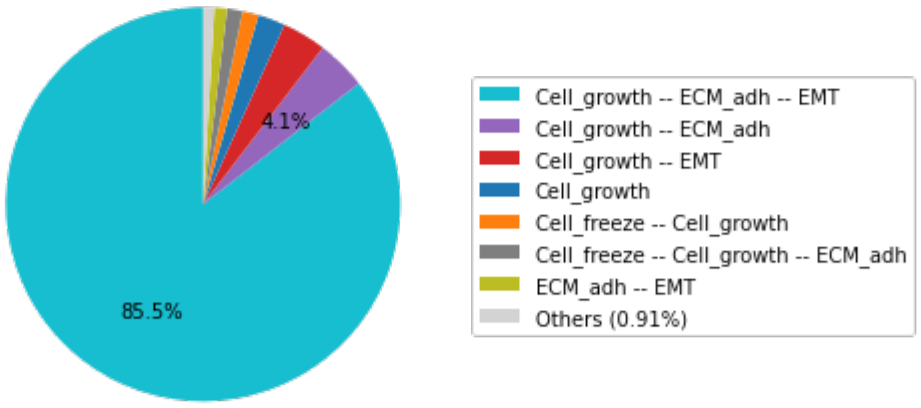

(('miR203', 'ON'), ('RAC1', 'OFF'))

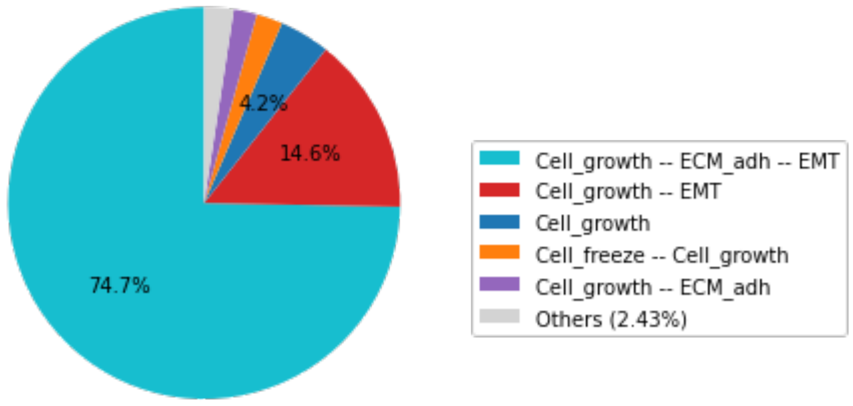

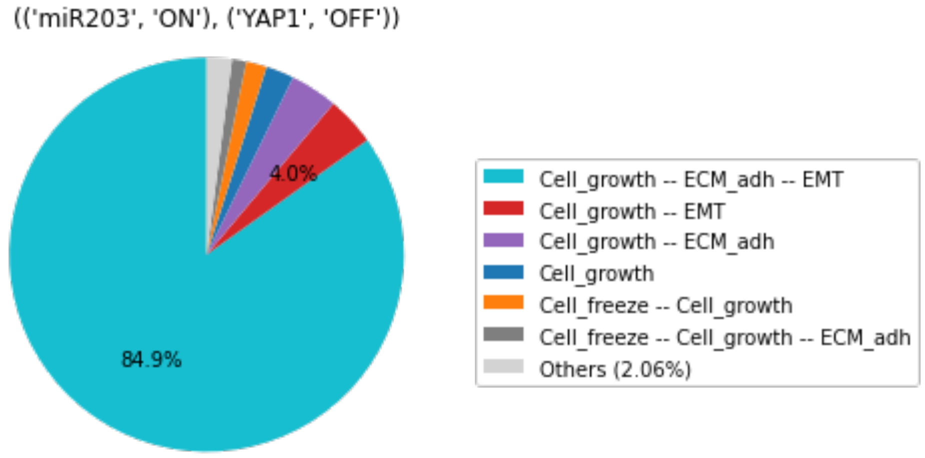
(('miR203', 'ON'), ('YAP1', 'OFF'))
4.0%
84.9%
Cell_growth -- ECM_adh -- EMT
Cell_growth -- EMT
Cell_growth -- ECM_adh
Cell_growth
Cell_freeze -- Cell_growth
Cell_freeze -- Cell_growth -- ECM_adh
Others (2.06%)

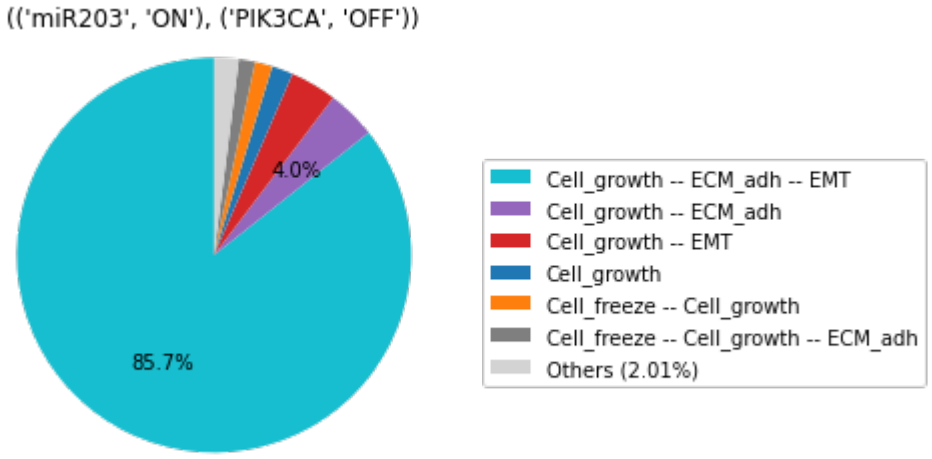
(('miR203', 'ON'), ('PIK3CA', 'OFF'))
4.0%
85.7%
Cell_growth -- ECM_adh -- EMT
Cell_growth -- ECM_adh
Cell_growth -- EMT
Cell_growth
Cell_freeze -- Cell_growth
Cell_freeze -- Cell_growth -- ECM_adh
Others (2.01%)

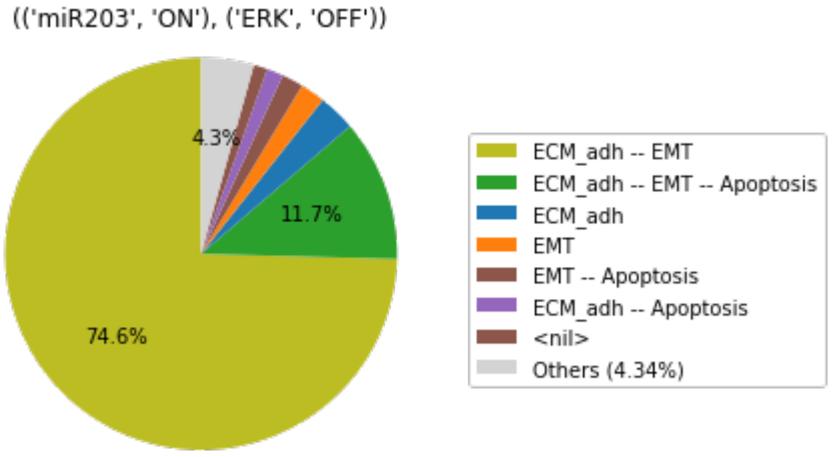
(('miR203', 'ON'), ('ERK', 'OFF'))
4.3%
11.7%
74.6%
ECM_adh -- EMT
ECM_adh -- EMT -- Apoptosis
ECM_adh
EMT
EMT -- Apoptosis
ECM_adh -- Apoptosis
<nil>
Others (4.34%)

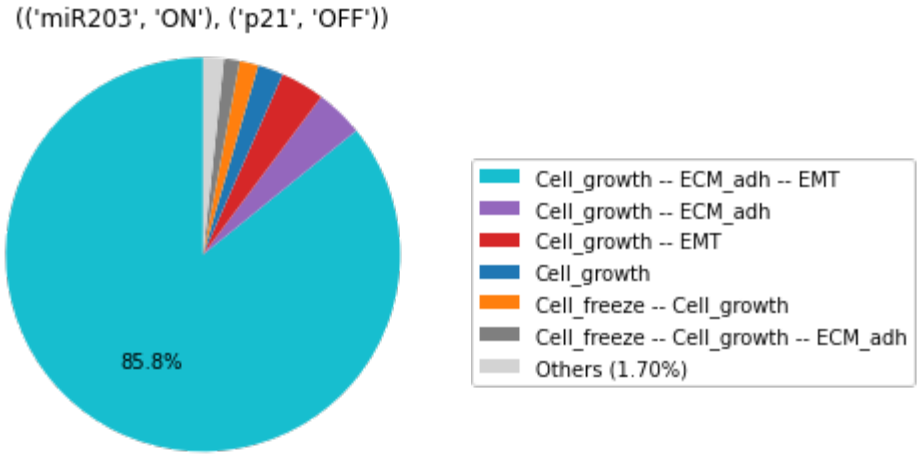
(('miR203', 'ON'), ('p21', 'OFF'))
85.8%
Cell_growth -- ECM_adh -- EMT
Cell_growth -- ECM_adh
Cell_growth -- EMT
Cell_growth
Cell_freeze -- Cell_growth
Cell_freeze -- Cell_growth -- ECM_adh
Others (1.70%)

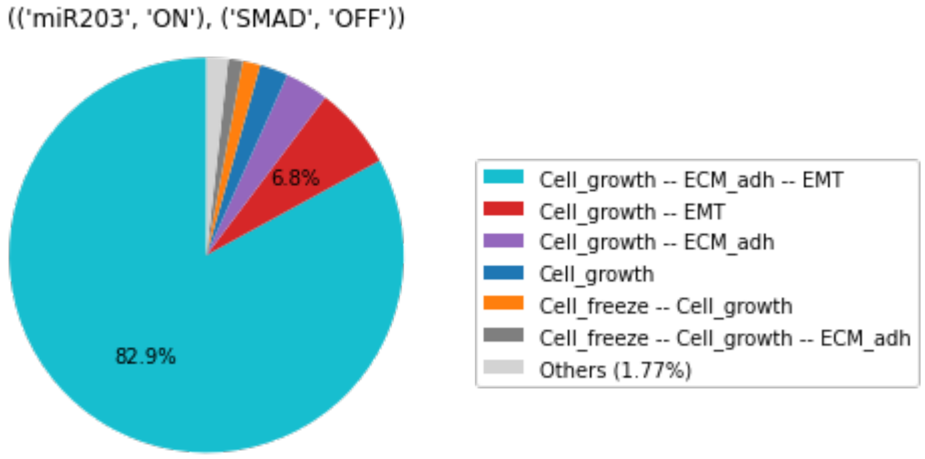
(('miR203', 'ON'), ('SMAD', 'OFF'))
6.8%
82.9%
Cell_growth -- ECM_adh -- EMT
Cell_growth -- EMT
Cell_growth -- ECM_adh
Cell_growth
Cell_freeze -- Cell_growth
Cell_freeze -- Cell_growth -- ECM_adh
Others (1.77%)

(('miR203', 'ON'), ('VIM', 'OFF'))

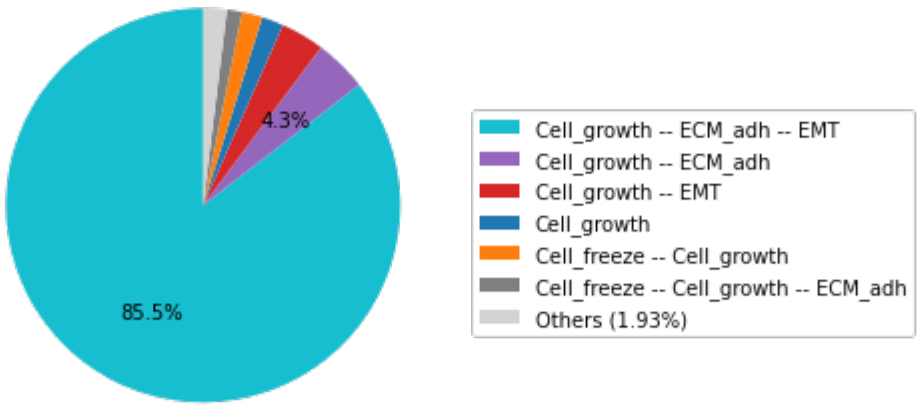

(('miR203', 'ON'), ('p53', 'OFF'))

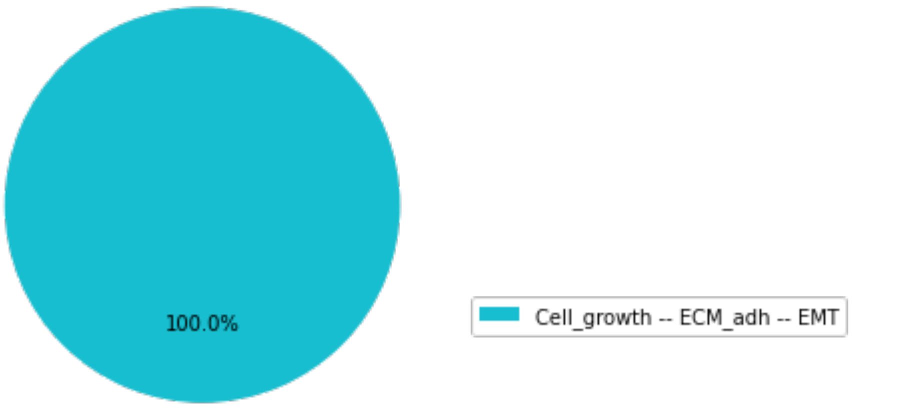

(('miR203', 'ON'), ('p63', 'OFF'))

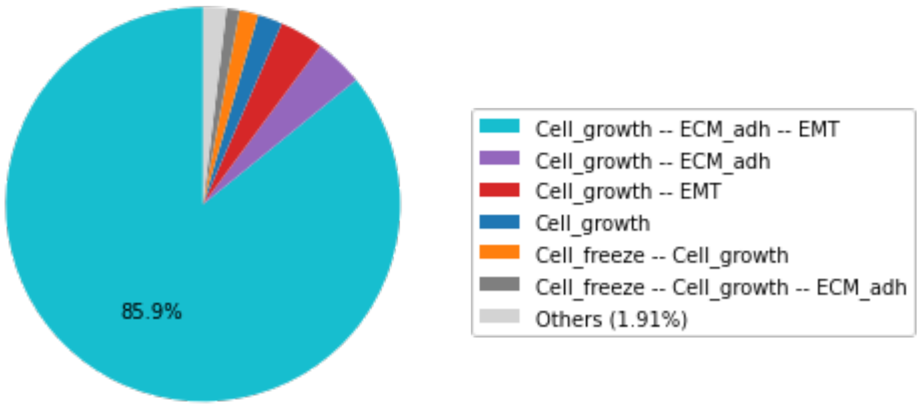

(('miR203', 'ON'), ('p73', 'OFF'))

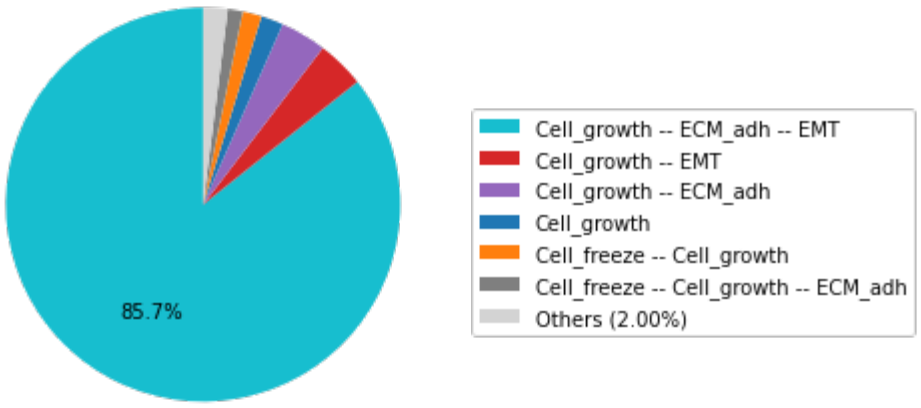

(('miR203', 'ON'), ('miR34', 'OFF'))

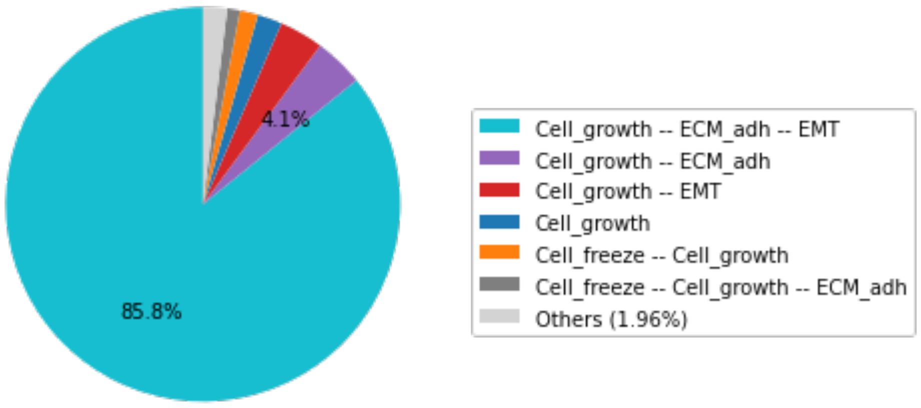

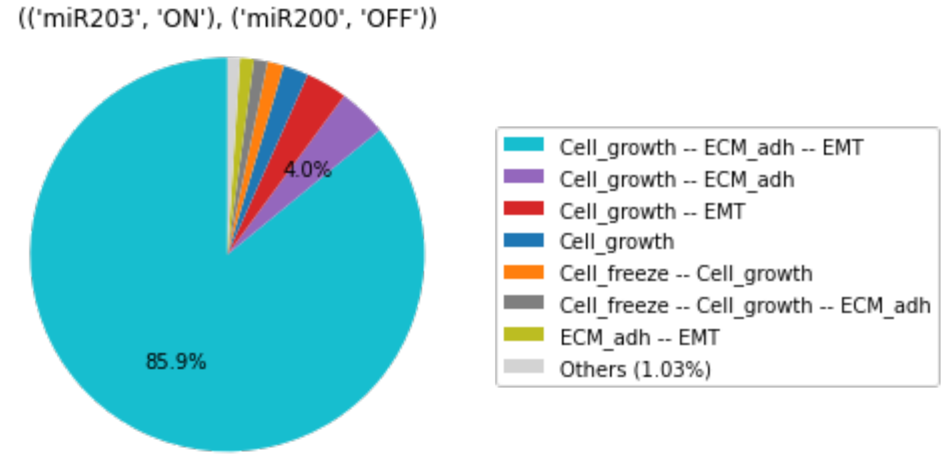
(('miR203', 'ON'), ('miR200', 'OFF'))
4.0%
85.9%
Cell_growth -- ECM_adh -- EMT
Cell_growth -- ECM_adh
Cell_growth -- EMT
Cell_growth
Cell_freeze -- Cell_growth
Cell_freeze -- Cell_growth -- ECM_adh
ECM_adh -- EMT
Others (1.03%)

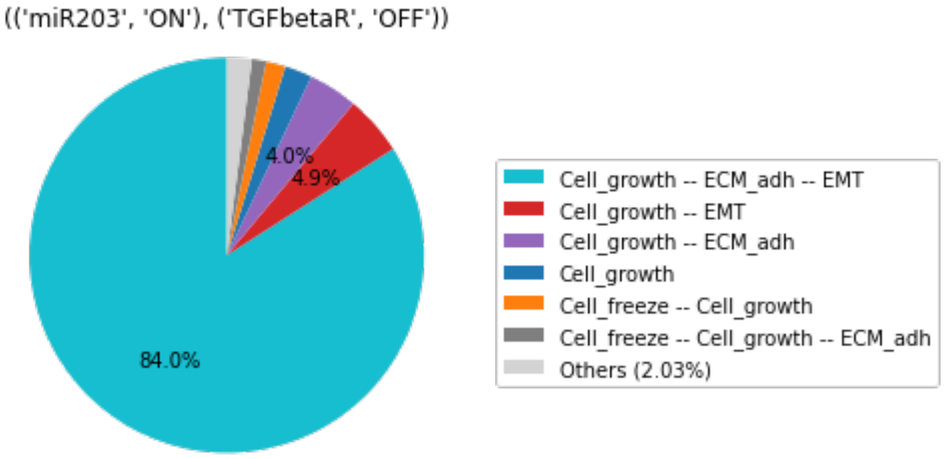
(('miR203', 'ON'), ('TGFbetaR', 'OFF'))
4.0%
4.9%
84.0%
Cell_growth -- ECM_adh -- EMT
Cell_growth -- EMT
Cell_growth -- ECM_adh
Cell_growth
Cell_freeze -- Cell_growth
Cell_freeze -- Cell_growth -- ECM_adh
Others (2.03%)

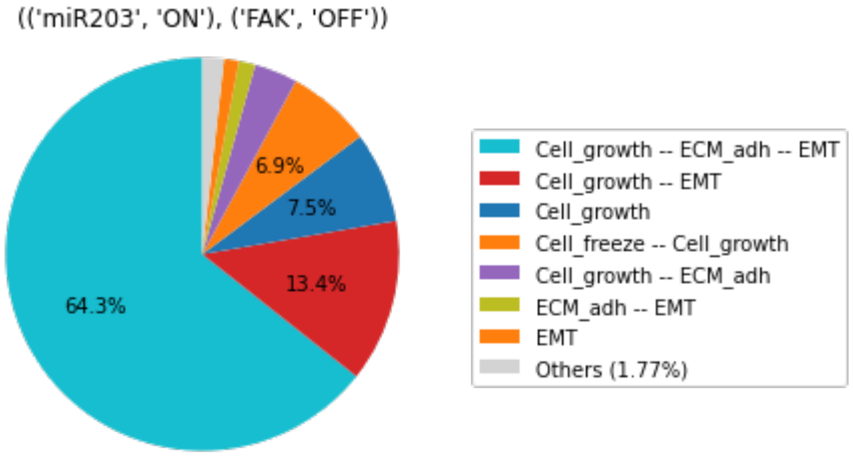
(('miR203', 'ON'), ('FAK', 'OFF'))
6.9%
7.5%
13.4%
64.3%
Cell_growth -- ECM_adh -- EMT
Cell_growth -- EMT
Cell_growth
Cell_freeze -- Cell_growth
Cell_growth -- ECM_adh
ECM_adh -- EMT
EMT
Others (1.77%)

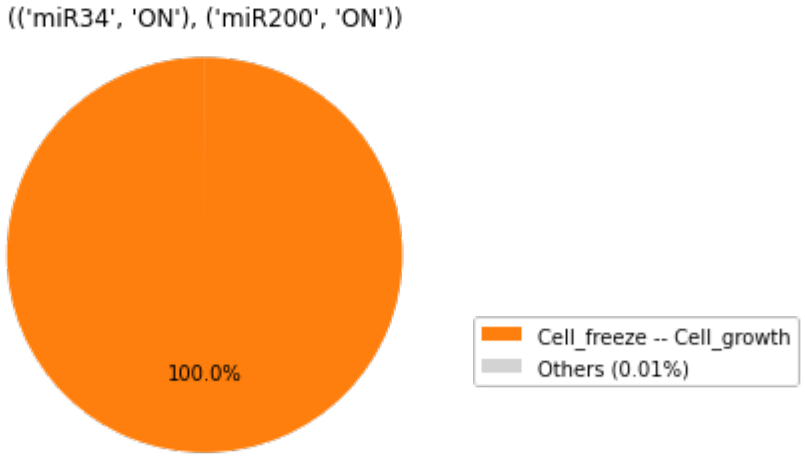
(('miR34', 'ON'), ('miR200', 'ON'))
100.0%
Cell_freeze -- Cell_growth
Others (0.01%)

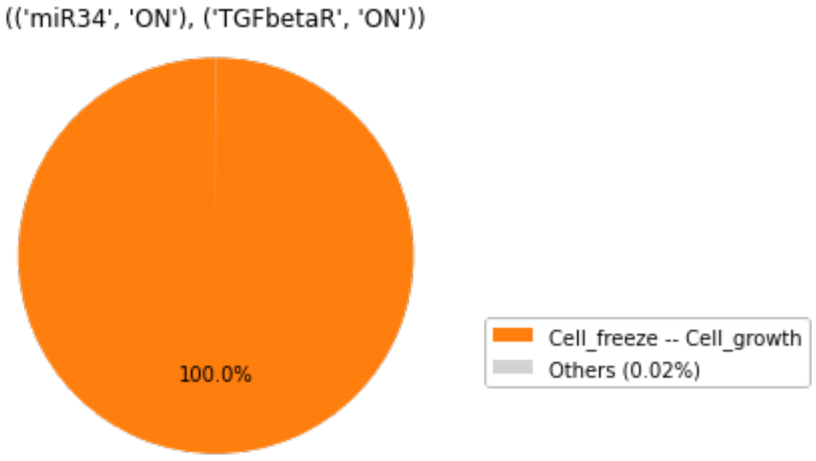
(('miR34', 'ON'), ('TGFbetaR', 'ON'))
100.0%
Cell_freeze -- Cell_growth
Others (0.02%)

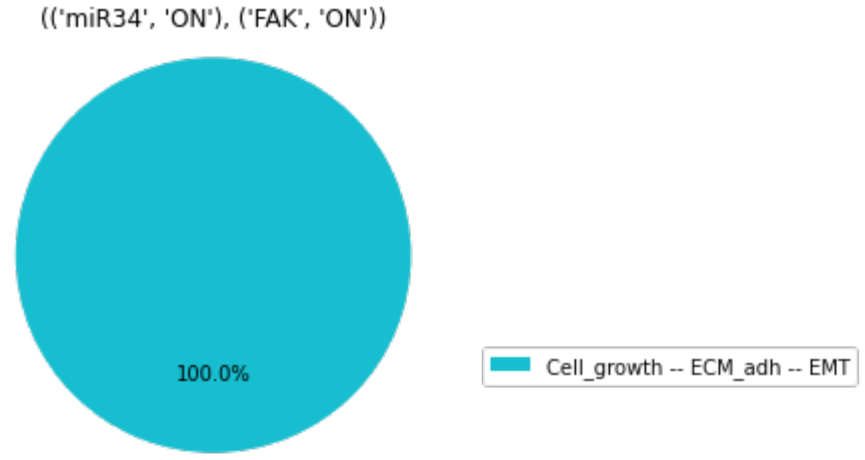
(('miR34', 'ON'), ('FAK', 'ON'))
100.0%
Cell_growth -- ECM_adh -- EMT

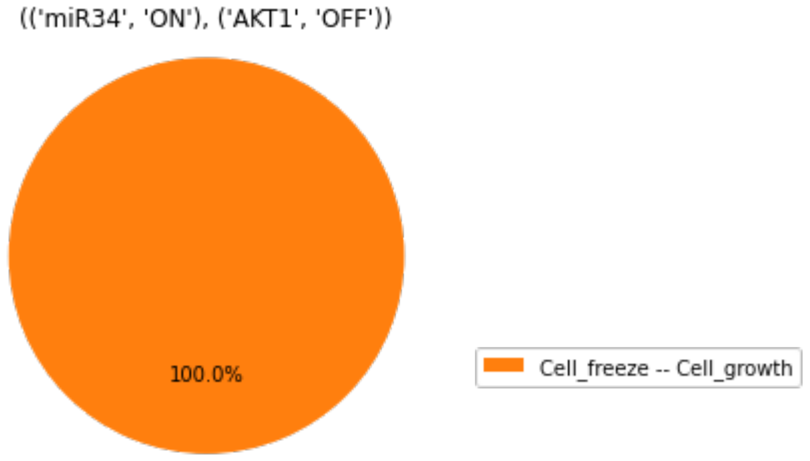
(('miR34', 'ON'), ('AKT1', 'OFF'))
100.0%
Cell_freeze -- Cell_growth

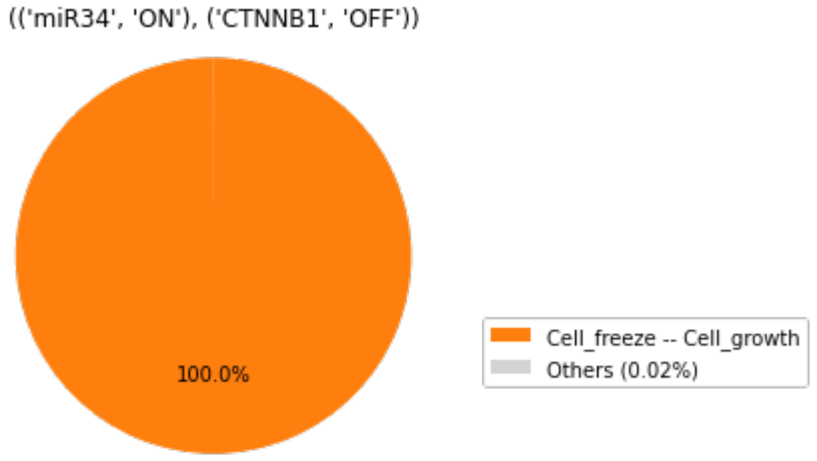
(('miR34', 'ON'), ('CTNNB1', 'OFF'))
100.0%
Cell_freeze -- Cell_growth
Others (0.02%)

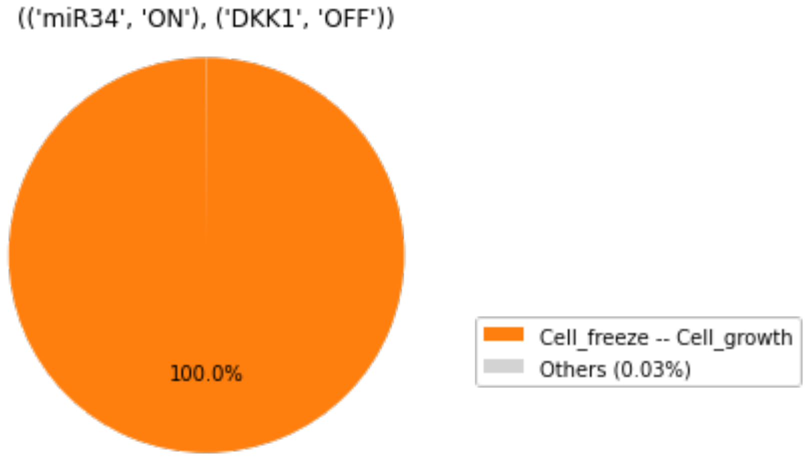
(('miR34', 'ON'), ('DKK1', 'OFF'))
100.0%
Cell_freeze -- Cell_growth
Others (0.03%)

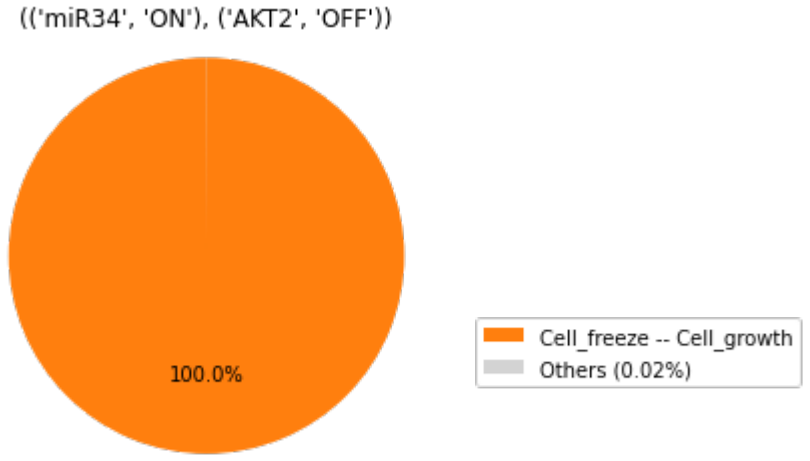
(('miR34', 'ON'), ('AKT2', 'OFF'))
100.0%
Cell_freeze -- Cell_growth
Others (0.02%)

(('miR34', 'ON'), ('RAC1', 'OFF'))

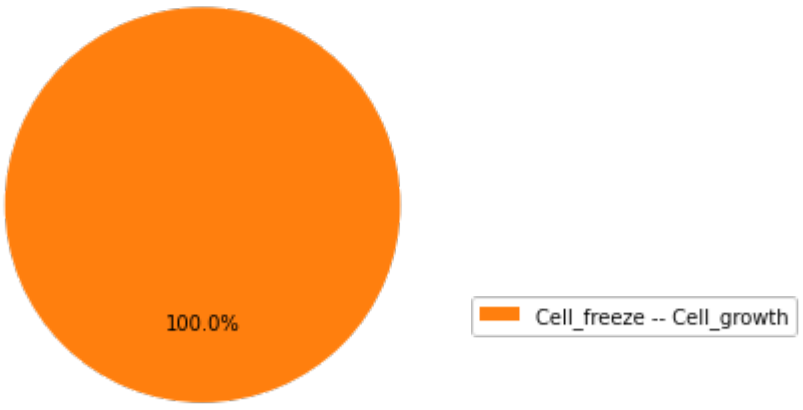

(('miR34', 'ON'), ('YAP1', 'OFF'))

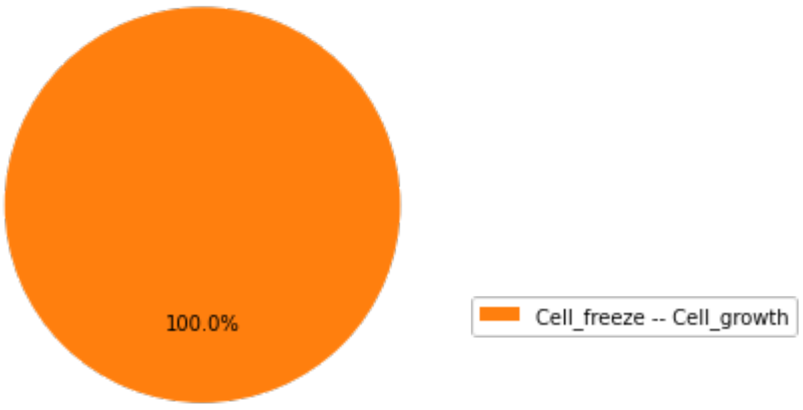

(('miR34', 'ON'), ('PIK3CA', 'OFF'))

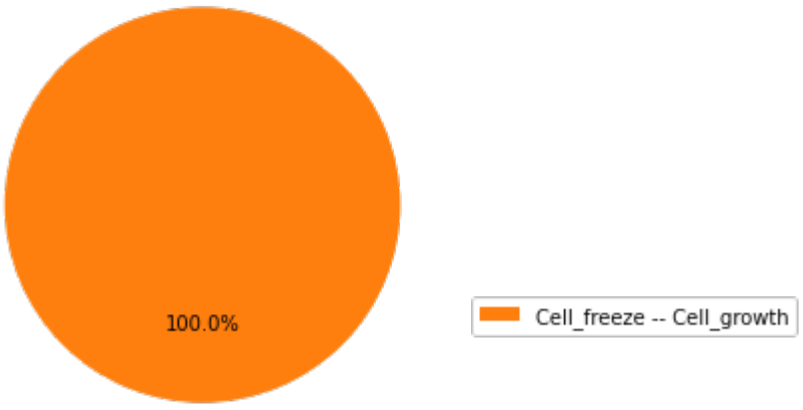

(('miR34', 'ON'), ('ERK', 'OFF'))

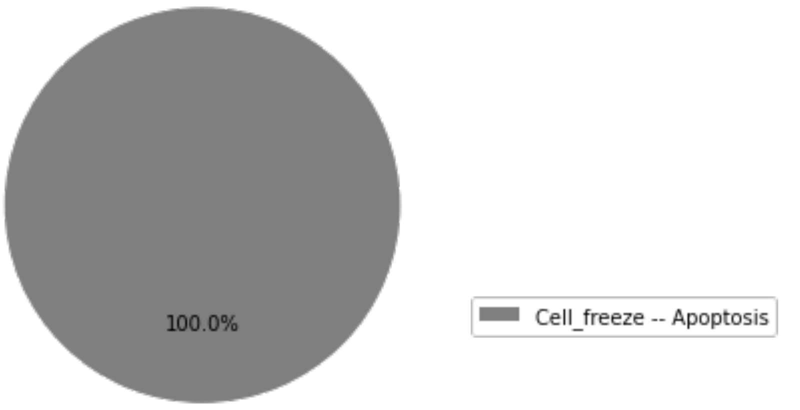

(('miR34', 'ON'), ('p21', 'OFF'))

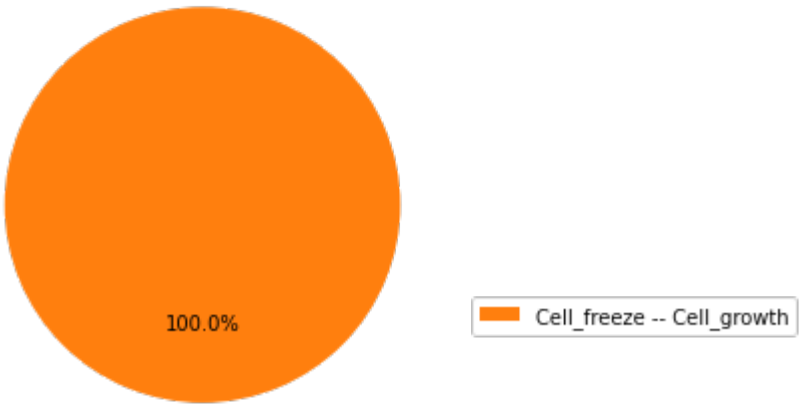

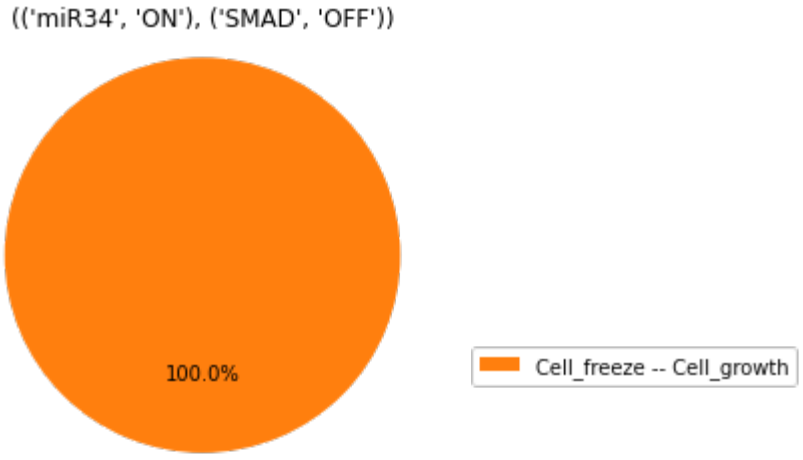
(('miR34', 'ON'), ('SMAD', 'OFF'))
100.0%
Cell_freeze -- Cell_growth

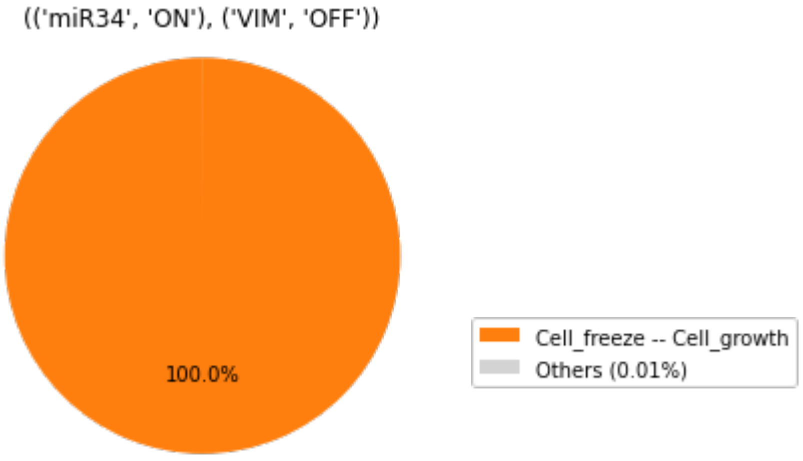
(('miR34', 'ON'), ('VIM', 'OFF'))
100.0%
Cell_freeze -- Cell_growth
Others (0.01%)

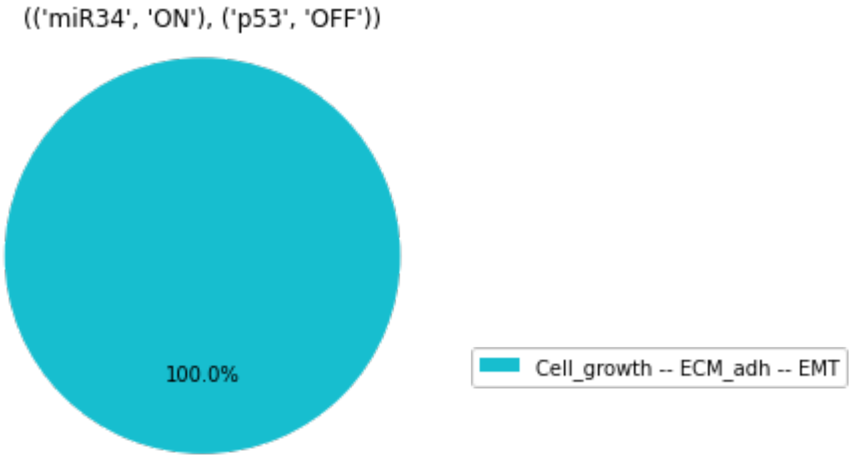
(('miR34', 'ON'), ('p53', 'OFF'))
100.0%
Cell_growth -- ECM_adh -- EMT

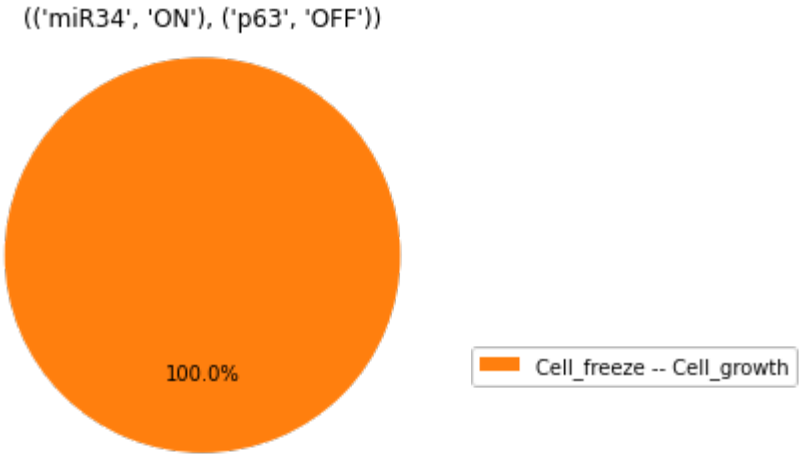
(('miR34', 'ON'), ('p63', 'OFF'))
100.0%
Cell_freeze -- Cell_growth

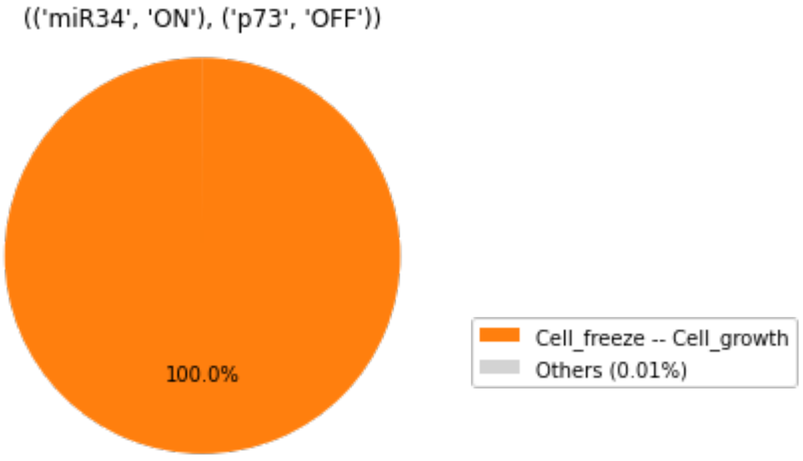
(('miR34', 'ON'), ('p73', 'OFF'))
100.0%
Cell_freeze -- Cell_growth
Others (0.01%)

(('miR34', 'ON'), ('miR203', 'OFF'))

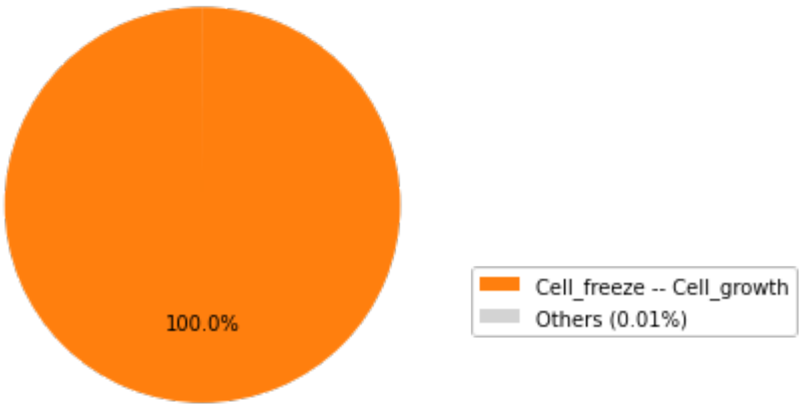

(('miR34', 'ON'), ('miR200', 'OFF'))

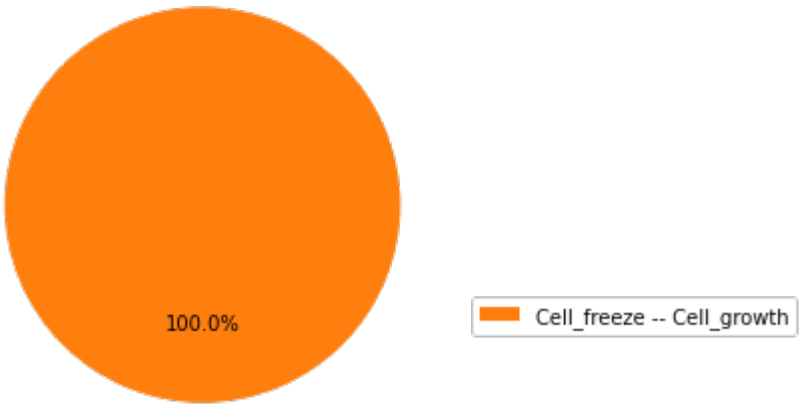

(('miR34', 'ON'), ('TGFbetaR', 'OFF'))

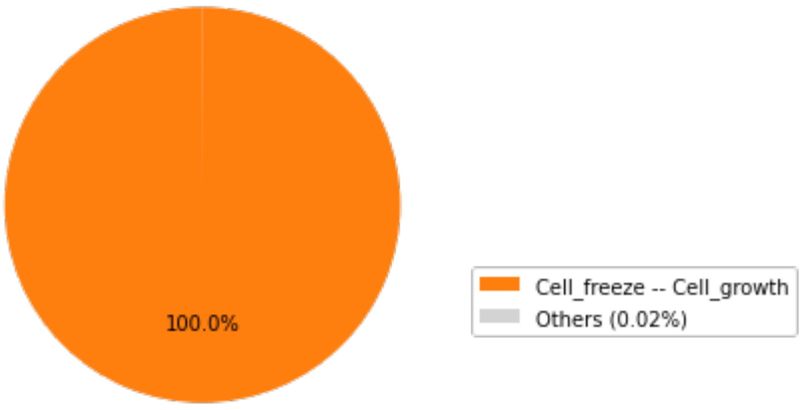

(('miR34', 'ON'), ('FAK', 'OFF'))

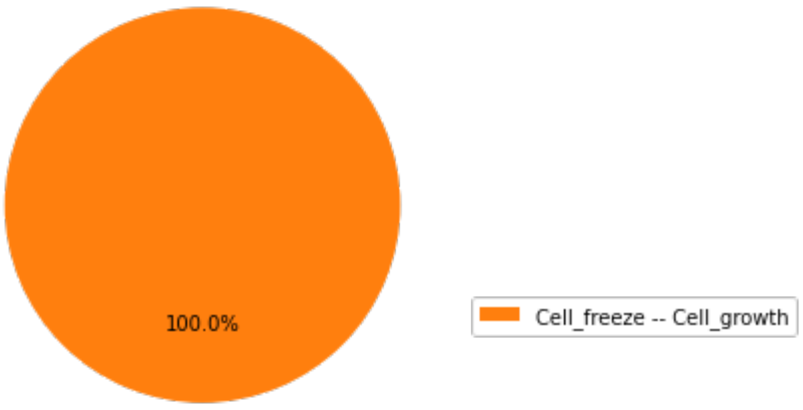

(('miR200', 'ON'), ('TGFbetaR', 'ON'))

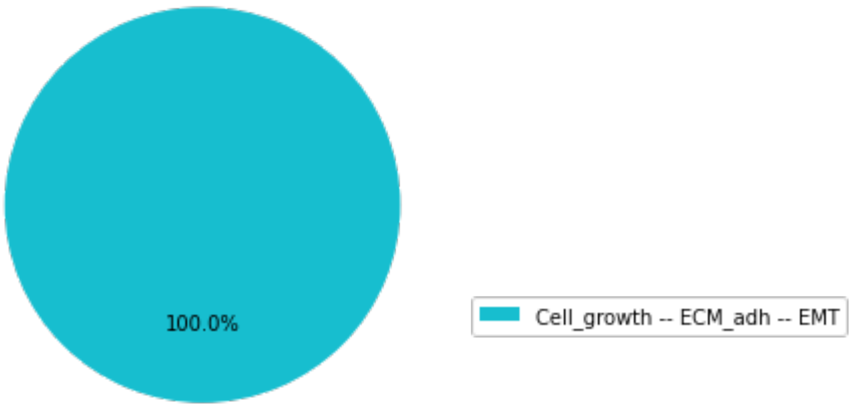

(('miR200', 'ON'), ('FAK', 'ON'))

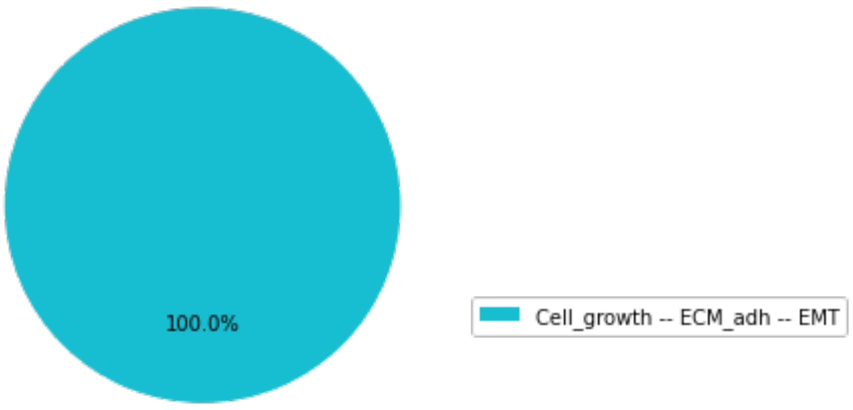

(('miR200', 'ON'), ('AKT1', 'OFF'))

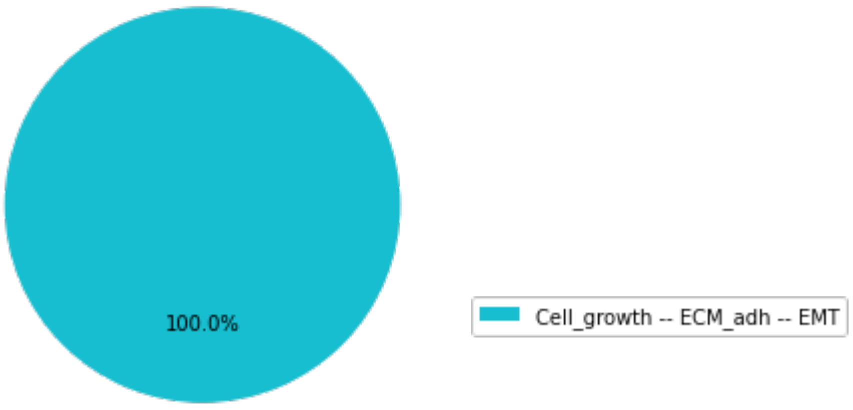

(('miR200', 'ON'), ('CTNNB1', 'OFF'))

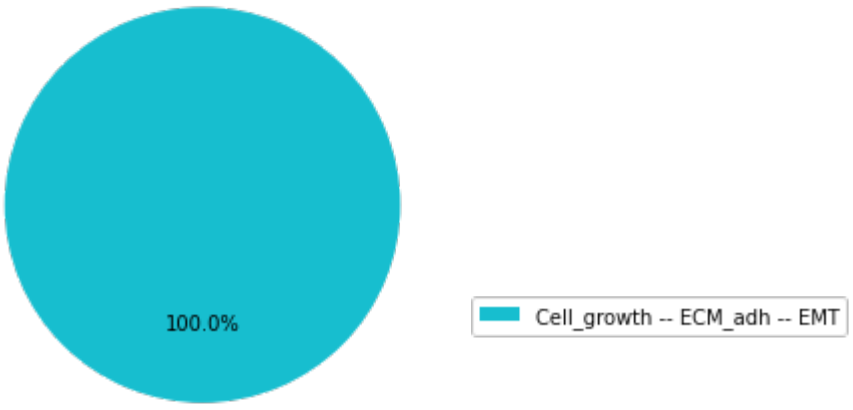

(('miR200', 'ON'), ('DKK1', 'OFF'))

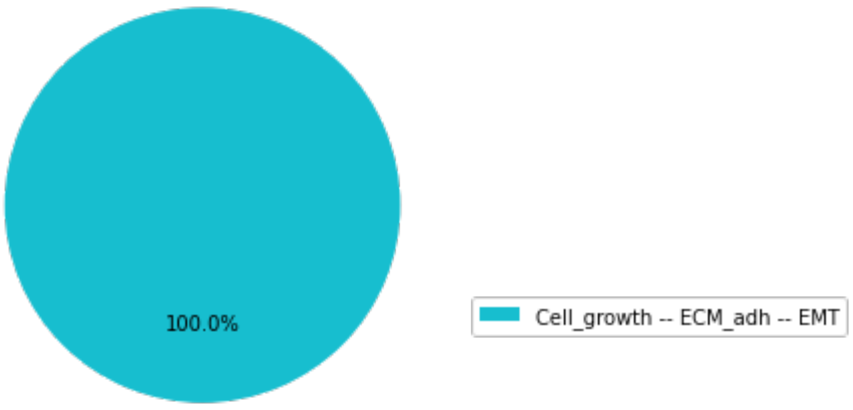

(('miR200', 'ON'), ('AKT2', 'OFF'))

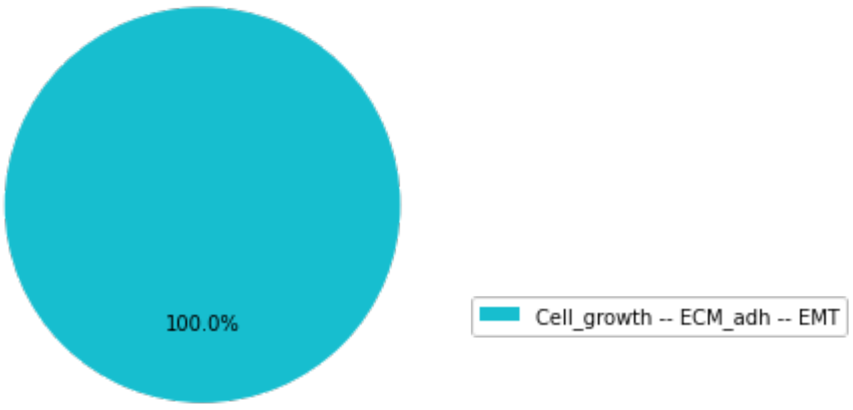

(('miR200', 'ON'), ('RAC1', 'OFF'))

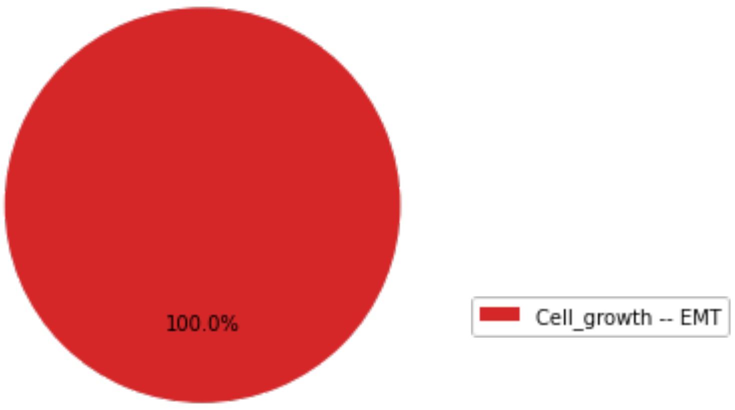

(('miR200', 'ON'), ('YAP1', 'OFF'))

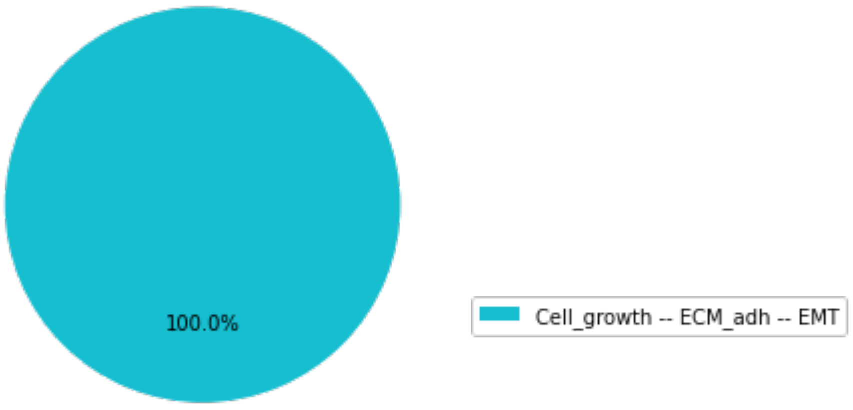

(('miR200', 'ON'), ('PIK3CA', 'OFF'))

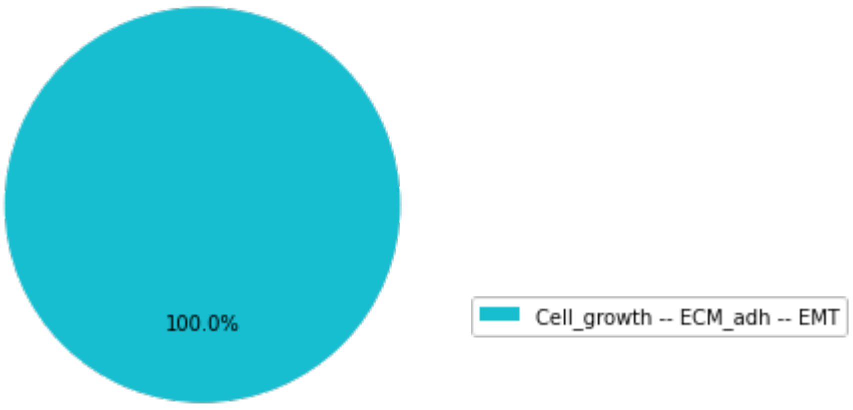

(('miR200', 'ON'), ('ERK', 'OFF'))

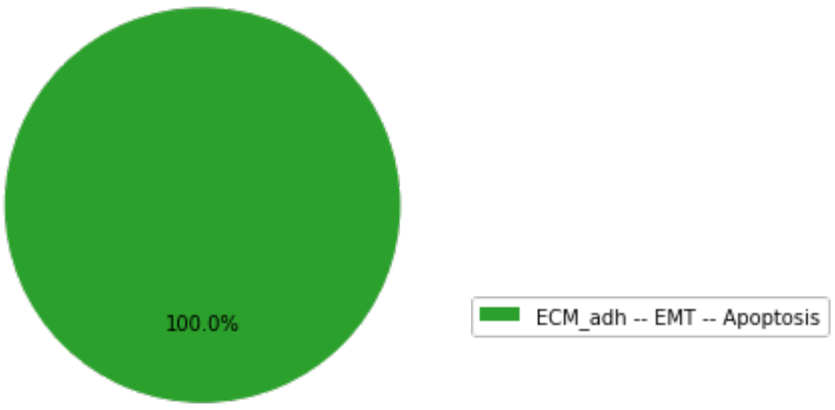

(('miR200', 'ON'), ('p21', 'OFF'))

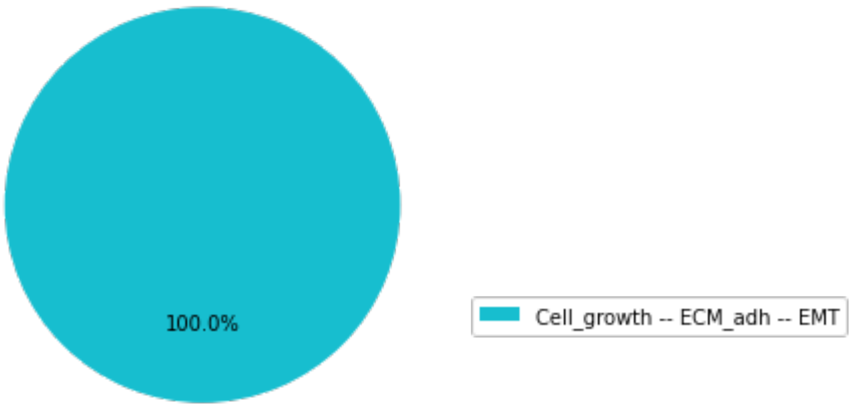

(('miR200', 'ON'), ('SMAD', 'OFF'))

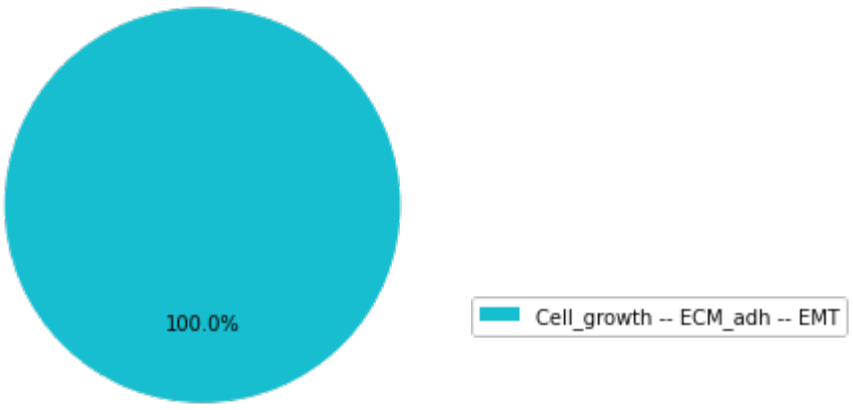

(('miR200', 'ON'), ('VIM', 'OFF'))

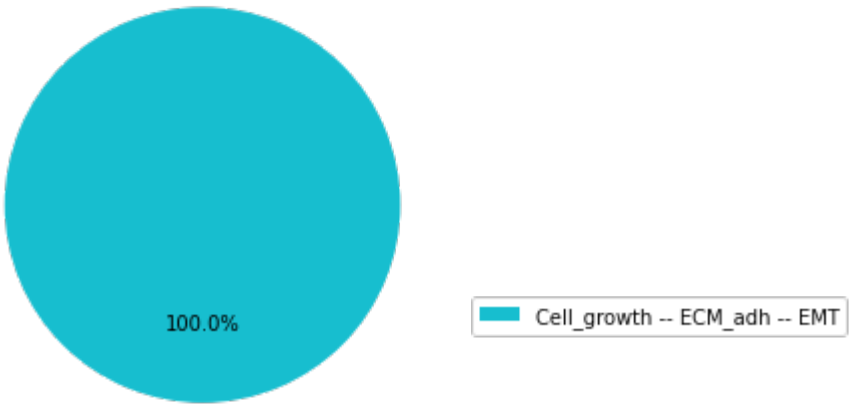

(('miR200', 'ON'), ('p53', 'OFF'))

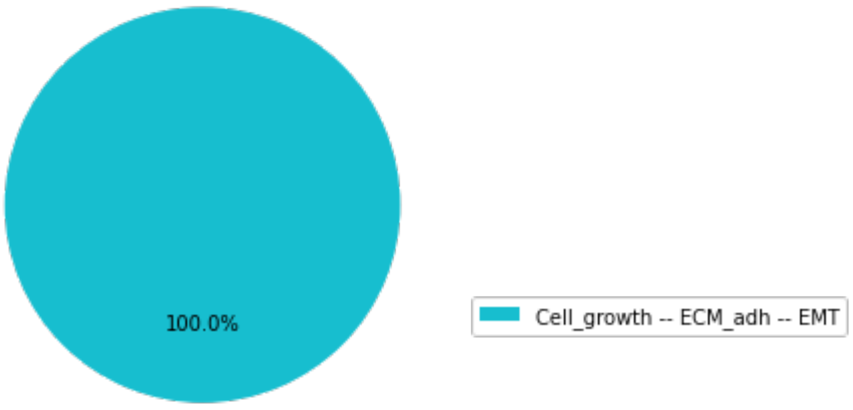

(('miR200', 'ON'), ('p63', 'OFF'))

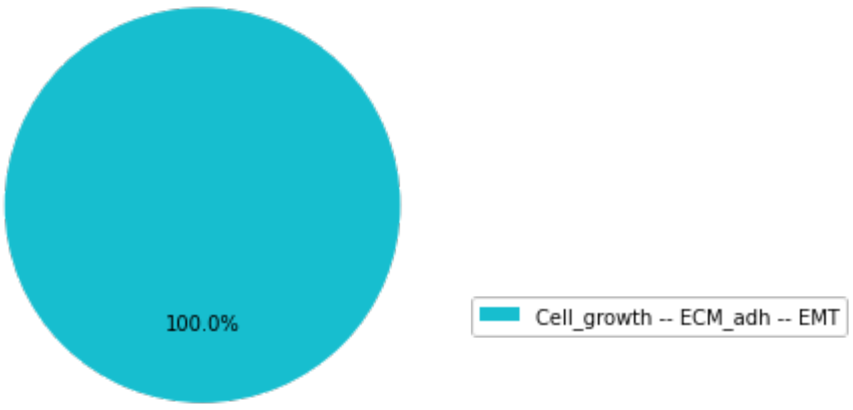

(('miR200', 'ON'), ('p73', 'OFF'))

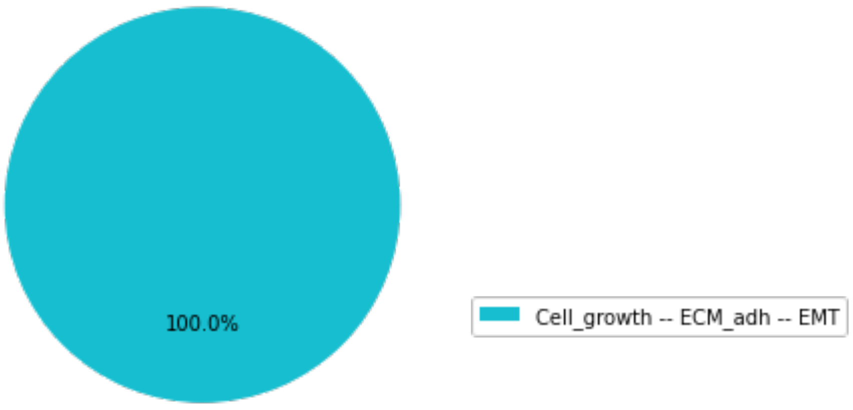

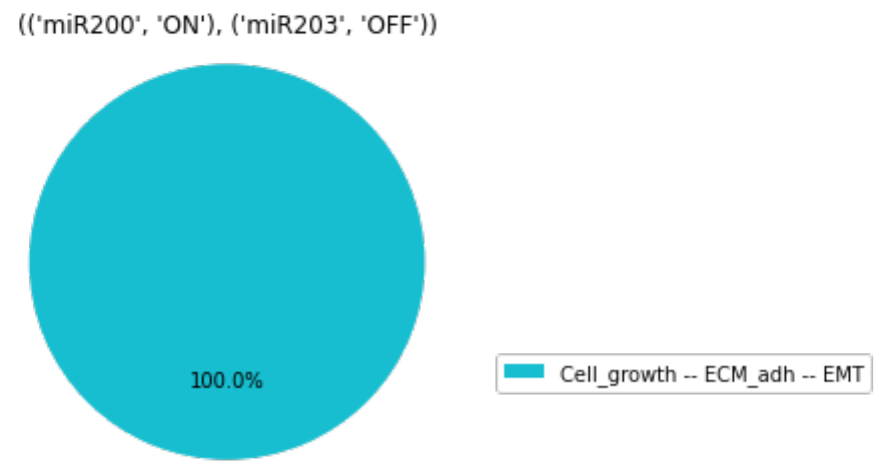
(('miR200', 'ON'), ('miR203', 'OFF'))
100.0%
Cell_growth -- ECM_adh -- EMT

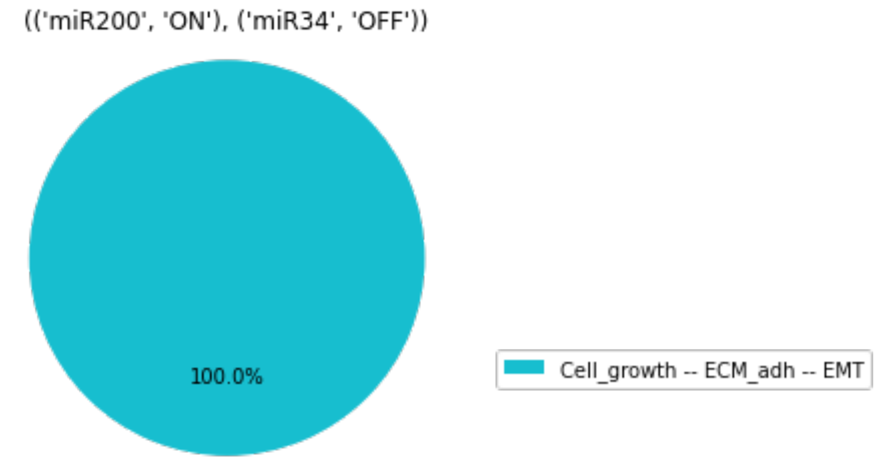
(('miR200', 'ON'), ('miR34', 'OFF'))
100.0%
Cell_growth -- ECM_adh -- EMT

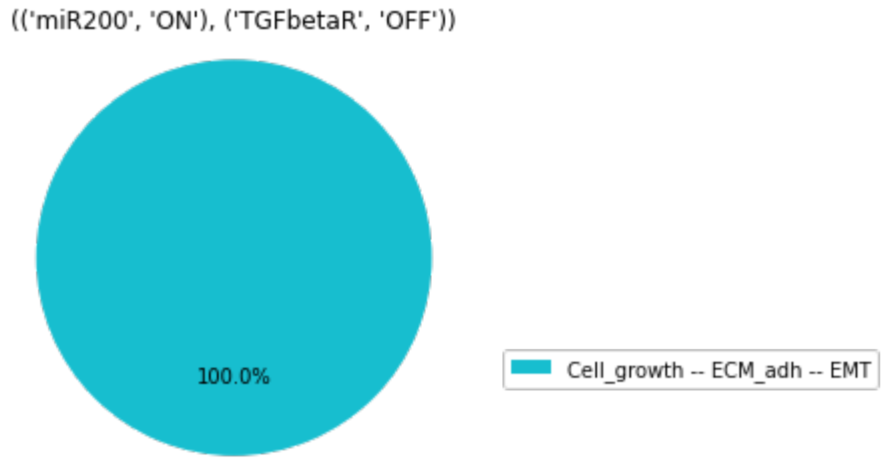
(('miR200', 'ON'), ('TGFbetaR', 'OFF'))
100.0%
Cell_growth -- ECM_adh -- EMT

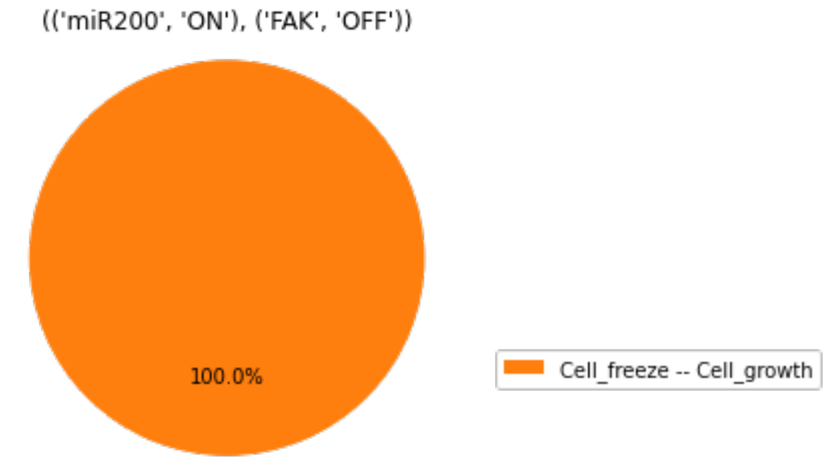
(('miR200', 'ON'), ('FAK', 'OFF'))
100.0%
Cell_freeze -- Cell_growth

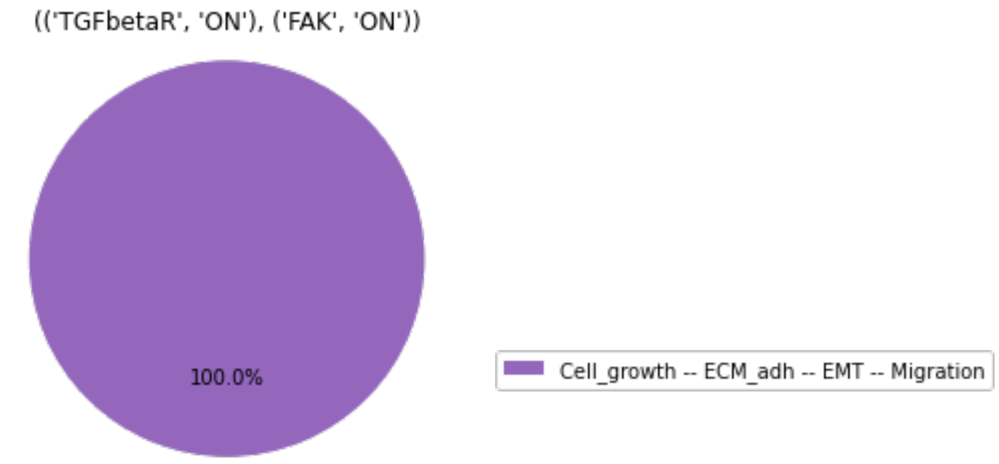
(('TGFbetaR', 'ON'), ('FAK', 'ON'))
100.0%
Cell_growth -- ECM_adh -- EMT -- Migration

(('TGFbetaR', 'ON'), ('AKT1', 'OFF'))

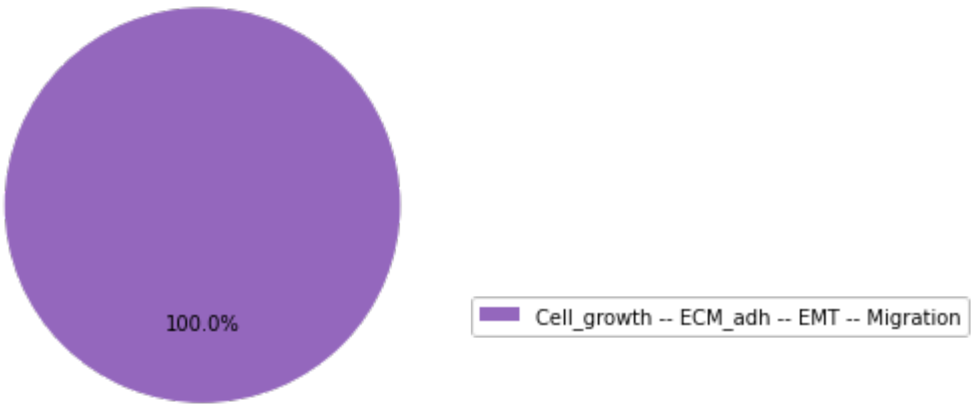

(('TGFbetaR', 'ON'), ('CTNNB1', 'OFF'))

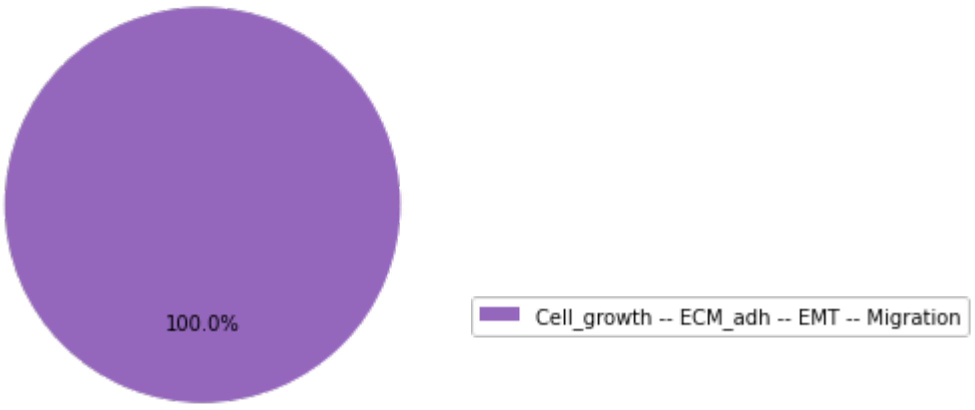

(('TGFbetaR', 'ON'), ('DKK1', 'OFF'))

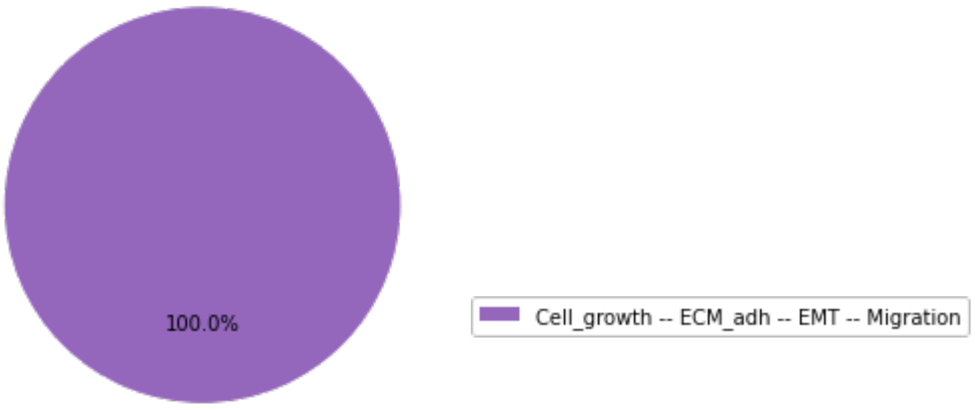

(('TGFbetaR', 'ON'), ('AKT2', 'OFF'))

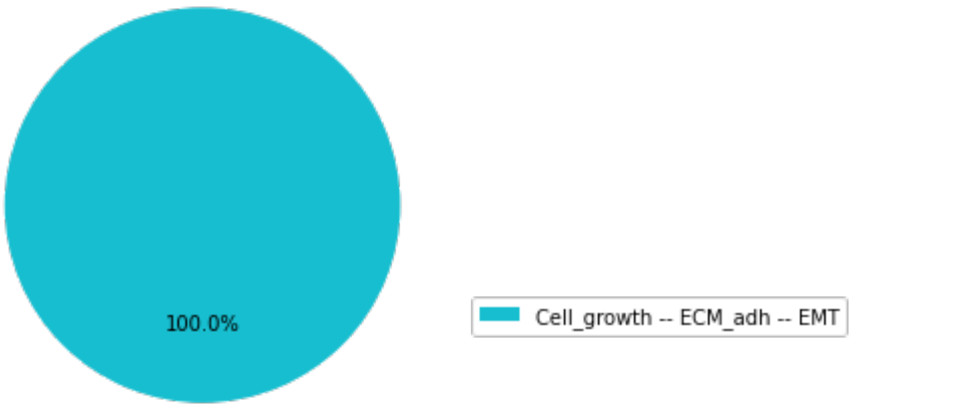

(('TGFbetaR', 'ON'), ('RAC1', 'OFF'))

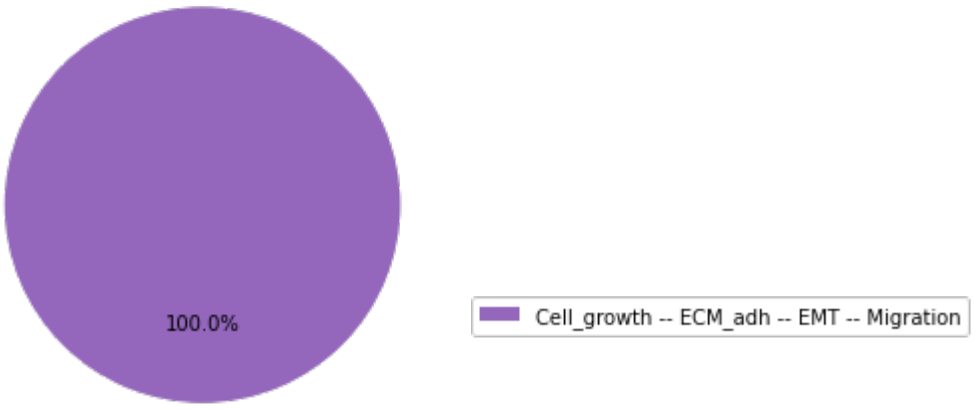

(('TGFbetaR', 'ON'), ('YAP1', 'OFF'))

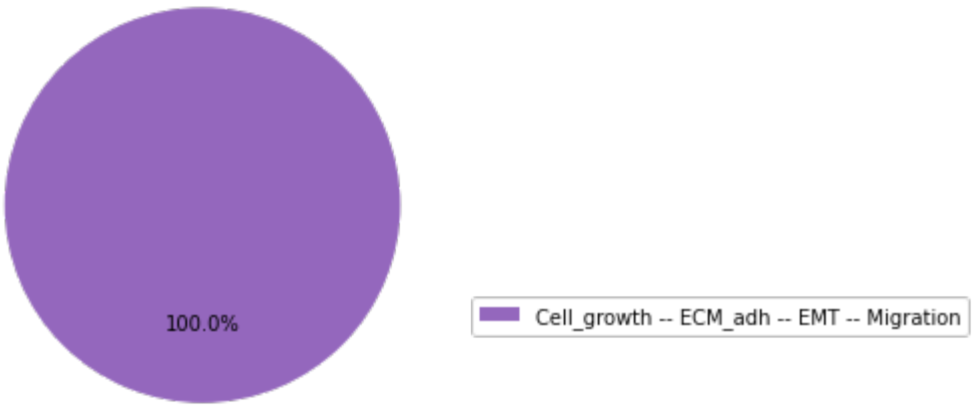

(('TGFbetaR', 'ON'), ('PIK3CA', 'OFF'))

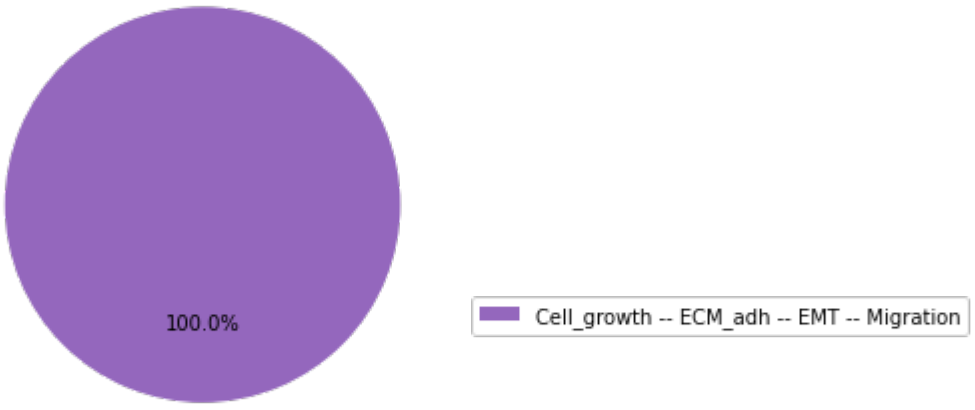

(('TGFbetaR', 'ON'), ('ERK', 'OFF'))

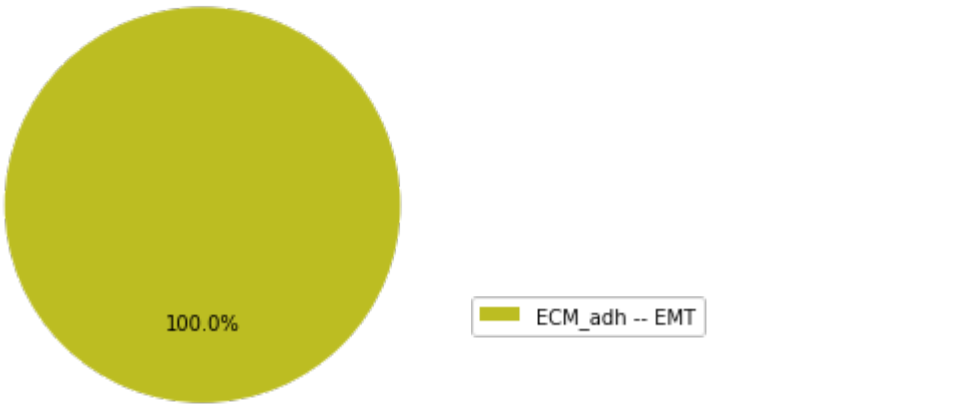

(('TGFbetaR', 'ON'), ('p21', 'OFF'))

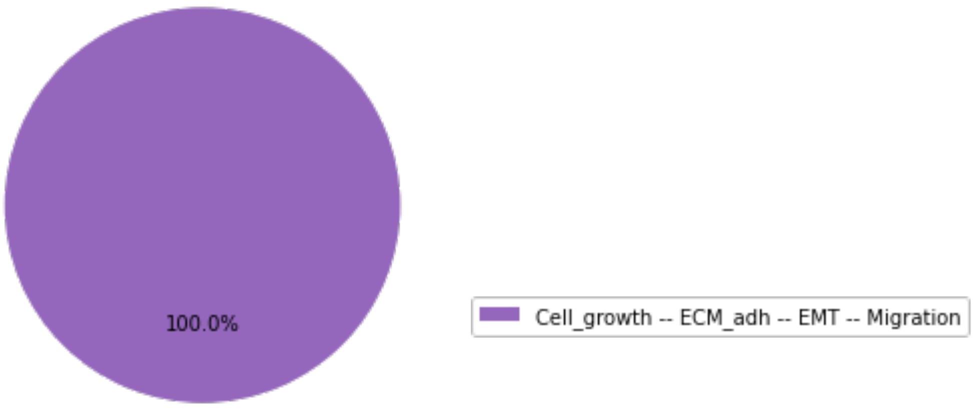

(('TGFbetaR', 'ON'), ('SMAD', 'OFF'))

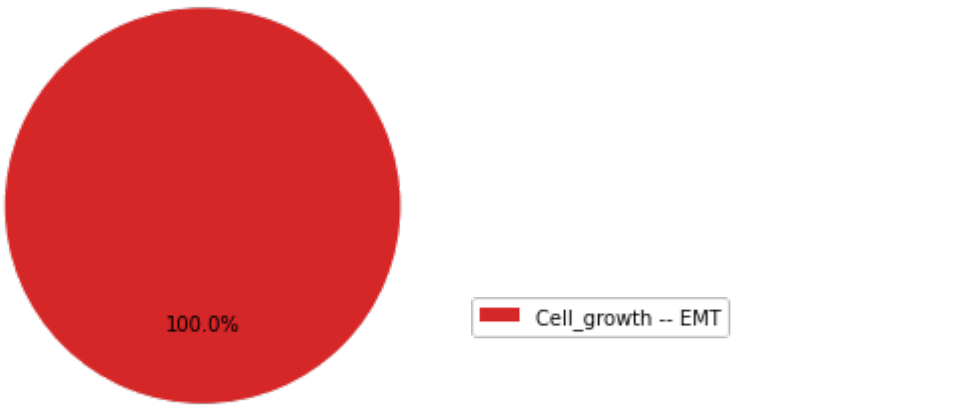

(('TGFbetaR', 'ON'), ('VIM', 'OFF'))

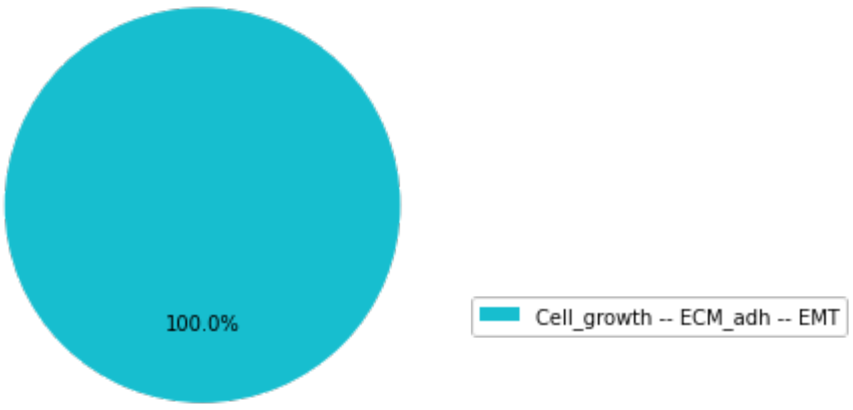

(('TGFbetaR', 'ON'), ('p53', 'OFF'))

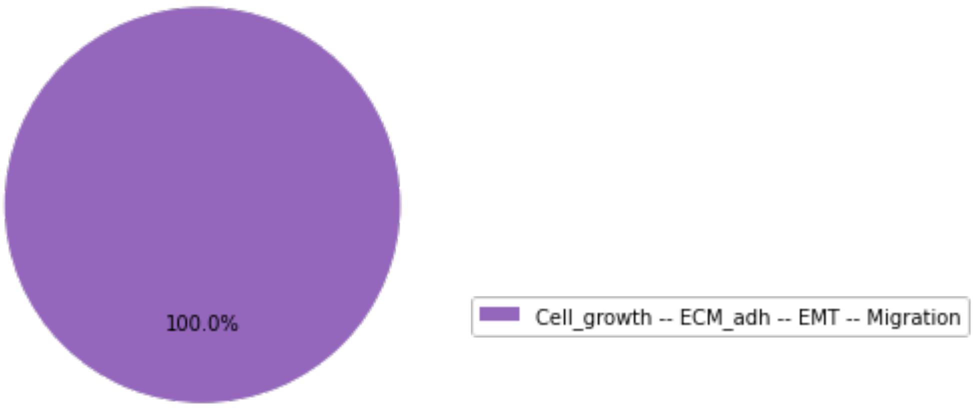

(('TGFbetaR', 'ON'), ('p63', 'OFF'))

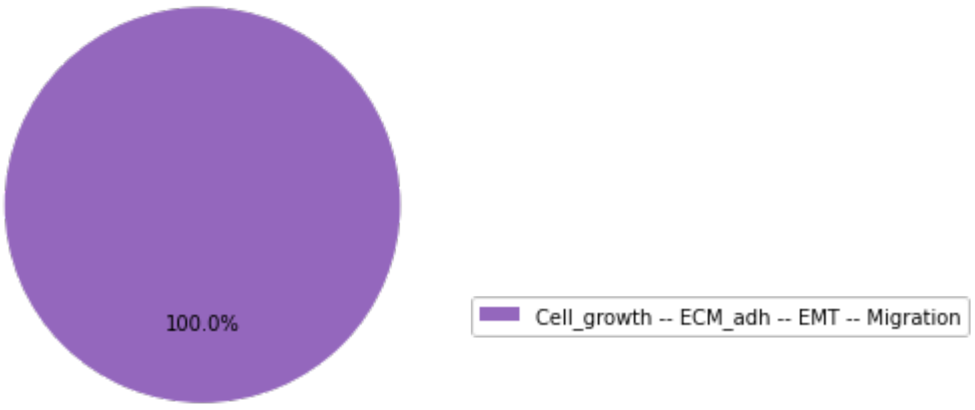

(('TGFbetaR', 'ON'), ('p73', 'OFF'))

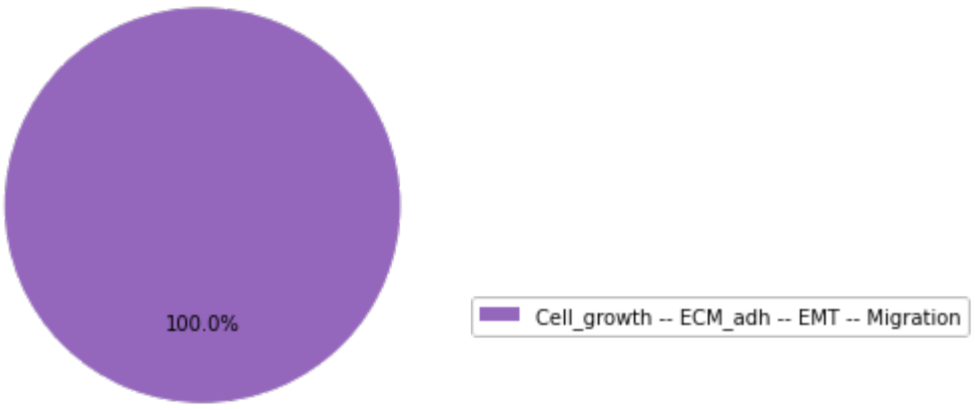

(('TGFbetaR', 'ON'), ('miR203', 'OFF'))

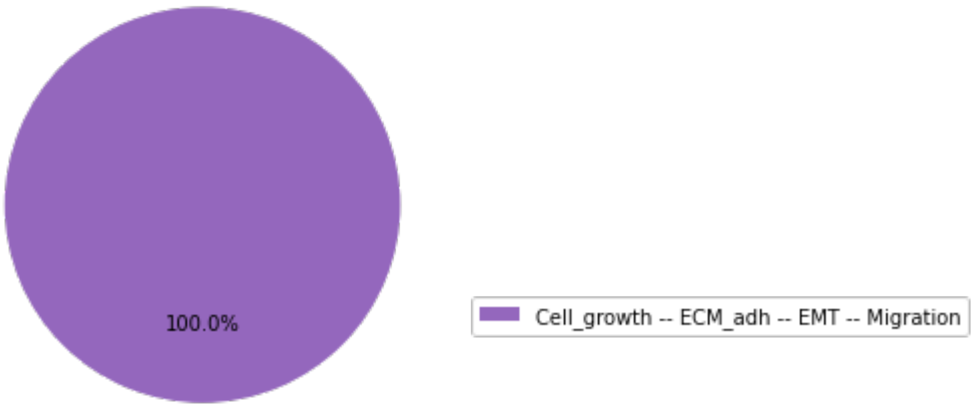

(('TGFbetaR', 'ON'), ('miR34', 'OFF'))

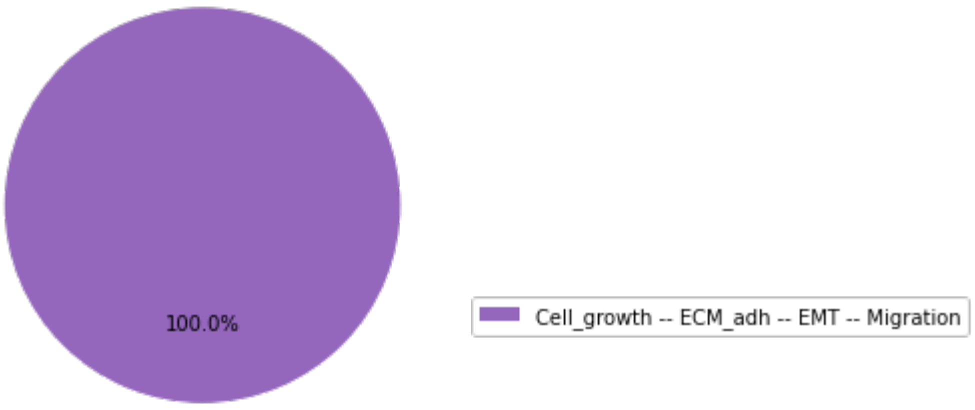

(('TGFbetaR', 'ON'), ('miR200', 'OFF'))

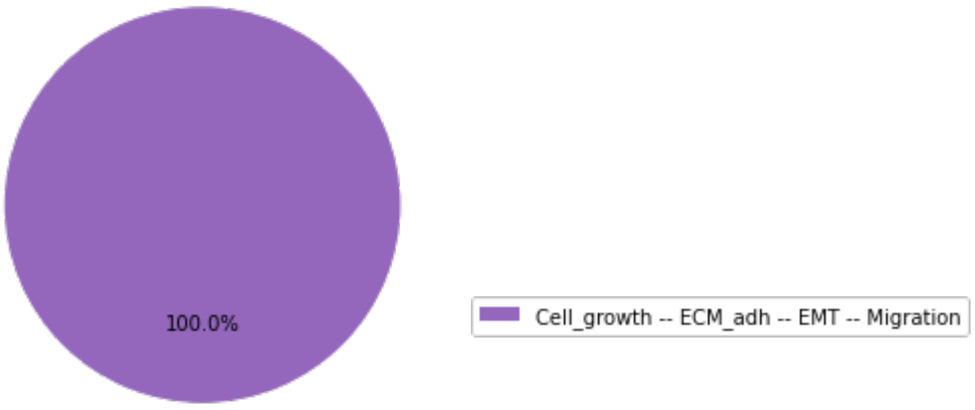

(('TGFbetaR', 'ON'), ('FAK', 'OFF'))

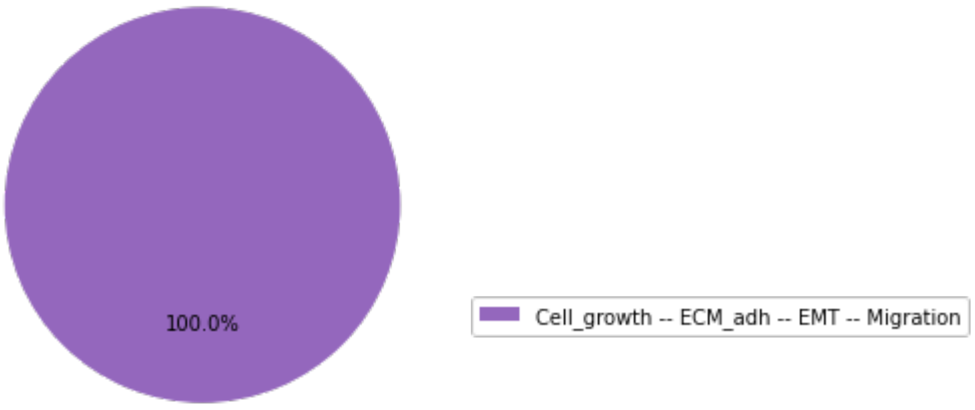

(('FAK', 'ON'), ('AKT1', 'OFF'))

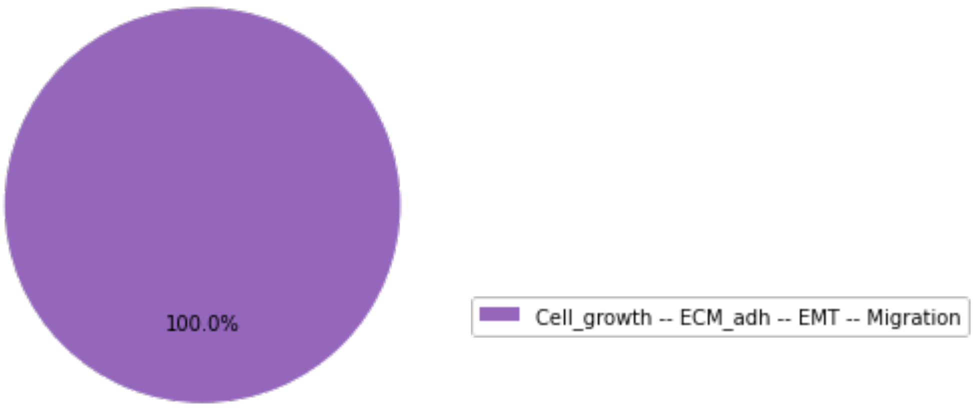

(('FAK', 'ON'), ('CTNNB1', 'OFF'))

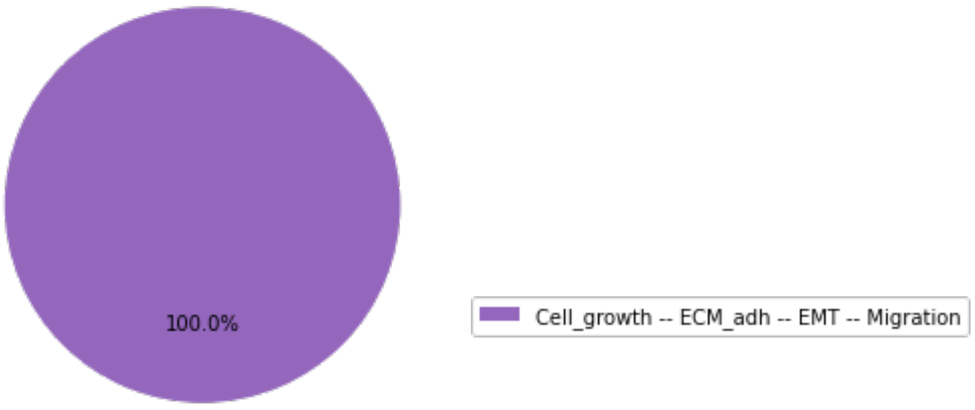

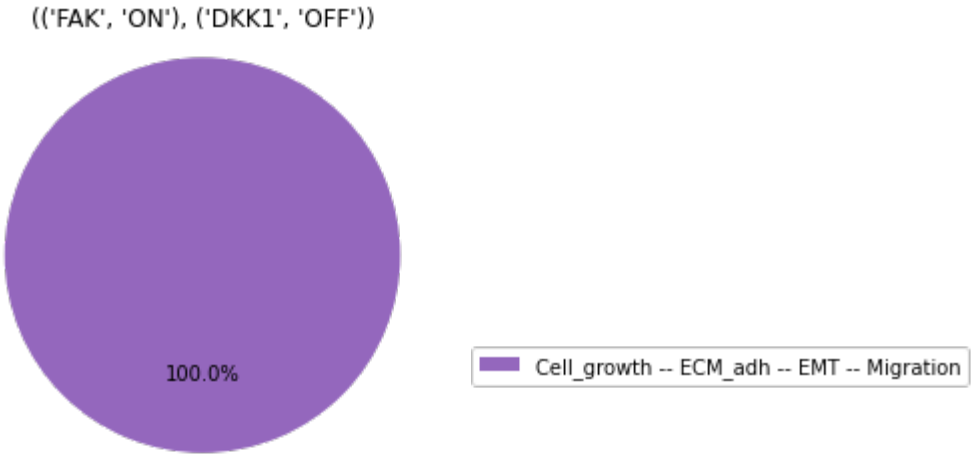
(('FAK', 'ON'), ('DKK1', 'OFF'))
100.0%
Cell_growth -- ECM_adh -- EMT -- Migration

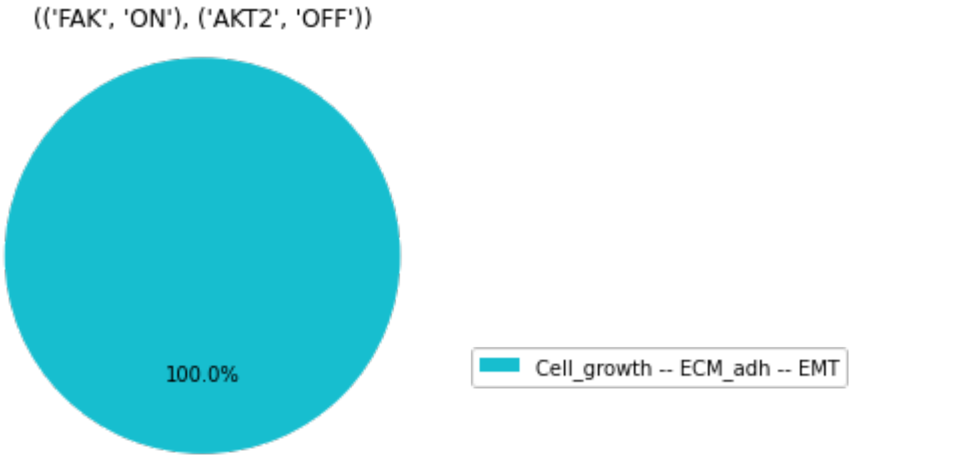
(('FAK', 'ON'), ('AKT2', 'OFF'))
100.0%
Cell_growth -- ECM_adh -- EMT

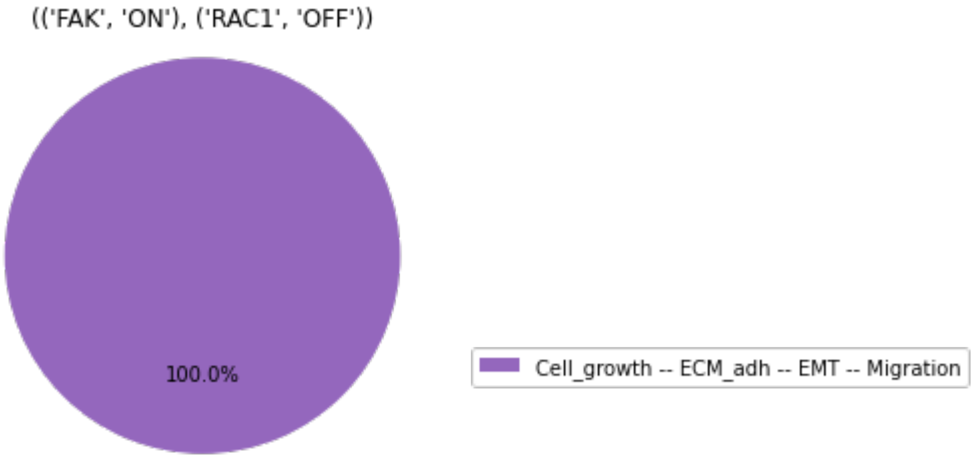
(('FAK', 'ON'), ('RAC1', 'OFF'))
100.0%
Cell_growth -- ECM_adh -- EMT -- Migration

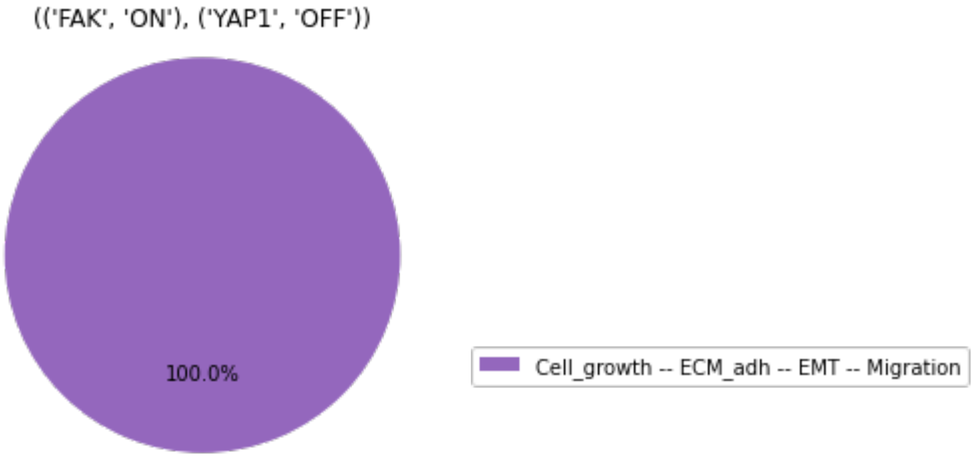
(('FAK', 'ON'), ('YAP1', 'OFF'))
100.0%
Cell_growth -- ECM_adh -- EMT -- Migration

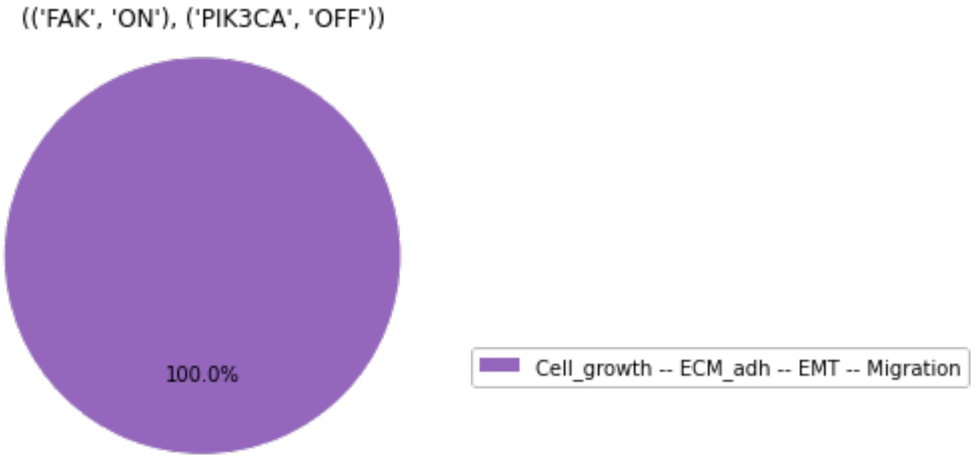
(('FAK', 'ON'), ('PIK3CA', 'OFF'))
100.0%
Cell_growth -- ECM_adh -- EMT -- Migration

(('FAK', 'ON'), ('ERK', 'OFF'))

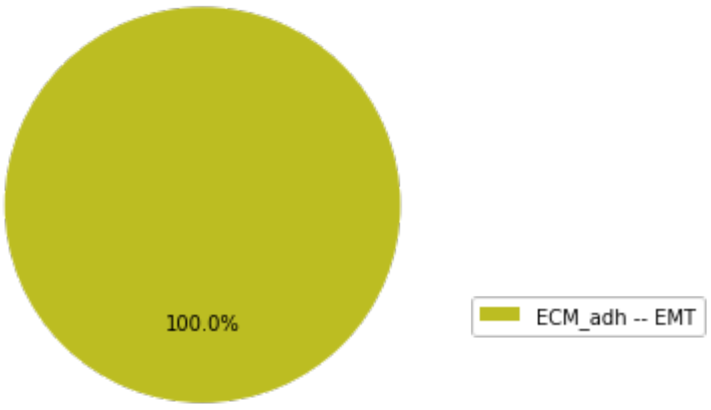

(('FAK', 'ON'), ('p21', 'OFF'))

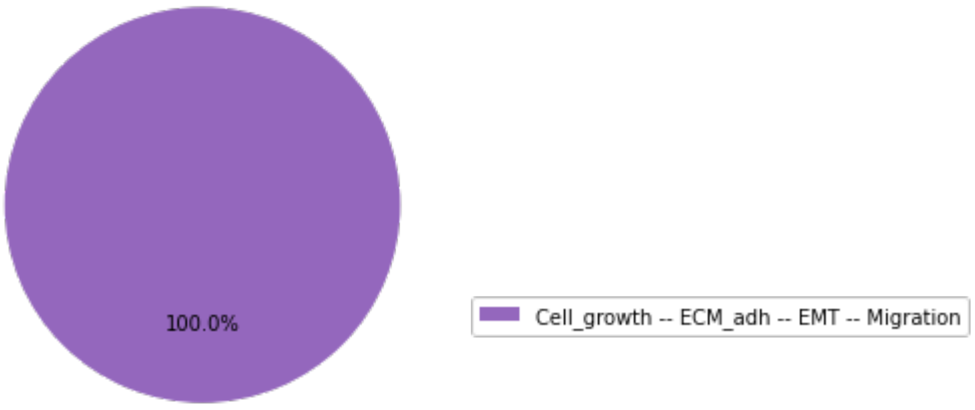

(('FAK', 'ON'), ('SMAD', 'OFF'))

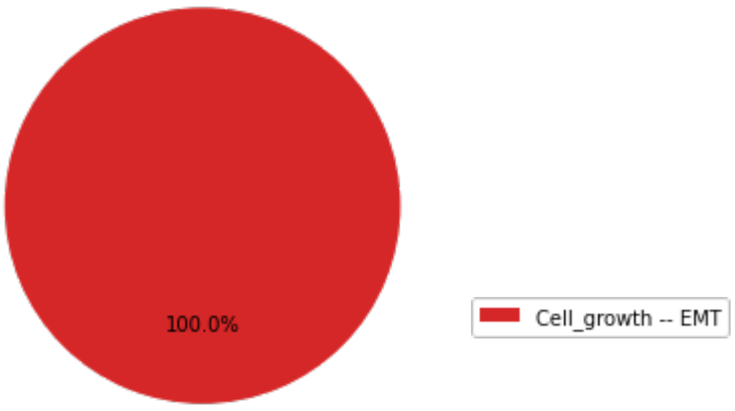

(('FAK', 'ON'), ('VIM', 'OFF'))

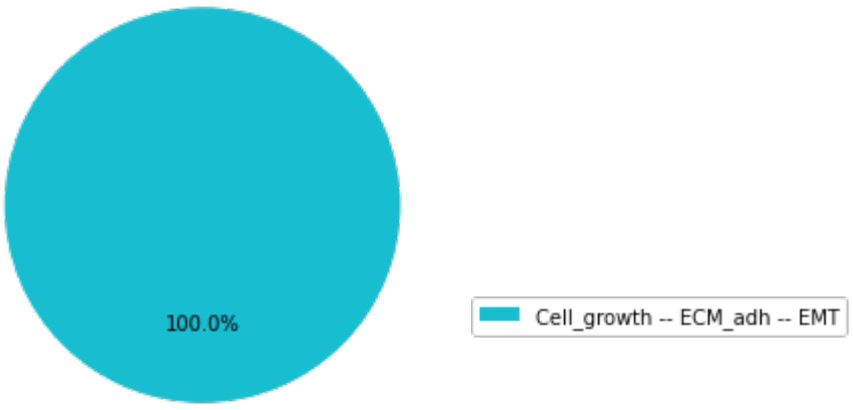

(('FAK', 'ON'), ('p53', 'OFF'))

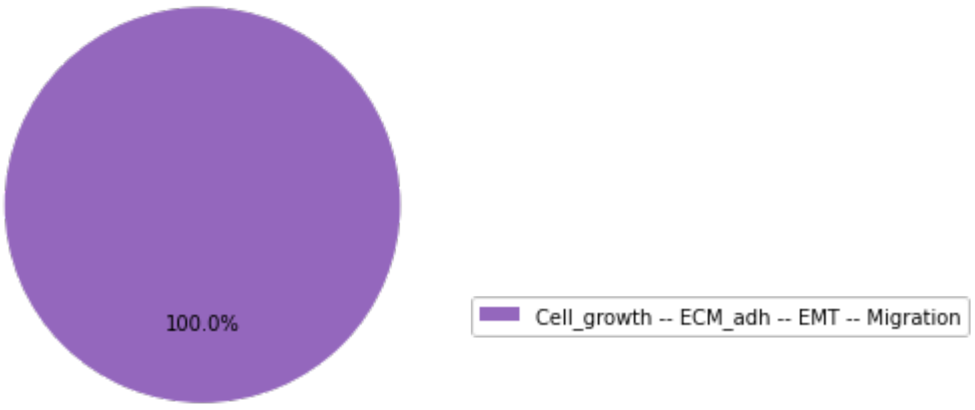

(('FAK', 'ON'), ('p63', 'OFF'))

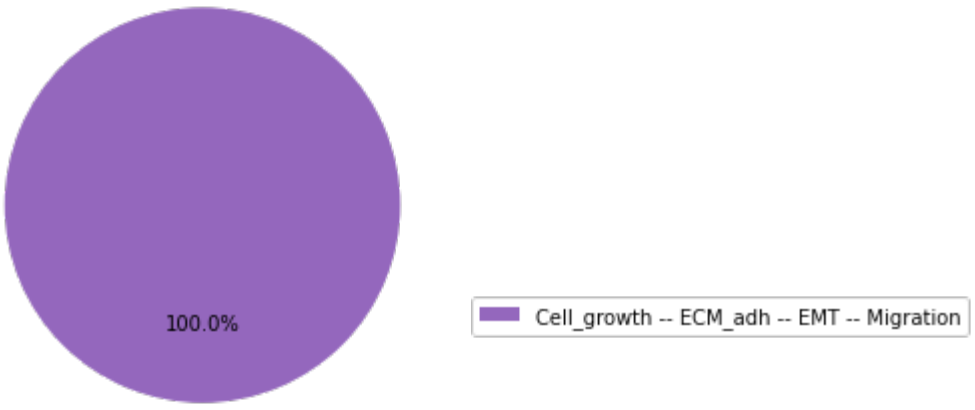

(('FAK', 'ON'), ('p73', 'OFF'))

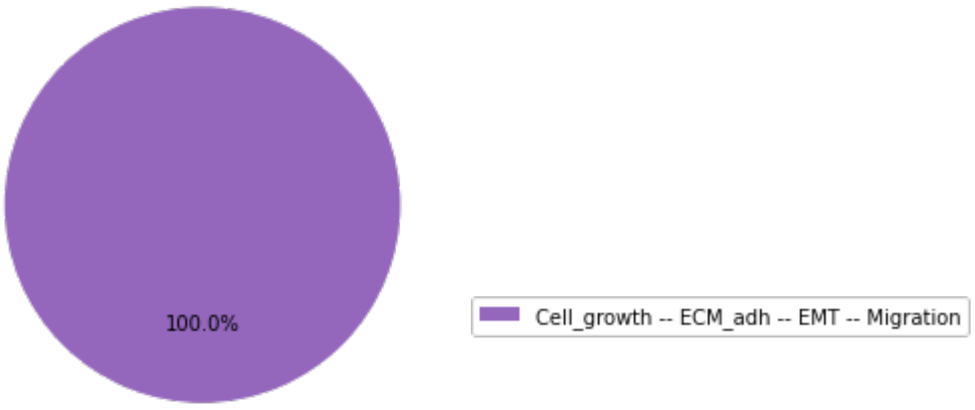

(('FAK', 'ON'), ('miR203', 'OFF'))

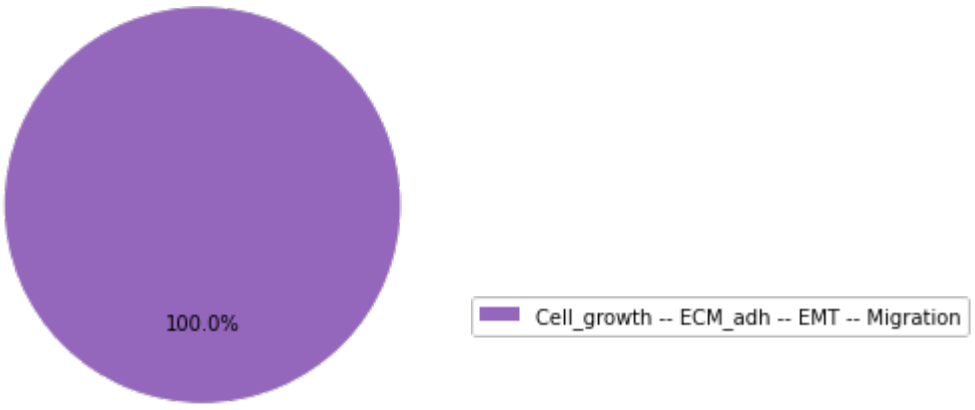

(('FAK', 'ON'), ('miR34', 'OFF'))

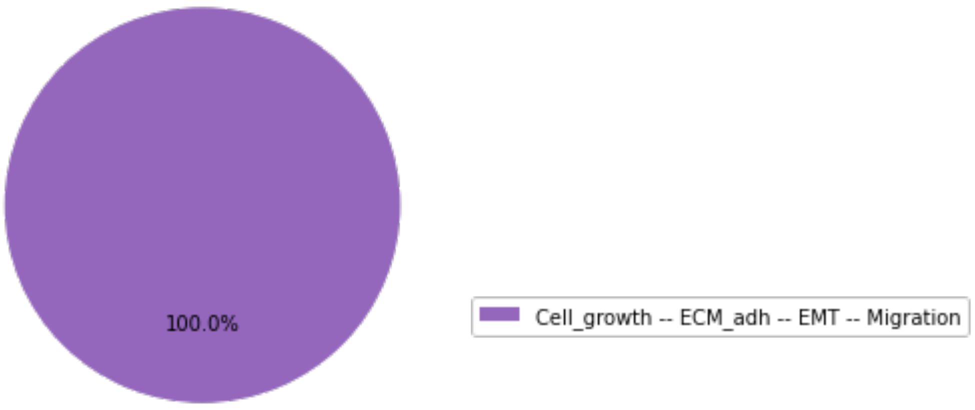

(('FAK', 'ON'), ('miR200', 'OFF'))

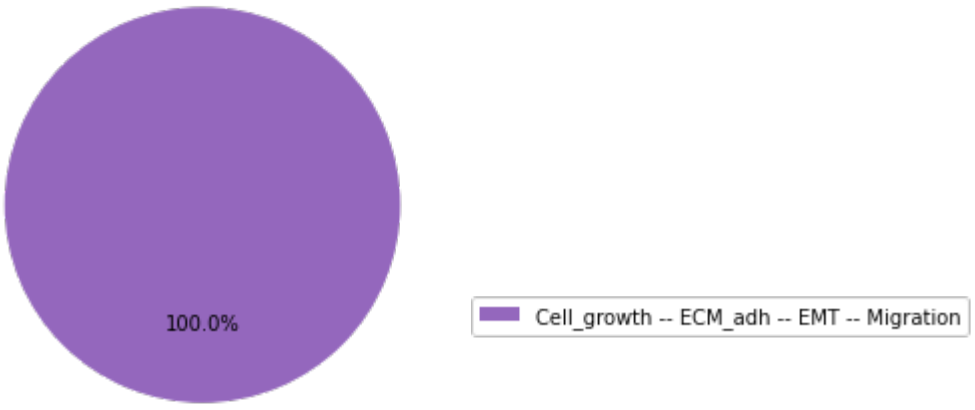

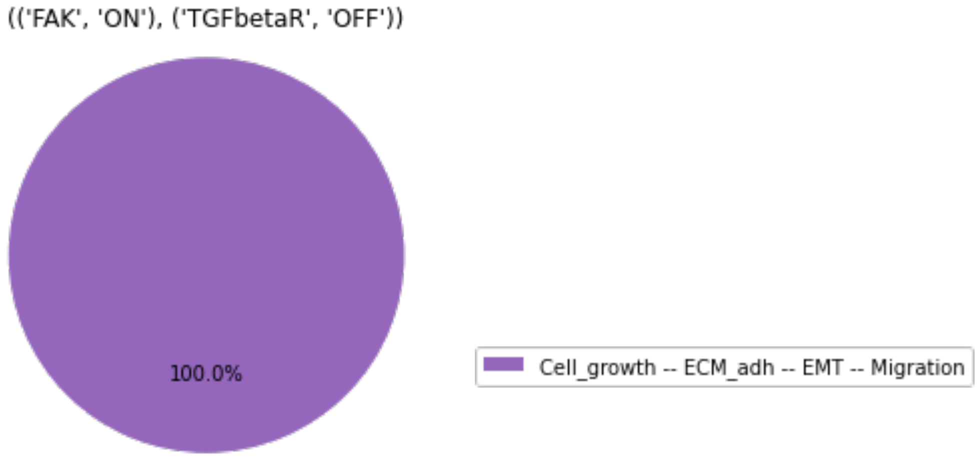
(('FAK', 'ON'), ('TGFbetaR', 'OFF'))
100.0%
Cell_growth -- ECM_adh -- EMT -- Migration

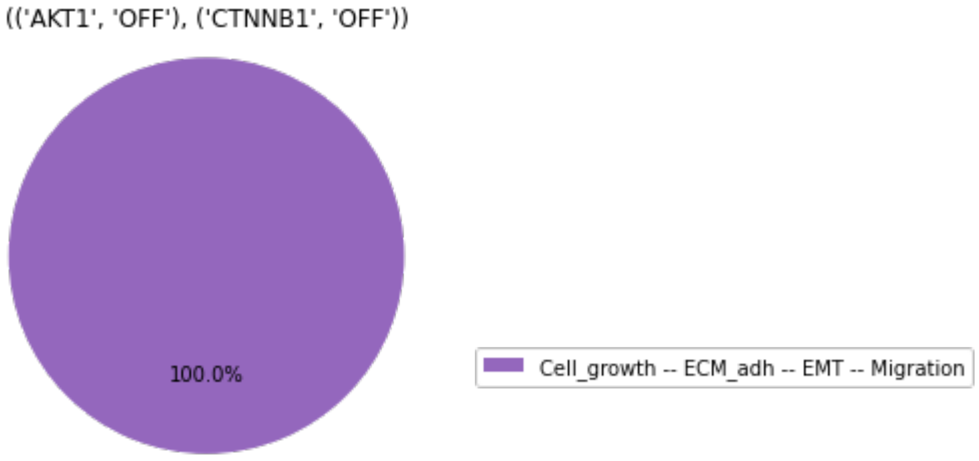
(('AKT1', 'OFF'), ('CTNNB1', 'OFF'))
100.0%
Cell_growth -- ECM_adh -- EMT -- Migration

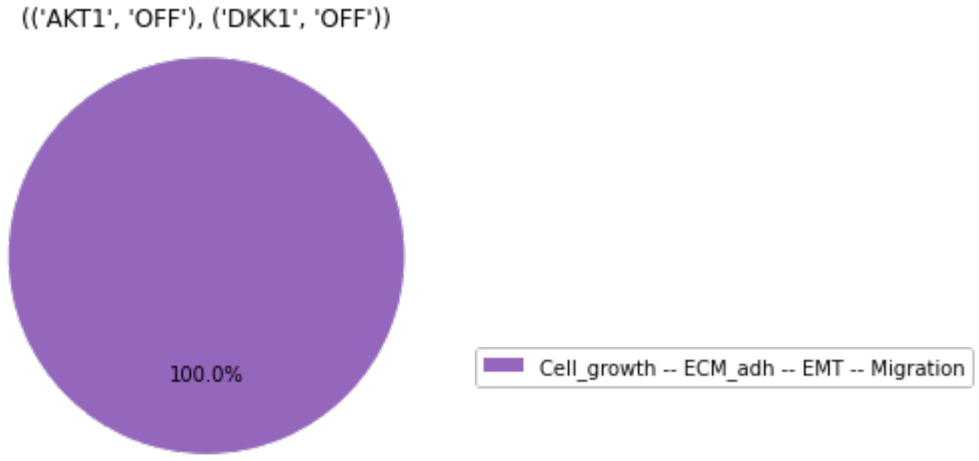
(('AKT1', 'OFF'), ('DKK1', 'OFF'))
100.0%
Cell_growth -- ECM_adh -- EMT -- Migration

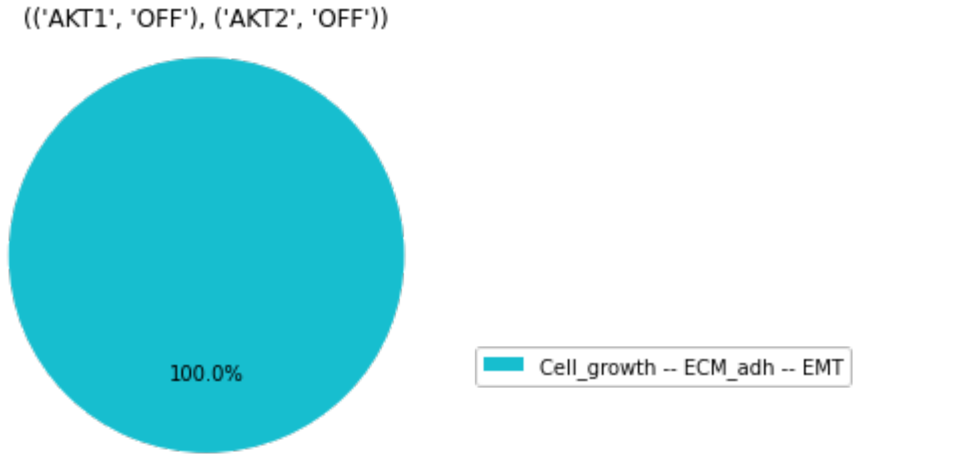
(('AKT1', 'OFF'), ('AKT2', 'OFF'))
100.0%
Cell_growth -- ECM_adh -- EMT

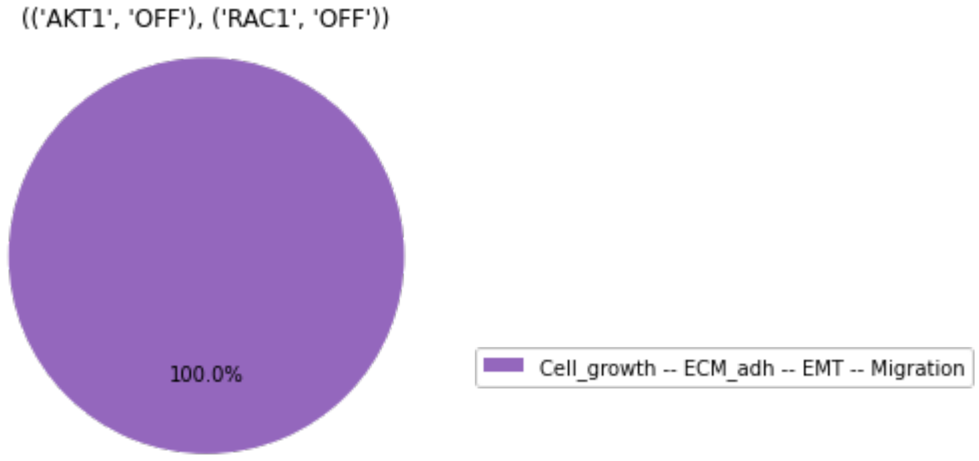
(('AKT1', 'OFF'), ('RAC1', 'OFF'))
100.0%
Cell_growth -- ECM_adh -- EMT -- Migration

(('AKT1', 'OFF'), ('YAP1', 'OFF'))

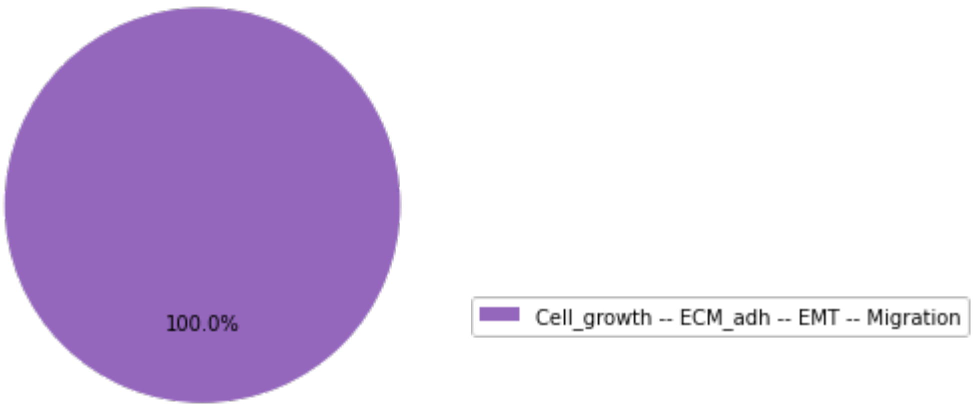

(('AKT1', 'OFF'), ('PIK3CA', 'OFF'))

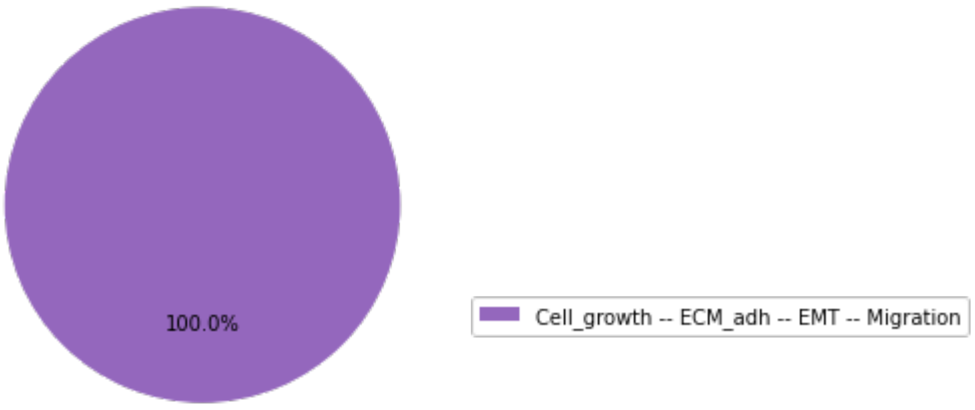

(('AKT1', 'OFF'), ('ERK', 'OFF'))

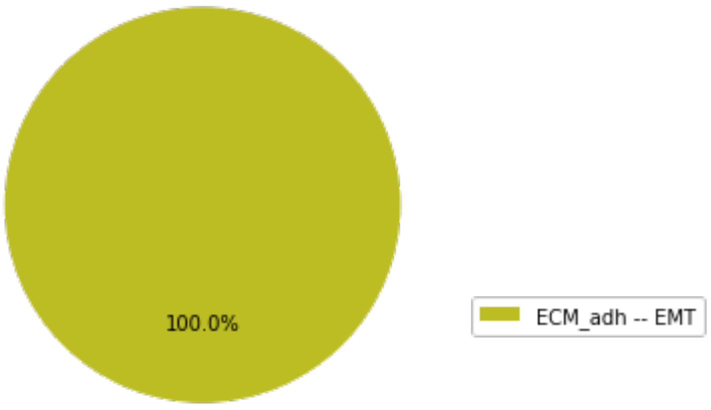

(('AKT1', 'OFF'), ('p21', 'OFF'))

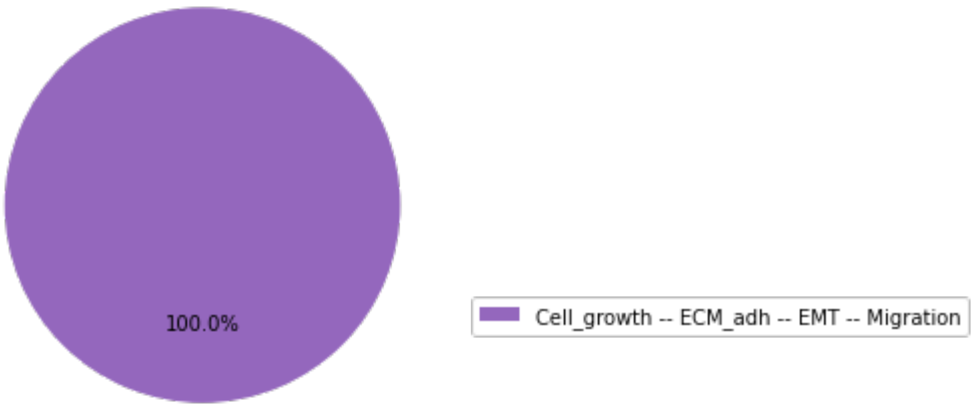

(('AKT1', 'OFF'), ('SMAD', 'OFF'))

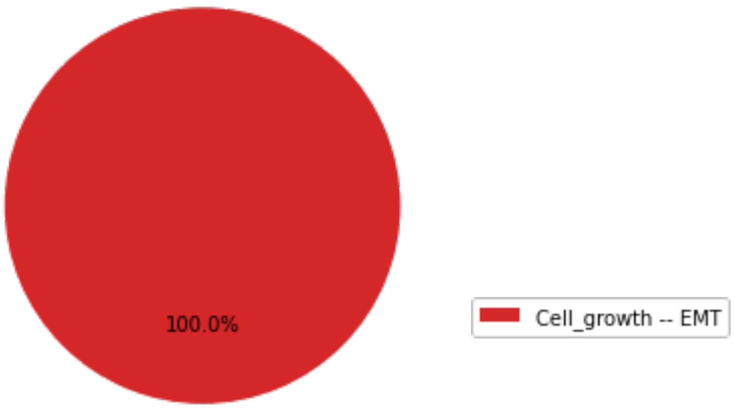

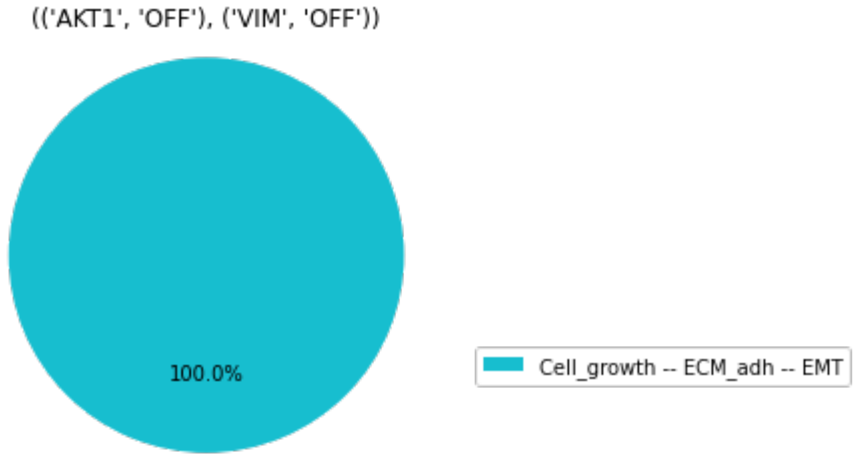
(('AKT1', 'OFF'), ('VIM', 'OFF'))
100.0%
Cell_growth -- ECM_adh -- EMT

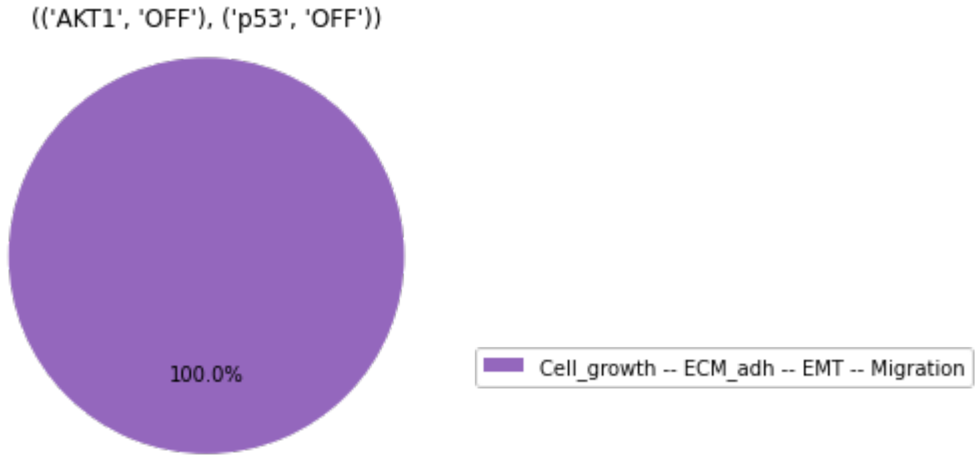
(('AKT1', 'OFF'), ('p53', 'OFF'))
100.0%
Cell_growth -- ECM_adh -- EMT -- Migration

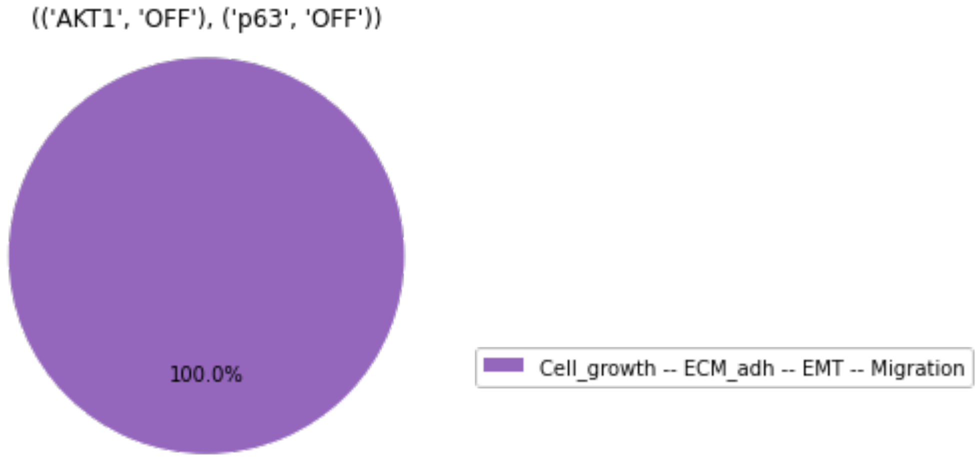
(('AKT1', 'OFF'), ('p63', 'OFF'))
100.0%
Cell_growth -- ECM_adh -- EMT -- Migration

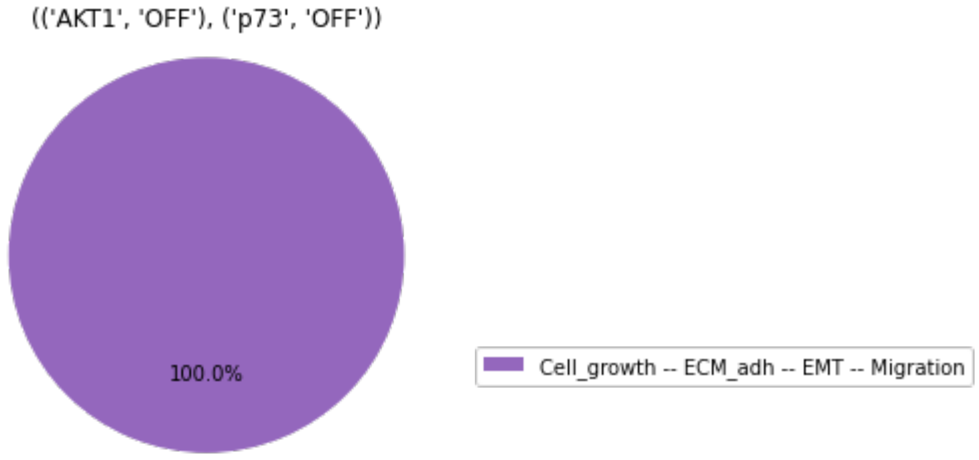
(('AKT1', 'OFF'), ('p73', 'OFF'))
100.0%
Cell_growth -- ECM_adh -- EMT -- Migration

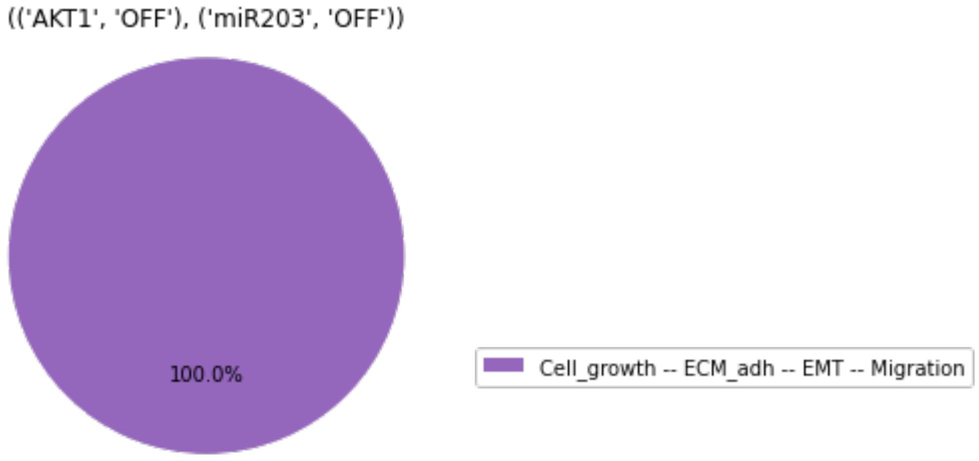
(('AKT1', 'OFF'), ('miR203', 'OFF'))
100.0%
Cell_growth -- ECM_adh -- EMT -- Migration

(('AKT1', 'OFF'), ('miR34', 'OFF'))

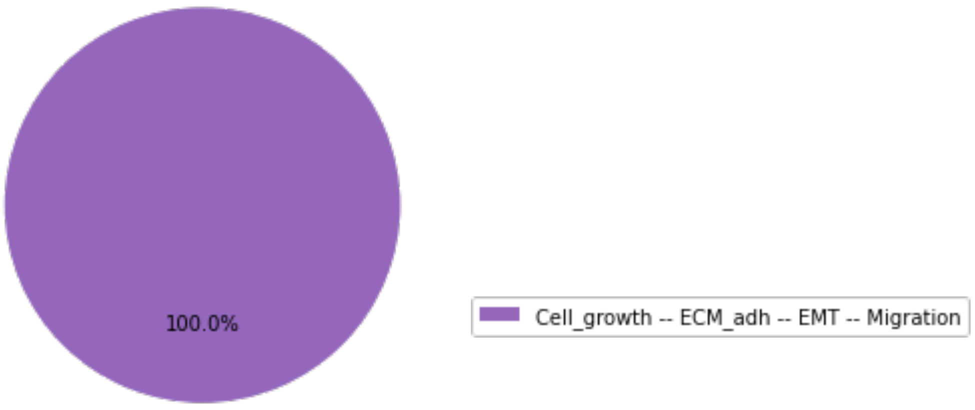

(('AKT1', 'OFF'), ('miR200', 'OFF'))

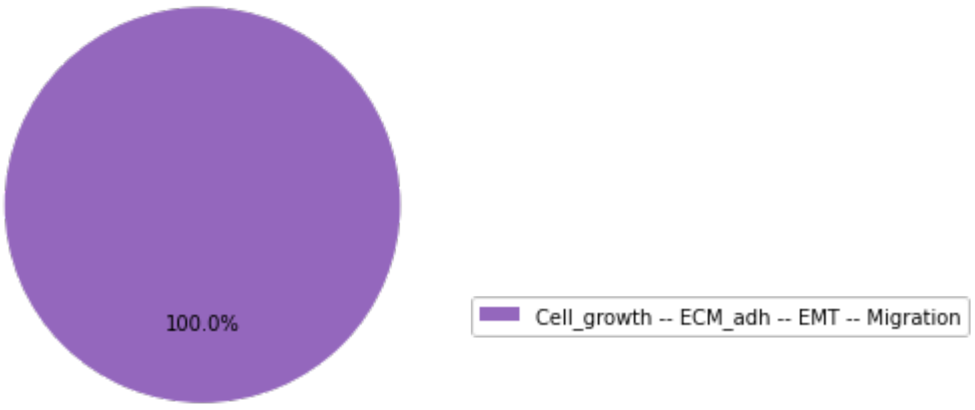

(('AKT1', 'OFF'), ('TGFbetaR', 'OFF'))

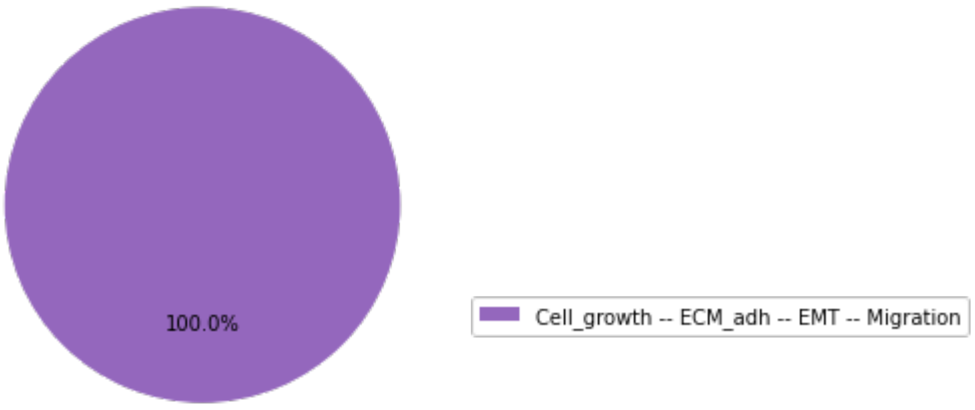

(('AKT1', 'OFF'), ('FAK', 'OFF'))

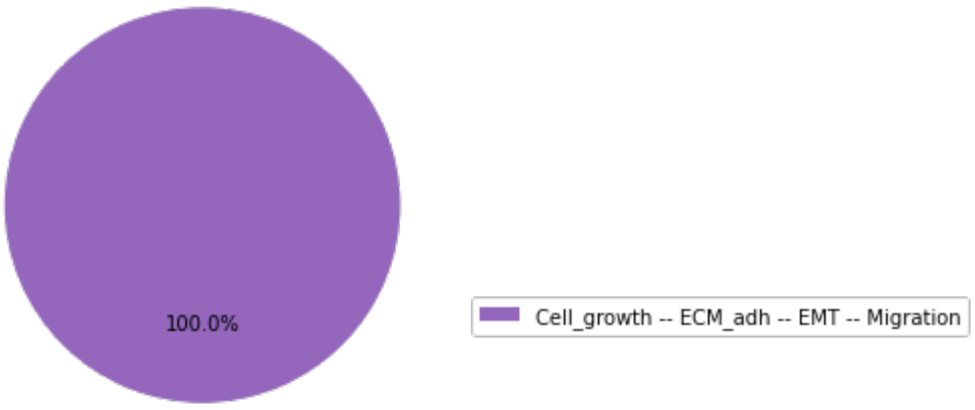

(('CTNNB1', 'OFF'), ('DKK1', 'OFF'))

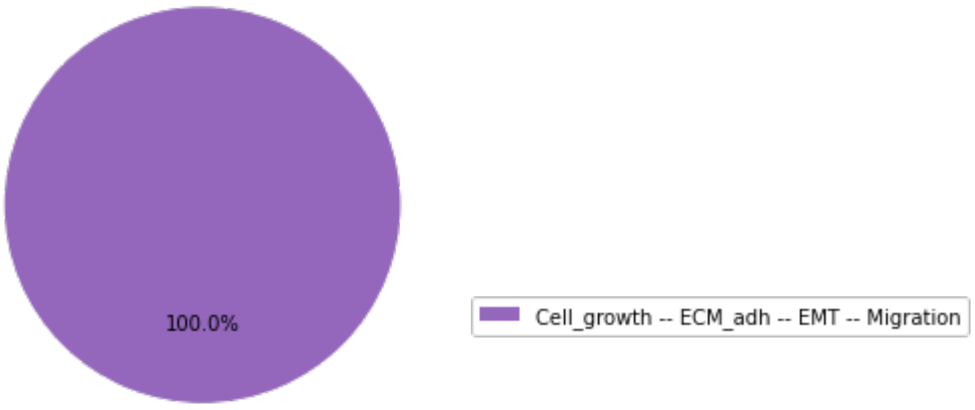

(('CTNNB1', 'OFF'), ('AKT2', 'OFF'))

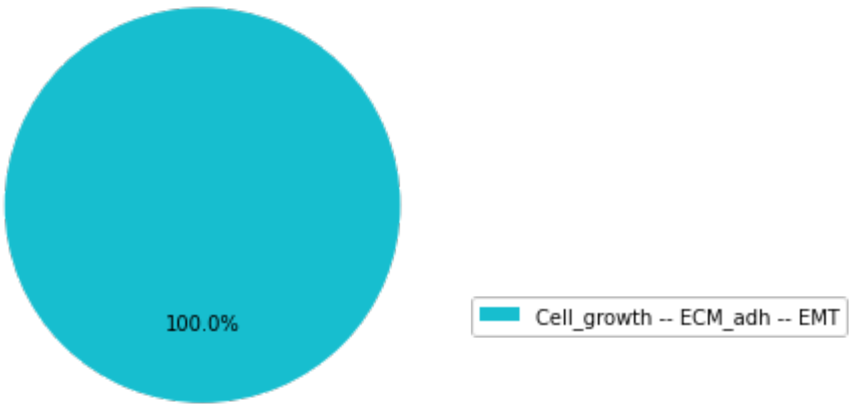

(('CTNNB1', 'OFF'), ('RAC1', 'OFF'))

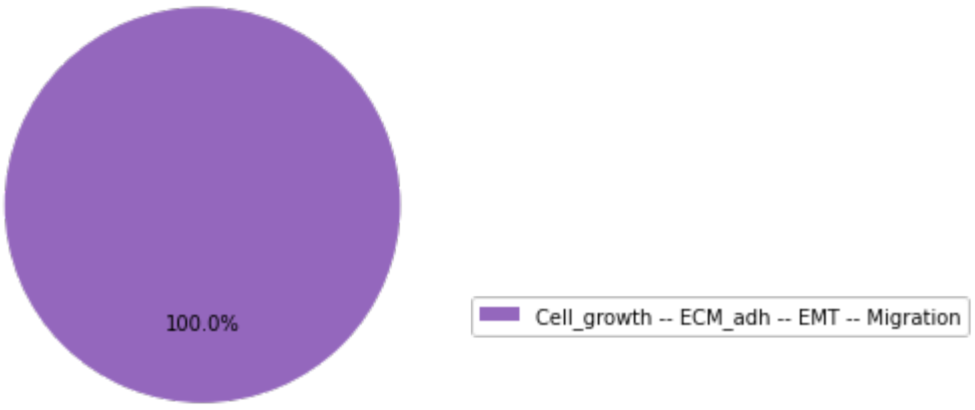

(('CTNNB1', 'OFF'), ('YAP1', 'OFF'))

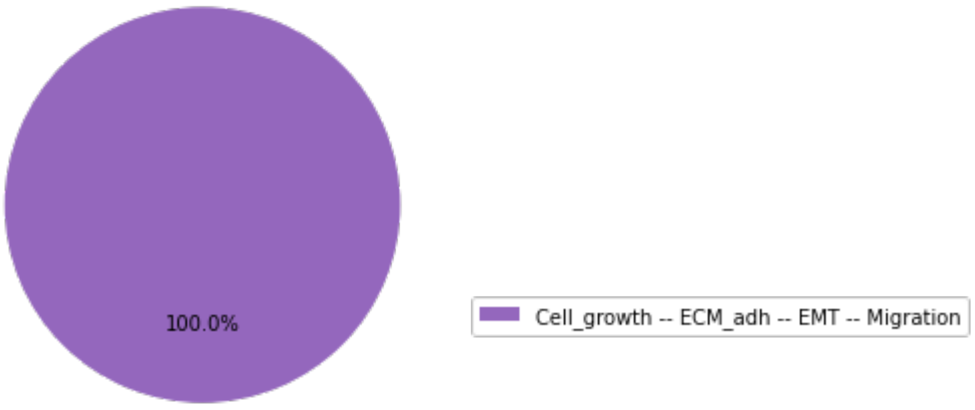

(('CTNNB1', 'OFF'), ('PIK3CA', 'OFF'))

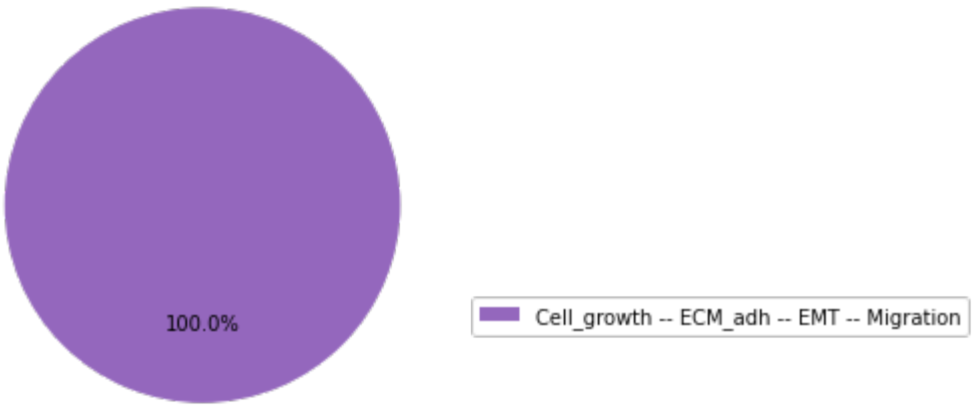

(('CTNNB1', 'OFF'), ('ERK', 'OFF'))

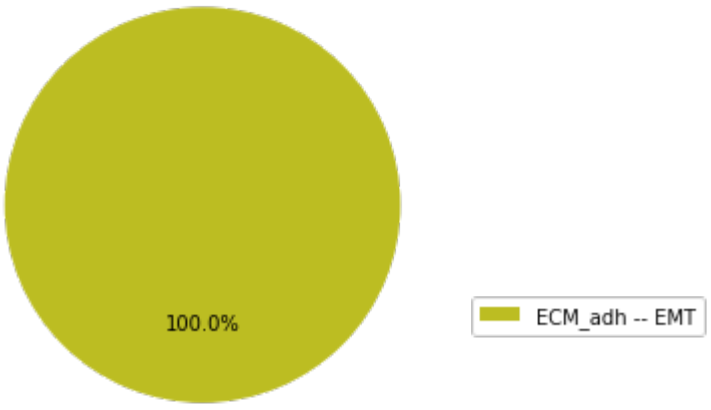

(('CTNNB1', 'OFF'), ('p21', 'OFF'))

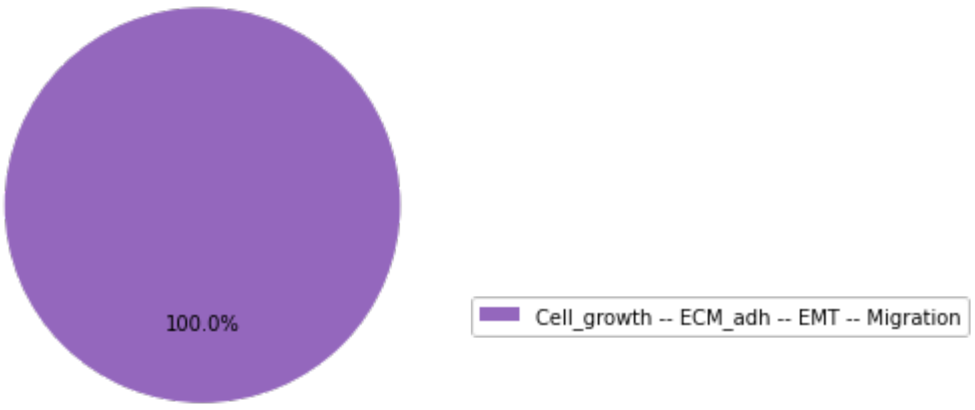

(('CTNNB1', 'OFF'), ('SMAD', 'OFF'))

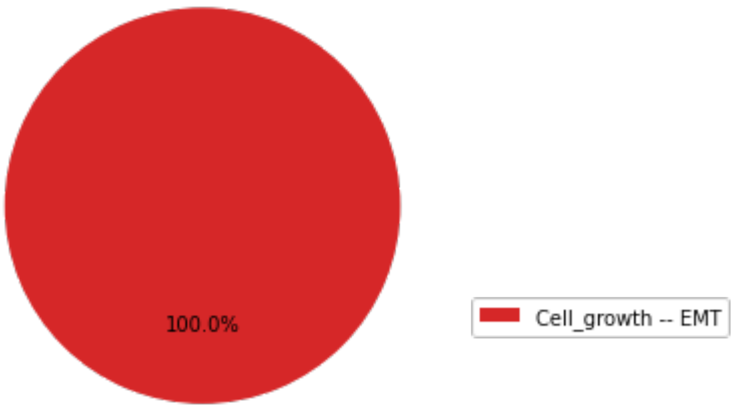

(('CTNNB1', 'OFF'), ('VIM', 'OFF'))

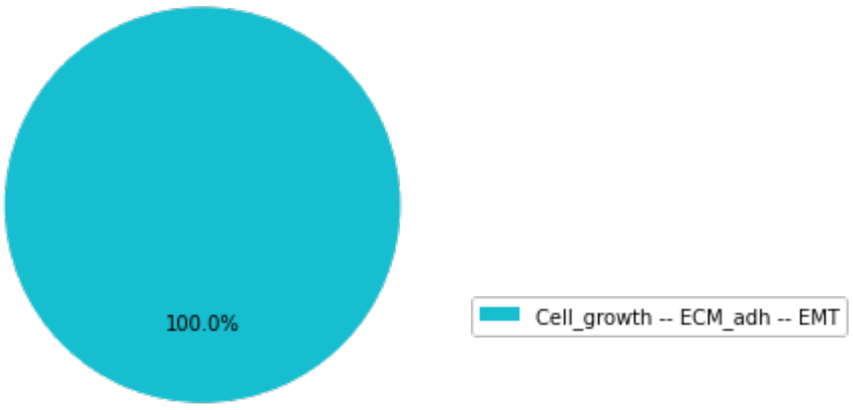

(('CTNNB1', 'OFF'), ('p53', 'OFF'))

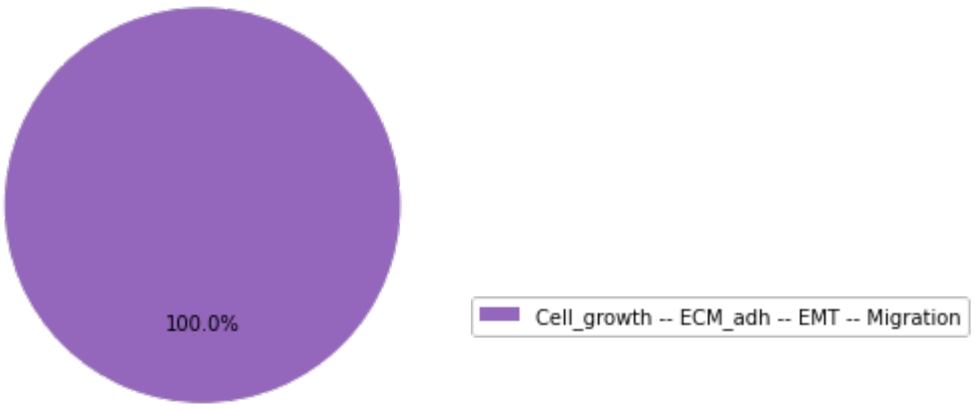

(('CTNNB1', 'OFF'), ('p63', 'OFF'))

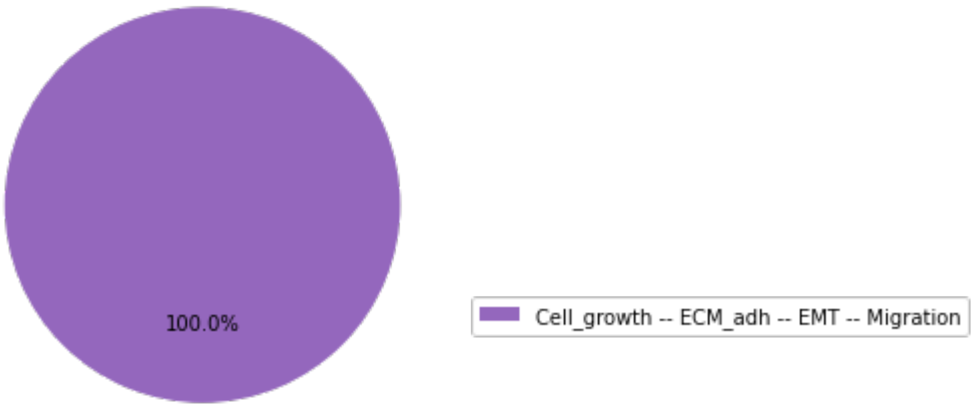

(('CTNNB1', 'OFF'), ('p73', 'OFF'))

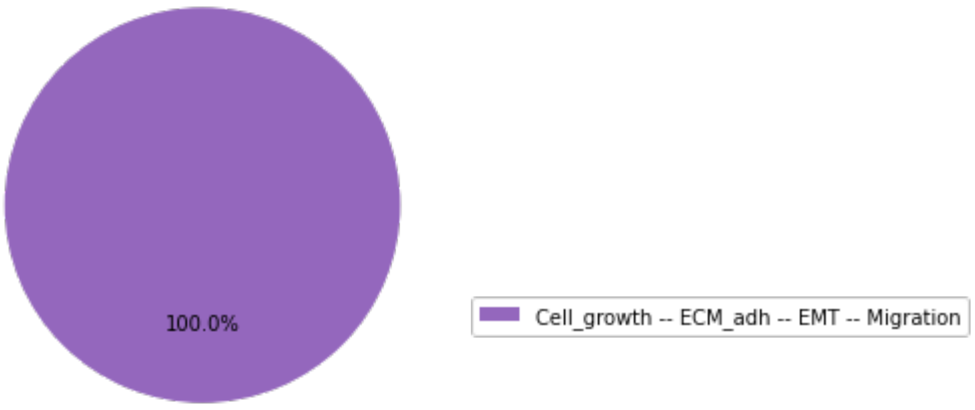

(('CTNNB1', 'OFF'), ('miR203', 'OFF'))

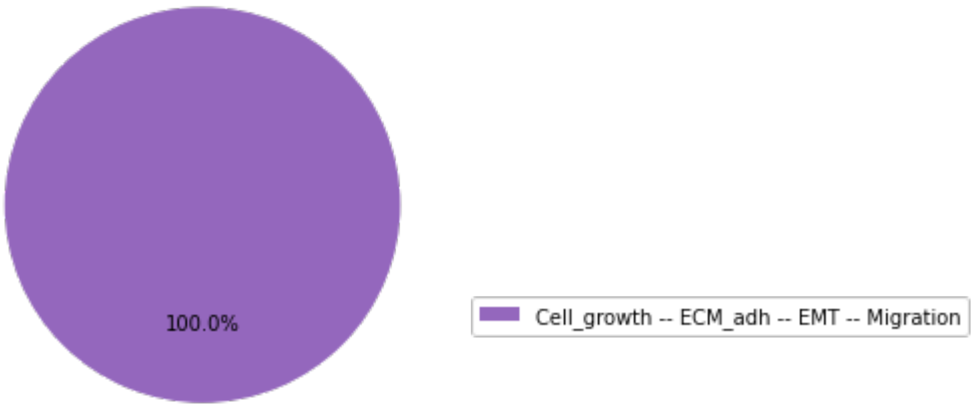

(('CTNNB1', 'OFF'), ('miR34', 'OFF'))

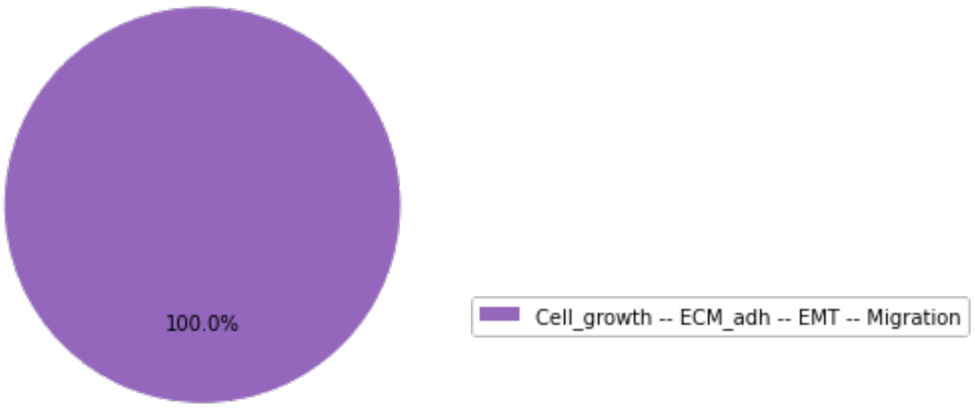

(('CTNNB1', 'OFF'), ('miR200', 'OFF'))

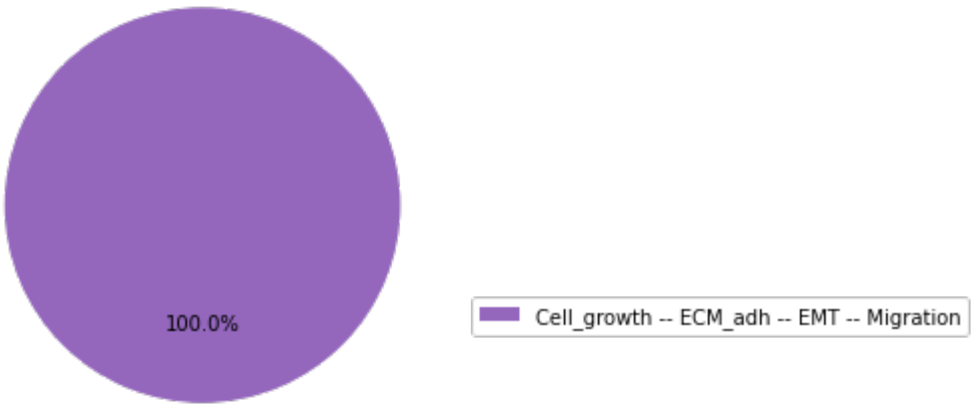

(('CTNNB1', 'OFF'), ('TGFbetaR', 'OFF'))

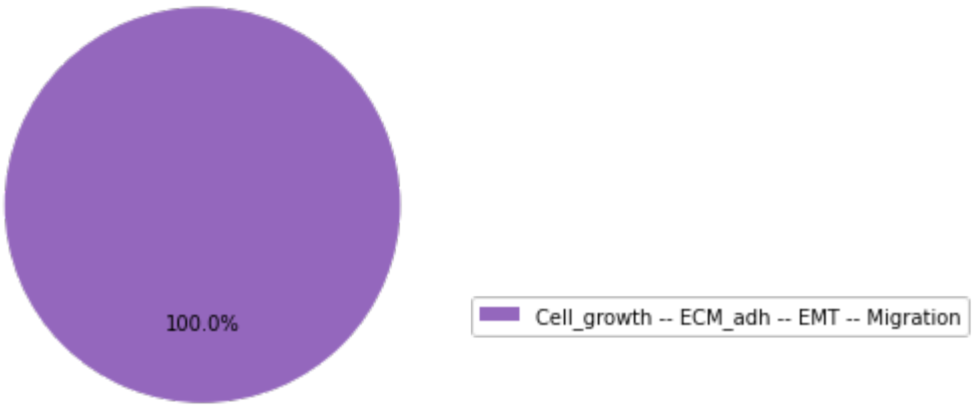

(('CTNNB1', 'OFF'), ('FAK', 'OFF'))

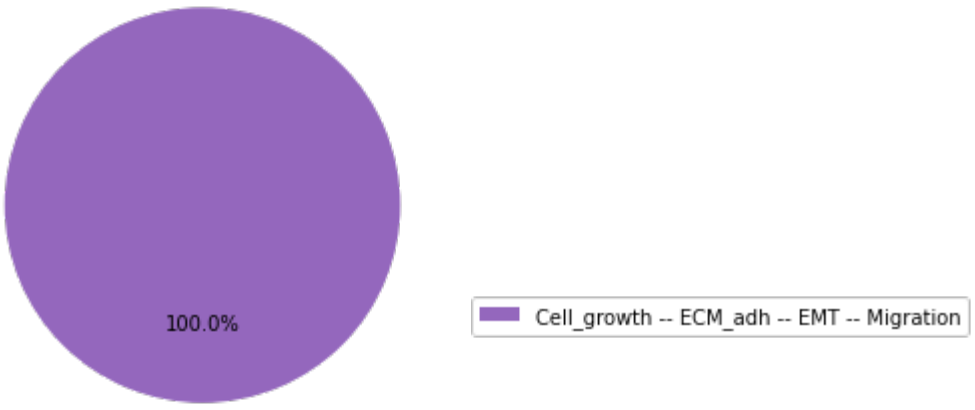

(('DKK1', 'OFF'), ('AKT2', 'OFF'))

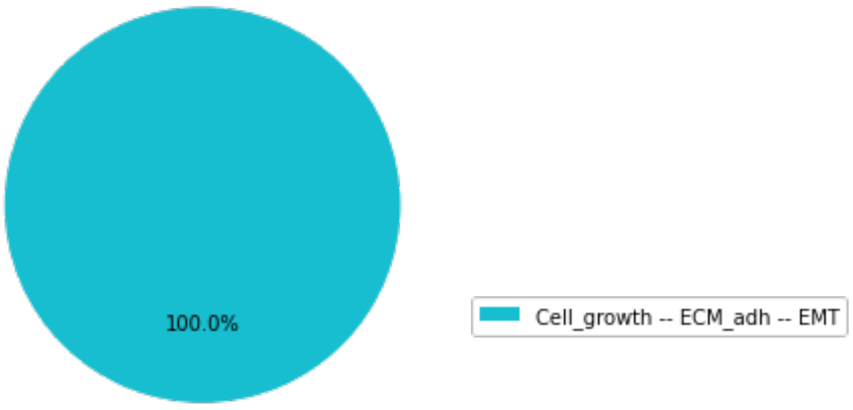

(('DKK1', 'OFF'), ('RAC1', 'OFF'))

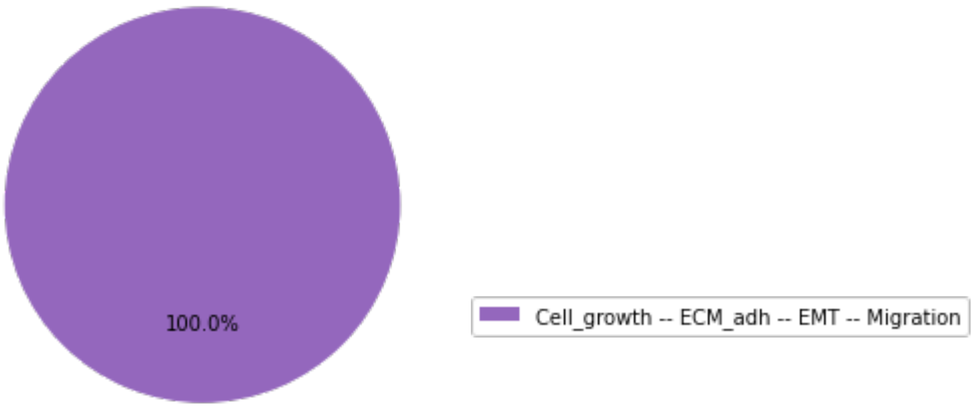

(('DKK1', 'OFF'), ('YAP1', 'OFF'))

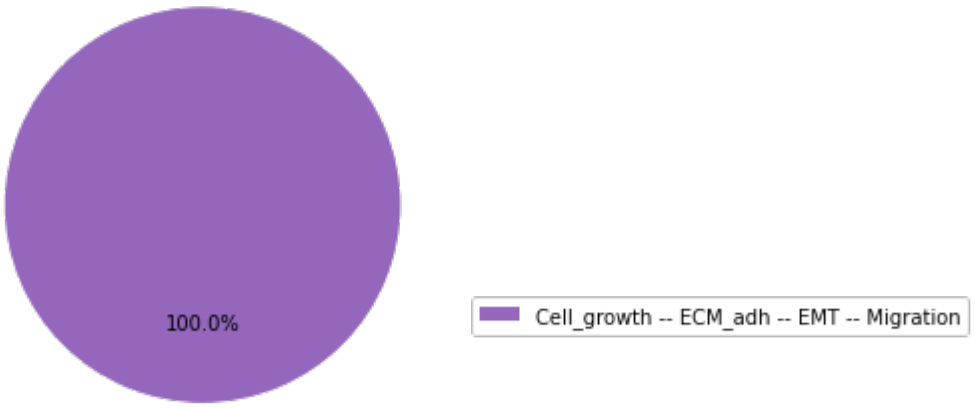

(('DKK1', 'OFF'), ('PIK3CA', 'OFF'))

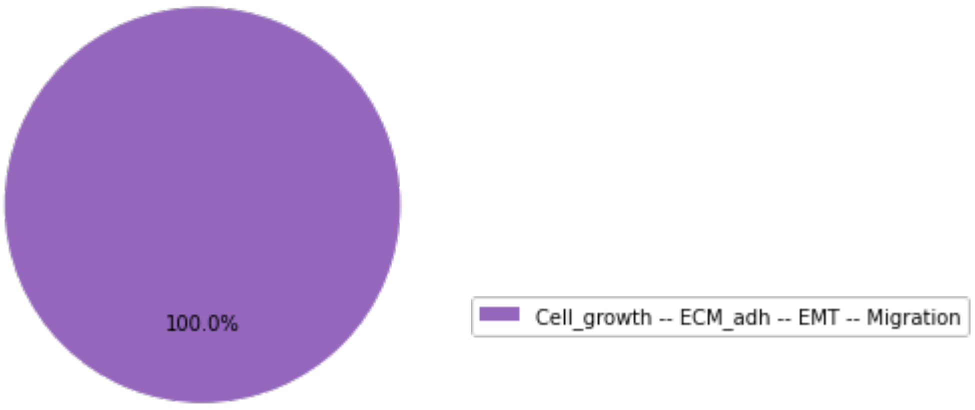

(('DKK1', 'OFF'), ('ERK', 'OFF'))

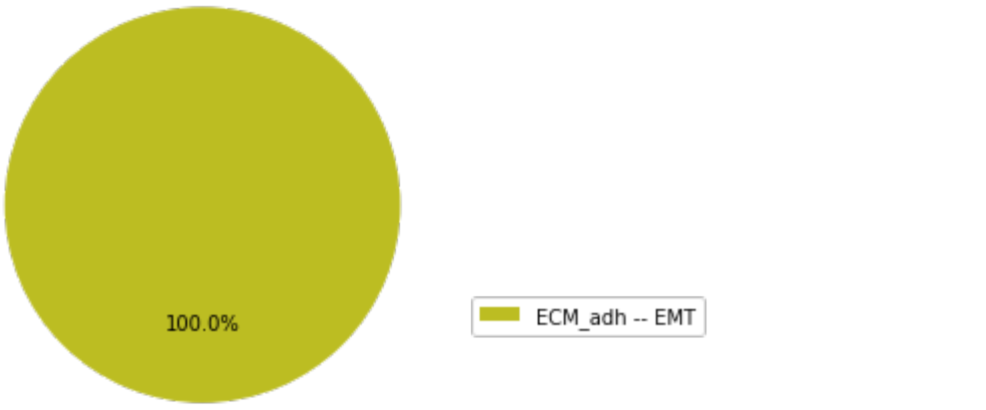

(('DKK1', 'OFF'), ('p21', 'OFF'))

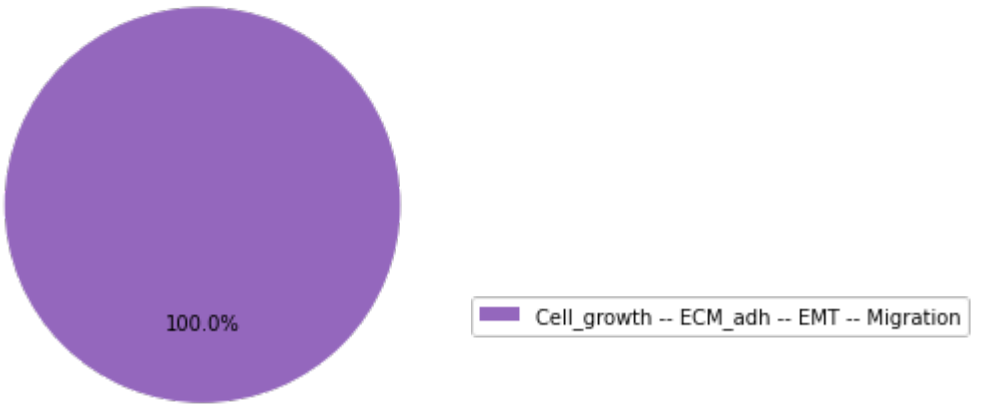

(('DKK1', 'OFF'), ('SMAD', 'OFF'))

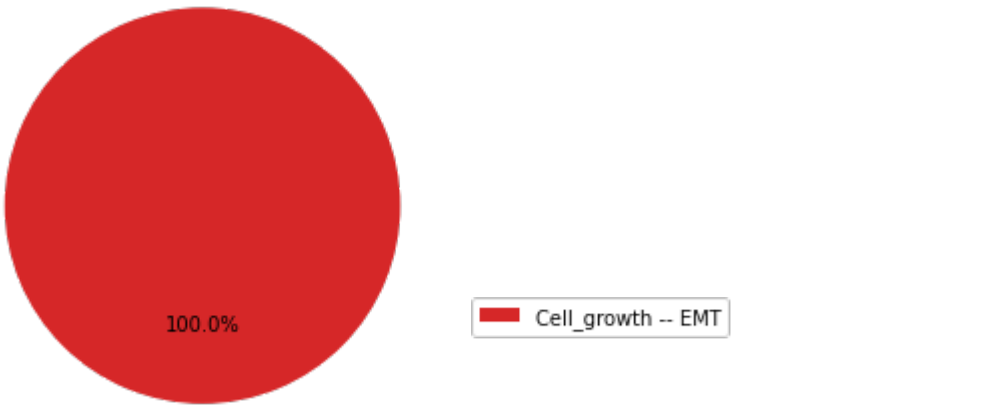

(('DKK1', 'OFF'), ('VIM', 'OFF'))

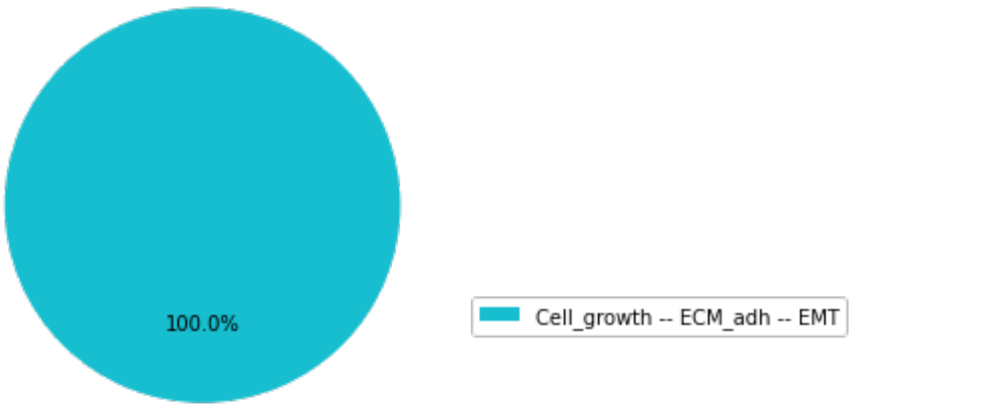

(('DKK1', 'OFF'), ('p53', 'OFF'))

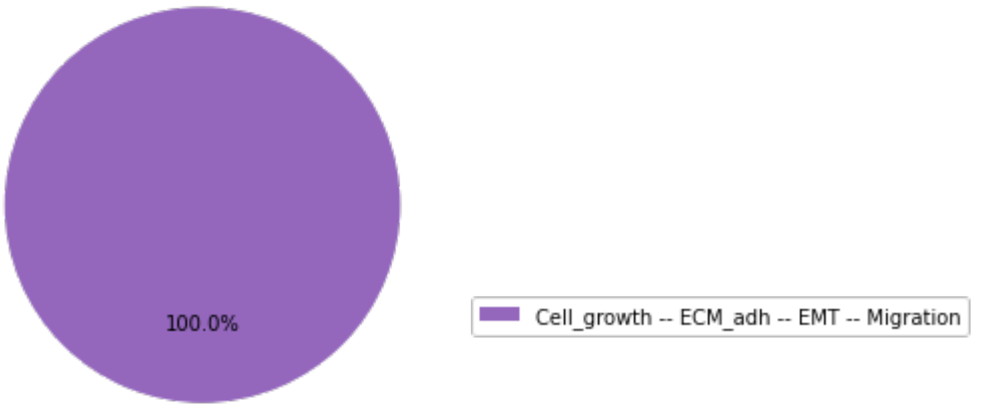

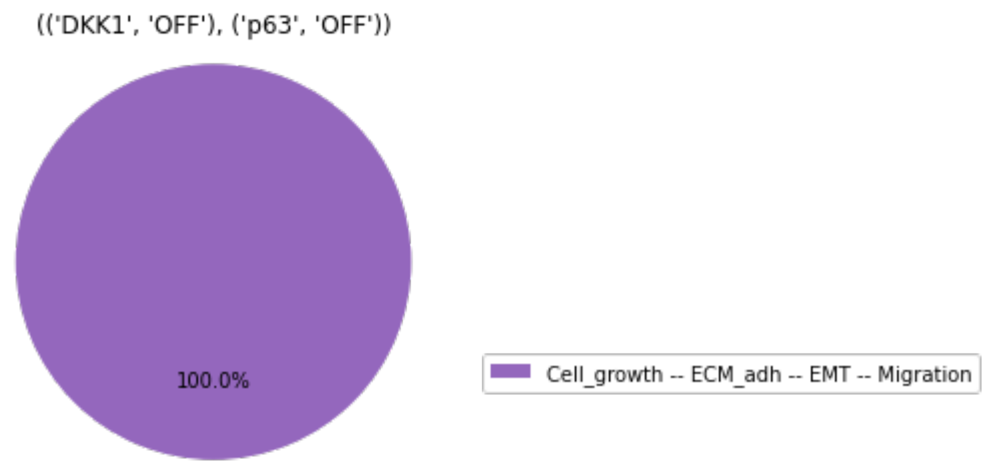
(('DKK1', 'OFF'), ('p63', 'OFF'))
100.0%
Cell_growth -- ECM_adh -- EMT -- Migration

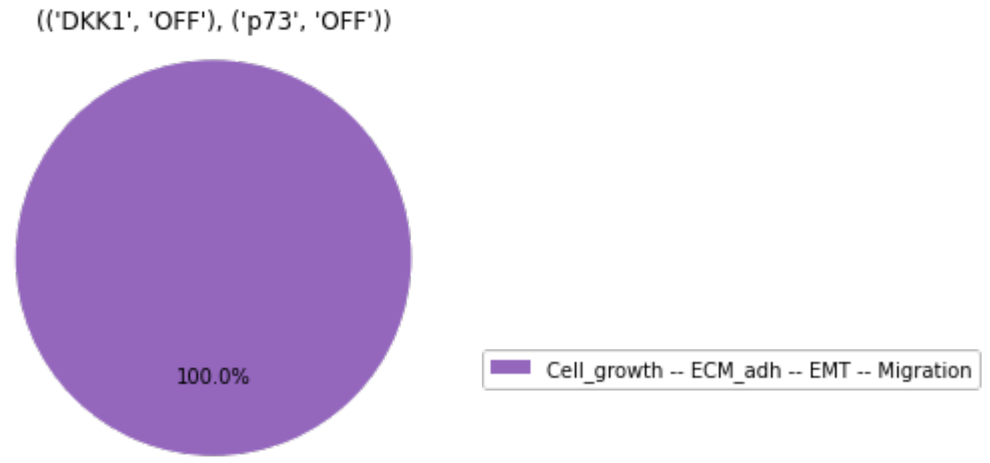
(('DKK1', 'OFF'), ('p73', 'OFF'))
100.0%
Cell_growth -- ECM_adh -- EMT -- Migration

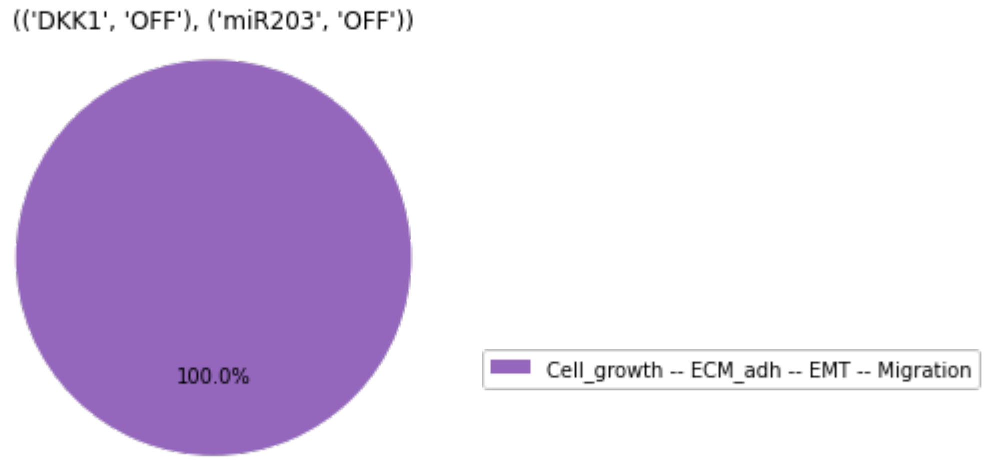
(('DKK1', 'OFF'), ('miR203', 'OFF'))
100.0%
Cell_growth -- ECM_adh -- EMT -- Migration

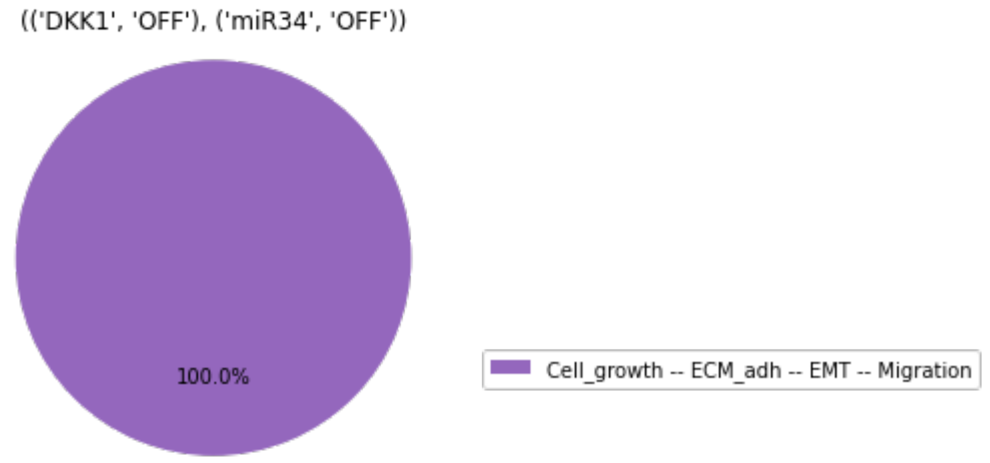
(('DKK1', 'OFF'), ('miR34', 'OFF'))
100.0%
Cell_growth -- ECM_adh -- EMT -- Migration

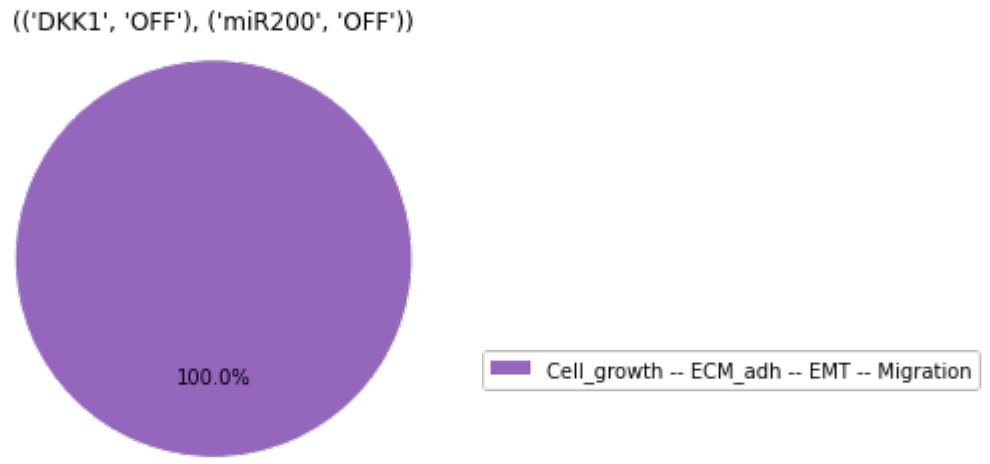
(('DKK1', 'OFF'), ('miR200', 'OFF'))
100.0%
Cell_growth -- ECM_adh -- EMT -- Migration

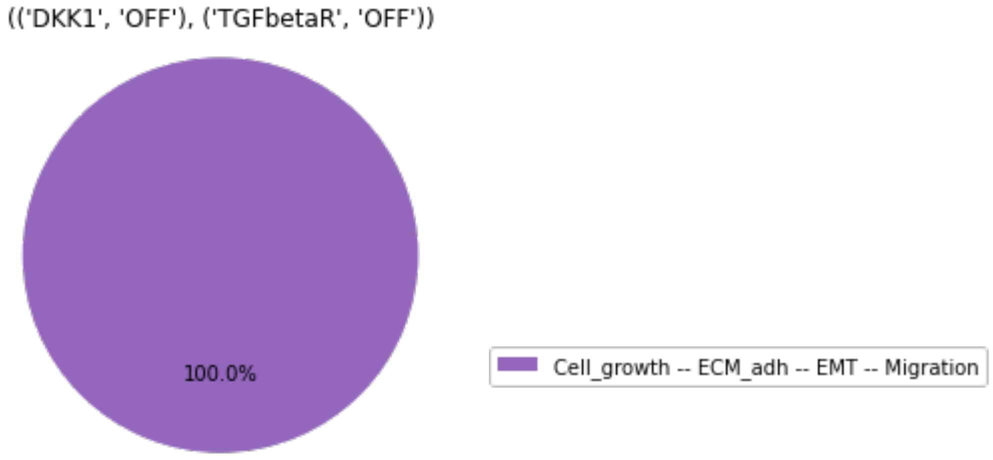
(('DKK1', 'OFF'), ('TGFbetaR', 'OFF'))
100.0%
Cell_growth -- ECM_adh -- EMT -- Migration

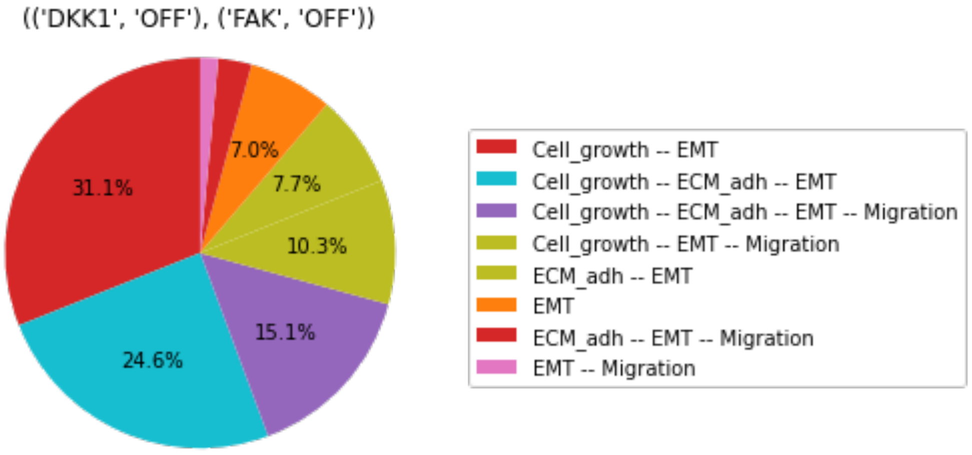
(('DKK1', 'OFF'), ('FAK', 'OFF'))
31.1%
7.0%
7.7%
10.3%
15.1%
24.6%
Cell_growth -- EMT
Cell_growth -- ECM_adh -- EMT
Cell_growth -- ECM_adh -- EMT -- Migration
Cell_growth -- EMT -- Migration
ECM_adh -- EMT
EMT
ECM_adh -- EMT -- Migration
EMT -- Migration

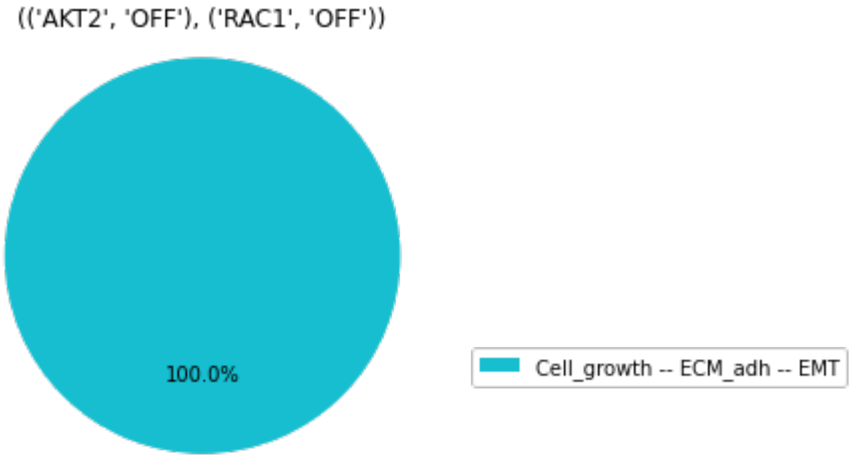
(('AKT2', 'OFF'), ('RAC1', 'OFF'))
100.0%
Cell_growth -- ECM_adh -- EMT

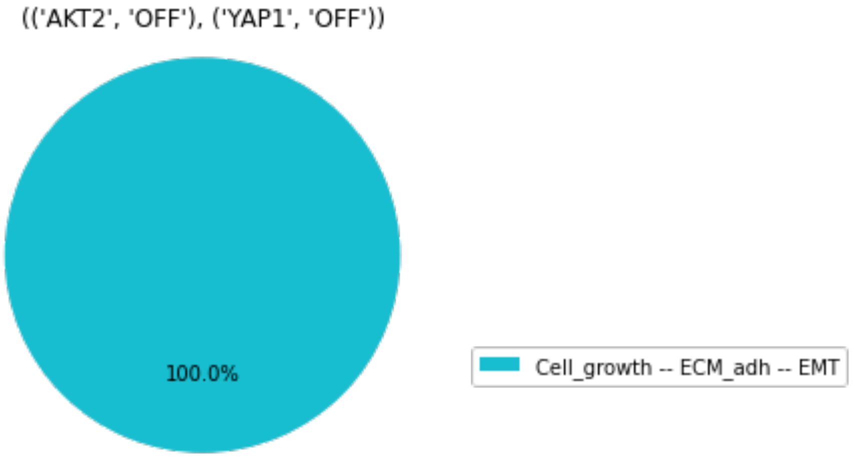
(('AKT2', 'OFF'), ('YAP1', 'OFF'))
100.0%
Cell_growth -- ECM_adh -- EMT

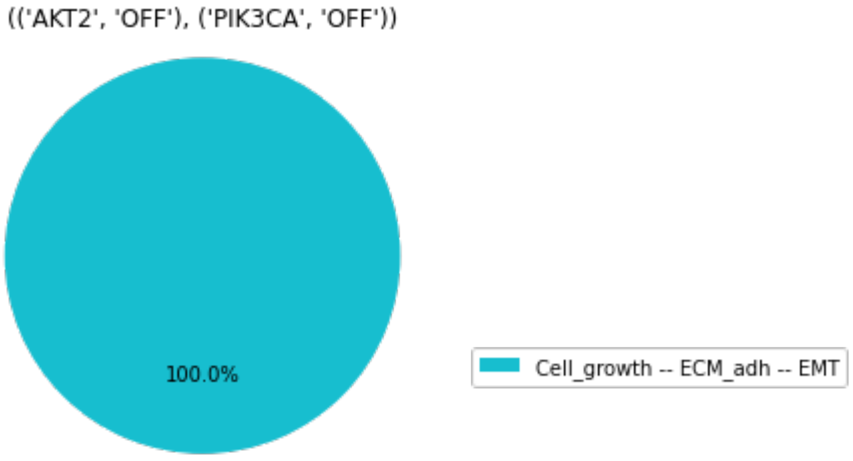
(('AKT2', 'OFF'), ('PIK3CA', 'OFF'))
100.0%
Cell_growth -- ECM_adh -- EMT

(('AKT2', 'OFF'), ('ERK', 'OFF'))

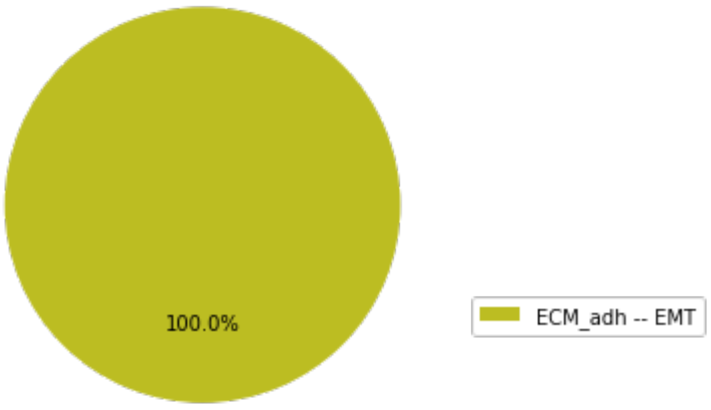

(('AKT2', 'OFF'), ('p21', 'OFF'))

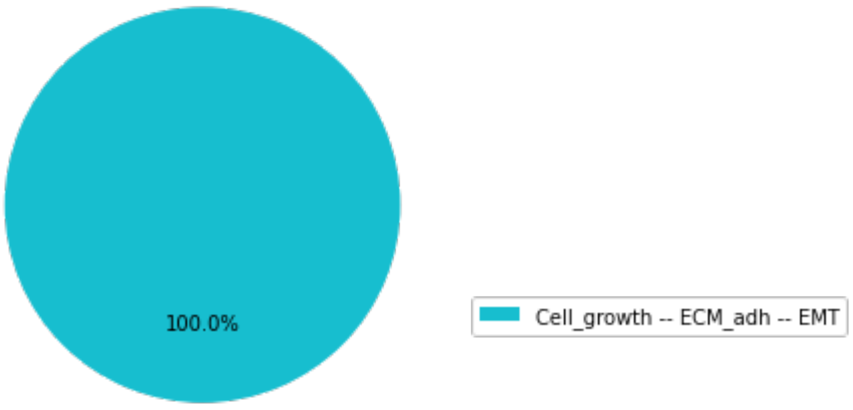

(('AKT2', 'OFF'), ('SMAD', 'OFF'))

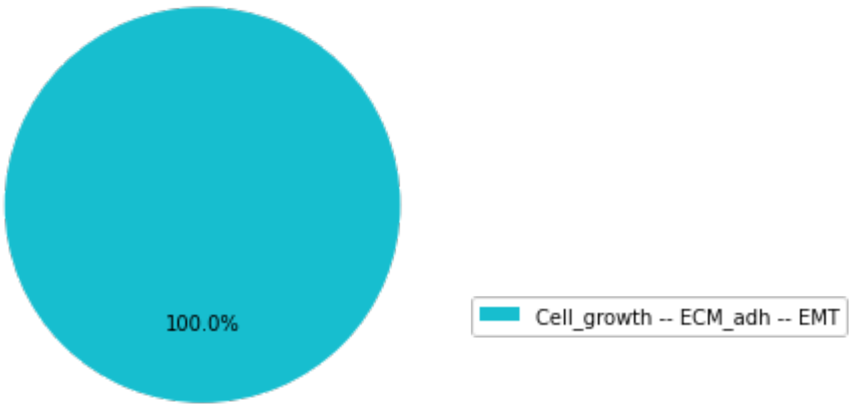

(('AKT2', 'OFF'), ('VIM', 'OFF'))

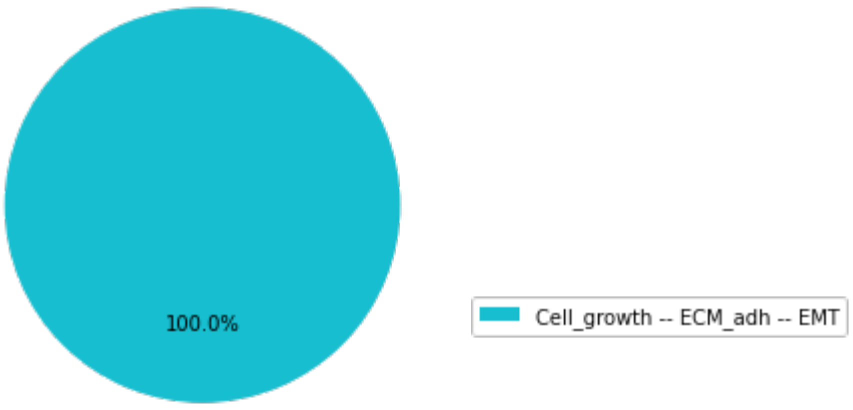

(('AKT2', 'OFF'), ('p53', 'OFF'))

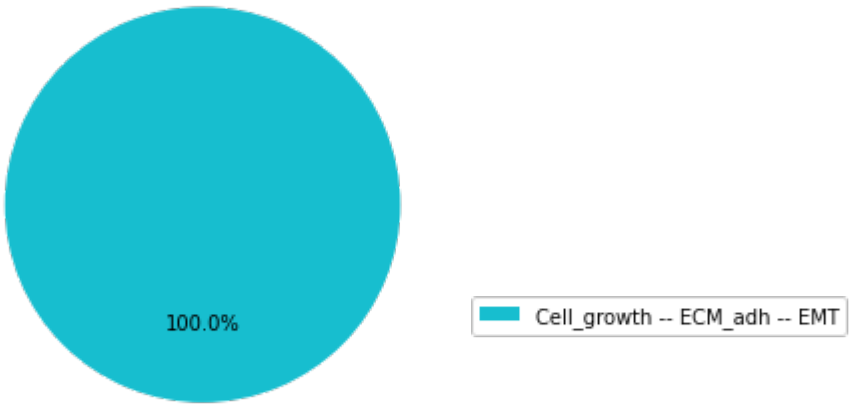

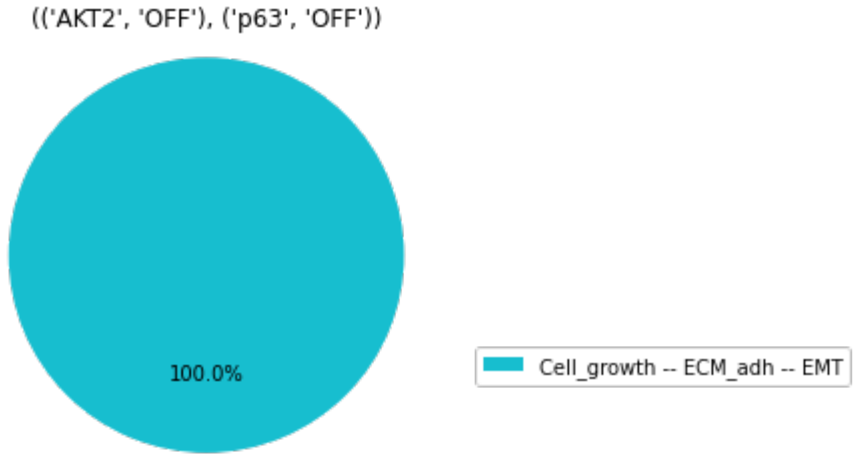
(('AKT2', 'OFF'), ('p63', 'OFF'))
100.0%
Cell_growth -- ECM_adh -- EMT

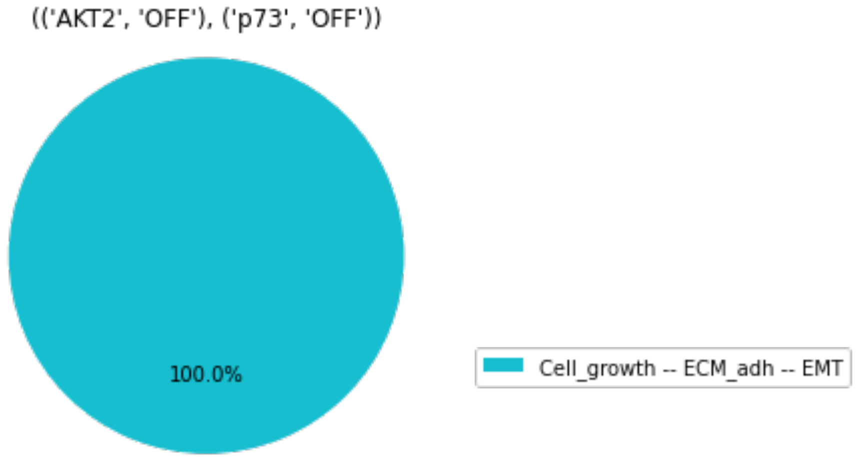
(('AKT2', 'OFF'), ('p73', 'OFF'))
100.0%
Cell_growth -- ECM_adh -- EMT

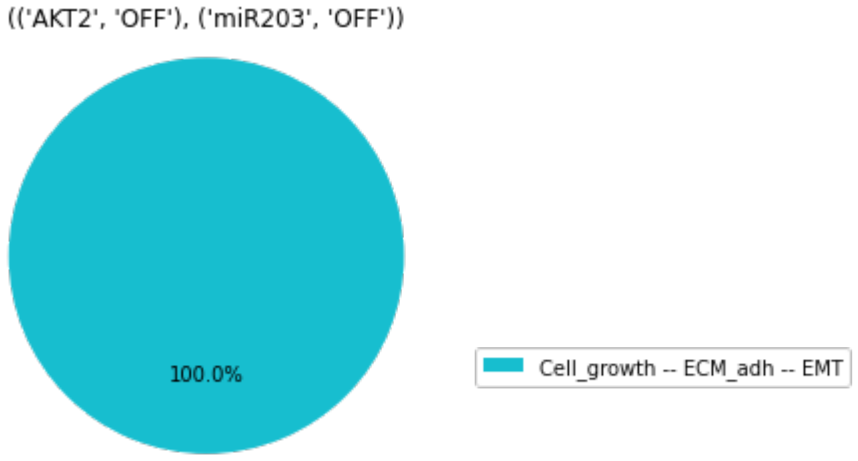
(('AKT2', 'OFF'), ('miR203', 'OFF'))
100.0%
Cell_growth -- ECM_adh -- EMT

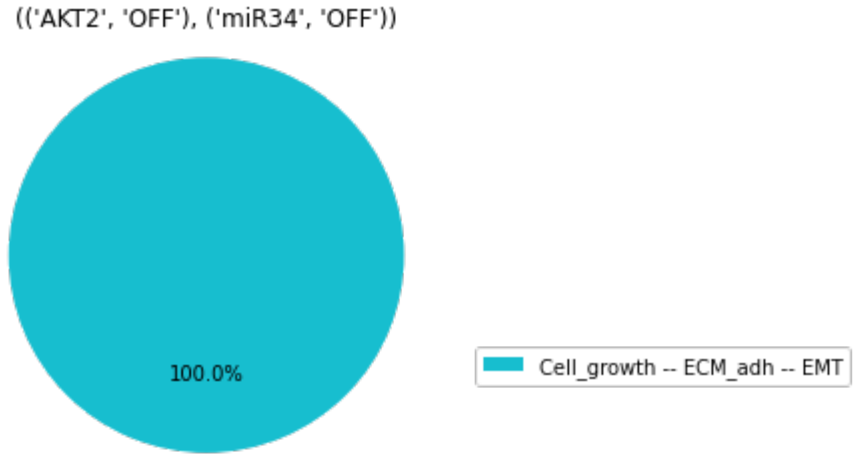
(('AKT2', 'OFF'), ('miR34', 'OFF'))
100.0%
Cell_growth -- ECM_adh -- EMT

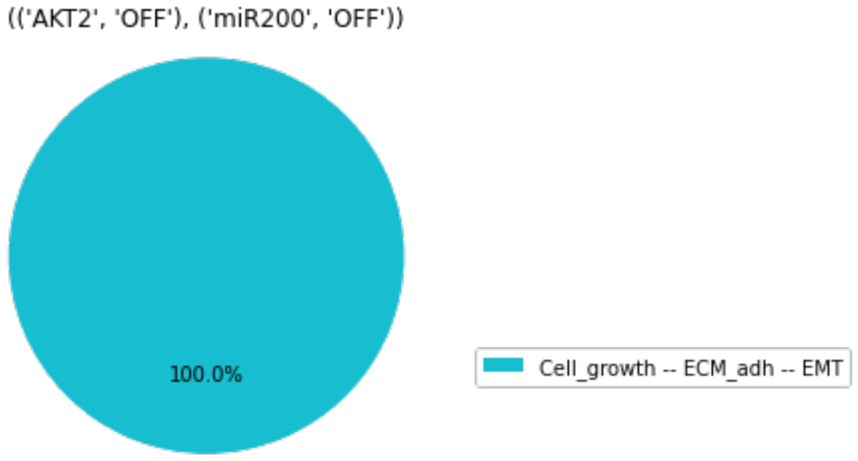
(('AKT2', 'OFF'), ('miR200', 'OFF'))
100.0%
Cell_growth -- ECM_adh -- EMT

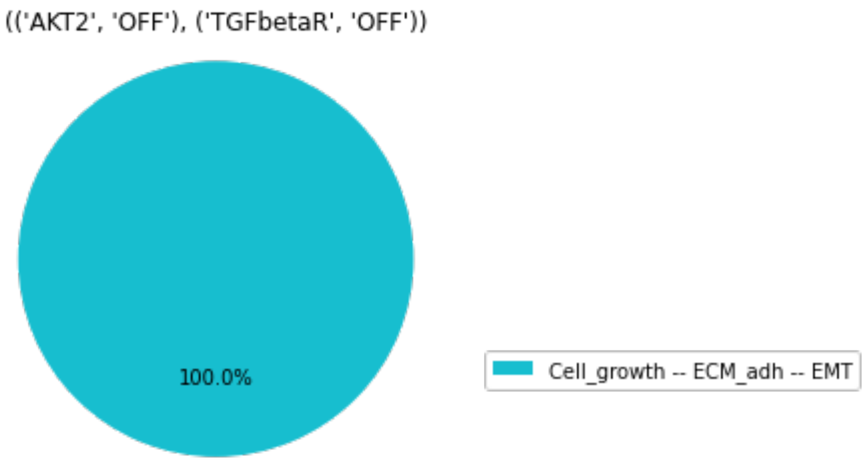
(('AKT2', 'OFF'), ('TGFbetaR', 'OFF'))
100.0%
Cell_growth -- ECM_adh -- EMT

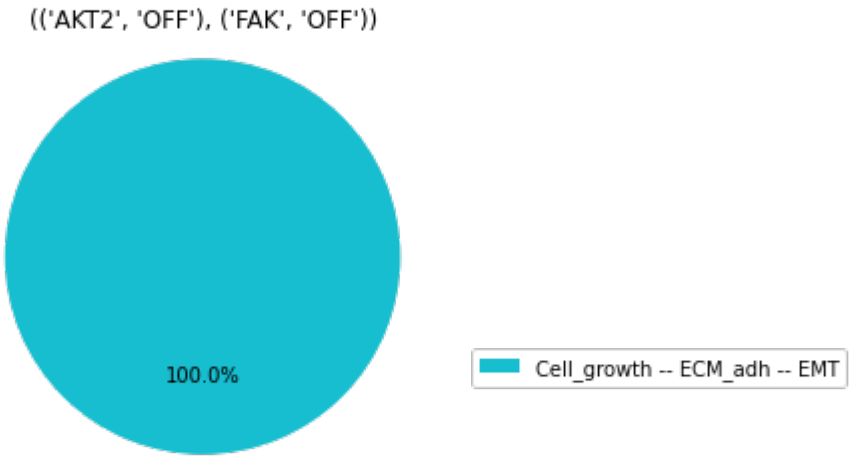
(('AKT2', 'OFF'), ('FAK', 'OFF'))
100.0%
Cell_growth -- ECM_adh -- EMT

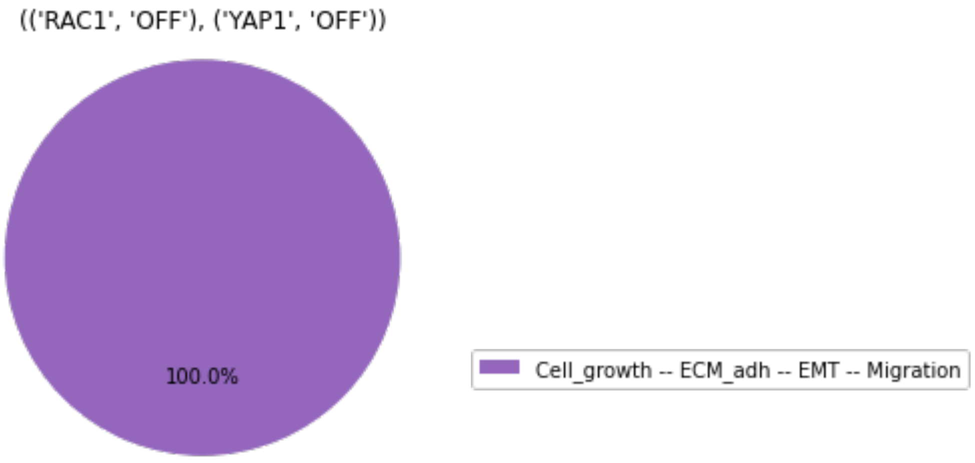
(('RAC1', 'OFF'), ('YAP1', 'OFF'))
100.0%
Cell_growth -- ECM_adh -- EMT -- Migration

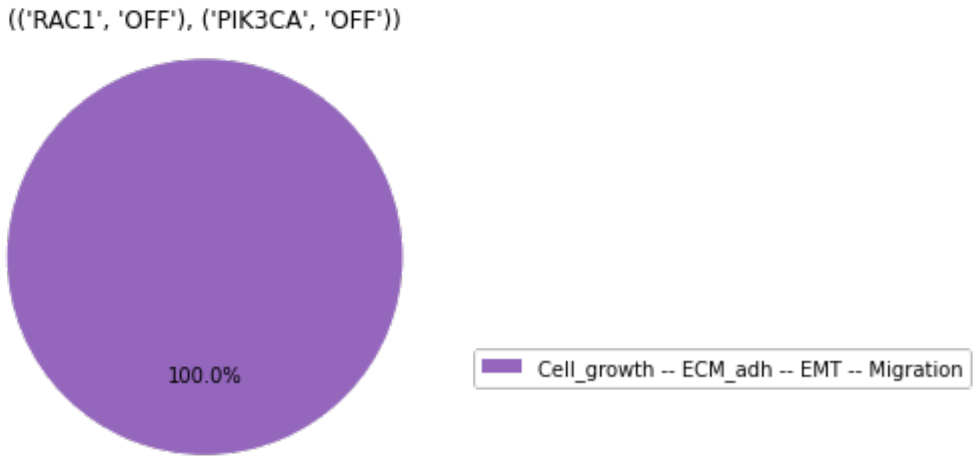
(('RAC1', 'OFF'), ('PIK3CA', 'OFF'))
100.0%
Cell_growth -- ECM_adh -- EMT -- Migration

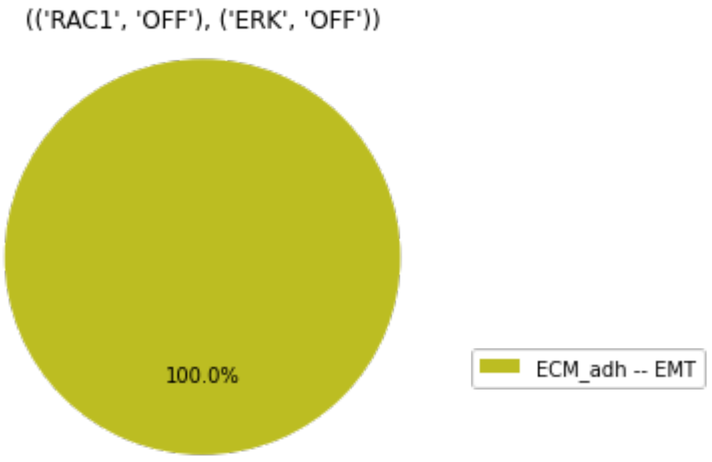
(('RAC1', 'OFF'), ('ERK', 'OFF'))
100.0%
ECM_adh -- EMT

(('RAC1', 'OFF'), ('p21', 'OFF'))

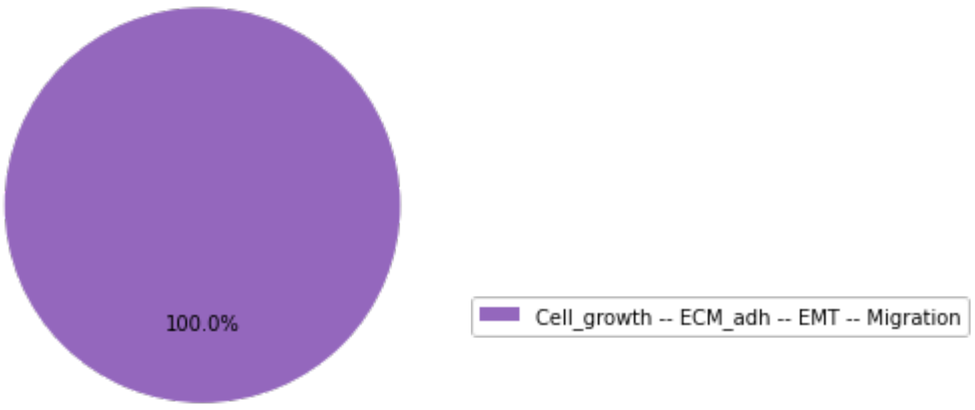

(('RAC1', 'OFF'), ('SMAD', 'OFF'))

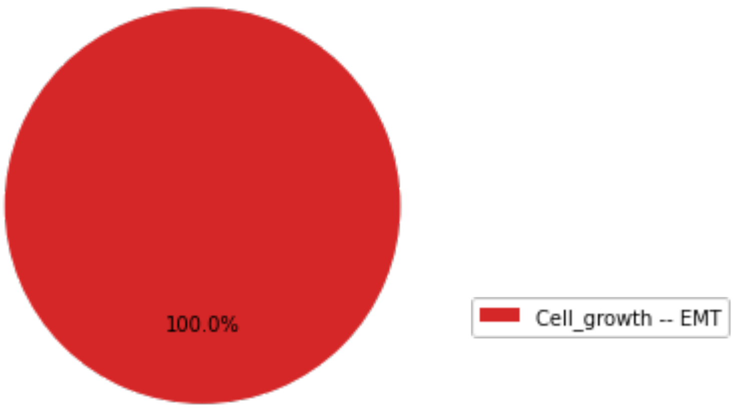

(('RAC1', 'OFF'), ('VIM', 'OFF'))

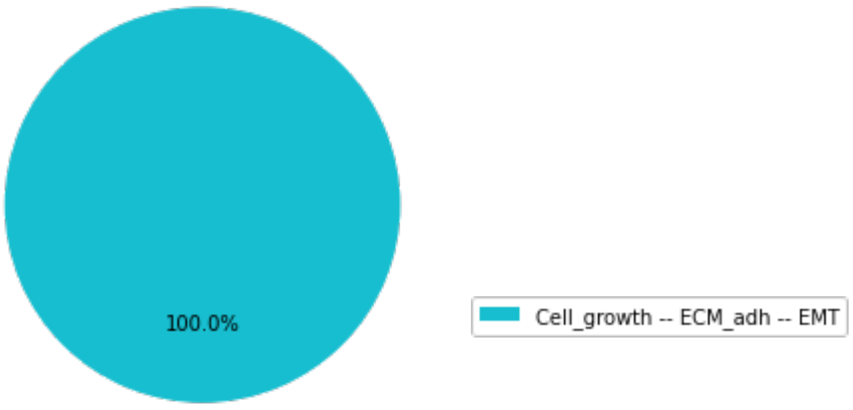

(('RAC1', 'OFF'), ('p53', 'OFF'))

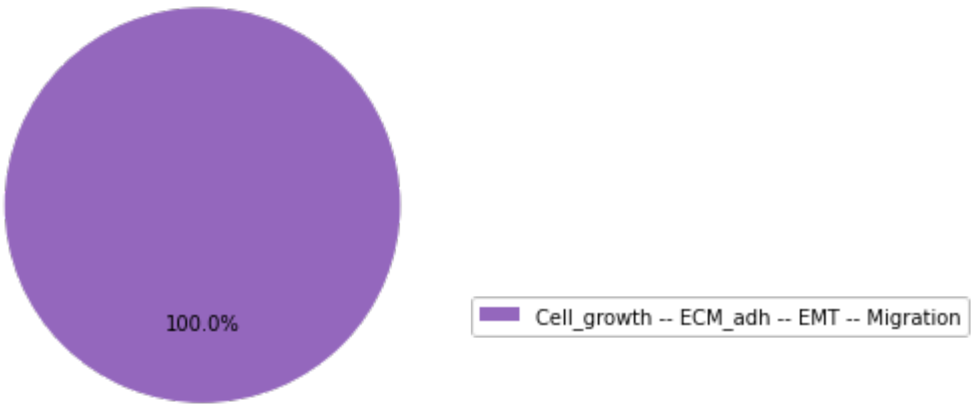

(('RAC1', 'OFF'), ('p63', 'OFF'))

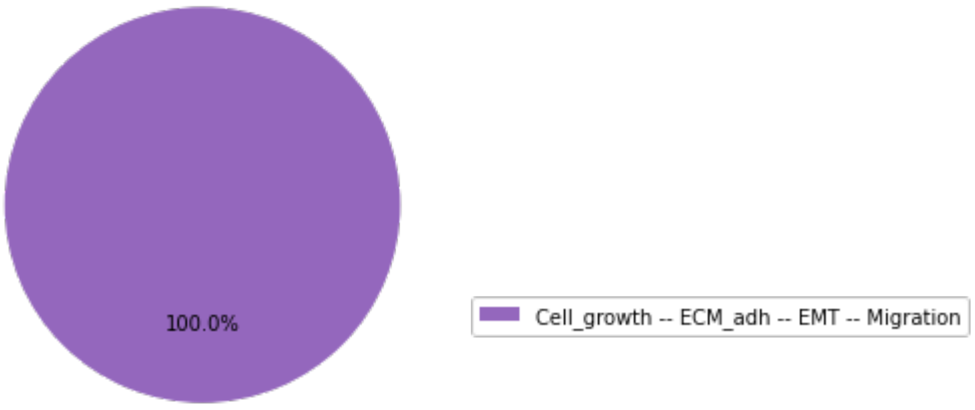

(('RAC1', 'OFF'), ('p73', 'OFF'))

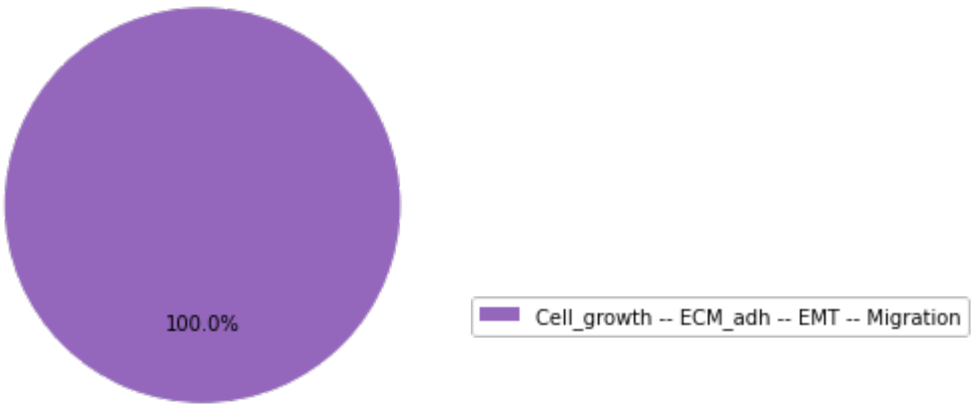

(('RAC1', 'OFF'), ('miR203', 'OFF'))

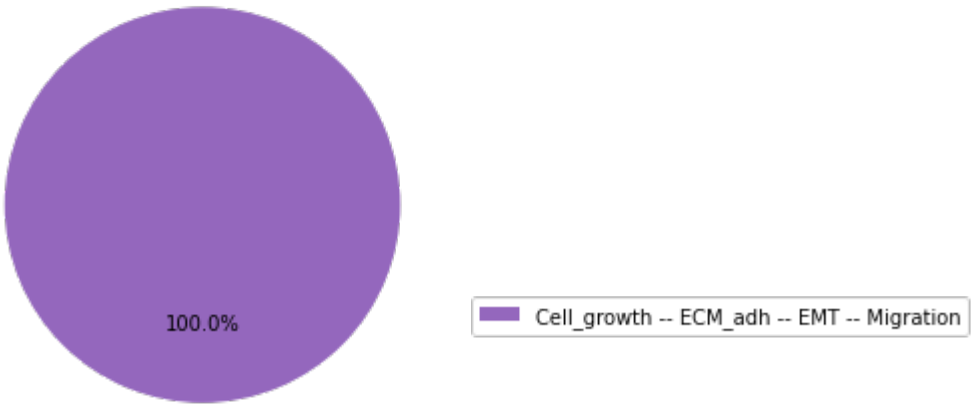

(('RAC1', 'OFF'), ('miR34', 'OFF'))

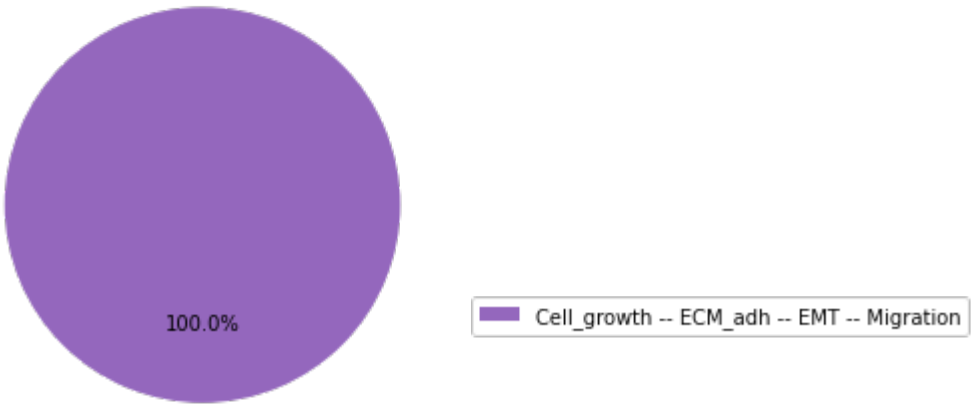

(('RAC1', 'OFF'), ('miR200', 'OFF'))

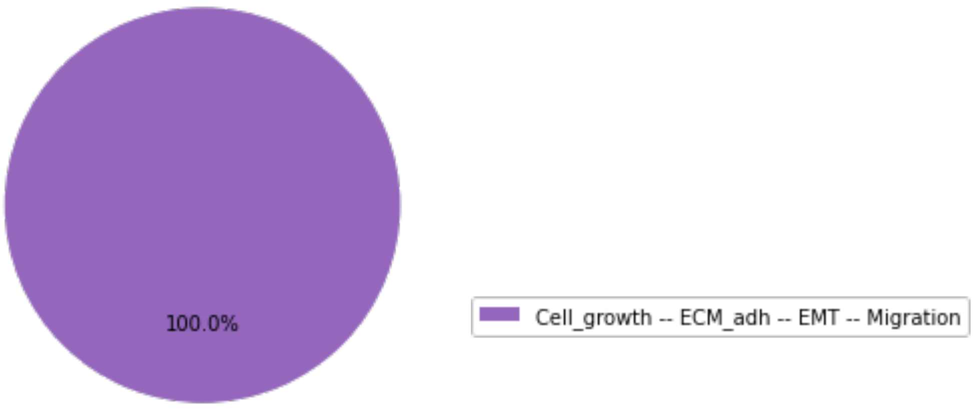

(('RAC1', 'OFF'), ('TGFbetaR', 'OFF'))

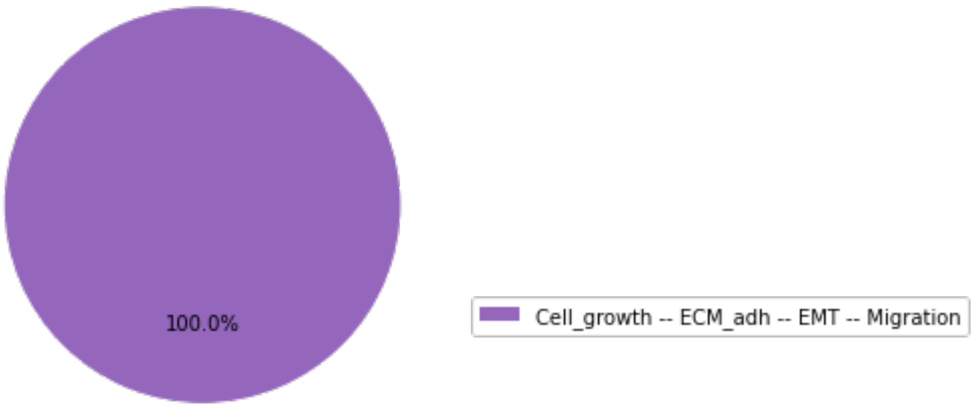

(('RAC1', 'OFF'), ('FAK', 'OFF'))

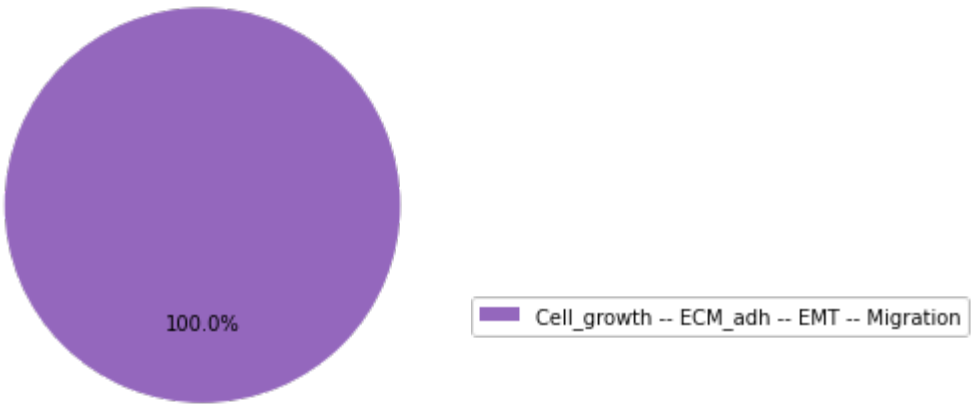

(('YAP1', 'OFF'), ('PIK3CA', 'OFF'))

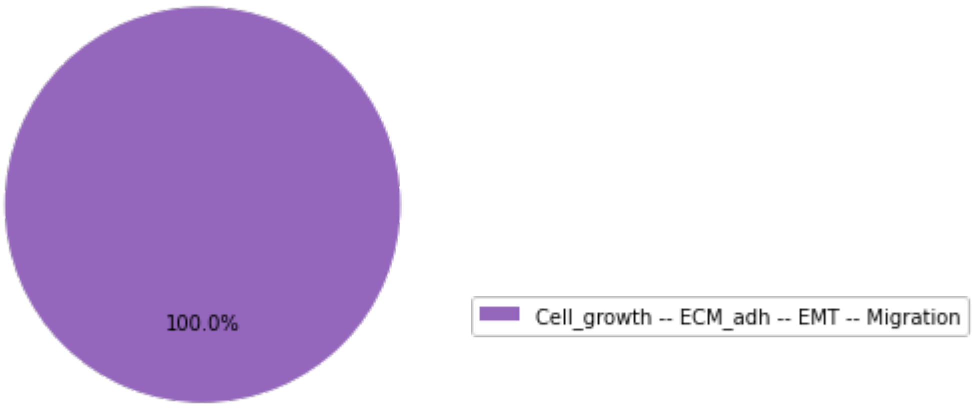

(('YAP1', 'OFF'), ('ERK', 'OFF'))

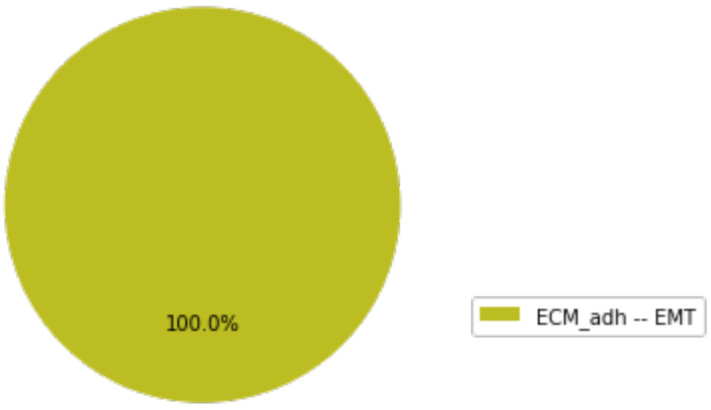

(('YAP1', 'OFF'), ('p21', 'OFF'))

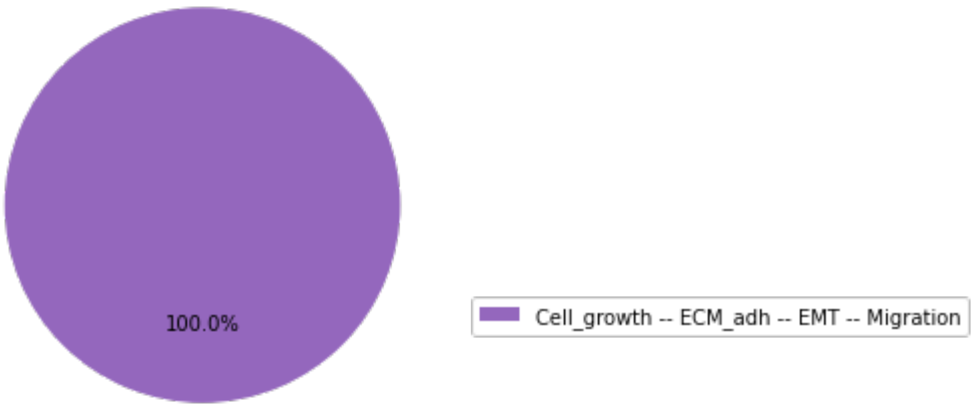

(('YAP1', 'OFF'), ('SMAD', 'OFF'))

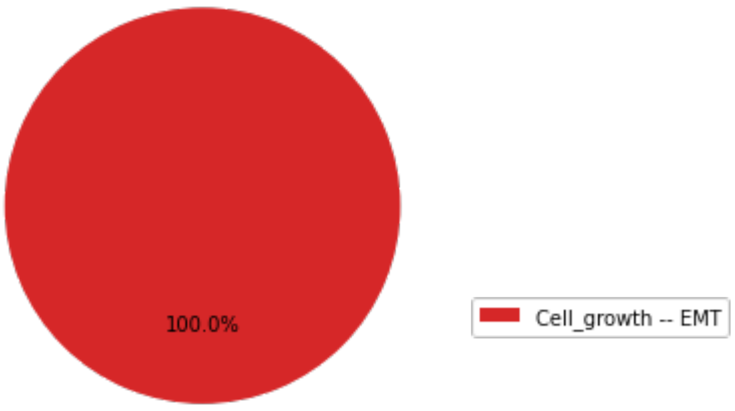

(('YAP1', 'OFF'), ('VIM', 'OFF'))

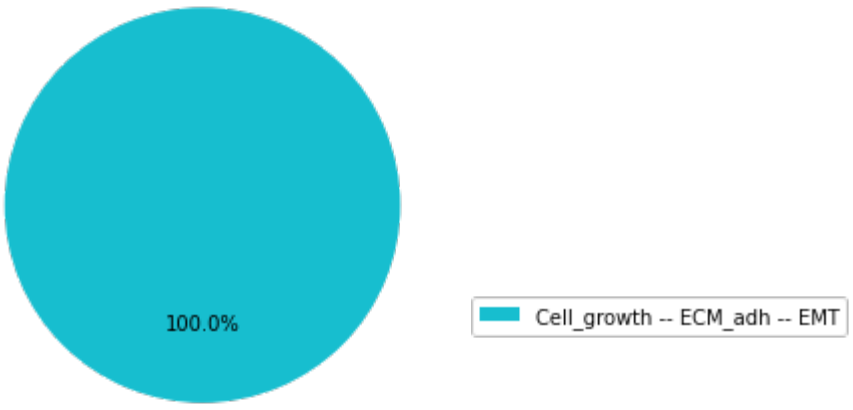

(('YAP1', 'OFF'), ('p53', 'OFF'))

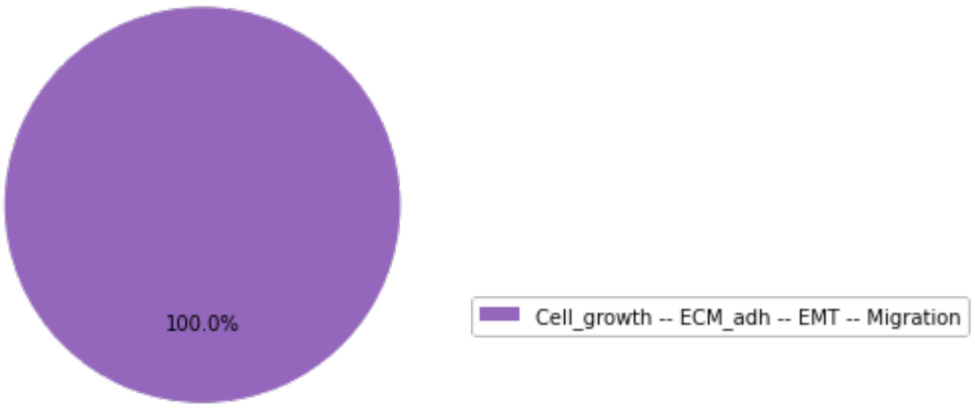

(('YAP1', 'OFF'), ('p63', 'OFF'))

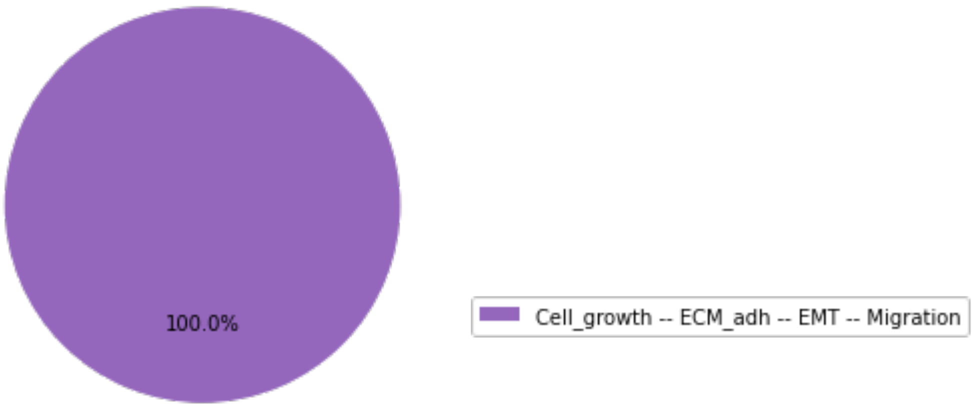

(('YAP1', 'OFF'), ('p73', 'OFF'))

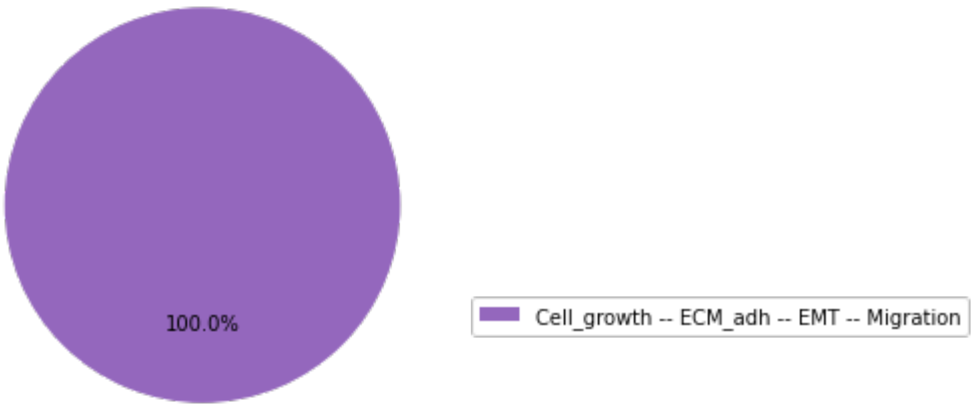

(('YAP1', 'OFF'), ('miR203', 'OFF'))

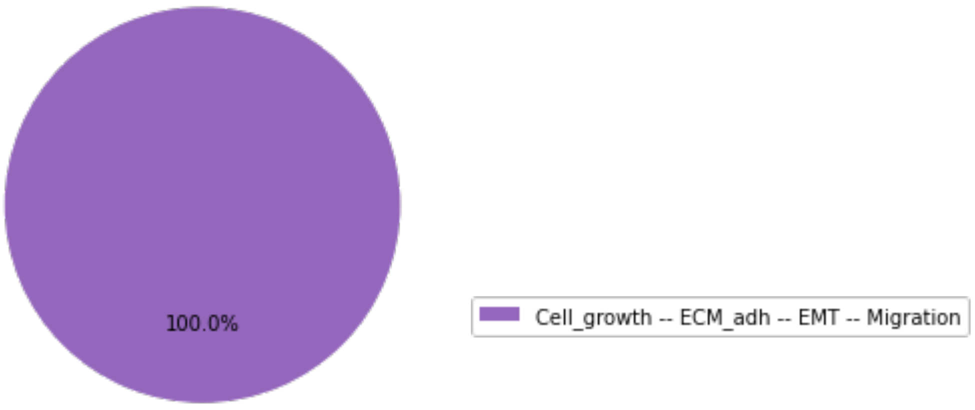

(('YAP1', 'OFF'), ('miR34', 'OFF'))

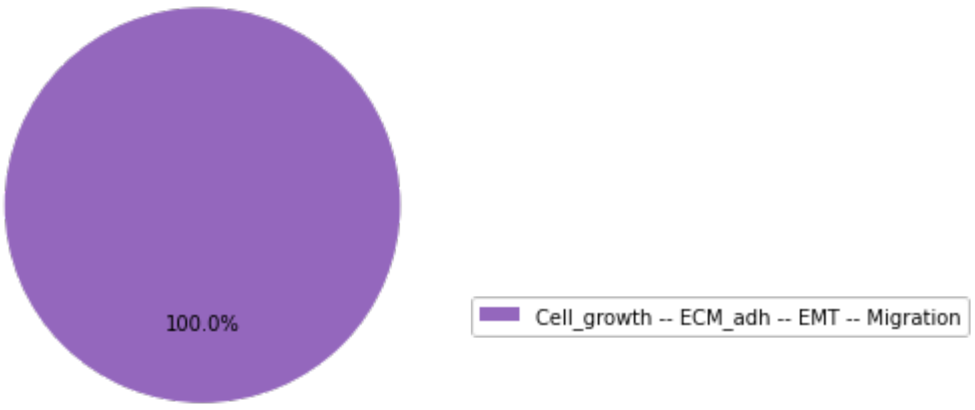

(('YAP1', 'OFF'), ('miR200', 'OFF'))

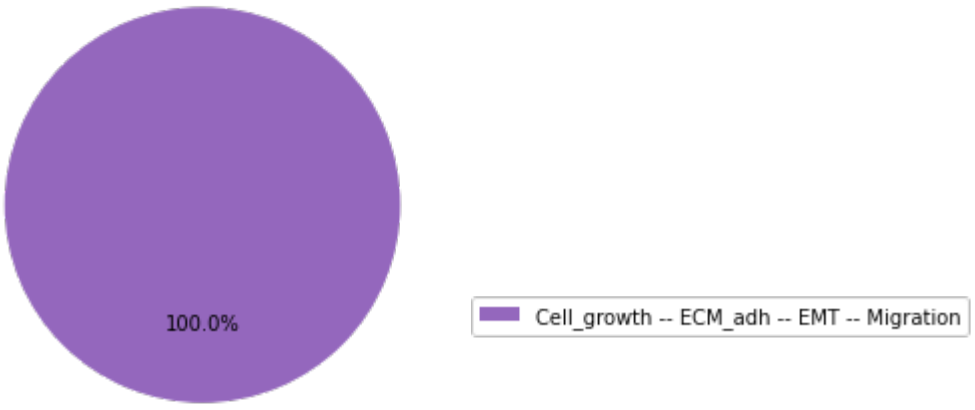

(('YAP1', 'OFF'), ('TGFbetaR', 'OFF'))

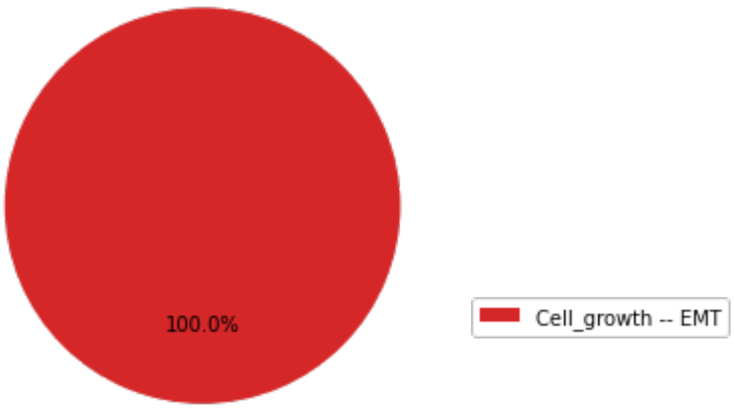

(('YAP1', 'OFF'), ('FAK', 'OFF'))

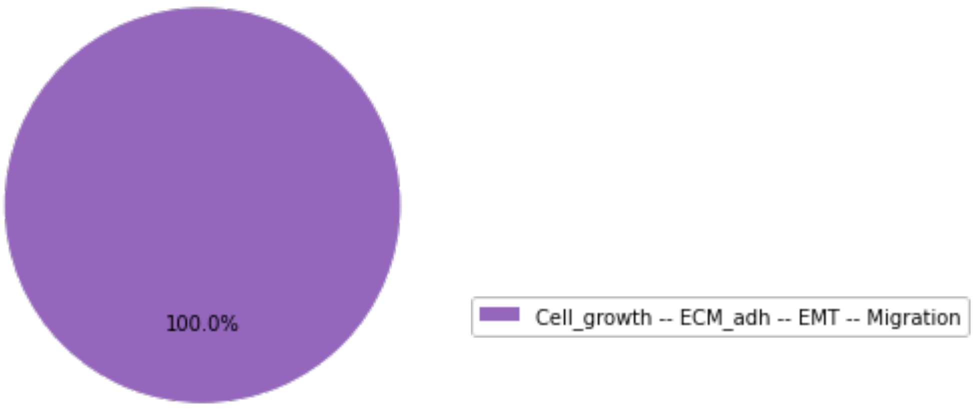

(('PIK3CA', 'OFF'), ('ERK', 'OFF'))

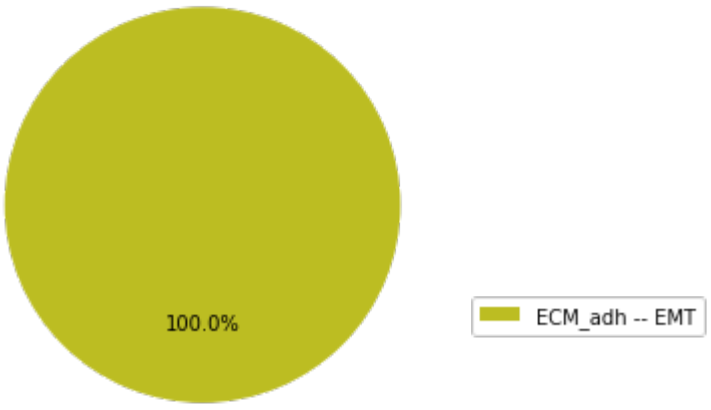

(('PIK3CA', 'OFF'), ('p21', 'OFF'))

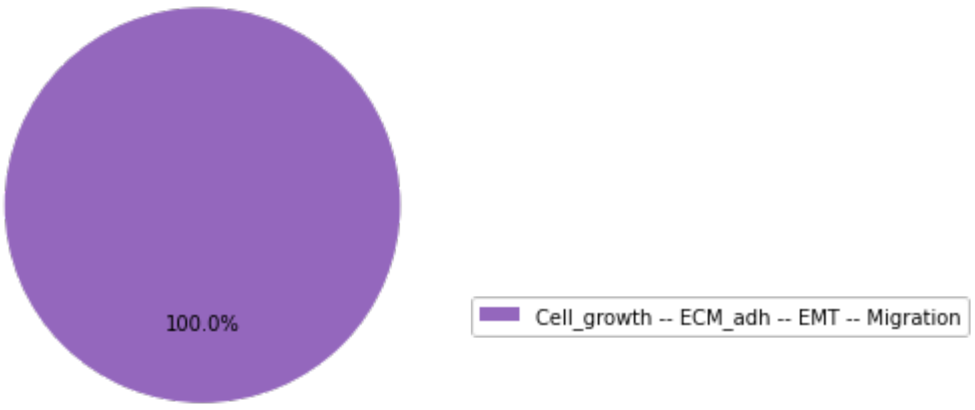

(('PIK3CA', 'OFF'), ('SMAD', 'OFF'))

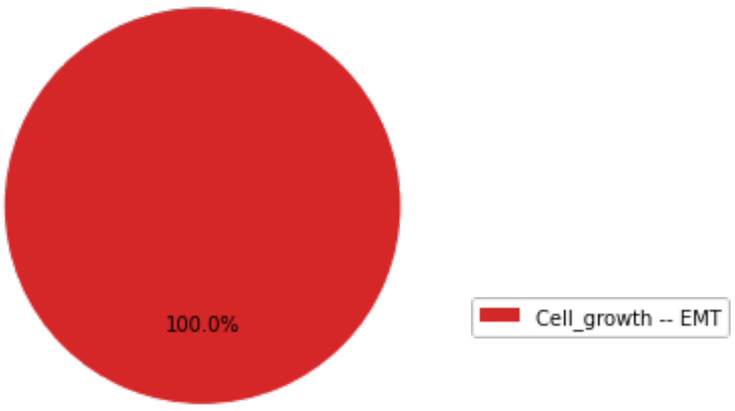

(('PIK3CA', 'OFF'), ('VIM', 'OFF'))

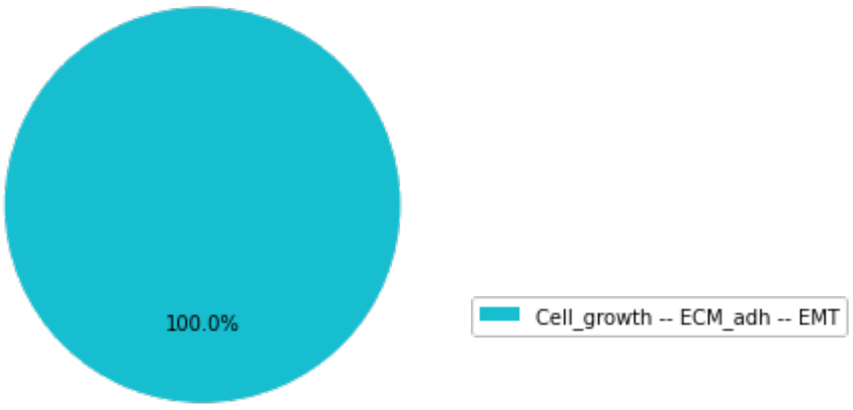

(('PIK3CA', 'OFF'), ('p53', 'OFF'))

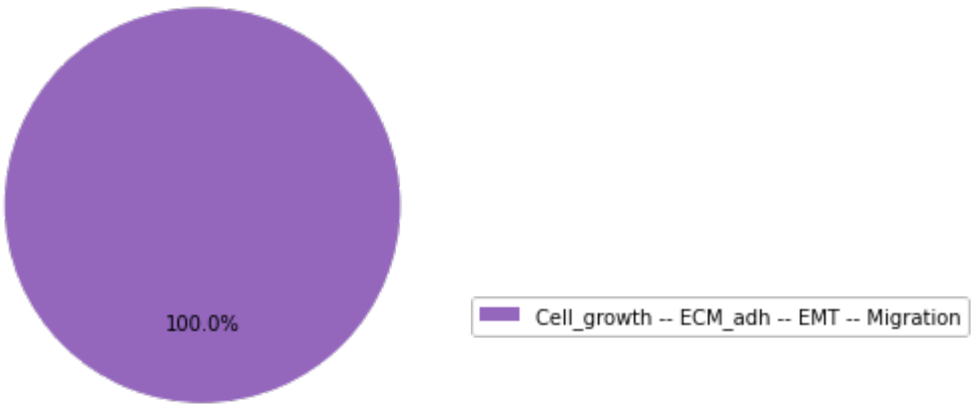

(('PIK3CA', 'OFF'), ('p63', 'OFF'))

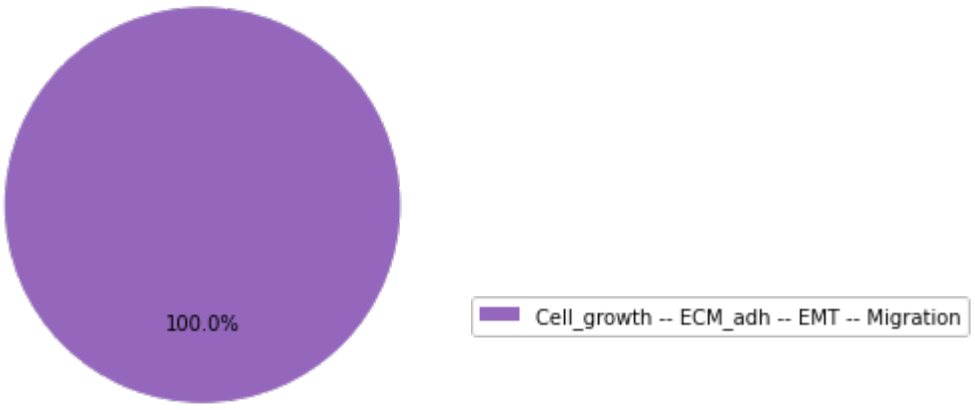

(('PIK3CA', 'OFF'), ('p73', 'OFF'))

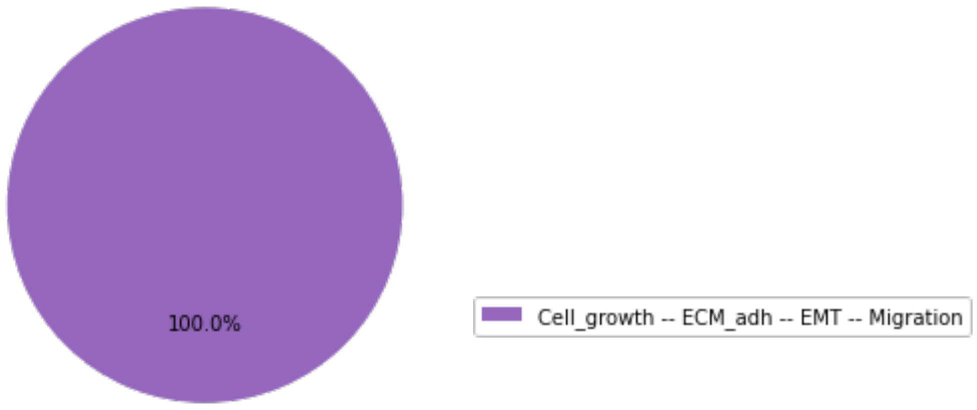

(('PIK3CA', 'OFF'), ('miR203', 'OFF'))

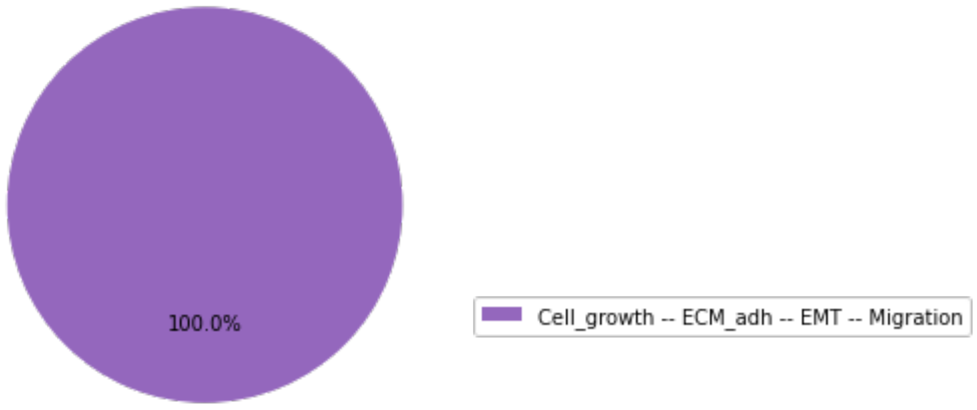

(('PIK3CA', 'OFF'), ('miR34', 'OFF'))

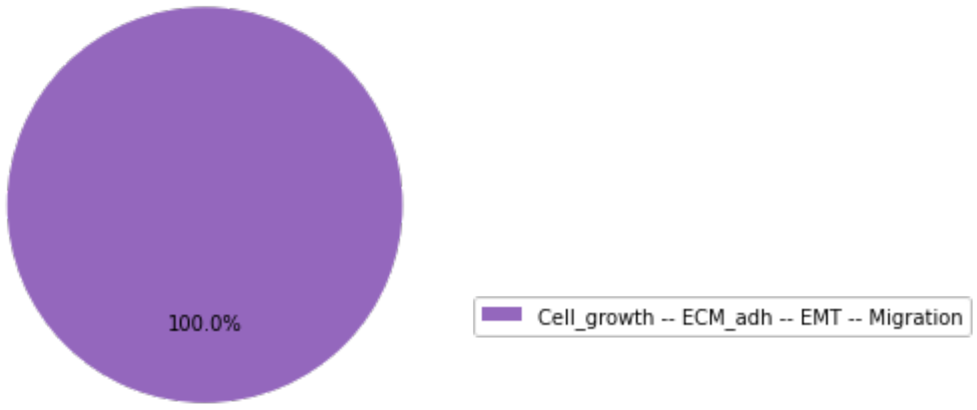

(('PIK3CA', 'OFF'), ('miR200', 'OFF'))

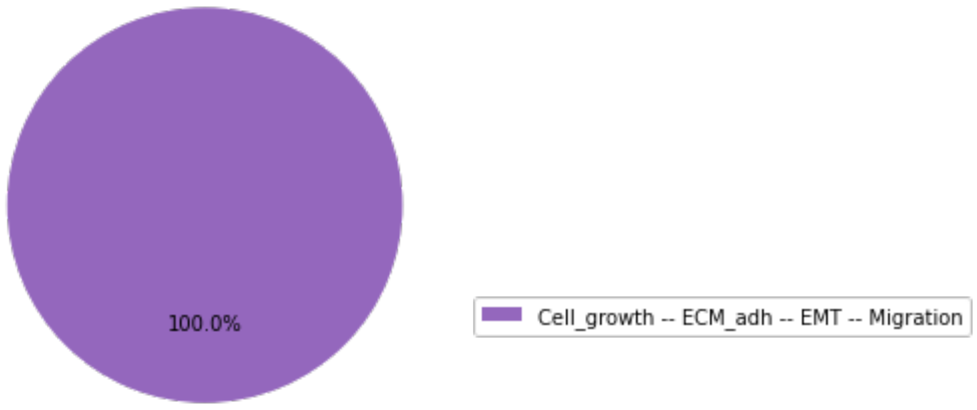

(('PIK3CA', 'OFF'), ('TGFbetaR', 'OFF'))

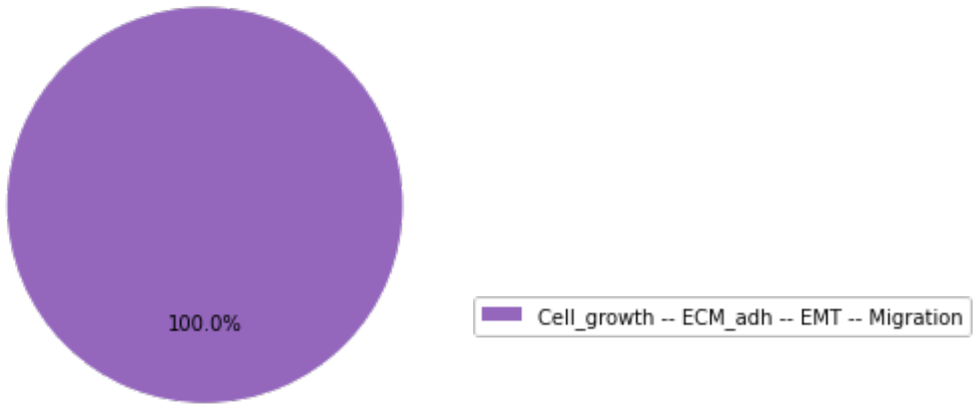

(('PIK3CA', 'OFF'), ('FAK', 'OFF'))

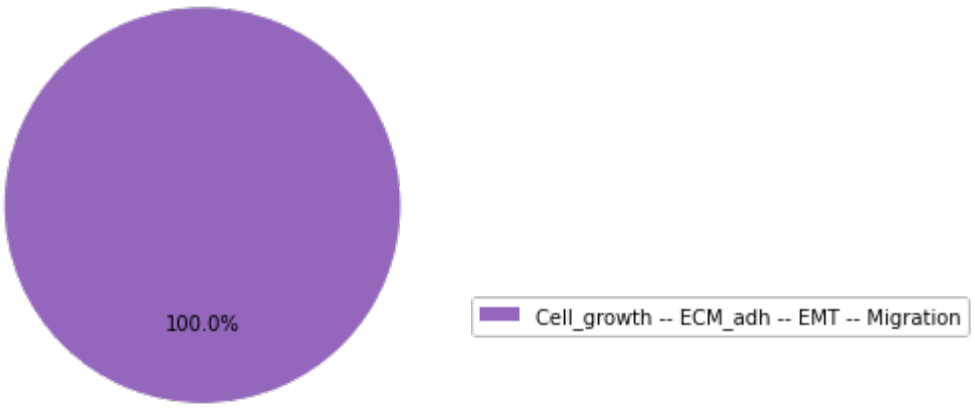

(('ERK', 'OFF'), ('p21', 'OFF'))

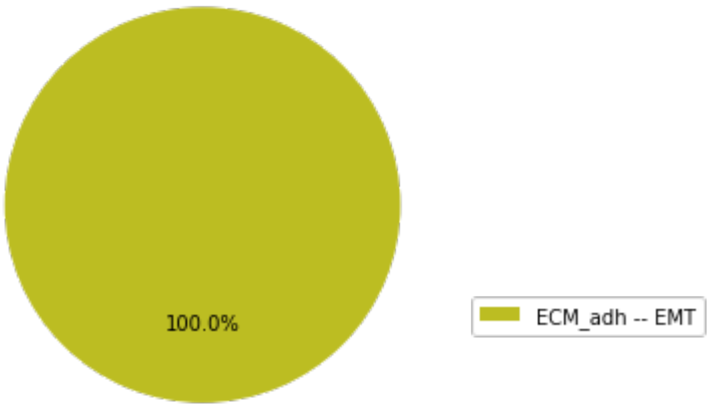

(('ERK', 'OFF'), ('SMAD', 'OFF'))

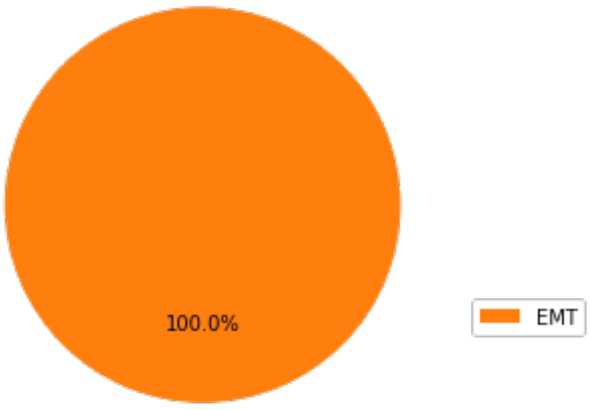

(('ERK', 'OFF'), ('VIM', 'OFF'))

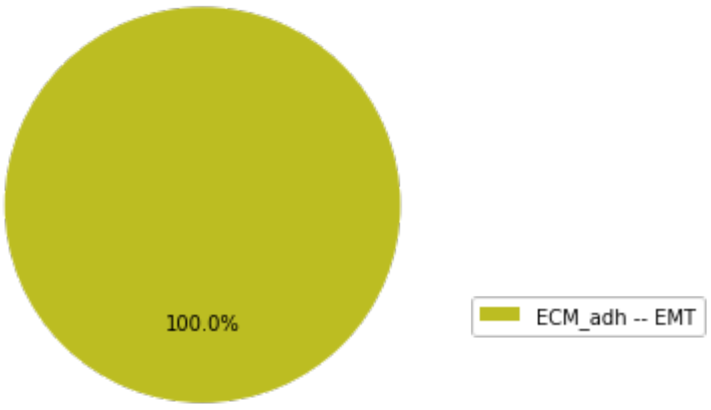

(('ERK', 'OFF'), ('p53', 'OFF'))

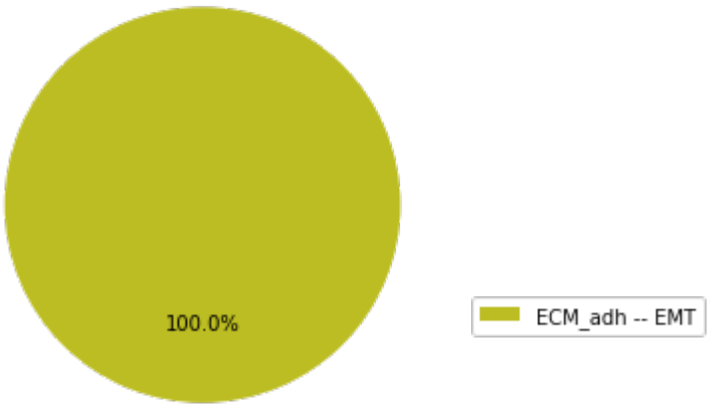

(('ERK', 'OFF'), ('p63', 'OFF'))

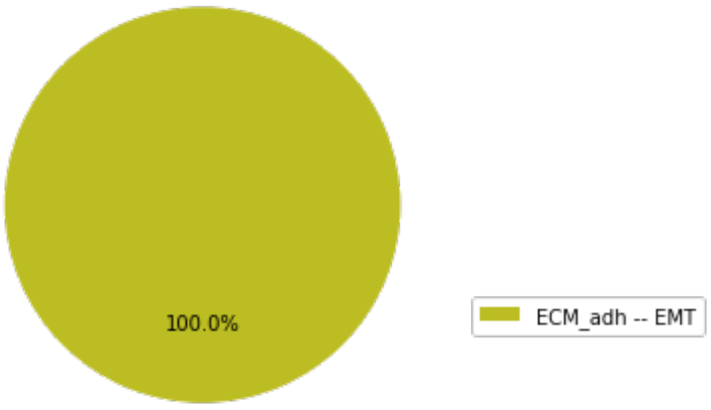

(('ERK', 'OFF'), ('p73', 'OFF'))

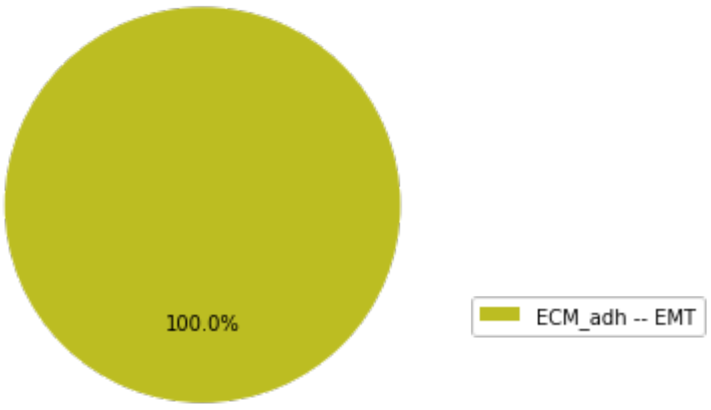

(('ERK', 'OFF'), ('miR203', 'OFF'))

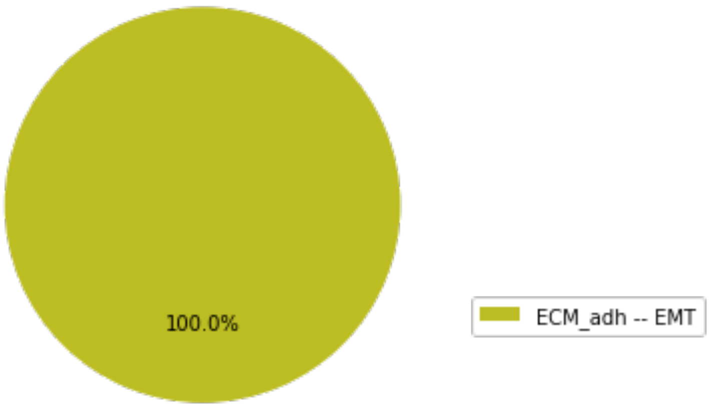

(('ERK', 'OFF'), ('miR34', 'OFF'))

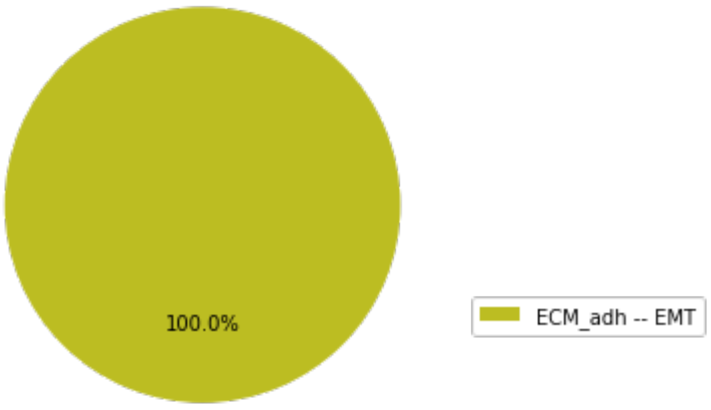

(('ERK', 'OFF'), ('miR200', 'OFF'))

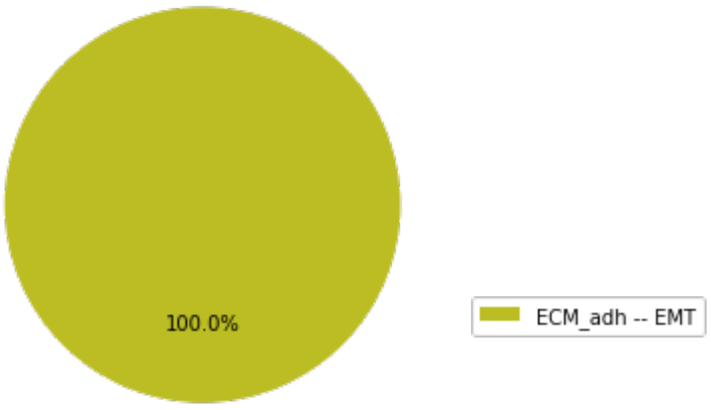

(('ERK', 'OFF'), ('TGFbetaR', 'OFF'))

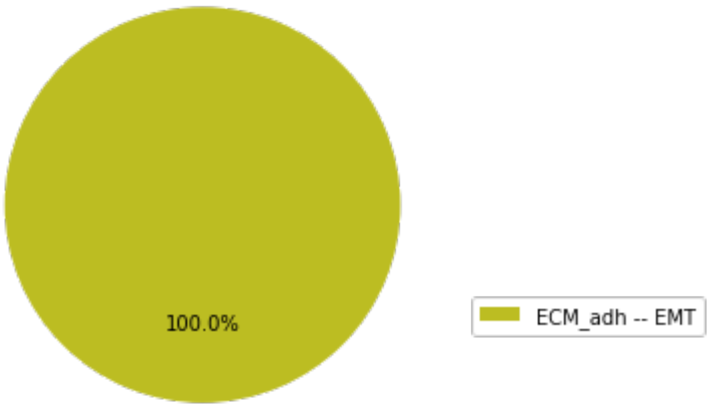

(('ERK', 'OFF'), ('FAK', 'OFF'))

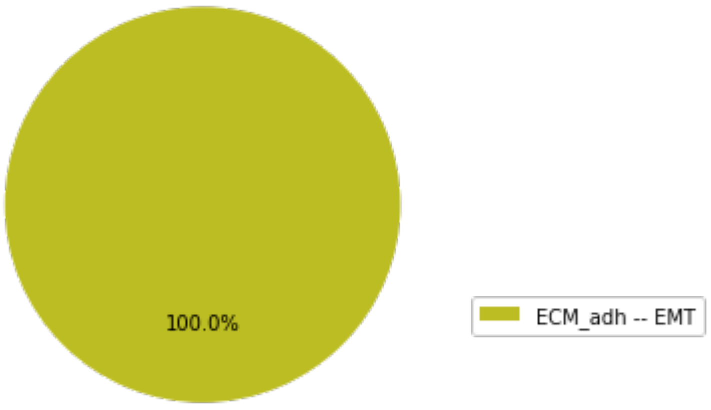

(('p21', 'OFF'), ('SMAD', 'OFF'))

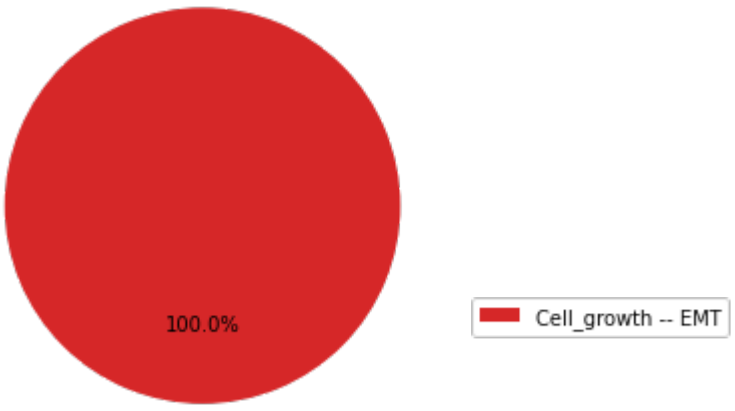

(('p21', 'OFF'), ('VIM', 'OFF'))

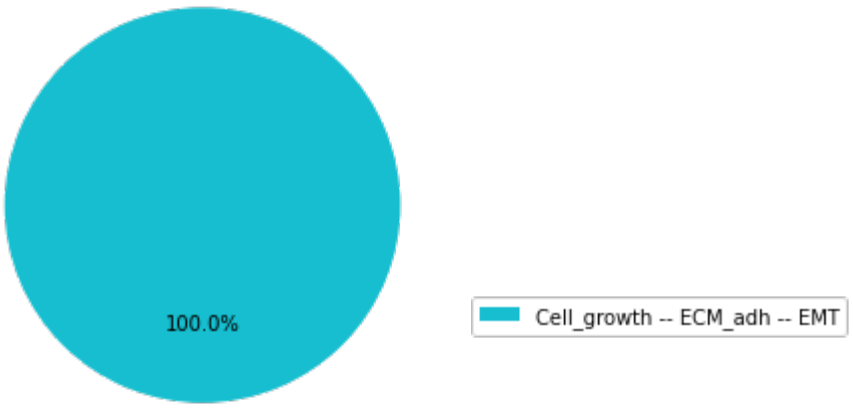

(('p21', 'OFF'), ('p53', 'OFF'))

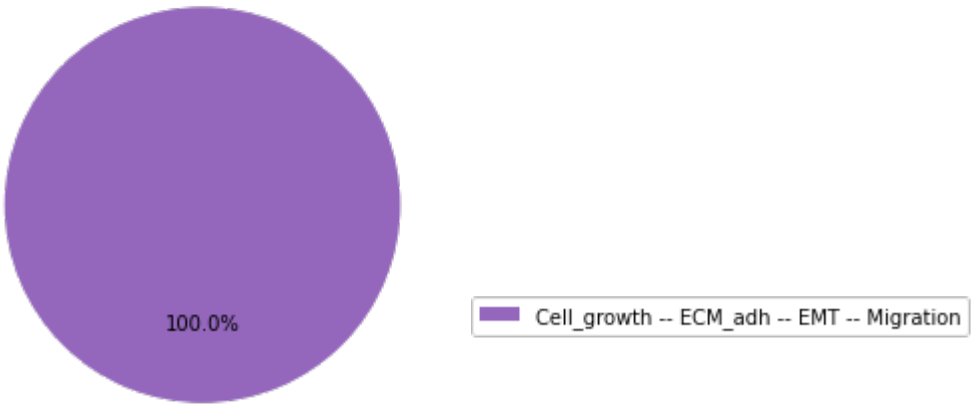

(('p21', 'OFF'), ('p63', 'OFF'))

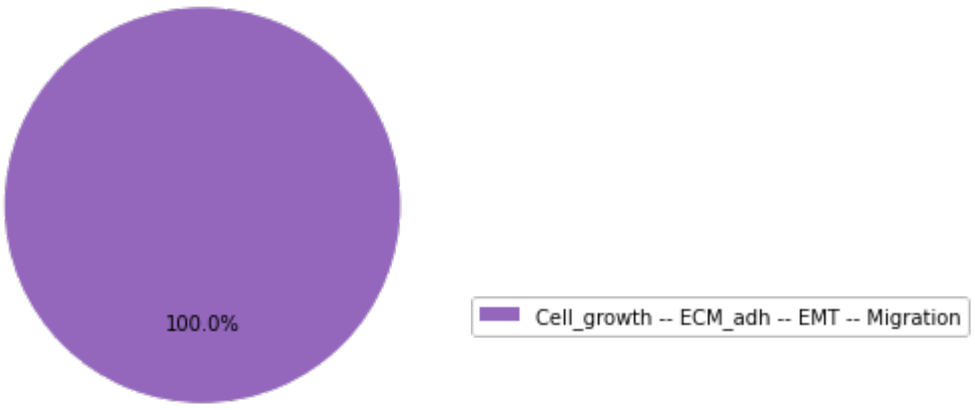

(('p21', 'OFF'), ('p73', 'OFF'))

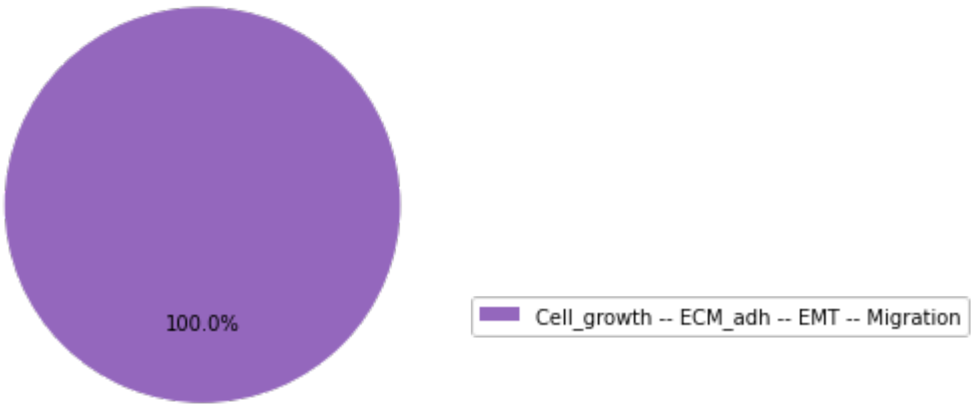

(('p21', 'OFF'), ('miR203', 'OFF'))

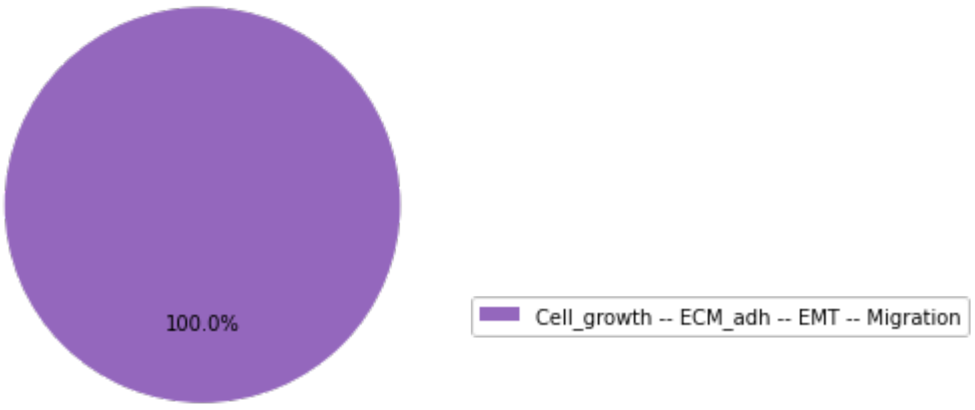

(('p21', 'OFF'), ('miR34', 'OFF'))

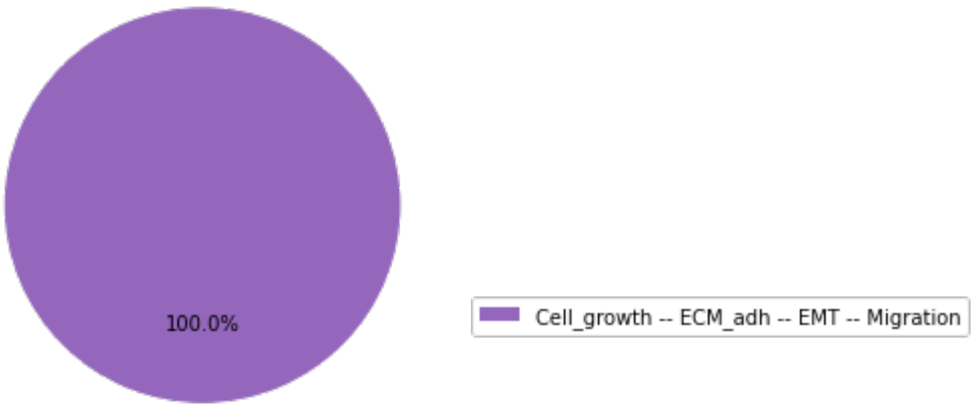

(('p21', 'OFF'), ('miR200', 'OFF'))

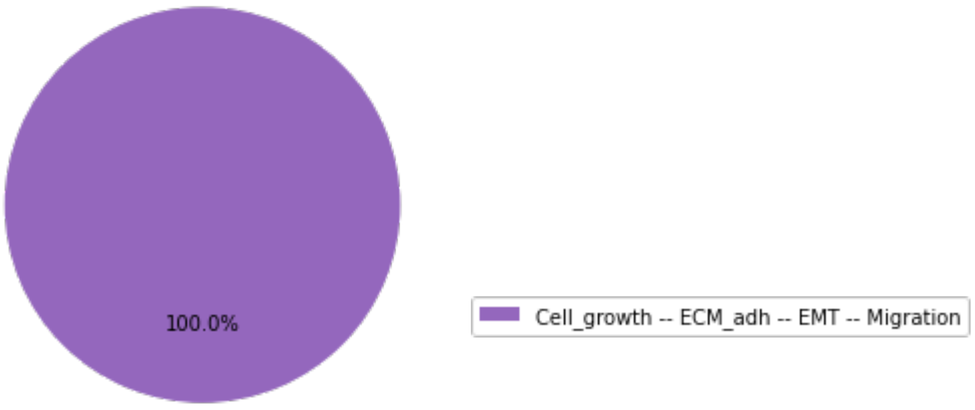

(('p21', 'OFF'), ('TGFbetaR', 'OFF'))

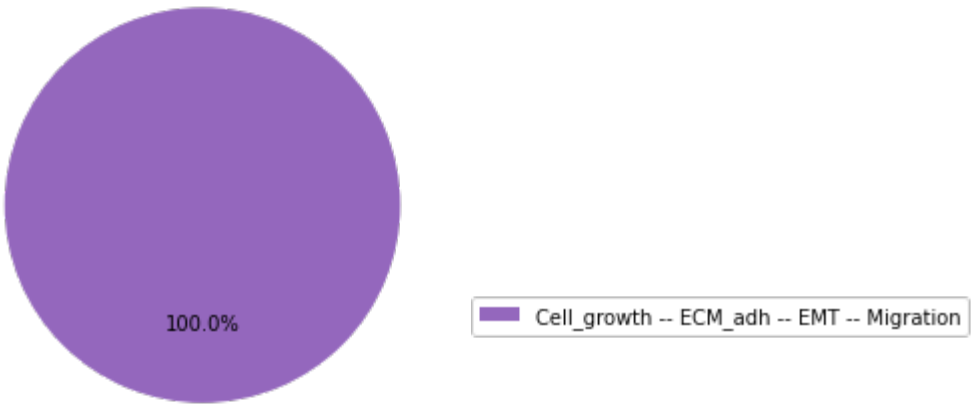

(('p21', 'OFF'), ('FAK', 'OFF'))

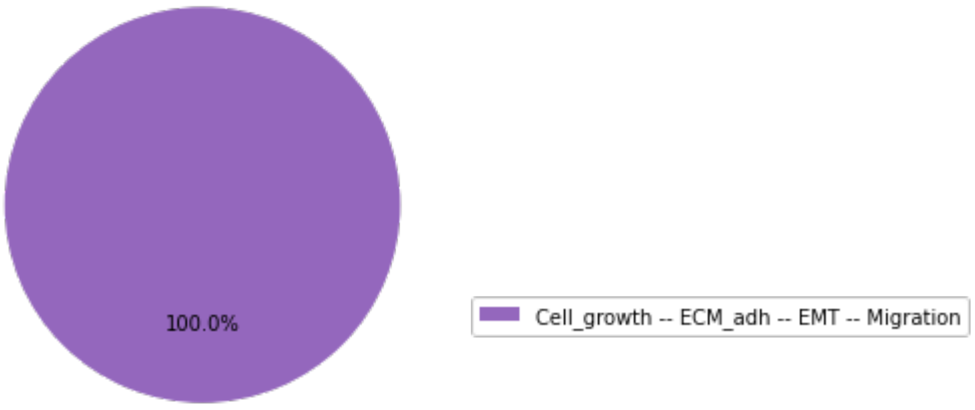

(('SMAD', 'OFF'), ('VIM', 'OFF'))

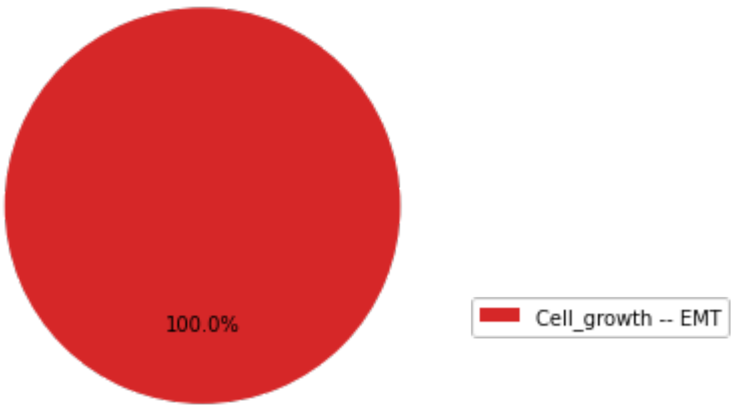

(('SMAD', 'OFF'), ('p53', 'OFF'))

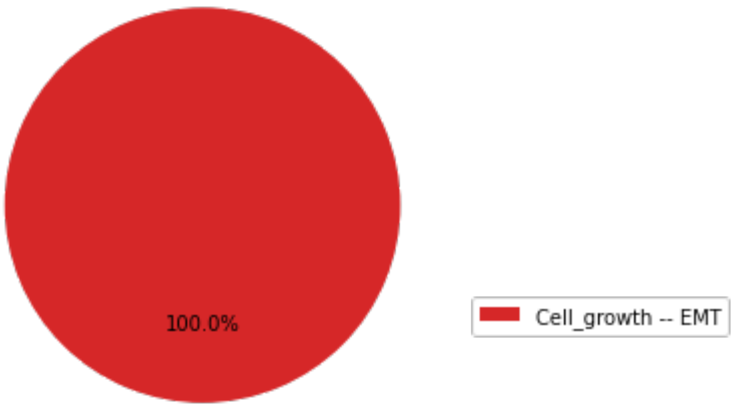

(('SMAD', 'OFF'), ('p63', 'OFF'))

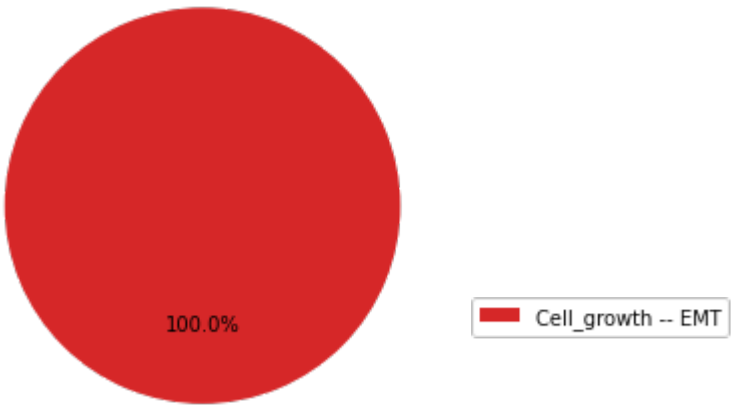

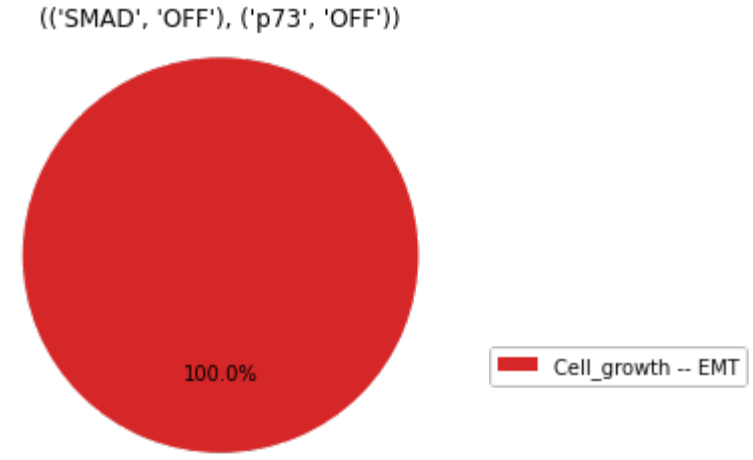
(('SMAD', 'OFF'), ('p73', 'OFF'))
100.0%
Cell_growth -- EMT

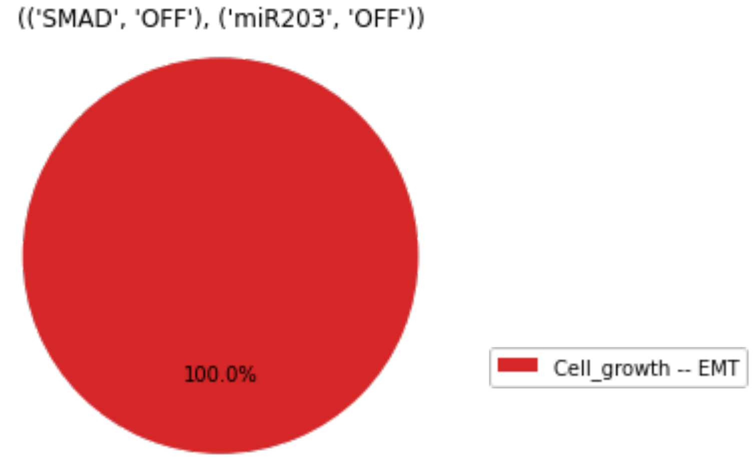
(('SMAD', 'OFF'), ('miR203', 'OFF'))
100.0%
Cell_growth -- EMT

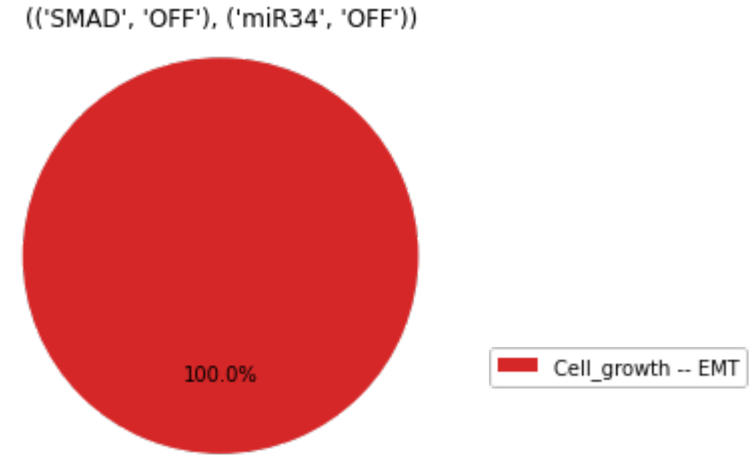
(('SMAD', 'OFF'), ('miR34', 'OFF'))
100.0%
Cell_growth -- EMT

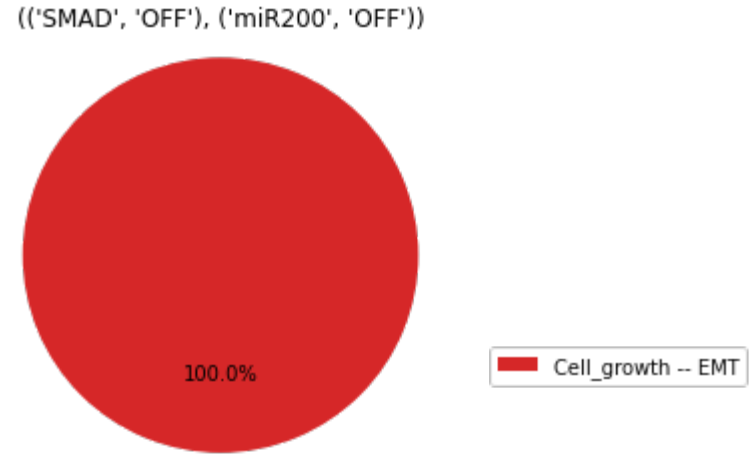
(('SMAD', 'OFF'), ('miR200', 'OFF'))
100.0%
Cell_growth -- EMT

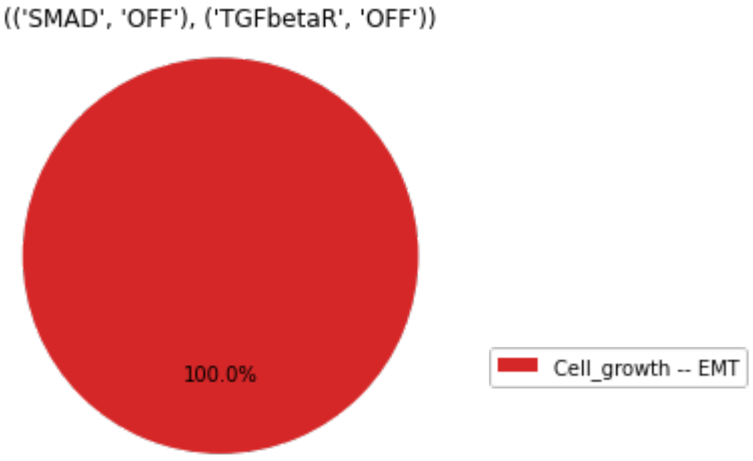
(('SMAD', 'OFF'), ('TGFbetaR', 'OFF'))
100.0%
Cell_growth -- EMT

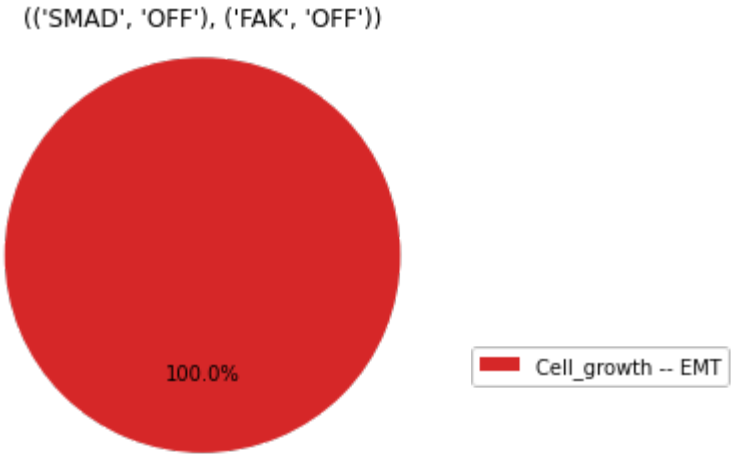
(('SMAD', 'OFF'), ('FAK', 'OFF'))
100.0%
Cell_growth -- EMT

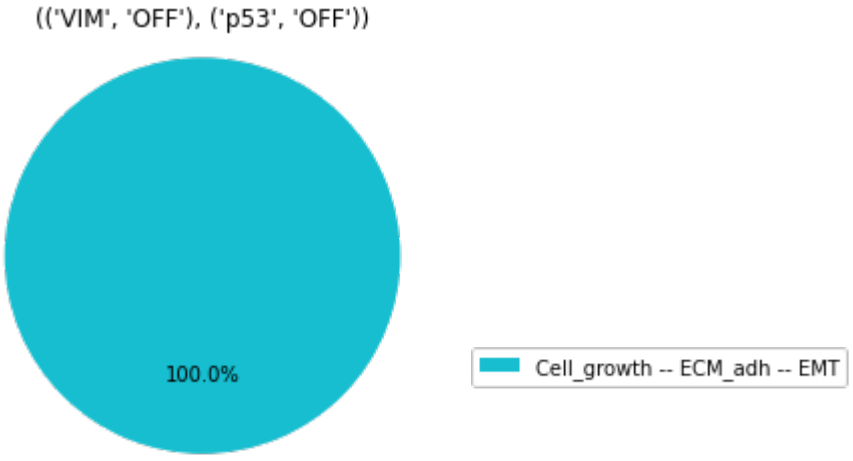
(('VIM', 'OFF'), ('p53', 'OFF'))
100.0%
Cell_growth -- ECM_adh -- EMT

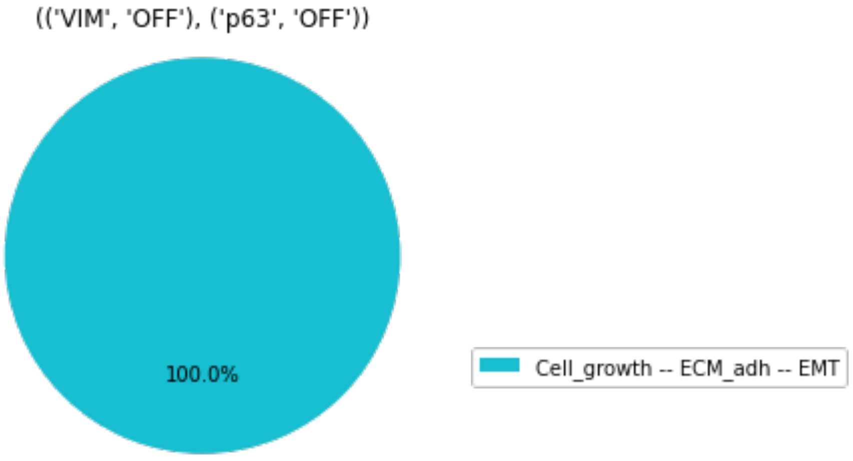
(('VIM', 'OFF'), ('p63', 'OFF'))
100.0%
Cell_growth -- ECM_adh -- EMT

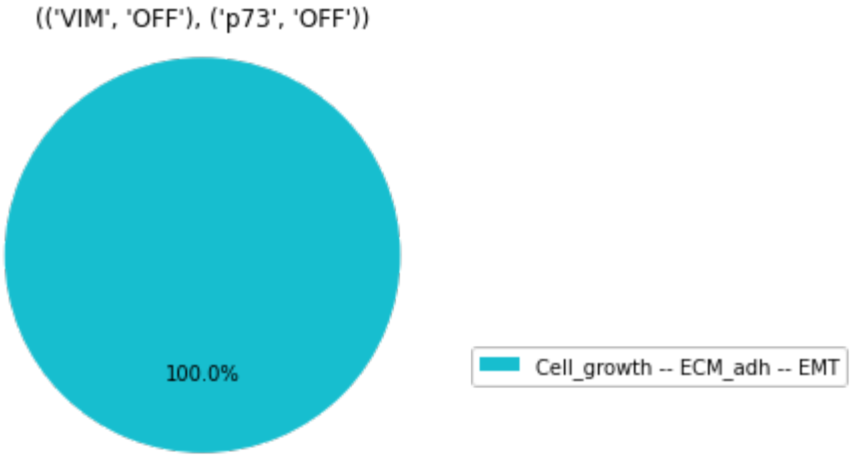
(('VIM', 'OFF'), ('p73', 'OFF'))
100.0%
Cell_growth -- ECM_adh -- EMT

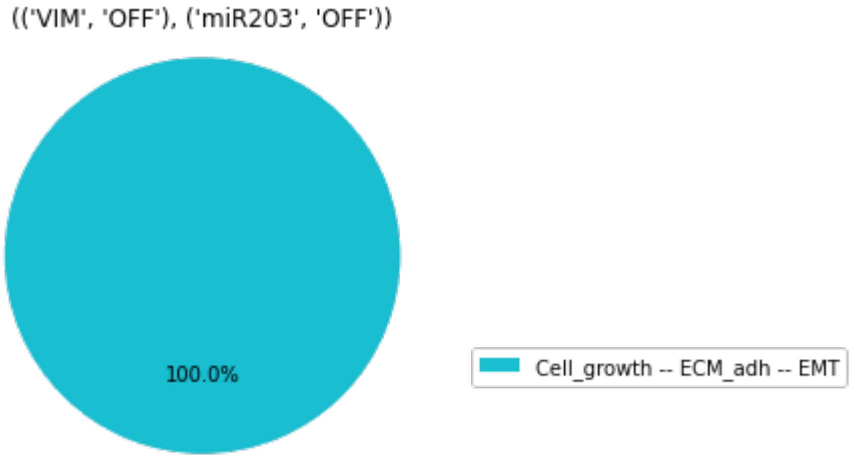
(('VIM', 'OFF'), ('miR203', 'OFF'))
100.0%
Cell_growth -- ECM_adh -- EMT

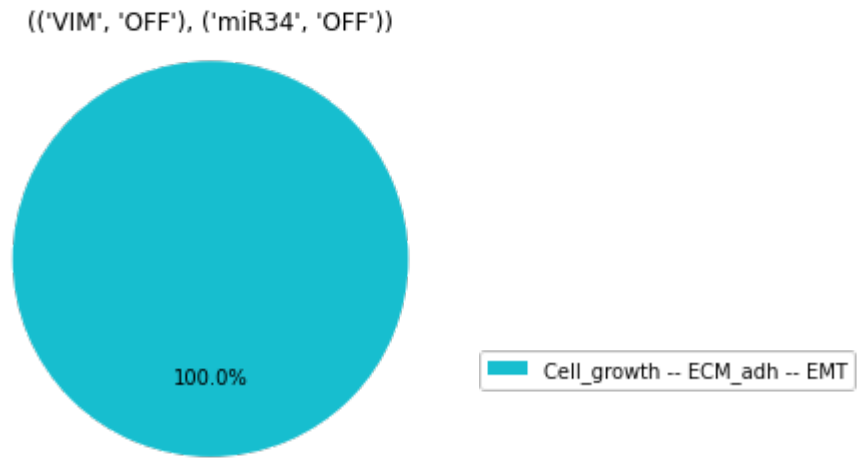
(('VIM', 'OFF'), ('miR34', 'OFF'))
100.0%
Cell_growth -- ECM_adh -- EMT

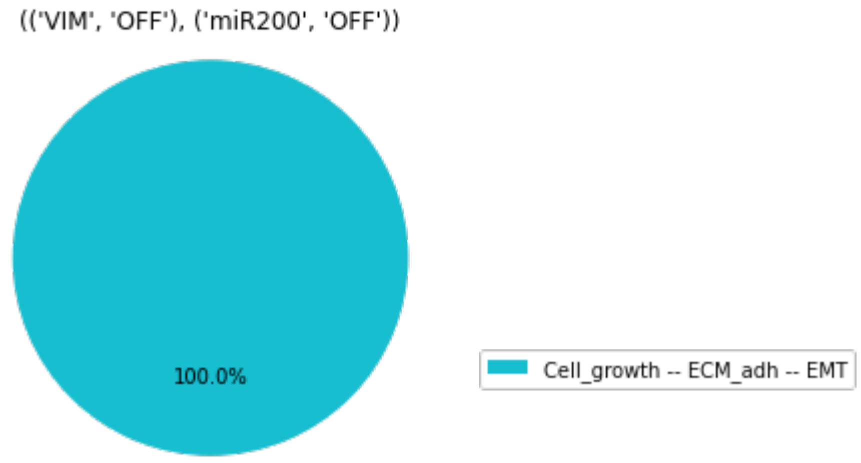
(('VIM', 'OFF'), ('miR200', 'OFF'))
100.0%
Cell_growth -- ECM_adh -- EMT

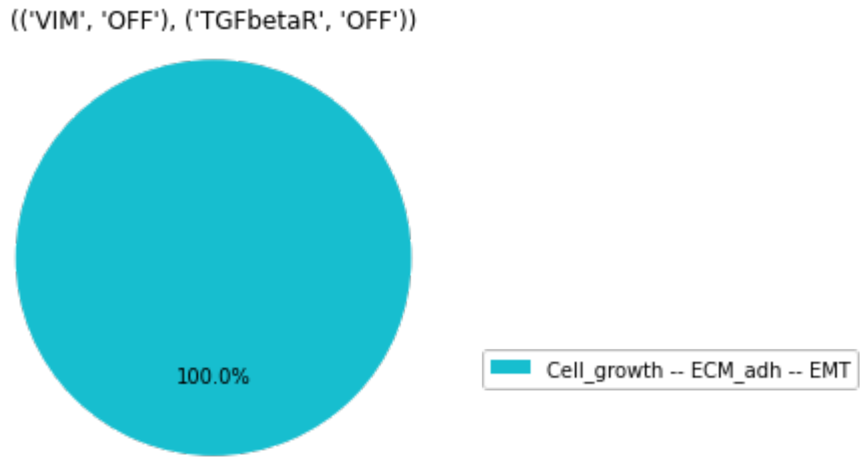
(('VIM', 'OFF'), ('TGFbetaR', 'OFF'))
100.0%
Cell_growth -- ECM_adh -- EMT

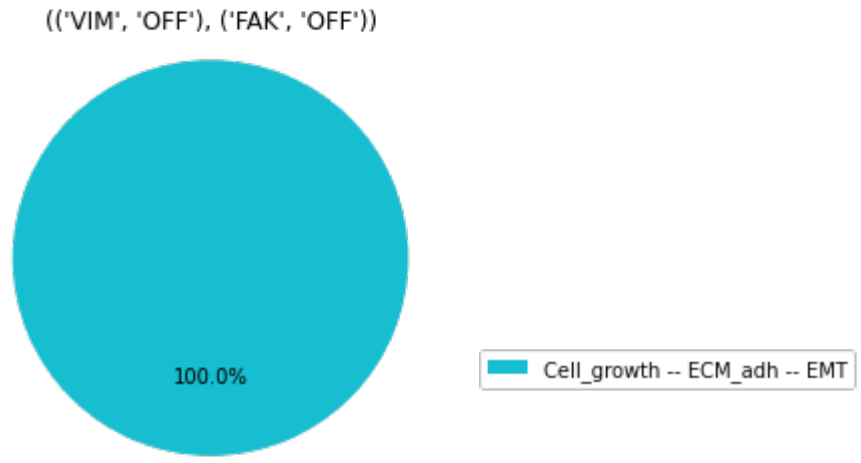
(('VIM', 'OFF'), ('FAK', 'OFF'))
100.0%
Cell_growth -- ECM_adh -- EMT

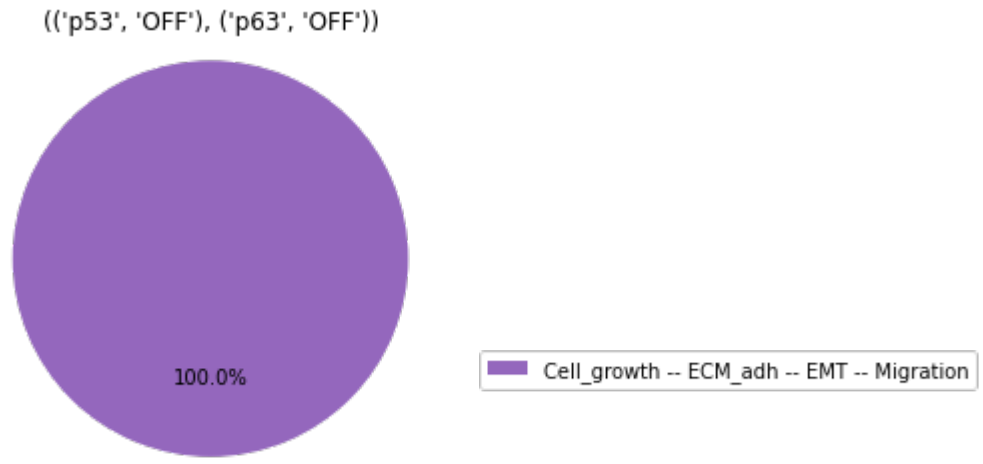
(('p53', 'OFF'), ('p63', 'OFF'))
100.0%
Cell_growth -- ECM_adh -- EMT -- Migration

(('p53', 'OFF'), ('p73', 'OFF'))

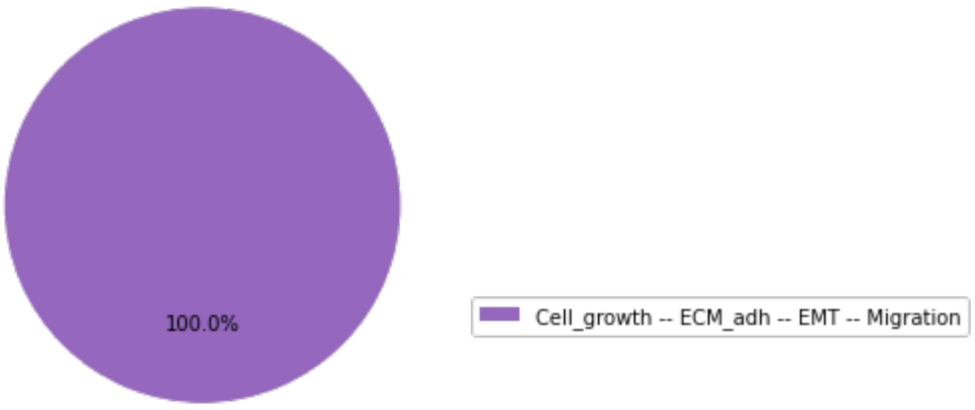

(('p53', 'OFF'), ('miR203', 'OFF'))

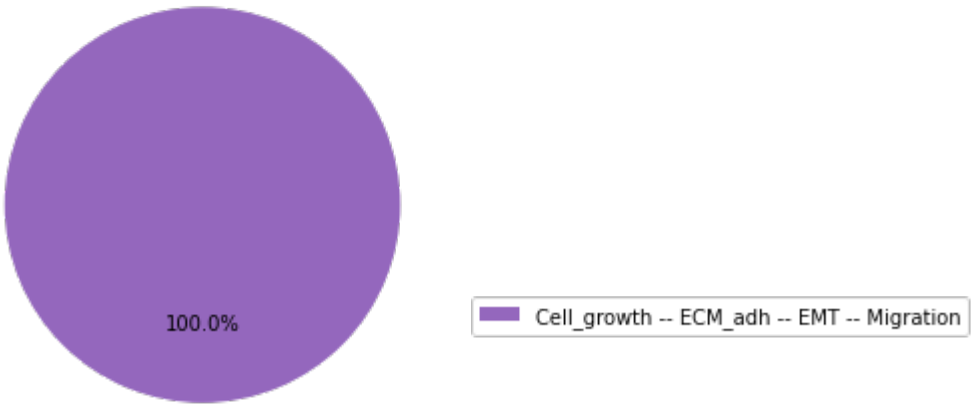

(('p53', 'OFF'), ('miR34', 'OFF'))

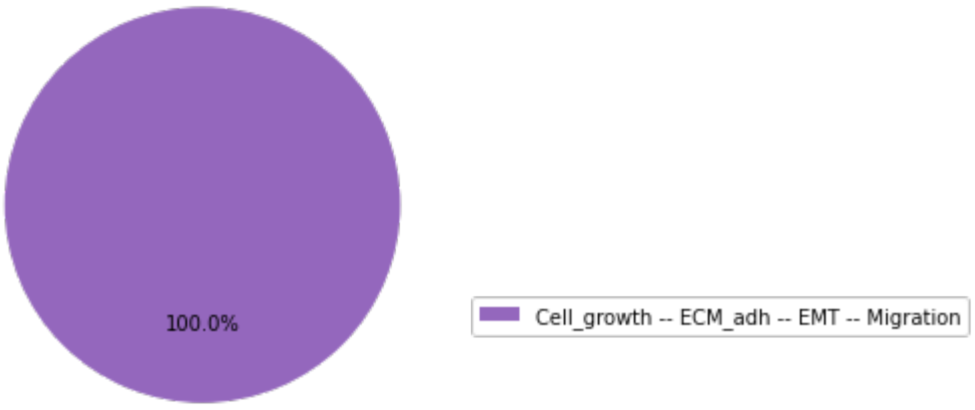

(('p53', 'OFF'), ('miR200', 'OFF'))

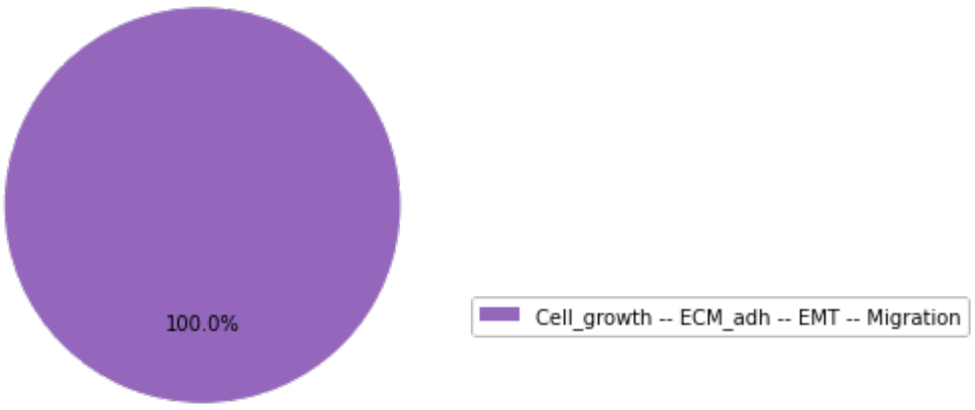

(('p53', 'OFF'), ('TGFbetaR', 'OFF'))

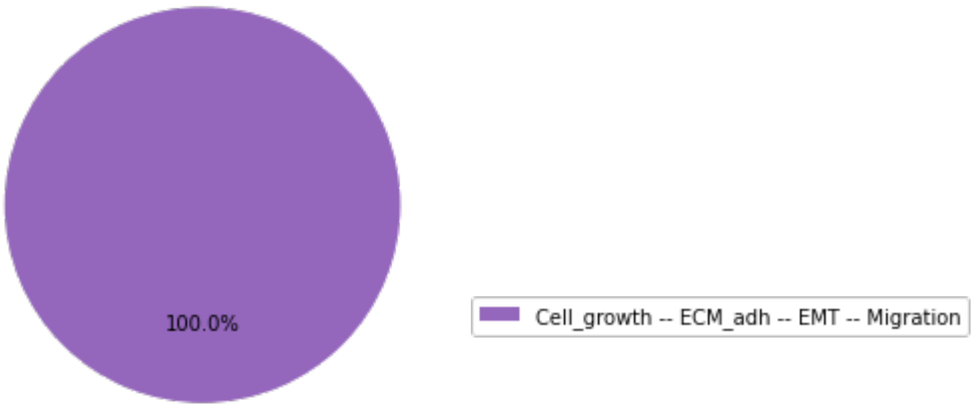

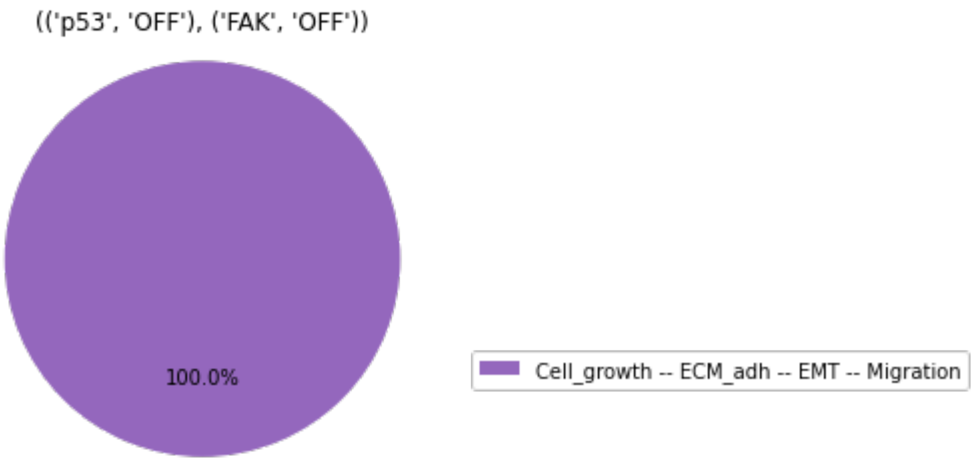
(('p53', 'OFF'), ('FAK', 'OFF'))
100.0%
Cell_growth -- ECM_adh -- EMT -- Migration

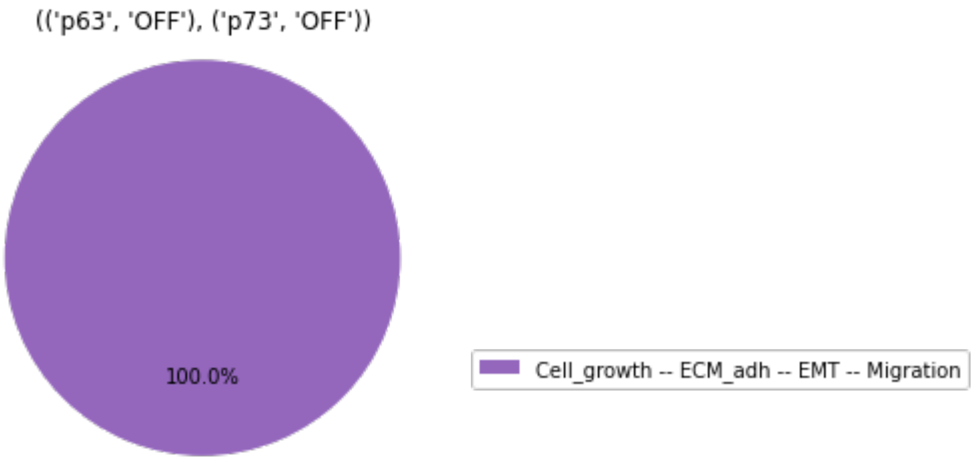
(('p63', 'OFF'), ('p73', 'OFF'))
100.0%
Cell_growth -- ECM_adh -- EMT -- Migration

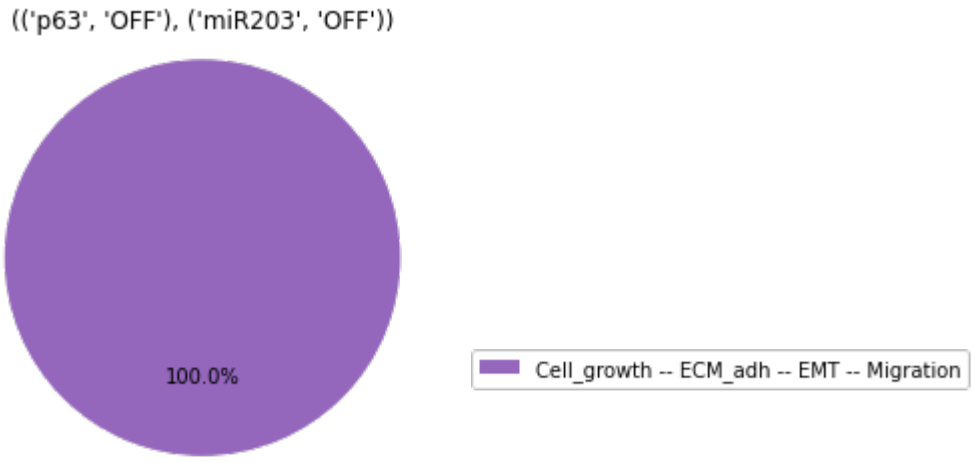
(('p63', 'OFF'), ('miR203', 'OFF'))
100.0%
Cell_growth -- ECM_adh -- EMT -- Migration

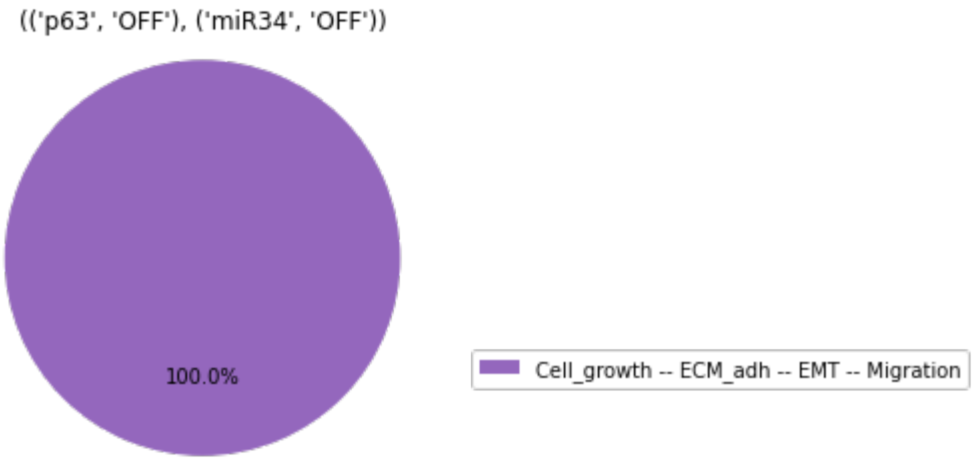
(('p63', 'OFF'), ('miR34', 'OFF'))
100.0%
Cell_growth -- ECM_adh -- EMT -- Migration

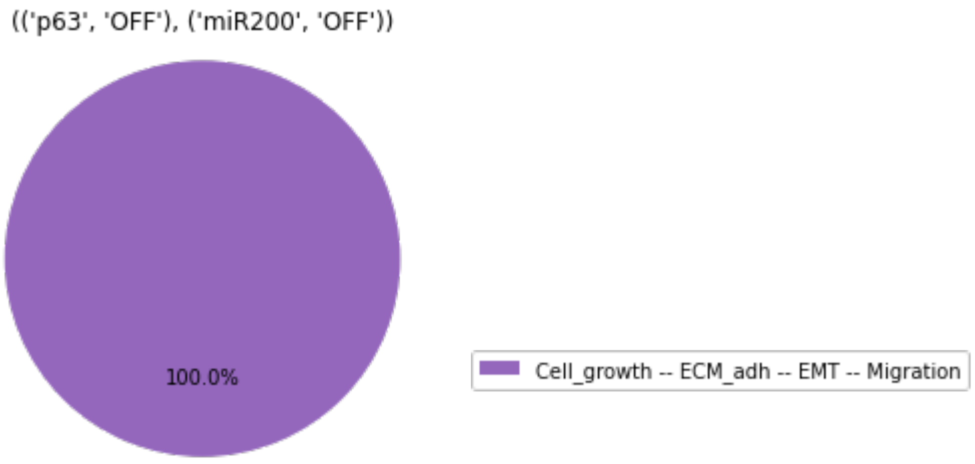
(('p63', 'OFF'), ('miR200', 'OFF'))
100.0%
Cell_growth -- ECM_adh -- EMT -- Migration

(('p63', 'OFF'), ('TGFbetaR', 'OFF'))

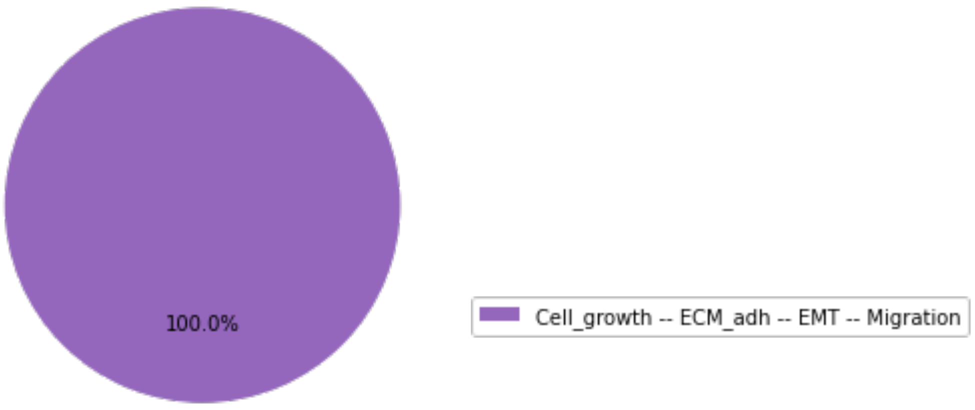

(('p63', 'OFF'), ('FAK', 'OFF'))

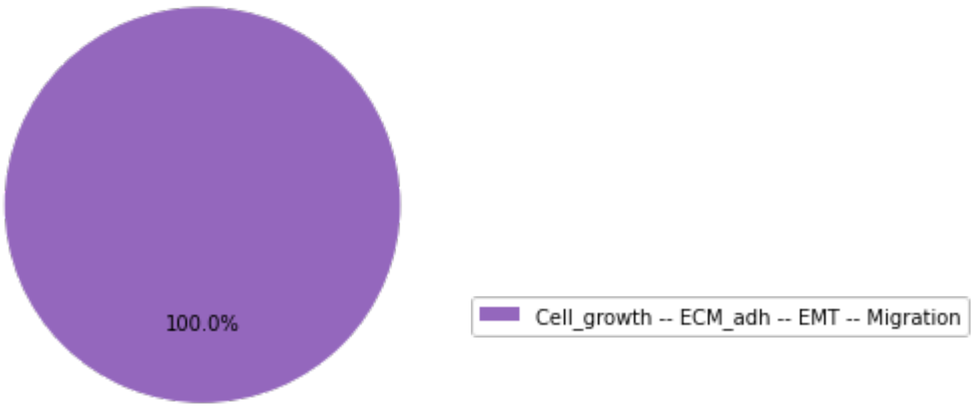

(('p73', 'OFF'), ('miR203', 'OFF'))

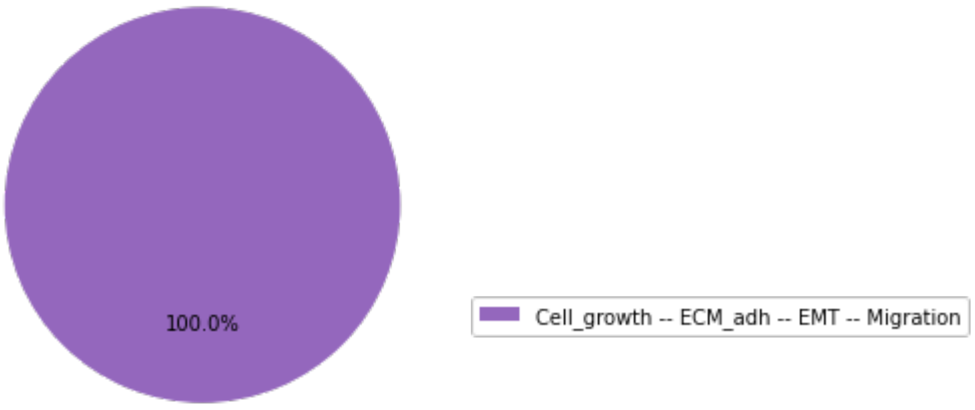

(('p73', 'OFF'), ('miR34', 'OFF'))

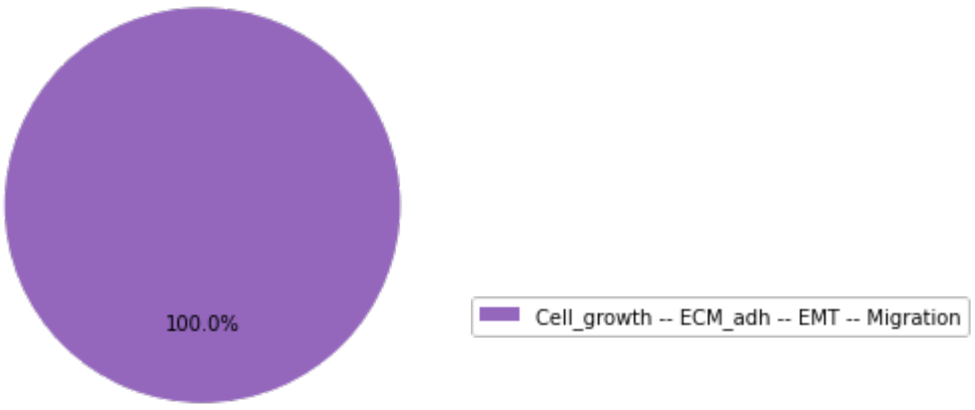

(('p73', 'OFF'), ('miR200', 'OFF'))

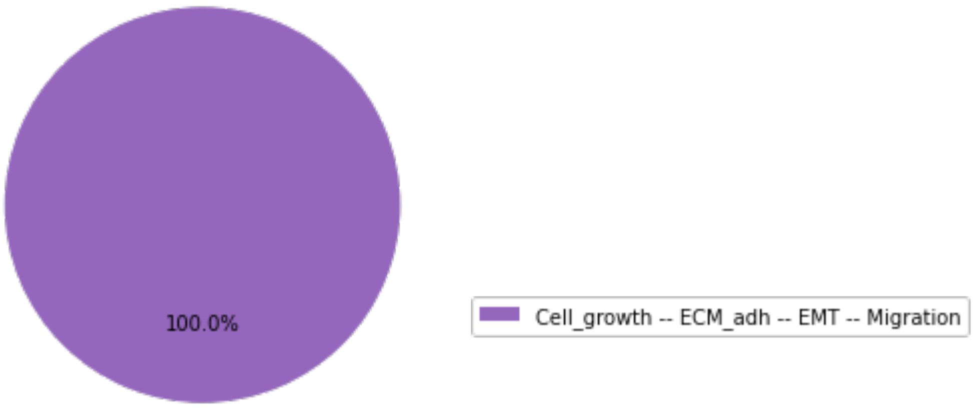

(('p73', 'OFF'), ('TGFbetaR', 'OFF'))

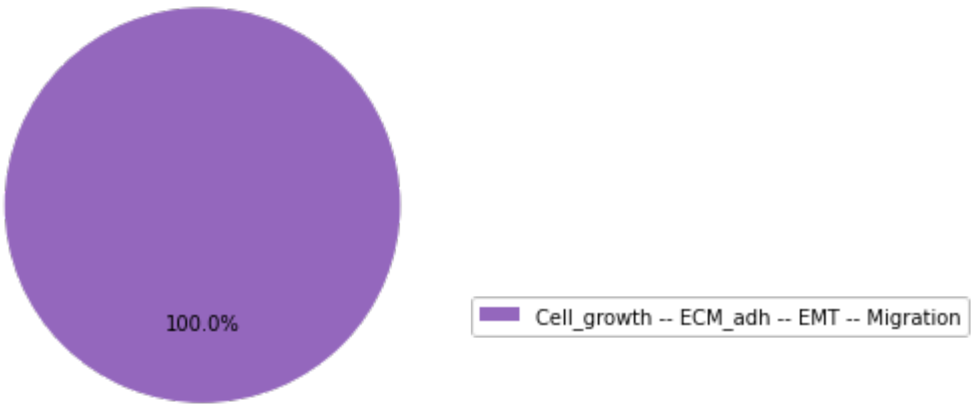

(('p73', 'OFF'), ('FAK', 'OFF'))

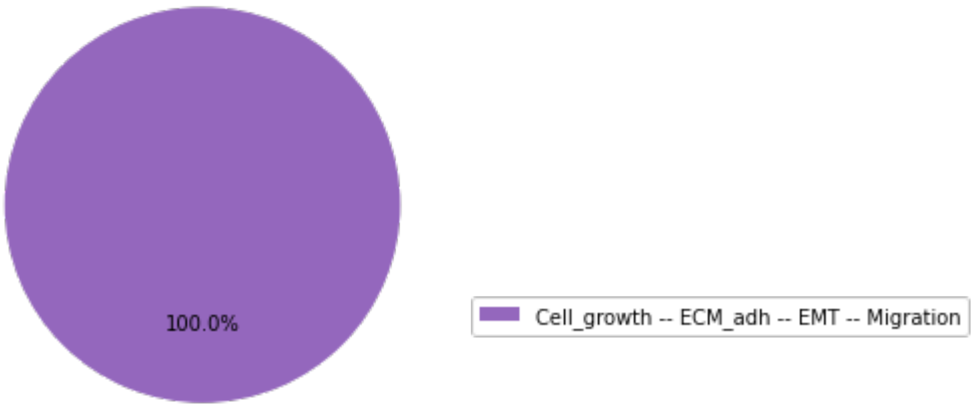

(('miR203', 'OFF'), ('miR34', 'OFF'))

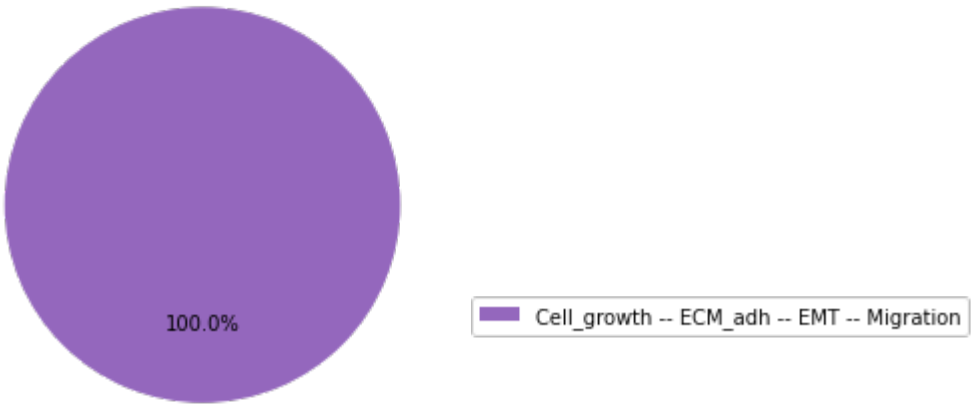

(('miR203', 'OFF'), ('miR200', 'OFF'))

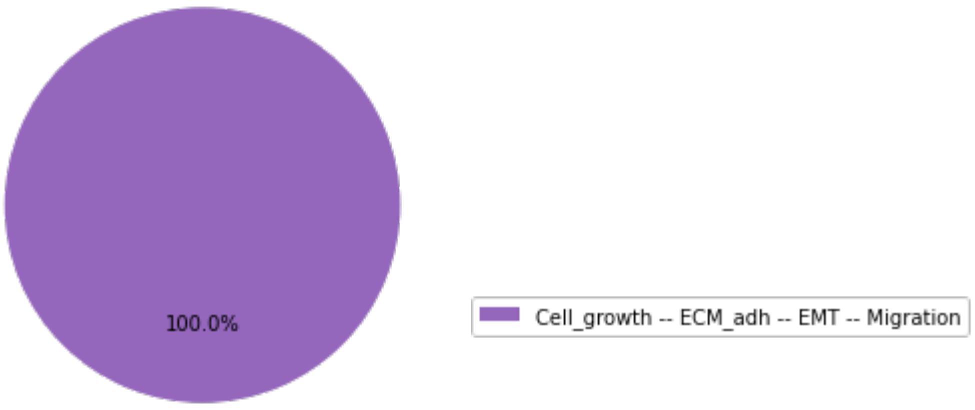

(('miR203', 'OFF'), ('TGFbetaR', 'OFF'))

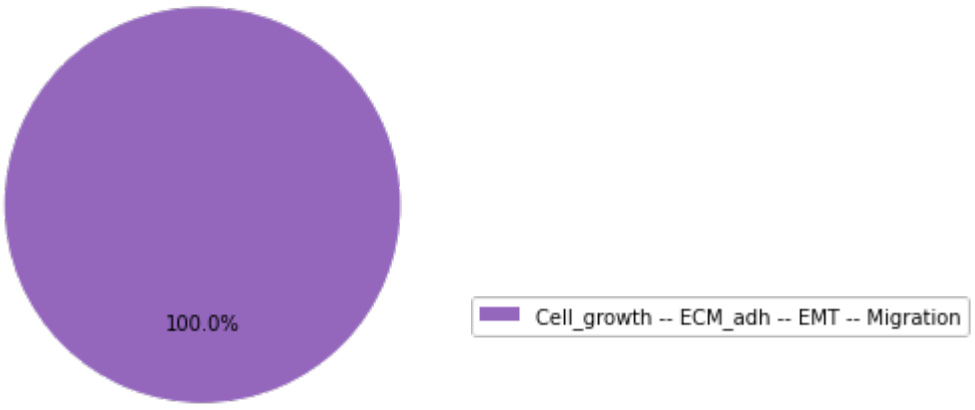

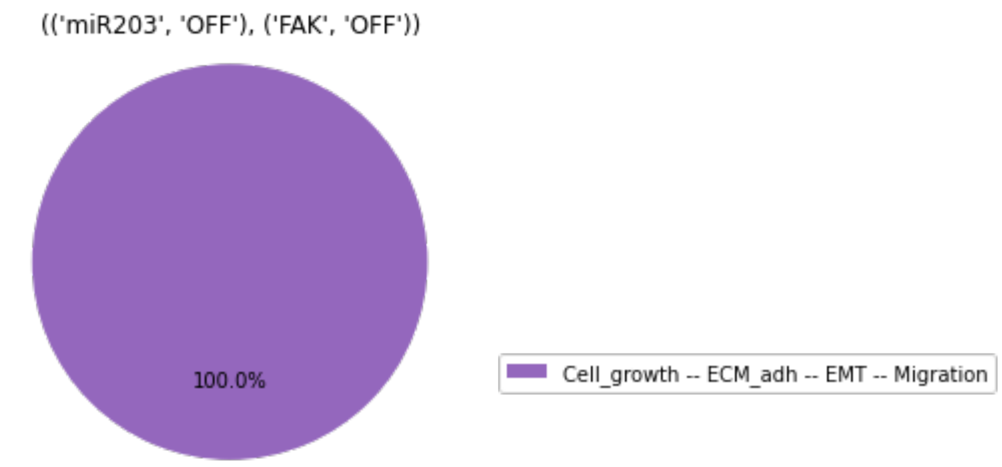
(('miR203', 'OFF'), ('FAK', 'OFF'))
100.0%
Cell_growth -- ECM_adh -- EMT -- Migration

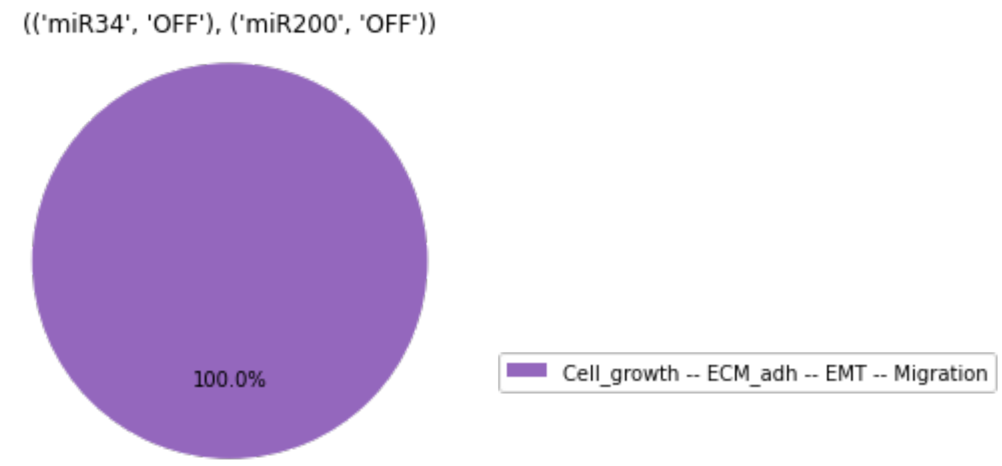
(('miR34', 'OFF'), ('miR200', 'OFF'))
100.0%
Cell_growth -- ECM_adh -- EMT -- Migration

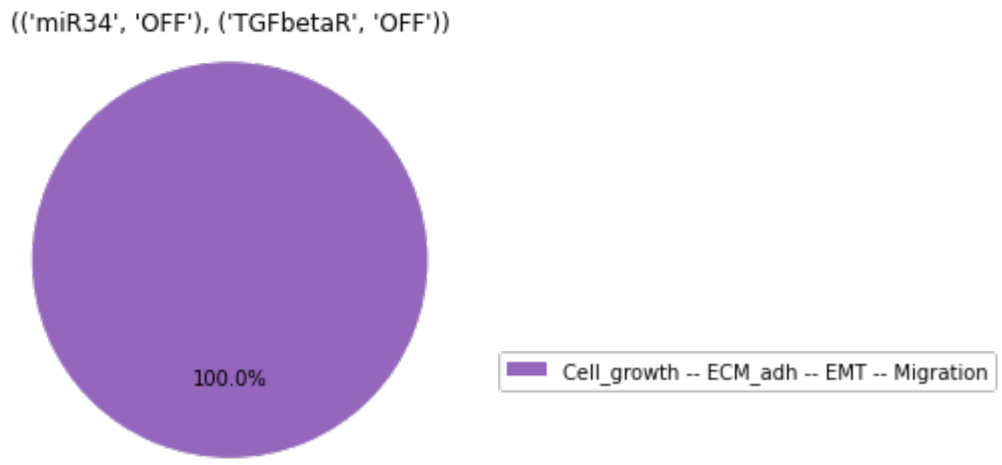
(('miR34', 'OFF'), ('TGFbetaR', 'OFF'))
100.0%
Cell_growth -- ECM_adh -- EMT -- Migration

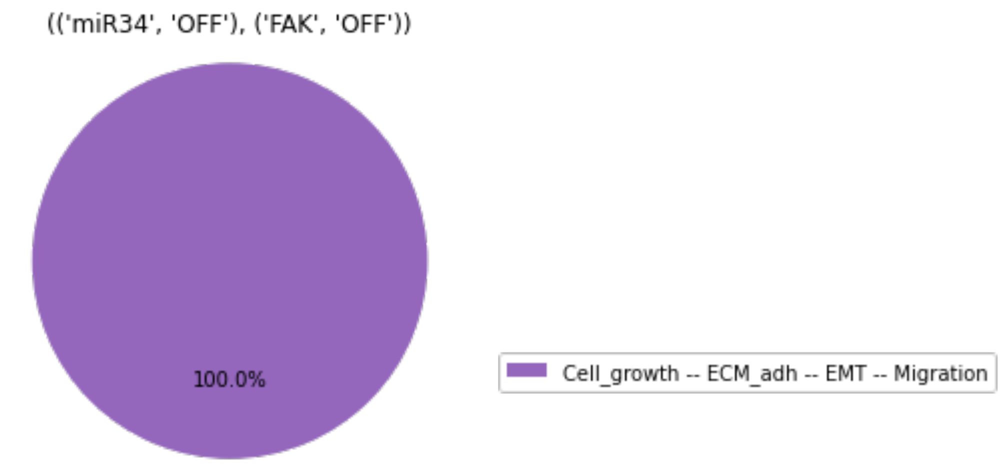
(('miR34', 'OFF'), ('FAK', 'OFF'))
100.0%
Cell_growth -- ECM_adh -- EMT -- Migration

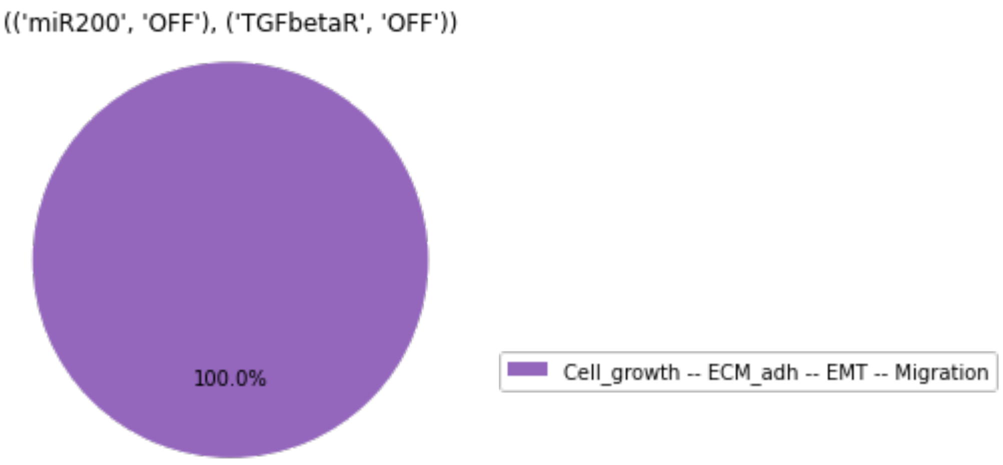
(('miR200', 'OFF'), ('TGFbetaR', 'OFF'))
100.0%
Cell_growth -- ECM_adh -- EMT -- Migration

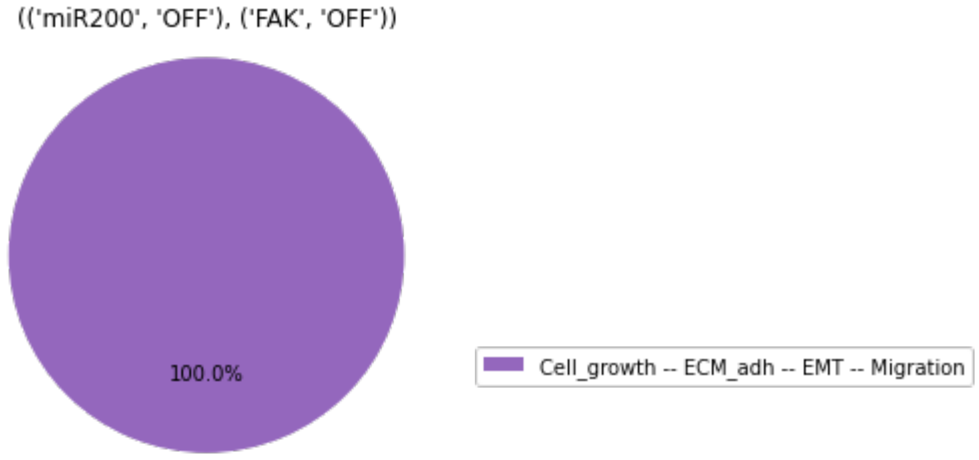

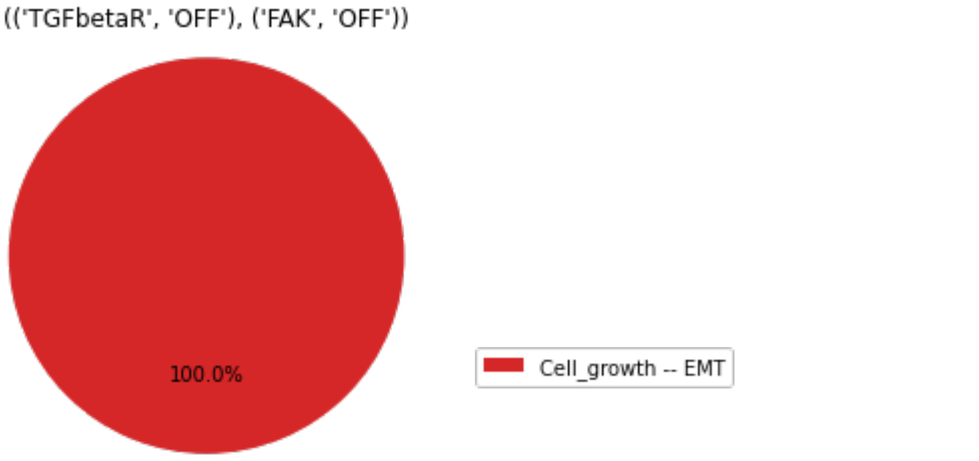

In [60]:
```
filtered_res = filter_sensititivy(res_single, node="ECM_degrad", maximum=0)
print(filtered_res.keys())
```

```
dict_keys([('AKT1', 'ON'), ('CTNNB1', 'ON'), ('DKK1', 'ON'), ('AKT2', 'ON'), ('RAC1', 'ON'), ('YAP1', 'ON'), ('PIK3CA', 'ON'), ('ERK', 'ON'), ('p21', 'ON'), ('SMAD', 'ON'), ('VIM', 'ON'), ('p53', 'ON'), ('p73', 'ON'), ('miR203', 'ON'), ('miR34', 'ON'), ('miR200', 'ON'), ('TGFbetaR', 'ON'), ('FAK', 'ON'), ('AKT1', 'OFF'), ('CTNNB1', 'OFF'), ('DKK1', 'OFF'), ('AKT2', 'OFF'), ('RAC1', 'OFF'), ('YAP1', 'OFF'), ('PIK3CA', 'OFF'), ('ERK', 'OFF'), ('p21', 'OFF'), ('SMAD', 'OFF'), ('VIM', 'OFF'), ('p53', 'OFF'), ('p63', 'OFF'), ('p73', 'OFF'), ('miR203', 'OFF'), ('miR34', 'OFF'), ('miR200', 'OFF'), ('TGFbetaR', 'OFF'), ('FAK', 'OFF')])
```

In [61]:
```
filtered_res1 = filter_sensititivy(res_single, node="EMT", maximum=0)
print(filtered_res1.keys())
```

```
dict_keys([('p53', 'ON'), ('miR34', 'ON')])
```

In [62]:
```
filtered_res2 = filter_sensititivy(res_single, node="Migration", maximum=0)
print(filtered_res2.keys())
```

```
dict_keys([('AKT1', 'ON'), ('CTNNB1', 'ON'), ('p53', 'ON'), ('p63', 'ON'), ('p73', 'ON'), ('miR203', 'ON'), ('miR34', 'ON'), ('miR200', 'ON'), ('AKT2', 'OFF'), ('ERK', 'OFF'), ('SMAD', 'OFF'), ('VIM', 'OFF')])
```

In [63]:
```
for elem in filtered_res:
    for elem2 in filtered_res1:
        for elem3 in filtered_res2:
            if elem == elem2 and elem2 == elem3:
                print(elem)
```

```
('p53', 'ON')
('miR34', 'ON')
```

Conclusion: among all of the possible mutations, in the perfect condition for proliferation, the common candidates to stop both MMPs release, Migration and EMT phenotypes are p53, p73 and miR34. Those are possible gene that can be overexpressed within the PhysiBoSS simulation, to see if effectively they block the invasion process.

In [64]:
```
filtered_res = filter_sensititivy(res_double, node="EMT", maximum=0)
print(filtered_res.keys())
```

```
dict_keys([(('AKT1', 'ON'), ('p53', 'ON')), (('DKK1', 'ON'), ('p53', 'ON')), (('DKK1', 'ON'), ('miR34', 'ON')), (('AKT2', 'ON'), ('p53', 'ON')), (('RAC1', 'ON'), ('p53', 'ON')), (('YAP1', 'ON'), ('p53', 'ON')), (('YAP1', 'ON'), ('miR34', 'ON')), (('PIK3CA', 'ON'), ('p53', 'ON')), (('PIK3CA', 'ON'), ('miR34', 'ON')), (('ERK', 'ON'), ('p53', 'ON')), (('ERK', 'ON'), ('miR34', 'ON')), (('p21', 'ON'), ('p53', 'ON')), (('SMAD', 'ON'), ('p53', 'ON')), (('SMAD', 'ON'), ('miR34', 'ON')), (('VIM', 'ON'), ('p53', 'ON')), (('VIM', 'ON'), ('miR34', 'ON')), (('p53', 'ON'), ('p63', 'ON')), (('p53', 'ON'), ('p73', 'ON')), (('p53', 'ON'), ('miR203', 'ON')), (('p53', 'ON'), ('miR34', 'ON')), (('p53', 'ON'), ('miR200', 'ON')), (('p53', 'ON'), ('TGFbetaR', 'ON')), (('p53', 'ON'), ('AKT1', 'OFF')), (('p53', 'ON'), ('CTNNB1', 'OFF')), (('p53', 'ON'), ('DKK1', 'OFF')), (('p53', 'ON'), ('AKT2', 'OFF')), (('p53', 'ON'), ('RAC1', 'OFF')), (('p53', 'ON'), ('YAP1', 'OFF')), (('p53', 'ON'), ('PIK3CA', 'OFF')), (('p53', 'ON'), ('ERK', 'OFF')), (('p53', 'ON'), ('p21', 'OFF')), (('p53', 'ON'), ('SMAD', 'OFF')), (('p53', 'ON'), ('VIM', 'OFF')), (('p53', 'ON'), ('p63', 'OFF')), (('p53', 'ON'), ('p73', 'OFF')), (('p53', 'ON'), ('miR203', 'OFF')), (('p53', 'ON'), ('miR34', 'OFF')), (('p53', 'ON'), ('miR200', 'OFF')), (('p53', 'ON'), ('TGFbetaR', 'OFF')), (('p53', 'ON'), ('FAK', 'OFF')), (('p63', 'ON'), ('FAK', 'OFF')), (('p73', 'ON'), ('FAK', 'OFF')), (('miR34', 'ON'), ('AKT1', 'OFF')), (('miR34', 'ON'), ('RAC1', 'OFF')), (('miR34', 'ON'), ('YAP1', 'OFF')), (('miR34', 'ON'), ('PIK3CA', 'OFF')), (('miR34', 'ON'), ('ERK', 'OFF')), (('miR34', 'ON'), ('p21', 'OFF')), (('miR34', 'ON'), ('SMAD', 'OFF')), (('miR34', 'ON'), ('p63', 'OFF')), (('miR34', 'ON'), ('p73', 'OFF')), (('miR34', 'ON'), ('miR200', 'OFF')), (('miR34', 'ON'), ('FAK', 'OFF')), (('miR200', 'ON'), ('FAK', 'OFF'))])
```

In [65]:
```
filtered_res1 = filter_sensititivy(res_double, node="ECM_degrad", maximum=0)
print(filtered_res.keys())
```

```
dict_keys([(('AKT1', 'ON'), ('p53', 'ON')), (('DKK1', 'ON'), ('p53', 'ON')), (('DKK1', 'ON'), ('miR34', 'ON')), (('AKT2', 'ON'), ('p53', 'ON')), (('RAC1', 'ON'), ('p53', 'ON')), (('YAP1', 'ON'), ('p53', 'ON')), (('YAP1', 'ON'), ('miR34', 'ON')), (('PIK3CA', 'ON'), ('p53', 'ON')), (('PIK3CA', 'ON'), ('miR34', 'ON')), (('ERK', 'ON'), ('p53', 'ON')), (('ERK', 'ON'), ('miR34', 'ON')), (('p21', 'ON'), ('p53', 'ON')), (('SMAD', 'ON'), ('p53', 'ON')), (('SMAD', 'ON'), ('miR34', 'ON')), (('VIM', 'ON'), ('p53', 'ON')), (('VIM', 'ON'), ('miR34', 'ON')), (('p53', 'ON'), ('p63', 'ON')), (('p53', 'ON'), ('p73', 'ON')), (('p53', 'ON'), ('miR203', 'ON')), (('p53', 'ON'), ('miR34', 'ON')), (('p53', 'ON'), ('miR200', 'ON')), (('p53', 'ON'), ('TGFbetaR', 'ON')), (('p53', 'ON'), ('AKT1', 'OFF')), (('p53', 'ON'), ('CTNNB1', 'OFF')), (('p53', 'ON'), ('DKK1', 'OFF')), (('p53', 'ON'), ('AKT2', 'OFF')), (('p53', 'ON'), ('RAC1', 'OFF')), (('p53', 'ON'), ('YAP1', 'OFF')), (('p53', 'ON'), ('PIK3CA', 'OFF')), (('p53', 'ON'), ('ERK', 'OFF')), (('p53', 'ON'), ('p21', 'OFF')), (('p53', 'ON'), ('SMAD', 'OFF')), (('p53', 'ON'), ('VIM', 'OFF')), (('p53', 'ON'), ('p63', 'OFF')), (('p53', 'ON'), ('p73', 'OFF')), (('p53', 'ON'), ('miR203', 'OFF')), (('p53', 'ON'), ('miR34', 'OFF')), (('p53', 'ON'), ('miR200', 'OFF')), (('p53', 'ON'), ('TGFbetaR', 'OFF')), (('p53', 'ON'), ('FAK', 'OFF')), (('p63', 'ON'), ('FAK', 'OFF')), (('p73', 'ON'), ('FAK', 'OFF')), (('miR34', 'ON'), ('AKT1', 'OFF')), (('miR34', 'ON'), ('RAC1', 'OFF')), (('miR34', 'ON'), ('YAP1', 'OFF')), (('miR34', 'ON'), ('PIK3CA', 'OFF')), (('miR34', 'ON'), ('ERK', 'OFF')), (('miR34', 'ON'), ('p21', 'OFF')), (('miR34', 'ON'), ('SMAD', 'OFF')), (('miR34', 'ON'), ('p63', 'OFF')), (('miR34', 'ON'), ('p73', 'OFF')), (('miR34', 'ON'), ('miR200', 'OFF')), (('miR34', 'ON'), ('FAK', 'OFF')), (('miR200', 'ON'), ('FAK', 'OFF'))])
```

In [66]:
```
filtered_res2 = filter_sensititivy(res_double, node="Migration", maximum=0)
print(filtered_res.keys())
```

```
dict_keys([(('AKT1', 'ON'), ('p53', 'ON')), (('DKK1', 'ON'), ('p53', 'ON')), (('DKK1', 'ON'), ('miR34', 'ON')), (('AKT2', 'ON'), ('p53', 'ON')), (('RAC1', 'ON'), ('p53', 'ON')), (('YAP1', 'ON'), ('p53', 'ON')), (('YAP1', 'ON'), ('miR34', 'ON')), (('PIK3CA', 'ON'), ('p53', 'ON')), (('PIK3CA', 'ON'), ('miR34', 'ON')), (('ERK', 'ON'), ('p53', 'ON')), (('ERK', 'ON'), ('miR34', 'ON')), (('p21', 'ON'), ('p53', 'ON')), (('SMAD', 'ON'), ('p53', 'ON')), (('SMAD', 'ON'), ('miR34', 'ON')), (('VIM', 'ON'), ('p53', 'ON')), (('VIM', 'ON'), ('miR34', 'ON')), (('p53', 'ON'), ('p63', 'ON')), (('p53', 'ON'), ('p73', 'ON')), (('p53', 'ON'), ('miR203', 'ON')), (('p53', 'ON'), ('miR34', 'ON')), (('p53', 'ON'), ('miR200', 'ON')), (('p53', 'ON'), ('TGFbetaR', 'ON')), (('p53', 'ON'), ('AKT1', 'OFF')), (('p53', 'ON'), ('CTNNB1', 'OFF')), (('p53', 'ON'), ('DKK1', 'OFF')), (('p53', 'ON'), ('AKT2', 'OFF')), (('p53', 'ON'), ('RAC1', 'OFF')), (('p53', 'ON'), ('YAP1', 'OFF')), (('p53', 'ON'), ('PIK3CA', 'OFF')), (('p53', 'ON'), ('ERK', 'OFF')), (('p53', 'ON'), ('p21', 'OFF')), (('p53', 'ON'), ('SMAD', 'OFF')), (('p53', 'ON'), ('VIM', 'OFF')), (('p53', 'ON'), ('p63', 'OFF')), (('p53', 'ON'), ('p73', 'OFF')), (('p53', 'ON'), ('miR203', 'OFF')), (('p53', 'ON'), ('miR34', 'OFF')), (('p53', 'ON'), ('miR200', 'OFF')), (('p53', 'ON'), ('TGFbetaR', 'OFF')), (('p53', 'ON'), ('FAK', 'OFF')), (('p63', 'ON'), ('FAK', 'OFF')), (('p73', 'ON'), ('FAK', 'OFF')), (('miR34', 'ON'), ('AKT1', 'OFF')), (('miR34', 'ON'), ('RAC1', 'OFF')), (('miR34', 'ON'), ('YAP1', 'OFF')), (('miR34', 'ON'), ('PIK3CA', 'OFF')), (('miR34', 'ON'), ('ERK', 'OFF')), (('miR34', 'ON'), ('p21', 'OFF')), (('miR34', 'ON'), ('SMAD', 'OFF')), (('miR34', 'ON'), ('p63', 'OFF')), (('miR34', 'ON'), ('p73', 'OFF')), (('miR34', 'ON'), ('miR200', 'OFF')), (('miR34', 'ON'), ('FAK', 'OFF')), (('miR200', 'ON'), ('FAK', 'OFF'))])
```

In [67]:
```
filtered_res1 = filter_sensititivy(res_double, node="Cell_growth", maximum=0)
print(filtered_res.keys())
```

```
dict_keys([(('AKT1', 'ON'), ('p53', 'ON')), (('DKK1', 'ON'), ('p53', 'ON')), (('DKK1', 'ON'), ('miR34', 'ON')), (('AKT2', 'ON'), ('p53', 'ON')), (('RAC1', 'ON'), ('p53', 'ON')), (('YAP1', 'ON'), ('p53', 'ON')), (('YAP1', 'ON'), ('miR34', 'ON')), (('PIK3CA', 'ON'), ('p53', 'ON')), (('PIK3CA', 'ON'), ('miR34', 'ON')), (('ERK', 'ON'), ('p53', 'ON')), (('ERK', 'ON'), ('miR34', 'ON')), (('p21', 'ON'), ('p53', 'ON')), (('SMAD', 'ON'), ('p53', 'ON')), (('SMAD', 'ON'), ('miR34', 'ON')), (('VIM', 'ON'), ('p53', 'ON')), (('VIM', 'ON'), ('miR34', 'ON')), (('p53', 'ON'), ('p63', 'ON')), (('p53', 'ON'), ('p73', 'ON')), (('p53', 'ON'), ('miR203', 'ON')), (('p53', 'ON'), ('miR34', 'ON')), (('p53', 'ON'), ('miR200', 'ON')), (('p53', 'ON'), ('TGFbetaR', 'ON')), (('p53', 'ON'), ('AKT1', 'OFF')), (('p53', 'ON'), ('CTNNB1', 'OFF')), (('p53', 'ON'), ('DKK1', 'OFF')), (('p53', 'ON'), ('AKT2', 'OFF')), (('p53', 'ON'), ('RAC1', 'OFF')), (('p53', 'ON'), ('YAP1', 'OFF')), (('p53', 'ON'), ('PIK3CA', 'OFF')), (('p53', 'ON'), ('ERK', 'OFF')), (('p53', 'ON'), ('p21', 'OFF')), (('p53', 'ON'), ('SMAD', 'OFF')), (('p53', 'ON'), ('VIM', 'OFF')), (('p53', 'ON'), ('p63', 'OFF')), (('p53', 'ON'), ('p73', 'OFF')), (('p53', 'ON'), ('miR203', 'OFF')), (('p53', 'ON'), ('miR34', 'OFF')), (('p53', 'ON'), ('miR200', 'OFF')), (('p53', 'ON'), ('TGFbetaR', 'OFF')), (('p53', 'ON'), ('FAK', 'OFF')), (('p63', 'ON'), ('FAK', 'OFF')), (('p73', 'ON'), ('FAK', 'OFF')), (('miR34', 'ON'), ('AKT1', 'OFF')), (('miR34', 'ON'), ('RAC1', 'OFF')), (('miR34', 'ON'), ('YAP1', 'OFF')), (('miR34', 'ON'), ('PIK3CA', 'OFF')), (('miR34', 'ON'), ('ERK', 'OFF')), (('miR34', 'ON'), ('p21', 'OFF')), (('miR34', 'ON'), ('SMAD', 'OFF')), (('miR34', 'ON'), ('p63', 'OFF')), (('miR34', 'ON'), ('p73', 'OFF')), (('miR34', 'ON'), ('miR200', 'OFF')), (('miR34', 'ON'), ('FAK', 'OFF')), (('miR200', 'ON'), ('FAK', 'OFF'))])
```

In [68]:
```
for elem in filtered_res:
    for elem2 in filtered_res1:
        for elem3 in filtered_res2:
            if elem == elem2 and elem2 == elem3:
                print(elem)
```

```
(('AKT1', 'ON'), ('p53', 'ON'))
(('p21', 'ON'), ('p53', 'ON'))
(('p53', 'ON'), ('ERK', 'OFF'))
(('miR34', 'ON'), ('ERK', 'OFF'))
```

Conclusion: among the possible double mutations, the ones shown in the previous cell, can block both the differentiation of epithelial cells into mesenchymal, and inhibit the growth of the tumor. Thus, these are possible candidates to simulate on PhysiBoSS, to check if they effectively stop the tumor development.
