# Supplementary material for: Multiscale model of the different modes of cancer cell invasion
Source: Bioinformatics. 2023 Jun 8;39(6):btad374. doi: 10.1093/bioinformatics/btad374 (PMC10293590; doi:10.1093/bioinformatics/btad374)
Supplement: btad374_Supplementary_Data [file btad374_supplementary_data.zip › Supplementary Information.pdf]

Supplementary Information

****

Multiscale modeling allows to study the different modes of cancer cell invasion

Marco Ruscone[1,2,3,*], Arnau Montagud[5], Philippe Chavrier[6], Olivier Destaing[7], Isabelle Bonnet[8], Andrei Zinovyev[1,2,3], Emmanuel Barillot[1,2,3], Vincent Noel[1,2,3,†] and Laurence Calzone[1,2,3,*,†]

Outline

1. Connecting the agent-based model to the Boolean Model
2. The intracellular model - Logical formulae
3. Simulation of initial conditions with MaBoSS
4. Modeling with PhysiBoSS
5. EMT activation - molecular players
6. Cell Cycle model in PhysiBoSS
7. Experimental data used to validate the Boolean model
8. List of parameters of the model
9. Lodillinksy results on intracellular model and ERK scenario
10. In silico experiments and predictions
11. Simulation of local light-activation of the SRC oncoprotein in an epithelial monolayer promotes collective extrusion - 3D scenario and ERK knock-out
12. Cell adhesion and density changes in the ECM regulate modes of invasion
13. Quantification of the migration modes
14. Computational complexity of the simulation
15. Sensitivity analysis on model parameters
a. Bootstrap analysis
b. Sensitivity analysis
16. Bibliography

1. Connecting the agent-based model to the Boolean Model

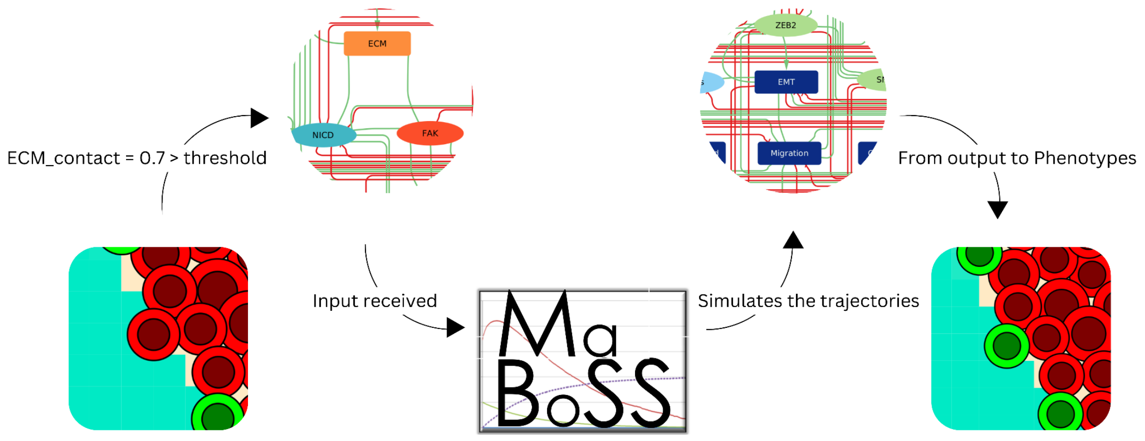

Figure S1. Connection between the agent-based model and the cellular model.

In Figure S1, we describe the links between the variables of the agent-based model (ABM) model and the Boolean model (BM).

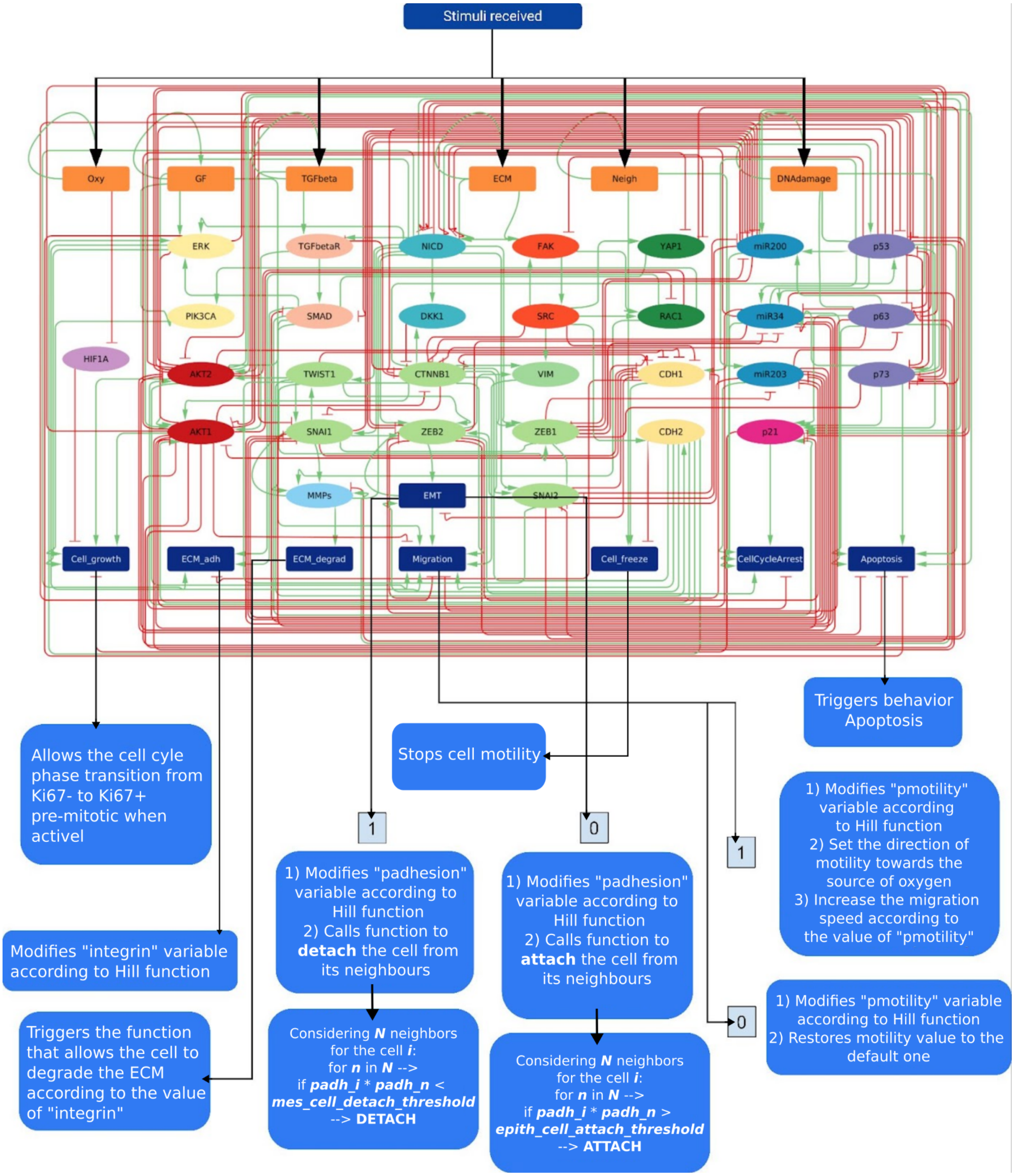

Figure S2: *Scheme of the links between the intracellular model and the behaviors of the agents. The blue boxes at the bottom described more in detail the implementation of the cell behavior in c++.*

2. The intracellular model - Logical formulae

We list below the logical rules associated with each of the nodes of the model with their references.
Note that for Cohen et al. 2015 [1] and Selvaggio et al. 2018 [5], the detailed references of the rules can be found in the initial publications.

The notation for the logical connectors is:
& for AND
| for OR
! for NOT

TABLES OF LOGICAL RULES

Variable	Logical rule	Source
HIF1A	!Oxy	[2]
FAK	(ECM | (SRC)) & !p53	For ECM/SRC interaction:[3]<br>For p53 interaction: [4]
YAP1	((!AKT1 | ! AKT2) & SRC)	[3]
RAC1	(SRC | FAK) & !(AKT1 | AKT2)	Following discussions with experts, [6], [7]
PIK3CA	(GF | RAC1)	[6]
MMPs	(MMPs & (((NICD & SMAD)| RAC1) & !p73)) | p63	Following discussions with experts, [7], [10]<br>MMP autocatalytic activation: accounts for the maintenance of the MMP already secreted in the environment (e.g., MMP2)
SRC	FAK	[5]
NICD	(!p73 & !p53 & !p63 & !miR34 & !miR200 & (ECM | FAK))	[1]
CTNNB1	(!DKK1 & !p53 & !AKT1 & !p63 & !miR34 & !miR200 & !CDH1 & CDH2 & !SRC)	[1]
DKK1	(!NICD & CTNNB1) | (NICD)	[1]
AKT2	TWIST1 & (TGFbeta | GF | CDH2) & !(miR203 | miR34 | p53)	[1]
ZEB1	((TWIST1 & SNAI1) | CTNNB1 | SNAI2 | NICD) & ! miR200	[1]
SNAI1	(NICD | TWIST1) & ! miR203 & ! miR34 & ! p53 & ! CTNNB1	[1]
ZEB2	(SNAI1 | (SNAI2 & TWIST1) | NICD) & ! miR200 & ! miR203	[1]

p73	(!AKT2 & !ZEB1 & !p53 & !AKT1 & DNAdamage & !YAP1)	[1], [8] interaction found in KEGG database, reference: 21808241
p53	(DNAdamage | CTNNB1 | NICD | miR34) & ! SNAI2 & ! p73 & ! AKT1 & ! AKT2	[1]
AKT1	(CTNNB1 & (NICD | TGFbetaR | GF | CDH2) & ! p53 & ! miR34 & ! CDH1)	[1]
p63	(!NICD & !AKT2 & !p53 & !AKT1 & DNAdamage & !miR203)	[1]
miR34	!(SNAI1 | ZEB1 | ZEB2) & (p53 | p73) & AKT2 & ! p63 & ! AKT1	[1]
SNAI2	(TWIST1 | CTNNB1 | NICD) & ! miR200 & ! p53 & ! miR203	[1]
miR200	(p63 | p53 | p73) & !(AKT2 | SNAI1 | SNAI2 | ZEB1 | ZEB2)	[1]
TWIST1	CTNNB1 | NICD | SNAI1	[1]
CDH1	(!AKT2 & !ZEB1 & !ZEB2 & !SNAI1 & !SNAI2 & !TWIST1 & !SRC & Neigh)	[1]<br>SRC: discussions with experts, [9]
CDH2	(TWIST1 | SRC)	[1], discussions with experts, [5], [9]
TGFbetaR	(NICD & !CTNNB1 & TGFbeta)	[1]
miR203	(!ZEB1 & !ZEB2 & !SNAI1 & p53)	[1]
ERK	((SMAD | CDH2 | GF | NICD) & !AKT1)	[1]
SMAD	(!miR200 | !miR203) & (TGFbetaR | YAP1)	[1] [5]
p21	((SMAD & NICD) | p63 | p53 | p73 | AKT2) & !(AKT1 | ERK)	[1]
VIM	CTNNB1 | ZEB2 | SRC	[1] [9]
EMT	(!CDH1 & CDH2) | EMT & (!CDH1 & CDH2)	[1]
Migration	(AKT2 & !AKT1 & !miR200 & ERK & VIM & EMT & ((CDH2 & SMAD) | (CTNNB1)) & !p63)	[1]
Apoptosis	(p53 | p63 | p73 | miR200 | miR34) & ! ZEB2 & ! AKT1 & ! ERK	[1]
ECM_adh	(NICD & !CDH1 & SMAD) | RAC1	[6], [7]
ECM_degrad	MMPs	
CellCycleArrest	(miR203 | miR200 | miR34 | ZEB2 | p21) & !AKT1	[1]

Cell_freeze	(Neigh & !CDH2 & CDH1)	
Cell_growth	((ERK & !p21) | (AKT1 & AKT2 & PIK3CA)) & !HIF1A	For HIF1A interaction: [2]

Table S1. Table of logical rules of the intracellular model

TABLE OF PHENOTYPES AND THEIR MEANING

To simplify the analysis, we defined some read-outs, or phenotypes, that are a combination of other variables of the model. We explain below the biological interpretation of these phenotypes.

EMT	Node monitoring the epithelial to mesenchymal transition, it changes the values of adhesion of the cells.
Migration	Activates the migration of cells towards the source of Oxygen.
Apoptosis	Triggers the death of the cell.
ECM_adh	Changes the value of the "integrin" parameter inside the code, increasing the adhesion to the ECM and the amount of ECM that is degraded when ECM_degrad is ON.
ECM_degrad	Triggers the uptake of ECM, allowing the cells to degrade the ECM.
CellCycleArrest	No behaviours linked to the extracellular model but refers to a non-dividing state
Cell_freeze	Remove ability to perform motility from the cell
Cell_growth	Allows the cell to grow and switch to phase Ki67+ pre-mitotic

Table S2. Table of outputs of the model and their biological meaning.

TABLE OF INPUTS AND THEIR MEANING

Oxy	indicates if the cell is sensing enough oxygen to proliferate. It is triggered if the amount of oxygen in the cell is less then the default threshold defined in PhysiCell
TGFbeta	indicates if the cell is sensing TGFbeta in the closest voxel within its maximum range (defined in the config file). The threshold for the node activation can be modified in the config file by the user.
ECM	indicates if the cell is sensing ECM in the closest voxel within its maximum range (defined in the config file). The threshold for the node activation can be modified in the config file by the user.
Neigh	indicates if the cell is in physical contact with other cells. The threshold for the node activation can be modified in the config file by the user.

DNAdamage	indicates if the cell is sensing damage to the DNA in terms of nucleus deformation. It can be triggered if there is an overlap between the cell nucleus and a voxel with ECM.

Table S3. Table of inputs of the model and their biological meaning

3. Simulation of initial conditions with MaBoSS

MaBoSS algorithm is based on Markov chains applied on the state transition graph. It generates trajectories that cover the all state space and estimates probabilities to have a cell state equal to 1. A maximum time is chosen so that the system has reached an asymptotic solution. In our case, we chose max_time = 150. The final probabilities are then visualized as a pie chart to facilitate comparisons between conditions.

These probabilities change according to the initial condition. In the intracellular model, different combinations of the inputs can represent different extracellular conditions. We explore the inputs to mimic the different microenvironmental conditions in which the cell will be put in a PhysiBoSS model. We thus study the possible fates of the cell in the center of the tumor and at the border of the tumor.

In Figure S3, left panel, all trajectories will lead to one cell state where Cell_freeze and Cell_growth will be equal to 1 and the rest of the selected phenotypes to 0 for the inputs [Oxy,GF,Neigh]=[1,1,1] corresponding to conditions inside the tumor where the cell has access to Oxygen, growth factors and is in contact with neighboring cells. Note that if cells have no access to growth factors, the cell will simply stay still (Cell_freeze = 1, see Jupyter notebook for simulation).
Note in this figure, we focus on the visualization of a subset of nodes, the outputs. As a consequence, this model state [Cell_freeze,Cell_growth]=[1,1] can correspond to more than one stable state, but all the stable states will have in common the outputs we are visualizing (internal nodes might differ and thus be considered as different stable states).

At the border of the tumor in the absence of DNA damage and where the cell can be in contact with the ECM and TGFbeta (Figure S3, middle panel). All trajectories will lead to a state where the cell has gone through EMT, has acquired the potential to migrate, but can still be in contact with other cells (this is the case for cells moving along a fiber for instance).

We notice that when we activate DNA damage, about 76% of the trajectories show an inhibition of EMT (Figure S3).

Finally, in Figure S3, right panel, DNA damage has been triggered by an overlap between the tumor cell nuclear radius and the radius of the voxel containing the ECM. More clearly, this corresponds to a situation of high pressure conditions (crowding), which cause

nucleus deformation and as consequence, the rupture of the nuclear envelope. This is then interpreted as DNA damage in the model.
In this case, a signal is sent to degrade the ECM. When ECM_adh is activated, the cell activates the recruitment of integrins which influence the amount of ECM that a tumor cell can degrade at each time step. The node ECM_degrad is monitored by the the metalloproteases (MMPs)

In PhysiBoSS, if several model states are reachable, only one trajectory of MaBoSS is “chosen”, which means that in the case of S3, right panel, the fate of the cell will be one of the outputs with the corresponding probability, e.g., ~22% of the trajectories will lead to [Cell_growth,ECM_adh,EMT, Migration]=[1,1,1,1].

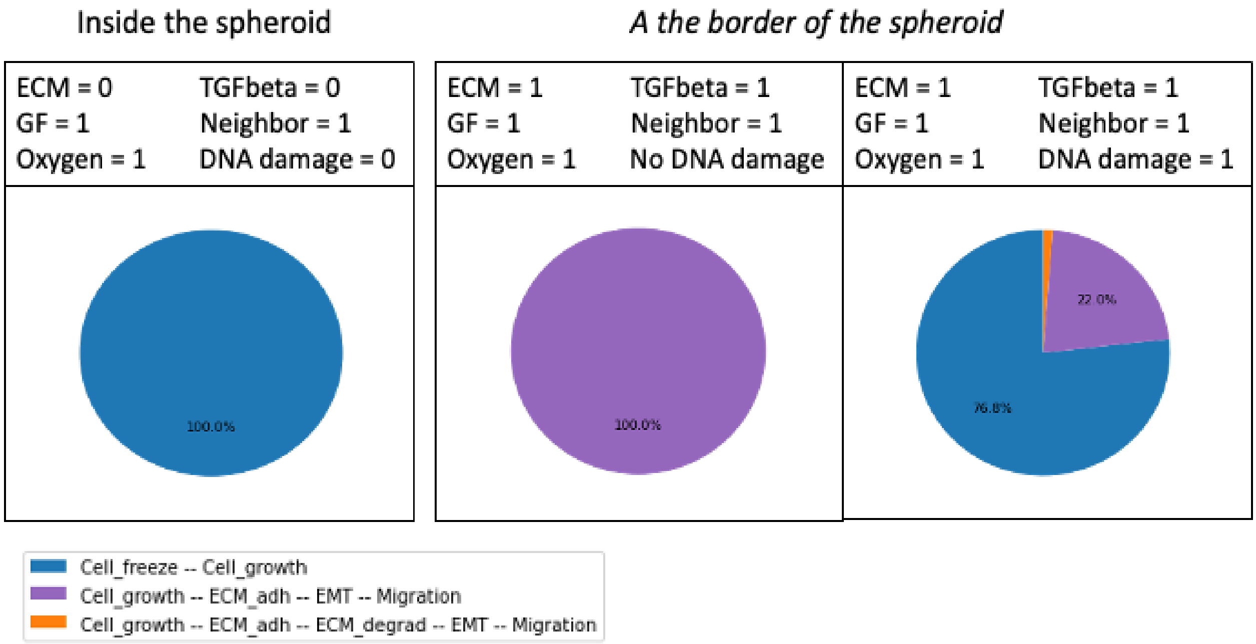

Figure S3. Asymptotic solutions of the MaBoSS model are depicted as pie charts representing the proportion of cells in a state (i.e., phenotype). Two conditions are simulated: (A) at the center of the tumor with the presence of oxygen, growth factors and in contact with other cells, and with the same conditions but (B) at the border of the tumor, in contact with ECM and exposed to TGFbeta, and (C) with DNA damage.

MaBoSS is run until the system has reached its asymptotic solution. However, if we wait too long (time = 150 units of time), very few cells are able to degrade the ECM. At time = 12 u.t. many cells are able to degrade the ECM (about 60%) which is more expected. For that reason, we choose the maximum time for a simulation to be 12 for the PhysiBoSS model, to take into account this transient behaviour. The study of the individual cell model allows to set the parameters that will be used in the multiscale model.

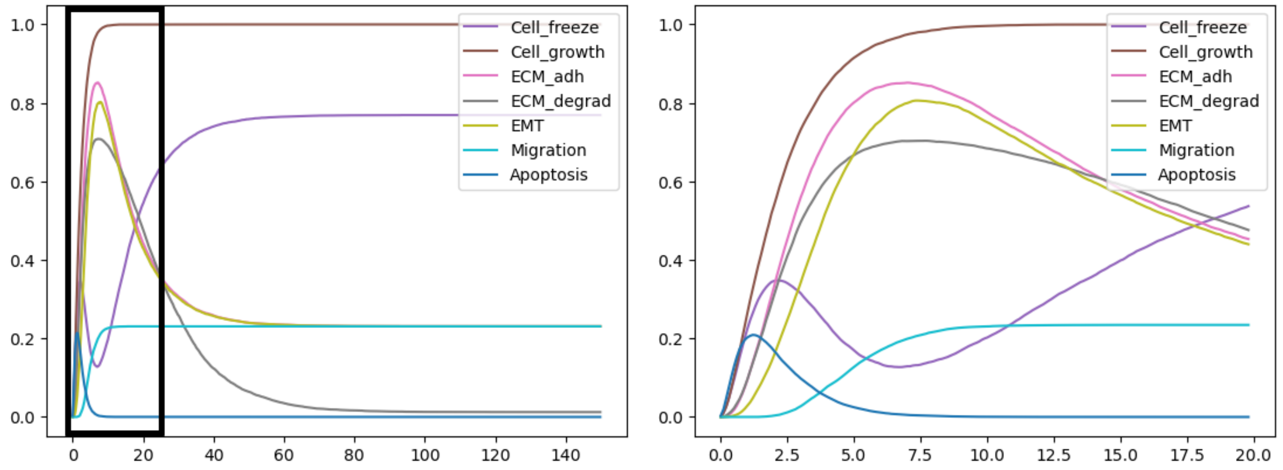

Figure S4. Transient effect of the ECM degradation in the case of DNA damage at the border of the spheroid.

In the case that DNA damage is triggered at the border of the spheroid in the absence of growth factors, apoptosis is triggered. If Growth factors are ON, they prevent apoptosis by the effect of ERK. More precisely, in response to DNA damage the apoptotic pathway is triggered (p53, p63, p73 and miRNA) but *Apoptosis* is not activated because of GF and ERK which inhibit it. When the cells detach from the main tumor and migrate, there is no longer compression of the nucleus and as a consequence, the apoptotic pathway is not activated anymore.

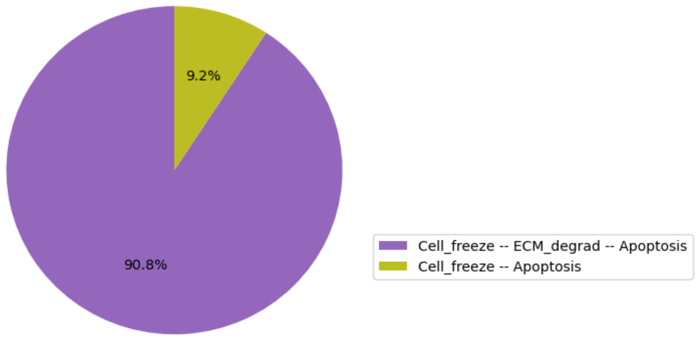

Figure S5. Apoptosis when Growth factors are OFF at the border of the spheroid when DNA damage has been activated.

We also refer the reader to the Jupyter notebook showing all the simulations of the MaBoSS model.

4. Modeling with PhysiBoSS

a. Mutations

Mutations or drug treatments are simulated by forcing the value of the corresponding nodes to 0 in the case of an inhibiting mutation (or treatment) and to 1 in the case of an activating mutation (or treatment).

b. Substrates

The oxygen in our definition of the model, diffuses from the boundary of the domain. In a 2D simulation, it diffuses from the side of the squared domain x: [-300; 300] y :[-300; 300]. In 3D,it diffuses from each face of the cubic domain x: [-300; 300] y :[-300; 300] z: [-300; 300]. The boundary condition can be modified to simulate an hypoxic or normoxic environment. In our model, we used the PhysiCell standard value of 38 mmHg with a diffusion coefficient of 6000 micron^2/min (the standard PhysiCell value is 10000 but we reduced it 40% considering the presence of the ECM according to what is reported in [16]. The ECM is a substrate with no diffusion or decay rate, which can be considered as value stored in each voxel. Unlike the oxygen and the TGFbeta or any other substrate that PhysiCell/PhysiBoSS the ECM in our model influence physically the cells, adding a repulsion terms to their motility once they approached a voxel with ECM =! 0. To do so, we added a custom function in the custom.cpp file that takes care of the physical interaction between the voxels around a cell containing ECM and the cell itself.
In the configuration file, we inserted some parameters that can be regulated by the users to simulate a softer or denser ECM or modify the repulsion strength to simulate, for example, more stiffness condition.

c. Set-up of the experiments

We adapted the simulation set-ups to simulate the published experiments. For instance, to simulate the experiments from Moitrier and colleagues, we set up a one-cell thick circle of cells to obtain a monolayer of cells grown on a Petri dish. In addition, we introduced a parameter to represent the blue light activation. At a certain simulation time (measured in minutes), the blue light substrate appears in the simulation at the center of the monolayer, as it happens in the experiments.

5. EMT activation - molecular players

EMT allows the cell to switch to a more motile phenotype, losing adhesion to neighboring cells and promoting invasiveness at the single cell level by reducing the expression of E-Cadherin (CDH1) and increasing the expression of N-Cadherin (CDH2). This cadherin switch is driven by the EMT regulators such as Twist1, Zeb1, Zeb2, Snai1, or Snai2 (Zhao et al. 2017) which activation can be induced by the focal adhesion kinase (FAK) activity, the tyrosine kinase SRC phosphorylation in response to cell-matrix contact [11][12], RhoGTPAses activity (RhoA, Rac1, Cdc42) [13][14], or the presence of Transforming Growth Factor Beta (TGF-beta)[15].

6. Cell Cycle model in PhysiBoSS

PhysiBoSS already provides the user with different options for cell cycle description.
For this study, we chose the model of the cell cycle based on Ki67 (Advanced Ki67 CellCycle model).

This cell cycle is composed of 3 phases and 3 transitions. Each cell starts in interphase Ki67-. Once the *Cell_growth* output node is activated, it triggers the transition to the next phase, Ki67+ pre-mitotic.

The cell then starts growing and once it reaches a certain threshold, it divides into two daughter cells that inherit the phenotype of the mother cell. Both daughter cells stay in Ki67+ post-mitotic phase and switch again to an early cell cycle phase Ki67- at a default transition rate provided by PhysiCell.

7. Experimental data used to validate the Boolean model

We selected three publications with images and videos to validate the model. The first one is based on the work of Ilina et al. to explore how different densities of collagen, the major component of the ECM, affect the invasion modes. Figure S3 compares the simulated experiments with Figure 2.i of their work, where they used 4T1 breast cancer cell lines in 3D collagen matrices of different densities.
The second example compares the model to the experimental results of Lodillinsky and colleagues. In their work, they observed the regulation of MT1-MMP by upregulating p63 in breast cancer ductal carcinoma in situ (DCIS). The model is able to reproduce images presented in Figure of their publication where they explored the effects of the expression of a shRNA targeting p63 on invasion (immediately or after 2 days), as seen in Figure 2 in the main text. The configuration for this experiment involves multicellular spheroids of DCIS cells embedded in a 3D type I collagen matrix.
Finally, we considered the work of Moitrier et al. where it was shown that a direct activation of the SRC oncoprotein causes the displaying of some EMT activators in Madin-Darby Canine Kidney cells (MDCK). Their configuration involves a monolayer of MDCK cells that express a light-sensitive version of SRC. Using this approach, they were able to directly modulate the SRC expression using a blue light illumination pattern. We compare the results of our simulations with Moitrier’s Figure 4.a, as seen in Figure 3 in the main text. The panel shows the cell flow and its reversibility during collective extrusion.

8. List of parameters of the model

Here we report a brief list of the main parameters of the simulation with a short description.

Parameter	Description	Value
Domain	3D / 2D space domain	600x600(x600)μm
Δspace	voxel's unit measure	10 μm
Cell-substrates interaction parameters		
ecm_adhesion_min	set the min adhesion between cells and ECM	1
ecm_adhesion_max	set the max adhesion between cells and ECM	2
cell_ecm_repulsion	set the value of ECM repulsion	15μm/min
Cell parameters		
max_interaction_factor	set the max distance of interaction	1.3μm
homotypic_adhesion_min	set the min adhesion between cells of the same type	0.4
homotypic_adhesion_max	set the max adhesion between cells of the same type	0.8
Threshold parameters		
contact_ECM_threshold	change the threshold needed to trigger ECM interaction	0.05
contact_TGFβ_threshold	change the threshold needed to trigger TGFβ interaction	0.02
contact_cell_cell_threshold	change the threshold needed to trigger Neigh node	0.3
epith_cell_junctions_attach_threshold	change the threshold needed to attach cells in cluster with cell junction	0.05
mes_cell_junctions_detach_threshold	change the threshold needed to detach cells in cluster with cell junction	0.03
Motility parameters		
migration_bias	change value of migration bias for cells with migration node active	0.85
migration_speed	change value of migration speed for cells with migration node active	0.5μm/min
motility_amplitude_min	change the min value of motility amplitude	0.1
motility_amplitude_max	change the max value of motility amplitude	0.8
Substrates parameters		
config_radius	change the initial radius of the tumor	100μm
TGFβ_radius	change radius of the TGFβ substrate	90μm
densityβ	change initial density of the TGFβ substrate	0.4
density_ECM	change initial density of the ECM substrate	0.5
ECM_degradation	change the amount of ECM degraded by the cells	0.05
ECM_TGFβ_ratio	change the amount of TGFβ degraded by the cells	0.002

TGFβ_degradation	change the threshold needed to start sensing TGFbeta inside a voxel with ECM	0.75

Table S4. Simulation parameters for the physical parameters

9. Lodillinksy results on intracellular model and ERK scenario

In the work of Lodillinsky et colleagues, they showed that there exists a direct interaction between one of the isoforms of p63, ΔNp63α, and MT1-MMP. Following their work, we directly linked in the boolean model the p63 and the MMPs node.

The figure below is an extract of the analysis on the boolean model from the Jupyter Notebook (Supp. Mat. 2, section 11 and 12). In wild type conditions, p63 overexpression induces a high level of ECM degradation while blocking Migration and reducing EMT.

p63 inhibition instead, lowers the level of ECM degradation, while rising EMT and Migration.

At the border of the tumor, the overexpression of p63 completely induces the degradation phenotype while blocking migration, which as reported in the main text, can be considered as tumor expansion. Inhibition of p63 leads to a complete inhibition of the degradation phenotypes, while maintaining active Migration and EMT, which can be considered as tumor confinement.

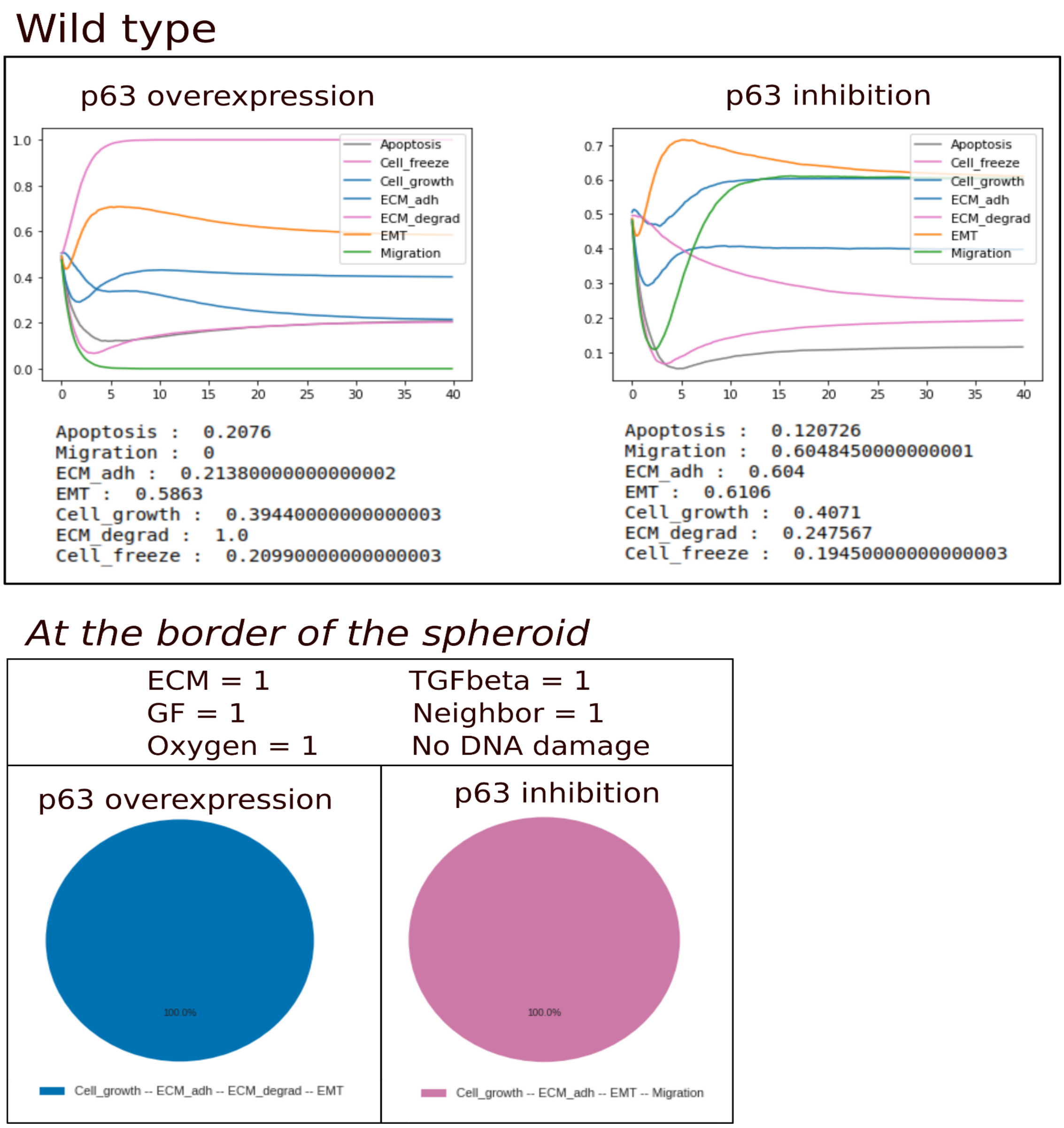

Figure S6. MaBoSS simulations of wild type, p63 overexpression and inhibition

To better assess the difference between the knock-in and knock-out of p63, we performed an analysis of the density of the tumor. For each condition, we extrapolated the average radius of the tumor, calculated the total surface and finally got the density using the total number of cells (expressed as # of cells / area). Finally we plot a heatmap that represents the number of cells in each pixel.

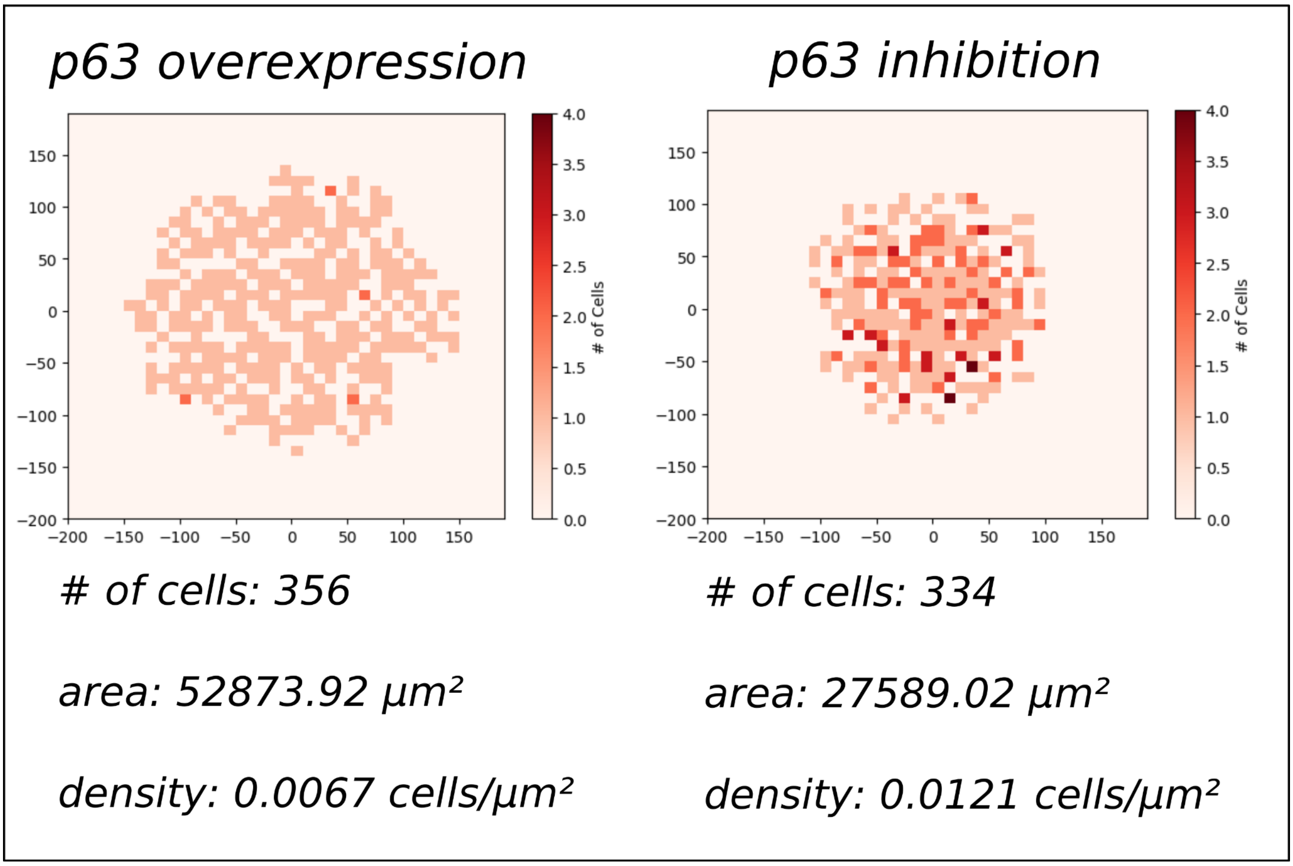

Figure S7. Density of the tumor with PhysiBoSS simulations

As expected, p63 inhibiting condition leads to a tumor confinement situation, characterized by a reduced area occupied by the tumor and a major density.

Finally, we further extended the Lodillinsky scenario introducing an ERK knockout on top of the p63 overexpression, in order to stop the invasive phenotype. As shown in the figure below, the ERK knock-out induces almost immediately the apoptosis in almost all the cells of the tumor, effectively blocking the tumor expansion.

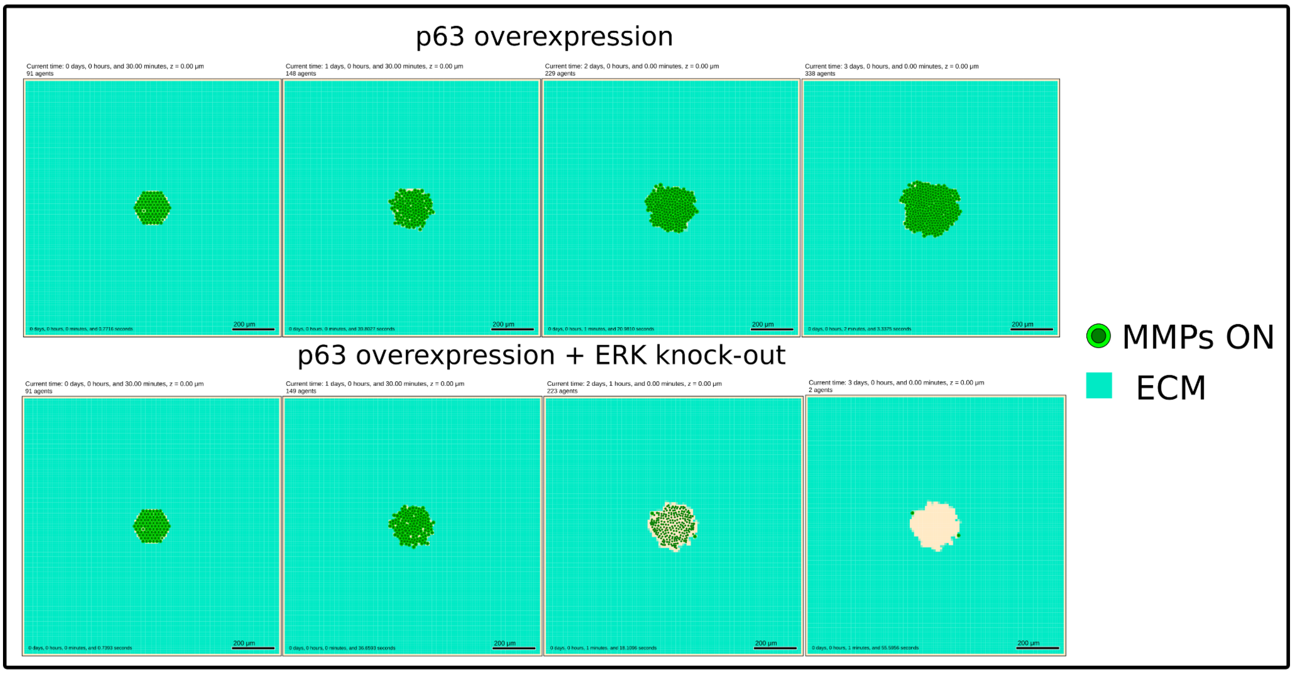

Figure S8. Simulations for predictions of the effect of some in silico treatments

10. *In silico* experiments and predictions

We show two *in silico* treatment examples selected from the automatic search that show an interesting output. The two candidates are CTNNB1 and miR34 overexpressions.

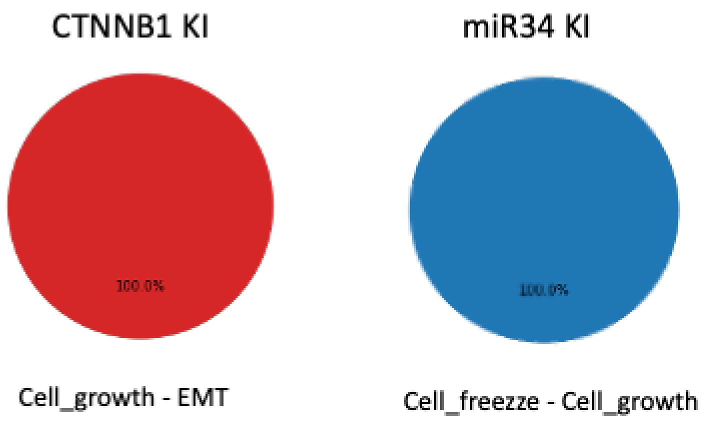

Figure S9. MaBoSS pie charts of two model alterations.

For CTNNB1 KI: Cells become mesenchymal but do not migrate
For miR34 KI: Cells remain epithelial and lose their ability to move

Following this analysis of the intracellular model (see Supplementary_file2-Intracellular_model_analysis) we tested a possible overexpressing mutation of CTNNB1. As shown in the figure, compared to the standard condition, this mutation is not preventing the tumor from growing, but greatly affects the invasive capacity.

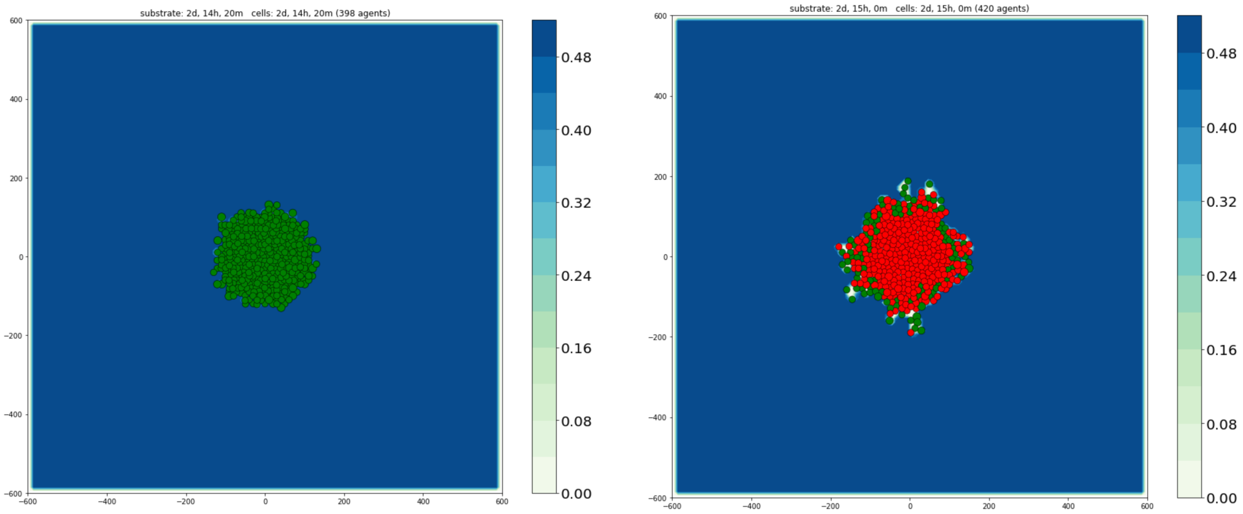

Figure S10: On the left, simulation of the model in standard initial condition with no mutation. Green cells represent mesenchymal cells, red cells epithelial. On the right, knock-out mutation of CTNNB1.

11. Simulation of local light-activation of the SRC oncoprotein in an epithelial monolayer promotes collective extrusion - 3D scenario and ERK knock-out

The following are the 3D representation of the SRC experiment presented in 3.2 of the main text.

The different shades of red represent the amount of cell junctions. The cells in the center of the monolayer are affected by the light that causes the SRC activating mutation.

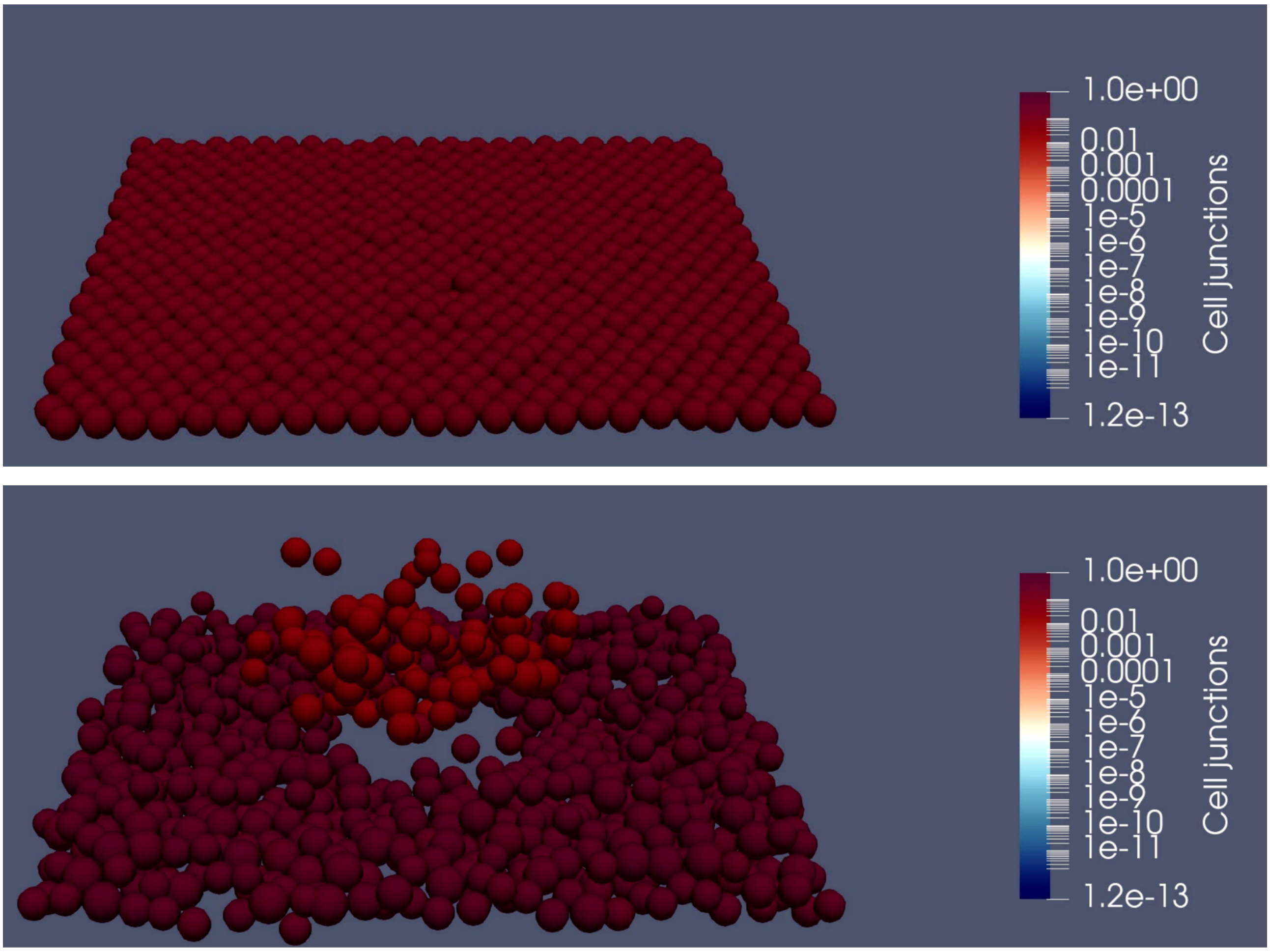

Figure S11: 3D representation of the SRC in silico experiment. To avoid the activation of the mesenchymal phenotype, the extracellular matrix was removed. The cells at the center of the monolayer undergo EMT upon activation of SRC mutation.

We then extended the 2D scenario simulating the SRC overexpression on the whole tumor. This caused a burst of invasion, speeding up the formation of both clusters and migrating single cells.

In the following snapshots, red cells are in an epithelial state, while green cells are in mesenchymal state.

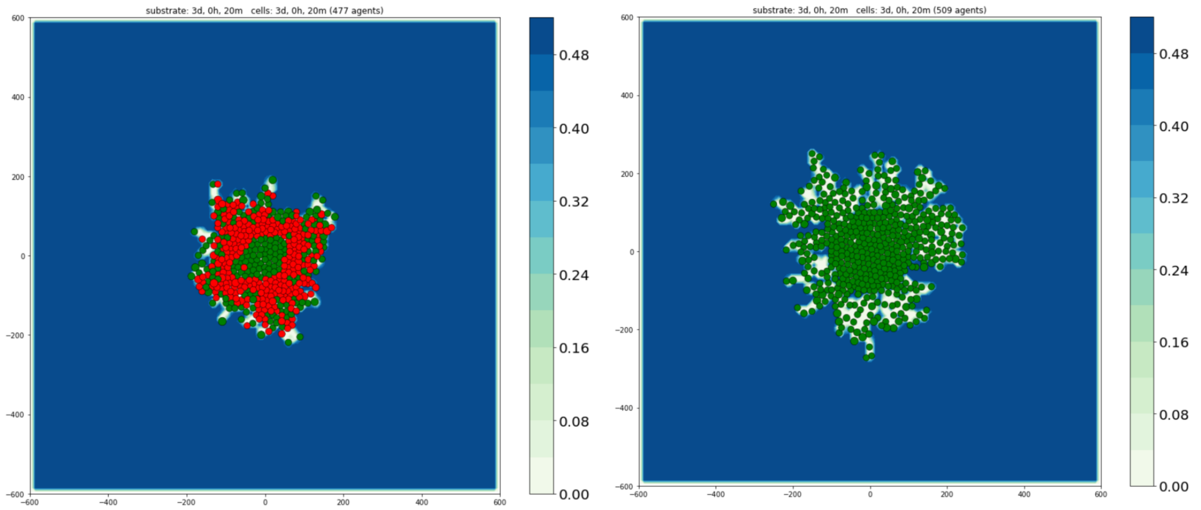

Figure S12: comparison between SRC experiment with activating mutation in the middle of the monolayer (left panel) and at whole tumor level (right panel).

As a further extension of the scenario, we simulated an ERK inhibition to try to stop the mesenchymal phenotype when SRC is overexpressed. We introduced mid simulation an ERK inhibiting mutation during the SRC overexpression on the whole tumor.

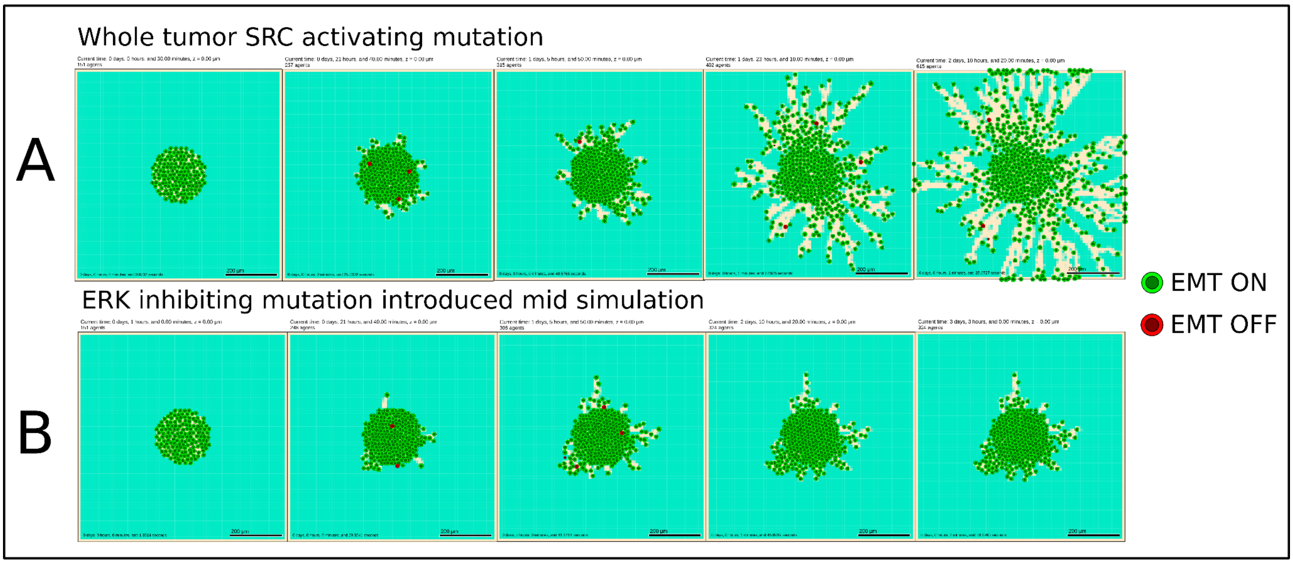

Figure S13 A) simulation of SRC experiment with activating mutation at whole tumor level for different time points. B) Introduction of ERK knock-out mid simulation. In this case, ERK knock-out stops cell movement and proliferation without causing Apoptosis.

12. Cell adhesion and density changes in the ECM regulate modes of invasion

This scenario explores the role of the ECM without any modification of the intracellular model. The model is able to reproduce images from data that were not used to build the model. In a recent study, Ilina and colleagues [17]have shown how modifying the ECM density and uniformity affects the migration modes. The experiments were performed on the 4T1 breast cancer cell line, with partial expression of CDH1. The authors observed single cell detachment for low and medium density of the collagen, the experimental proxy of the ECM. In highly dense collagen, single cell invasion was no longer observed.
We tested the impact of changing the density of the ECM with our model by changing the value of the parameter *ECM_density.* We applied a uniform random distribution in the composition of the ECM in the voxels to simulate non-uniform low density ECM (0.1<*ECM_density*<0.5). In this condition, we observed a high rate of single cell detachment as reported in the experiments (Figure S3-B) but also in the number of clusters. In a uniform and highly dense ECM (*ECM_density* = 1.0), we confirm low single cell detachment and uncoordinated collective invasion (Figure S3-A). This is due to cells at the border of the tumor going through EMT, losing cell-junction adhesion, but remaining

confined in the ECM. The model was able to reproduce both experimental observations and confirmed the role of the ECM in the types of migration that can occur during the early metastatic process.
However, the method of quantifying invasion modes remains imprecise, and will be the subject of further study in future releases (see first works on it in next section).

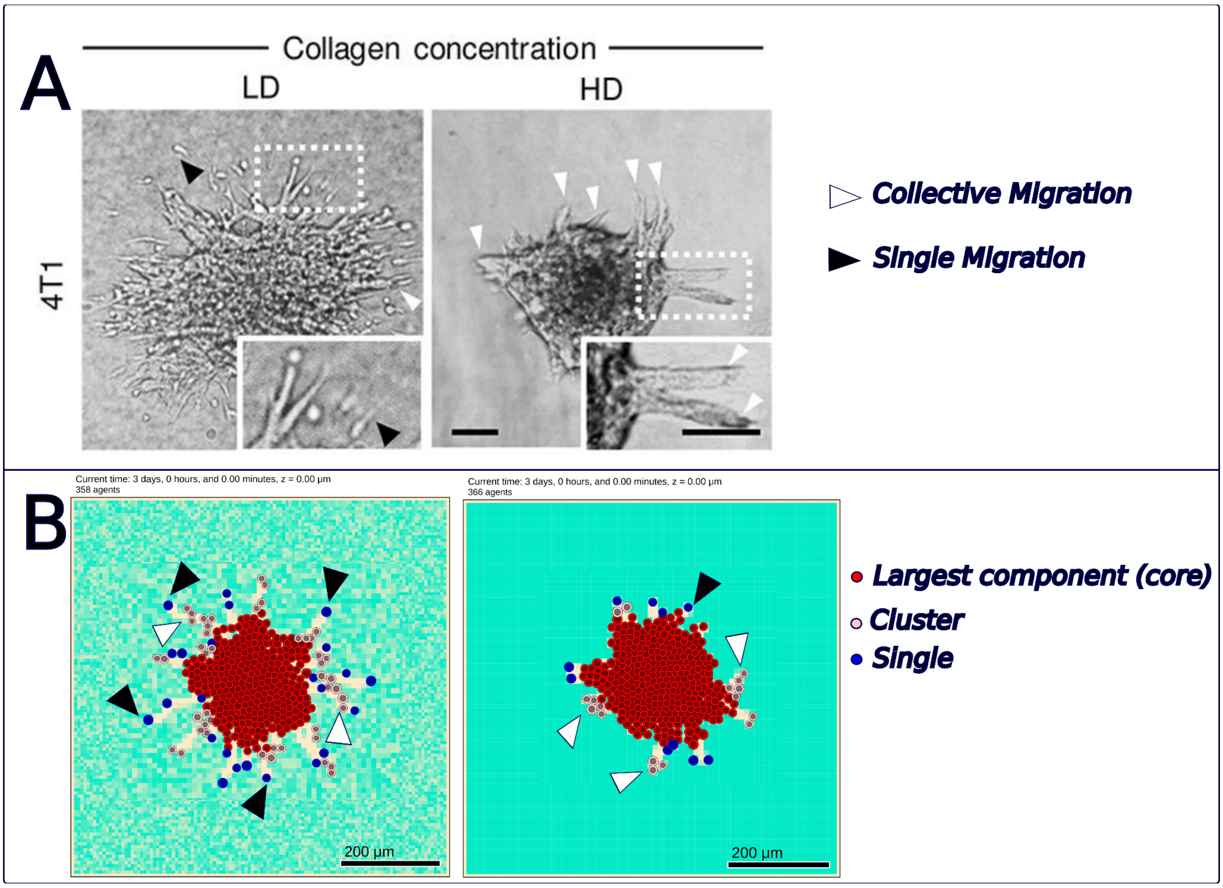

Figure S14 [copyright from Ilina et al 2020]: Single vs. collective migration observations when varying ECM density and uniformity. (A) experimental observations in response to different ECM density (in uniform (right) and non-uniform conditions, reproduced from Ilina et al 2020). (B) Model simulations of the uniform and high density ECM (right) and non-uniform low density ECM (left). White voxels represent low ECM density.

13. Quantification of the migration modes

Single cell migration occurs when a cell has lost the cell junctions with the surrounding cells and starts the invasion process on its own. Collective migration in cancer can happen when one of two dynamical features appear: (1) a group of epithelial cells go through EMT and start degrading a specific area of the ECM without developing cell junctions before migrating along extracellular boundaries (i.e., fibers, basal membrane, etc. that cannot be degraded) in an uncoordinated collective movement [17]or (2) a group of mesenchymal cells surrounding a small cluster of epithelial cells detach themselves from the main body of the tumor, thus, when epithelial cells have cell junctions with other mesenchymal cells which push them through the ECM.

In order to quantify and verify the amount of single cells and cells in a cluster, we took advantage of another output of the PhysiBoSS simulation: at each time step, the software

prints a .csv file with the list of cells and their interacting neighbors. We then imported the list on Python and used the package NetworkX to extrapolate a network where each node represents a cell/agent and each edge represents an existing interaction between cells. Finally, for the sensitivity analysis, we counted the amount of single cells, the amount of cluster and the number of cells in a cluster.
Below we present a scheme of the process.

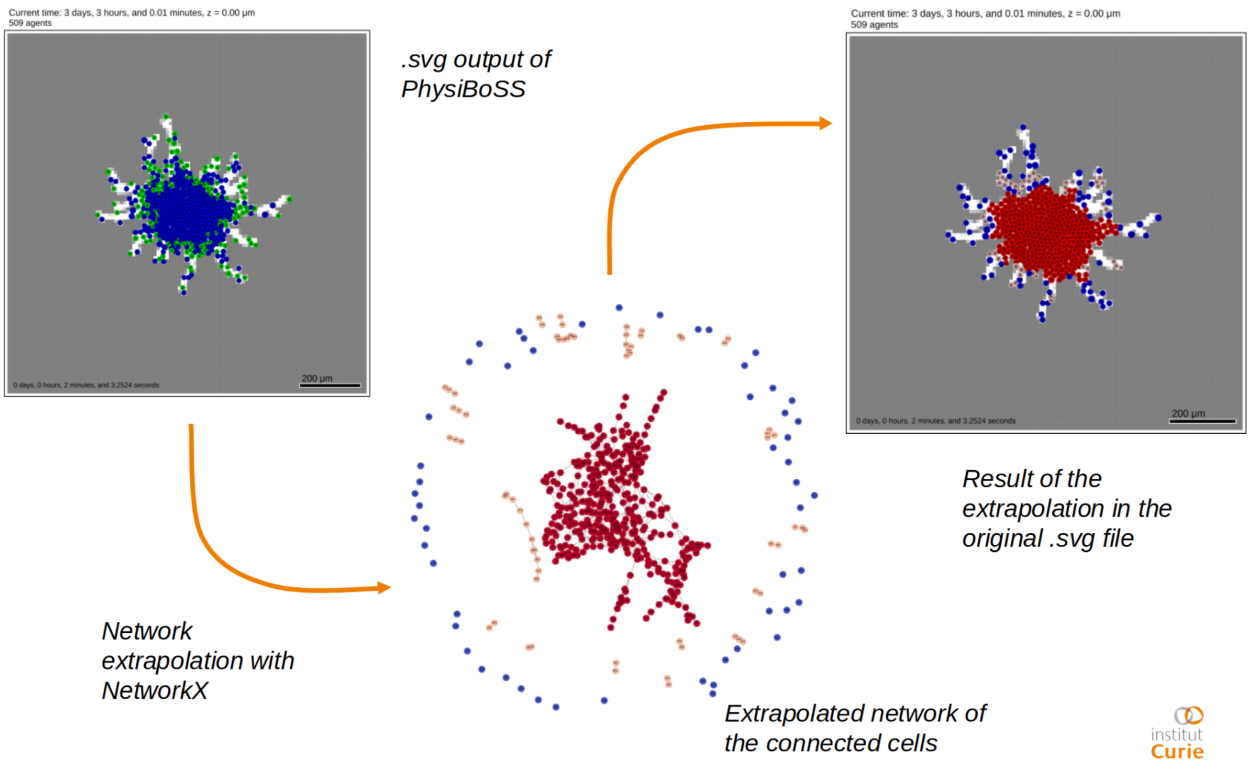

Figure S15: Scheme that represents the process of extrapolating the network of cells and then incorporate in the corresponding svg snapshot of the simulation.

14. Computational complexity of the simulation

Simulations were performed on the cluster abacus at Institut Curie, which contains 28 nodes with each running an Intel(R) Xeon(R) Gold 6130 CPU @ 2.10GHz with 40 cores, and with 192GB RAM.

Our 2D model simulates a cell population starting with 91 cells and growing to 393 cells, and is able to run in 2 minutes and 30 seconds on 30 cores of a single node.
Our 3D model simulates a cell population starting with 588 cells and growing to 7573 cells, and is able to run in 2 hours and 42 minutes on 30 cores of a single node.

The simulation of Boolean networks in PhysiBoSS represents a very small contribution (around 0.2% for our model) to the computational cost of the simulation, which is mainly due to the simulation of the diffusion of the substrates first (~80%), followed by the cell-cell and cell-substrate interactions (~20%). When only taking into account the update of cell-cell and cell-substrate interactions, the cost is around 100 times larger compared to

the cost of simulating the Boolean network. Thanks to MaBoSS, using Markov chains to simulate the Boolean network, the cost of simulation scales linearly with the number of nodes.

15. Sensitivity analysis on model parameters

We run a sensitivity analysis on the parameters of the ABM model that are linked to the BM. The goal is to measure how each parameter affects the amount of cells that migrate as single cells or as clusters. We select 7 parameters among the ones shown in the previous section. For each parameter, we choose a range of values to test based on our previous experience: these parameters are inherited from PhysiCell simulations [18] and chosen from trial and error tests to match experimental observations. We performed a bootstrap analysis to verify the minimum number of replicates that reduce the most noise due to the stochasticity of the simulation. Finally, each value, we did 50 runs and took the mean value and the squared mean error to see how the stochasticity of the simulation affected the results.

Each parameter has been tested independently. To limit the computational cost, we performed the sensitivity analysis on one parameter at a time.
The simulations took almost 90 hours on the cluster abacus at the Curie Institute (28 nodes for a total of 1120 cores, 5.25Tb of RAM).

The parameters are the following:

**Parameters**	**Range/step**	**Potential range**	**Number of values selected**
cell_ecm_repulsion<br>regulates the amount of repulsion between cell and ECM	0 < **15** < 75<br>/ 5	[0-infinite]	16
epith_cell_attach_threshold<br>changes the activation threshold needed to attach cells in cluster with cell junction	0 < **0.05** < 1<br>/ 0.05	[0-1]	21
mes_cell_detach_threshold<br>change the activation threshold needed to detach cells in cluster with cell junction	0 < **0.03** < 1<br>/ 0.05	[0-1]	21
cell_cell_contact_threshold<br>changes the activation threshold of the value *cell_contact* needed to trigger Neigh node	0 < **0.3** < 3.5<br>/ 0.02	[0-infinite]	19
cell_ecm_contact_threshold<br>changes the activation threshold of the value *ecm_contact* needed to trigger ECM	0 < **0.05** < 1<br>/ 0.05	[0-1]	21

node			
migration_bias<br>changes the value of migration bias for cells with *Migration* node active	0 < **0.8** < 1<br>/ 0.05	[0-1]	21
migration_speed<br>changes the value of migration speed for cells with *Migration* node active	0 < **0.7** < 1<br>/ 0.05	[0-1]	21

Table S5. List of parameters used for the sensitivity analysis

In order to thoroughly explore the entire spectrum of potential values and verify the robustness of our results, we opt for selecting a uniform number of values for each parameter.

a. Bootstrap analysis

We performed a series of simulations focused on a single parameter (*migration_speed*) but with an increasing number of replicates per value of the parameter (10, 20, 50, 100), in order to study how much the stochasticity affects the overall results of the model.
The figures below represent the evolution of the number of single cells (blue), number of clusters (orange) and average number of cells in a cluster (green) for different values of the “migration bias” parameter, each represented on the y-axis (Count). On the x-axis is displayed the value selected for the “migration bias” parameter.
Each box represents the quartile of the distribution, the whiskers extend to show the rest of the distribution and the points are considered “outliers” (ref. seaborn.boxplot).

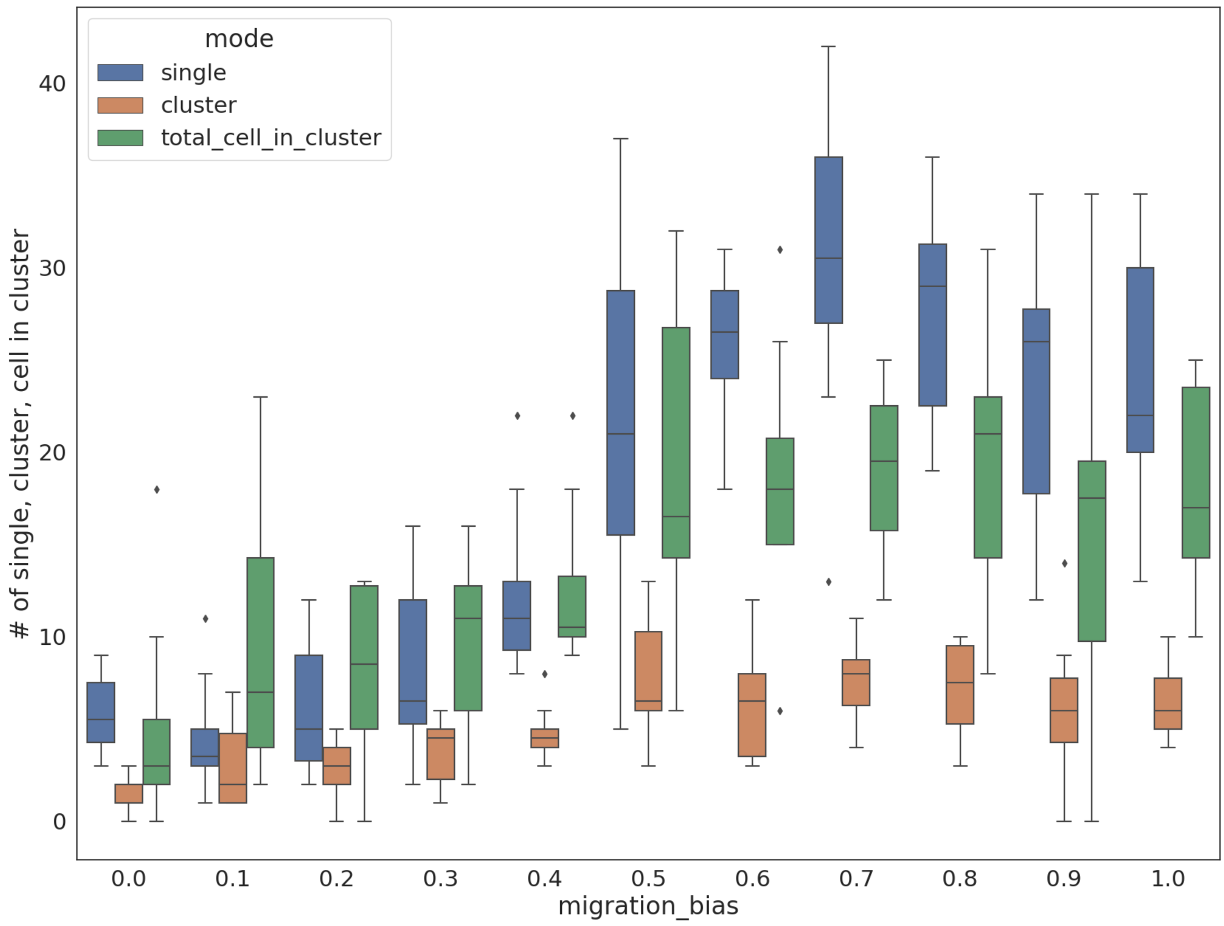

Fig. S16 Analysis of the evolution of the migrations modes for different values of the parameters “migration_bias” executed with 10 replicates.

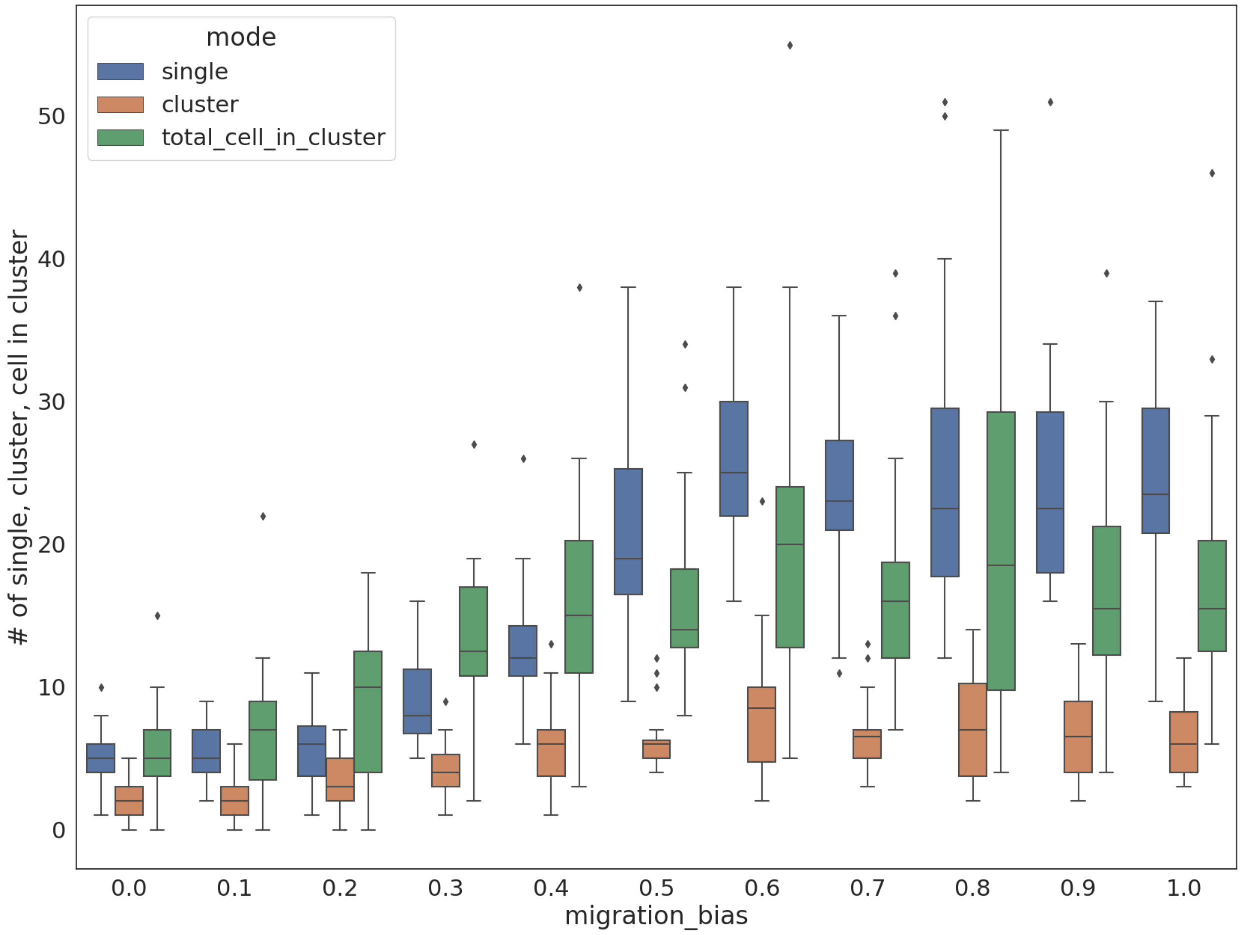

Fig. S17 Analysis of the evolution of the migrations modes for different values of the parameters "migration_bias" executed with 20 replicates.

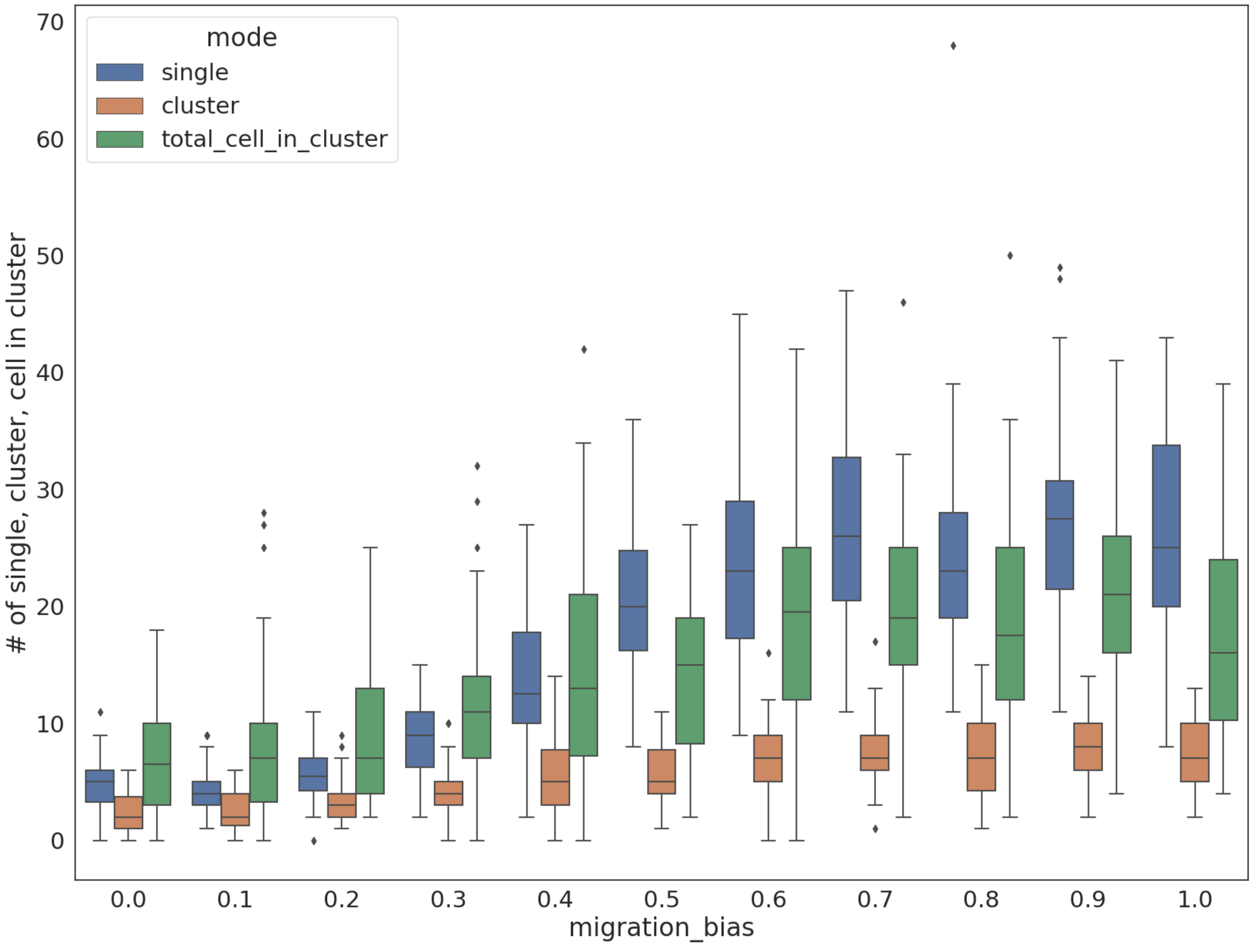

Fig. S18 Analysis of the evolution of the migrations modes for different values of the parameters "migration_bias" executed with 50 replicates.

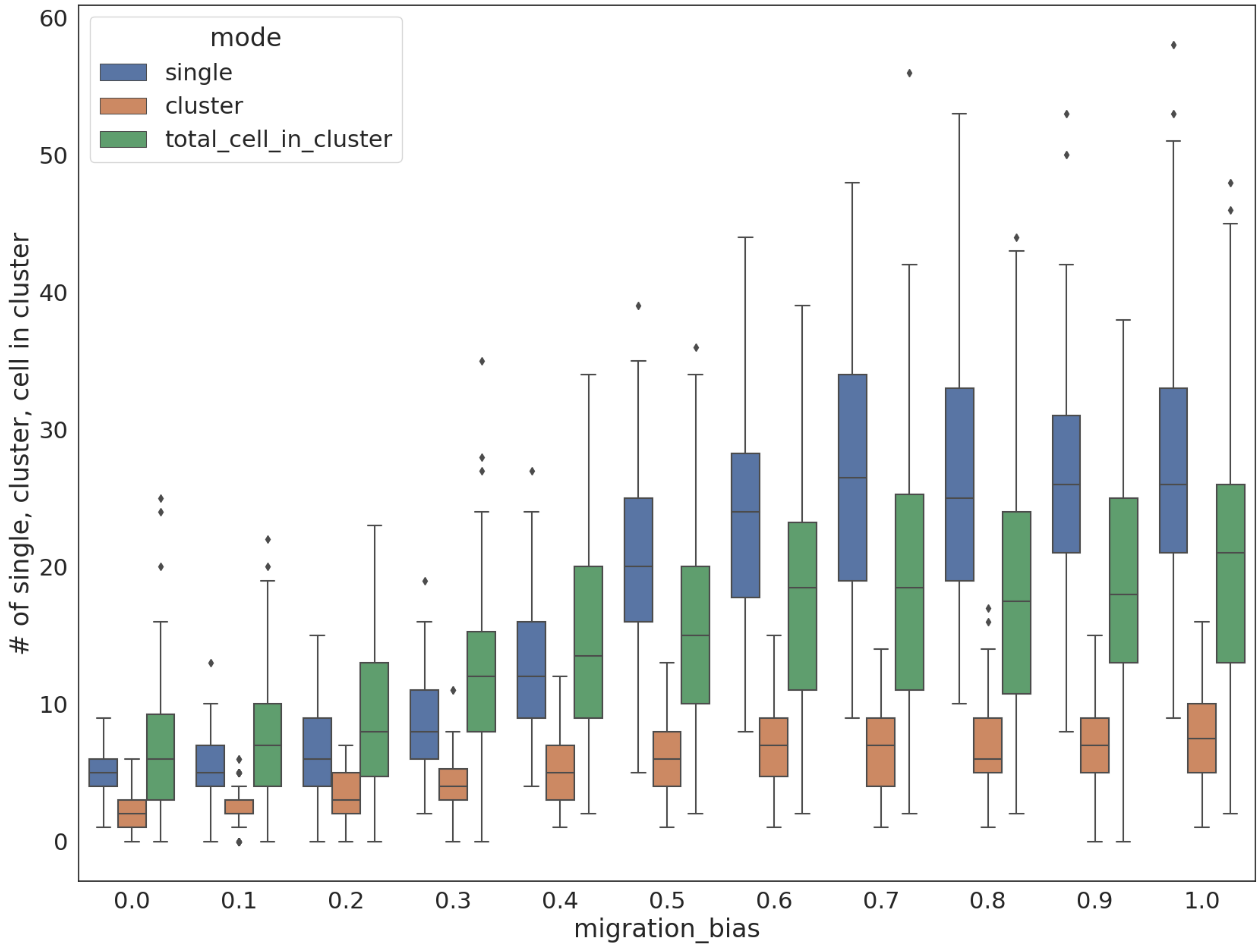

Fig. S19 Analysis of the evolution of the migrations modes for different values of the parameters “migration_bias” executed with 100 replicates.

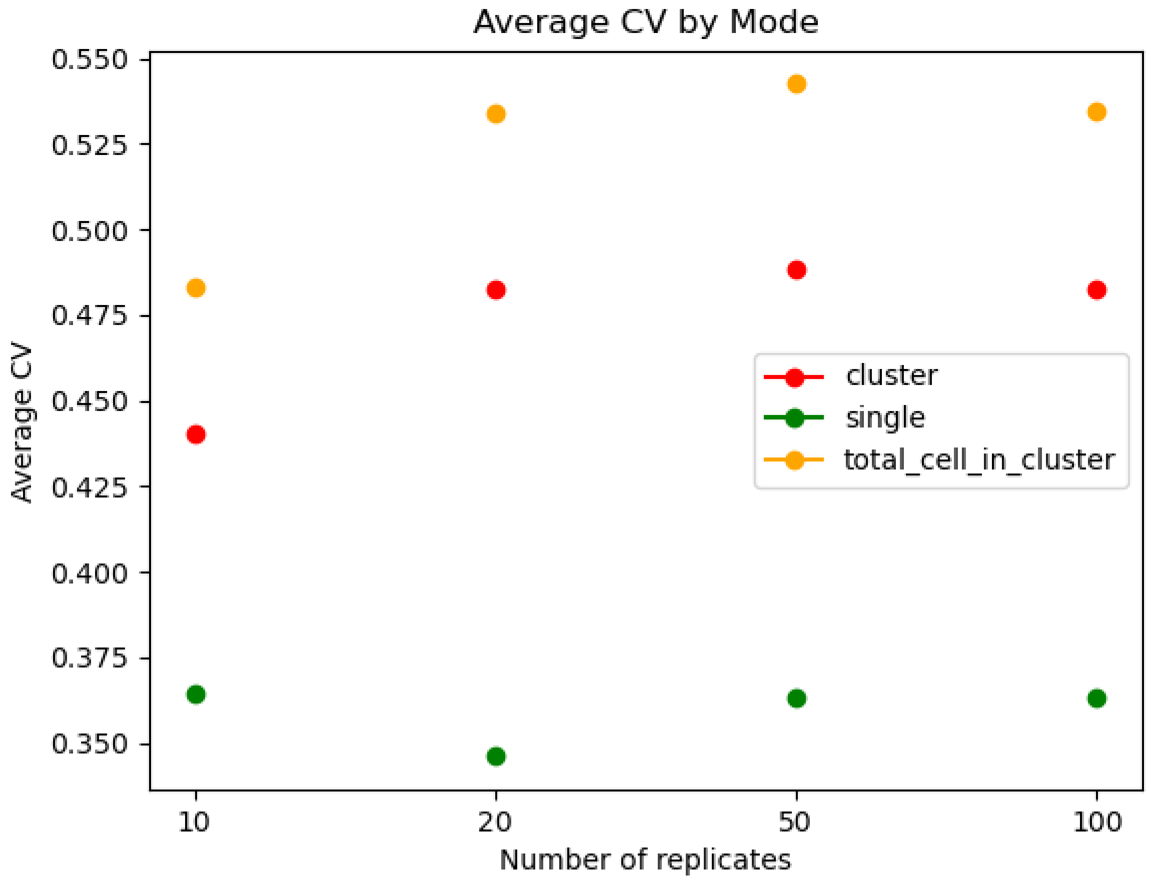

Fig. S20 Average coefficients of variation for each migration mode, for every number of replicates.

One important conclusion of this analysis is that the dispersion in our quantification stays relatively stable, despite the increased number of replicates. To quantify this, we computed the average coefficient of variation for each type of migration mode (Fig. S20). We see mostly stable values, indicating that the noise in our quantification of the migration modes is intrinsic to our model.

However, what we can observe is that the curves formed by the average values of the migration modes are getting smoother, meaning that with an increased number of replicates, we get a better average quantification of the influence of the parameters on the migration modes. To analyze this, we fitted the parameter dependency on a sigmoid, and computed R2 scores for each number of replicates (Fig. S21). With this analysis, we could indeed show that with each increased number of replicates, the R2 score would go down.

Taking this information together with our capabilities to perform a large number of computations, we decided to settle on 50 replicates per simulation.

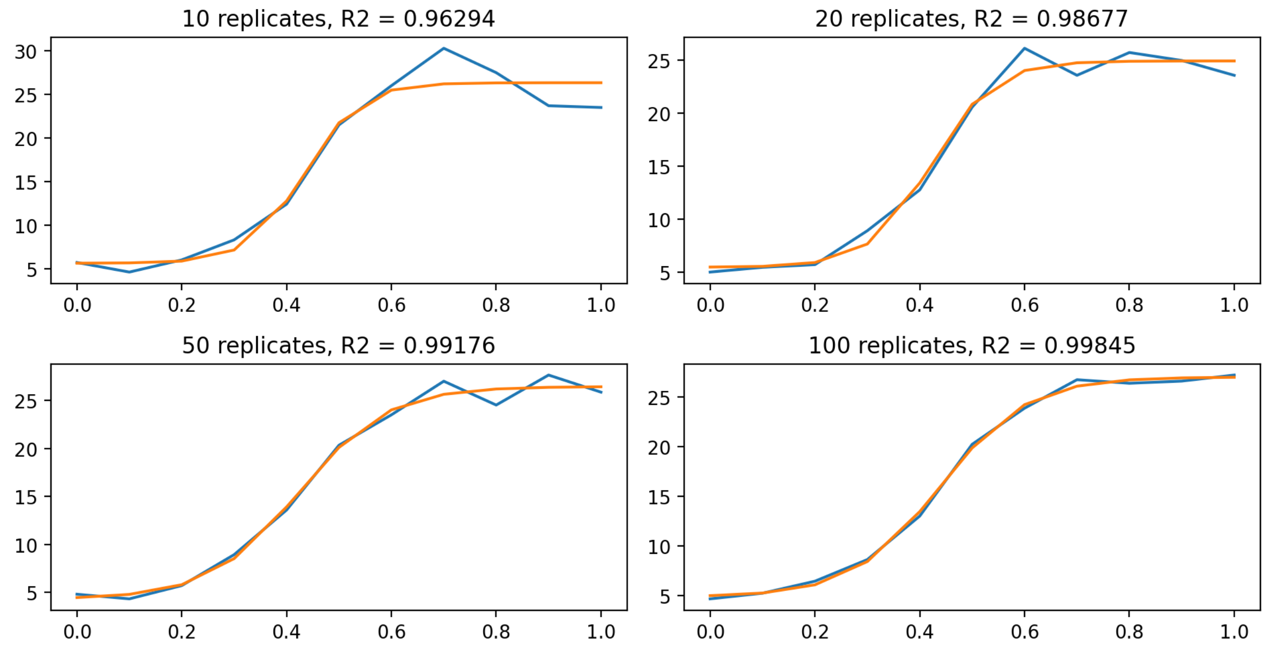

Fig. S21 Example of sigmoid fit of the quantification of single migration mode according to the migration bias, for 10, 20, 50 and 100 replicates.

b. Sensitivity analysis

The graphs below show the proportions of cells that are found as single cells (blue) or in clusters (orange) for various values of the parameters. Some results and interpretations are discussed in the main text.

The range of values selected for each parameter was chosen based on the parameter itself: some thresholds, such as "*epith_cell_attach_threshold*" "*mes_cell_detach_threshold*" and "*cell_ecm_contact_threshold*" depend on the variable to which they are associated. "*epith_cell_attach_threshold*" and "*mes_cell_detach_threshold*" for example are associated with the variable "*padhesion*" which is a percentage (0 < padhesion < 1). The same goes for "*cell_ecm_contact_threshold*", whose associated variable is "*ecm_contact*". "*migration_bias*" is also a percentage and therefore has its possible values are limited between 0 and 1.

For "*migration_speed*", we did not explore values above 1 because it caused cells to reach the spatial limit of the simulation, blocking the cells and causing problems in quantifying the invasion methods.

"*cell_ecm_repulsion*" represents a force and has no superior limit. We decided to stop the range of values to 75 because after this value there was no significant change in the invasion methods.

The same applies to "*cell_cell_contact_threshold*", which despite being a threshold, its associated variable was not normalized.

- *cell_ecm_repulsion*

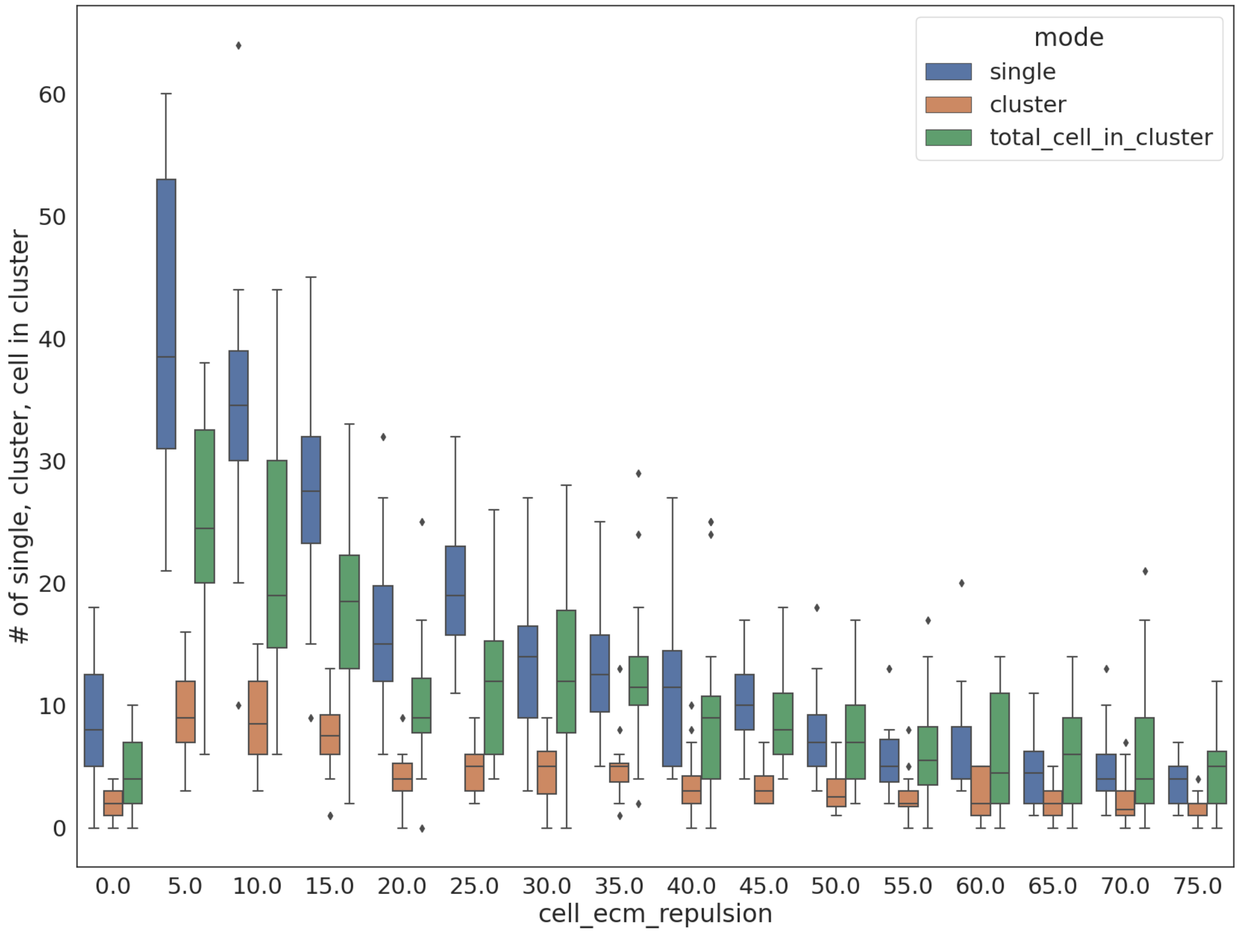

Figure S22: *Amount of single cell vs cell in cluster for different values of cell_ecm_repulsion*

This parameter controls the repulsion force that the ECM applies to the cells.
For cell_ecm_repulsion=0, the ECM loses its confinement property, allowing the tumor to expand evenly and minimizing invasive behavior.
For values higher than 5, the ECM starts repelling the cells, allowing the triggering of the invasive properties of the cells, but for higher values it also increases its confinement capacity, minimizing invasion but also blocking tumor expansion.

- *epith_cell_attach_threshold*

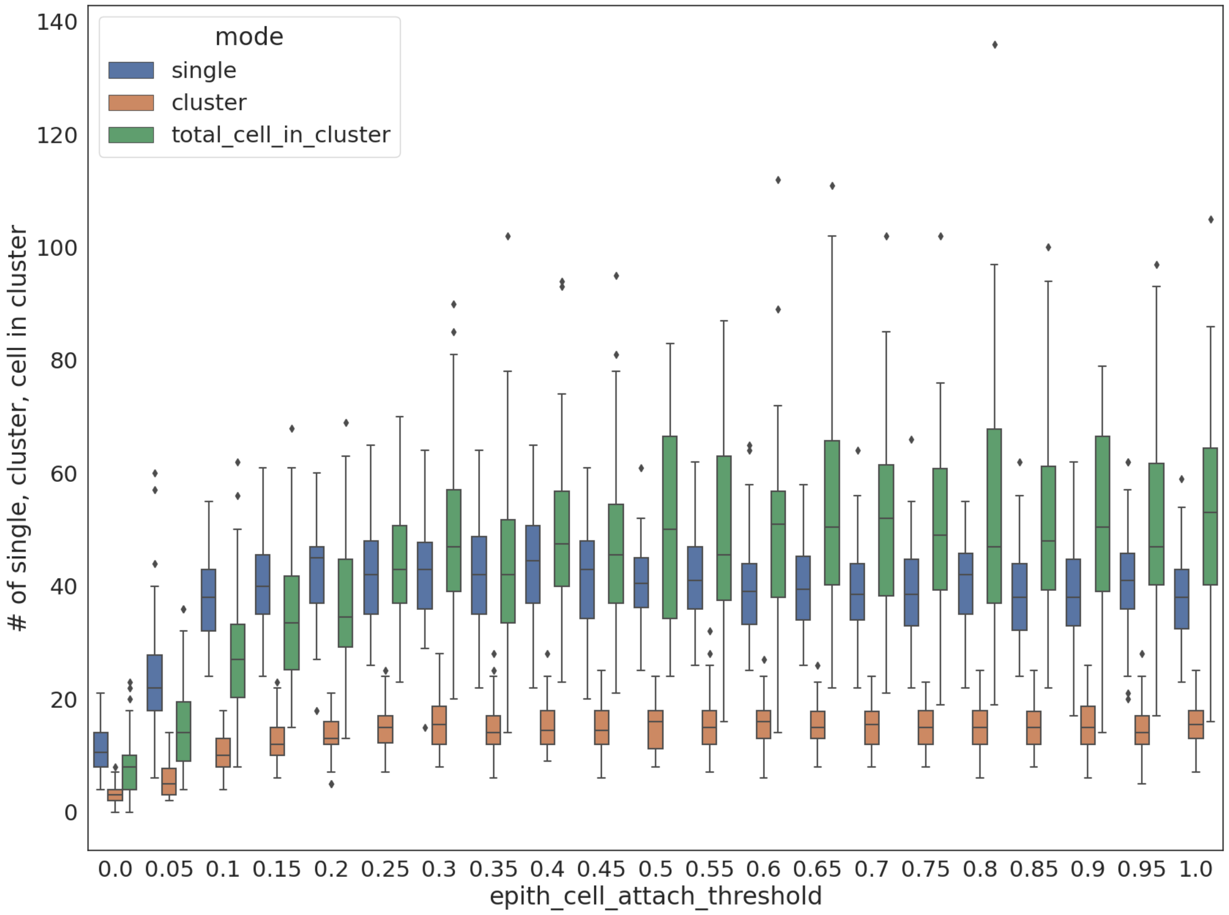

Figure S23: Amount of single cell vs cell in cluster for different values of epith_cell_attach_threshold

This parameter controls the activation threshold that allows cells in the epithelial state to form tight junctions with the cells around them, forming spring-like adhesion.
Starting from 0, increasing this threshold leads to an increase in the number of individually invading cells, the total number of cells in the clusters and a slight increase in the number of clusters. The latter reaches a plateau around epith_cell_attach_threshold = 0.2, while the number of single cells and cells in clusters increases. For high values of the parameter, the number of cells in clusters tends to exceed the number of single cells.

- *mes_cell_detach_threshold*

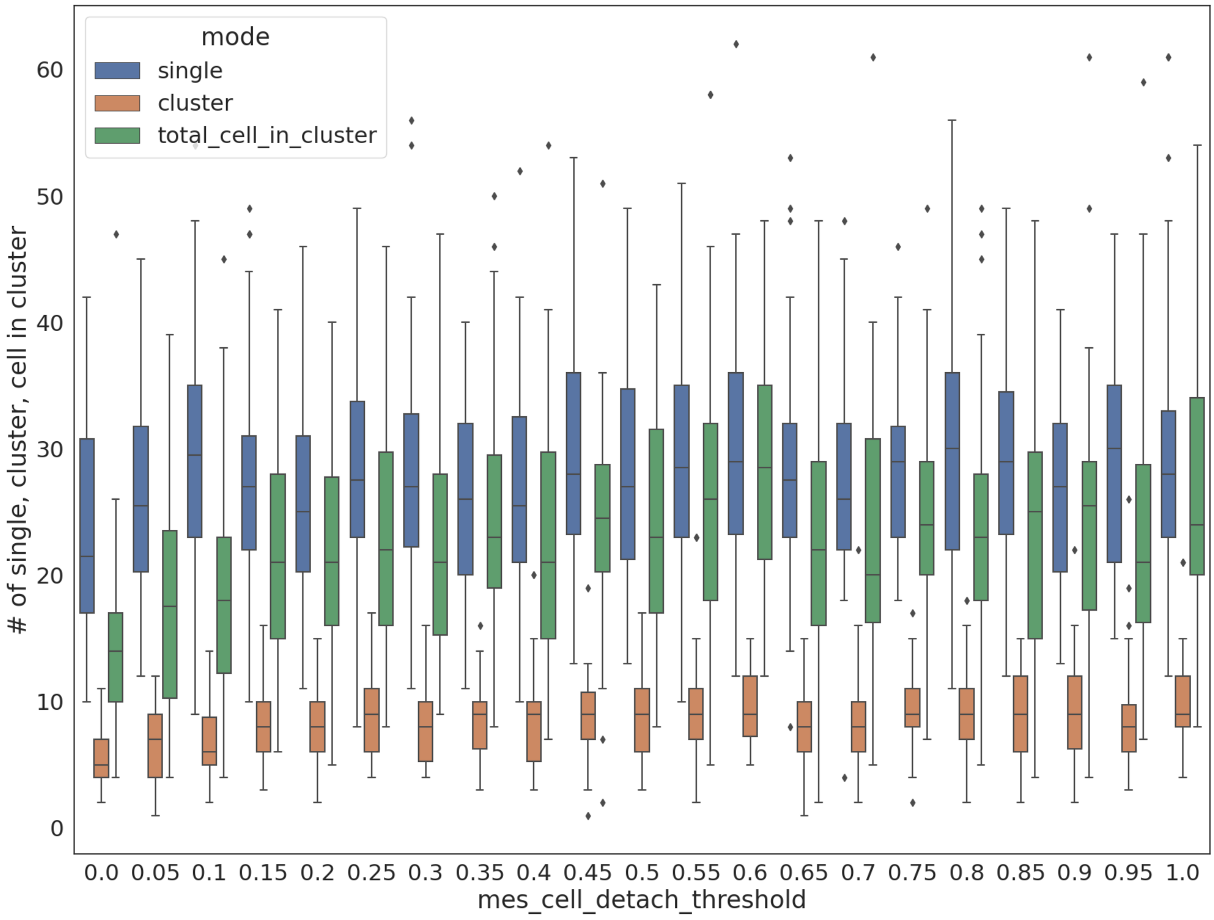

Figure S24: *Amount of single cell vs cell in cluster for different values of mes_cell_detach_threshold*

This parameter controls the activation threshold that allows cells in the mesenchymal state to disband any tight junctions previously formed with the cells around them. From the analysis, this parameter seems robust for values higher than 0.05 and it shows no changes in the rate between single and collective migrating cells.

- *cell_cell_contact_threshold*

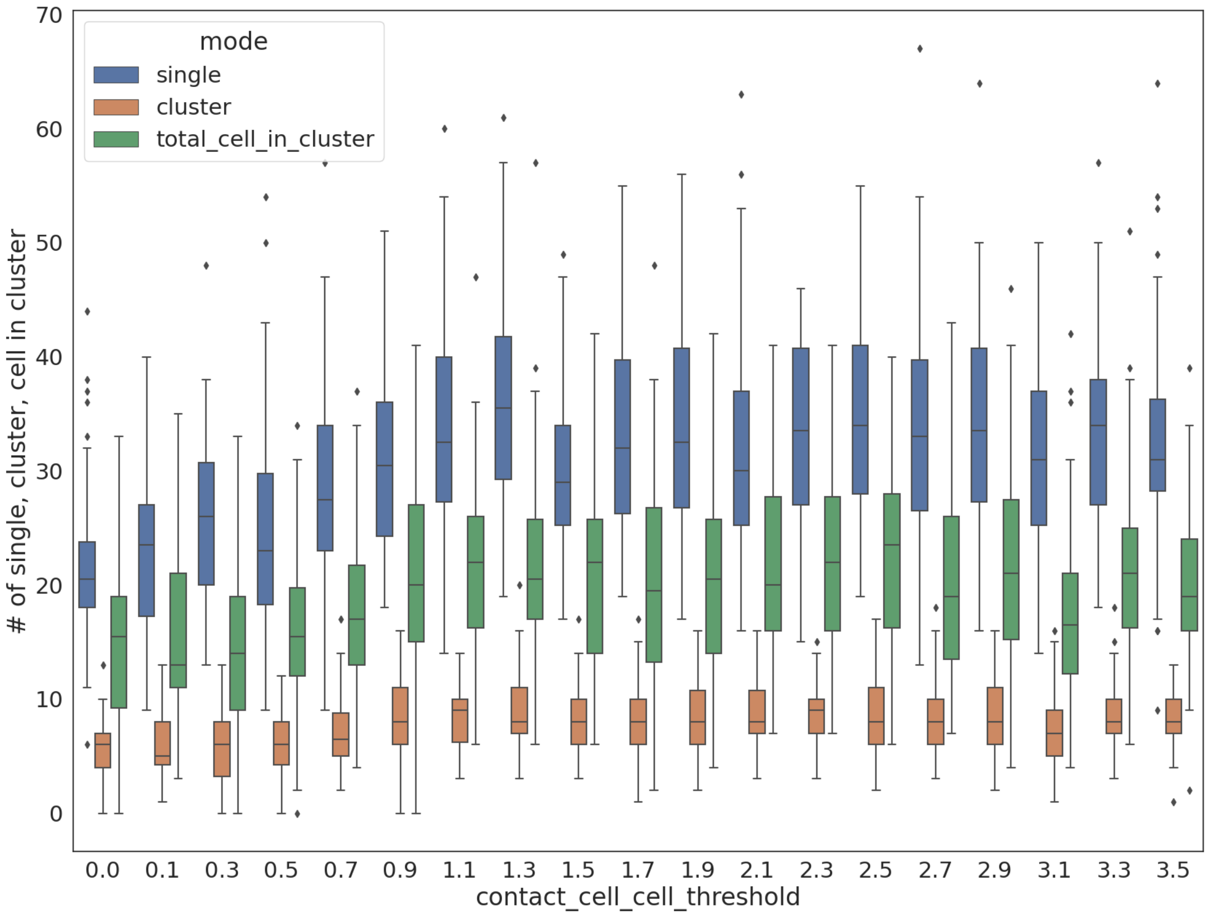

Figure S25: *Amount of single cell vs cell in cluster for different values of cell_cell_contact_threshold*

This parameter allows the activation threshold of the 'Neigh' input node to be adjusted, based on the amount of cell contact.
The analysis shows that the parameter has little impact on the separation of clusters and single cells. There is a slight growth in the values of single cells and cells in clusters, while there are almost no changes in the total number of clusters.

- *cell_ecm_contact_threshold*

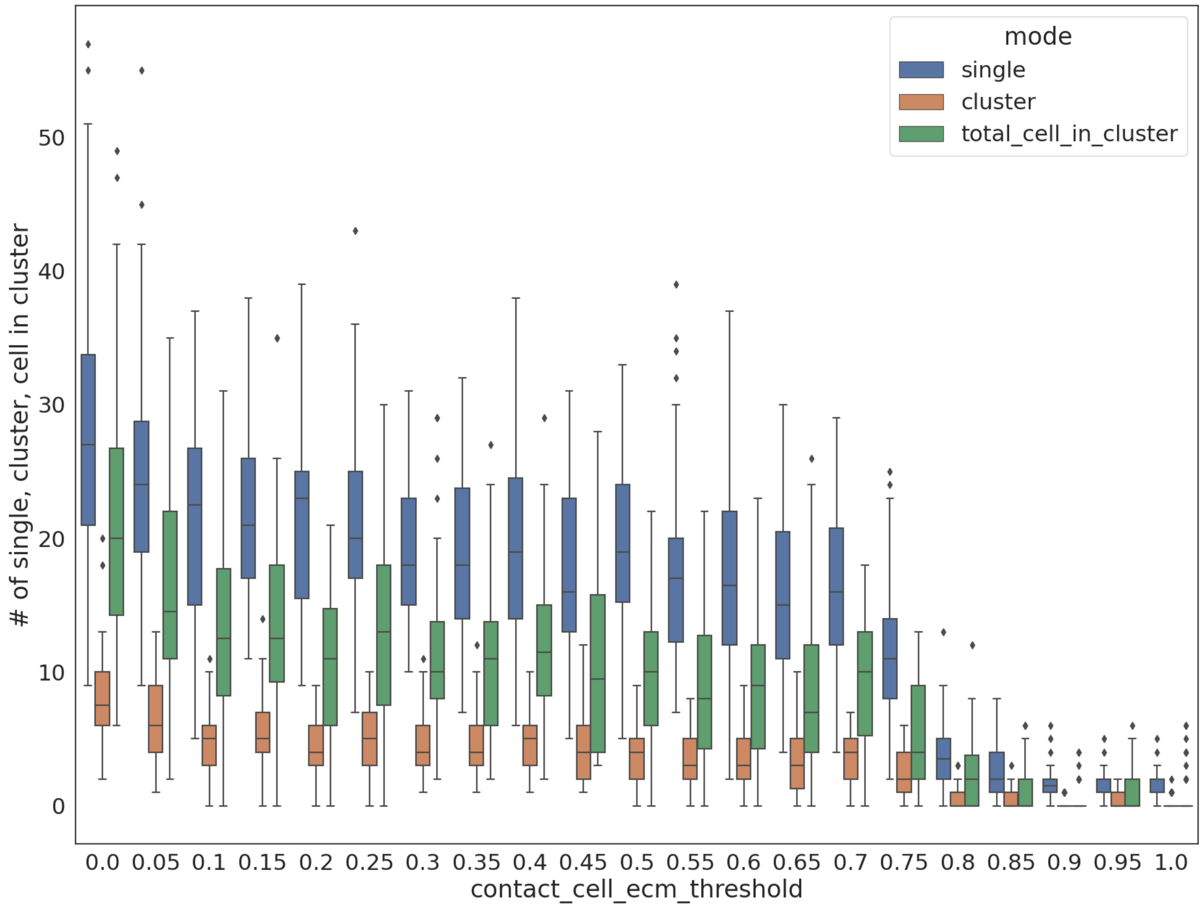

Figure S26: *Amount of single cell vs cell in cluster for different values of cell_ecm_contact_threshold*

This parameter allows the activation threshold of the 'ECM' input node to be adjusted, based on the amount of ECM contact.

The analysis of this parameter shows a slight decrease in the number of single cells and cells in clusters, while the total number of clusters is almost unchanged. For values greater than 0.7, the number of single cells and cells in clusters decreases significantly, to almost zero for values greater than 0.85.

- *migration_bias*

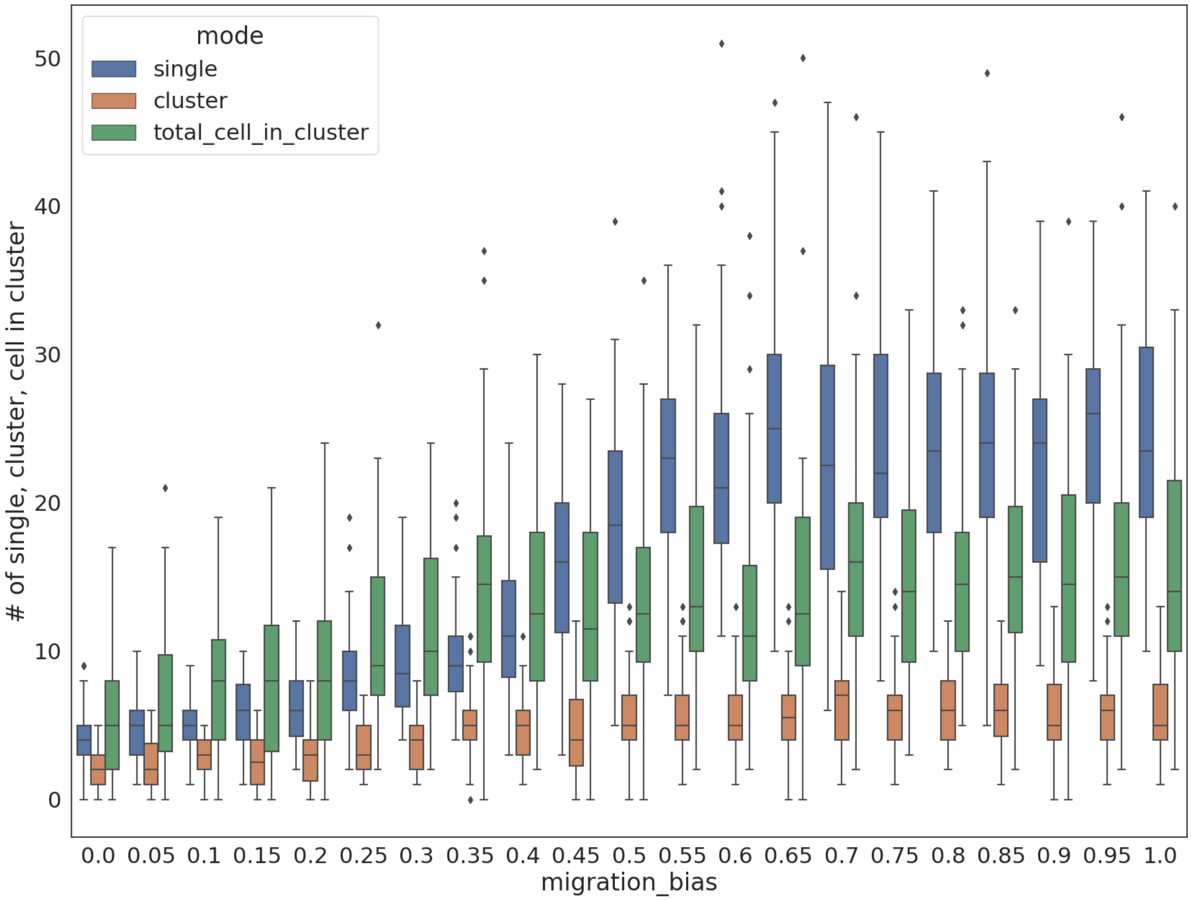

Figure S27: *Amount of single cell vs cell in cluster for different values of migration_bias*

This parameter controls the tendency of a cell to migrate towards its chemoattractant source, varying from 0 (pure random walk) to 1 (pure deterministic movement).

From the analysis, this parameter heavily influences the amount of single migration: for values below 0.45, the amount of cells migrating in clusters increases similarly to the number of single cells. For higher values, the number of single cells grows faster, exceeding the number of cells in clusters and reaching a plateau around a value of 0.55.

- *migration_speed*

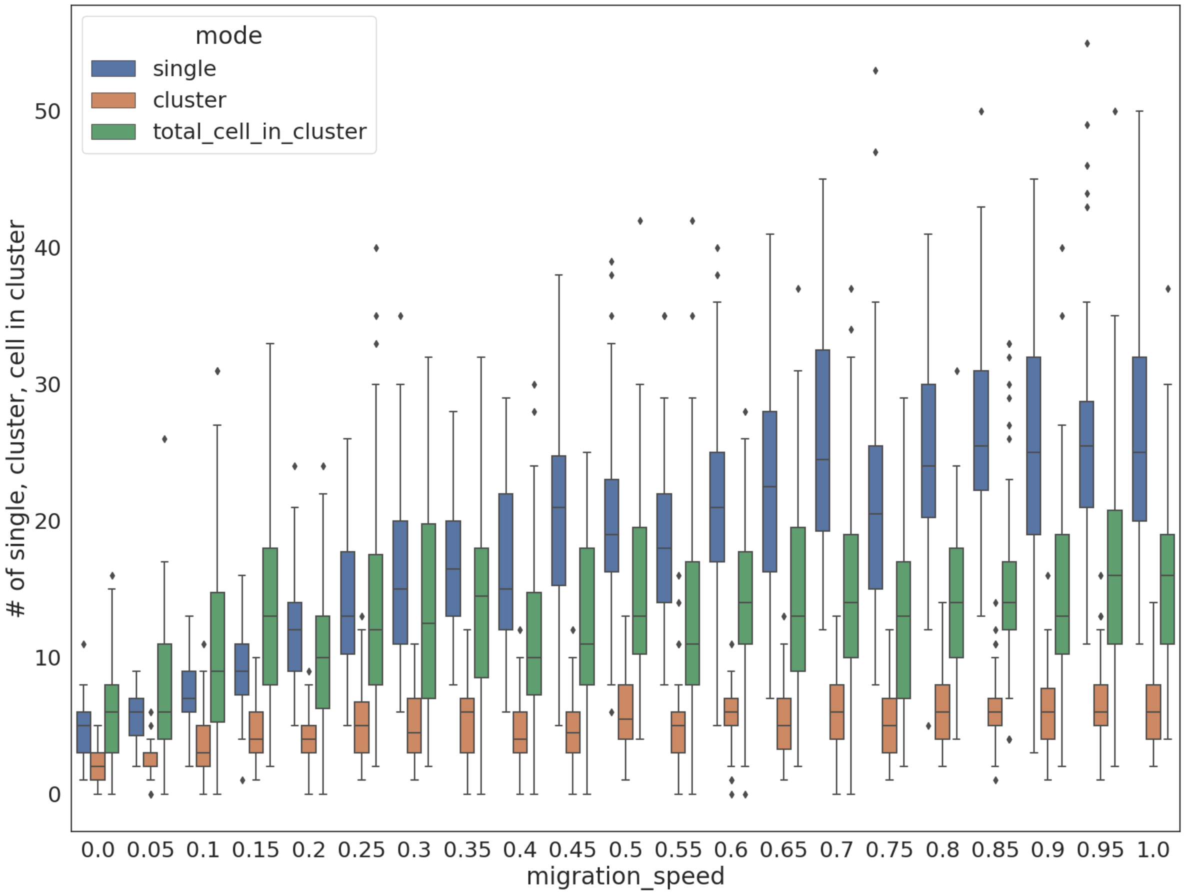

Figure S28: *Amount of single cell vs cell in cluster for different values of migration_speed*

This parameter regulates the velocity of a cell that migrates towards its chemoattractant source.

The analysis reveals that this parameter has minimal impact on the segregation of clusters and single cells. When values exceed 0.2, the number of cells migrating in clusters reaches a plateau. However, the number of single cells continues to increase until a value of 0.7, after which it stabilizes around 25. When migration_speed=0, cells do not move due

to oxygen attraction, but due to cellular replication-induced pushing, leading to homogeneous tumor growth. The number of recorded single cells results from the chosen quantification method.

16. Bibliography

[1] Cohen, David P A et al. "Mathematical Modelling of Molecular Pathways Enabling Tumour Cell Invasion and Migration." *PLoS computational biology* vol. 11,11 e1004571. 3 Nov. 2015, doi:10.1371/journal.pcbi.1004571

[2] Schwab LP, Peacock DL, Majumdar D, et al. Hypoxia-inducible factor 1α promotes primary tumor growth and tumor-initiating cell activity in breast cancer. *Breast Cancer Res*. 2012;14(1):R6. Published 2012 Jan 7. doi:10.1186/bcr3087

[3] Rausch V, Hansen CG. The Hippo Pathway, YAP/TAZ, and the Plasma Membrane. Trends Cell Biol. 2020 Jan;30(1):32-48. doi: 10.1016/j.tcb.2019.10.005. Epub 2019 Dec 2. PMID: 31806419.

[4] Lim ST, Chen XL, Lim Y, et al. Nuclear FAK promotes cell proliferation and survival through FERM-enhanced p53 degradation. Mol Cell. 2008;29(1):9-22. doi:10.1016/j.molcel.2007.11.031

[5] Selvaggio G, Canato S, Pawar A, et al. Hybrid Epithelial-Mesenchymal Phenotypes Are Controlled by Microenvironmental Factors. *Cancer Res*. 2020;80(11):2407-2420. doi:10.1158/0008-5472.CAN-19-3147

[6] Parri, M., Chiarugi, P. Rac and Rho GTPases in cancer cell motility control. *Cell Commun Signal* **8**, 23 (2010). https://doi.org/10.1186/1478-811X-8-23

[7] Jung H, Yoon SR, Lim J, Cho HJ, Lee HG. Dysregulation of Rho GTPases in Human Cancers. Cancers (Basel). 2020;12(5):1179. Published 2020 May 7. doi:10.3390/cancers12051179

[8] Zhao B, Tumaneng K, Guan KL. The Hippo pathway in organ size control, tissue regeneration and stem cell self-renewal. *Nat Cell Biol*. 2011;13(8):877-883. Published 2011 Aug 1. doi:10.1038/ncb2303

[9] Moitrier, S., Pricoupenko, N., Kerjouan, A. et al. Local light-activation of the Src oncoprotein in an epithelial monolayer promotes collective extrusion. Commun Phys 2, 98 (2019). https://doi.org/10.1038/s42005-019-0198-5

[10] Lodillinsky C, Infante E, Guichard A, et al. p63/MT1-MMP axis is required for in situ to invasive transition in basal-like breast cancer. Oncogene. 2016;35(3):344-357. doi:10.1038/onc.2015.87

[11]Hauck, Christof R., Datsun A. Hsia, et David D. Schlaepfer. « The Focal Adhesion Kinase--A Regulator of Cell Migration and Invasion ». IUBMB Life (International Union of Biochemistry and Molecular Biology: Life) 53, no 2 (1 février 2002): 115‑19. https://doi.org/10.1080/15216540211470.

[12]Kerjouan, Adèle, Cyril Boyault, Christiane Oddou, Edwige Hiriart-Bryant, Alexei Grichine, Alexandra Kraut, Mylène Pezet, et al. « Control of SRC Molecular Dynamics Encodes Distinct Cytoskeletal Responses by Specifying Signaling Pathway Usage ». Journal of Cell Science 134, no 2 (15 janvier 2021): jcs254599. https://doi.org/10.1242/jcs.254599.

[13]Parri, Matteo, et Paola Chiarugi. « Rac and Rho GTPases in Cancer Cell Motility Control ». Cell Communication and Signaling 8, no 1 (décembre 2010): 23. https://doi.org/10.1186/1478-811X-8-23.

[14]Jung, Haiyoung, Suk Ran Yoon, Jeewon Lim, Hee Jun Cho, et Hee Gu Lee. « Dysregulation of Rho GTPases in Human Cancers ». Cancers 12, no 5 (7 mai 2020): 1179. https://doi.org/10.3390/cancers12051179.

[15]Plou, J., Y. Juste-Lanas, V. Olivares, C. del Amo, C. Borau, et J. M. García-Aznar. « From Individual to Collective 3D Cancer Dissemination: Roles of Collagen Concentration and TGF-β ». Scientific Reports 8, no 1 (décembre 2018): 12723. https://doi.org/10.1038/s41598-018-30683-4.

[16]Colom, Adai, Roland Galgoczy, Isaac Almendros, Antonio Xaubet, Ramon Farré, et Jordi Alcaraz. « Oxygen Diffusion and Consumption in Extracellular Matrix Gels: Implications for Designing Three-Dimensional Cultures: Implications For Designing Three-Dimensional Cultures ». Journal of Biomedical Materials Research Part A 102, no 8 (août 2014): 2776‑84. https://doi.org/10.1002/jbm.a.34946.
[17]Ilina, Olga, Pavlo G. Gritsenko, Simon Syga, Jürgen Lippoldt, Caterina A. M. La Porta, Oleksandr Chepizhko, Steffen Grosser, et al. « Cell–Cell Adhesion and 3D Matrix Confinement Determine Jamming Transitions in Breast Cancer Invasion ». Nature Cell Biology 22, no 9 (septembre 2020): 1103‑15. https://doi.org/10.1038/s41556-020-0552-6.
[18]Ghaffarizadeh, Ahmadreza, Randy Heiland, Samuel H. Friedman, Shannon M. Mumenthaler, et Paul Macklin. « PhysiCell: An Open Source Physics-Based Cell Simulator for 3-D Multicellular Systems ». PLOS Computational Biology 14, no 2 (23 février 2018): e1005991. https://doi.org/10.1371/journal.pcbi.1005991.
